# A Prebiotic Precursor to Life’s Phosphate Transfer System with an ATP Analog and Histidyl Peptide Organocatalysts

**DOI:** 10.1021/jacs.4c01156

**Published:** 2024-03-06

**Authors:** Oliver R. Maguire, Iris B. A. Smokers, Bob G. Oosterom, Alla Zheliezniak, Wilhelm T. S. Huck

**Affiliations:** Institute for Molecules and Materials, Radboud University Nijmegen, Heyendaalseweg 135, Nijmegen AJ 6525, The Netherlands

## Abstract

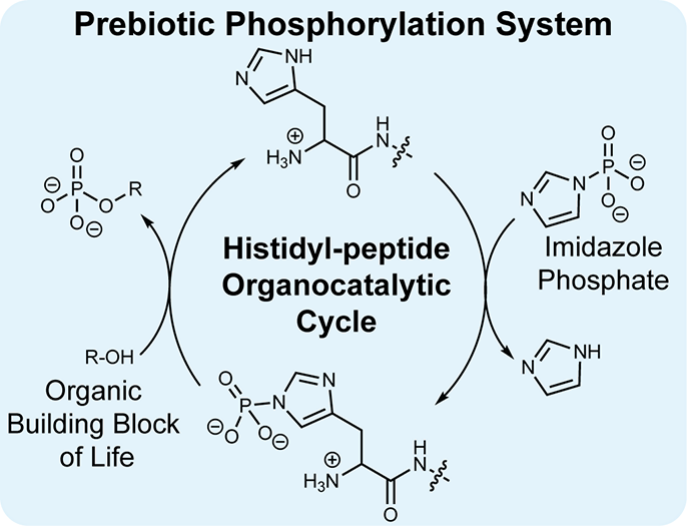

Biochemistry is dependent upon enzyme catalysts accelerating key reactions. At the origin of life, prebiotic chemistry must have incorporated catalytic reactions. While this would have yielded much needed amplification of certain reaction products, it would come at the possible cost of rapidly depleting the high energy molecules that acted as chemical fuels. Biochemistry solves this problem by combining kinetically stable and thermodynamically activated molecules (e.g., ATP) with enzyme catalysts. Here, we demonstrate a prebiotic phosphate transfer system involving an ATP analog (imidazole phosphate) and histidyl peptides, which function as organocatalytic enzyme analogs. We demonstrate that histidyl peptides catalyze phosphorylations via a phosphorylated histidyl intermediate. We integrate these histidyl-catalyzed phosphorylations into a complete prebiotic scenario whereby inorganic phosphate is incorporated into organic compounds though physicochemical wet–dry cycles. Our work demonstrates a plausible system for the catalyzed production of phosphorylated compounds on the early Earth and how organocatalytic peptides, as enzyme precursors, could have played an important role in this.

## 1 Introduction

Understanding the origins of life remains one of Science’s greatest challenges. Enormous progress has been made in discovering plausible prebiotic reaction pathways to the building blocks of life.^1−7^ Nevertheless, with the increasing complexity of the chemical pathways that could have supported the origins of life comes the realization that prebiotic chemistry lacks the required selectivity and control over reaction rates to avoid the formation of a wide range of undesired molecules. Some form of catalysis must have been incorporated in prebiotic systems at an early stage in order to direct the reaction outcomes. Indeed, the RNA world scenario relies heavily upon the dual role of RNA as a catalyst as well as an information storage polymer.^8,9^ Furthermore, various transition metal ions and mineral surfaces have been widely used for their catalytic potential.^6,7,10,11^

**Figure 1:**
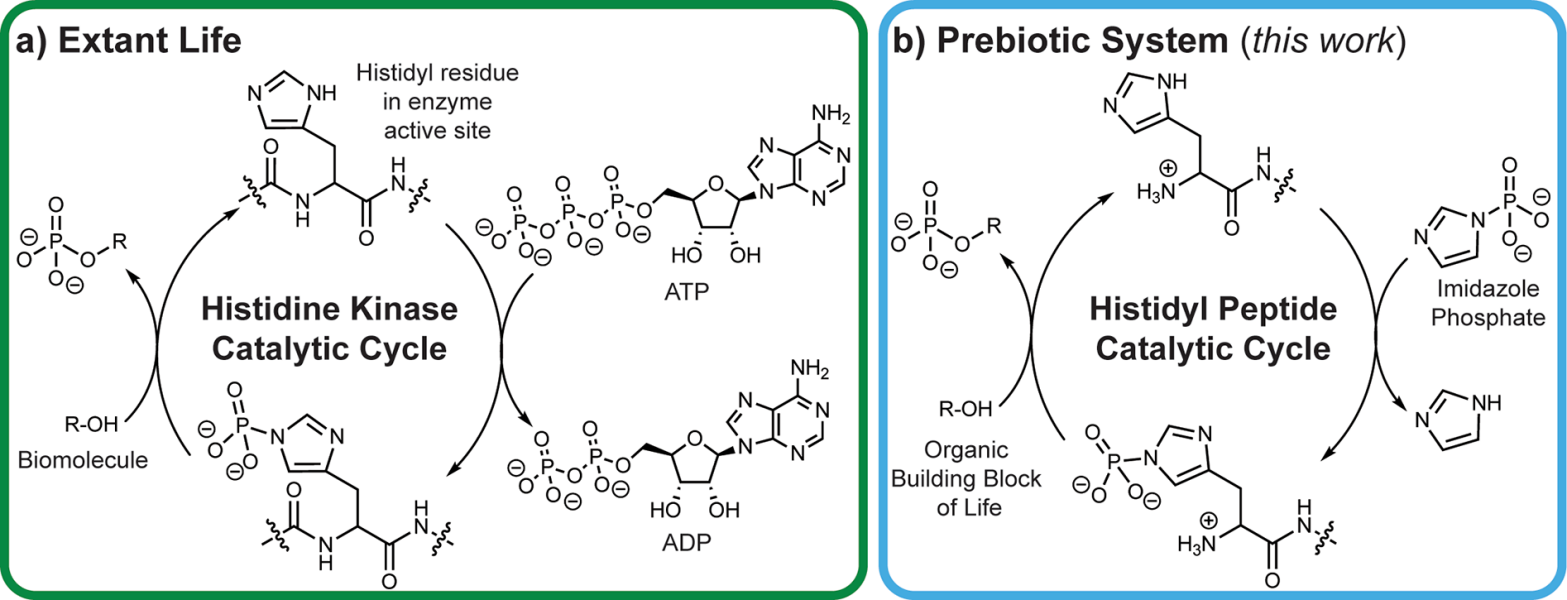
(a) The phosphate transfer system used by life with ATP as a kinetically stable and thermodynamically activated source of phosphate and enzymes, such as histidine kinases, which catalyze phosphorylation reactions to a nucleophilic biomolecule (R–OH). (b) The prebiotic phosphate transfer system demonstrated here with imidazole phosphate as a kinetically stable and thermodynamically activated phosphate source and a histidyl peptide as a prebiotic organocatalyst for the phosphorylation of organic building blocks of life (R–OH).

However, with catalysts accelerating reactions, primordial systems would have risked the rapid dissipation of high energy molecules. We are inspired by the way extant biology controls the flux of energy dissipation through the combination of kinetically stable and thermodynamically activated (KSTA) compounds (e.g., ATP) with enzyme catalysts (e.g., histidine kinases). The histidine kinase catalytic cycle proceeds via a phosphorylated histidyl residue in the active site (Figure 1a). The kinetic stability of KSTA compounds ensures that the chemical potential held within the molecule is not dissipated in deleterious side reactions.^12^ This is exemplified by the kinetic stability of ATP, which has a hydrolysis half-life of 2 years at 25 °C and pH 8.^13^ This strategy of using KSTA compounds with enzyme catalysts is key to directing reaction outcomes in cellular biochemical networks.

We recently reported a prebiotically plausible KSTA ATP analog in the form of imidazole phosphate.^14^ Imidazole phosphate forms under the mildest of conditions (pH 7.3, 22 °C) from a solution of prebiotically plausible reagents cyanate, orthophosphate, and imidazole.^15−19^ Like ATP, imidazole phosphate has a good hydrolytic stability and is thus accumulated in solution. Phosphate transfer from imidazole phosphate to organic compounds does not function in solution as the activity of water is too high.^14^ However, after a wet-to-dry transition from a solution to a water-depleted paste, the phosphate transfer reaction from imidazole phosphate to all the basic building blocks of life occurs. This physicochemical orthophosphate cycle could have provided a continuous supply of phosphorylated organic compounds for prebiotic chemistry in the early Earth. We now address the challenge of combining this cycle with a prebiotic form of catalysis.

An enzyme catalyst for phosphate transfer is clearly not compatible with prebiotic chemistry. However, peptides on the early Earth are considered to be plausible prebiotic organocatalysts and this includes histidyl peptides.^20,21^ Prebiotic syntheses of histidine and peptides with histidyl residues are known.^22−27^ Histidyl-based peptides have shown promise as prebiotic organocatalysts. His–His can catalyze the oligomerization of peptides, the dephosphorylation of nucleotides, the hydrolysis of oligonucleotides, and the oligomerization reaction with c2,3-nucleotides.^24,28−30^ His–His is proposed to derive its catalytic activity from a general acid–base catalysis by a proton relay mechanism. Ser-His is another prebiotic organocatalyst that catalyzes a wide range of reactions, e.g., peptide bond formation between an amino acid ester and an amino acid.^31−33^ This was exploited to drive vesicle growth via Ser-His localizing inside the membrane of vesicles and catalyzing the formation of a hydrophobic dipeptide.^34^ Ser-His can catalyze the formation of oligonucleotides starting from imidazole-activated nucleotides.^35^ Histidyl-containing cyclic peptides catalyze also the hydrolysis of pyrophosphate.^36^ Histidine may also ligate metals, e.g., Zn^2+^, to form complexes that catalyze depsipeptide oligomerization.^37^ Phosphorylated histidyl peptides have been shown to undergo liquid–liquid phase separation to form coacervates.^38^ In our aforementioned physicochemical orthophosphate cycle, we hypothesized that a histidyl peptide could serve as a catalyst for phosphorylations with imidazole phosphate given the similarity in the chemical structure between imidazole and histidine.

Phosphorylation is one of life’s most important reactions. It is a key requirement for the realization of many of life’s essential features such as compartmentalization (phospholipids), preservation of genetic information (DNA’s phosphodiester backbone), and the prime energy transfer process to drive endergonic reactions (nucleotide triphosphates, e.g., ATP).^39,40^ The incorporation of phosphate chemistry into prebiotic systems is thus seen as an essential requirement for the emergence of life.^17,41−47^

Here, we demonstrate the construction of a prebiotically plausible phosphate transfer system that combines the formation of a KSTA ATP analog with histidyl peptide organocatalysis (Figure 1b). We show first that histidyl peptides catalyze the phosphate transfer of imidazole phosphate to water (i.e., hydrolysis) via the formation of a phosphorylated histidyl intermediate and quantify rate constants for this reaction. We then demonstrate that in a paste, histidyl peptides catalyze the phosphate transfer from imidazole phosphate to organic molecules via the phosphorylated histidyl intermediate. We explore how the amino acid sequence in the histidyl peptide catalyst affects its catalytic activity. Finally, we demonstrate a full physicochemical orthophosphate cycle in the presence of histidyl peptide catalysts, which incorporates both the *in situ* formation of the KSTA imidazole phosphate and histidyl peptide-catalyzed phosphorylations.

## 2 Results

### 2.1 Histidyl Peptides Catalyze the Hydrolysis of Imidazole Phosphate via a Phosphorylated Histidyl Intermediate

We sought a prebiotically plausible catalyst to overcome the kinetic stability of imidazole phosphate and thereby control the release of energy in the phosphate transfer reaction. We noted that in extant life, histidine kinases catalyze phosphorylation reactions.^48−55^ We reasoned that histidine or a histidyl residue could be a plausible prebiotic organocatalyst to overcome the kinetic stability of imidazole phosphate due to the structural similarity between imidazole and the imidazolyl ring on histidine. In our previous study on the formation of imidazole phosphate, we observed that in water, imidazole phosphate slowly hydrolyzed over time.^14^ For comparison, the uncatalyzed hydrolysis half-life for imidazole phosphate is *t*_1/2_ = 23.1 h at pH 7.0 and 40.1 °C,^56^ for ATP *t*_1/2_ = 73 days at pH 8.0 and 39 °C,^13^ and for carbamoyl phosphate *t*_1/2_ = 40 min at pH 7.2 and 37 °C.^57^ Thus, imidazole phosphate is less kinetically stable than ATP but more kinetically stable than carbamoyl phosphate. To test whether histidyl peptides act as catalysts for phosphate transfer, we chose to study the hydrolysis of imidazole phosphate.

**Figure 2:**
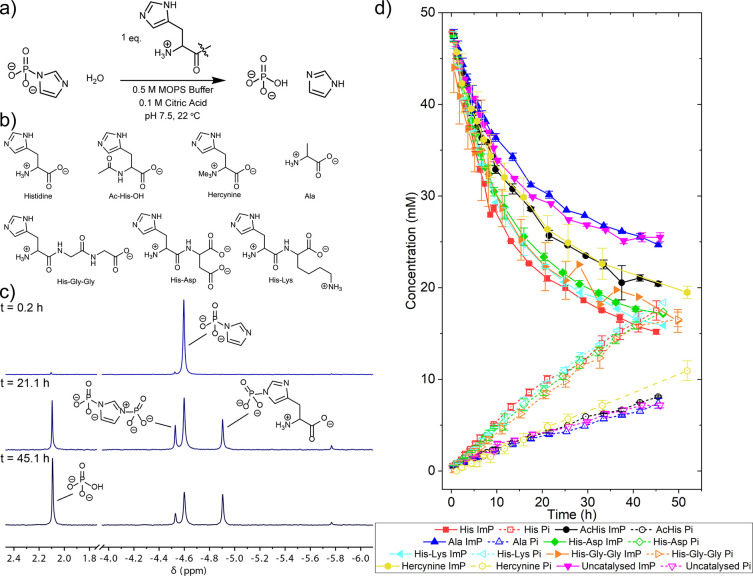
Histidyls catalyze the hydrolysis of imidazole phosphate. (a) Reaction overview and conditions. (b) The histidyl amino acid and peptide residues used to study the hydrolysis reaction. (c) Representative ^31^P NMR spectra over time for the reaction of 50 mM imidazole phosphate (Ca^2+^ salt) and 50 mM of histidine catalyst in 0.5 M MOPS buffer and 0.1 M citric acid at pH 7.5 and 22 °C. (d) Changes in concentration of imidazole phosphate (solid lines) and the hydrolysis product orthophosphate (dashed lines) over time. The reactions were repeated in duplicate or triplicate—data points are the mean value, and error bars are the standard deviation. ImP = imidazole phosphate, Pi = orthophosphate.

We monitored the hydrolysis of imidazole phosphate in the presence and absence of histidyls to determine whether or not the hydrolysis was catalyzed (Figure 2a and Section S2). First, we followed the uncatalyzed background hydrolysis of imidazole phosphate using ^31^P NMR spectroscopy with 50 mM calcium imidazole phosphate in 0.5 M MOPS buffer and 0.1 M citric acid and 50 mM HMPA internal standard at pH 7.5 and 22 °C (Section S2.10). The citric acid was added to prevent precipitation of calcium phosphate. Under these conditions, the imidazole phosphate (ImP) slowly hydrolyzed to orthophosphate (Pi) over a period of 48 h (Figure 2d, filled and open pink inverted triangle traces). Formation of diphosphoimidazole from the reaction of imidazole phosphate with itself was also observed (Figure S16), and this accounts for the difference in concentration between imidazole phosphate and orthophosphate in Figure 2d (Figure S17). To determine the reaction rate constants for the hydrolysis of imidazole phosphate, a reaction kinetics scheme was fitted to the experimental data (Figures S29 and S30). The first-order rate constant for the hydrolysis of imidazole phosphate was determined to be *k*_hyd,ImP_ = 3.9 × 10^–7^ s^–1^ (Table S14). The first-order rate constant for the hydrolysis of diphosphoimidazole (DPI) was an order of magnitude larger at *k*_hyd,DPI_ = 8.6 × 10^–6^ s^–1^ (Table S14).

We next followed the hydrolysis of imidazole phosphate in the presence of 50 mM histidine under identical conditions to see if histidine catalyzed the reaction. A representative set of ^31^P NMR spectra over time is shown in Figure 2c. At the start, the only phosphorylated species present in solution is imidazole phosphate (δ 4.65 ppm). Over time, a phosphorylated histidine intermediate (δ 4.92 ppm) is formed. We confirmed with ^1^H ^31^P HMBC NMR spectroscopy that this phosphate is located on the imidazolyl ring of the histidine residue (Figure S3). DPI (δ 4.55 ppm) is also formed over time. The concentration of the hydrolysis product orthophosphate (δ 2.10 ppm) increases over time. In the presence of the histidine, the hydrolysis reaction was accelerated compared to the uncatalyzed reaction, as seen in both the accelerated disappearance of imidazole phosphate and the accelerated appearance of orthophosphate (Figure 2d, filled and open red square traces). A reaction kinetics scheme for the histidyl-catalyzed hydrolysis of imidazole phosphate was again fitted to the experimental data (Figure S29 and S30). The first-order rate constant for the hydrolysis of the phosphorylated histidine intermediate (PHis) was determined to be *k*_hyd,PHis_ = 1.4 × 10^–5^ s^–1^ (Table S14)—a value that is approximately two orders of magnitude higher than that of the background hydrolysis of imidazole phosphate. This therefore confirmed that histidine catalyzed the hydrolysis reaction.

Having observed the catalyzed hydrolysis of imidazole phosphate, we next sought to confirm that the phosphorylated histidyl was the catalytic species in the reaction. In principle, the catalysis could be via either the formation of a phosphorylated imidazolyl histidine (observed in ^31^P NMR spectra) or via an N-terminal phosphoramidate (not observed in our ^31^P NMR spectra for histidine; however, this species forms in <0.5% yield for His-Asp, His-Lys, and His-Gly-Gly, see representative NMR spectra in Section S2). To determine if the catalysis proceeded via an N-terminal phosphoramidate, we performed the hydrolysis reaction in the presence of 50 mM alanine (Figure 2d, filled and open blue triangle traces). No catalysis of the hydrolysis reaction was observed, and the disappearance of imidazole phosphate and the appearance of orthophosphate overlaid well with the uncatalyzed reaction. Admittedly, the formation of the N-terminal phosphoramidate is unlikely, as at pH 7.5, the N-terminus is found as the ammonium species and thus the lone pair on nitrogen is unavailable to act as a nucleophile.

Having confirmed that an N-terminal phosphoramidate is not the main catalytic species and thus the catalysis must arise from the imidazolyl ring on histidine (catalysis of phosphate ester hydrolysis by imidazolyl rings are known in the literature^58^), we next sought to see if catalysis by the imidazolyl ring acted alone or in concert with the ammonium at the N-terminus. We performed the hydrolysis reaction in the presence of 50 mM acetylated N-terminal histidine (Figure 2b and d, filled and open black circle traces). Here, the disappearance of imidazole phosphate was accelerated and a phosphorylated acetyl histidine imidazolyl was formed (Figure S4). However, no acceleration in the formation of orthophosphate was observed and the phosphate remained trapped upon the histidyl. Thus, in the absence of a protonated amino acid N-terminal, no catalysis of hydrolysis was observed. To further assess the importance of the cationic N-terminus, we performed the reaction with hercynine—a histidine with an N-terminal −NMe_3_^+^ group. Here, we observed again the accelerated disappearance of imidazole phosphate but orthophosphate formation was not accelerated compared to the background (Figure 2d, filled and open yellow hexagon traces). The absence of catalysis in the presence of a cationic trimethylated N-terminal suggests that the hydrolysis transition state may be stabilized by the formation of an intramolecular H-bond between the cationic ammonium N-terminal and the departing phosphate group. This is in addition to any stability conferred via favorable electrostatic interactions between the two groups. Therefore, the combination of the imidazolyl ring and the N-terminal ammonium is required to catalyze the reaction.

Finally, to explore whether catalysis by histidyls is general, we performed the hydrolysis experiments in the presence of several histidyl residue-containing peptides: His-Asp, His-Lys, and His-Gly-Gly. In all cases, the hydrolysis of imidazole phosphate was catalyzed (Figure 2d, His-Asp, filled and open green diamonds; His-Lys, left-pointing filled and open turquoise triangles; His-Gly-Gly, right-pointing filled and open orange triangles). The first-order rate constant for the hydrolysis of the phosphorylated histidine intermediates (*k*_hyd,PHis_) is of similar value to those observed for histidine (His-Asp *k*_hyd,PHis_ = 1.7 × 10^–5^ s^–1^, His-Lys *k*_hyd,PHis_ = 1.7 × 10^–5^ s^–1^, and His-Gly-Gly *k*_hyd,PHis_ = 2.1 × 10^–5^ s^–1^, Table S14).

### 2.2 Histidyl Peptides Catalyze the Phosphorylations of Organic Molecules in a Wet-to-Dry Transition

In our previous study, we found that phosphate transfer from imidazole phosphate to organic compounds occurred in water-depleted pastes but not in bulk solution. This is due to the high activity of water in solution, which outcompetes organic nucleophiles for the phosphate.^14^ Such wet-to-dry transitions are deemed to be prebiotically plausible—akin to a puddle or pond drying.^59−62^ Here, we also use water-depleted paste conditions to facilitate phosphate transfer to organic nucleophiles in the presence of histidyl peptide catalysts.

**Figure 3:**
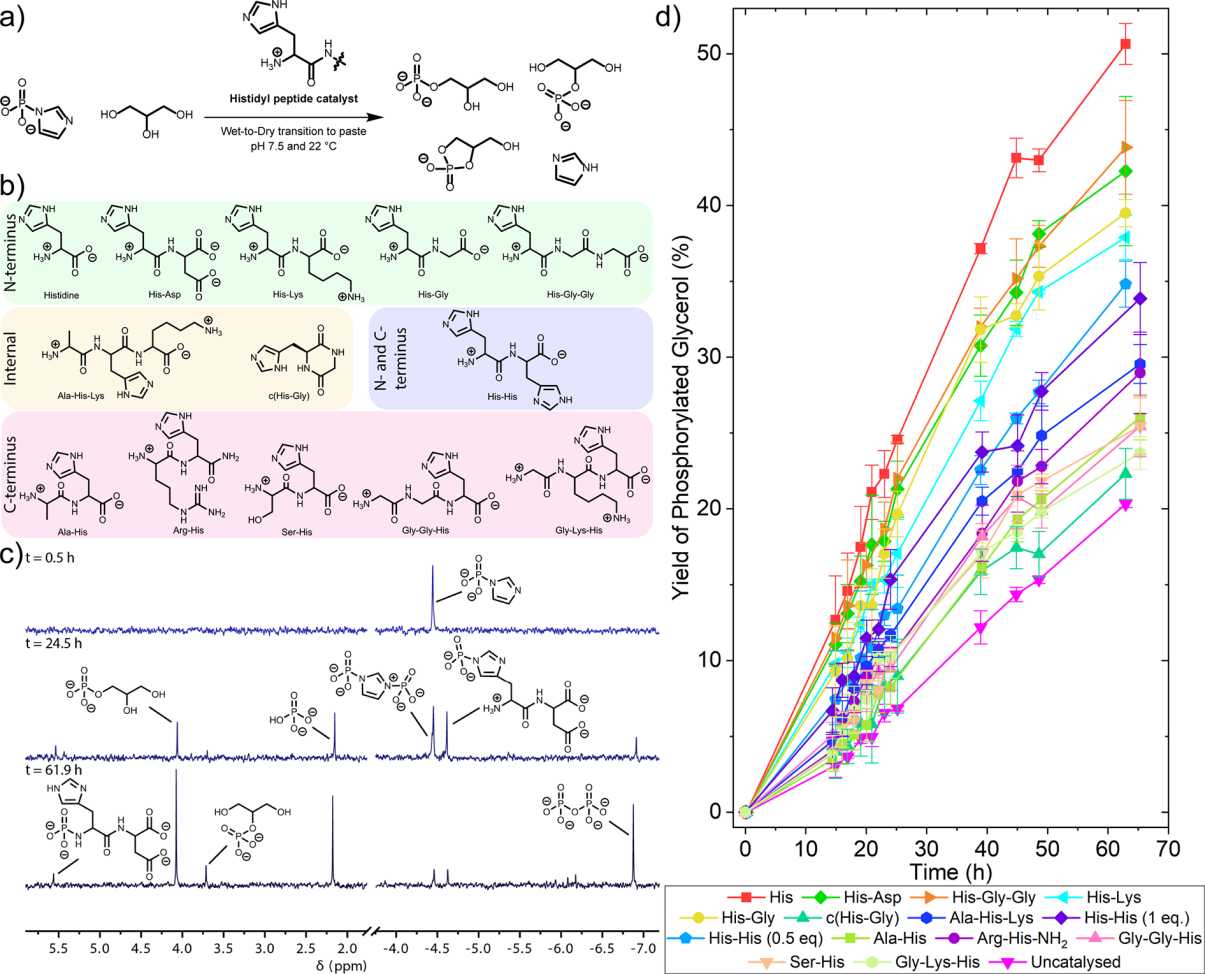
Histidyl residues catalyze the phosphate transfer reaction from imidazole phosphate to glycerol. (a) Reaction overview and conditions. (b) The histidyl amino acid and peptide organocatalysts studied. (c) Representative ^31^P NMR spectra over time for the reaction of 0.13 mmol imidazole phosphate, 3.25 mmol of glycerol, and 0.13 mmol His-Asp at pH 7.5 and 22 °C. (d) Changes in yield of phosphorylated glycerol over time. The yields of all phosphorylated glycerol species (glycerol-1-phosphate, glycerol-2-phopshate, and cyclo-glycerol-1,2-phosphate) are summated. The reactions were repeated in duplicate or triplicate—data points are the mean value, and error bars are the standard deviation.

We used glycerol as a model substrate to assess how the structure of histidyl peptides affects their catalytic performance. Phosphorylated glycerol is an amphiphile precursor required for the formation of membranes and compartmentalization.^63,64^ The phosphorylations were performed by preparing aqueous solutions containing the amphiphile precursor glycerol, calcium imidazole phosphate, and a histidyl peptide at pH 7.5 (Figure 3a and Section S3). The solution was evaporated to a paste by leaving the solution open to the air at 22 °C. The reaction was followed for 62 h by the periodic removal of samples from the paste. Samples were dissolved in a 0.5 M citric acid buffer at pH 6.85 with an HMPA internal standard and analyzed by ^31^P NMR spectroscopy. The yield of phosphorylated glycerol at each time point was determined by summating together the yield of glycerol-1-phosphate, glycerol-2-phopshate, and cyclo-glycerol-1,2-phosphate.

To observe whether or not phosphate transfer to glycerol is catalyzed by histidyl peptides, we used the uncatalyzed reaction for comparison. An aqueous solution of 65 mM imidazole phosphate and 1625 mM glycerol nucleophile at pH 7.5 was dried to a paste at 22 °C (Section S3.8). NB: these concentrations are prebiotically relevant if we consider that in drying puddles, the concentrations can become arbitrarily high. In the paste, glycerol was steadily phosphorylated and reached a yield of 20.3 ± 0.2% over a period of 62 h (Figure 3d, filled magenta inverted triangle trace). Next, we performed the reaction in the presence of a 65 mM His-Asp peptide. In the ^31^P NMR spectra, we observed the formation of the phosphorylated histidyl intermediate (Figure 3c, δ 4.62 ppm) (*in situ* NMR characterization of the phosphorylated intermediate is in Sections S2.6.1 and S5.3). Encouragingly, we observed that the formation of phosphorylated glycerol products was accelerated in the presence of His-Asp compared to the uncatalyzed reaction. This is seen from the steeper increase in the formation of phosphorylated glycerol over time (Figure 3d, filled green diamond trace). The yield of phosphorylated glycerol was 42.3 ± 4.9% after 62 h—double the yield of the uncatalyzed reaction.

In our previous study, we heated the pastes to 50 °C with 5 eq. of glycerol, which gave a glycerol phosphate yield of 35% after 48 h.^14^ Here, at 22 °C with 5 equiv of glycerol, the uncatalyzed reaction has a glycerol phosphate yield of 7.5% after 41 h while the histidine-catalyzed reaction has a 17% yield of glycerol phosphate at 41 h (Section S3.20). In comparison to our previous study, lowering the temperature by 28 °C in the uncatalyzed reaction results in a 4.7-fold drop in the glycerol phosphate yield. However, in the presence of the histidine catalyst, the yield of glycerol phosphate only drops 2.1-fold despite being 28 °C lower in temperature.

We explored how the structure of the histidyl peptide catalyst affected its ability to catalyze the phosphate transfer reaction. We performed the reaction with a range of different histidyl peptides, which had histidyl residues at either the N-terminus, in the center of the peptide chain or at the C-terminus (Figure 3b, Sections S3.2–S3.16). In all cases, the phosphorylation was catalyzed but the catalytic activity varied depending upon the position of the histidyl residue in the peptide chain (Figure 3d). The general trend in catalytic performance was that N-terminus > center > C-terminus histidyls. The closer the histidyl is in proximity to the cationic ammonium N-terminal, the greater the acceleration in the phosphate transfer reaction. This is consistent with the results from the hydrolysis experiments above. In these reactions, the amino acid histidine proved to be the most effective catalyst for transferring phosphate to glycerol.

The histidyl-catalyzed phosphate transfer proceeded again via the formation of a phosphorylated imidazolyl histidyl intermediate. This intermediate was observed in the ^31^P NMR spectra (Figure 3c) and formed typically in a 30–35% yield after 16 h. Unlike in the hydrolysis reactions above, we did observe formation of a minor amount (typically in *a* < 5% yield after 16 h) of N-terminal histidyl phosphoramidate (Figure 3c, δ 5.62 ppm). Given that, in all cases, the yield of the phosphorylated imidazolyl intermediate significantly exceeded that of the N-terminal phosphoramidates and our above results on hydrolysis, we consider that the phosphorylated imidazolyl intermediate is the most catalytically relevant species. The results from the wet-to-dry experiments displayed good repeatability in the yield of glycerol phosphate at each time point. A histogram (Figure S142) of the standard deviations from all data points in Figure 3d shows that the standard deviation was typically <2.5%.

The histidyls also catalyze the formation of other phosphate-containing products. The products from these reactions include orthophosphate (from hydrolysis) and pyrophosphate (from phosphate anhydride bond formation) (Figure 3c). The formation of orthophosphate and pyrophosphate was catalyzed by histidyl peptides (Figures S140 and S141). In the above experiments, we used a 25-fold excess of the glycerol in order to direct phosphorylation predominantly onto the organic nucleophile. At lower excesses of glycerol of 5-fold and 10-fold (0.65 and 1.30 mmol, Sections S3.19 and S3.20), we still observed catalysis of phosphate transfer to glycerol (Figure S156). However, we also observed a broader range of phosphorylation reactions, which compete with the phosphorylation of the glycerol. The products from these reactions include orthophosphate, pyrophosphate, and triphosphate (Figure S143).

We performed a set of experiments starting from solutions made with 0.13 mmol of imidazole phosphate and 1.30 mmol of glycerol and with different equivalents of histidine catalyst (1.00, 0.75, 0.50, and 0.25 eq.). In all reactions, the phosphorylation of glycerol was accelerated (Figure S156). Importantly, at lower equivalents of histidine, the yield of phosphorylated glycerol was greater than would be expected if a histidine only participated in one catalytic cycle. For example, as shown in Figure S156, after 48.8 h the yield of glycerol phosphate is 29.0% for 1.00 eq. of histidine and 26.5% for 0.5 eq. of histidine (the uncatalyzed reaction has a yield of 12.2%). If all histidines only participated in a single catalytic cycle, then it would be expected that the yield for 0.50 eq. would be half that of 1.00 eq. This confirms that at least some of the histidyls turned over and participated in multiple catalytic cycles.

### A Physicochemical Orthophosphate Cycle with *In Situ* Formation of Imidazole Phosphate and Histidyl Peptide-Catalyzed Phosphorylations

**Figure 4:**
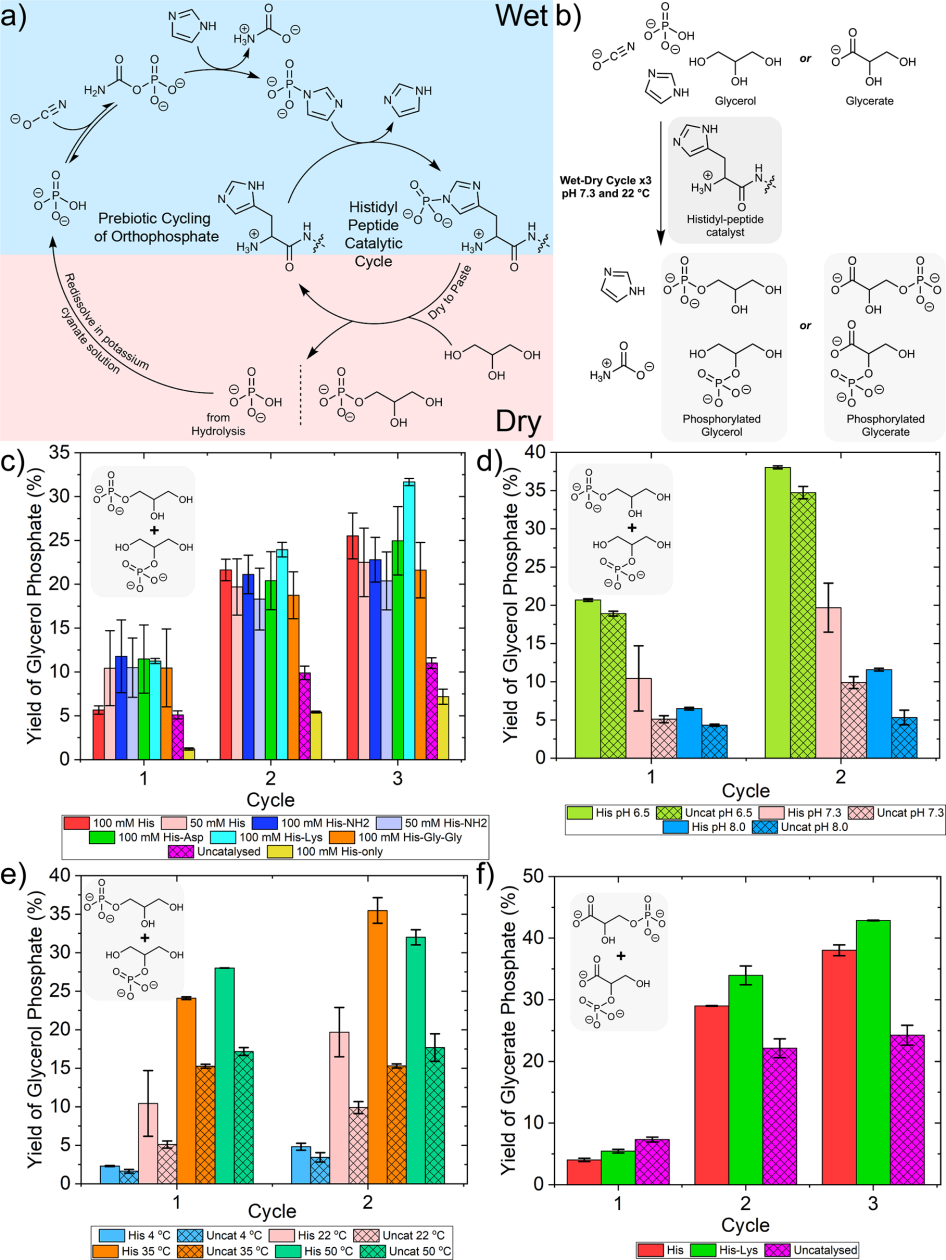
A physicochemical cycle for the histidyl peptide-catalyzed phosphorylation of glycerol and glycerate with *in situ* formation of imidazole phosphate. (a) Overview of the physicochemical cycle. Imidazole phosphate forms from a solution of orthophosphate, cyanate, and imidazole via carbamoyl phosphate. Transfer of the phosphate group to the histidyl peptide catalyst occurs in solution, as well as in the paste. Upon drying to a paste, the histidyl peptides catalyze the transfer of phosphate to glycerol/glycerate. Second and third cycles are performed by redissolving the paste in a potassium cyanate solution. (b) Reaction overview and conditions for the wet–dry cycles. (c) The percentage incorporation of orthophosphate into glycerol phosphate (summated from glycerol-1-phosphate and glycerol-2-phosphate) over three cycles—referred to as “Yield” on the *y*-axis. Experiments initiated from a solution of 20 mM sodium phosphate, 230 mM potassium cyanate, 100 M imidazole, 500 mM glycerol nucleophile, and 100 mM histidine/His-NH_2_/His-Asp/His-Lys/His-Gly-Gly or 50 mM histidine/His-NH_2_ at pH 7.3 and 22 °C. The results from the uncatalyzed reaction are shown. The experiment performed in the absence of imidazole with only 100 mM histidine catalyst present is also shown (His-only). The mean yields of glycerol phosphate based upon triplicate experiments are plotted, and error bars are the standard deviation. (d) The effect of pH on the histidine-catalyzed and uncatalyzed phosphorylation of glycerol over two cycles. Experiments were initiated from a solution of 20 mM sodium phosphate, 230 mM potassium cyanate, 100 M imidazole, 500 mM glycerol nucleophile, and 50 mM histidine at pH 6.5, pH 7.3, and pH 8.0 at 22 °C. (e) The effect of temperature on the histidine-catalyzed and uncatalyzed phosphorylation of glycerol during the dry stage of two wet/dry cycles. Experiments initiated from a solution of 20 mM sodium phosphate, 230 mM potassium cyanate, 100 M imidazole, 500 mM glycerol nucleophile, and 50 mM histidine at 4, 22, 35, and 50 °C. (f) The percentage incorporation of orthophosphate into glycerate phosphate (summated from glycerate-3-phosphate and glycerate-2-phosphate) over three cycles—referred to as “Yield” on the *y*-axis. Experiments initiated from a solution of 20 mM sodium phosphate, 230 mM potassium cyanate, and 100 M imidazole with 500 mM glycerol nucleophile and 100 mM histidine/His-Lys at pH 7.3 and 22 °C. The uncatalyzed reaction is also shown. Note that cycle 1 had a 24 h drying period, and cycles 2 and 3 had a 48 h drying period. The mean yields of glycerate phosphate based upon duplicate experiments are plotted, and error bars are the standard deviation.

Finally, we constructed a physicochemical orthophosphate cycle incorporating both *in situ* formation of the KSTA imidazole phosphate and histidyl organocatalysis. The cycle starts in solution, where orthophosphate is activated via a reaction with cyanate to form carbamoyl phosphate (Figure 4a). This then reacts with imidazole to form the KSTA imidazole phosphate, which traps the activated phosphate in a kinetically stable state. The formation of imidazole phosphate enables the activated phosphate to enter into the histidyl peptide catalytic cycle. The phosphate is transferred from the imidazole phosphate to the imidazolyl of the histidyl peptide catalyst to form a phosphorylated histidyl intermediate. Upon drying the solution to a paste, where the activity of water is lower, this phosphorylated histidyl intermediate accelerates the phosphorylation of the prebiotically important organic nucleophiles, e.g., glycerol. Note that both the formation of imidazole phosphate and the phosphorylated histidyl intermediate are aided by the wet-to-dry transition due to the increasing concentrations of all reactants as the solvent water evaporates. To complete the cycle, the paste is redissolved in a cyanate solution, which refuels the system. The remaining orthophosphate can then be converted into imidazole phosphate and later transferred onward onto the organic nucleophile. Multiple passes through the physicochemical cycle lead to the accumulation of phosphorylated compounds. We demonstrated previously that carbamoyl phosphate does not phosphorylate organic compounds in the wet/dry cycles.^14^

To demonstrate this physicochemical orthophosphate cycle with histidyl organocatalysis, we prepared solutions of 20 mM sodium phosphate, 230 mM potassium cyanate, and 100 M imidazole with 500 mM glycerol nucleophile, in both the presence and absence of 100 mM histidyl catalyst at pH 7.3 and 22 °C (Section S4). The solutions were left for 24 h in order to allow carbamoyl phosphate and imidazole phosphate to accumulate. To facilitate the histidyl-catalyzed phosphate transfer to glycerol, we dried the solutions down into paste by leaving them to evaporate in the open air for 48 h at 22 °C. To demonstrate that this cycle could function repeatedly, we dissolved the paste back into 230 mM potassium cyanate solution at pH 7.3 and then repeated the aforementioned procedure.

The histidyl peptides catalyzed the phosphorylation of glycerol over the course of three wet–dry cycles (Figure 4c). We tested a range of histidyl peptide-based catalysts with an N-terminal histidyl residue including histidine, His-NH_2_, His-Asp, His-Lys, and His-Gly-Gly. We also tested the reaction with lower catalyst concentrations of 50 mM for both histidine and His-NH_2_. In the first cycle, the yield of phosphorylated glycerol in the histidyl catalyzed is 11 ± 0.5%, for the majority of histidyl catalysts with the exception of 100 mM histidine, which has a yield of 5.7 ± 0.5% (red bar). The catalyzed yield is double that of the uncatalyzed reaction, which has a yield of 5.1 ± 0.5% (magenta bar). In the second cycle, the yield of glycerol phosphate from all the histidyl-catalyzed reactions, including 100 mM histidine, is 20.5 ± 1.0%. For the uncatalyzed reaction, the yield is 9.9 ± 0.8%. Thus, in the second cycle, all histidyl peptides produce double the yield of glycerol phosphate compared to the uncatalyzed reaction. For the third cycle, the yield in the histidyl-catalyzed reaction remains higher than the uncatalyzed reaction (24.2 ± 3.5% vs 11.0 ± 0.6%), although the increase in yield per cycle begins to level off.

We assessed in this system the importance of activating and trapping orthophosphate in the KSTA imidazole phosphate prior to the phosphorylation of the histidyl catalyst. It is conceivable that the histidyl peptides could be directly phosphorylated by carbamoyl phosphate, and we wished to explore how this affected the phosphorylation of glycerol. We performed the experiment under identical conditions with 100 mM histidine but without imidazole present (Figure 4c, tan bar, Section S4.10). Over the three cycles, the yields of phosphorylated glycerol were ∼4-fold lower per cycle than for the reactions where both histidine and imidazole are present (Figure 4c, tan vs red bars). The yields from this histidine-only reaction were lower even than those of the uncatalyzed reaction (Figure 4c, tan vs magenta bars). This result demonstrates the importance of the phosphorylation pathway proceeding via the formation of the KSTA imidazole phosphate rather than proceeding directly through the catalyst.

For all of the N-terminal histidyl peptides tested, the catalytic performance was not significantly affected by the identity of the adjacent amino acid residue (Figure 4c). His-Lys marginally outperforms the other histidyl peptides by the advent of the third cycle, although this is not substantial. Potentially, this is due to the additional positive charge from the Lys residue, which aids the phosphate transfer to glycerol by electrostatically stabilizing the transition state—although further mechanistic studies are required to determine this for certain.

In the case of 100 mM histidine, we presume that the absence of catalyzed phosphate transfer in the first cycle is due to histidine being both the most effective hydrolysis catalyst (Figure 2d) and the most effective phosphorylation catalyst in the paste (Figure 3d). In the first cycle, histidine’s role as a hydrolysis catalyst is to dominate. In the second cycle, histidine’s role as a phosphorylation catalyst that dominates and catalyzed glycerol phosphate formation was observed. We believe that this is a result of the outcome of the first cycle influencing the second cycle. At the end of the first cycle imidazole phosphate and phosphorylated histidine remain in the paste. The presence of these species in combination with the refueling of the reaction by cyanate leads to a higher yield of imidazole phosphate and phosphorylated histidine being present in the paste in the second cycle. This is seen in the higher yields at the end of the second cycle compared to the first cycle of imidazole phosphate (second cycle = 26.9 ± 0.6% vs first cycle = 14.3 ± 0.3%) and phosphorylated histidine (second cycle = 16.1 ± 1.8% vs first cycle = 7.1 ± 0.6%) (Figures S162, S164, and S166). Thus, a higher concentration of these species survives from solution into the paste and leads to a higher rate of phosphorylation of glycerol and hence the observed increase in yield of phosphorylated glycerol in the second and third cycles with 100 mM histidine.

We examined how a series of different environmental conditions affected the histidyl-catalyzed physicochemical orthophosphate cycle, including pH, temperature, and mineral surfaces. We performed both the histidine-catalyzed (50 mM) and uncatalyzed physicochemical orthophosphate cycles at pH 6.5, pH 7.3, and pH 8.0 (Figure 4d, Sections S4.21–S4.24). Histidine catalyzed the phosphorylation of glycerol at all pHs. In comparison to pH 7.3, the reactions at pH 6.5 gave a higher yield of phosphorylated glycerol (second cycle = 34.7 ± 0.2% at pH 6.5 vs 19.7 ± 3.2% at pH 7.3), although the difference between the histidine-catalyzed and uncatalyzed reactions is reduced. For pH 8.0, the yield of glycerol phosphate is lower than that at pH 7.3 (second cycle = 11.6 ± 0.2%). We also tested the effect of different temperatures (4, 22, 35, and 50 °C) upon the phosphorylation of glycerol in the pastes during the dry stage of the wet/dry cycles (Figure 4e, Sections S4.15–S4.20). At all temperatures, histidine (50 mM) catalyzed the phosphorylation of glycerol. At 22 °C, the yield of phosphorylated glycerol was 19.7 ± 3.2% after two cycles. At 4 °C, the yield was reduced to 4.8 ± 0.5% after the second cycle. At higher temperatures, the yield of phosphorylated glycerol exceeded that at 22 °C with yields of 35.5 ± 1.7% at 35 °C and 32.0 ± 1.0% at 50 °C. We also tested the histidyl-catalyzed physicochemical orthophosphate cycle in the presence of two minerals: montmorillonite and hydroxyapatite (Sections S4.25–S4.32 and Figures S255 and S264). In both cases, histidine catalyzed the phosphorylation of glycerol. Thus, the histidyl-catalyzed physicochemical orthophosphate cycle works across a broad range of possible prebiotic conditions.

Finally, we examined whether the histidyl peptides would also catalyze other substrates. We examined the phosphorylation of glycerate as 2-phosphoglycerate is a precursor in the formation of phosphoenolpyruvate—biology’s highest-energy phosphate.^65^ The physicochemical orthophosphate cycle in the presence of the histidyl catalysts histidine and His-Lys was performed starting from a solution of 20 mM sodium phosphate, 230 mM potassium cyanate, and 100 M imidazole with 500 mM glycerol nucleophile, in the presence and absence of 100 mM histidyl catalyst at pH 7.3 and 22 °C. In the first cycle, no catalysis of phosphorylated glycerate was observed (Figure 4d). However, in the second and third cycles, catalysis was observed after sufficient imidazole phosphate and phosphorylated histidine had accumulated.

## 3 Discussion

Here, we have demonstrated a plausible prebiotic precursor to life’s phosphate transfer system (Figure 4a). Our system is constructed by combining a KSTA ATP analog, i.e., imidazole phosphate, and a kinase enzyme analog, i.e., organocatalytic histidyl peptides. First, we showed that histidyl peptides catalyze the transfer of phosphate from imidazole phosphate with a set of hydrolysis reactions. This reaction proceeded via the formation of a phosphorylated imidazolyl histidyl—a less stable amidophosphate than imidazole phosphate. When we quantified the rate constants for this reaction, we determined that histidyl peptides increase the hydrolysis rate constant by two orders of magnitude. Next, we demonstrated that upon moving to a physical environment with a lower water activity by drying to a paste, the histidyl peptides catalyze the phosphorylation of the organic nucleophile glycerol. Peptides with an N-terminal histidyl residue were observed to be the best catalysts. Finally, we integrated the histidyl peptide-catalyzed phosphorylations into a complete prebiotic scenario for producing phosphorylated organic compounds. Here, orthophosphate was first activated and then kinetically trapped in the form of an imidazole phosphate. Upon drying to a paste, histidyl peptides catalyzed the phosphorylation of both glycerol and glycerate. Repeated cycling led to a stepwise increase in the yield of the phosphorylated organic compounds. This system demonstrates a plausible prebiotic route for the catalyzed production of phosphorylated compounds on the early Earth.

We have shown that histidyl peptide-catalyzed phosphate transfer reactions could have plausibly been present at the origins of life. This provides a new role for organocatalytic peptides at the origins of life. The organocatalytic activity of histidyl peptides could provide a possible reason as to why life eventually settled upon protein-based enzymes as the main type of catalyst for biochemical reactions.

All of our chemistry functions under the most benign of conditions at pH 7.3–7.5 and 22 °C. This is advantageous as unlike many other prebiotic methods for phosphate transfer, no heating or other aggressive physical or chemical conditions are required.^1,66,67^ Furthermore, we demonstrated that the system works across a range of prebiotically plausible environmental conditions. The pH of the ocean on the early Earth is estimated to range between pH 6.0 and 7.5,^68,69^ and the system here is consistent with these estimates as it works in the pH range 6.5–8.0. The wet/dry cycles could, for example, have potentially occurred at the ocean shoreline during high and low tides. We demonstrated that the histidyl-catalyzed phosphorylations function across a range of different temperatures 4–50 °C and in the presence of different mineral surfaces.

Life’s phosphate transfer system (1) activates orthophosphate, (2) traps it (mainly) in kinetically stable and thermodynamically activated ATP, and then (3) phosphorylates organic molecules using enzyme catalysts. Inspired by this general strategy, we have demonstrated here a prebiotic precursor that uses a similar strategy whereby orthophosphate is activated and trapped in the KSTA imidazole phosphate and then a histidyl peptide organocatalyst phosphorylates organic molecules.

A key future challenge for prebiotic chemistry is to demonstrate a phosphate transfer system (including activation of orthophosphate, trapping in a KSTA molecule, and catalyzed phosphorylation), which is fully functional in solution. Individual steps of a phosphate transfer system have been demonstrated in solution such as activation and trapping of orthophosphate^14,44^ and phosphorylation.^70^ However, a complete system has not yet been demonstrated in solution. The demonstration of a prebiotic phosphate transfer system in solution is beset by two challenges: (i) the high activity of water means that it outcompetes other nucleophiles for activated sources of phosphate,^14^ and (ii) the most thermodynamically favored product to form in aqueous solution is orthophosphate, not phosphorylated organic compounds.^41^ Life has overcome these challenges by using enzymes to selectively catalyze phosphorylations. The enzyme active site selectively binds the substrates to be phosphorylated and with the loss of the substrates solvent shell as it enters the active site, the water activity within the active site is lowered.^71−73^ Our work here has been inspired by how life uses enzymes. Our use of wet/dry cycles (commonly used in prebiotic chemistry^59−62^) enables the phosphorylation of organic compounds by lowering water activity. The histidyl peptides catalyze the phosphorylation (the *k*_cat_ in enzyme kinetics). Going forward, a possible next step is to find a catalyst that binds the organic substrate (the *K*_M_ in enzyme kinetics) in an optimal conformation to accept the phosphate from the phosphorylated histidyl intermediate such that phosphorylation of the organic substrate is favored over water. Ultimately, such a catalyst could eliminate the need for wet/dry cycles to lower water activity. Potentially, histidyl peptides with additional amino acid residues to bind substrates could achieve this.

## 4 Conclusions

In conclusion, we have demonstrated here a prebiotic precursor to life’s phosphate transfer system with imidazole phosphate as an ATP analog and histidyl peptide organocatalysts as kinase enzyme analogs. This system is inspired by the chemical strategy that life uses with kinetically stable and thermodynamically activated molecules with enzyme catalysts. Our system demonstrates a new possible organocatalytic role for histidyl peptides at the origins of life. Thus, our work presents an important step forward in the advance of prebiotic chemical systems toward life.

## Supporting Information Available

General procedures; representative ^31^P NMR spectra, concentration vs time plots, reaction schemes, rate equations and fitting of the histidyl-catalyzed hydrolysis of imidazole phosphate; representative ^31^P NMR spectra and yield vs time plots for the histidyl-catalyzed phosphorylation of glycerol by imidazole phosphate; representative ^31^P NMR spectra and yield vs time plots for the physicochemical orthophosphate cycles with histidyl catalysts; *in situ* NMR spectroscopic characterization of phosphorylated histidyl intermediates (PDF)

## Abbreviations

KSTA: kinetically stable and thermodynamically activated
ImP: imidazole phosphate
DPI: diphosphoimidazole
PHis: phosphorylated histidyl intermediate
HMPA: hexamethylphosphoramide
