# Supplementary material for: A Prebiotic Precursor to Life’s Phosphate Transfer System with an ATP Analog and Histidyl Peptide Organocatalysts
Source: J Am Chem Soc. 2024 Mar 6;146(11):7839–49. doi: 10.1021/jacs.4c01156 (PMC10958518; doi:10.1021/jacs.4c01156)
Supplement: Supplementary file 1 — ja4c01156_si_001.pdf [file ja4c01156_si_001.pdf]

Supporting Information

A prebiotic precursor to Life's phosphate transfer system with an ATP analog and histidyl peptide organocatalysts

Oliver R. Maguire*, Iris B. A. Smokers, Bob G. Oosterom, Alla Zheliezniak, Wilhelm T. S. Huck*

Institute for Molecules and Materials, Radboud University Nijmegen, Heyendaalseweg 135, 6525 AJ Nijmegen, The Netherlands.

*Correspondence to: o.maguire@science.ru.nl w.huck@science.ru.nl.

Table of Contents

S1 General Procedures 9

S1.1 Materials 9

S1.2 Instrumentation and Software 9

S1.3 Yield determination using ^{31}P NMR spectroscopy 10

S1.3.1 A note on yield determination using ^{31}P NMR spectra 10

S1.3.2 Measurement of T1 relaxation times for quantitative ^{31}P NMR experiments 10

S2 Histidyl-catalyzed hydrolysis of imidazole phosphate (Main Text Figure 2) 11

S2.1 Preparation of stock solutions 11

S2.2 Experimental method 11

S2.3 Hydrolysis of imidazole phosphate with histidine 12

S2.3.1 Characterisation of phosphorylated histidine intermediate 16

S2.4 Hydrolysis of imidazole phosphate with acetyl histidine 16

S2.4.1 Characterisation of phosphorylated acetyl histidine intermediate 18

S2.5 Hydrolysis of imidazole phosphate with alanine 19

S2.6 Hydrolysis of imidazole phosphate with His-Asp 21

S2.6.1 Characterisation of phosphorylated His-Asp intermediate 23

S2.7 Hydrolysis of imidazole phosphate with His-Lys 24

S2.7.1 Characterisation of phosphorylated His-Lys intermediate 26

S2.8 Hydrolysis of imidazole phosphate with His-Gly-Gly 27

S2.8.1 Characterisation of phosphorylated His-Gly-Gly intermediate 29

S2.9 Hydrolysis of imidazole phosphate with hercynine 30

S2.9.1 Characterisation of phosphorylated hercynine intermediate 32

S2.10 Uncatalysed hydrolysis of imidazole phosphate 32

S2.11 Hydrolysis of imidazole phosphate with histidine with 1.0 M MOPS 34

S2.12 Hydrolysis of imidazole phosphate with histidine with 0.75 M MOPS 36

S2.13 Hydrolysis of imidazole phosphate with histidine with 0.5 M MOPS 38

S2.14 Assessing whether buffer catalysis plays a role in the histidyl catalysed hydrolysis of imidazole phosphate. 41

S2.15 Uncatalysed hydrolysis of imidazole phosphate with 50 mM Imidazole 41

S2.16 Comparison of uncatalysed hydrolysis of imidazole phosphate with 50 mM Imidazole to other hydrolysis reactions 44

S2.17 Unchanged concentration of histidyl catalyst during hydrolysis reaction 45

S2.18 Determination of rate constants for the hydrolysis of imidazole phosphate 45

S3 Histidyl-catalyzed phosphorylation of glycerol by imidazole phosphate (Main Text Figure 3) 50

S3.1 Experimental Method 50

S3.2 Phosphorylation of glycerol by imidazole phosphate with histidine catalyst 51

S3.2.1 Experiment 1 - 3.25 mmol glycerol + 0.13 mmol imidazole phosphate + 0.13 mmol histidine 51
S3.2.2 Experiment 2 - 3.25 mmol glycerol + 0.13 mmol imidazole phosphate + 0.13 mmol histidine 52
S3.2.3 Experiment 3 - 3.25 mmol glycerol + 0.13 mmol imidazole phosphate + 0.13 mmol histidine 53
S3.2.4 Combined results for 3.25 mmol glycerol + 0.13 mmol imidazole phosphate + 0.13 mmol histidine 55
S3.2.5 Comparison between samples measured immediately and samples stored at -20 °C for 1 week for 3.25 mmol glycerol + 0.13 mmol imidazole phosphate + 0.13 mmol histidine 56
S3.3 Phosphorylation of glycerol by imidazole phosphate with His-Asp catalyst 57
S3.3.1 Experiment 1 - 3.25 mmol glycerol + 0.13 mmol imidazole phosphate + 0.13 mmol His-Asp 57
S3.3.2 Experiment 2 - 3.25 mmol glycerol + 0.13 mmol imidazole phosphate + 0.13 mmol His-Asp 58
S3.3.3 Experiment 3 - 3.25 mmol glycerol + 0.13 mmol imidazole phosphate + 0.13 mmol His-Asp 59
S3.3.4 Combined results for 3.25 mmol glycerol + 0.13 mmol imidazole phosphate + 0.13 mmol His-Asp 61
S3.3.5 Comparison between samples measured immediately and samples stored at -20 °C for 1 week for 3.25 mmol glycerol + 0.13 mmol imidazole phosphate + 0.13 mmol His-Asp 62
S3.4 Phosphorylation of glycerol by imidazole phosphate with His-Lys catalyst 63
S3.4.1 Experiment 1 - 3.25 mmol glycerol + 0.13 mmol imidazole phosphate + 0.13 mmol His-Lys 63
S3.4.2 Experiment 2 - 3.25 mmol glycerol + 0.13 mmol imidazole phosphate + 0.13 mmol His-Lys 64
S3.4.3 Experiment 3 - 3.25 mmol glycerol + 0.13 mmol imidazole phosphate + 0.13 mmol His-Lys 65
S3.4.4 Combined results for 3.25 mmol glycerol + 0.13 mmol imidazole phosphate + 0.13 mmol His-Lys 67
S3.4.5 Comparison between samples measured immediately and samples stored at -20 °C for 1 week for 3.25 mmol glycerol + 0.13 mmol imidazole phosphate + 0.13 mmol His-Lys 68
S3.5 Phosphorylation of glycerol by imidazole phosphate with His-Gly-Gly catalyst 69
S3.5.1 Experiment 1 - 3.25 mmol glycerol + 0.13 mmol imidazole phosphate + 0.13 mmol His-Gly-Gly 69
S3.5.2 Experiment 2 - 3.25 mmol glycerol+0.13 mmol imidazole phosphate+0.13 mmol His-Gly-Gly 70
S3.5.3 Experiment 3 - 3.25 mmol glycerol + 0.13 mmol imidazole phosphate + 0.13 mmol His-Gly-Gly 72
S3.5.4 Combined results for 3.25 mmol glycerol + 0.13 mmol imidazole phosphate + 0.13 mmol His-Gly-Gly 73
S3.6 Phosphorylation of glycerol by imidazole phosphate with His-His catalyst (0.5 equivalent) 74
S3.6.1 Experiment 1 - 3.25 mmol glycerol + 0.13 mmol imidazole phosphate + 0.07 mmol His-His 74
S3.6.2 Experiment 2 - 3.25 mmol glycerol + 0.13 mmol imidazole phosphate + 0.07 mmol His-His 76
S3.6.3 Combined results for 3.25 mmol glycerol + 0.13 mmol imidazole phosphate + 0.07 mmol His-His 77
S3.6.4 Characterisation of phosphorylated His-His intermediate 77
S3.7 Phosphorylation of glycerol by imidazole phosphate with His-His catalyst (1 equivalent) 79
S3.7.1 Experiment 1 - 3.25 mmol glycerol + 0.13 mmol imidazole phosphate + 0.13 mmol His-His 79
S3.7.2 Experiment 2 - 3.25 mmol glycerol + 0.13 mmol imidazole phosphate + 0.13 mmol His-His 80
S3.7.3 Experiment 3 - 3.25 mmol glycerol + 0.13 mmol imidazole phosphate + 0.13 mmol His-His 81
S3.7.4 Combined results for 3.25 mmol glycerol + 0.13 mmol imidazole phosphate + 0.13 mmol His-His 82
S3.8 Uncatalysed phosphorylation of glycerol by imidazole phosphate 83
S3.8.1 Experiment 1 - 3.25 mmol glycerol + 0.13 mmol imidazole phosphate 83
S3.8.2 Experiment 2 - 3.25 mmol glycerol + 0.13 mmol imidazole phosphate 84
S3.8.3 Experiment 3 - 3.25 mmol glycerol + 0.13 mmol imidazole phosphate 85
S3.8.4 Combined results for 3.25 mmol glycerol + 0.13 mmol imidazole phosphate 86

S3.8.5 Comparison between samples measured immediately and samples stored at -20 °C for 1 week for 3.25 mmol glycerol + 0.13 mmol imidazole phosphate 87
S3.9 Phosphorylation of glycerol by imidazole phosphate with His-Gly catalyst 87
S3.9.1 Experiment 1 - 3.25 mmol glycerol + 0.13 mmol imidazole phosphate + 0.13 mmol His-Gly 88
S3.9.2 Experiment 2 - 3.25 mmol glycerol + 0.13 mmol imidazole phosphate + 0.13 mmol His-Gly 89
S3.9.3 Experiment 3 - 3.25 mmol glycerol + 0.13 mmol imidazole phosphate + 0.13 mmol His-Gly 90
S3.9.4 Combined results for 3.25 mmol glycerol + 0.13 mmol imidazole phosphate + 0.13 mmol His-Gly 91
S3.9.5 Characterisation of phosphorylated His-Gly intermediate 91
S3.10 Phosphorylation of glycerol by imidazole phosphate with c(His-Gly) catalyst 92
S3.10.1 Experiment 1 - 3.25 mmol glycerol + 0.13 mmol imidazole phosphate + 0.13 mmol c(His-Gly) 92
S3.10.2 Experiment 2 - 3.25 mmol glycerol + 0.13 mmol imidazole phosphate + 0.13 mmol c(His-Gly) 94
S3.10.3 Experiment 3 - 3.25 mmol glycerol + 0.13 mmol imidazole phosphate + 0.13 mmol c(His-Gly) 95
S3.10.4 Combined results for 3.25 mmol glycerol + 0.13 mmol imidazole phosphate + 0.13 mmol c(His-Gly)96
S3.10.5 Characterisation of phosphorylated c(His-Gly) intermediate 96
S3.11 Phosphorylation of glycerol by imidazole phosphate with Ala-His-Lys catalyst 97
S3.11.1 Experiment 1 - 3.25 mmol glycerol + 0.13 mmol imidazole phosphate + 0.13 mmol Ala-His-Lys 97
S3.11.2 Experiment 2 - 3.25 mmol glycerol + 0.13 mmol imidazole phosphate + 0.13 mmol Ala-His-Lys 99
S3.11.3 Experiment 3 - 3.25 mmol glycerol + 0.13 mmol imidazole phosphate + 0.13 mmol Ala-His-Lys 100
S3.11.4 Combined results for 3.25 mmol glycerol + 0.13 mmol imidazole phosphate + 0.13 mmol Ala-His-Lys 101
S3.11.5 Characterisation of phosphorylated Ala-His-Lys intermediate 101
S3.12 Phosphorylation of glycerol by imidazole phosphate with Arg-His-NH_2 catalyst 102
S3.12.1 Experiment 1 - 3.25 mmol glycerol + 0.13 mmol imidazole phosphate + 0.13 mmol Arg-His-NH_2 103
S3.12.2 Experiment 2 - 3.25 mmol glycerol + 0.13 mmol imidazole phosphate + 0.13 mmol Arg-His-NH_2 104
S3.12.3 Experiment 3 - 3.25 mmol glycerol + 0.13 mmol imidazole phosphate + 0.13 mmol Arg-His-NH_2 105
S3.12.4 Combined results for 3.25 mmol glycerol + 0.13 mmol imidazole phosphate + 0.13 mmol Arg-His-NH_2 106
S3.12.5 Characterisation of phosphorylated Arg-His-NH_2 intermediate 106
S3.13 Phosphorylation of glycerol by imidazole phosphate with Ala-His catalyst 107
S3.13.1 Experiment 1 - 3.25 mmol glycerol + 0.13 mmol imidazole phosphate + 0.13 mmol Ala-His 107
S3.13.2 Experiment 2 - 3.25 mmol glycerol + 0.13 mmol imidazole phosphate + 0.13 mmol Ala-His 109
S3.13.3 Experiment 3 - 3.25 mmol glycerol + 0.13 mmol imidazole phosphate + 0.13 mmol Ala-His 110
S3.13.4 Combined results for 3.25 mmol glycerol + 0.13 mmol imidazole phosphate + 0.13 mmol Ala-His 111
S3.13.5 Characterisation of phosphorylated Ala-His intermediate 111
S3.14 Phosphorylation of glycerol by imidazole phosphate with Ser-His catalyst 112
S3.14.1 Experiment 1 - 3.25 mmol glycerol + 0.13 mmol imidazole phosphate + 0.13 mmol Ser-His 112
S3.14.2 Experiment 2 - 3.25 mmol glycerol + 0.13 mmol imidazole phosphate + 0.13 mmol Ser-His 114
S3.14.3 Experiment 3 - 3.25 mmol glycerol + 0.13 mmol imidazole phosphate + 0.13 mmol Ser-His 115
S3.14.4 Combined results for 3.25 mmol glycerol + 0.13 mmol imidazole phosphate + 0.13 mmol Ser-His 116
S3.14.5 Characterisation of phosphorylated Ser-His intermediate 116

S3.15 Phosphorylation of glycerol by imidazole phosphate with Gly-Lys-His catalyst 117

S3.15.1 Experiment 1 - 3.25 mmol glycerol + 0.13 mmol imidazole phosphate + 0.13 mmol Gly-Lys-His 117

S3.15.2 Experiment 2 - 3.25 mmol glycerol + 0.13 mmol imidazole phosphate + 0.13 mmol Gly-Lys-His 119

S3.15.3 Experiment 3 - 3.25 mmol glycerol + 0.13 mmol imidazole phosphate + 0.13 mmol Gly-Lys-His 120

S3.15.4 Combined results for 3.25 mmol glycerol + 0.13 mmol imidazole phosphate + 0.13 mmol Gly-Lys-His 121

S3.15.5 Characterisation of phosphorylated Gly-Lys-His intermediate 121

S3.16 Phosphorylation of glycerol by imidazole phosphate with Gly-Gly-His catalyst 122

S3.16.1 Experiment 1 - 3.25 mmol glycerol + 0.13 mmol imidazole phosphate + 0.13 mmol Gly-Gly-His 123

S3.16.2 Experiment 2 - 3.25 mmol glycerol + 0.13 mmol imidazole phosphate + 0.13 mmol Gly-Gly-His 124

S3.16.3 Experiment 3 - 3.25 mmol glycerol + 0.13 mmol imidazole phosphate + 0.13 mmol Gly-Gly-His 125

S3.16.4 Combined results for 3.25 mmol glycerol + 0.13 mmol imidazole phosphate + 0.13 mmol Gly-Gly-His 126

S3.16.5 Characterisation of phosphorylated Gly-Gly-His intermediate 126

S3.17 Comparison of histidyl catalysts for phosphate transfer reactions 127

S3.17.1 Comparison of phosphorylation of glycerol by histidyl catalysts. 127

S3.17.2 Comparison of orthophosphate production by histidyl catalysts 128

S3.17.3 Comparison of pyrophosphate production by histidyl catalysts 128

S3.18 The quality of repeatability of the phosphate transfer reactions in the paste reactions 129

S3.19 Comparison of histidyl catalysts for phosphate transfer reactions with 10-fold excess of Glycerol 129

S3.19.1 Experimental Method 129

S3.19.2 Phosphorylation of glycerol by imidazole phosphate with 1.00 eq. histidine catalyst 130

S3.19.3 Phosphorylation of glycerol by imidazole phosphate with 0.75 eq. histidine catalyst 132

S3.19.4 Phosphorylation of glycerol by imidazole phosphate with 0.50 eq. histidine catalyst 133

S3.19.5 Phosphorylation of glycerol by imidazole phosphate with 0.25 eq. histidine catalyst 135

S3.19.6 Uncatalysed phosphorylation of glycerol by imidazole phosphate 136

S3.19.7 Comparison of different equivalents of histidine catalyst upon the phosphorylation of glycerol by imidazole phosphate. 139

S3.20 Comparison of histidyl catalysts for phosphate transfer reactions with 5-fold excess of Glycerol 139

S3.20.1 Experimental Method 139

S3.20.2 Phosphorylation of glycerol by imidazole phosphate with 1.0 eq. histidine catalyst and 5.0 eq. of glycerol 140

S3.20.3 Uncatalysed phosphorylation of glycerol by imidazole phosphate with 5 eq. of glycerol 141

S4 Physicochemical orthophosphate cycles with histidyl catalysts (Main Text Figure 4) 143

S4.1 Experimental Method 143

S4.2 Wet/Dry Cycle for the phosphorylation of glycerol by imidazole phosphate with a histidine catalyst 144

S4.2.1 Wet/Dry cycle for the phosphorylation of glycerol catalysed by 100 mM histidine catalyst 144

S4.2.2 Wet/Dry cycle for the phosphorylation of glycerol catalysed by 100 mM histidine catalyst 1st repeat145

S4.2.3 Wet/Dry cycle for the phosphorylation of glycerol catalysed by 100 mM histidine catalyst 2nd repeat 146

S4.3 Wet/Dry Cycle for the phosphorylation of glycerol by imidazole phosphate with 50 mM histidine catalyst 148

S4.3.1 Wet/Dry cycle for the phosphorylation of glycerol catalysed by 50 mM histidine catalyst 148

S4.3.2 Wet/Dry cycle for the phosphorylation of glycerol catalysed by 50 mM histidine catalyst 1st repeat149

S4.3.3 Wet/Dry cycle for the phosphorylation of glycerol catalysed by 50 mM histidine catalyst 2nd repeat150

S4.3.4 Wet/Dry cycle for the phosphorylation of glycerol catalysed by 50 mM histidine catalyst 3rd repeat151

S4.4 Wet/Dry Cycle for the phosphorylation of glycerol by imidazole phosphate with 100 mM His-NH_2 catalyst 153

S4.4.1 Wet/Dry cycle for the phosphorylation of glycerol catalysed by 100 mM His-NH_2 catalyst 153

S4.4.2 Wet/Dry cycle for the phosphorylation of glycerol catalysed by 100 mM His-NH_2 catalyst 1st repeat154

S4.4.3 Wet/Dry cycle for the phosphorylation of glycerol catalysed by 100 mM His-NH_2 catalyst 2nd repeat155

S4.5 Wet/Dry Cycle for the phosphorylation of glycerol by imidazole phosphate with 50 mM His-NH_2 catalyst157

S4.5.1 Wet/Dry cycle for the phosphorylation of glycerol catalysed by 50 mM His-NH_2 catalyst 157

S4.5.2 Wet/Dry cycle for the phosphorylation of glycerol catalysed by 50 mM His-NH_2 catalyst 1st repeat158

S4.5.3 Wet/Dry cycle for the phosphorylation of glycerol catalysed by 50 mM His-NH_2 catalyst 2nd repeat159

S4.6 Wet/Dry Cycle for the phosphorylation of glycerol by imidazole phosphate with a His-Asp catalyst 161

S4.6.1 Wet/Dry cycle for the phosphorylation of glycerol catalysed by 100 mM His-Asp catalyst 161

S4.6.2 Wet/Dry cycle for the phosphorylation of glycerol catalysed by 100 mM His-Asp catalyst 1st repeat162

S4.6.3 Wet/Dry cycle for the phosphorylation of glycerol catalysed by 100 mM His-Asp catalyst 2nd repeat163

S4.7 Wet/Dry Cycle for the phosphorylation of glycerol by imidazole phosphate with a His-Lys catalyst 165

S4.7.1 Wet/Dry cycle for the phosphorylation of glycerol catalysed by 100 mM His-Lys catalyst 165

S4.7.2 Wet/Dry cycle for the phosphorylation of glycerol catalysed by 100 mM His-Lys catalyst 1st repeat166

S4.7.3 Wet/Dry cycle for the phosphorylation of glycerol catalysed by 100 mM His-Lys catalyst 2nd repeat167

S4.8 Wet/Dry Cycle for the phosphorylation of glycerol by imidazole phosphate with a His-Gly-Gly catalyst169

S4.8.1 Wet/Dry cycle for the phosphorylation of glycerol catalysed by 100 mM His-Gly-Gly catalyst 169

S4.8.2 Wet/Dry cycle for the phosphorylation of glycerol catalysed by 100 mM His-Gly-Gly catalyst 1st repeat 170

S4.9 Wet/Dry Cycle with phosphorylation of glycerol by imidazole phosphate – uncatalysed reaction 172

S4.9.1 Wet/Dry cycle for the phosphorylation of glycerol catalysed uncatalysed reaction 172

S4.9.2 Wet/Dry cycle for the phosphorylation of glycerol catalysed uncatalysed reaction 1st repeat 173

S4.9.3 Wet/Dry cycle for the phosphorylation of glycerol catalysed uncatalysed reaction 2nd repeat 174

S4.10 Wet/Dry Cycle for the phosphorylation of glycerol with 100 mM histidine catalyst and no imidazole present 176

S4.10.1 Wet/Dry cycle for the phosphorylation of glycerol catalysed by 100 mM histidine catalyst and no imidazole present 176

S4.10.2 Wet/Dry cycle for the phosphorylation of glycerol catalysed by 100 mM histidine catalyst and no imidazole present 177

S4.10.3 Wet/Dry cycle for the phosphorylation of glycerol catalysed by 100 mM histidine catalyst and no imidazole present 178
S4.11 Experimental Method with glycerate 180
S4.12 Wet/Dry Cycle for the phosphorylation of glycerate by imidazole phosphate with a histidine catalyst 180
S4.12.1 Wet/Dry cycle for the phosphorylation of glycerate catalysed by 100 mM histidine catalyst 181
S4.12.2 Wet/Dry cycle for the phosphorylation of glycerate catalysed by 100 mM histidine catalyst 1st repeat 182
S4.13 Wet/Dry Cycle for the phosphorylation of glycerate by imidazole phosphate with a His-Lys catalyst 183
S4.13.1 Wet/Dry cycle for the phosphorylation of glycerate catalysed by 100 mM His-Lys catalyst 183
S4.13.2 Wet/Dry cycle for the phosphorylation of glycerate catalysed by 100 mM His-Lys catalyst 1st repeat184
S4.14 Wet/Dry Cycle with phosphorylation of glycerate by imidazole phosphate – uncatalysed reaction 186
S4.14.1 Wet/Dry cycle for the phosphorylation of glycerate uncatalysed reaction 186
S4.14.2 Wet/Dry cycle for the phosphorylation of glycerol uncatalysed reaction 1st repeat 187
S4.15 Wet/Dry Cycle for the phosphorylation of glycerol by imidazole phosphate with 50 mM histidine catalyst at 4 °C 188
S4.16 Wet/Dry Cycle for the phosphorylation of glycerol by imidazole phosphate at 4 °C 190
S4.17 Wet/Dry Cycle for the phosphorylation of glycerol by imidazole phosphate with 50 mM histidine catalyst at 35 °C 191
S4.18 Wet/Dry Cycle for the phosphorylation of glycerol by imidazole phosphate at 35 °C 193
S4.19 Wet/Dry Cycle for the phosphorylation of glycerol by imidazole phosphate with 50 mM histidine catalyst at 50 °C 194
S4.20 Wet/Dry Cycle for the phosphorylation of glycerol by imidazole phosphate at 50 °C 196
S4.21 Wet/Dry Cycle for the phosphorylation of glycerol by imidazole phosphate with 50 mM histidine catalyst at pH 6.5 197
S4.22 Wet/Dry Cycle for the phosphorylation of glycerol by imidazole phosphate at pH 6.5 199
S4.23 Wet/Dry Cycle for the phosphorylation of glycerol by imidazole phosphate with 50 mM histidine catalyst at pH 8.0 200
S4.24 Wet/Dry Cycle for the phosphorylation of glycerol by imidazole phosphate at pH 8.0 202
S4.25 Wet/Dry Cycle for the phosphorylation of glycerol by imidazole phosphate with 1 % weight/volume Montmorillonite 203
S4.26 Wet/Dry Cycle for the phosphorylation of glycerol by imidazole phosphate with 1 % weight/volume Montmorillonite 205
S4.27 Wet/Dry Cycle for the phosphorylation of glycerol by imidazole phosphate with 1 % weight/weight Montmorillonite 206
S4.28 Wet/Dry Cycle for the phosphorylation of glycerol by imidazole phosphate with 1 % weight/weight Montmorillonite 208
S4.29 Wet/Dry Cycle for the phosphorylation of glycerol by imidazole phosphate with 1 % weight/volume Hydroxyapatite 210

S4.30 Wet/Dry Cycle for the phosphorylation of glycerol by imidazole phosphate with 1 % weight/volume Hydroxyapatite 211

S4.31 Wet/Dry Cycle for the phosphorylation of glycerol by imidazole phosphate with 1 % weight/weight Hydroxyapatite 213

S4.32 Wet/Dry Cycle for the phosphorylation of glycerol by imidazole phosphate with 1 % weight/weight Hydroxyapatite 214

S5 *In situ* NMR Spectroscopic Characterisation of Phosphorylated Histidyls 217

S5.1 Phosphorylated Histidine Intermediate 217

S5.2 Phosphorylated Acetyl-Histidine Intermediate 221

S5.3 Phosphorylated His-Asp Intermediate 224

S5.4 Phosphorylated His-Lys Intermediate 227

S5.5 Phosphorylated His-Gly-Gly Intermediate 230

S5.6 Phosphorylated Hercynine Intermediate 233

S5.7 Phosphorylated His-Gly Intermediate 236

S5.8 Phosphorylated c(His-Gly) Intermediate 239

S5.9 Phosphorylated Ala-His Intermediate 242

S5.10 Phosphorylated Ser-His Intermediate 245

S5.11 Phosphorylated Arg-His-NH_2 Intermediate 248

S5.12 Phosphorylated His-His Intermediate 251

S5.13 Phosphorylated Gly-Gly-His Intermediate 254

S5.14 Phosphorylated Gly-Lys-His Intermediate 257

S6 Supporting References 260

S1 General Procedures

S1.1 Materials

All chemicals and reagents were used as received from commercial suppliers. We used MilliQ (MQ) water (i.e., ultrapure deionized water) from Millipore Corporation.

The following compounds were purchased from Sigma Aldrich/Merck: deuterium oxide, imidazole, zinc chloride, magnesium chloride, hexamethylphosphoramide, phosphor(V)oxychloride, L-histidine monochloride monohydrate, glycerol, rac-glycerol 1-phosphate sodium salt hydrate, glycerol-2-phosphate sodium salt, Gly-Gly-His, His-Gly-Gly, Montmorillonite.

The following compounds were purchased from Fisher Scientific: sodium phosphate monobasic anhydrous, D-glucose, volumetric 1.0 M hydrochloric acid, volumetric 2.0 M hydrochloric acid, volumetric 1.0 M sodium hydroxide, volumetric 2.0 M sodium hydroxide, Thermo Scientific Orion Standard All-in-One pH buffer Kit, hydroxyapatite.

The following compounds were purchased from Bachem: His-Gly, His-Asp, His-Lys HBr salt, His-Gly-Gly, Ala-His-Lys acetate salt, c(His-Gly), His-His TFA salt, Ala-His, Arg-His-NH_2 acetate salt, Ser-His, Gly-Lys-His acetate salt.

The following compounds were purchased from Bioconnect: hercynine.

The following compounds were purchased from Merck: potassium cyanate.

The following compounds were purchased from Fluorochem: citric acid.

Calcium imidazole phosphate was synthesised according to the same procedure as detailed in our previous publication.[1]

S1.2 Instrumentation and Software

Nuclear Magnetic Resonance (NMR) spectra for ^{1}H, ^{31}P and ^{13}C nuclei were measured on a *Bruker-AVANCE III 500* spectrometer at 500 MHz or on a *Bruker-AVANCE III 400* spectrometer at 400 MHz. The chemical shifts for ^{1}H and given in parts per million (ppm) and calibrated using a residual solvent peak of 4.79 for D_2O in ^{1}H NMR. ^{31}P Multiplets are reported as s (singlet), d (doublet), dd (doublet of doublet), t (triplet), q (quartet) and m (multiplet). Coupling constants, *J*, are reported in Hertz (Hz). The number of protons (n) for a given resonance is indicated as nH and is based on the spectral integration values.

Quantitative ^{31}P NMR spectroscopy was performed for all ^{31}P NMR spectra. A pulse sequence was set up with 8 transients (nt = 8), a P1 = 13 ms which corresponds to approximately a 90° pulse angle and a d_1 relaxation delay of 30 s, in order to ensure full relaxation of nuclei between each transient. A solvent suppression sequence was used for the measurement of ^{1}H NMR spectra when a 9 : 1 H_2O : D_2O solvent was used.

The pH of solutions was determined using a Mettler Toledo Five Easy FE20 pH meter and a Hamilton SpinTrode pH probe. The pH meter calibrated with Thermo Scientific Orion Standard buffers at pH 4.01, pH 7.00, pH 10.00.

All data was processed in MestReNova 14, OriginPro 2018b and the Spyder Python Environment.

S1.3 Yield determination using ^{31}P NMR spectroscopy

S1.3.1 A note on yield determination using ^{31}P NMR spectra

The yields reported in the paper and in the SI were determined by integrating all the peaks in the ^{31}P NMR spectra, summating all of these integrals and calculating the yield based upon this total summated integral.

S1.3.2 Measurement of T1 relaxation times for quantitative ^{31}P NMR experiments

We took from our previous study[1] the measurements of the 90° pulse angle and T1 relaxation times for the ^{31}P nuclei in the key phosphate containing species in this study. For clarity this data is included below (Supporting Table 1). The longest T1 relaxation time measured was 3.2 s for orthophosphate. To be quantitative an NMR experiment with a 90° pulse requires a delay of 5*T1. This delay ensures full relaxation of the nuclei back into orientation of the applied magnetic field. For our ^{31}P-NMR experiments with orthophosphate T1 = 3.2 s, this means that a minimum delay of 16.0 s is required in order for quantitative ^{31}P-NMR experiments. We used a d1 delay of 30.0 s and along with a 1.5 – 2.0 s acquisition time, this means that a total of 31.5 – 32.0 s was given for the nuclei to relax. Thus this is approximately double the minimum delay required and therefore our ^{31}P-NMR experiments are quantitative.

Supporting Table 1: Measured 90° pulse lengths (P90) and T1 relaxation times for important phosphate containing compounds used in our study. The P90 and T1 were determined in a 100 mM solution of the compound in 0.5 M citric acid buffer pH 6.85 at 22.0 °C. This citric acid buffer was typically used in our study to analyse the pastes.

Compound	P90 (µs)	T1 (s)
Orthophosphate	13.300	3.20
Carbamoyl phosphate	13.375	2.15
Imidazole phosphate (Calcium salt)	13.350	1.40
Glycerol-1-phosphate	13.688	2.23
Glycerol-2-phosphate	13.750	2.20

S2 Histidyl-catalyzed hydrolysis of imidazole phosphate (Main Text Figure 2)

S2.1 Preparation of stock solutions

A 0.5 M MOPS buffer contianing 0.1 M citric acid was prepared by dissolving 1.57 g (7.50 mmol) MOPS, 288 mg (1.50 mmol) of citric acid and in 15 mL of 9 : 1 H_2O : D_2O and 5.0 M potassium hydroxide was used to adjust the pH of the buffer to 7.50. An internal standard of 50 mM HMPA was added to the solution via the addition of 750 µL of 1.0 M HMPA solution (Note that this volume of HMPA stock solution contributed to the aforementioned total volume 15 mL of the 0.5 M and 0.1 M citric acid solution).

The role of the citric acid in the buffer is to chelate calcium ions and therefore solubilise all calcium phosphate salts. Further details on citric acid dissolution are given in the Supporting Information of O. R. Maguire, I. B. A. Smokers, W. T. S. Huck *Nat. Commun.* **12,** 5517 (2021).

S2.2 Experimental method

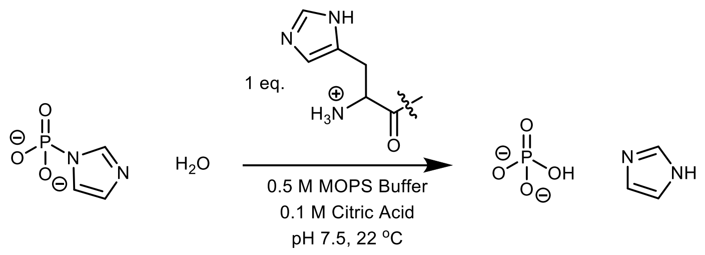

A 50 mM calcium imidazole phosphate and 50 mM histidine solution was prepared by dissolving 4.7 mg (0.025 mmol) calcium imidazole phosphate and 3.9 mg (0.025 mmol, 1 eq) histidine in 0.5 mL 0.5 M MOPS buffer at pH 7.5 in 9 : 1 H_2O : D_2O containing 0.1 M citric acid and 50 mM HMPA internal standard. At the start of the reaction, the pH of the solution was corrected to pH 7.5 if it had changed using 5.0 M HCl and 5.0 M KOH. The reaction was followed by ^{31}P-NMR and ^{1}H-NMR spectroscopy, measuring spectra at a series of time points over the course of 2 days. The pH of the sample was measured at time points over the course of the reaction.

S2.3 Hydrolysis of imidazole phosphate with histidine

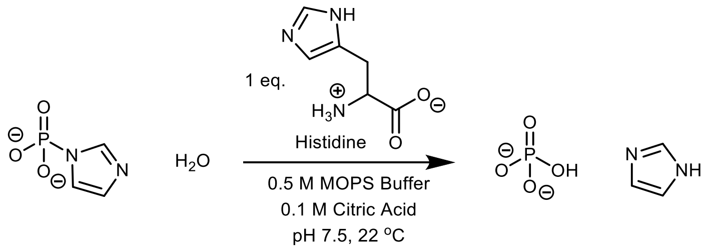

The experiment was performed as detailed in Section S2.2.

Supporting Figure 1 depicts representative ^{31}P NMR spectra for the reaction. The changes in concentration over time for all phosphate containing species are shown in Supporting Figure 2. Supporting Table 2 show the mean concentration and standard deviation of imidazole phosphate and orthophosphate from the triplicate experiments and were used to plot Main Text Figure 2d.

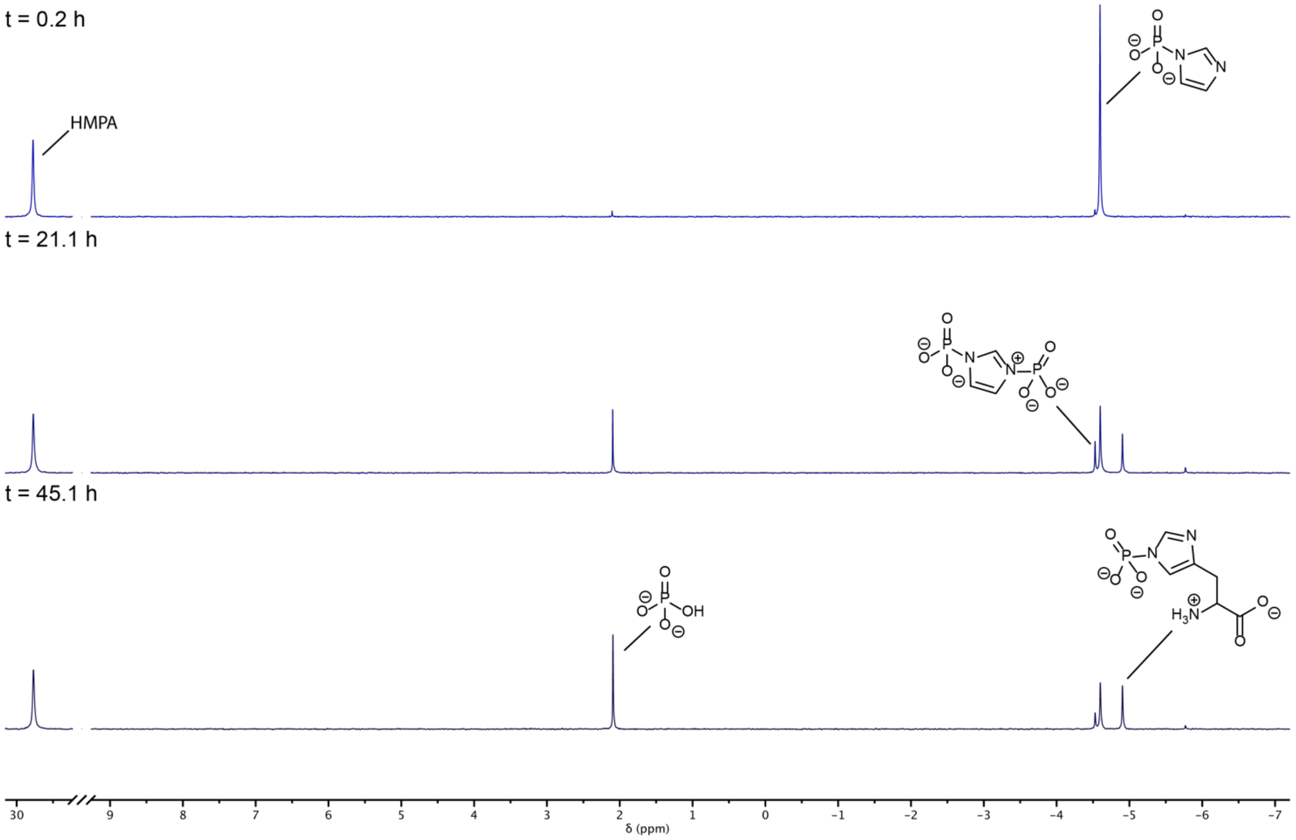

Supporting Figure 1: Representative ^{31}P-NMR spectra over time for the reaction of 50 mM calcium imidazole phosphate and 50 mM histidine solution in 0.5 M MOPS buffer at pH 7.5 in 9 : 1 H_2O : D_2O containing 0.1 M citric acid and 50 mM HMPA internal standard.

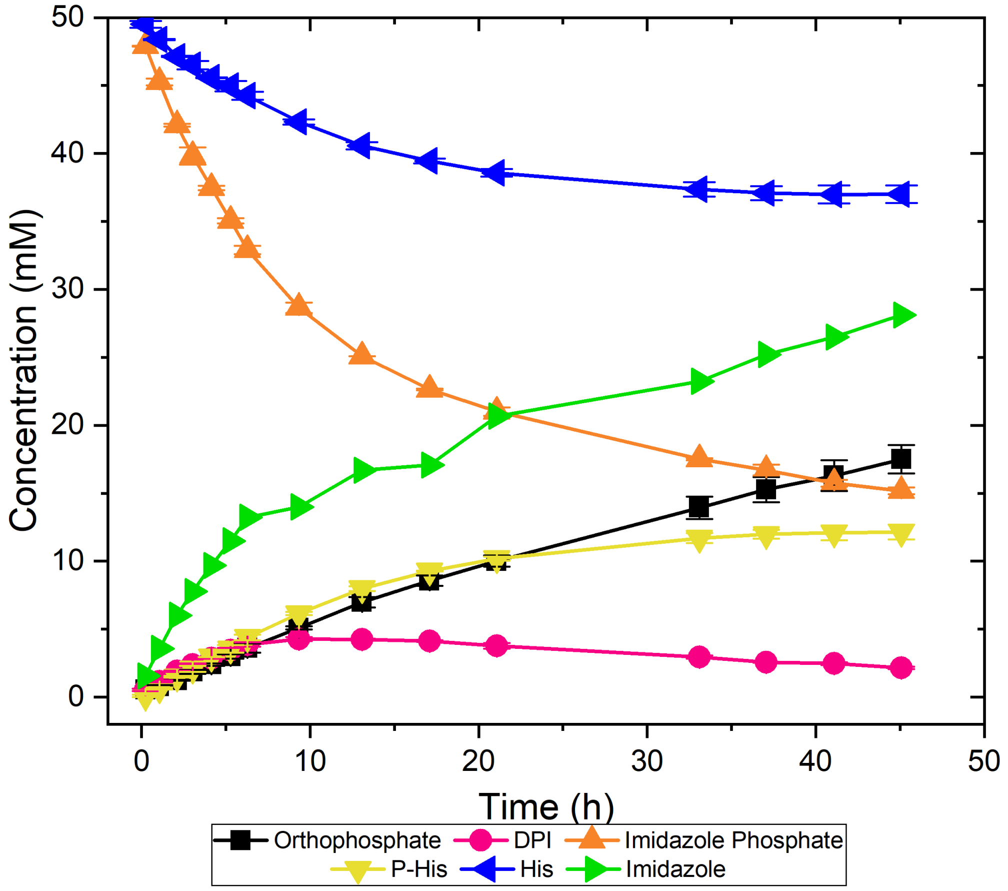

Supporting Figure 2: Changes in concentration over time for the reaction of 50 mM calcium imidazole phosphate and 50 mM histidine solution in 0.5 M MOPS buffer at pH 7.5 in 9 : 1 H_2O : D_2O containing 0.1 M citric acid and 50 mM HMPA internal standard. DPI = Diphosphoimidazole. These data are the mean values and standard deviation based upon duplicate experiments.

Supporting Table 2: Average changes in concentration over time from triplicate experiments for imidazole phosphate and orthophosphate in the ^{31}P-NMR spectra for 50 mM calcium imidazole phosphate and 50 mM histidine solution in 0.5 M MOPS buffer at pH 7.5 in 9 : 1 H_2O : D_2O containing 0.1 M citric acid and 50 mM HMPA internal standard. Standard deviation provided for each value was based on two repeats. These data are used to plot Main Text Figure 2d.

Time (h)	Imidazole Phosphate (mM)			Orthophosphate (mM)		
0.24	48.42	±	0.06	0.00	±	0.00
1.06	45.77	±	0.16	0.24	±	0.01
2.11	42.54	±	0.02	0.69	±	0.05
3.03	40.26	±	0.56	1.33	±	0.07
4.15	37.80	±	0.19	1.85	±	0.01
5.29	35.43	±	0.12	2.48	±	0.07
6.28	33.26	±	0.26	3.09	±	0.26
7.23	31.62	±	0.00	3.16	±	0.00
8.57	28.22	±	0.00	3.45	±	0.00
9.33	28.98	±	0.44	4.58	±	0.04
13.08	25.36	±	0.06	6.51	±	0.31
17.08	22.90	±	0.02	8.09	±	0.30
21.08	21.25	±	0.34	9.56	±	0.33
25.07	20.15	±	0.00	10.41	±	0.00
29.08	18.78	±	0.00	11.63	±	0.00
33.09	17.71	±	0.04	13.53	±	0.76
37.07	16.89	±	0.37	14.88	±	0.87
41.08	15.92	±	0.23	15.91	±	1.08
45.08	15.35	±	0.22	17.14	±	1.01

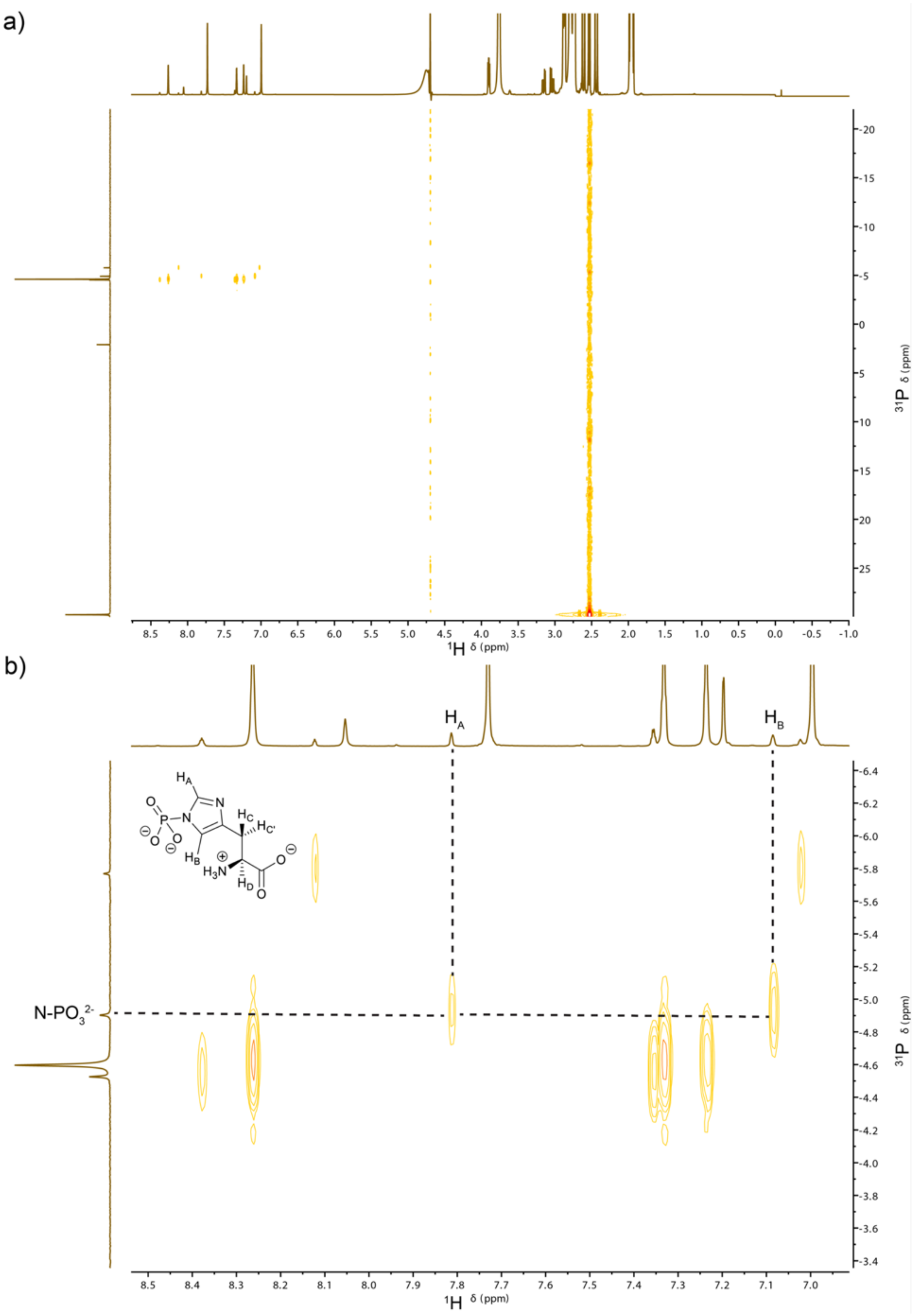

Supporting Figure 3: a) Full ^{1}H ^{31}P HMBC spectrum for the reaction of 50 mM calcium imidazole phosphate and 50 mM histidine solution in 0.5 M MOPS buffer at pH 7.5 in 9 : 1 H_2O : D_2O containing 0.1 M citric acid and 50 mM HMPA internal standard. b) Zoomed in ^{1}H ^{31}P HMBC spectrum on imidazolyl region.

S2.3.1 Characterisation of phosphorylated histidine intermediate

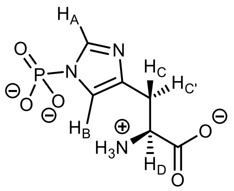

The phosphorylated histidine intermediate was characterised *in situ*. A solution of 50 mM histidine and 50 mM calcium imidazole phosphate in 0.5 mL 0.5 M MOPS buffer at pH 7.5 in 9 : 1 H_2O : D_2O containing 0.1 M citric acid and 50 mM HMPA internal standard was prepared according to the procedure in Section S2.2.

^{31}P NMR (202.46 MHz, 0.5 M MOPS + 0.1 M Citric Acid in 9 : 1 H_2O : D_2O at pH 7.5 and 22 °C): *phosphorylated histidine intermediate* δ (ppm) = - 4.90 (s, 1P). ^{1}H NMR (500.13 MHz, 0.5 M MOPS + 0.1 M Citric Acid in 9 : 1 H_2O : D_2O at pH 7.5 and 22 °C): *phosphorylated histidine intermediate* δ (ppm) = 7.82 (s, 1H, H_A), 7.09 (s, 1H, H_B), 3.90 (dd, 1H, H_D), 3.16 (dd, 1H, H_C), 3.02 (dd, 1H, $H_{C'}$). ^{13}C NMR (125.77 MHz, 0.5 M MOPS + 0.1 M Citric Acid in 9 : 1 H_2O : D_2O at pH 7.5 and 22 °C): *phosphorylated histidine intermediate* δ (ppm) = 174.1 (s, 1C, COOH), 138.8 (s, 1C, imid C-H_A), 133.7 (s, 1C, imid), 118.8 (s, 1C, imid C-H_B), 54.8 (s, 1C, C-H_D), 27.7 (s, 1C, C-$H_{C+C'}$).

S2.4 Hydrolysis of imidazole phosphate with acetyl histidine

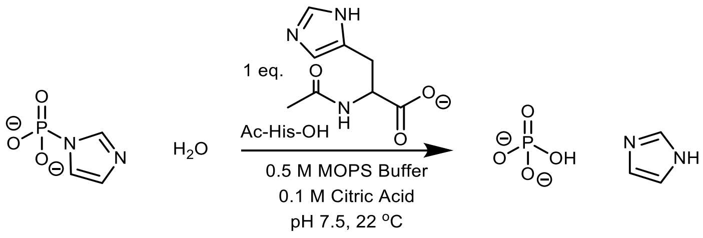

An identical procedure was used as detailed in Section S2.2 but with 50 mM acetyl histidine instead of histidine and prepared from 5.4 mg (0.025 mmol, 1 eq) of acetyl histidine.

Supporting Figure 4 depicts representative ^{31}P NMR spectra for the reaction. The changes in concentration over time for all phosphate containing species are shown in Supporting Figure 5. Supporting Table 3 show the mean concentration and standard deviation of imidazole phosphate and orthophosphate from the triplicate experiments and were used to plot Main Text Figure 2d.

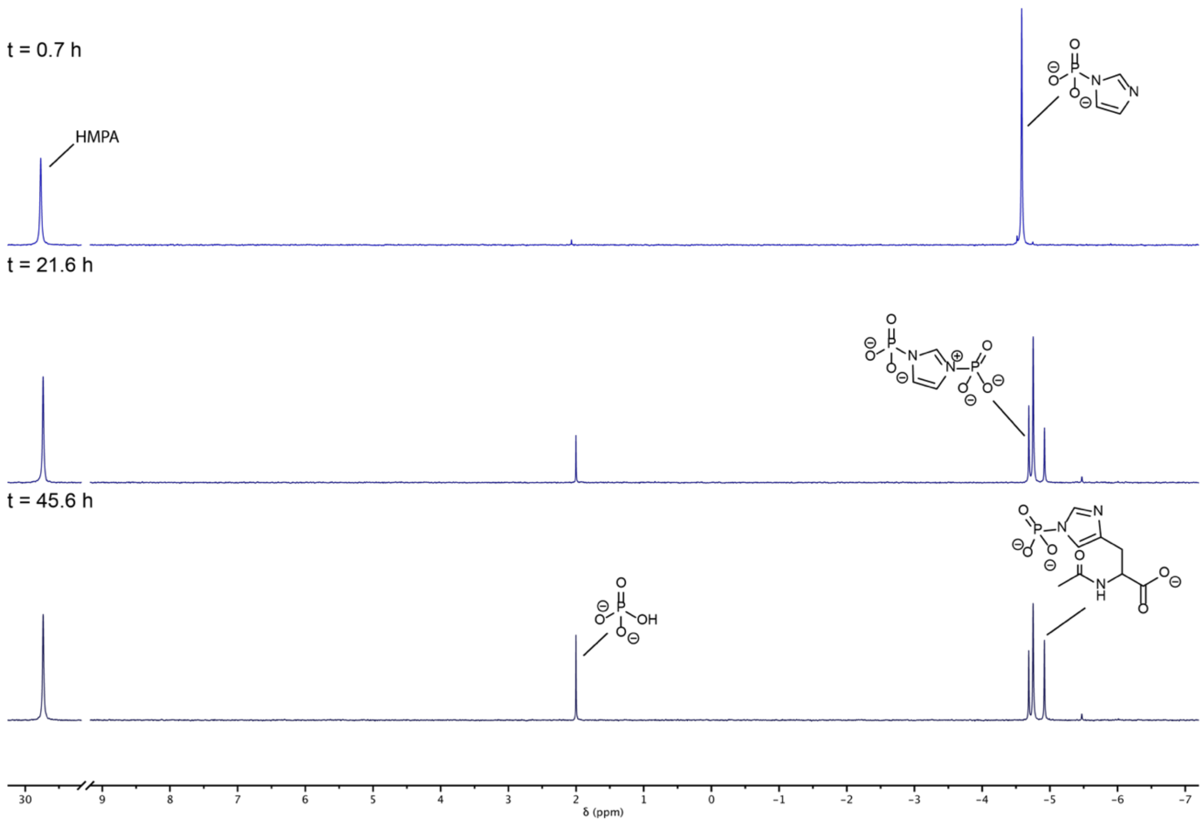

Supporting Figure 4: Representative ^{31}P-NMR spectra over time for the reaction of 50 mM calcium imidazole phosphate and 50 mM acetyl histidine solution in 0.5 M MOPS buffer at pH 7.5 in 9 : 1 H_2O : D_2O containing 0.1 M citric acid and 50 mM HMPA internal standard.

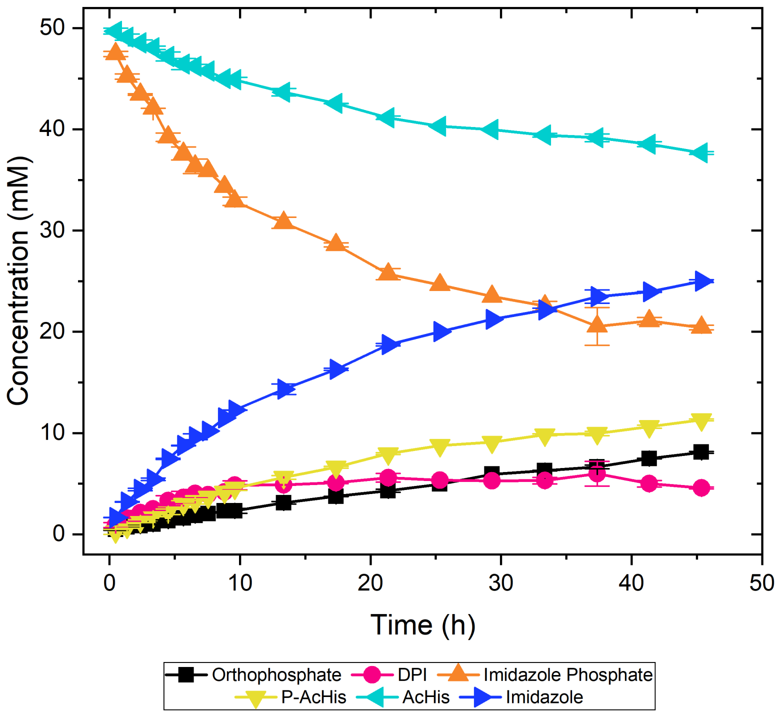

Supporting Figure 5: Changes in concentration over time for the reaction of 50 mM calcium imidazole phosphate and 50 mM acetyl histidine solution in 0.5 M MOPS buffer at pH 7.5 in 9 : 1 H_2O : D_2O containing 0.1 M citric acid and 50 mM HMPA internal standard. DPI = Diphosphoimidazole. These data are the mean values and standard deviation based upon duplicate experiments.

Supporting Table 3: Average changes in concentration over time from triplicate experiments for imidazole phosphate and orthophosphate in the ^{31}P-NMR spectra for 50 mM calcium imidazole phosphate and 50 mM acetyl histidine solution in 0.5 M MOPS buffer at pH 7.5 in 9 : 1 H_2O : D_2O containing 0.1 M citric acid and 50 mM HMPA internal standard. Standard deviation provided for each value was based on three repeats. These data are used to plot Main Text Figure 2d.

Time (h)	Imidazole Phosphate (mM)	Orthophosphate (mM)
0.48	47.44 ± 0.25	0.45 ± 0.07
1.35	45.21 ± 0.27	0.71 ± 0.03
2.37	43.44 ± 0.08	0.83 ± 0.04
3.35	42.07 ± 0.00	0.99 ± 0.05
4.50	39.22 ± 0.41	1.32 ± 0.11
5.67	37.60 ± 0.64	1.58 ± 0.01
6.58	36.35 ± 0.71	1.85 ± 0.06
7.57	35.88 ± 0.00	2.03 ± 0.00
8.82	34.32 ± 0.00	2.30 ± 0.00
9.61	32.88 ± 0.42	2.32 ± 0.23
13.35	30.77 ± 0.55	3.11 ± 0.13
17.36	28.59 ± 0.19	3.74 ± 0.03
21.36	25.69 ± 0.55	4.30 ± 0.14
25.32	24.63 ± 0.00	4.96 ± 0.00
29.32	23.48 ± 0.00	5.93 ± 0.00
33.36	22.54 ± 0.48	6.29 ± 0.03
37.36	20.54 ± 1.87	6.65 ± 0.18
41.36	21.07 ± 0.34	7.47 ± 0.03
45.36	20.42 ± 0.24	8.10 ± 0.10

S2.4.1 Characterisation of phosphorylated acetyl histidine intermediate

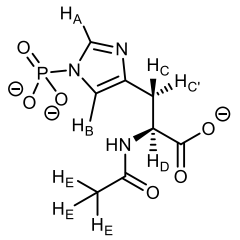

The phosphorylated acetyl histidine intermediate was characterised *in situ*. A solution of 50 mM acetyl histidine and 50 mM calcium imidazole phosphate in 0.5 mL 0.5 M MOPS buffer at pH 7.5 in 9 : 1 H_2O : D_2O containing 0.1 M citric acid and 50 mM HMPA internal standard was prepared according to the procedure in Section S2.2 and S2.4.

^{31}P NMR (202.46 MHz, 0.5 M MOPS + 0.1 M Citric Acid in 9 : 1 H_2O : D_2O at pH 7.5 and 22 °C): *phosphorylated acetyl histidine intermediate* δ (ppm) = - 4.75 (s, 1P). ^{1}H NMR (500.13 MHz, 0.5 M MOPS + 0.1 M Citric Acid in 9 : 1 H_2O : D_2O at pH 7.5 and 22 °C): *phosphorylated acetyl histidine intermediate* δ (ppm) = 8.08 (s, 1H, H_A), 7.09 (s, 1H, H_B), 4.34 (dd, 1H, H_D), 3.06 (dd, 1H, H_C), 2.93 (dd, 1H, $H_{C'}$), 1.90 (s, 1H, H_E). ^{13}C NMR (125.77 MHz, 0.5 M MOPS + 0.1 M Citric Acid in 9 : 1 H_2O : D_2O at pH 7.5 and 22 °C): *phosphorylated acetyl histidine*

intermediate δ (ppm) = 177.5 (s, 1C, COOH), 173.5 (s, 1C, Ac N-C=O), 136.4 (s, 1C, imid C-H$_A$), 132.8 (s, 1C, imid), 118.5 (s, 1C, imid C-H$_B$), 54.8 (s, 1C, C-H$_D$), 28.4 (s, 1C, C-H$_{C+C'}$), 22.1 (s, 1C, C-H$_E$).

S2.5 Hydrolysis of imidazole phosphate with alanine

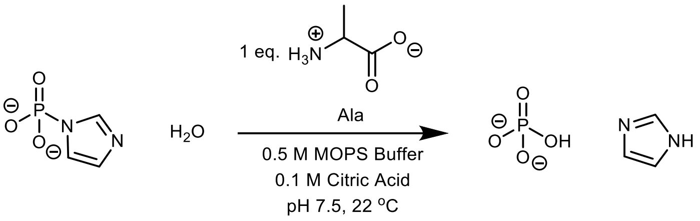

An identical procedure was used as detailed in Section S2.2 but with 50 mM alanine instead of histidine and prepared from 2.2 mg (0.025 mmol, 1 eq) of alanine.

Supporting Figure 6 depicts representative ^{31}P NMR spectra for the reaction. The changes in concentration over time for all phosphate containing species are shown in Supporting Figure 7. Supporting Table 4 show the mean concentration and standard deviation of imidazole phosphate and orthophosphate from the triplicate experiments and were used to plot Main Text Figure 2d.

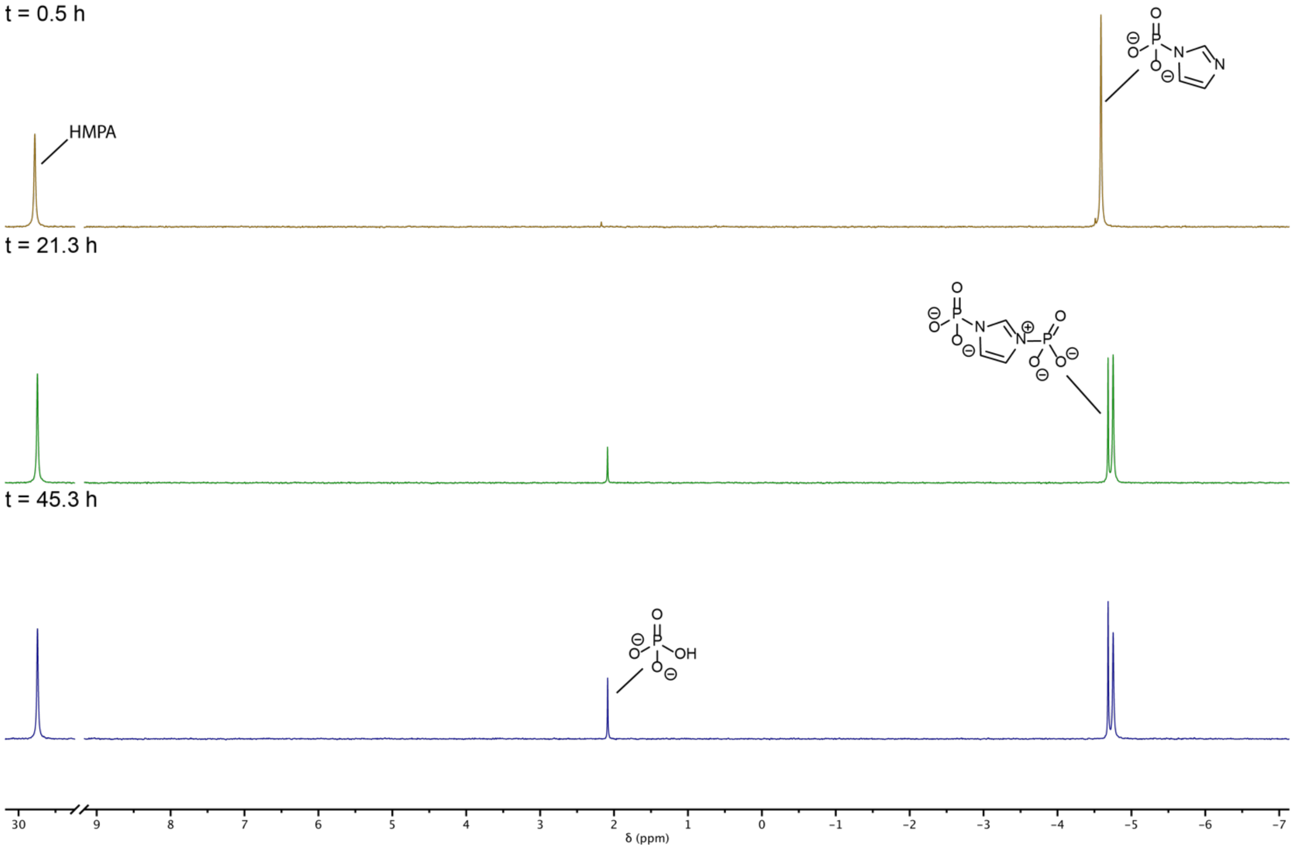

Supporting Figure 6: Representative ^{31}P-NMR spectra over time for the reaction of 50 mM calcium imidazole phosphate and 50 mM alanine solution in 0.5 M MOPS buffer at pH 7.5 in 9 : 1 H_2O : D_2O containing 0.1 M citric acid and 50 mM HMPA internal standard.

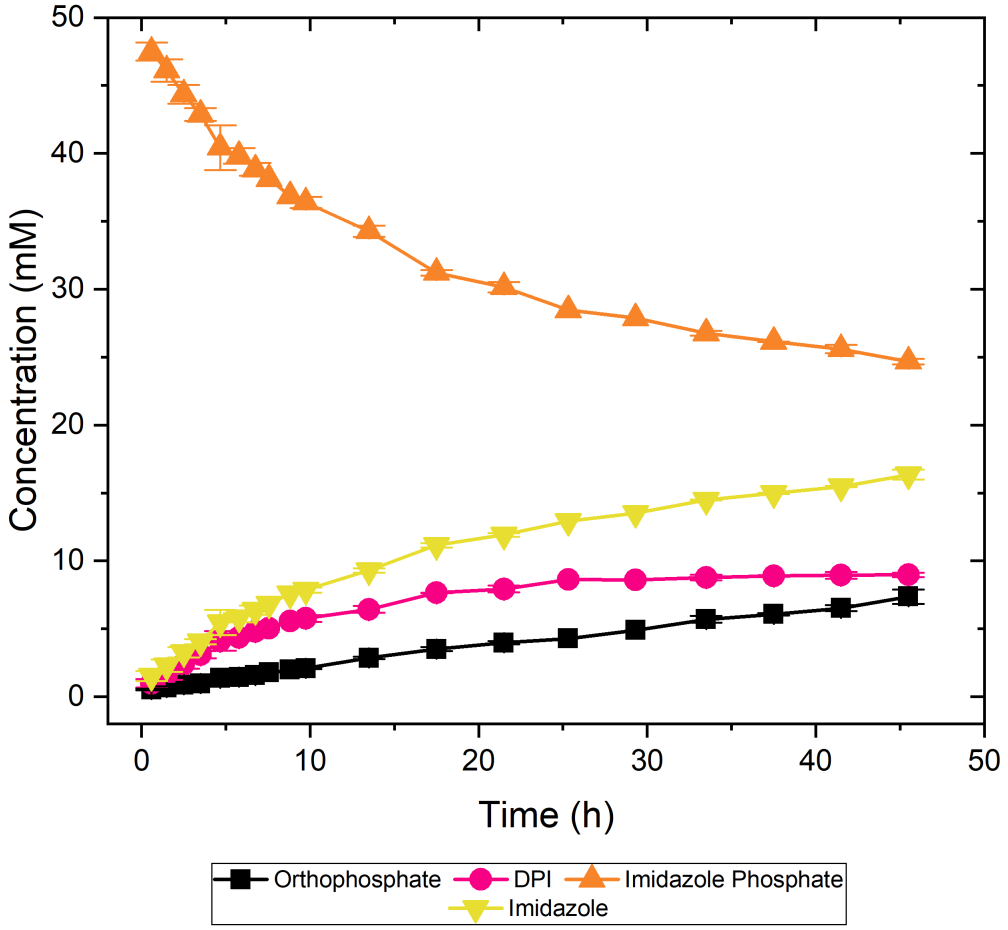

Supporting Figure 7: Changes in concentration over time for the reaction of 50 mM calcium imidazole phosphate and 50 mM alanine solution in 0.5 M MOPS buffer at pH 7.5 in 9 : 1 H_2O : D_2O containing 0.1 M citric acid and 50 mM HMPA internal standard. DPI = Diphosphoimidazole. These data are the mean values and standard deviation based upon duplicate experiments.

Supporting Table 4: Average changes in concentration over time from triplicate experiments for imidazole phosphate and orthophosphate in the ^{31}P-NMR spectra for 50 mM calcium imidazole phosphate and 50 mM alanine solution in 0.5 M MOPS buffer at pH 7.5 in 9 : 1 H_2O : D_2O containing 0.1 M citric acid and 50 mM HMPA internal standard. Standard deviation provided for each value was based on three repeats. These data are used to plot Main Text Figure 2d.

Time (h)	Imidazole Phosphate (mM)			Orthophosphate (mM)		
0.59	47.50	±	0.68	0.53	±	0.06
1.49	46.10	±	0.82	0.67	±	0.10
2.51	44.34	±	0.70	0.88	±	0.08
3.50	42.85	±	0.46	0.97	±	0.06
4.68	40.41	±	1.64	1.37	±	0.20
5.79	39.81	±	0.59	1.45	±	0.09
6.75	38.82	±	0.48	1.57	±	0.20
7.57	38.12	±	0.00	1.78	±	0.00
8.82	36.85	±	0.00	1.99	±	0.00
9.75	36.38	±	0.42	2.09	±	0.06
13.49	34.27	±	0.41	2.86	±	0.09
17.50	31.20	±	0.19	3.50	±	0.14
21.50	30.14	±	0.38	3.98	±	0.13
25.32	28.45	±	0.00	4.28	±	0.00
29.32	27.87	±	0.00	4.90	±	0.00
33.50	26.75	±	0.18	5.70	±	0.24
37.50	26.13	±	0.02	6.08	±	0.09
41.50	25.59	±	0.31	6.53	±	0.21
45.50	24.67	±	0.20	7.38	±	0.53

S2.6 Hydrolysis of imidazole phosphate with His-Asp

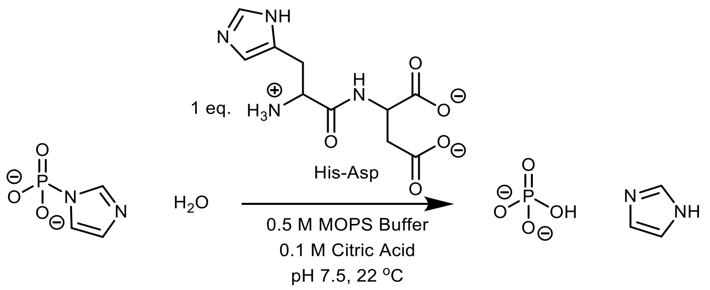

An identical procedure was used as detailed in Section S2.2 but with 50 mM His-Asp instead of histidine and prepared from 6.8 mg (0.025 mmol, 1 eq) of His-Asp.

Supporting Figure 8 depicts representative ^{31}P NMR spectra for the reaction. The changes in concentration over time for all phosphate containing species are shown in Supporting Figure 9. Supporting Table 5 show the mean concentration and standard deviation of imidazole phosphate and orthophosphate from the triplicate experiments and were used to plot Main Text Figure 2d.

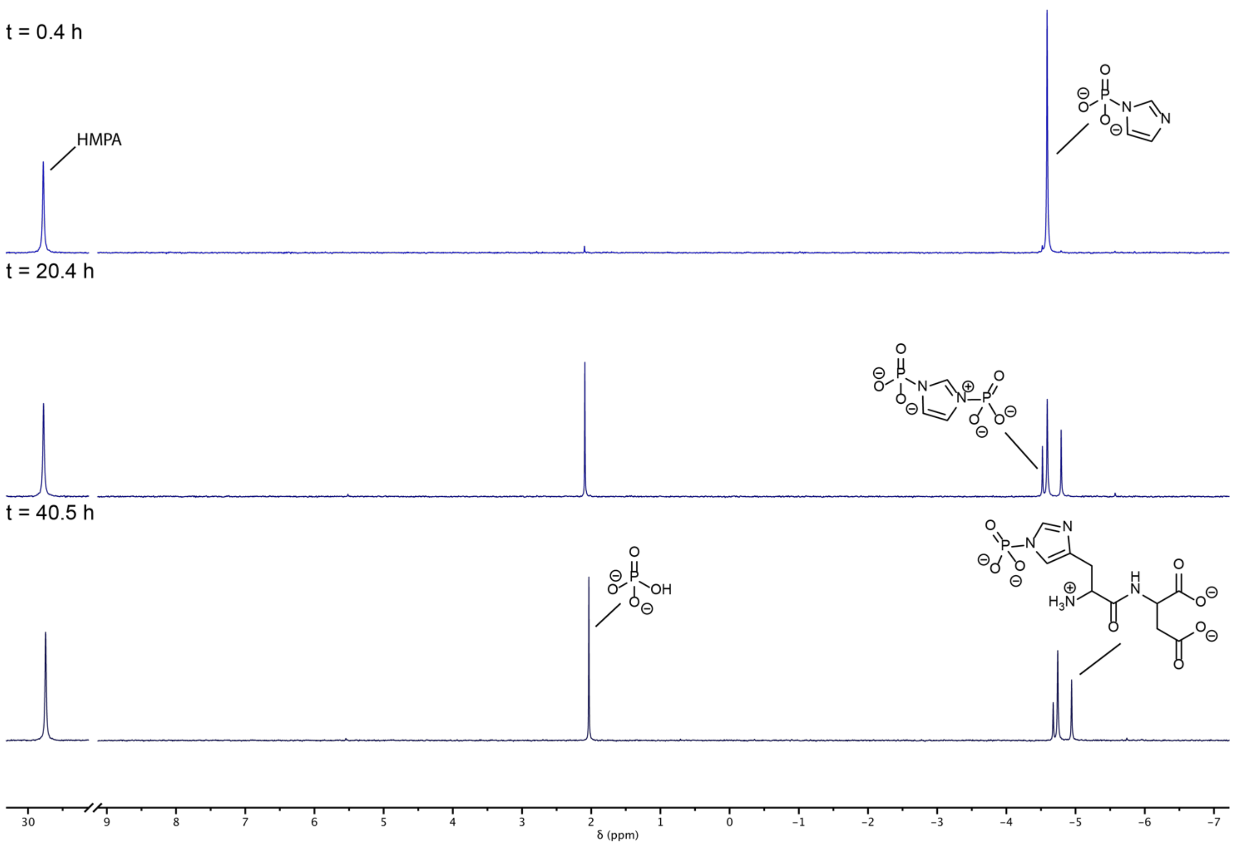

Supporting Figure 8: Representative ^{31}P-NMR spectra over time for the reaction of 50 mM calcium imidazole phosphate and 50 mM His-Asp solution in 0.5 M MOPS buffer at pH 7.5 in 9 : 1 H_2O : D_2O containing 0.1 M citric acid and 50 mM HMPA internal standard.

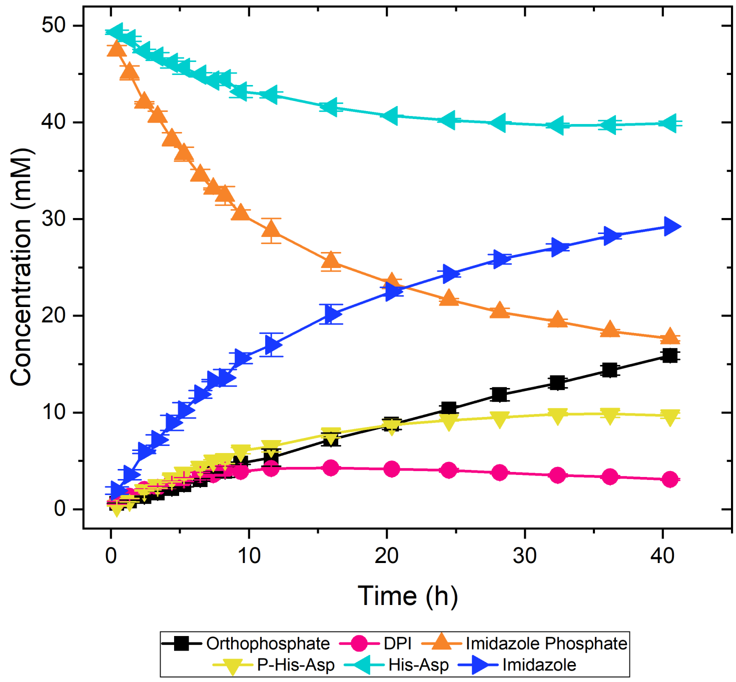

Supporting Figure 9: Changes in concentration over time for the reaction of 50 mM calcium imidazole phosphate and 50 mM His-Asp solution in 0.5 M MOPS buffer at pH 7.5 in 9 : 1 H_2O : D_2O containing 0.1 M citric acid and 50 mM HMPA internal standard. DPI = Diphosphoimidazole. These data are the mean values and standard deviation based upon triplicate experiments.

Supporting Table 5: Average changes in concentration over time from triplicate experiments for imidazole phosphate and orthophosphate in the ^{31}P-NMR spectra for 50 mM calcium imidazole phosphate and 50 mM His-Asp solution in 0.5 M MOPS buffer at pH 7.5 in 9 : 1 H_2O : D_2O containing 0.1 M citric acid and 50 mM HMPA internal standard. Standard deviation provided for each value was based on three repeats. These data are used to plot Main Text Figure 2d.

Time (h)	Imidazole Phosphate (mM)	Orthophosphate (mM)
0.43	47.41 ± 0.54	0.59 ± 0.03
1.35	45.11 ± 0.71	0.85 ± 0.11
2.42	42.03 ± 0.10	1.29 ± 0.08
3.38	40.59 ± 0.56	1.66 ± 0.19
4.42	38.25 ± 0.68	2.11 ± 0.11
5.30	36.71 ± 0.71	2.52 ± 0.21
6.47	34.54 ± 0.59	3.03 ± 0.13
7.42	33.13 ± 0.10	3.81 ± 0.05
8.26	32.39 ± 0.97	3.88 ± 0.57
9.42	30.48 ± 0.47	4.76 ± 0.12
11.62	28.77 ± 1.28	5.34 ± 0.88
15.95	25.57 ± 0.94	7.22 ± 0.65
20.36	23.35 ± 0.41	8.77 ± 0.52
24.51	21.65 ± 0.14	10.34 ± 0.37
28.17	20.37 ± 0.40	11.82 ± 0.62
32.37	19.41 ± 0.24	13.04 ± 0.48
36.17	18.40 ± 0.19	14.37 ± 0.49
40.53	17.67 ± 0.26	15.88 ± 0.38
46.58	17.15 ± 0.00	17.35 ± 0.00

S2.6.1 Characterisation of phosphorylated His-Asp intermediate

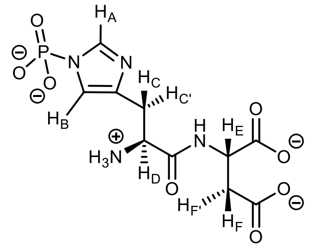

The phosphorylated His-Asp intermediate was characterised *in situ*. A solution of 50 mM His-Asp and 50 mM calcium imidazole phosphate in 0.5 mL 0.5 M MOPS buffer at pH 7.5 in 9 : 1 H_2O : D_2O containing 0.1 M citric acid and 50 mM HMPA internal standard was prepared according to the procedure in Section S2.2 and S2.4.

^{31}P NMR (202.46 MHz, 0.5 M MOPS + 0.1 M Citric Acid in 9 : 1 H_2O : D_2O at pH 7.5 and 22 °C): *phosphorylated His-Asp intermediate* δ (ppm) = - 4.79 (s, 1P). ^{1}H NMR (500.13 MHz, 0.5 M MOPS + 0.1 M Citric Acid in 9 : 1 H_2O : D_2O at pH 7.5 and 22 °C): *phosphorylated His-Asp intermediate* δ (ppm) = 8.03 (s, 1H, H_A), 7.18 (s, 1H, H_B), 4.36 (dd, 1H, H_E), 3.98 (dd, 1H, H_D), 3.07 (dd, 1H, $H_{C+C'}$), 2.63 (1H, H_F), 2.45 (1H, $H_{F'}$). ^{13}C NMR (125.77 MHz, 0.5 M MOPS + 0.1 M Citric Acid in 9 : 1 H_2O : D_2O at pH 7.5 and 22 °C): *phosphorylated His-Asp intermediate*

δ (ppm) = 182.0 (s, 1C, COOH), 178.8 (s, 1C, COOH), 171.7 (s, 1C, Amide N-C=O), 138.2 (s, 1C, imid C-H_A), 131.3 (s, 1C, imid), 119.9 (s, 1C, imid C-H_B), 53.3 (s, 1C, C-H_E), 53.4 (s, 1C, C-H_D), 45.4 (s, 1C, C-H_F), 29.4 (s, 1C, C-H_C).

S2.7 Hydrolysis of imidazole phosphate with His-Lys

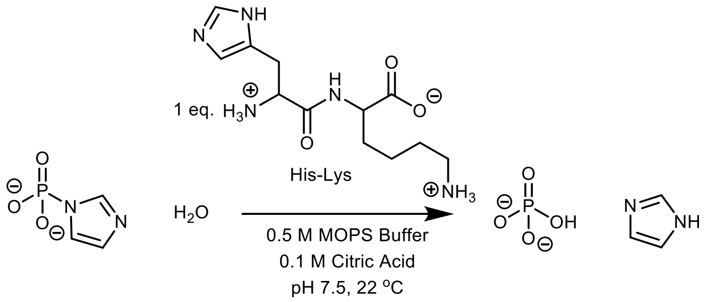

An identical procedure was used as detailed in Section S2.2 but with 50 mM His-Lys instead of histidine and prepared from 9.1 mg (0.025 mmol, 1 eq) of His-Lys.HBr.

Supporting Figure 10 depicts representative ^{31}P NMR spectra for the reaction. The changes in concentration over time for all phosphate containing species are shown in Supporting Figure 11. Supporting Table 6 show the mean concentration and standard deviation of imidazole phosphate and orthophosphate from the triplicate experiments and were used to plot Main Text Figure 2d.

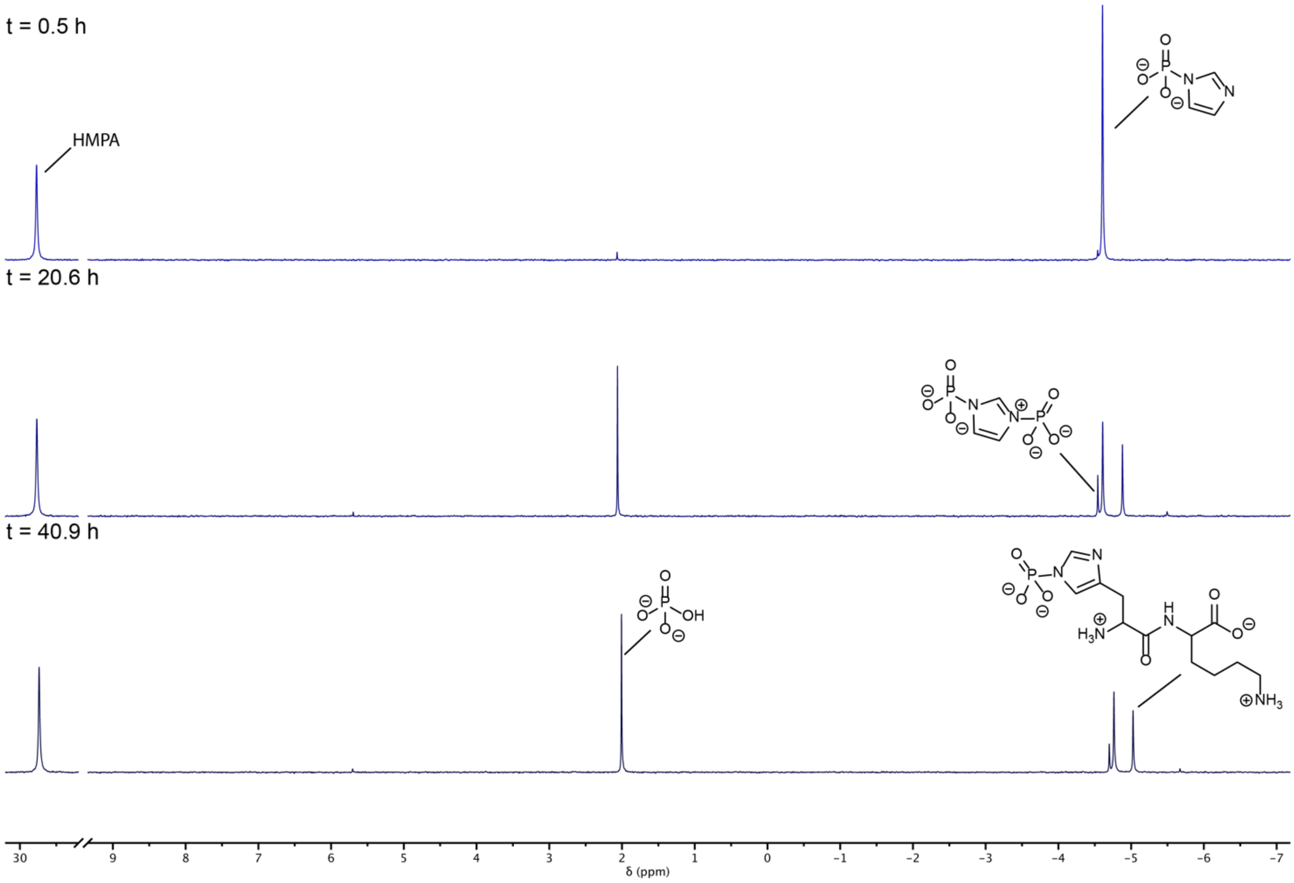

Supporting Figure 10: Representative ^{31}P-NMR spectra over time for the reaction of 50 mM calcium imidazole phosphate and 50 mM His-Lys solution in 0.5 M MOPS buffer at pH 7.5 in 9 : 1 H_2O : D_2O containing 0.1 M citric acid and 50 mM HMPA internal standard.

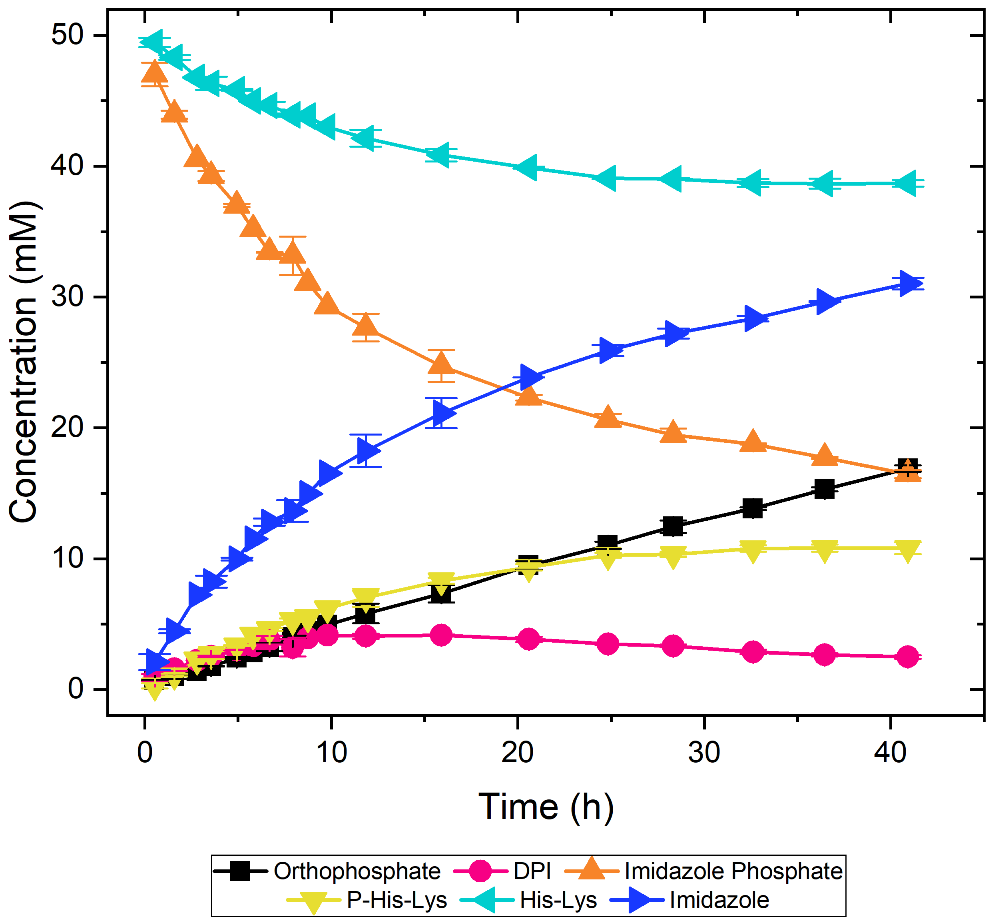

Supporting Figure 11: Changes in concentration over time for the reaction of 50 mM calcium imidazole phosphate and 50 mM His-Lys solution in 0.5 M MOPS buffer at pH 7.5 in 9 : 1 H_2O : D_2O containing 0.1 M citric acid and 50 mM HMPA internal standard. DPI = Diphosphoimidazole. These data are the mean values and standard deviation based upon duplicate experiments.

Supporting Table 6: Average changes in concentration over time from triplicate experiments for imidazole phosphate and orthophosphate in the ^{31}P-NMR spectra for 50 mM calcium imidazole phosphate and 50 mM His-Lys solution in 0.5 M MOPS buffer at pH 7.5 in 9 : 1 H_2O : D_2O containing 0.1 M citric acid and 50 mM HMPA internal standard. Standard deviation provided for each value was based on three repeats. These data are used to plot Main Text Figure 2d.

Time (h)	Imidazole Phosphate (mM)			Orthophosphate (mM)		
0.53	47.01	±	0.91	0.70	±	0.02
1.59	43.95	±	0.31	1.01	±	0.18
2.80	40.54	±	0.00	1.37	±	0.00
3.56	39.25	±	0.38	1.78	±	0.03
4.93	37.00	±	0.13	2.42	±	0.08
5.80	35.17	±	0.00	2.82	±	0.00
6.68	33.44	±	0.03	3.23	±	0.32
7.93	33.15	±	1.48	4.00	±	0.12
8.73	31.08	±	0.00	4.44	±	0.00
9.78	29.30	±	0.00	4.93	±	0.00
11.85	27.67	±	1.04	5.80	±	0.75
15.89	24.72	±	1.21	7.34	±	0.67
20.59	22.30	±	0.21	9.47	±	0.32
24.83	20.62	±	0.48	11.01	±	0.28
28.33	19.47	±	0.49	12.45	±	0.46
32.62	18.77	±	0.04	13.83	±	0.10
36.46	17.70	±	0.07	15.31	±	0.16
40.92	16.48	±	0.31	16.89	±	0.24
46.70	15.87	±	0.00	18.38	±	0.00

S2.7.1 Characterisation of phosphorylated His-Lys intermediate

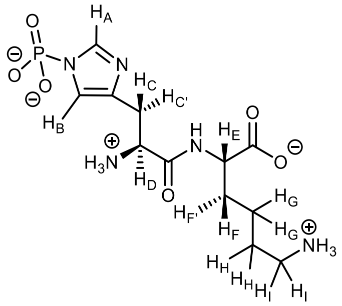

The phosphorylated His-Lys intermediate was characterised *in situ*. A solution of 50 mM His-Lys and 50 mM calcium imidazole phosphate in 0.5 mL 0.5 M MOPS buffer at pH 7.5 in 9 : 1 H_2O : D_2O containing 0.1 M citric acid and 50 mM HMPA internal standard was prepared according to the procedure in Section S2.2 and S2.4.

^{31}P NMR (202.46 MHz, 0.5 M MOPS + 0.1 M Citric Acid in 9 : 1 H_2O : D_2O at pH 7.5 and 22 °C): *phosphorylated His-Lys intermediate* δ (ppm) = - 4.87 (s, 1P). ^{1}H NMR (500.13 MHz, 0.5 M MOPS + 0.1 M Citric Acid in 9 : 1 H_2O : D_2O at pH 7.5 and 22 °C): *phosphorylated His-Lys intermediate* δ (ppm) = 7.98 (s, 1H, H_A), 7.14 (s, 1H, H_B), 4.05 (s, 1H, H_E), 3.92 (dd, 1H, H_D), 3.02 (dd, 2H, $H_{C+C'}$), 2.91 (t, 2H, $H_{I+I'}$), 1.72 (m, 1H, $H_{F/F'}$), 1.65 (m, 1H,

$H_{F'/F}$), 1.28 (m, 2H, $H_{G+G'}$), 1.60 (m, 2H, $H_{H+H'}$). ^{13}C NMR (125.77 MHz, 0.5 M MOPS + 0.1 M Citric Acid in 9 : 1 H_2O : D_2O at pH 7.5 and 22 °C): *phosphorylated His-Lys intermediate* δ (ppm) = 178.5 (s, 1C, COOH), 172.5 (s, 1C, Amide N-C=O), 137.8 (s, 1C, imid C-H_A), 132.2 (s, 1C, imid), 119.3 (s, 1C, imid C-H_B), 55.2 (s, 1C, C-H_E), 53.7 (s, 1C, C-H_D), 39.5 (s, 1C, C-H_I), 29.8 (s, 1C, C-$H_{C+C'}$), 30.8 (s, 1C, C-$H_{F+F'}$), 22.2 (s, 1C, C-H_G), 26.5 (s, 1C, C-H_H).

S2.8 Hydrolysis of imidazole phosphate with His-Gly-Gly

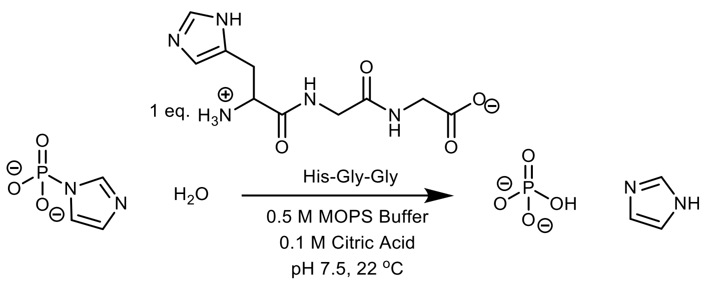

An identical procedure was used as detailed in Section S2.2 but with 50 mM His-Gly-Gly instead of histidine and prepared from 6.7 mg (0.025 mmol, 1 eq) of His-Gly-Gly.

Supporting Figure 12 depicts representative ^{31}P NMR spectra for the reaction. The changes in concentration over time for all phosphate containing species are shown in Supporting Figure 13. Supporting Table 7 show the mean concentration and standard deviation of imidazole phosphate and orthophosphate from the triplicate experiments and were used to plot Main Text Figure 2d.

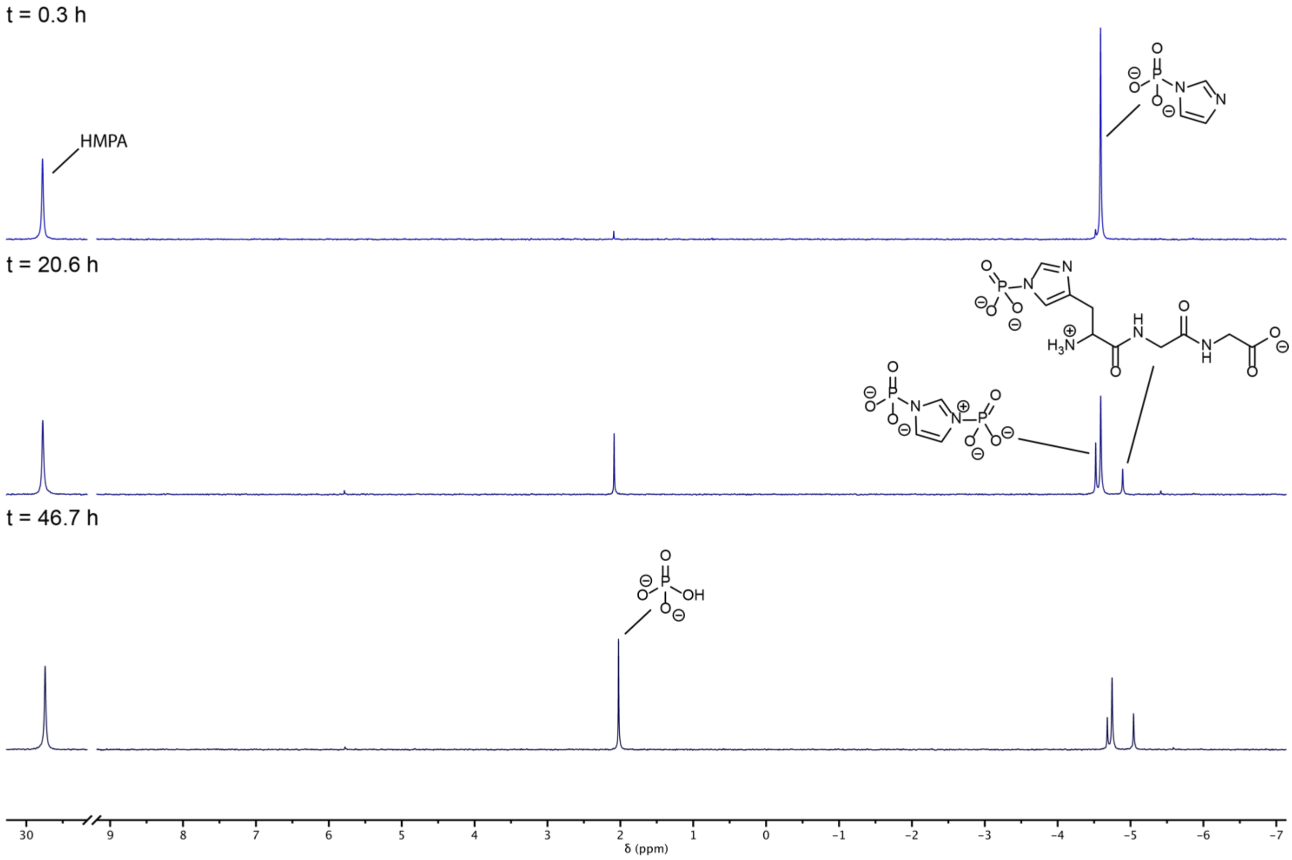

Supporting Figure 12: Representative ^{31}P-NMR spectra over time for the reaction of 50 mM calcium imidazole phosphate and 50 mM His-Gly-Gly solution in 0.5 M MOPS buffer at pH 7.5 in 9 : 1 H_2O : D_2O containing 0.1 M citric acid and 50 mM HMPA internal standard.

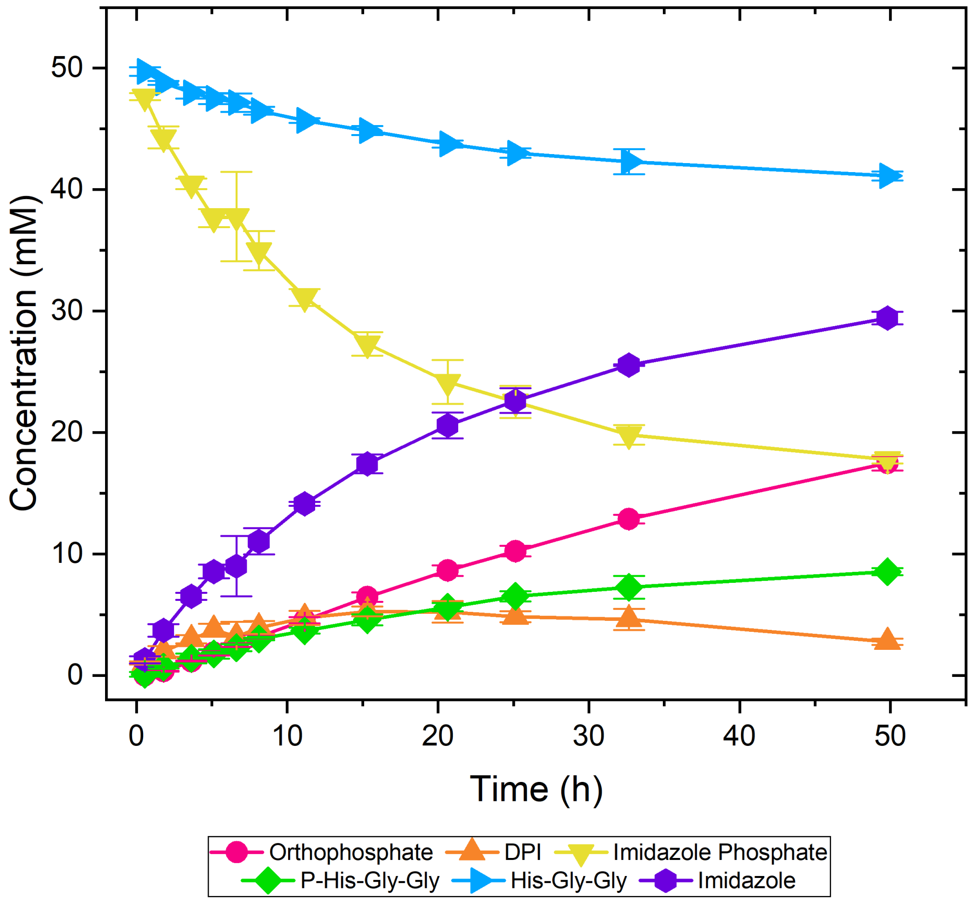

Supporting Figure 13: Changes in concentration over time for the reaction of 50 mM calcium imidazole phosphate and 50 mM His-Gly-Gly solution in 0.5 M MOPS buffer at pH 7.5 in 9 : 1 H_2O : D_2O containing 0.1 M citric acid and 50 mM HMPA internal standard. These data are the mean values and standard deviation based upon triplicate experiments.

Supporting Table 7: Average changes in concentration over time from triplicate experiments for imidazole phosphate and orthophosphate in the ^{31}P-NMR spectra for 50 mM calcium imidazole phosphate and 50 mM His-Gly-Gly solution in 0.5 M MOPS buffer at pH 7.5 in 9 : 1 H_2O : D_2O containing 0.1 M citric acid and 50 mM HMPA internal standard. Standard deviation provided for each value was based on three repeats. These data are used to plot Main Text Figure 2d.

Time (h)	Imidazole Phosphate (mM)			Orthophosphate (mM)		
0.56	47.65	±	0.28	0.00	±	0.00
1.79	44.30	±	0.91	0.37	±	0.04
3.64	40.48	±	0.44	1.17	±	0.07
5.13	37.66	±	0.75	1.88	±	0.24
6.63	37.78	±	3.67	2.51	±	0.15
8.13	34.99	±	1.61	3.20	±	0.17
11.16	31.13	±	0.70	4.55	±	0.26
15.31	27.30	±	0.98	6.45	±	0.39
20.64	24.17	±	1.81	8.64	±	0.43
25.13	22.53	±	1.33	10.24	±	0.44
32.66	19.82	±	0.82	12.87	±	0.35
49.80	17.79	±	0.34	17.48	±	0.60

S2.8.1 Characterisation of phosphorylated His-Gly-Gly intermediate

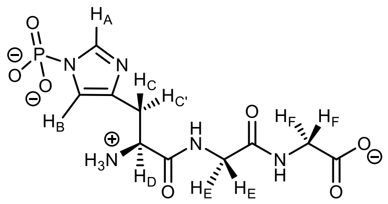

The phosphorylated His-Gly-Gly intermediate was characterised *in situ*. A solution of 50 mM His-Gly-Gly and 50 mM calcium imidazole phosphate in 0.5 mL 0.5 M MOPS buffer at pH 7.5 in 9 : 1 H_2O : D_2O containing 0.1 M citric acid and 50 mM HMPA internal standard was prepared according to the procedure in Section S2.2 and S2.4.

^{31}P NMR (202.46 MHz, 0.5 M MOPS + 0.1 M Citric Acid in 9 : 1 H_2O : D_2O at pH 7.5 and 22 °C): *phosphorylated His-Gly-Gly intermediate* δ (ppm) = - 4.89 (s, 1P). ^{1}H NMR (500.13 MHz, 0.5 M MOPS + 0.1 M Citric Acid in 9 : 1 H_2O : D_2O at pH 7.5 and 22 °C): *phosphorylated His-Gly-Gly intermediate* δ (ppm) = 7.85 (s, 1H, H_A), 7.11 (s, 1H, H_B), 3.97 (dd, 1H, H_D), 3.01 (dd, 2H, $H_{C+C'}$), 3.89 (s, 1H, $H_{E+E'}$), 3.71 (d, 1H, $H_{F+F'}$). ^{13}C NMR (125.77 MHz, 0.5 M MOPS + 0.1 M Citric Acid in 9 : 1 H_2O : D_2O at pH 7.5 and 22 °C): *phosphorylated His-Gly-Gly intermediate* δ (ppm) = 176.4 (s, 1C, COOH), 174.0 (s, 1C, His-Gly Amide N-C=O), 171.0 (s, 1C, Gly-Gly Amide N-C=O), 138.3 (s, 1C, imid C-H_A), 133.0 (s, 1C, imid), 119.0 (s, 1C, imid C-H_B), 53.9 (s, 1C, C-H_D), 30.1 (s, 1C, C-$H_{C+C'}$), 43.2 (s, 1C, C-H_F), 42.6 (s, 1C, C-H_E).

S2.9 Hydrolysis of imidazole phosphate with hercynine

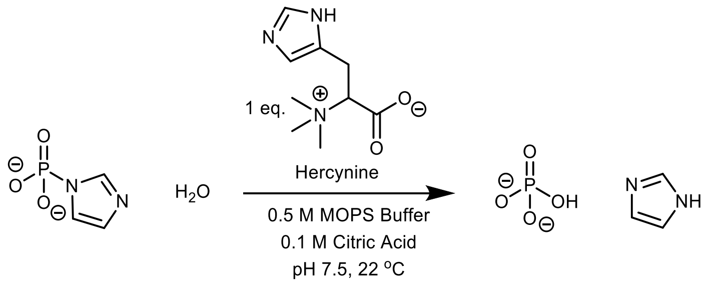

An identical procedure was used as detailed in Section S2.2 but with 50 mM hercynine instead of histidine and prepared from 4.9 mg (0.025 mmol, 1 eq) of hercynine.

Supporting Figure 14 depicts representative ^{31}P NMR spectra for the reaction. The changes in concentration over time for all phosphate containing species are shown in Supporting Figure 15. Supporting Table 8 show the mean concentration and standard deviation of imidazole phosphate and orthophosphate from the triplicate experiments and were used to plot Main Text Figure 2d.

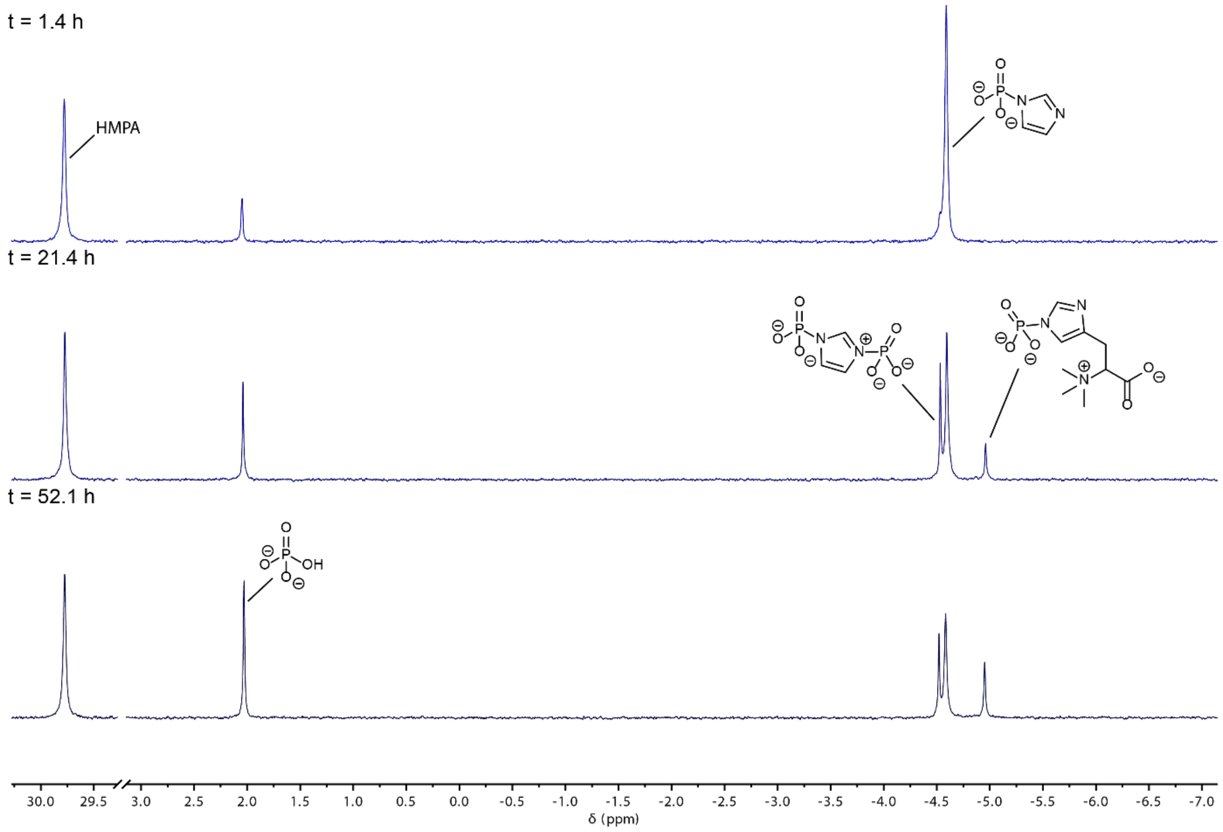

Supporting Figure 14: Representative ^{31}P-NMR spectra over time for the reaction of 50 mM calcium imidazole phosphate and 50 mM hercynine solution in 0.5 M MOPS buffer at pH 7.5 in 9 : 1 H_2O : D_2O containing 0.1 M citric acid and 50 mM HMPA internal standard.

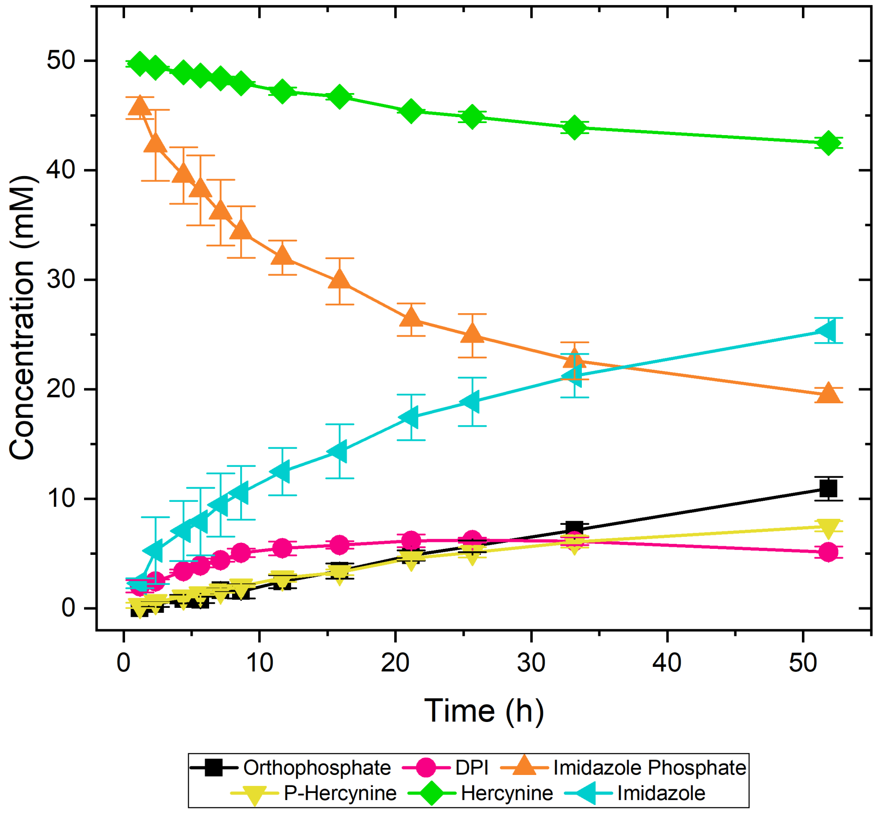

Supporting Figure 15: Changes in concentration over time for the reaction of 50 mM calcium imidazole phosphate and 50 mM hercynine solution in 0.5 M MOPS buffer at pH 7.5 in 9 : 1 H_2O : D_2O containing 0.1 M citric acid and 50 mM HMPA internal standard. These data are the mean values and standard deviation based upon triplicate experiments.

Supporting Table 8: Average changes in concentration over time from triplicate experiments for imidazole phosphate and orthophosphate in the ^{31}P-NMR spectra for 50 mM calcium imidazole phosphate and 50 mM hercynine solution in 0.5 M MOPS buffer at pH 7.5 in 9 : 1 H_2O : D_2O containing 0.1 M citric acid and 50 mM HMPA internal standard. Standard deviation provided for each value was based on three repeats. These data are used to plot Main Text Figure 2d.

Time (h)	Imidazole Phosphate (mM)			Orthophosphate (mM)		
1.19	42.58	±	0.23	0.00	±	0.86
2.34	40.75	±	0.11	0.47	±	0.52
4.41	38.13	±	0.08	0.83	±	0.39
5.65	36.74	±	0.18	0.97	±	0.53
7.14	34.79	±	0.17	1.69	±	0.80
8.64	33.09	±	0.16	1.73	±	0.16
11.68	30.91	±	0.26	2.38	±	1.36
15.90	28.80	±	0.06	3.40	±	0.88
21.16	25.42	±	0.33	4.79	±	1.05
25.65	24.01	±	0.29	5.60	±	0.70
33.18	21.77	±	0.66	7.09	±	1.01
51.87	18.78	±	0.21	10.64	±	1.09

S2.9.1 Characterisation of phosphorylated hercynine intermediate

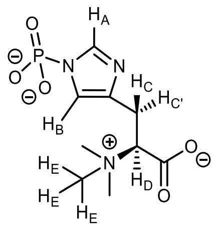

The phosphorylated hercynine intermediate was characterised *in situ*. A solution of 50 mM hercynine and 50 mM calcium imidazole phosphate in 0.5 mL 0.5 M MOPS buffer at pH 7.5 in 9 : 1 H_2O : D_2O containing 0.1 M citric acid and 50 mM HMPA internal standard was prepared according to the procedure in Section S2.2 and S2.4.

^{31}P NMR (202.46 MHz, 0.5 M MOPS + 0.1 M Citric Acid in 9 : 1 H_2O : D_2O at pH 7.5 and 22 °C): *phosphorylated hercynine intermediate* δ (ppm) = - 4.99 (s, 1P). ^{1}H NMR (500.13 MHz, 0.5 M MOPS + 0.1 M Citric Acid in 9 : 1 H_2O : D_2O at pH 7.5 and 22 °C): *phosphorylated hercynine intermediate* δ (ppm) = 7.75 (s, 1H, H_A), 7.02 (s, 1H, H_B), 3.82 (dd, 1H, H_D), 3.17 (1H, H_C), 3.12 (1H, $H_{C'}$), 3.18 (s, 9H, H_E). ^{13}C NMR (125.77 MHz, 0.5 M MOPS + 0.1 M Citric Acid in 9 : 1 H_2O : D_2O at pH 7.5 and 22 °C): *phosphorylated hercynine intermediate* δ (ppm) = 170.9 (s, 1C, COOH), 138.7 (s, 1C, imid C-H_A), 133.1 (s, 1C, imid), 118.5 (s, 1C, imid C-H_B), 78.3 (s, 1C, C-H_D), 25.1 (s, 1C, C-$H_{C+C'}$), 52.0 (s, 1C, C-H_E).

S2.10 Uncatalysed hydrolysis of imidazole phosphate

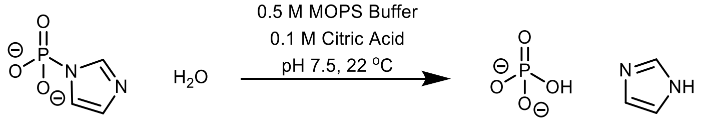

An identical procedure was used as detailed in Section S2.2 but with no histidyl catalyst added.

Supporting Figure 16 depicts representative ^{31}P NMR spectra for the reaction. The changes in concentration over time for all phosphate containing species are shown in Supporting Figure 17. Supporting Table 9 show the mean concentration and standard deviation of imidazole phosphate and orthophosphate from the triplicate experiments and were used to plot Main Text Figure 2d.

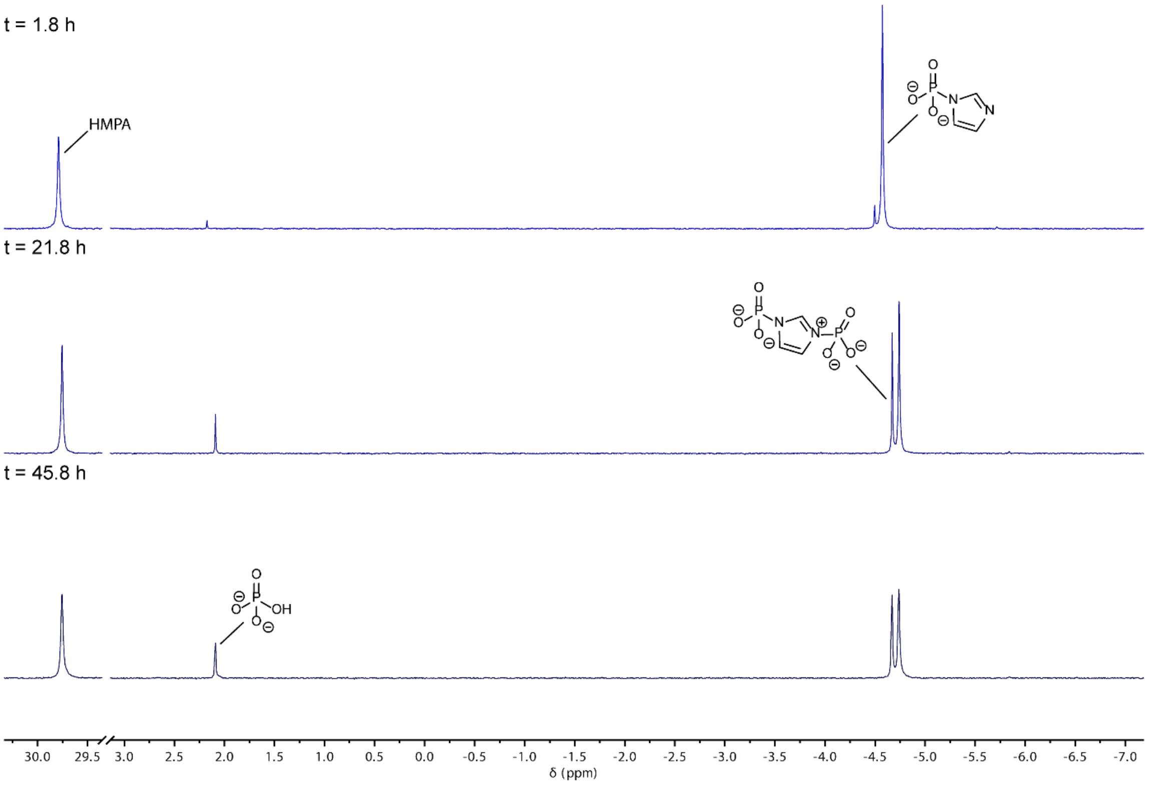

Supporting Figure 16: Representative ^{31}P-NMR spectra over time for the reaction of 50 mM calcium imidazole phosphate in 0.5 M MOPS buffer at pH 7.5 in 9 : 1 H_2O : D_2O containing 0.1 M citric acid and 50 mM HMPA internal standard.

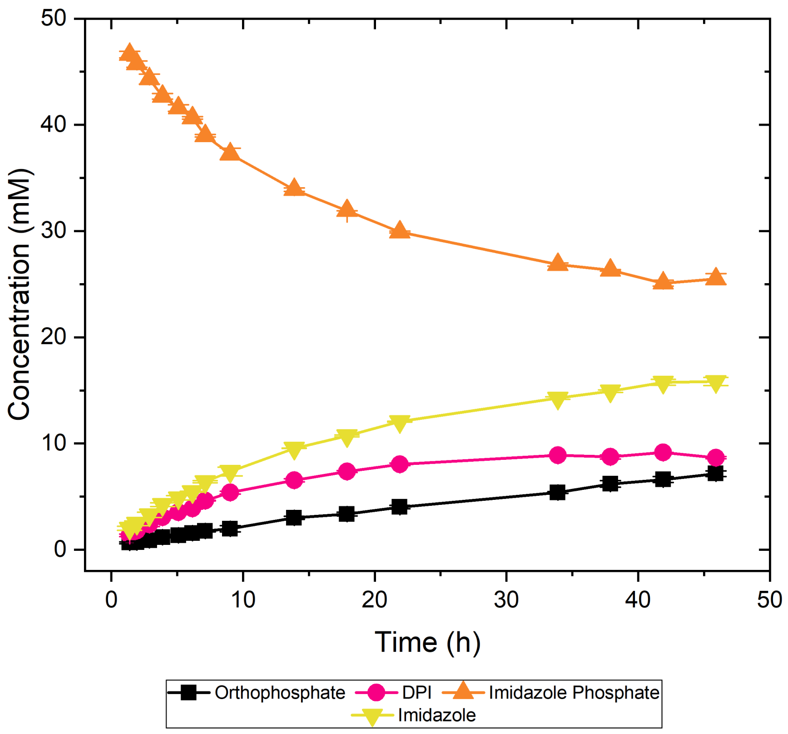

Supporting Figure 17: Changes in concentration over time for the reaction of 50 mM calcium imidazole phosphate in 0.5 M MOPS buffer at pH 7.5 in 9 : 1 H_2O : D_2O containing 0.1 M citric acid and 50 mM HMPA internal standard. DPI = Diphosphoimidazole. These data are the mean values and standard deviation based upon duplicate experiments.

Supporting Table 9: Average changes in concentration over time from triplicate experiments for imidazole phosphate and orthophosphate in the ^{31}P-NMR spectra for 50 mM calcium imidazole phosphate in 0.5 M MOPS buffer at pH 7.5 in 9 : 1 H_2O : D_2O containing 0.1 M citric acid and 50 mM HMPA internal standard. Standard deviation provided for each value was based on three repeats. These data are used to plot Main Text Figure 2d.

Time (h)	Imidazole Phosphate (mM)			Orthophosphate (mM)		
1.80	46.62	±	0.31	0.66	±	0.09
1.90	45.72	±	0.30	0.70	±	0.15
2.80	44.31	±	0.47	0.88	±	0.03
3.80	42.68	±	0.27	1.19	±	0.07
5.05	41.58	±	0.30	1.36	±	0.10
6.07	40.65	±	0.11	1.56	±	0.01
7.07	38.97	±	0.12	1.77	±	0.08
8.07	37.24	±	0.54	1.98	±	0.28
9.32	36.95	±	0.00	1.78	±	0.00
10.32	33.74	±	0.00	2.08	±	0.00
13.80	33.89	±	0.15	3.00	±	0.14
17.82	31.91	±	0.00	3.36	±	0.17
21.82	29.90	±	0.09	4.02	±	0.16
25.80	29.15	±	0.00	4.31	±	0.00
29.80	27.41	±	0.00	4.85	±	0.00
33.82	26.84	±	0.14	5.38	±	0.09
37.82	26.32	±	0.07	6.19	±	0.31
41.82	25.09	±	0.28	6.61	±	0.29
45.82	25.49	±	0.49	7.16	±	0.27

S2.11 Hydrolysis of imidazole phosphate with histidine with 1.0 M MOPS

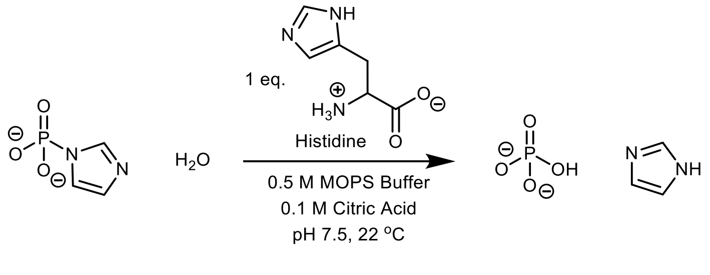

The experiment was performed as detailed in Section S2.2 but with a 1.0 M MOPS solution and 200 mM citric acid at pH 7.5.

Supporting Figure 18 depicts representative ^{31}P NMR spectra for the reaction. The changes in concentration over time for all phosphate containing species are shown in Supporting Figure 19. Supporting Table 10 show the mean concentration and standard deviation of imidazole phosphate and orthophosphate from the triplicate experiments and were used to plot Main Text Figure 2d.

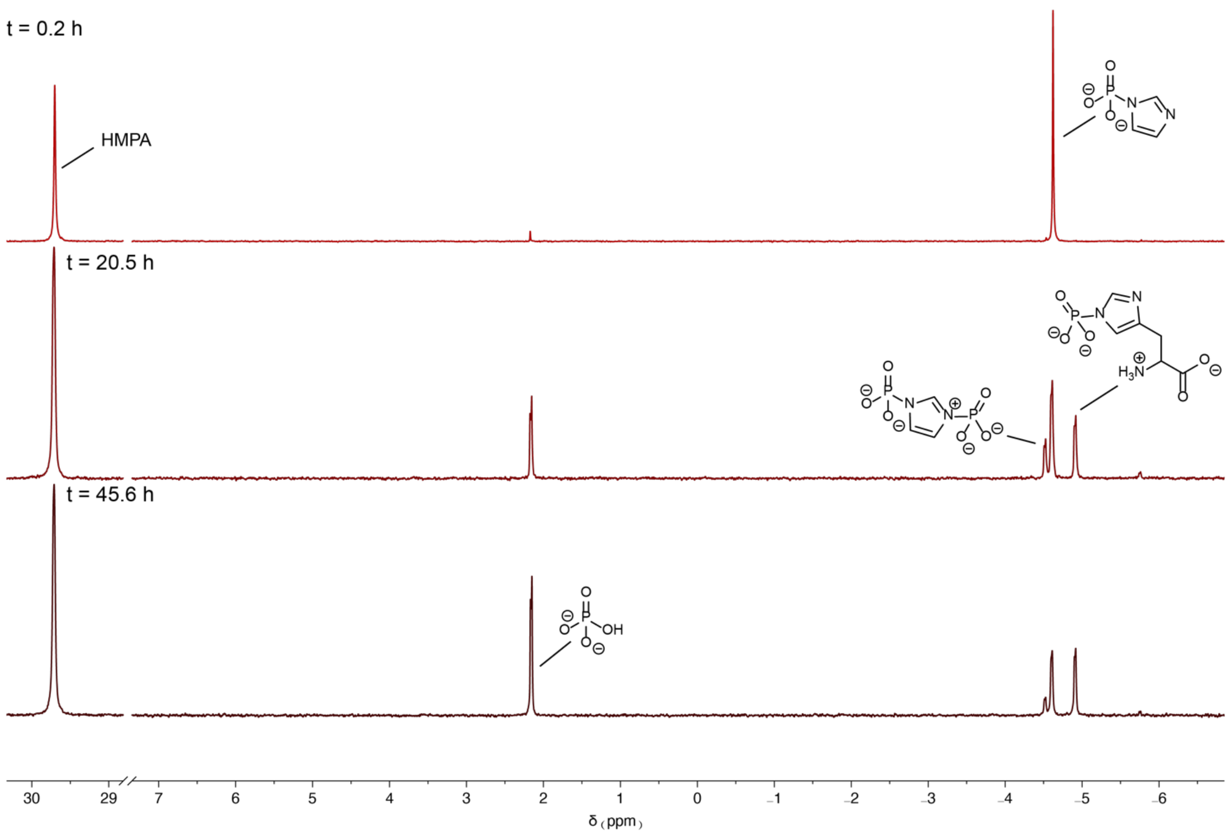

Supporting Figure 18: Representative ^{31}P-NMR spectra over time for the reaction of 50 mM calcium imidazole phosphate and 50 mM histidine solution in 1.0 M MOPS buffer at pH 7.5 in 9 : 1 H_2O : D_2O containing 0.2 M citric acid and 50 mM HMPA internal standard.

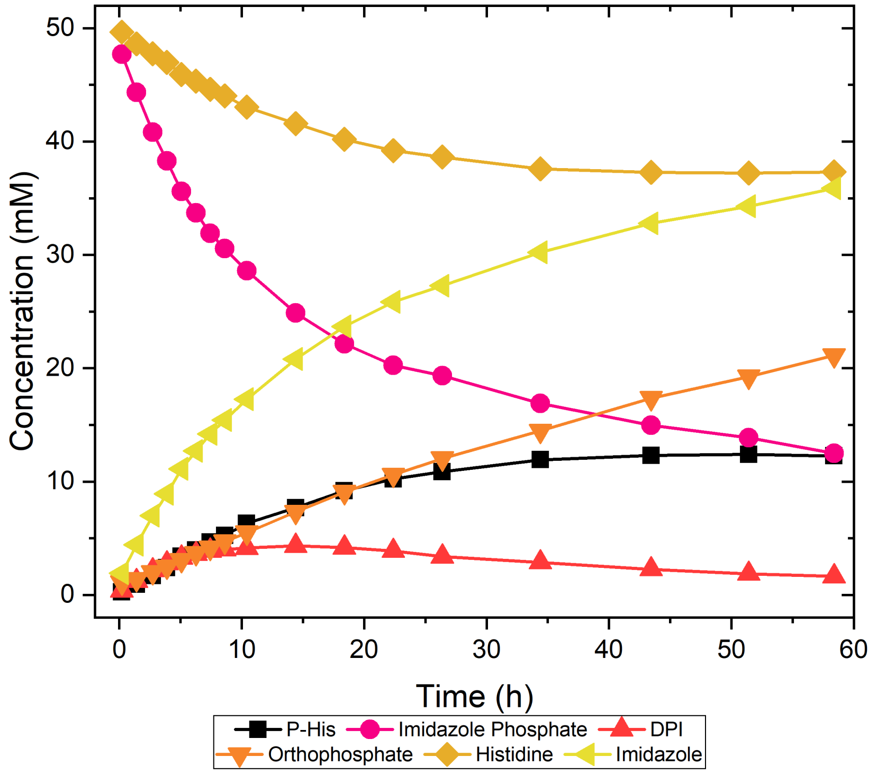

Supporting Figure 19: Representative changes in concentration over time for the reaction of 50 mM calcium imidazole phosphate and 50 mM histidine solution in 1.0 M MOPS buffer at pH 7.5 in 9 : 1 H_2O : D_2O containing 0.2 M citric acid and 50 mM HMPA internal standard. DPI = Diphosphoimidazole.

Supporting Table 10: Average changes in concentration over time from triplicate experiments for imidazole phosphate and orthophosphate in the ^{31}P-NMR spectra for 50 mM calcium imidazole phosphate and 50 mM histidine solution in 1.0 M MOPS buffer at pH 7.5 in 9 : 1 H_2O : D_2O containing 0.2 M citric acid and 50 mM HMPA internal standard. Standard deviation provided for each value was based on two repeats. These data are used to plot Main Text Figure 2d.

Time (h)	Imidazole Phosphate (mM)	Orthophosphate (mM)
0.24	47.78 ± 0.12	1.15 ± 0.06
1.06	44.13 ± 0.30	1.45 ± 0.09
2.11	40.95 ± 0.17	1.93 ± 0.11
3.03	38.14 ± 0.22	2.44 ± 0.06
4.15	35.71 ± 0.15	3.06 ± 0.01
5.29	33.80 ± 0.15	3.64 ± 0.07
6.28	32.01 ± 0.15	4.07 ± 0.12
7.23	30.59 ± 0.05	4.64 ± 0.10
8.57	28.88 ± 0.41	5.27 ± 0.33
9.33	25.79 ± 1.30	6.63 ± 0.99
13.08	22.83 ± 0.97	8.54 ± 0.76
17.08	21.05 ± 1.11	9.92 ± 0.94
21.08	19.46 ± 0.16	11.50 ± 0.77
25.07	17.63 ± 1.03	13.50 ± 1.37
29.08	15.73 ± 1.07	16.10 ± 1.79
45.08	14.18 ± 0.47	18.36 ± 1.26

S2.12 Hydrolysis of imidazole phosphate with histidine with 0.75 M MOPS

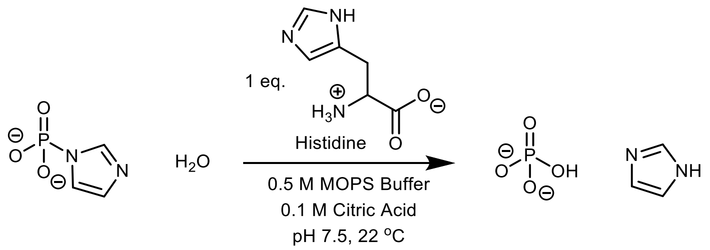

The experiment was performed as detailed in Section S2.2 but with a 0.75 M MOPS solution and 200 mM citric acid at pH 7.5.

Supporting Figure 20 depicts representative ^{31}P NMR spectra for the reaction. The changes in concentration over time for all phosphate containing species are shown in Supporting Figure 21. Supporting Table 11 show the mean concentration and standard deviation of imidazole phosphate and orthophosphate from the triplicate experiments and were used to plot Main Text Figure 2d.

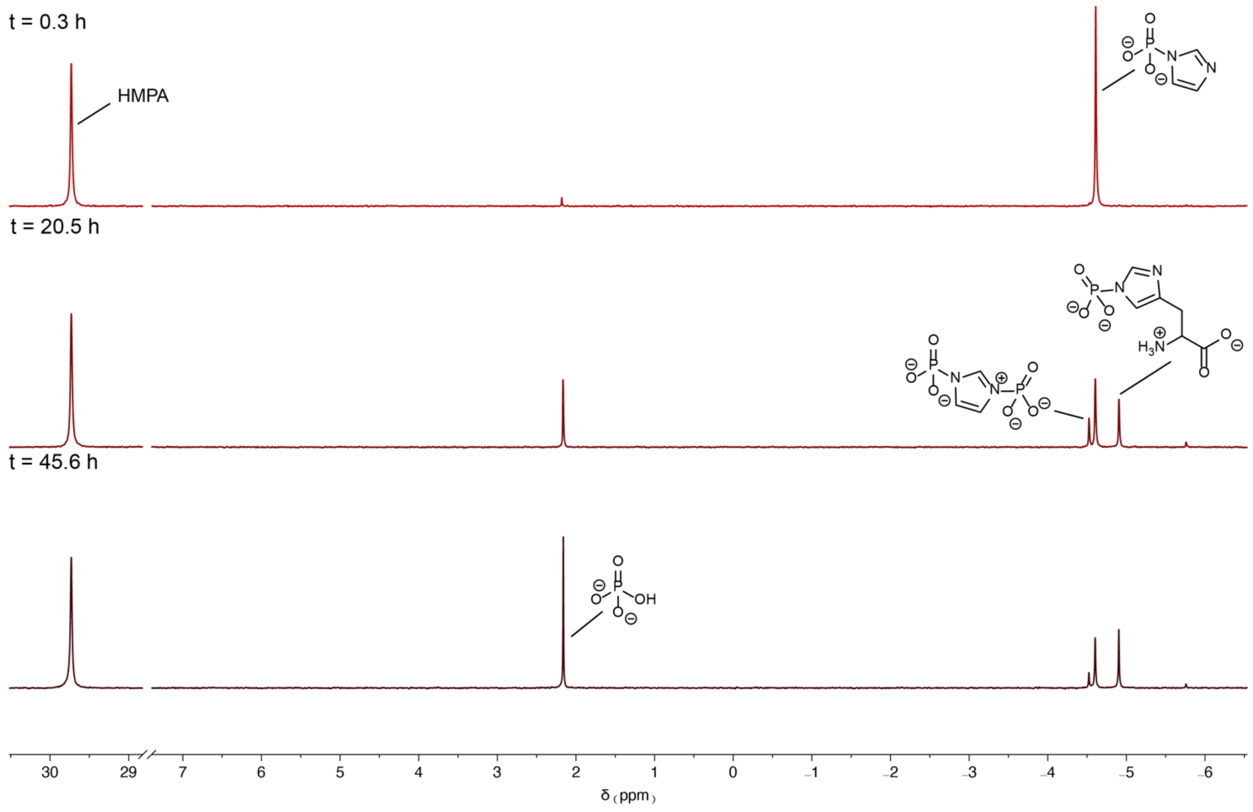

Supporting Figure 20: Representative ^{31}P-NMR spectra over time for the reaction of 50 mM calcium imidazole phosphate and 50 mM histidine solution in 0.75 M MOPS buffer at pH 7.5 in 9 : 1 H_2O : D_2O containing 0.2 M citric acid and 50 mM HMPA internal standard.

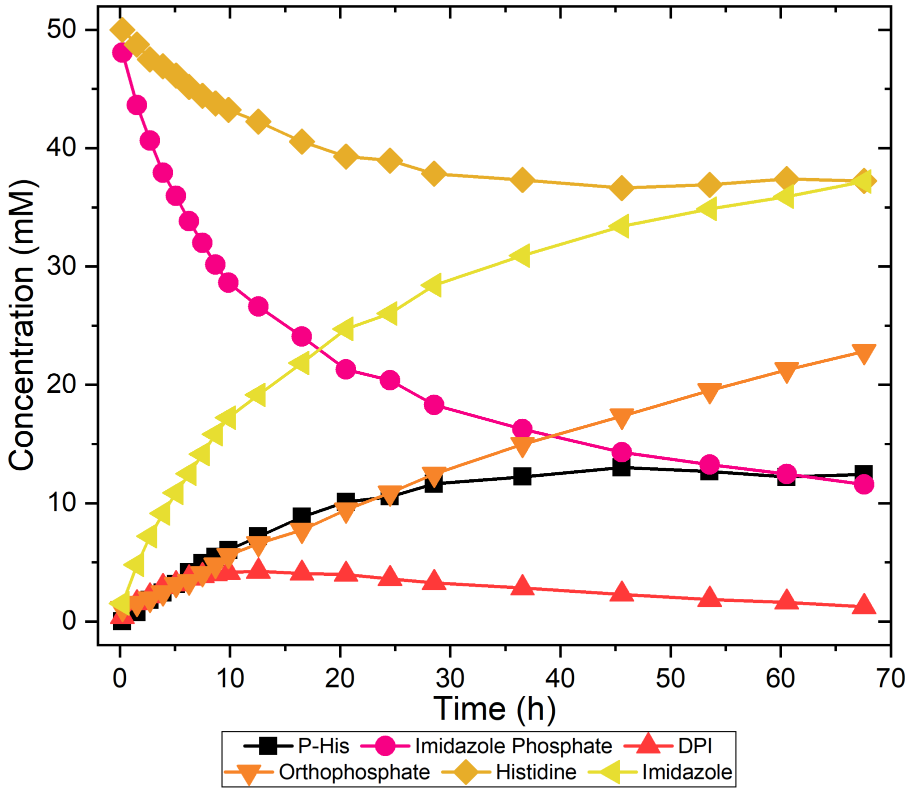

Supporting Figure 21: Representative changes in concentration over time for the reaction of 50 mM calcium imidazole phosphate and 50 mM histidine solution in 0.75 M MOPS buffer at pH 7.5 in 9 : 1 H_2O : D_2O containing 0.2 M citric acid and 50 mM HMPA internal standard. DPI = Diphosphoimidazole.

Supporting Table 11: Average changes in concentration over time from triplicate experiments for imidazole phosphate and orthophosphate in the ^{31}P-NMR spectra for 50 mM calcium imidazole phosphate and 50 mM histidine solution in 0.75 M MOPS buffer at pH 7.5 in 9 : 1 H_2O : D_2O containing 0.2 M citric acid and 50 mM HMPA internal standard. Standard deviation provided for each value was based on two repeats. These data are used to plot Main Text Figure 2d.

Time (h)	Imidazole Phosphate (mM)	Orthophosphate (mM)
0.24	47.77 ± 0.42	1.09 ± 0.04
1.06	44.46 ± 1.15	1.29 ± 0.36
2.11	40.37 ± 0.37	1.98 ± 0.13
3.03	37.48 ± 0.62	2.38 ± 0.08
4.15	35.46 ± 0.70	2.95 ± 0.27
5.29	33.30 ± 0.76	3.45 ± 0.12
6.28	32.02 ± 0.03	3.85 ± 0.28
7.23	30.13 ± 0.04	4.69 ± 0.12
8.57	28.49 ± 0.20	5.44 ± 0.19
9.33	25.82 ± 1.12	6.71 ± 0.17
13.08	23.05 ± 1.47	8.31 ± 0.84
17.08	20.93 ± 0.52	9.48 ± 0.07
21.08	19.67 ± 0.99	11.25 ± 0.55
25.07	17.92 ± 0.53	13.22 ± 1.07
41.08	15.40 ± 1.21	16.39 ± 2.02
45.08	14.01 ± 0.42	18.40 ± 1.47

S2.13 Hydrolysis of imidazole phosphate with histidine with 0.5 M MOPS

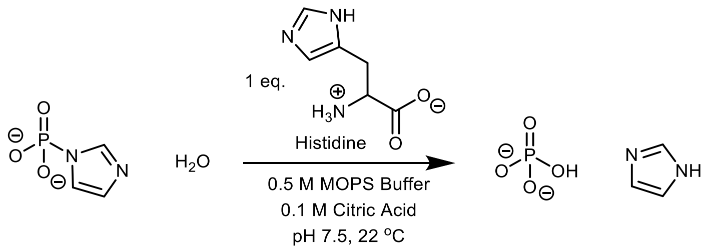

The experiment was performed as detailed in Section S2.2 but with a 0.5 M MOPS and 200 mM citric acid solution at pH 7.5.

Supporting Figure 22 depicts representative ^{31}P NMR spectra for the reaction. The changes in concentration over time for all phosphate containing species are shown in Supporting Figure 23. Supporting Table 12 show the mean concentration and standard deviation of imidazole phosphate and orthophosphate from the triplicate experiments and were used to plot Main Text Figure 2d.

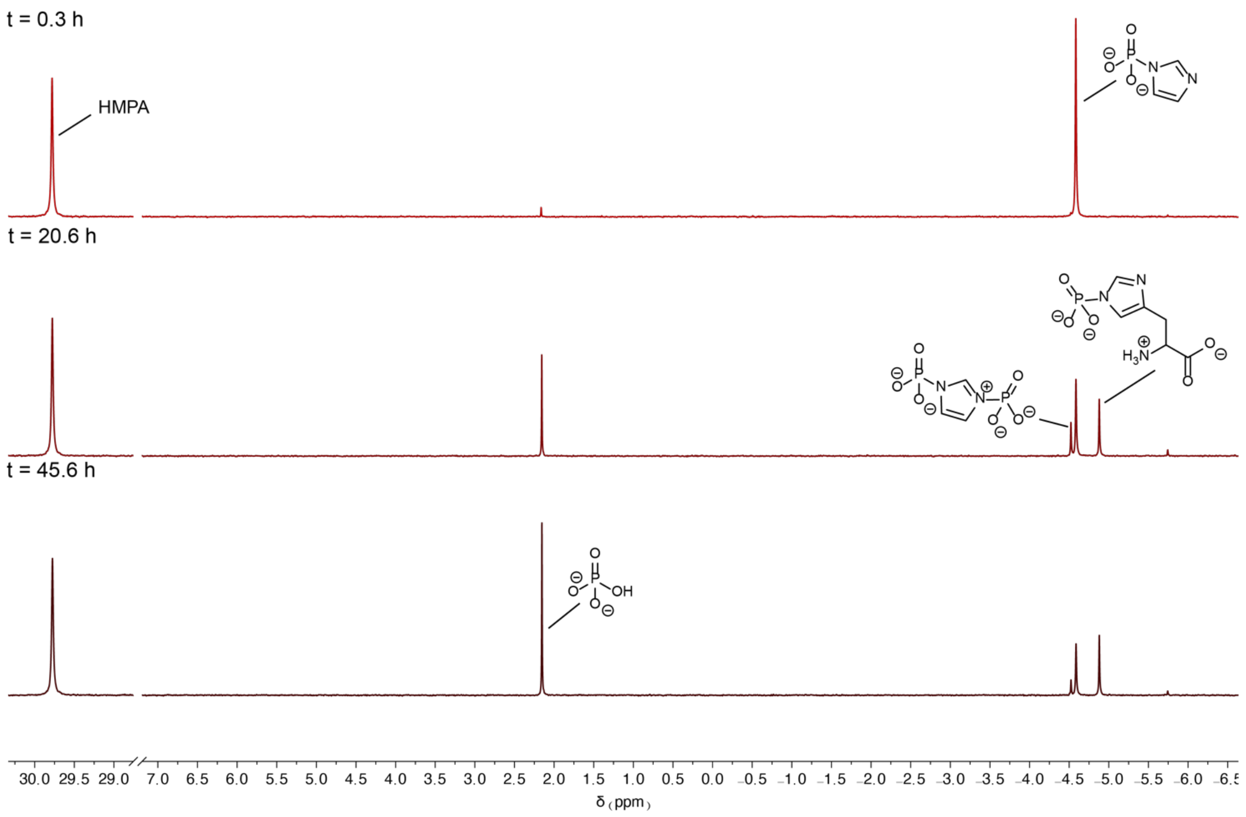

Supporting Figure 22: Representative ^{31}P-NMR spectra over time for the reaction of 50 mM calcium imidazole phosphate and 50 mM histidine solution in 0.5 M MOPS buffer at pH 7.5 in 9 : 1 H_2O : D_2O containing 0.2 M citric acid and 50 mM HMPA internal standard.

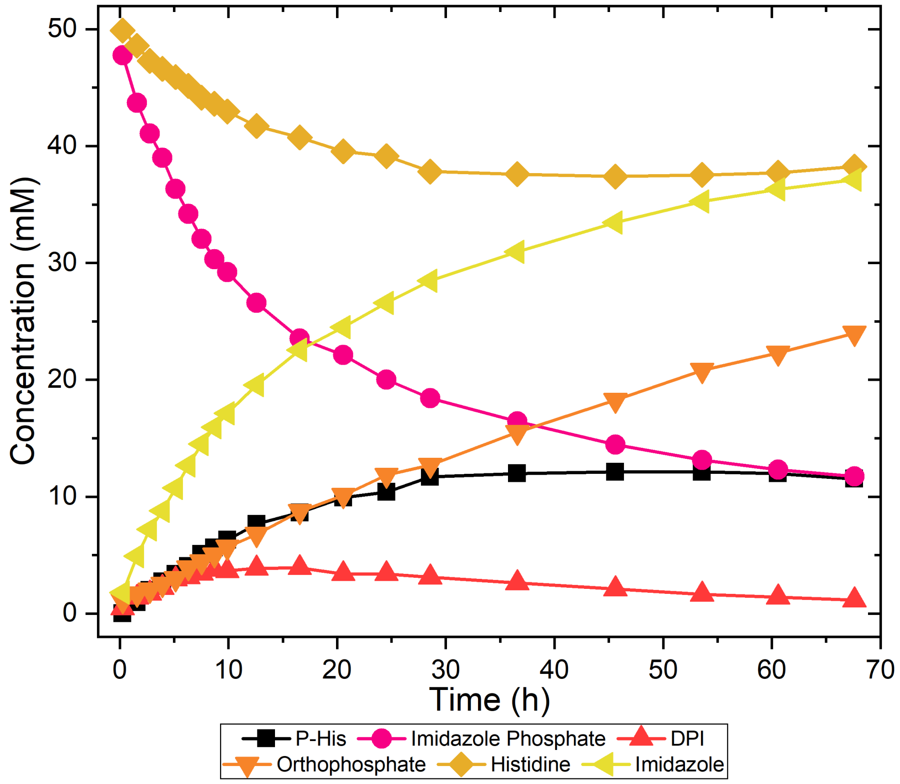

Supporting Figure 23: Representative changes in concentration over time for the reaction of 50 mM calcium imidazole phosphate and 50 mM histidine solution in 0.5 M MOPS buffer at pH 7.5 in 9 : 1 H_2O : D_2O containing 0.2 M citric acid and 50 mM HMPA internal standard. DPI = Diphosphoimidazole.

Supporting Table 12: Average changes in concentration over time from triplicate experiments for imidazole phosphate and orthophosphate in the ^{31}P-NMR spectra for 50 mM calcium imidazole phosphate and 50 mM histidine solution in 0.5 M MOPS buffer at pH 7.5 in 9 : 1 H_2O : D_2O containing 0.2 M citric acid and 50 mM HMPA internal standard. Standard deviation provided for each value was based on two repeats. These data are used to plot Main Text Figure 2d.

Time (h)	Imidazole Phosphate (mM)			Orthophosphate (mM)		
0.24	47.71	±	0.06	1.12	±	0.01
1.06	43.78	±	0.12	1.64	±	0.06
2.11	40.85	±	0.32	2.03	±	0.02
3.03	38.62	±	0.54	2.62	±	0.17
4.15	35.94	±	0.55	3.15	±	0.21
5.29	34.04	±	0.22	3.92	±	0.02
6.28	31.98	±	0.11	4.33	±	0.17
7.23	30.41	±	0.14	4.98	±	0.07
8.57	28.82	±	0.57	5.72	±	0.04
9.33	25.60	±	1.38	7.13	±	0.50
13.08	23.06	±	0.68	8.93	±	0.26
17.08	21.37	±	1.04	10.39	±	0.38
21.08	19.56	±	0.65	11.96	±	0.17
25.07	17.62	±	1.13	13.71	±	1.43
41.08	15.77	±	0.95	16.44	±	1.32
45.08	14.26	±	0.28	18.84	±	0.81

S2.14 Assessing whether buffer catalysis plays a role in the histidyl catalysed hydrolysis of imidazole phosphate.

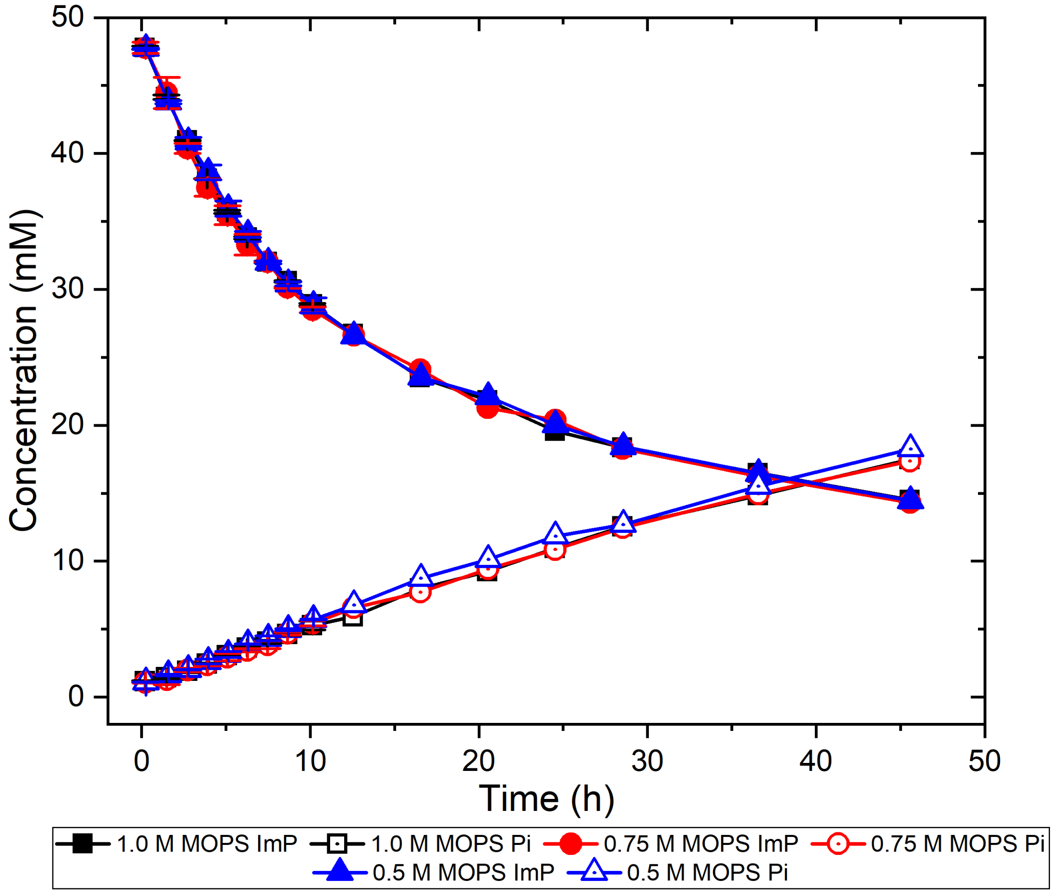

Supporting Figure 24: Comparison of the hydrolysis of imidazole phosphate at different concentrations of MOPS buffer (0.5 M, 0.75 M and 1.0 M). The changes in concentration of orthophosphate (the imidazole phosphate hydrolysis product) over time for the reaction of 50 mM calcium imidazole phosphate and 50 mM histidine solution in 0.5 M, 0.75 M and 1.0 M MOPS buffer at pH 7.5 in 9 : 1 H_2O : D_2O containing 0.1 M citric acid and 50 mM HMPA internal standard.

Supporting Figure 24 shows that the histidyl catalysed hydrolysis of imidazole phosphate is unaffected by the concentration of the MOPS buffer and thus buffer catalysis of the hydrolysis reaction does not occur.

S2.15 Uncatalysed hydrolysis of imidazole phosphate with 50 mM Imidazole

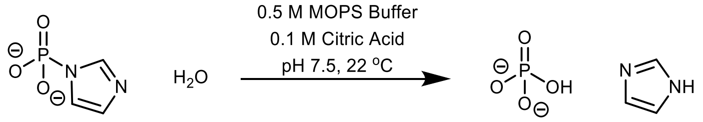

An identical procedure was used as detailed in Section S2.2 but with 50 mM imidazole added and no histidyl catalyst added.

Supporting Figure 25 depicts representative ^{31}P NMR spectra for the reaction. The changes in concentration over time for all phosphate containing species are shown in Supporting Figure 26. Supporting Table 13 show the mean concentration and standard deviation of imidazole phosphate and orthophosphate from the triplicate experiments and were used to plot Main Text Figure 2d.

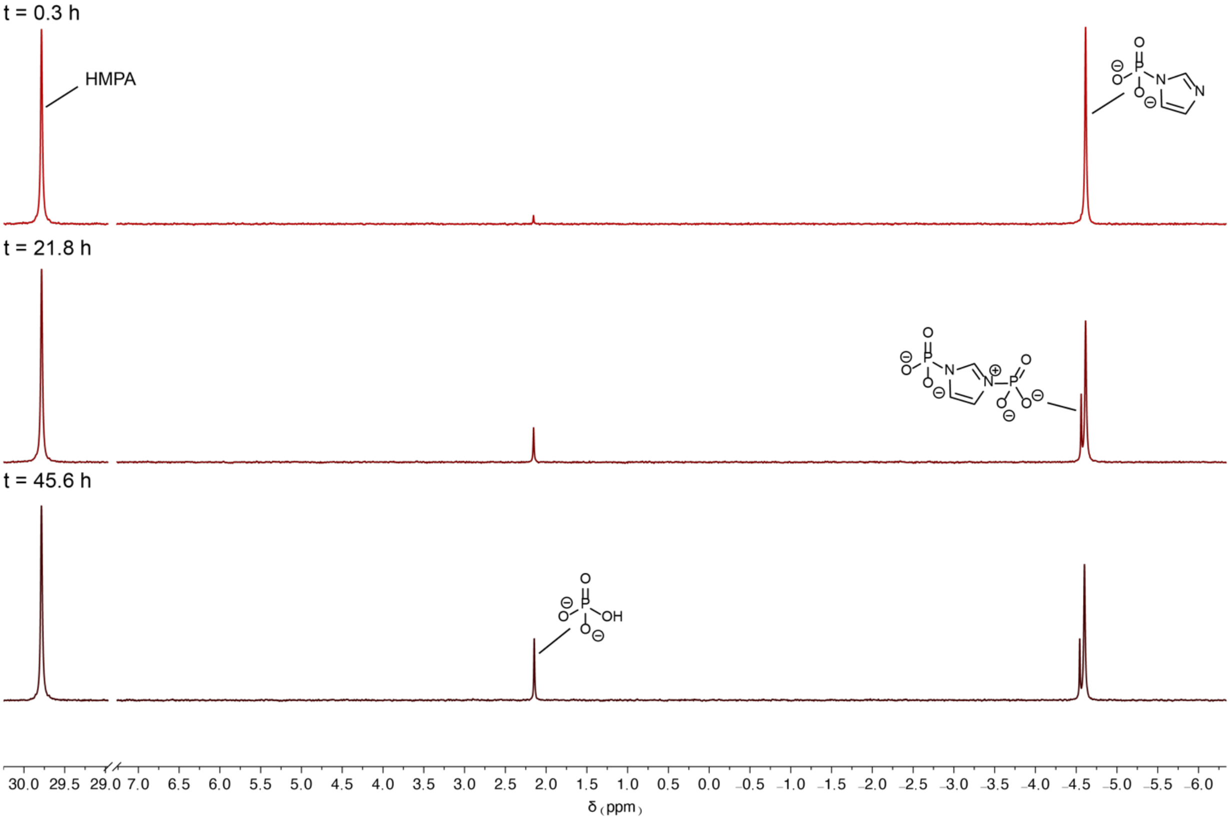

Supporting Figure 25: Representative ^{31}P-NMR spectra over time for the reaction of 50 mM calcium imidazole phosphate and 50 mM imidazole in 0.5 M MOPS buffer at pH 7.5 in 9 : 1 H_2O : D_2O containing 0.1 M citric acid and 50 mM HMPA internal standard.

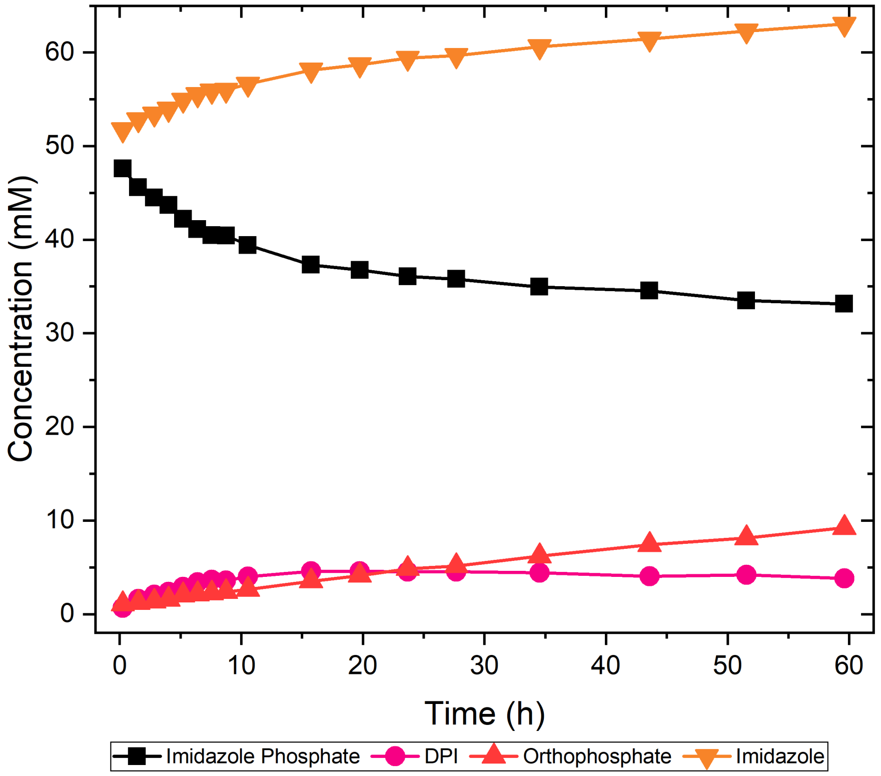

Supporting Figure 26: Representative changes in concentration over time for the reaction of 50 mM calcium imidazole phosphate and 50 mM imidazole in 0.5 M MOPS buffer at pH 7.5 in 9 : 1 H_2O : D_2O containing 0.1 M citric acid and 50 mM HMPA internal standard. DPI = Diphosphoimidazole.

Supporting Table 13: Average changes in concentration over time from triplicate experiments for imidazole phosphate and orthophosphate in the ^{31}P-NMR spectra for 50 mM calcium imidazole phosphate and 50 mM imidazole in 0.5 M MOPS buffer at pH 7.5 in 9 : 1 H_2O : D_2O containing 0.1 M citric acid and 50 mM HMPA internal standard. Standard deviation provided for each value was based on three repeats. These data are used to plot Main Text Figure 2d.

Time (h)	Imidazole Phosphate (mM)			Orthophosphate (mM)		
1.80	47.77	±	0.27	1.05	±	0.05
1.90	45.61	±	0.05	1.33	±	0.15
2.80	44.40	±	0.14	1.32	±	0.03
3.80	43.63	±	0.05	1.55	±	0.03
5.05	42.12	±	0.10	1.99	±	0.03
6.07	41.45	±	0.47	2.08	±	0.07
7.07	40.51	±	0.05	2.22	±	0.03
8.07	39.98	±	0.63	2.33	±	0.07
9.32	39.30	±	0.15	2.56	±	0.09
10.32	37.78	±	0.67	3.19	±	0.48
13.80	36.56	±	0.28	3.85	±	0.39
17.82	36.01	±	0.08	4.50	±	0.48
33.82	35.51	±	0.40	4.91	±	0.33
37.82	35.02	±	0.10	5.72	±	0.66
41.82	34.41	±	0.17	6.85	±	0.79
45.82	33.42	±	0.11	7.66	±	0.66

S2.16 Comparison of uncatalysed hydrolysis of imidazole phosphate with 50 mM Imidazole to other hydrolysis reactions

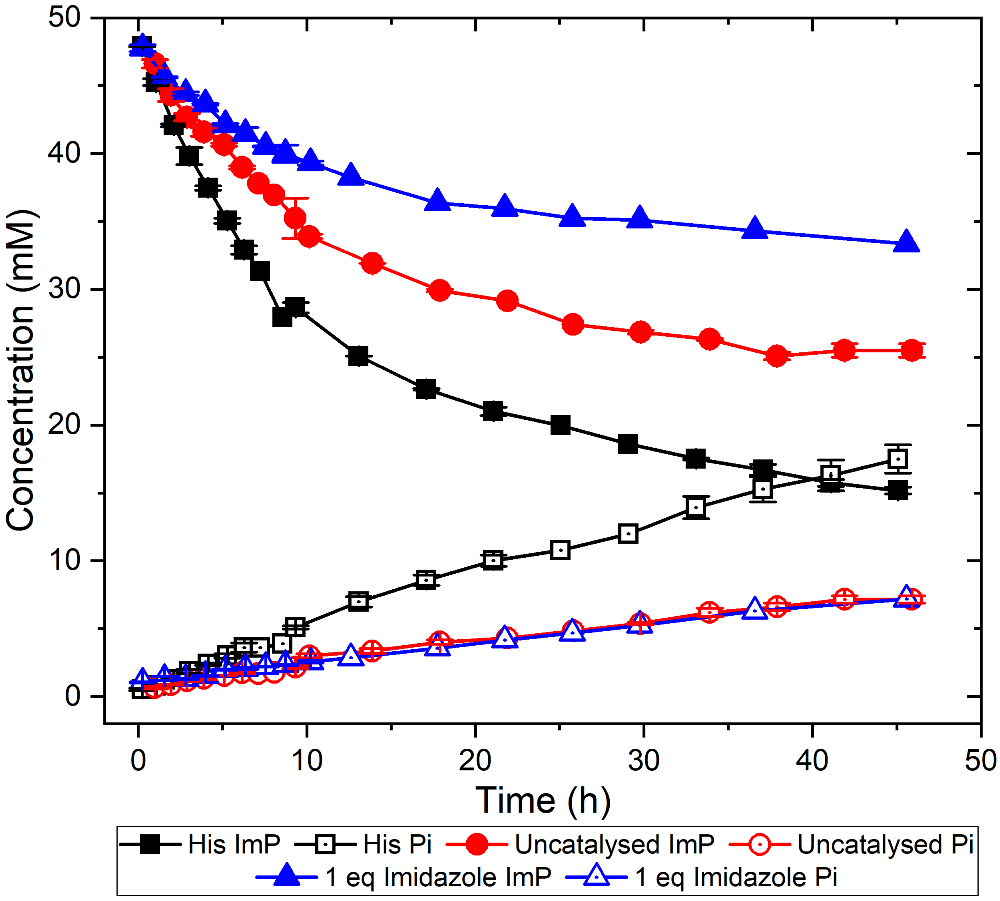

Supporting Figure 27: Comparison of the uncatalysed hydrolysis of imidazole phosphate with the hydrolysis in the presence of 50 mM histidine or 50 mM imidazole. The changes in concentration of orthophosphate (the imidazole phosphate hydrolysis product) over time for the reaction of 50 mM calcium imidazole phosphate and 50 mM histidine solution in 0.5 M MOPS buffer at pH 7.5 in 9 : 1 H_2O : D_2O containing 0.1 M citric acid and 50 mM HMPA internal standard.

Supporting Figure 27 shows a comparison between the uncatalysed hydrolysis of imidazole phosphate and in the presence of either 50 mM histidine or 50 mM imidazole. 50 mM histidine accelerates the loss of imidazole phosphate and the formation of orthophosphate the hydrolysis product compared to the uncatalysed reaction. In the presence of 50 mM imidazole the hydrolysis reaction is not accelerated as the concentration of orthophosphate formed is identical to the uncatalysed reaction. However, the formation of diphosphoimidazole is suppressed by the additional imidazole in the reaction which thus slows the loss of imidazole phosphate.

S2.17 Unchanged concentration of histidyl catalyst during hydrolysis reaction

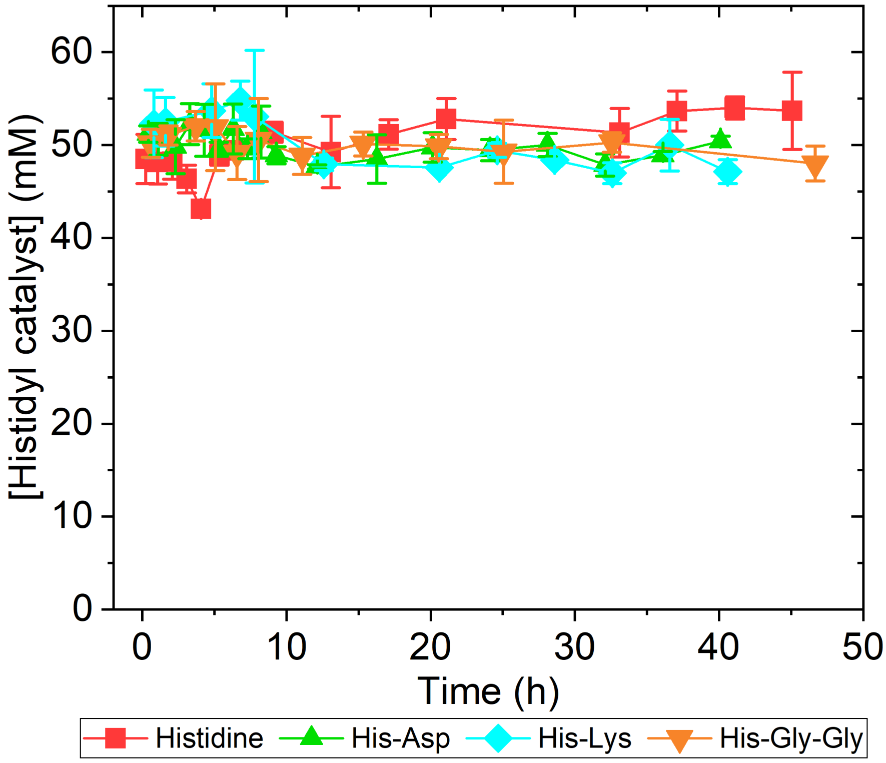

Supporting Figure 28: The concentration of histidyl catalysts throughout the course of the hydrolysis reactions measured by ^{1}Hs NMR spectroscopy with concentrations measured relative to a 3-(trimethylsilyl)propionic acid internal standard. Histidyl concentration was calculated from summation of the histidyl and the phosphorylated histidyl intermediate. The reactions contained 50 mM of the histidine/His-Asp/His-Lys/His-Gly-Gly catalyst and 50 mM calcium imidazole phosphate in 0.5 M MOPS buffer at pH 7.5 in 9 : 1 H_2O : D_2O containing 0.1 M citric acid and 50 mM HMPA internal standard.

Supporting Figure 28 shows that the histidyl catalyst was not consumed during the course of the reaction. Thus demonstrating that histidyl is a catalyst for the hydrolysis of imidazole phosphate.

S2.18 Determination of rate constants for the hydrolysis of imidazole phosphate

To determine the rate constants for the hydrolysis reaction of imidazole phosphate the averaged experimental data from S2.3 – 2.10 was used to fit the hydrolysis rate equations. Two different reaction schemes for the histidyl catalysed and uncatalysed reaction were constructed. For the uncatalysed hydrolysis reaction the reaction scheme in Supporting Figure 29 was made and from which the rate equations Eq.1 – Eq.4 were derived. For the histidyl catalysed hydrolysis reaction the reaction scheme in Supporting Figure 30 was constructed and from which the rate equations Eq.5 – Eq.10 were derived. An adapted version of the SymFit Python script was used to fit the reaction schemes to the experimental data.[2]

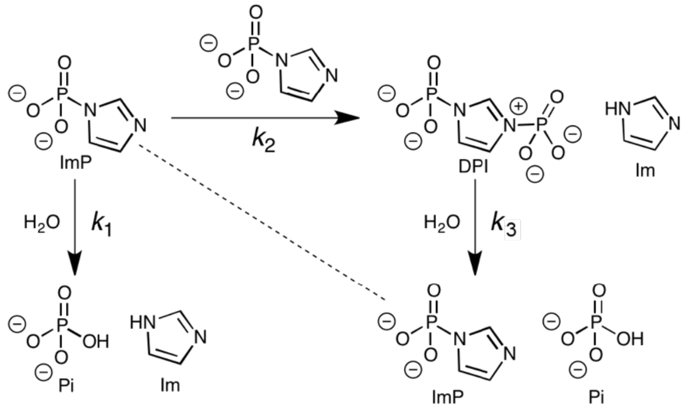

Supporting Figure 29: The reaction scheme for the uncatalysed hydrolysis of imidazole phosphate.

The rate equations for the uncatalysed hydrolysis from the reaction scheme (Supporting Figure 18):

$$\frac{\mathrm{d[ImP]}}{\mathrm{dt}} = -\,k_1[\mathrm{ImP}] - k_2[\mathrm{ImP}][\mathrm{ImP}] + k_3[\mathrm{DPI}] \quad \text{(Eq.1)}$$

$$\frac{\mathrm{d[Pi]}}{\mathrm{dt}} = k_1[\mathrm{ImP}] + k_3[\mathrm{DPI}] \quad \text{(Eq.2)}$$

$$\frac{\mathrm{d[Im]}}{\mathrm{dt}} = k_1[\mathrm{ImP}] + 0.5 * k_2[\mathrm{ImP}][\mathrm{ImP}] \quad \text{(Eq.3)}$$

$$\frac{\mathrm{d[DPI]}}{\mathrm{dt}} = 0.5 * k_2[\mathrm{ImP}][\mathrm{ImP}] - k_3[\mathrm{DPI}] \quad \text{(Eq.4)}$$

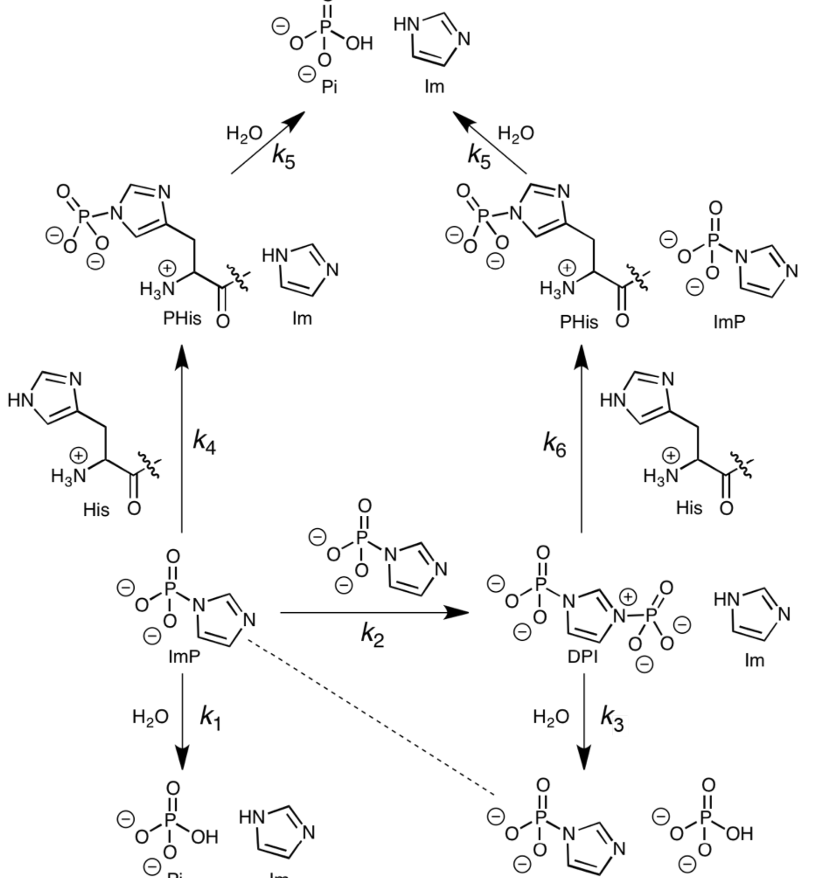

Supporting Figure 30: The reaction scheme for the histidyl-catalysed hydrolysis of imidazole phosphate.

The rate equations for the histidyl catalysed hydrolysis from the reaction scheme (Supporting Figure 19):

$$\frac{\mathrm{d[ImP]}}{\mathrm{dt}} = -\,k_1[\mathrm{ImP}] - k_2[\mathrm{ImP}][\mathrm{ImP}] + k_3[\mathrm{DPI}] - k_4[\mathrm{ImP}][\mathrm{His}] + k_6[\mathrm{DPI}][\mathrm{His}] \qquad \text{(Eq.5)}$$

$$\frac{\mathrm{d[Pi]}}{\mathrm{dt}} = k_1[\mathrm{ImP}] + k_3[\mathrm{DPI}] + k_5[\mathrm{PHis}] \qquad \text{(Eq.6)}$$

$$\frac{\mathrm{d[Im]}}{\mathrm{dt}} = k_1[\mathrm{ImP}] + 0.5 * k_2[\mathrm{ImP}][\mathrm{ImP}] + k_4[\mathrm{ImP}][\mathrm{His}] \qquad \text{(Eq.7)}$$

$$\frac{\mathrm{d[DPI]}}{\mathrm{dt}} = 0.5 * k_2[\mathrm{ImP}][\mathrm{ImP}] - k_3[\mathrm{DPI}] - k_6[\mathrm{DPI}][\mathrm{His}] \qquad \text{(Eq.8)}$$

$$\frac{\mathrm{d[His]}}{\mathrm{dt}} = -k_4[\mathrm{ImP}][\mathrm{His}] + k_5[\mathrm{PHis}] - k_6[\mathrm{DPI}][\mathrm{His}] \qquad \text{(Eq.9)}$$

$$\frac{\mathrm{d[His]}}{\mathrm{dt}} = k_4[\mathrm{ImP}][\mathrm{His}] - k_5[\mathrm{PHis}] + k_6[\mathrm{DPI}][\mathrm{His}] \qquad \text{(Eq.10)}$$

The rate constants k_1, k_2 and k_3 for the uncatalysed hydrolysis reaction were determined first by fitting Eq.1 – Eq.4 to the experimental data (Supporting Figure 31a and Supporting Table 14).

This was followed by the determination of the rate constants k_4, k_5 and k_6 for the histidyl catalysed hydrolysis reactions from fitting Eq.5 – Eq.10 to the experimental data (Supporting Figure 31b–g, Supporting Table 14). During these fittings the rate constants k_1, k_2 and k_3 were fixed at the values obtained from the uncatalysed fitting.

In the main paper $k_1 = k_{\mathrm{hyd,ImP}}$, $k_3 = k_{\mathrm{hyd,DPI}}$ and $k_5 = k_{\mathrm{hyd,PHis}}$.

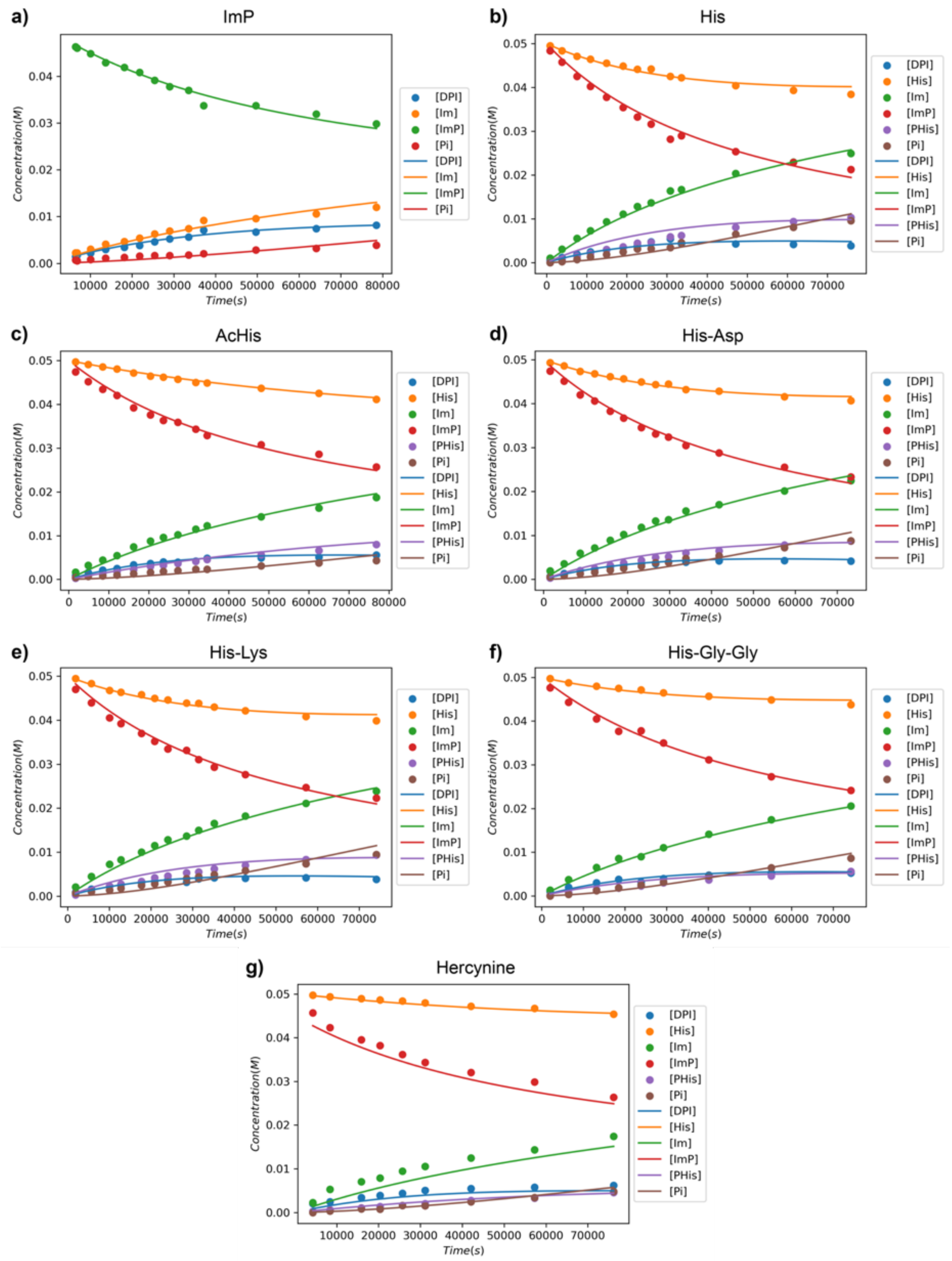

Supporting Figure 31: a) Fitting of the rate equations Eq.1–Eq.4 from the reaction scheme in Supporting Figure S29 (the lines in plots) to the experimental data (• in plots) to determine the rate constants for the uncatalysed hydrolysis of imidazole phosphate. b) – g) Fitting of the rate equations Eq.5–Eq.10 from the reaction scheme in Supporting Figure S30 (the lines in plots) to the experimental data (• in plots) to determine the rate constants for the histidyl catalysed hydrolysis of imidazole phosphate. DPI = Diphosphoimidazole, His = histidyl, Im = imidazole, ImP = imidazole phosphate, PHis = phosphorylated histidyl intermediate, Pi = orthophosphate.

Supporting Table 14: The rate constants for the uncatalysed and histidyl catalysed hydrolysis of imidazole phosphate determined from the fitting of the rate equations Eq.1 – Eq.10.

Rate constant	k_1 (s^{-1})	k_2 (M^{-1} s^{-1})	k_3 (s^{-1})	k_4 (M^{-1} s^{-1})	k_5 (s^{-1})	k_6 (M^{-1} s^{-1})	$R^{2\,a}$
Reactants	ImP + H_2O	ImP + ImP	DPI + H_2O	ImP + His	PHis + H_2O	DPI + His	-
Products	P_i + Im	DPI + Im	P_i + ImP	PHis + Im	P_i + His	PHis + ImP	-
Uncatalysed	$3.85 \times 10^{-7} \pm 5.01 \times 10^{-7}$	$2.19 \times 10^{-4} \pm 2.47 \times 10^{-5}$	$8.62 \times 10^{-6} \pm 4.00 \times 10^{-6}$	-	-	-	0.96
His	$3.85 \times 10^{-7} \pm 5.01 \times 10^{-7\,b}$	$2.19 \times 10^{-4} \pm 2.47 \times 10^{-5\,b}$	$8.62 \times 10^{-6} \pm 4.00 \times 10^{-6\,b}$	$1.67 \times 10^{-4} \pm 9.17 \times 10^{-5}$	$1.40 \times 10^{-5} \pm 1.19 \times 10^{-4}$	$6.64 \times 10^{-5} \pm 5.80 \times 10^{-3}$	0.96
AcHis	$3.85 \times 10^{-7} \pm 5.01 \times 10^{-7\,b}$	$2.19 \times 10^{-4} \pm 2.47 \times 10^{-5\,b}$	$8.62 \times 10^{-6} \pm 4.00 \times 10^{-6\,b}$	$7.06 \times 10^{-5} \pm 4.43 \times 10^{-5}$	$4.47 \times 10^{-6} \pm 4.41 \times 10^{-5}$	$1.20 \times 10^{-4} \pm 1.43 \times 10^{-3}$	0.97
His-Asp	$3.85 \times 10^{-7} \pm 5.01 \times 10^{-7\,b}$	$2.19 \times 10^{-4} \pm 2.47 \times 10^{-5\,b}$	$8.62 \times 10^{-6} \pm 4.00 \times 10^{-6\,b}$	$1.34 \times 10^{-4} \pm 6.91 \times 10^{-5}$	$1.69 \times 10^{-5} \pm 8.76 \times 10^{-5}$	$1.55 \times 10^{-4} \pm 3.73 \times 10^{-3}$	0.97
His-Lys	$3.85 \times 10^{-7} \pm 5.01 \times 10^{-7\,b}$	$2.19 \times 10^{-4} \pm 2.47 \times 10^{-5\,b}$	$8.62 \times 10^{-6} \pm 4.00 \times 10^{-6\,b}$	$1.48 \times 10^{-4} \pm 8.05 \times 10^{-5}$	$1.73 \times 10^{-5} \pm 9.73 \times 10^{-5}$	$1.48 \times 10^{-4} \pm 4.48 \times 10^{-3}$	0.96
His-Gly-Gly	$3.85 \times 10^{-7} \pm 5.01 \times 10^{-7\,b}$	$2.19 \times 10^{-4} \pm 2.47 \times 10^{-5\,b}$	$8.62 \times 10^{-6} \pm 4.00 \times 10^{-6\,b}$	$8.45 \times 10^{-5} \pm 9.00 \times 10^{-5}$	$2.14 \times 10^{-5} \pm 3.79 \times 10^{-4}$	$9.73 \times 10^{-5} \pm 7.97 \times 10^{-3}$	0.98
Hercynine	$3.85 \times 10^{-7} \pm 5.01 \times 10^{-7\,b}$	$2.19 \times 10^{-4} \pm 2.47 \times 10^{-5\,b}$	$8.62 \times 10^{-6} \pm 4.00 \times 10^{-6\,b}$	$4.37 \times 10^{-5} \pm 1.53 \times 10^{-4}$	$1.15 \times 10^{-5} \pm 3.41 \times 10^{-4}$	$1.20 \times 10^{-4} \pm 6.28 \times 10^{-3}$	0.83

[a] The R^2 value is for the overall fit of the scheme to the experimental data. [b] Value fixed during fitting.

S3 Histidyl-catalyzed phosphorylation of glycerol by imidazole phosphate (Main Text Figure 3)

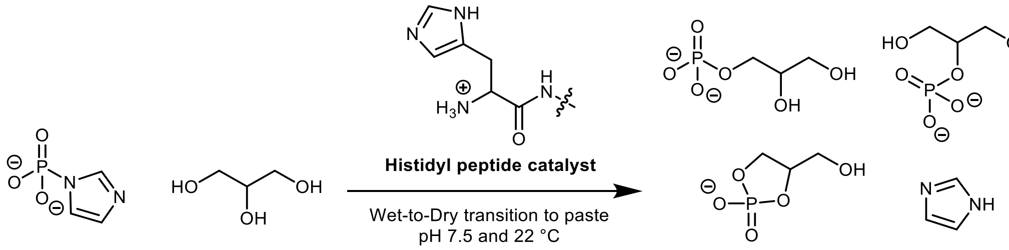

S3.1 Experimental Method

24.2 mg (0.13 mmol) of calcium imidazole phosphate and 299.3 mg (3.25 mmol, 25 eq) of glycerol were dissolved in 2.0 mL of MilliQ water to give a 65 mM calcium imidazole phosphate and a 1.63 M glycerol solution. The pH of the solution was adjusted to pH 7.5 with 5.0 M HCl and 5.0 M KOH solution. 20.2 mg (0.13 mmol, 1 eq) of histidine were dissolved in this solution to give a 65 mM histidine solution and again the pH of the solution was adjusted to pH 7.5 with 5.0 M HCl and 5.0 M KOH solution. The solution was then added to a petri dish and left with the lid off to dry at 22 °C for 2.5 days. The reaction was followed by periodically removing an approximately 10-20 mg sample from the paste and the sample as then placed into a -80 °C freezer. The samples were then analysed with ^{31}P-NMR, ^{1}H-NMR and ^{1}H ^{31}P HMBC NMR spectroscopy by dissolving them in 0.5 mL of 0.5 M citric acid buffer in 9 : 1 H_2O : D_2O at pH 6.85. NMR spectra of the samples were measured within 1.0 h of dissolution in order to prevent hydrolysis from interfering with the reliability of the results.

Beyond maintaing pH of the solutions for NMR analysis, the citric acid buffer also chelated calcium ions and therefore ensured full solubilisation of all calcium phosphate salts in the sample.

Some samples were initially stored at -20 °C rather than -80 °C whereupon some formation of the phosphorylated histidyl peptide and diphosphoimidazole occurred. Experiments were performed with histidine, His-Asp, His-Lys and the uncatalysed reaction where one sample was measured immediately on the NMR spectrometer and another sample was stored for 1 week at -20 °C. Comparisons between these two types of samples showed that only the first time point (~1 h) displayed any significant difference in the yield of phosphorylated histidine, diphosphoimidazole and imidazole phosphate (See SI Section S3.2.5, S3.3.5, S3.4.5 and S3.8.5). The yields of all other phosphate-containing compounds were unaffected, including, crucially, the yield of glycerol-phosphate. Where for the first time point phosphorylation in frozen samples occurred, it is noted in the figure caption.

S3.2 Phosphorylation of glycerol by imidazole phosphate with histidine catalyst

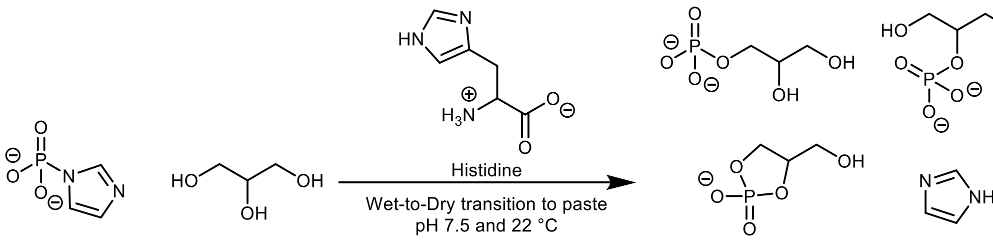

The experiment was carried out according to the procedure in S3.1. The experiment was repeated in triplicate. Supporting Figures 32, 34 and 36 depict representative ^{31}P NMR spectra for the reaction over time. The changes in yield over time for all phosphate containing species are shown in Supporting Figures 33, 35 and 37. The mean experimental results with the standard deviation of each experimental data point from the triplicate experiments are shown in Supporting Figure 38.

S3.2.1 Experiment 1 - 3.25 mmol glycerol + 0.13 mmol imidazole phosphate + 0.13 mmol histidine

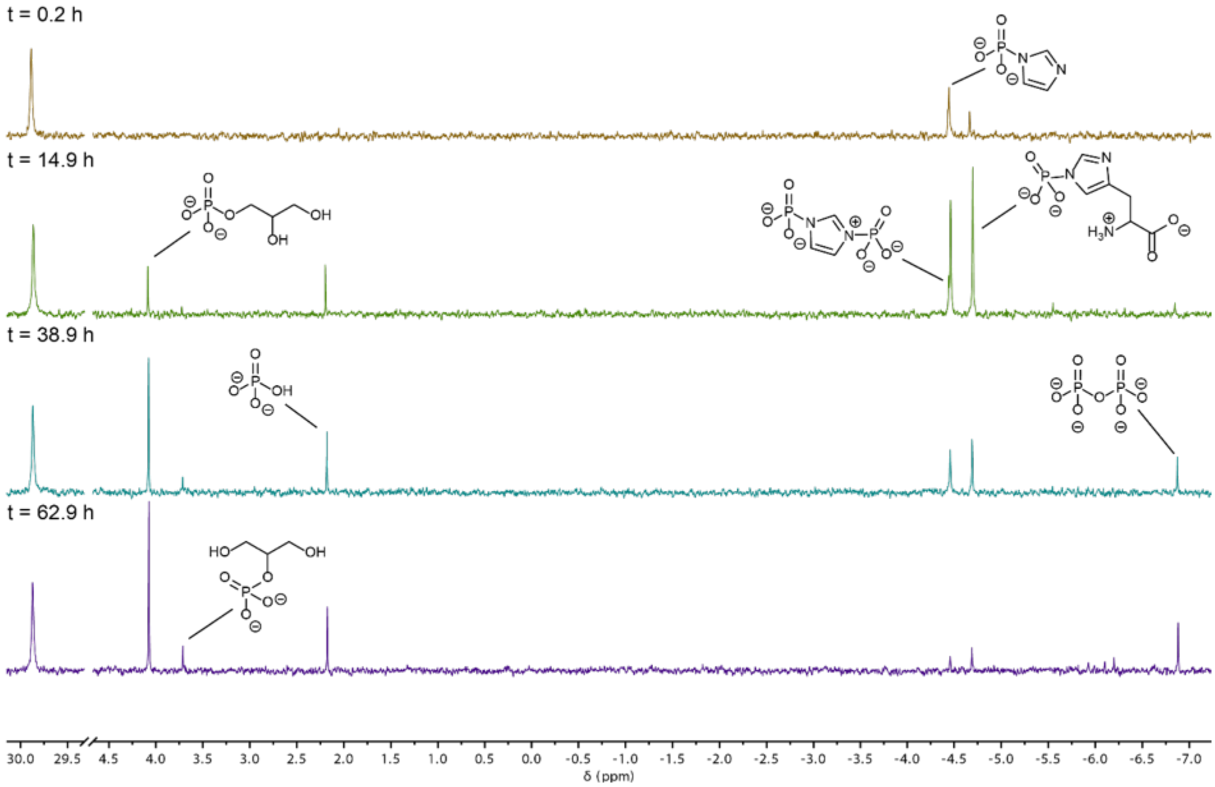

Supporting Figure 32: Representative ^{31}P-NMR spectra over time for the reaction of 0.13 mmol of calcium imidazole phosphate, 3.25 mmol of glycerol and 0.13 mmol of histidine at pH 7.5 and 22 °C.

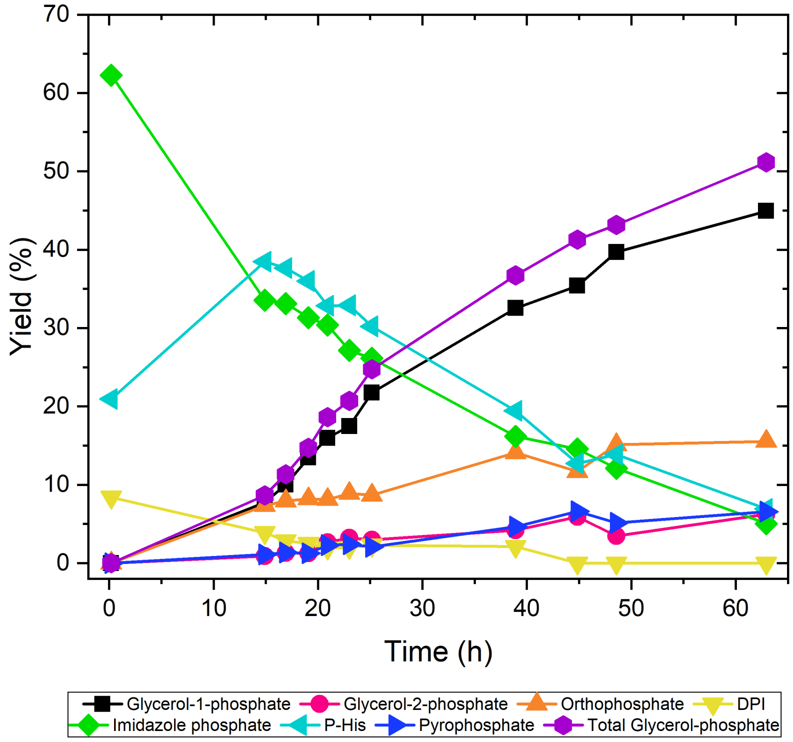

Supporting Figure 33: Changes in yield over time for the reaction of 0.13 mmol of calcium imidazole phosphate, 3.25 mmol of glycerol and 0.13 mmol of histidine at pH 7.5 and 22 °C. The first time point includes phosphorylation that took place in the freezer at -20 °C and thus the yields of imidazole phosphate, diphosphoimidazole and P-His are for this time point off.

S3.2.2 Experiment 2 - 3.25 mmol glycerol + 0.13 mmol imidazole phosphate + 0.13 mmol histidine

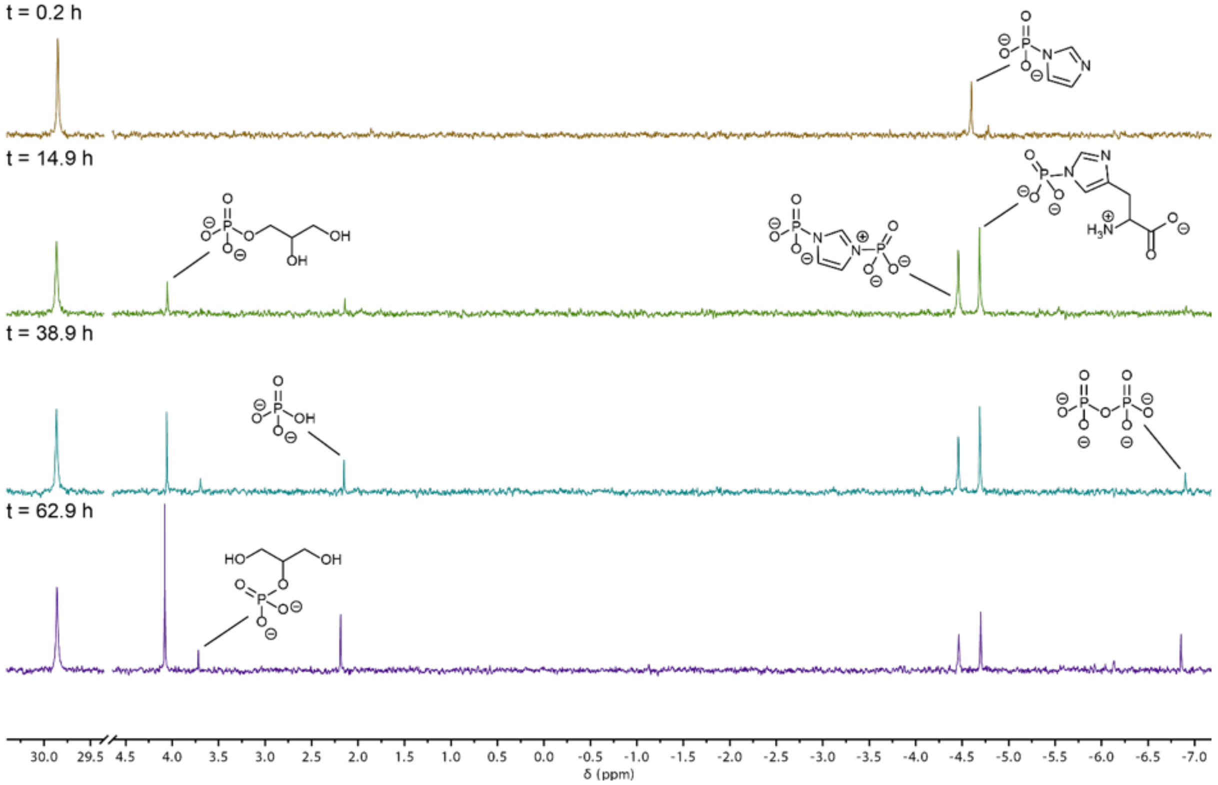

Supporting Figure 34: Representative ^{31}P-NMR spectra over time for the reaction of 0.13 mmol of calcium imidazole phosphate, 3.25 mmol of glycerol and 0.13 mmol of histidine at pH 7.5 and 22 °C.

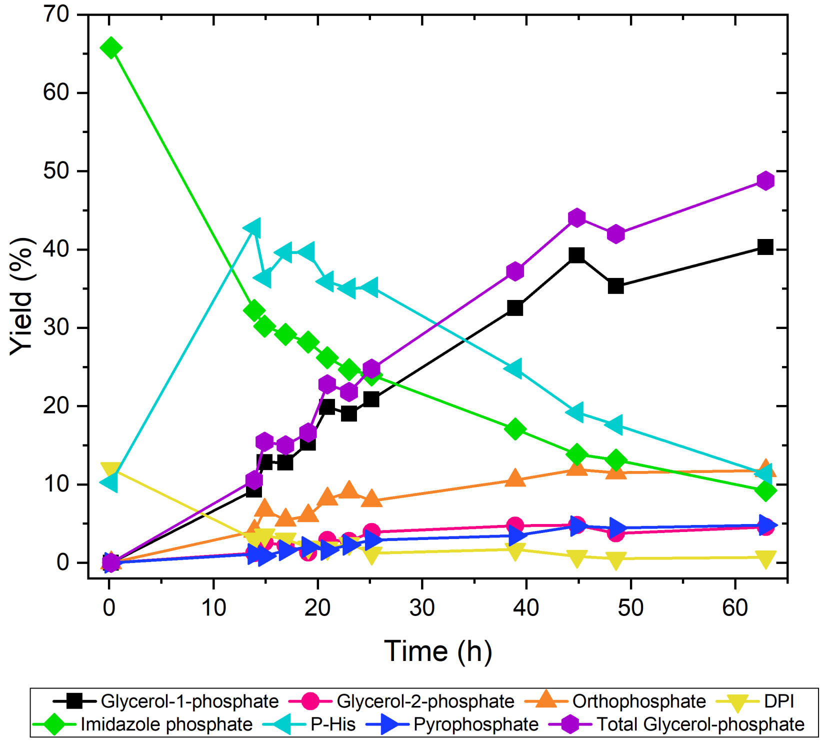

Supporting Figure 35: Changes in yield over time for the reaction of 0.13 mmol of calcium imidazole phosphate, 3.25 mmol of glycerol and 0.13 mmol of histidine at pH 7.5 and 22 °C. The first time point includes phosphorylation that took place in the freezer at -20 °C and thus the yields of imidazole phosphate, diphosphoimidazole and P-His are for this time point off.

S3.2.3 Experiment 3 - 3.25 mmol glycerol + 0.13 mmol imidazole phosphate + 0.13 mmol histidine

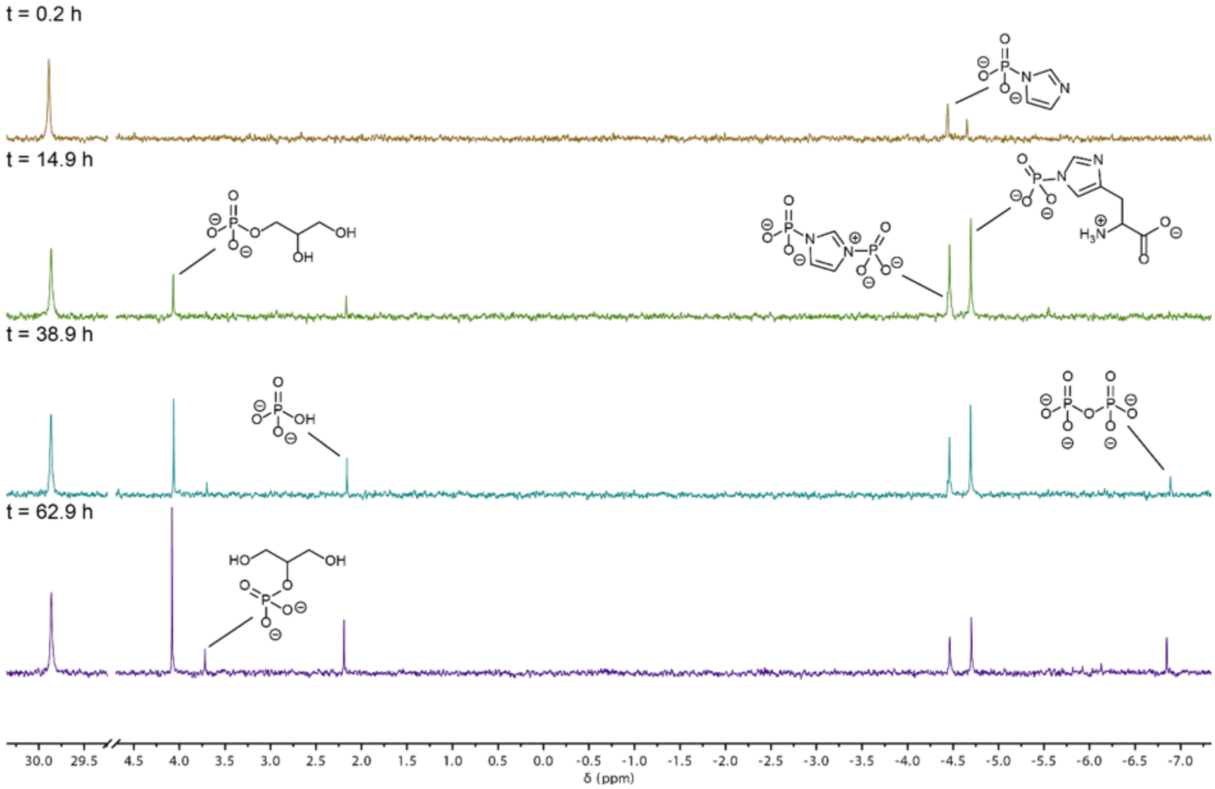

Supporting Figure 36: Representative ^{31}P-NMR spectra over time for the reaction of 0.13 mmol of calcium imidazole phosphate, 3.25 mmol of glycerol and 0.13 mmol of histidine at pH 7.5 and 22 °C.

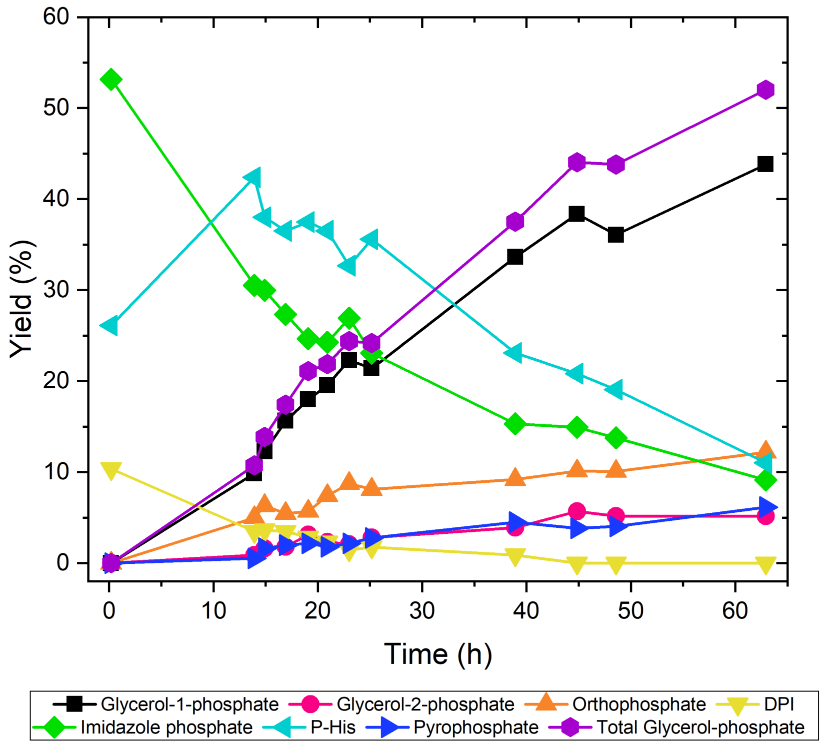

Supporting Figure 37: Changes in yield over time for the reaction of 0.13 mmol of calcium imidazole phosphate, 3.25 mmol of glycerol and 0.13 mmol of histidine at pH 7.5 and 22 °C. The first time point includes phosphorylation that took place in the freezer at -20 °C and thus the yields of imidazole phosphate, diphosphoimidazole and P-His are for this time point off.

S3.2.4 Combined results for 3.25 mmol glycerol + 0.13 mmol imidazole phosphate + 0.13 mmol histidine

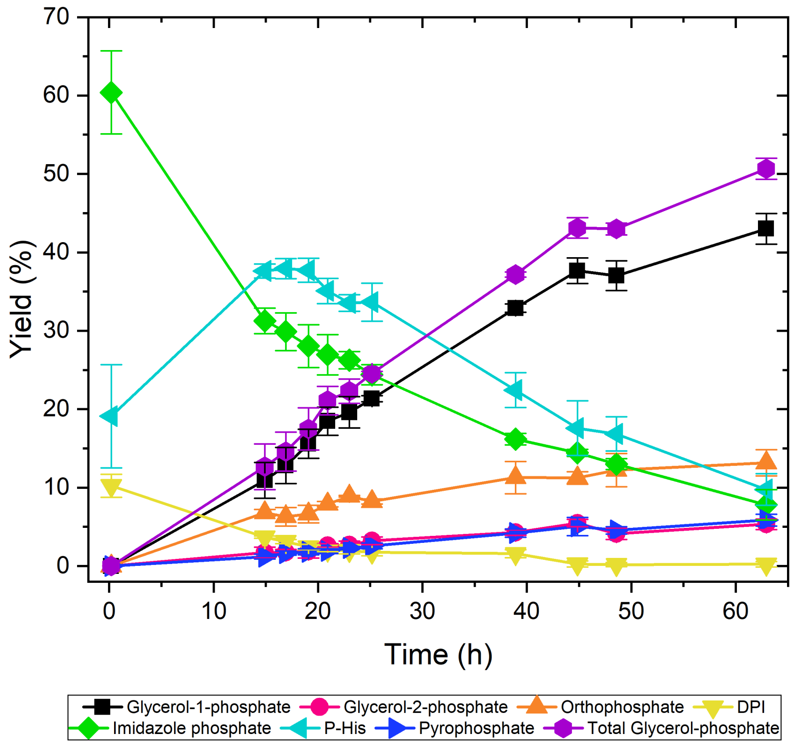

Supporting Figure 38: Changes in yield over time for the reaction of 0.13 mmol of calcium imidazole phosphate, 3.25 mmol of glycerol and 0.13 mmol of histidine at pH 7.5 and 22 °C. These data are the mean values and standard deviation based upon triplicate experiments. The first time point includes phosphorylation that took place in the freezer at -20 °C and thus the yields of imidazole phosphate, diphosphoimidazole and P-His are for this time point off.

S3.2.5 Comparison between samples measured immediately and samples stored at -20 °C for 1 week for 3.25 mmol glycerol + 0.13 mmol imidazole phosphate + 0.13 mmol histidine

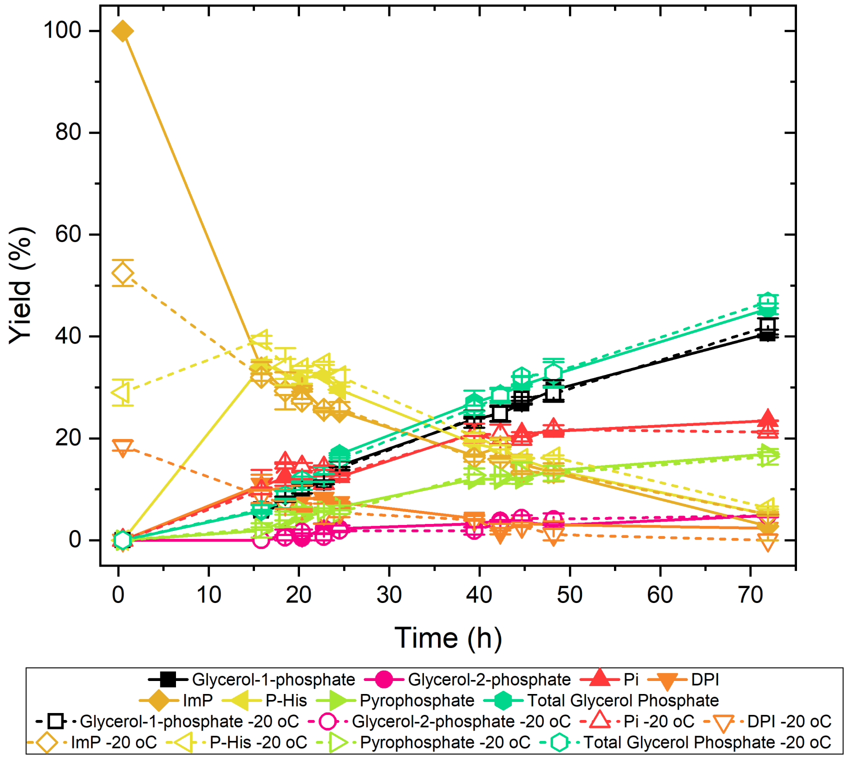

Supporting Figure 39: The changes in yield over time for the reaction of 0.13 mmol of calcium imidazole phosphate, 3.25 mmol of glycerol and 0.13 mmol of histidine at pH 7.5 and 22 °C. Solid lines are for the samples measured immediately and dashed lines are for samples measured after being stored for 1 week at -20 °C. These data are the mean values and standard deviation based upon duplicate experiments.

S3.3 Phosphorylation of glycerol by imidazole phosphate with His-Asp catalyst

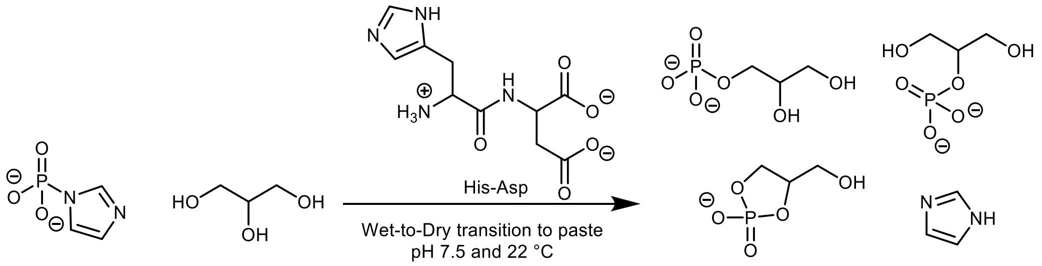

The experiment was carried out according to the procedure in S3.1 but with the 65 mM of histidine replaced by 65 mM His-Asp (35.1 mg, 0.13 mmol). The experiment was repeated in triplicate. Supporting Figures 40, 42 and 44 depict representative ^{31}P NMR spectra for the reaction over time. The changes in yield over time for all phosphate containing species are shown in Supporting Figures 41, 43 and 45. The mean experimental results with the standard deviation of each experimental data point from the triplicate experiments are shown in Supporting Figure 46.

S3.3.1 Experiment 1 - 3.25 mmol glycerol + 0.13 mmol imidazole phosphate + 0.13 mmol His-Asp

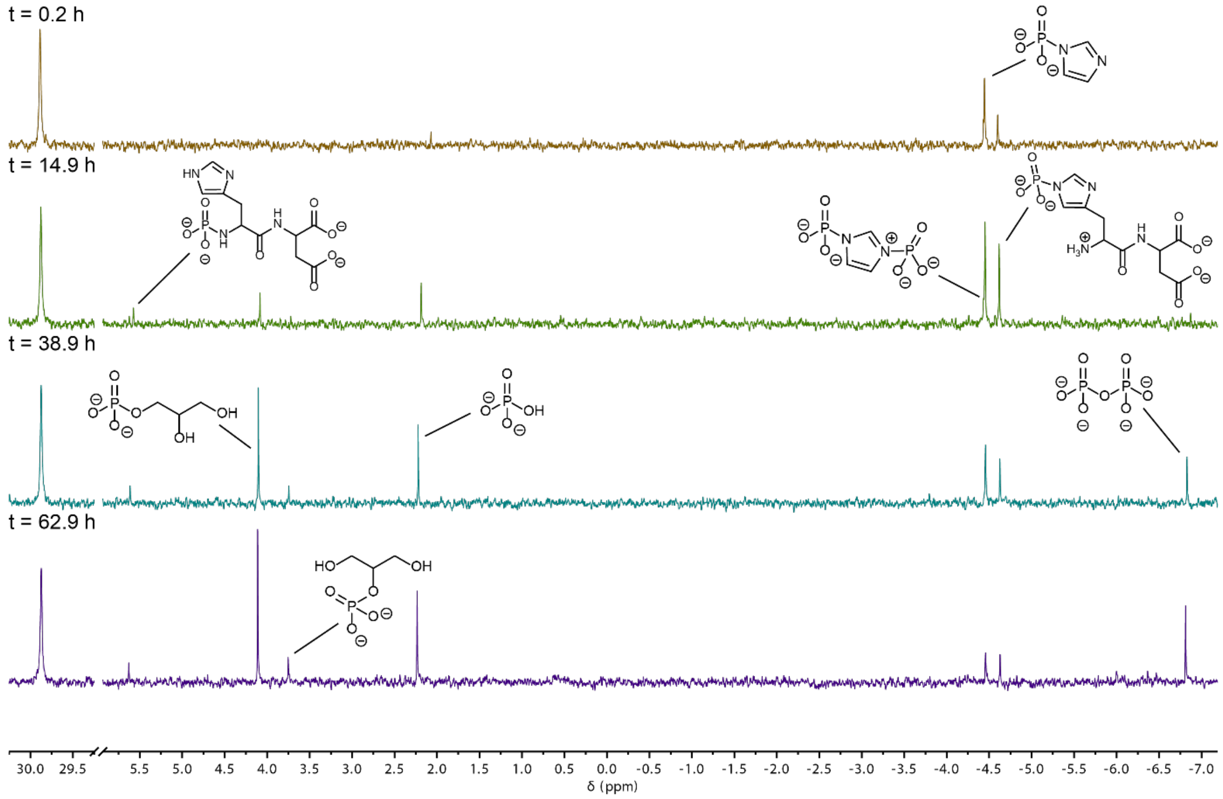

Supporting Figure 40: Representative ^{31}P-NMR spectra over time for the reaction of 0.13 mmol of calcium imidazole phosphate, 3.25 mmol of glycerol and 0.13 mmol of His-Asp at pH 7.5 and 22 °C.

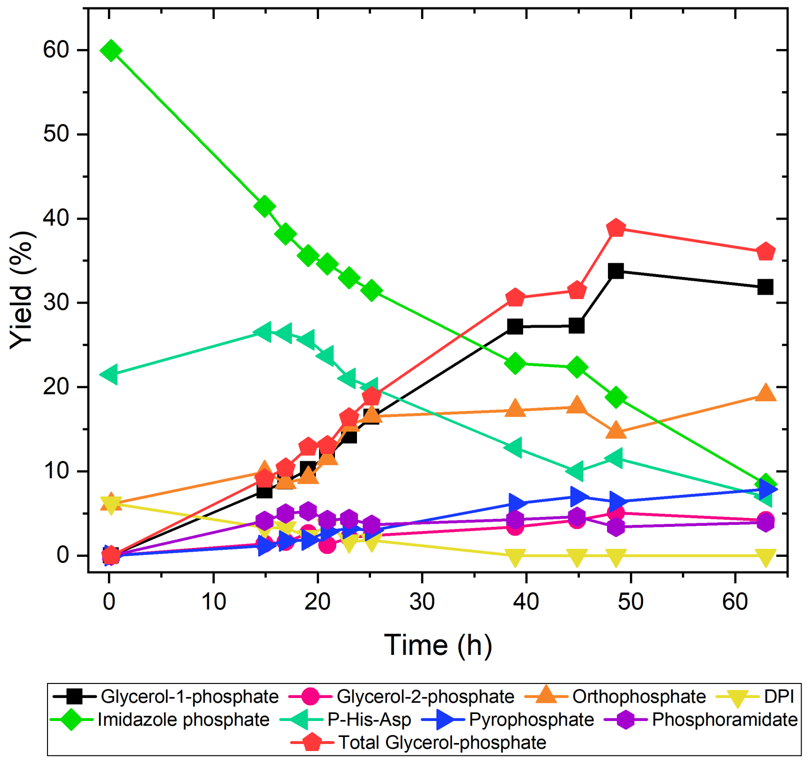

Supporting Figure 41: Changes in yield over time for the reaction of 0.13 mmol of calcium imidazole phosphate, 3.25 mmol of glycerol and 0.13 mmol of His-Asp at pH 7.5 and 22 °C. DPI = Diphosphoimidazole. The first time point includes phosphorylation that took place in the freezer at -20 °C and thus the yields of imidazole phosphate, diphosphoimidazole and P-His-Asp are for this time point off.

S3.3.2 Experiment 2 - 3.25 mmol glycerol + 0.13 mmol imidazole phosphate + 0.13 mmol His-Asp

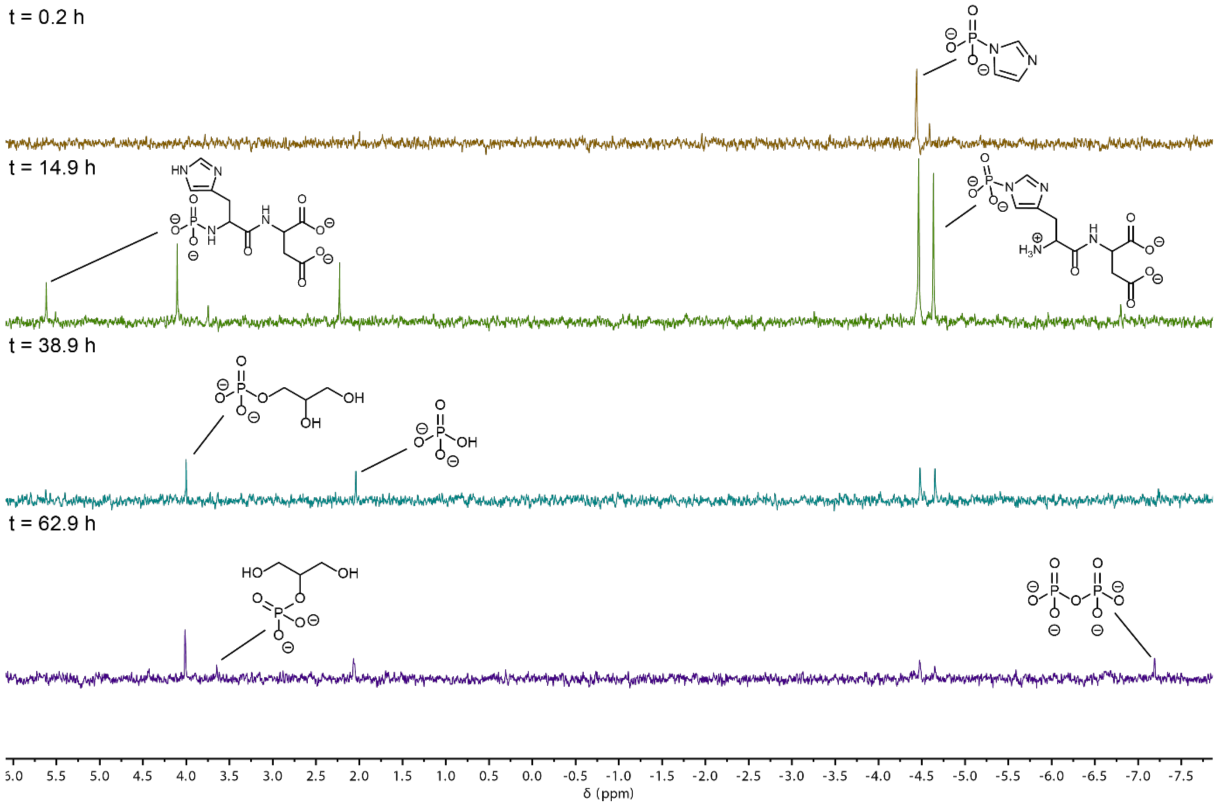

Supporting Figure 42: Representative ^{31}P-NMR spectra over time for the reaction of 0.13 mmol of calcium imidazole phosphate, 3.25 mmol of glycerol and 0.13 mmol of His-Asp at pH 7.5 and 22 °C.

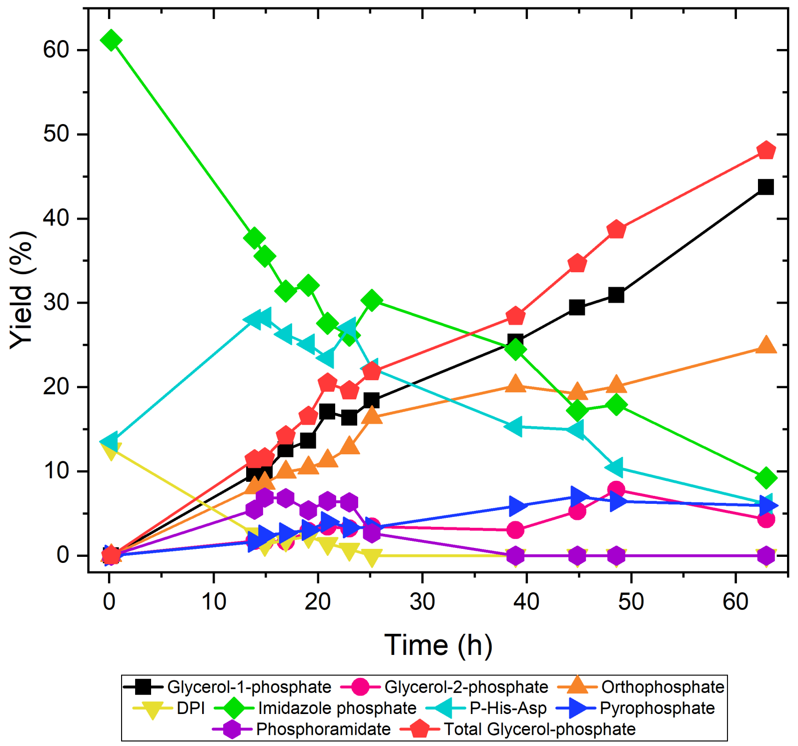

Supporting Figure 43: Changes in yield over time for the reaction of 0.13 mmol of calcium imidazole phosphate, 3.25 mmol of glycerol and 0.13 mmol of His-Asp at pH 7.5 and 22 °C. DPI = Diphosphoimidazole. The first time point includes phosphorylation that took place in the freezer at -20 °C and thus the yields of imidazole phosphate, diphosphoimidazole and P-His-Asp are for this time point off.

S3.3.3 Experiment 3 - 3.25 mmol glycerol + 0.13 mmol imidazole phosphate + 0.13 mmol His-Asp

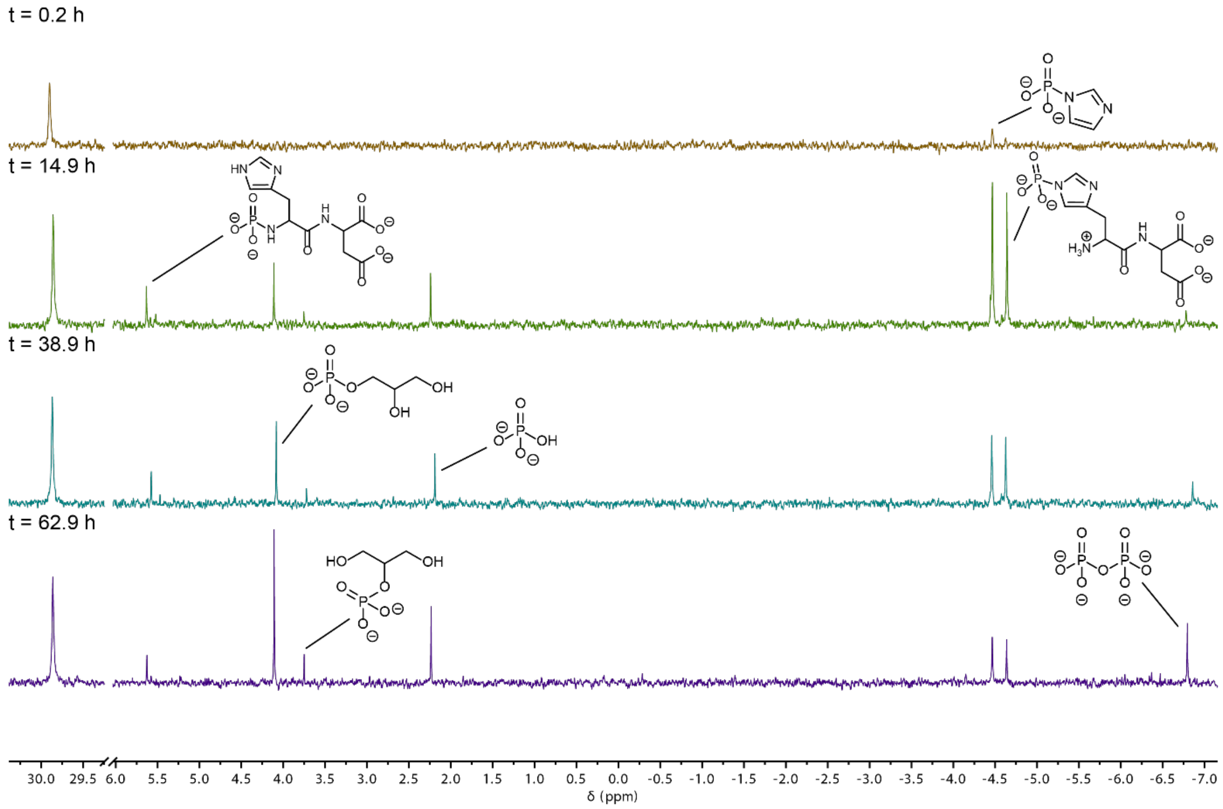

Supporting Figure 44: Representative ^{31}P-NMR spectra over time for the reaction of 0.13 mmol of calcium imidazole phosphate, 3.25 mmol of glycerol and 0.13 mmol of His-Asp at pH 7.5 and 22 °C.

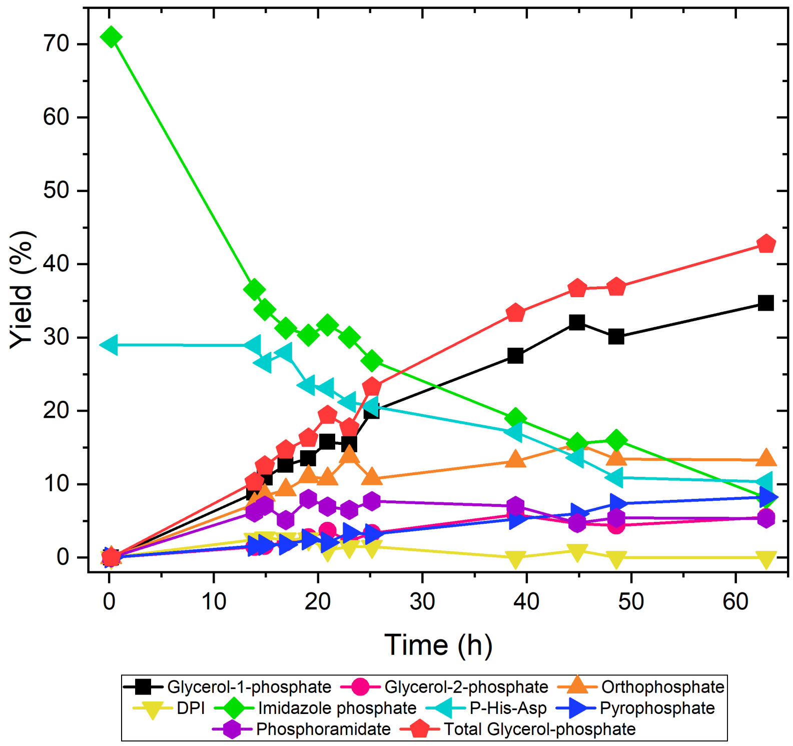

Supporting Figure 45: Changes in yield over time for the reaction of 0.13 mmol of calcium imidazole phosphate, 3.25 mmol of glycerol and 0.13 mmol of His-Asp at pH 7.5 and 22 °C. DPI = Diphosphoimidazole. The first time point includes phosphorylation that took place in the freezer at -20 °C and thus the yields of imidazole phosphate, diphosphoimidazole and P-His-Asp are for this time point off.

S3.3.4 Combined results for 3.25 mmol glycerol + 0.13 mmol imidazole phosphate + 0.13 mmol His-Asp

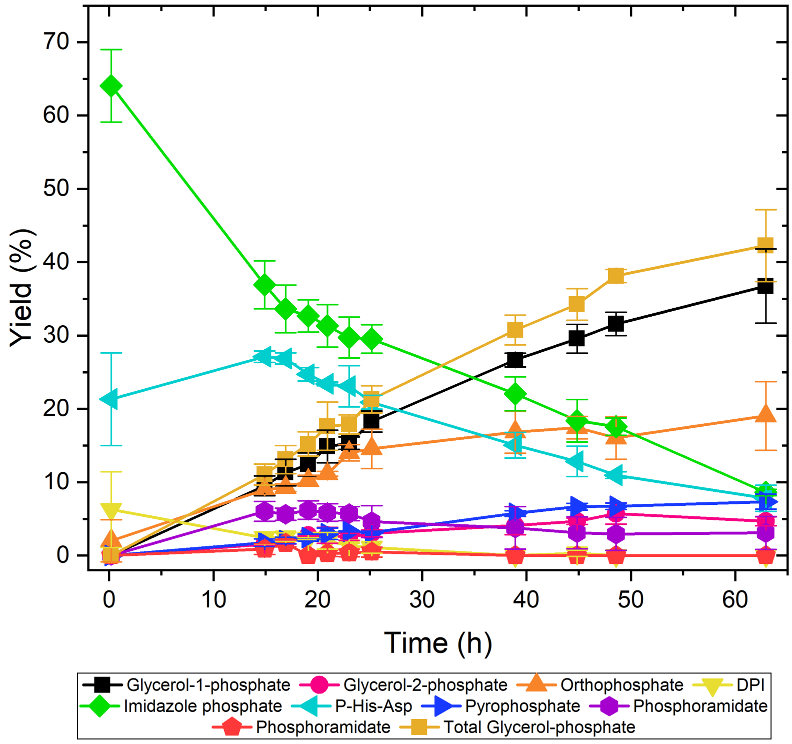

Supporting Figure 46: Changes in yield over time for the reaction of 0.13 mmol of calcium imidazole phosphate, 3.25 mmol of glycerol and 0.13 mmol of His-Asp at pH 7.5 and 22 °C. DPI = Diphosphoimidazole. These data are the mean values and standard deviation based upon triplicate experiments. The first time point includes phosphorylation that took place in the freezer at -20 °C and thus the yields of imidazole phosphate, diphosphoimidazole and P-His-Asp are for this time point off.

S3.3.5 Comparison between samples measured immediately and samples stored at -20 °C for 1 week for 3.25 mmol glycerol + 0.13 mmol imidazole phosphate + 0.13 mmol His-Asp

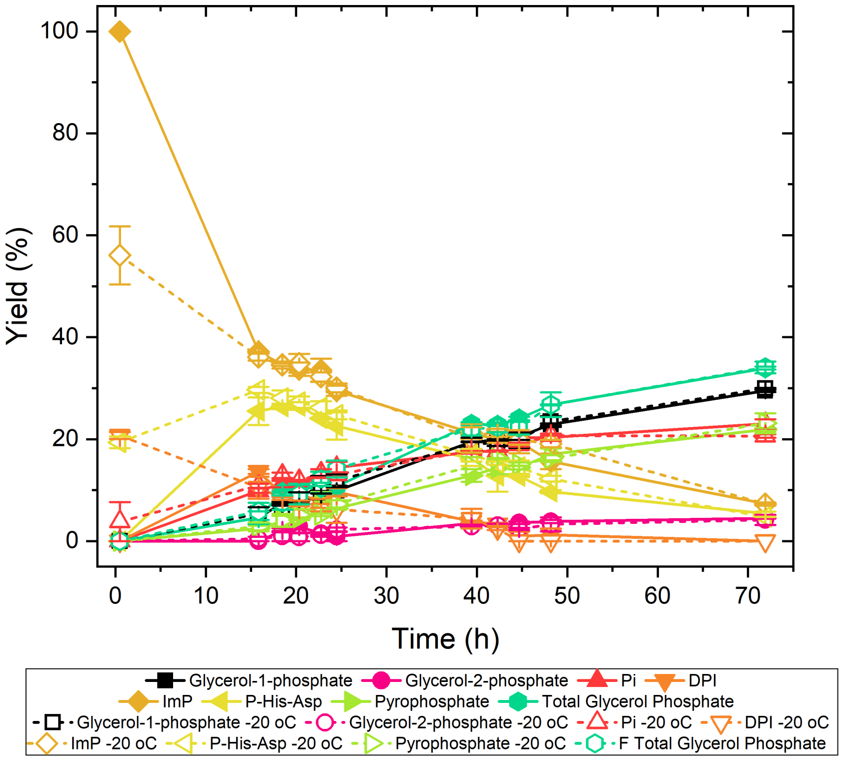

Supporting Figure 47: The changes in yield over time for the reaction of 0.13 mmol of calcium imidazole phosphate, 3.25 mmol of glycerol and 0.13 mmol of His-Asp at pH 7.5 and 22 °C. Solid lines are for the samples measured immediately and dashed lines are for samples measured after being stored for 1 week at -20 °C. These data are the mean values and standard deviation based upon duplicate experiments.

S3.4 Phosphorylation of glycerol by imidazole phosphate with His-Lys catalyst

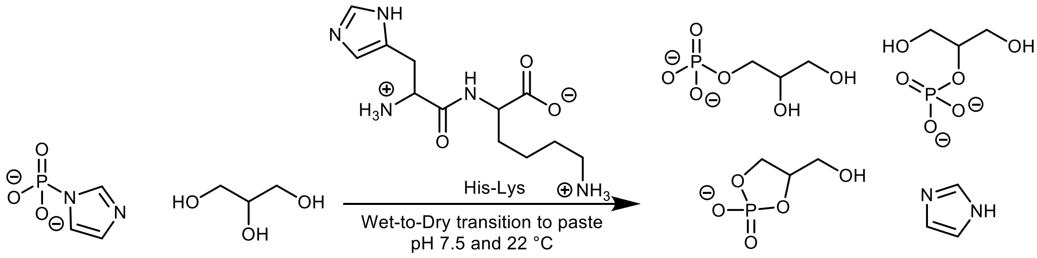

The experiment was carried out according to the procedure in S3.1 but with the 65 mM of histidine replaced by 65 mM His-Lys.HBr (47.3 mg, 0.13 mmol). The experiment was repeated in triplicate. Supporting Figures 48, 50 and 52 depict representative ^{31}P NMR spectra for the reaction over time. The changes in yield over time for all phosphate containing species are shown in Supporting Figures 49, 51 and 53. The mean experimental results with the standard deviation of each experimental data point from the triplicate experiments are shown in Supporting Figure 54.

S3.4.1 Experiment 1 - 3.25 mmol glycerol + 0.13 mmol imidazole phosphate + 0.13 mmol His-Lys

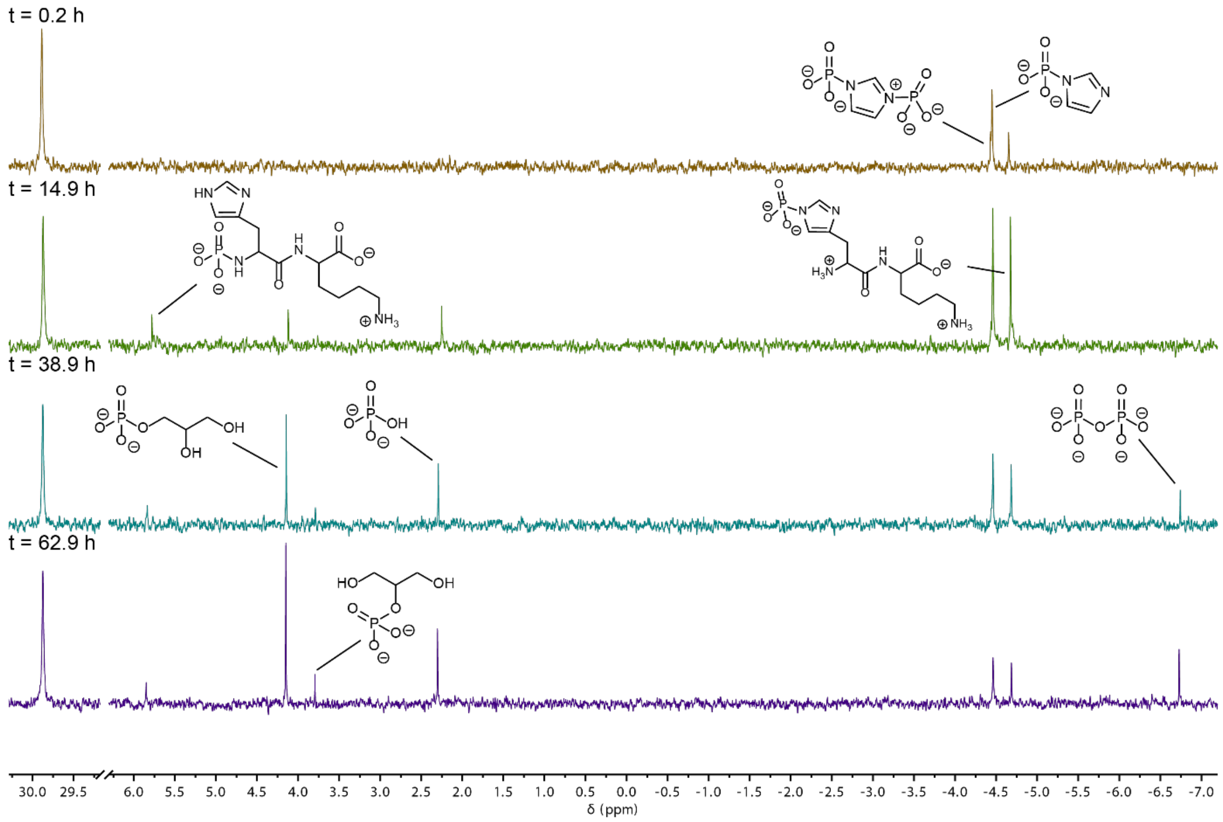

Supporting Figure 48: Representative ^{31}P-NMR spectra over time for the reaction of 0.13 mmol of calcium imidazole phosphate, 3.25 mmol of glycerol and 0.13 mmol of His-Lys at pH 7.5 and 22 °C.

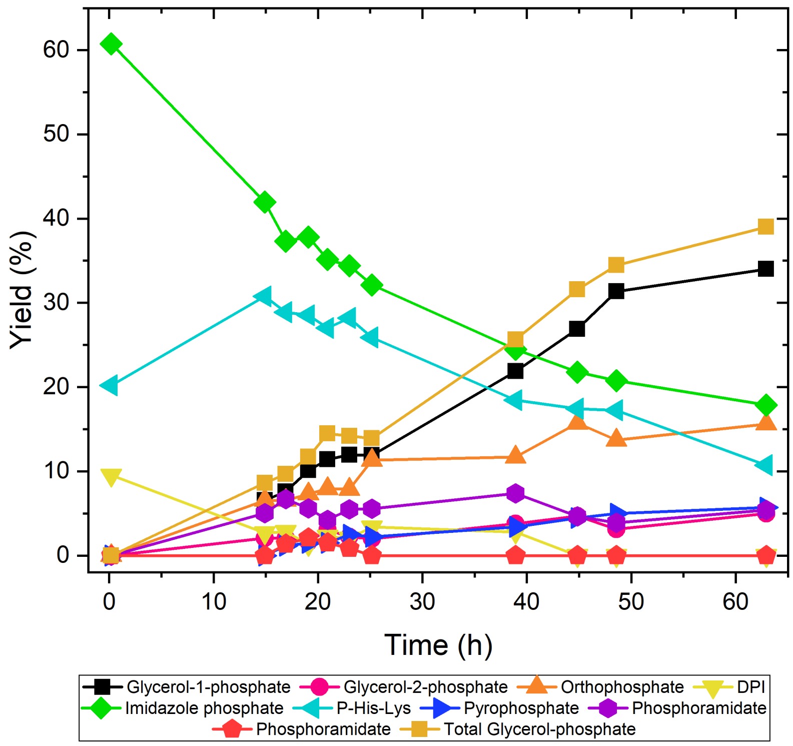

Supporting Figure 49: Changes in yield over time for the reaction of 0.13 mmol of calcium imidazole phosphate, 3.25 mmol of glycerol and 0.13 mmol of His-Lys at pH 7.5 and 22 °C. DPI = Diphosphoimidazole. The first time point includes phosphorylation that took place in the freezer at -20 °C and thus the yields of imidazole phosphate, diphosphoimidazole and P-His-Lys are for this time point off.

S3.4.2 Experiment 2 - 3.25 mmol glycerol + 0.13 mmol imidazole phosphate + 0.13 mmol His-Lys

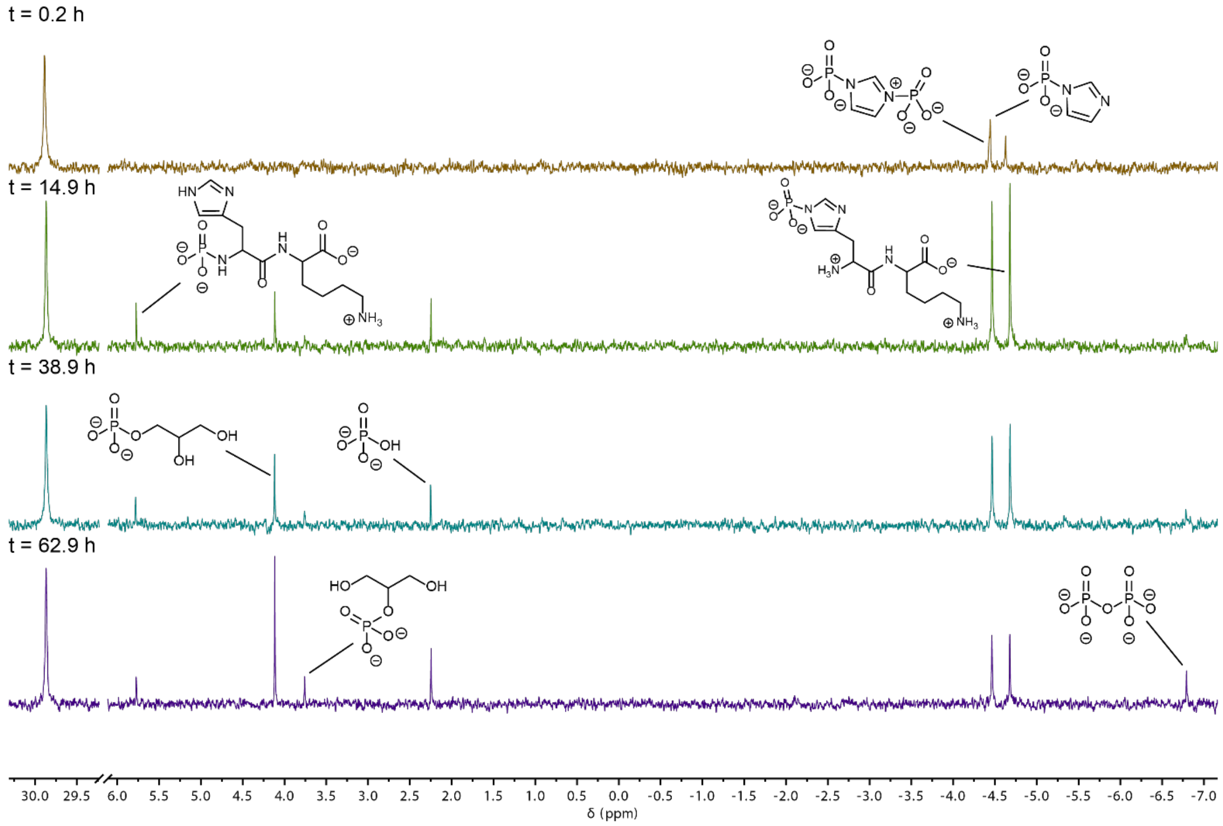

Supporting Figure 50: Representative ^{31}P-NMR spectra over time for the reaction of 0.13 mmol of calcium imidazole phosphate, 3.25 mmol of glycerol and 0.13 mmol of His-Lys at pH 7.5 and 22 °C.

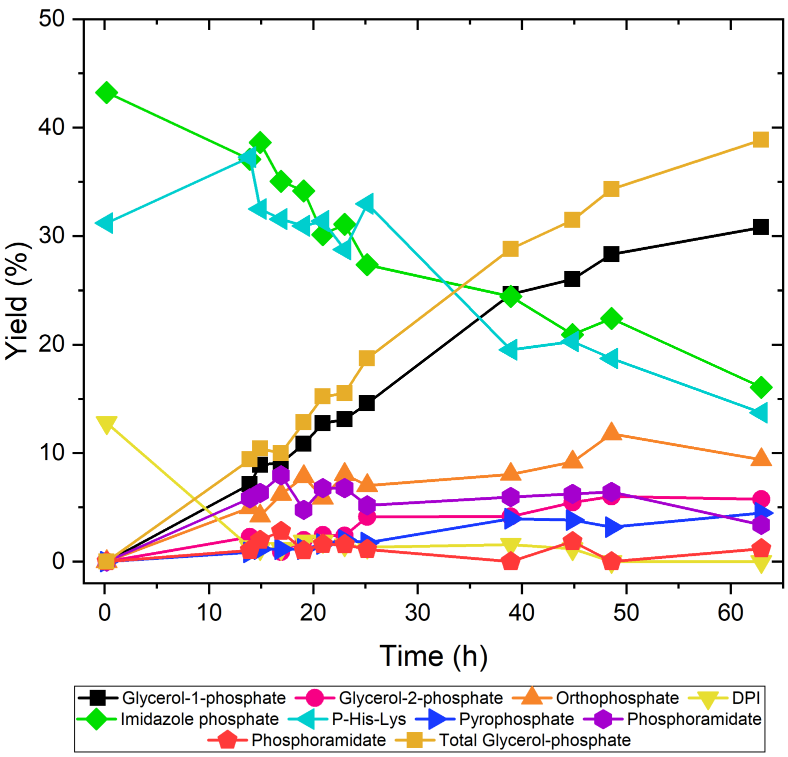

Supporting Figure 51: Changes in yield over time for the reaction of 0.13 mmol of calcium imidazole phosphate, 3.25 mmol of glycerol and 0.13 mmol of His-Lys at pH 7.5 and 22 °C. DPI = Diphosphoimidazole. The first time point includes phosphorylation that took place in the freezer at -20 °C and thus the yields of imidazole phosphate, diphosphoimidazole and P-His-Lys are for this time point off.

S3.4.3 Experiment 3 - 3.25 mmol glycerol + 0.13 mmol imidazole phosphate + 0.13 mmol His-Lys

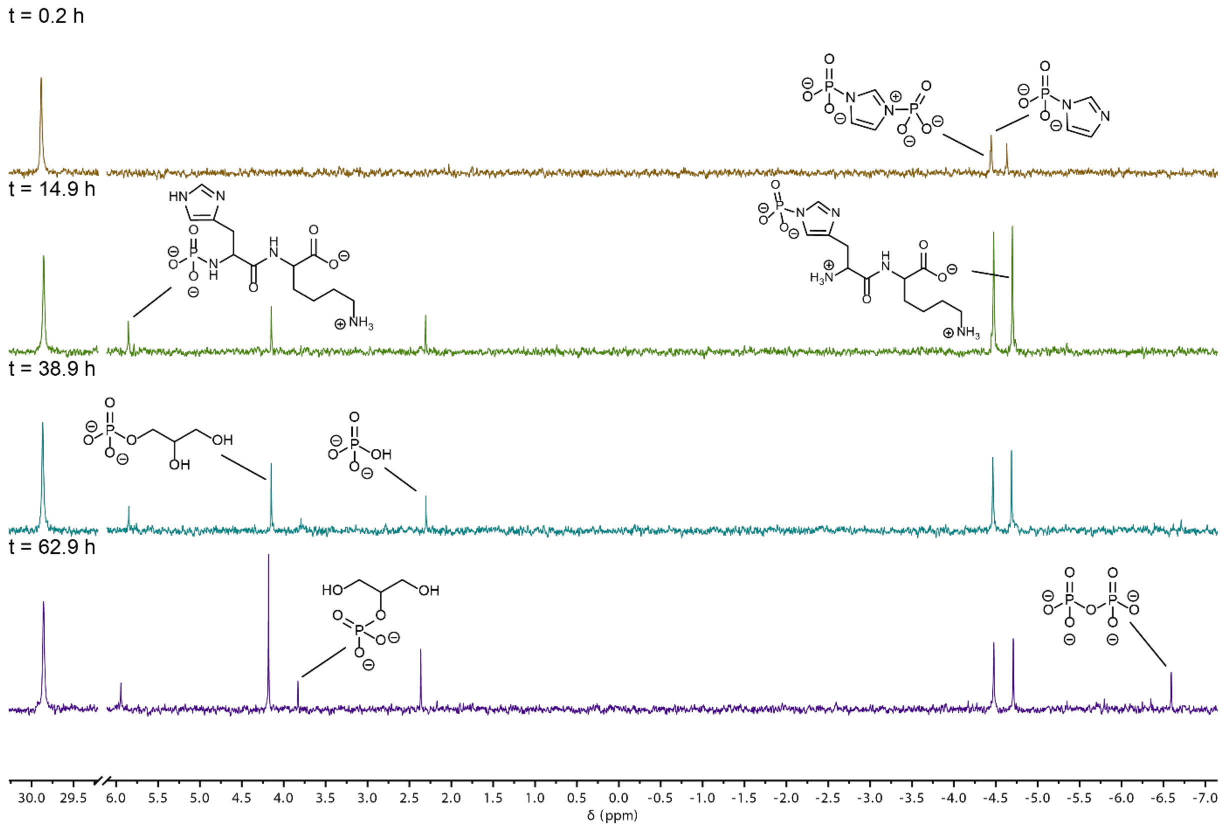

Supporting Figure 52: Representative ^{31}P-NMR spectra over time for the reaction of 0.13 mmol of calcium imidazole phosphate, 3.25 mmol of glycerol and 0.13 mmol of His-Lys at pH 7.5 and 22 °C.

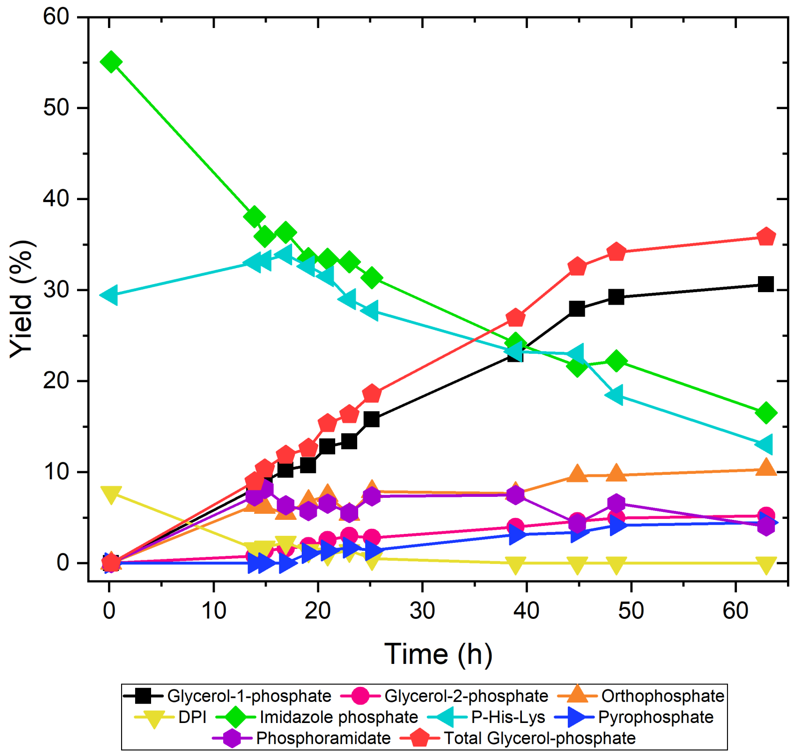

Supporting Figure 53: Changes in yield over time for the reaction of 0.13 mmol of calcium imidazole phosphate, 3.25 mmol of glycerol and 0.13 mmol of His-Lys at pH 7.5 and 22 °C. DPI = Diphosphoimidazole. The first time point includes phosphorylation that took place in the freezer at -20 °C and thus the yields of imidazole phosphate, diphosphoimidazole and P-His-Lys are for this time point off.

S3.4.4 *Combined results for 3.25 mmol glycerol + 0.13 mmol imidazole phosphate + 0.13 mmol His-Lys*

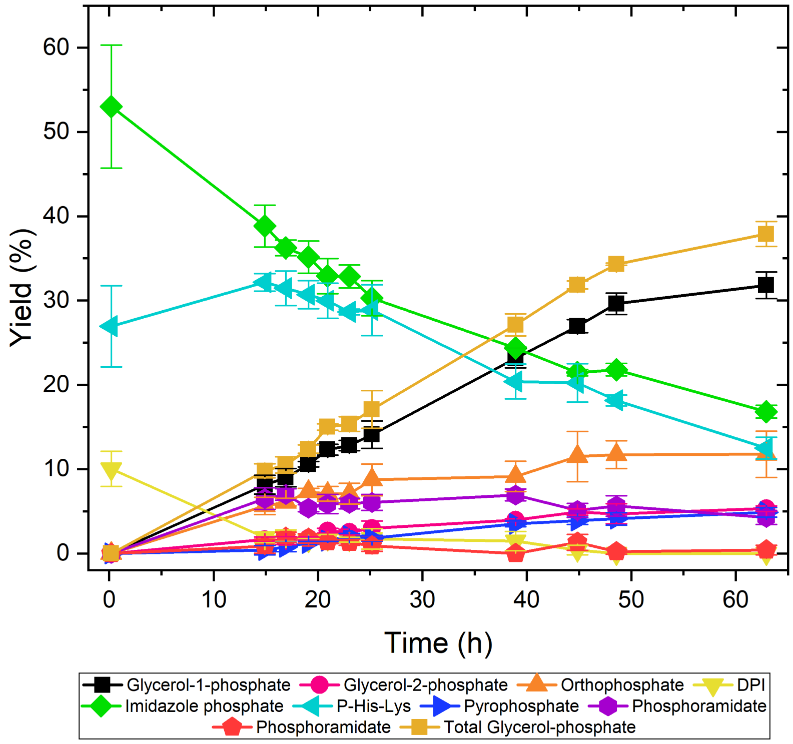

Supporting Figure 54: Changes in yield over time for the reaction of 0.13 mmol of calcium imidazole phosphate, 3.25 mmol of glycerol and 0.13 mmol of His-Lys at pH 7.5 and 22 °C. DPI = Diphosphoimidazole. These data are the mean values and standard deviation based upon triplicate experiments. The first time point includes phosphorylation that took place in the freezer at -20 °C and thus the yields of imidazole phosphate, diphosphoimidazole and P-His-Lys are for this time point off.

S3.4.5 *Comparison between samples measured immediately and samples stored at -20 °C for 1 week for 3.25 mmol glycerol + 0.13 mmol imidazole phosphate + 0.13 mmol His-Lys*

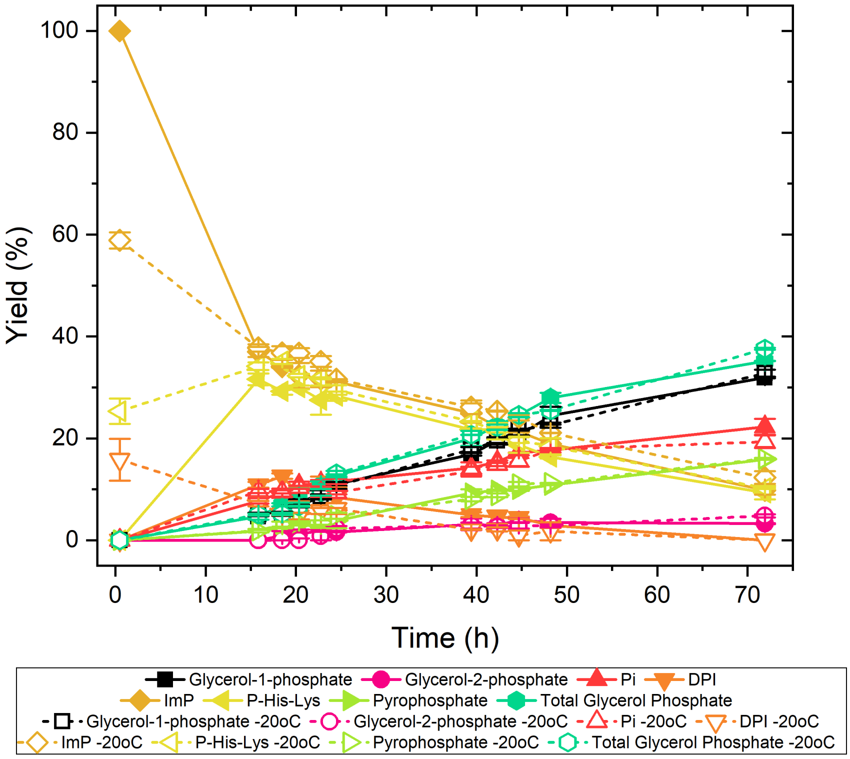

Supporting Figure 55: The changes in yield over time for the reaction of 0.13 mmol of calcium imidazole phosphate, 3.25 mmol of glycerol and 0.13 mmol of His-Lys at pH 7.5 and 22 °C. Solid lines are for the samples measured immediately and dashed lines are for samples measured after being stored for 1 week at -20 °C. These data are the mean values and standard deviation based upon duplicate experiments.

S3.5 Phosphorylation of glycerol by imidazole phosphate with His-Gly-Gly catalyst

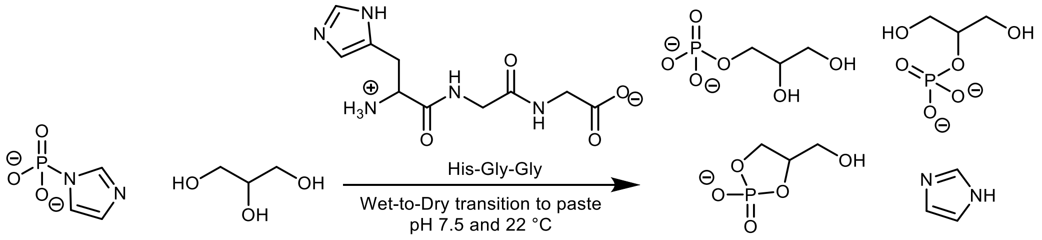

The experiment was carried out according to the procedure in S3.1 but with the 65 mM of histidine replaced by 65 mM His-Gly-Gly (35.0 mg, 0.13 mmol). The experiment was repeated in triplicate. Supporting Figures 56, 58 and 60 depict representative ^{31}P NMR spectra for the reaction over time. The changes in yield over time for all phosphate containing species are shown in Supporting Figures 57, 59 and 61. The mean experimental results with the standard deviation of each experimental data point from the triplicate experiments are shown in Supporting Figure 62.

S3.5.1 Experiment 1 - 3.25 mmol glycerol + 0.13 mmol imidazole phosphate + 0.13 mmol His-Gly-Gly

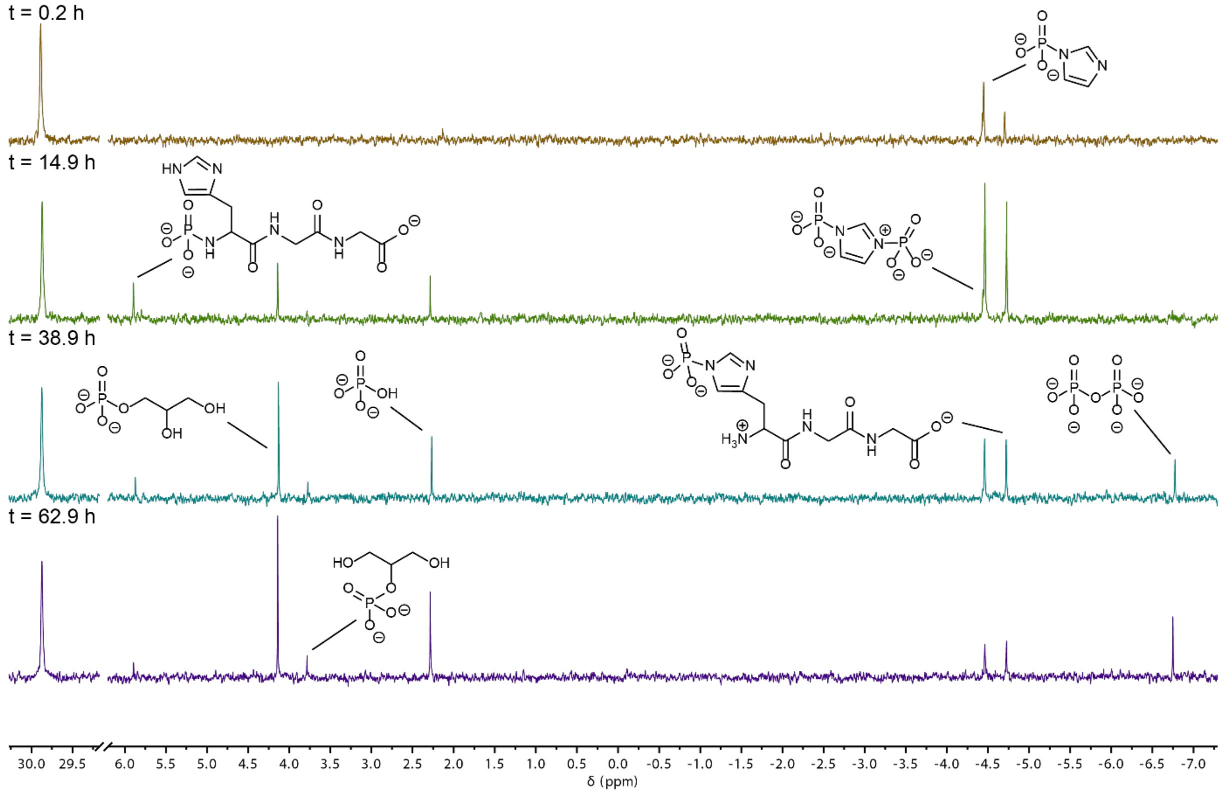

Supporting Figure 56: Representative ^{31}P-NMR spectra over time for the reaction of 0.13 mmol of calcium imidazole phosphate, 3.25 mmol of glycerol and 0.13 mmol of His-Gly-Gly at pH 7.5 and 22 °C.

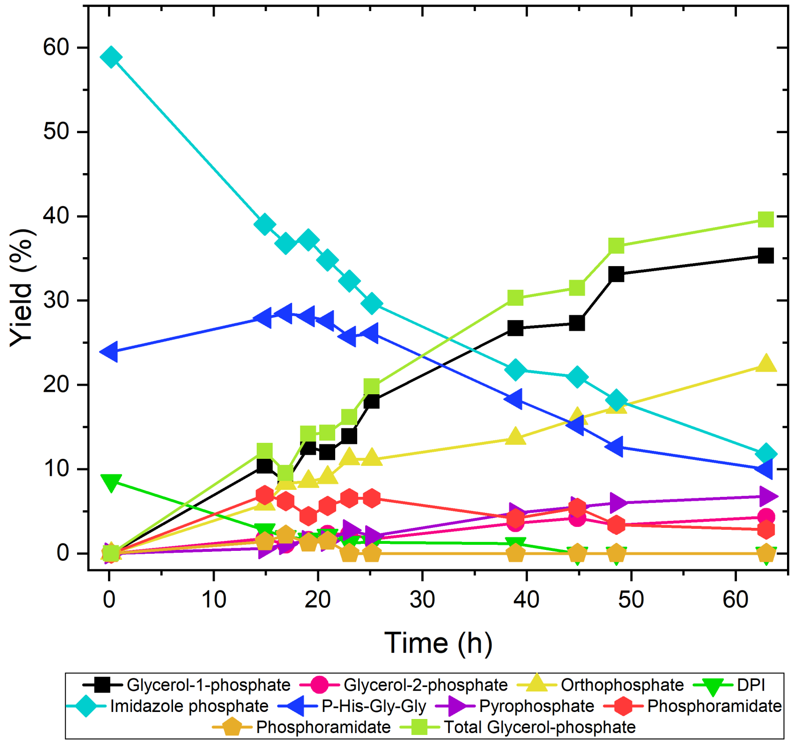

Supporting Figure 57: Changes in yield over time for the reaction of 0.13 mmol calcium imidazole phosphate, 3.25 mmol glycerol and 0.13 mmol His-Gly-Gly at pH 7.5 and 22 °C. DPI = Diphosphoimidazole. The first time point includes phosphorylation that took place in the freezer at -20 °C and thus the yields of imidazole phosphate, diphosphoimidazole and P-His-Gly-Gly are for this time point off.

S3.5.2 Experiment 2 - 3.25 mmol glycerol+0.13 mmol imidazole phosphate+0.13 mmol His-Gly-Gly

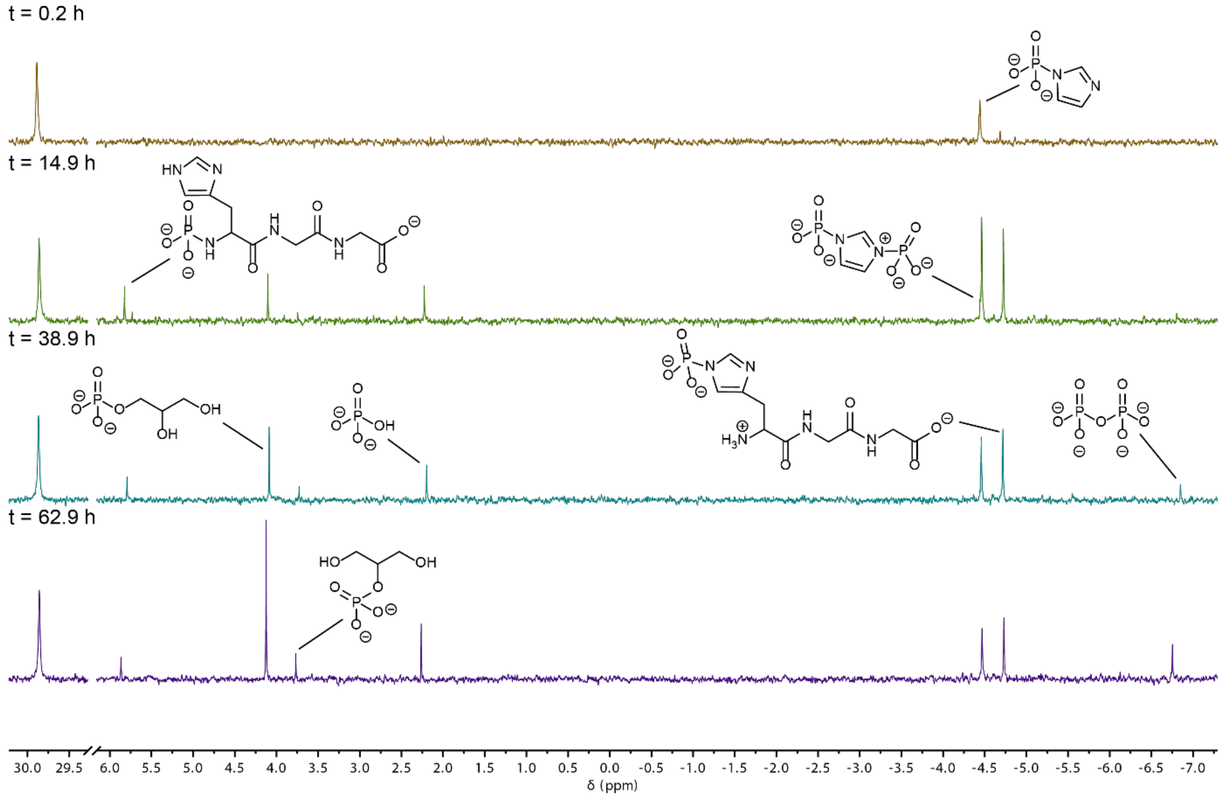

Supporting Figure 58: Representative ^{31}P-NMR spectra over time for the reaction of 0.13 mmol of calcium imidazole phosphate, 3.25 mmol of glycerol and 0.13 mmol of His-Gly-Gly at pH 7.5 and 22 °C.

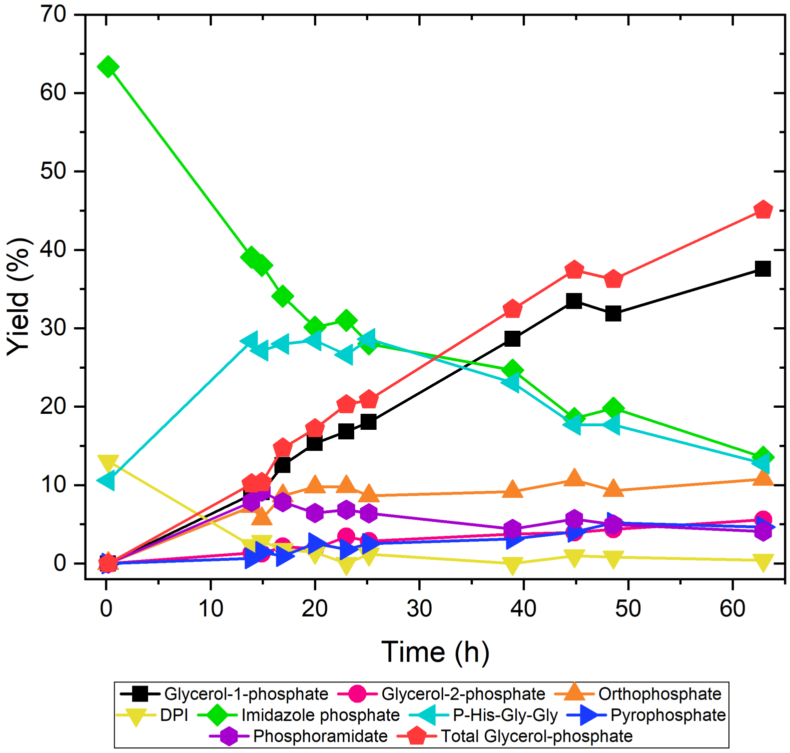

Supporting Figure 59: Changes in yield over time for the reaction of 0.13 mmol calcium imidazole phosphate, 3.25 mmol glycerol and 0.13 mmol His-Gly-Gly at pH 7.5 and 22 °C. DPI = Diphosphoimidazole. The first time point includes phosphorylation that took place in the freezer at -20 °C and thus the yields of imidazole phosphate, diphosphoimidazole and P-His-Gly-Gly are for this time point off.

S3.5.3 Experiment 3 - 3.25 mmol glycerol + 0.13 mmol imidazole phosphate + 0.13 mmol His-Gly-Gly

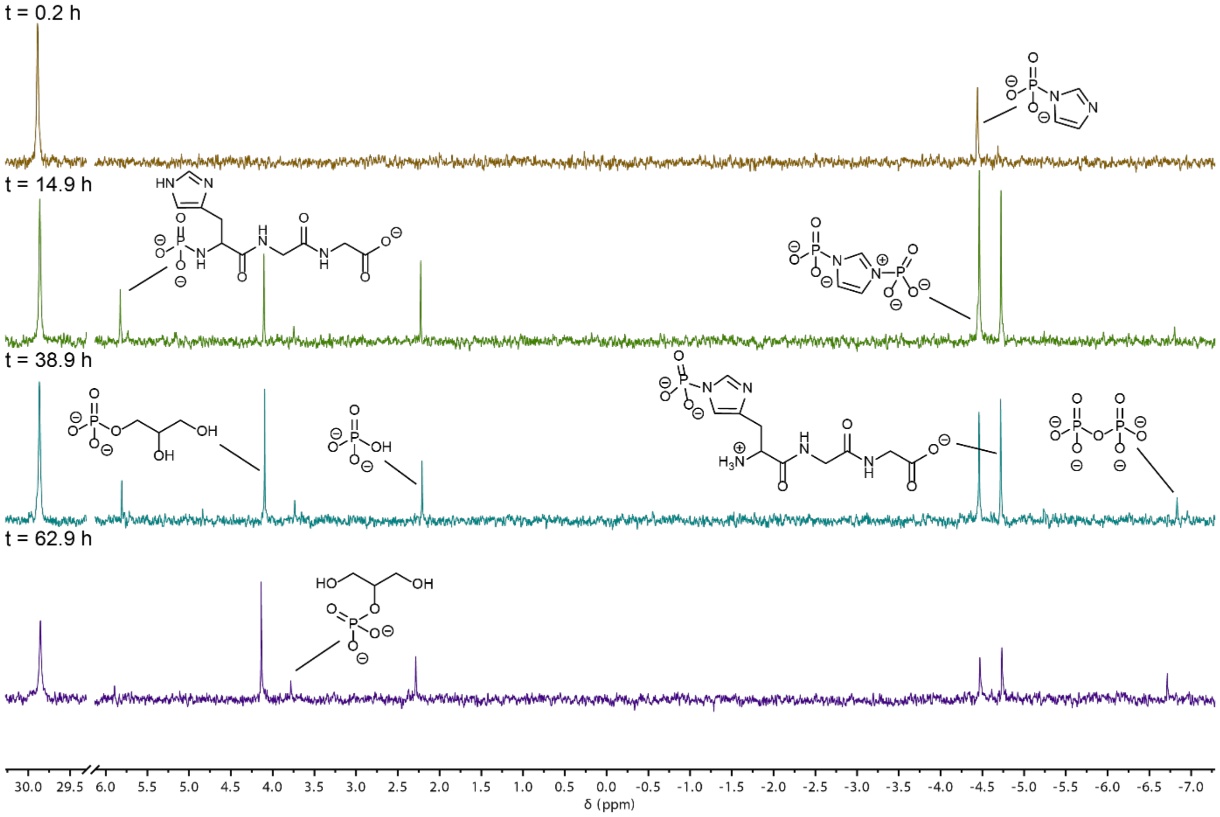

Supporting Figure 60: Representative ^{31}P-NMR spectra over time for the reaction of 0.13 mmol of calcium imidazole phosphate, 3.25 mmol of glycerol and 0.13 mmol of His-Gly-Gly at pH 7.5 and 22 °C.

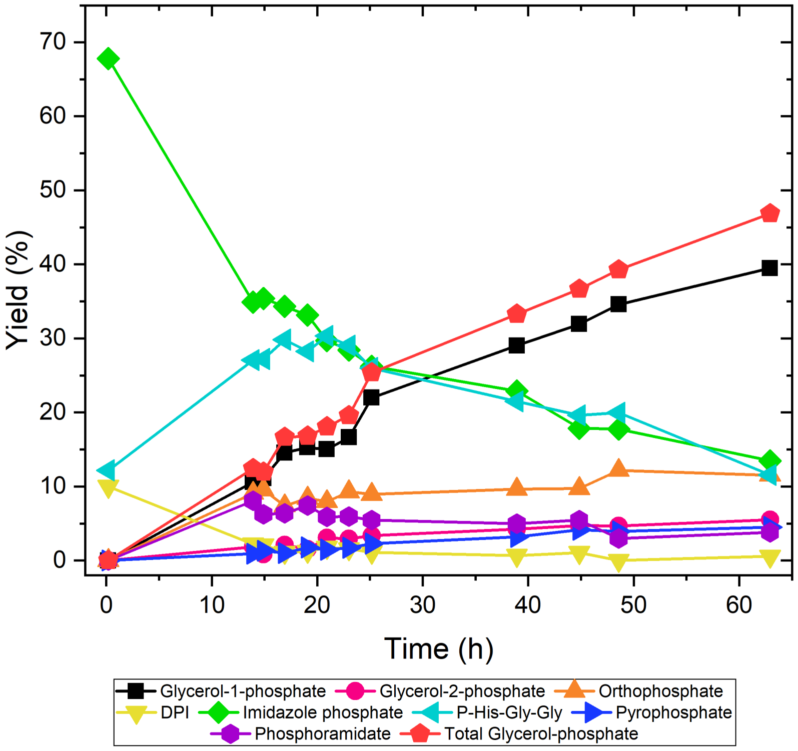

Supporting Figure 61: Changes in yield over time for the reaction of 0.13 mmol calcium imidazole phosphate, 3.25 mmol glycerol and 0.13 mmol His-Gly-Gly at pH 7.5 and 22 °C. DPI = Diphosphoimidazole. The first time point includes phosphorylation that took place in the freezer at -20 °C and thus the yields of imidazole phosphate, diphosphoimidazole and P-His-Gly-Gly are for this time point off.

S3.5.4 Combined results for 3.25 mmol glycerol + 0.13 mmol imidazole phosphate + 0.13 mmol His-Gly-Gly

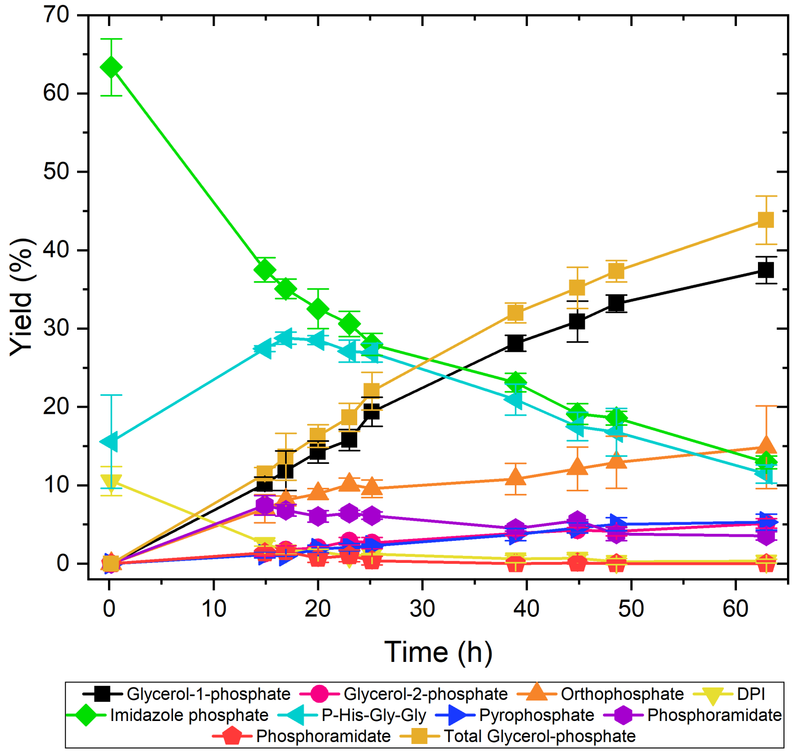

Supporting Figure 62: Changes in yield over time for the reaction of 0.13 mmol calcium imidazole phosphate, 3.25 mmol glycerol and 0.13 mmol His-Gly-Gly at pH 7.5 and 22 °C. DPI = Diphosphoimidazole. These data are the mean values and standard deviation based upon triplicate experiments. The first time point includes phosphorylation that took place in the freezer at -20 °C and thus the yields of imidazole phosphate, diphosphoimidazole and P-His-Gly-Gly are for this time point off.

S3.6 Phosphorylation of glycerol by imidazole phosphate with His-His catalyst (0.5 equivalent)

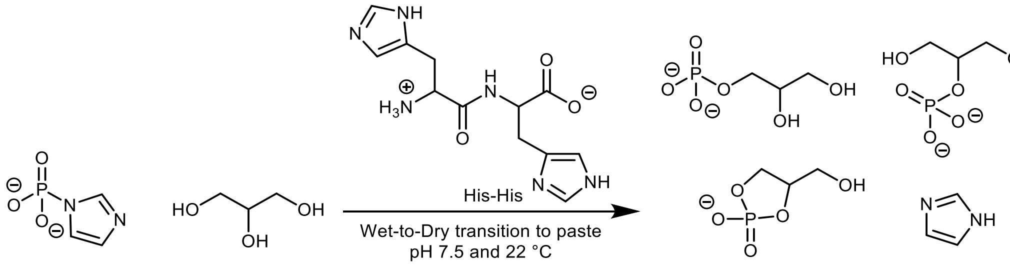

The experiment was carried out according to the procedure in S3.1 but with the 65 mM of histidine replaced by 32.5 mM His-His TFA salt (26.4 mg, 0.065 mmol). The experiment was repeated in duplicate. Supporting Figures 63 and 65 depict representative ^{31}P NMR spectra for the reaction over time. The changes in yield over time for all phosphate containing species are shown in Supporting Figures 64 and 66. The mean experimental results with the standard deviation of each experimental data point from the triplicate experiments are shown in Supporting Figure 67.

S3.6.1 Experiment 1 - 3.25 mmol glycerol + 0.13 mmol imidazole phosphate + 0.07 mmol His-His

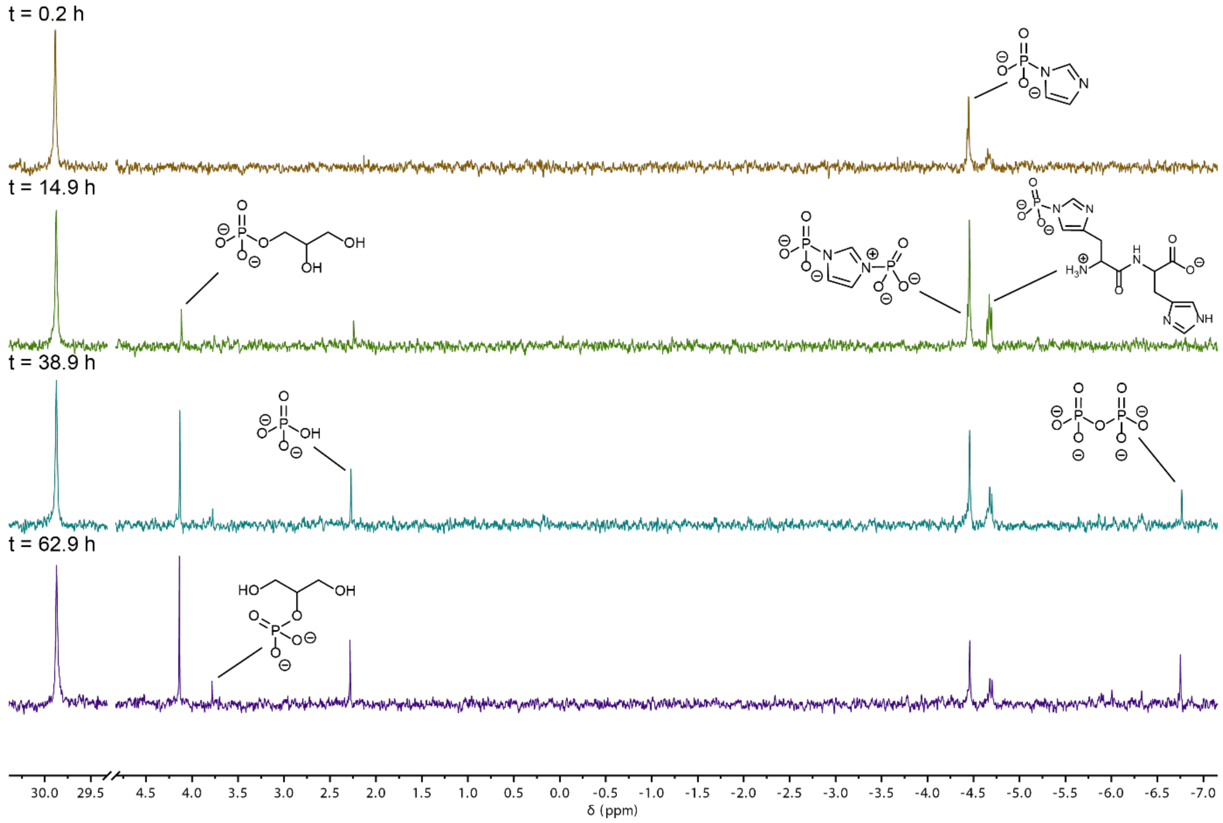

Supporting Figure 63: Representative ^{31}P-NMR spectra over time for the reaction of 0.13 mmol of calcium imidazole phosphate, 3.25 mmol of glycerol and 0.07 mmol of His-His at pH 7.5 and 22 °C.

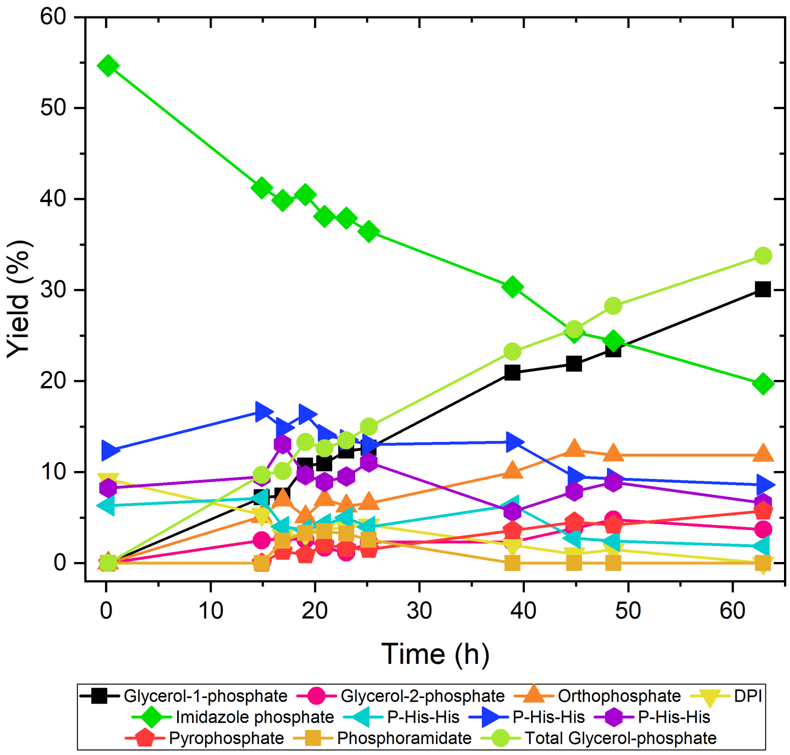

Supporting Figure 64: Changes in yield over time for the reaction of 0.13 mmol calcium imidazole phosphate, 3.25 mmol glycerol and 0.07 mmol His-His at pH 7.5 and 22 °C. DPI = Diphosphoimidazole. The first time point includes phosphorylation that took place in the freezer at -20 °C and thus the yields of imidazole phosphate, diphosphoimidazole and P-His-His are for this time point off.

S3.6.2 Experiment 2 - 3.25 mmol glycerol + 0.13 mmol imidazole phosphate + 0.07 mmol His-His

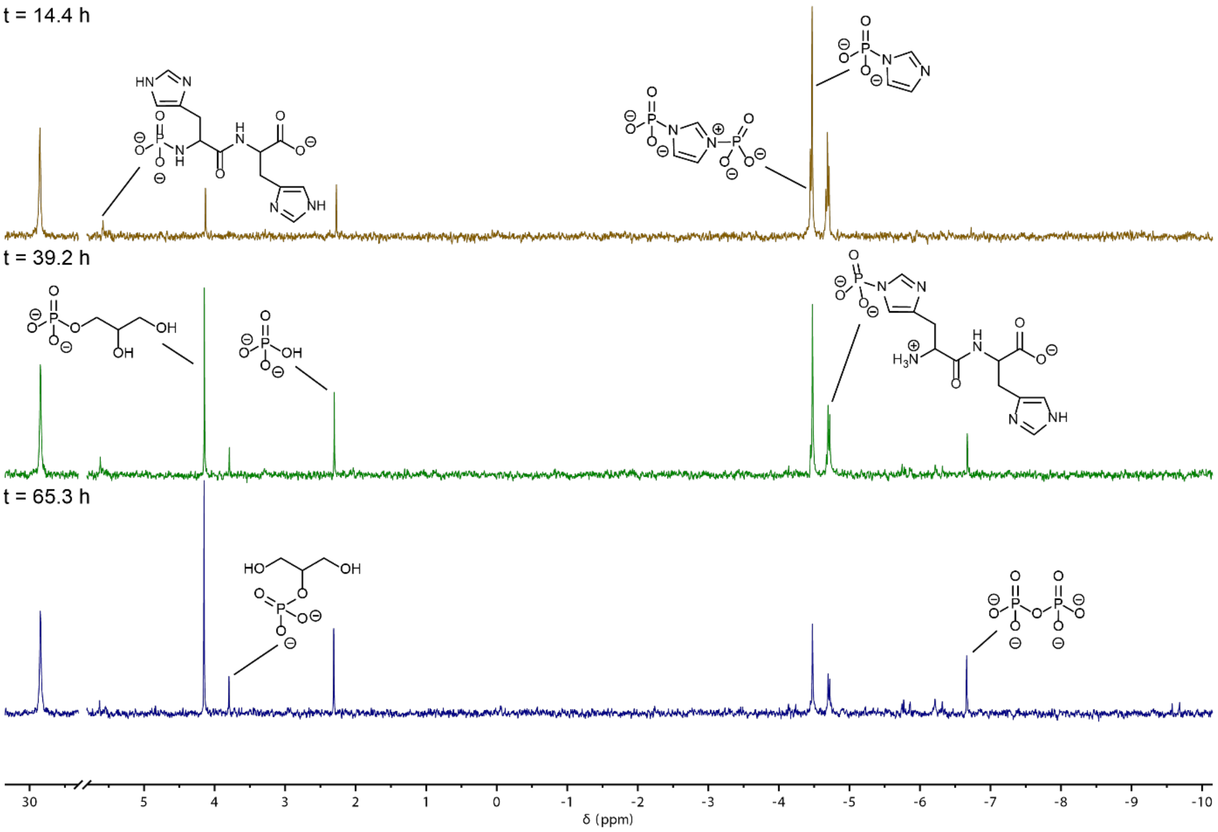

Supporting Figure 65: Representative ^{31}P-NMR spectra over time for the reaction of 0.13 mmol of calcium imidazole phosphate, 3.25 mmol of glycerol and 0.07 mmol of His-His at pH 7.5 and 22 °C.

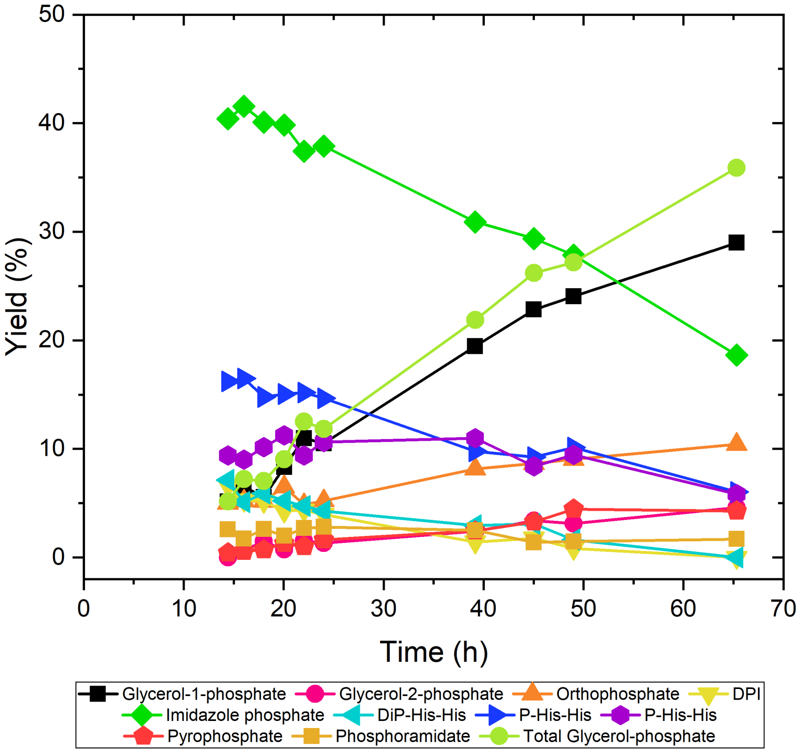

Supporting Figure 66: Changes in yield over time for the reaction of 0.13 mmol of calcium imidazole phosphate, 3.25 mmol of glycerol and 0.07 mmol of His-His at pH 7.5 and 22 °C. DPI = Diphosphoimidazole.

S3.6.3 Combined results for 3.25 mmol glycerol + 0.13 mmol imidazole phosphate + 0.07 mmol His-His

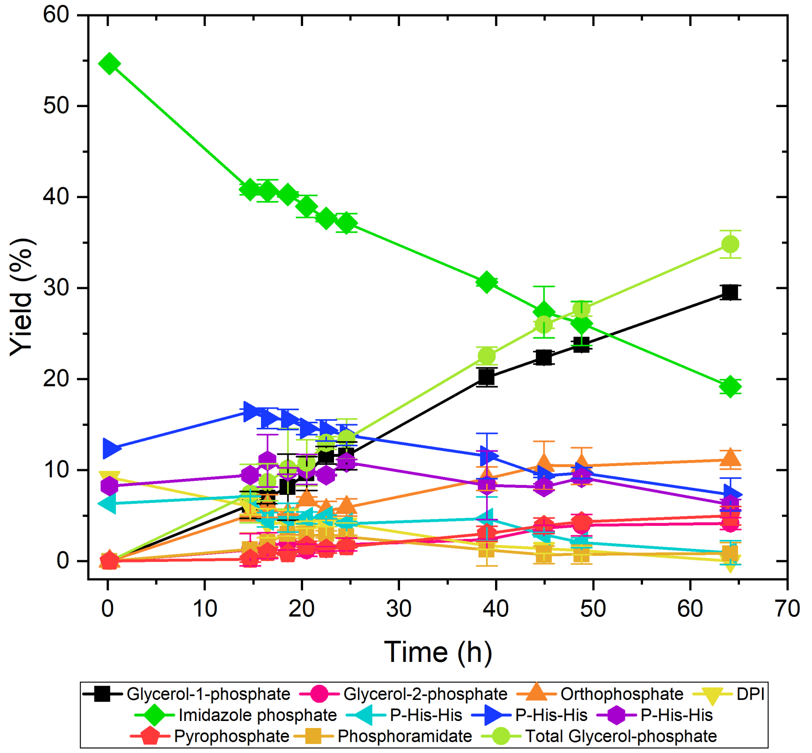

Supporting Figure 67: Changes in yield over time for the reaction of 0.13 mmol of calcium imidazole phosphate, 3.25 mmol of glycerol and 0.07 mmol of His-His at pH 7.5 and 22 °C. DPI = Diphosphoimidazole. These data are the mean values and standard deviation based upon duplicate experiments. The first time point includes phosphorylation that took place in the freezer at -20 °C and thus the yields of imidazole phosphate, diphosphoimidazole and P-His-His are for this time point off.

S3.6.4 Characterisation of phosphorylated His-His intermediate

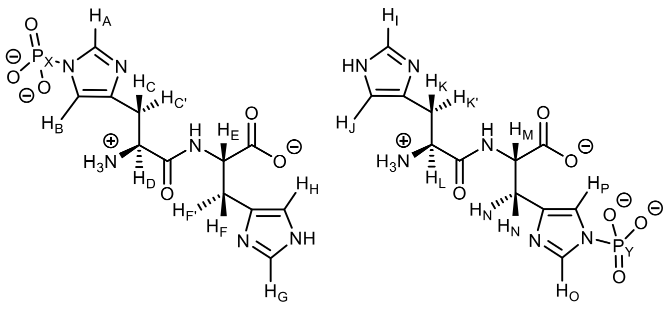

The phosphorylated His-His intermediates was characterised *in situ*. Both of the above intermediates were formed in solution with a higher concentration of phosphorylation on the N-terminal His compared to the C-terminal His. A solution of 50 mM His-His and 50 mM calcium imidazole phosphate in 0.5 mL 0.5 M MOPS buffer at pH 7.5 in 9 : 1 H_2O : D_2O containing 0.1 M citric acid and 50 mM HMPA internal standard was prepared according to the procedure in Section S2.2 and S2.4.

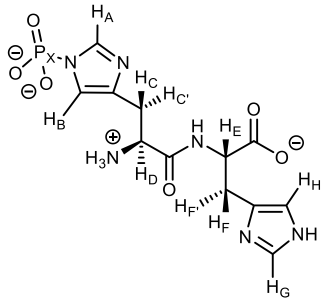

^{31}P NMR (202.46 MHz, 0.5 M MOPS + 0.1 M Citric Acid in 9 : 1 H_2O : D_2O at pH 7.5 and 22 °C): *phosphorylated N-terminal His-His intermediate* δ (ppm) = - 4.83 (s, 1P, P_x). ^{1}H NMR (500.13 MHz, 0.5 M MOPS + 0.1 M Citric Acid in 9 : 1 H_2O : D_2O at pH 7.5 and 22 °C): *phosphorylated N-terminal His-His intermediate* δ (ppm) = 7.98 (s, 1H, H_A), 7.78 (s, 1H, H_G), 7.05 (s, 1H, H_B), 6.93 (s, 1H, H_H), 4.31 (dd, 1H, H_E), 3.84 (dd, 1H, H_D), 2.97 (dd, 2H, $H_{C+C'}$), 3.06 (s, 1H, $H_{F/F'}$), 2.95 (s, 1H, $H_{F'/F}$). ^{13}C NMR (125.77 MHz, 0.5 M MOPS + 0.1 M Citric Acid in 9 : 1 H_2O : D_2O at pH 7.5 and 22 °C): *phosphorylated N-terminal His-His intermediate* δ (ppm) = 177.1 (s, 1C, COOH), 172.5 (s, 1C, Amide N-C=O), 136.9 (s, 1C, imid C-H_A), 133.7 (s, 1C, N-terminal P-His imid proximal), 130.8 (s, 1C, N-terminal P-His imid distant), 118.2 (s, 1C, imid C-H_B), 136.3 (s, 1C, C-H_G), 118.5 (s, 1C, C-H_H), 53.7 (s, 1C, C-H_D), 29.8 (s, 1C, C-$H_{C+C'}$), 55.0 (s, 1C, C-H_E), 28.5 (s, 1C, C-$H_{F+F'}$).

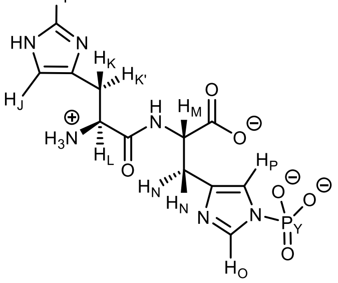

^{31}P NMR (202.46 MHz, 0.5 M MOPS + 0.1 M Citric Acid in 9 : 1 H_2O : D_2O at pH 7.5 and 22 °C): *phosphorylated C-terminal His-His intermediate* δ (ppm) = - 4.85 (s, 1P, P_Y). ^{1}H NMR (500.13 MHz, 0.5 M MOPS + 0.1 M Citric Acid in 9 : 1 H_2O : D_2O at pH 7.5 and 22 °C): *phosphorylated C-terminal His-His intermediate* δ (ppm) = 7.93 (s, 1H, H_O), 7.83 (s, 1H, H_I), 7.11 (s, 1H, H_P), 6.91 (s, 1H, H_J), 4.33 (dd, 1H, H_M), 3.90 (dd, 1H, H_L), 3.06 (dd, 1H, $H_{N/N'}$), 2.92 (dd, 1H, $H_{N'/N}$), 3.00 (dd, 1H, $H_{K/K'}$), 2.94 (dd, 1H, $H_{K'/K}$). ^{13}C NMR (125.77 MHz, 0.5 M MOPS + 0.1 M Citric Acid in 9 : 1 H_2O : D_2O at pH 7.5 and 22 °C): *phosphorylated C-terminal His-His intermediate* δ (ppm) = 177.1 (s, 1C, COOH), 172.5 (s, 1C, Amide N-C=O), 137.8 (s, 1C, imid C-H_O), 132.3 (s, 1C, C-terminal P-His imid proximal), 132.3 (s, 1C, C-terminal P-His imid distant), 119.2 (s, 1C, imid C-H_P), 135.7 (s, 1C, C-H_I), 117.9 (s, 1C, C-H_J), 54.9 (s, 1C, C-H_L), 55.0 (s, 1C, C-H_M), 29.6 (s, 1C, C-$H_{K+K'}$), 28.4 (s, 1C, C-H_N).

S3.7 Phosphorylation of glycerol by imidazole phosphate with His-His catalyst (1 equivalent)

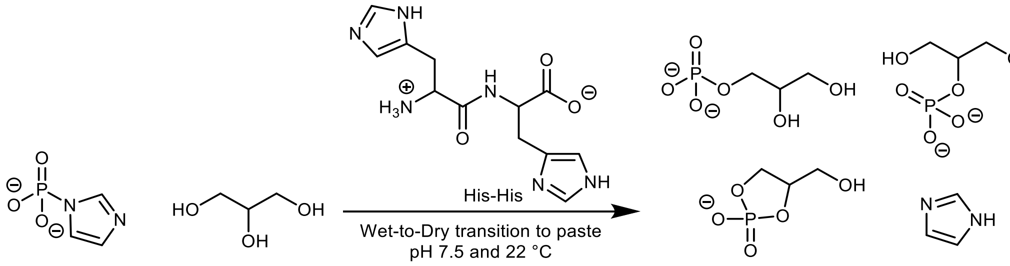

The experiment was carried out according to the procedure in S3.1 but with the 65 mM of histidine replaced by 65 mM His-His TFA salt (52.8 mg, 0.13 mmol). The experiment was repeated in triplicate. Supporting Figures 68, 70 and 72 depict representative ^{31}P NMR spectra for the reaction over time. The changes in yield over time for all phosphate containing species are shown in Supporting Figures 69, 71 and 73. The mean experimental results with the standard deviation of each experimental data point from the triplicate experiments are shown in Supporting Figure 74.

S3.7.1 Experiment 1 - 3.25 mmol glycerol + 0.13 mmol imidazole phosphate + 0.13 mmol His-His

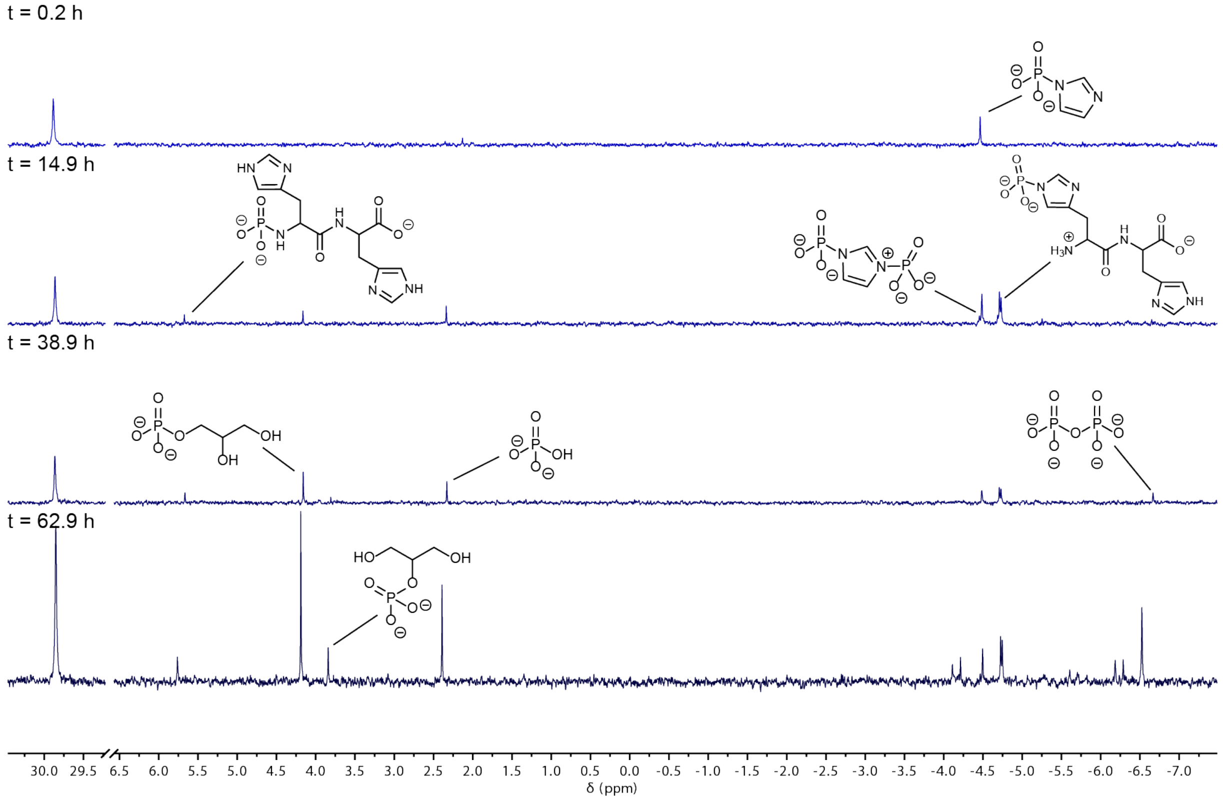

Supporting Figure 68: Representative ^{31}P-NMR spectra over time for the reaction of 0.13 mmol of calcium imidazole phosphate, 3.25 mmol of glycerol and 0.07 mmol of His-His at pH 7.5 and 22 °C.

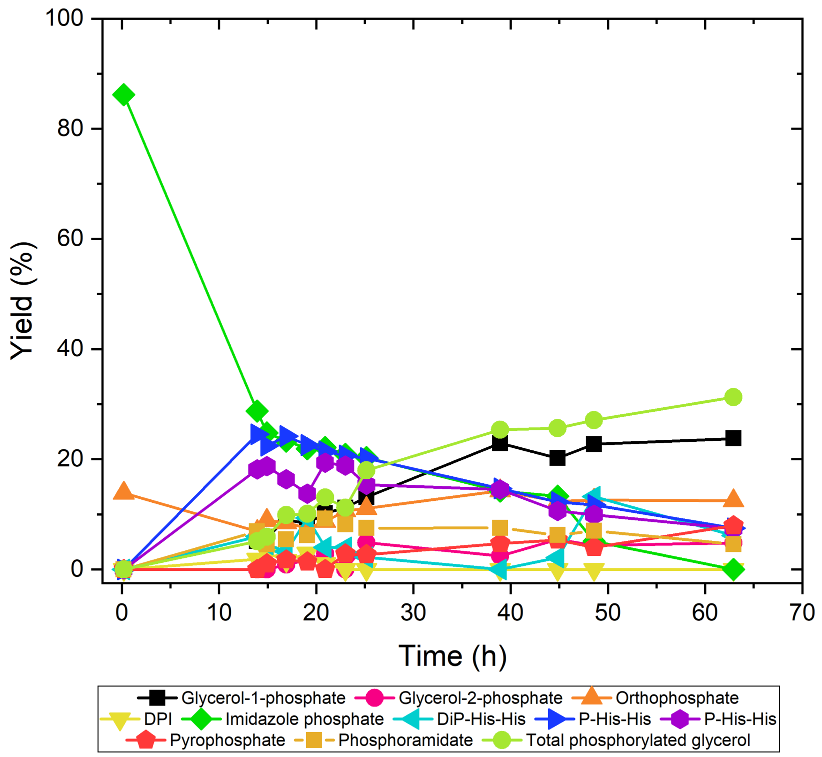

Supporting Figure 69: Changes in yield over time for the reaction of 0.13 mmol of calcium imidazole phosphate, 3.25 mmol of glycerol and 0.13 mmol of His-His at pH 7.5 and 22 °C. DPI = Diphosphoimidazole.

S3.7.2 Experiment 2 - 3.25 mmol glycerol + 0.13 mmol imidazole phosphate + 0.13 mmol His-His

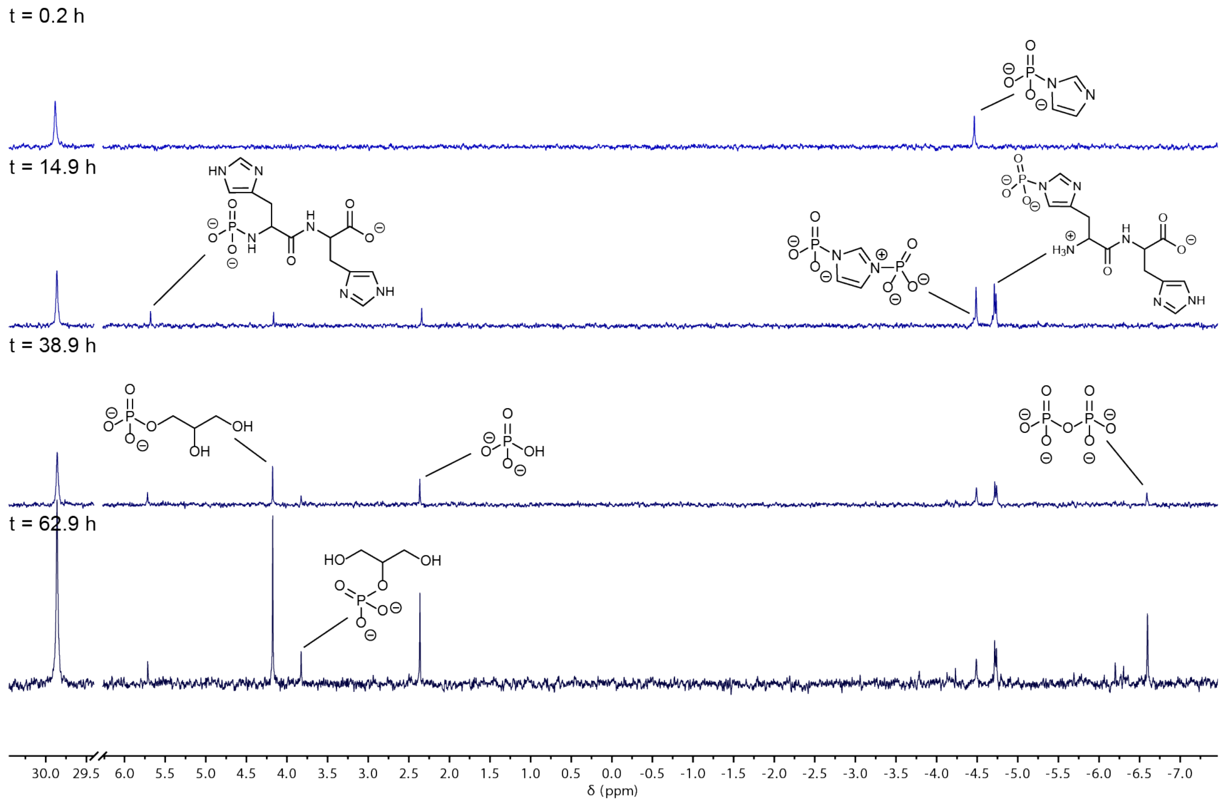

Supporting Figure 70: Representative ^{31}P-NMR spectra over time for the reaction of 0.13 mmol of calcium imidazole phosphate, 3.25 mmol of glycerol and 0.13 mmol of His-His at pH 7.5 and 22 °C.

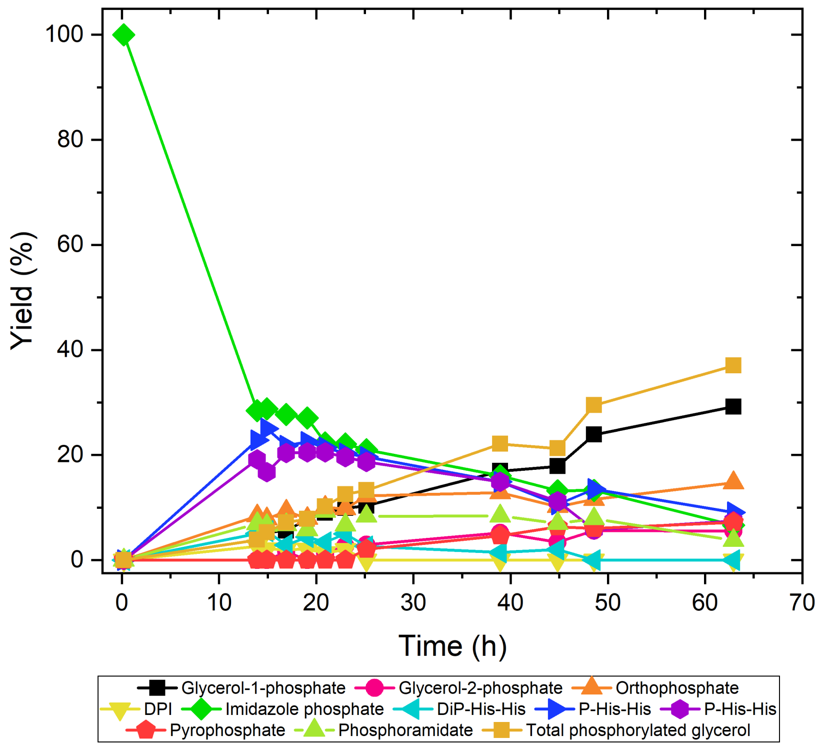

Supporting Figure 71: Changes in yield over time for the reaction of 0.13 mmol of calcium imidazole phosphate, 3.25 mmol of glycerol and 0.13 mmol of His-His at pH 7.5 and 22 °C. DPI = Diphosphoimidazole.

S3.7.3 Experiment 3 - 3.25 mmol glycerol + 0.13 mmol imidazole phosphate + 0.13 mmol His-His

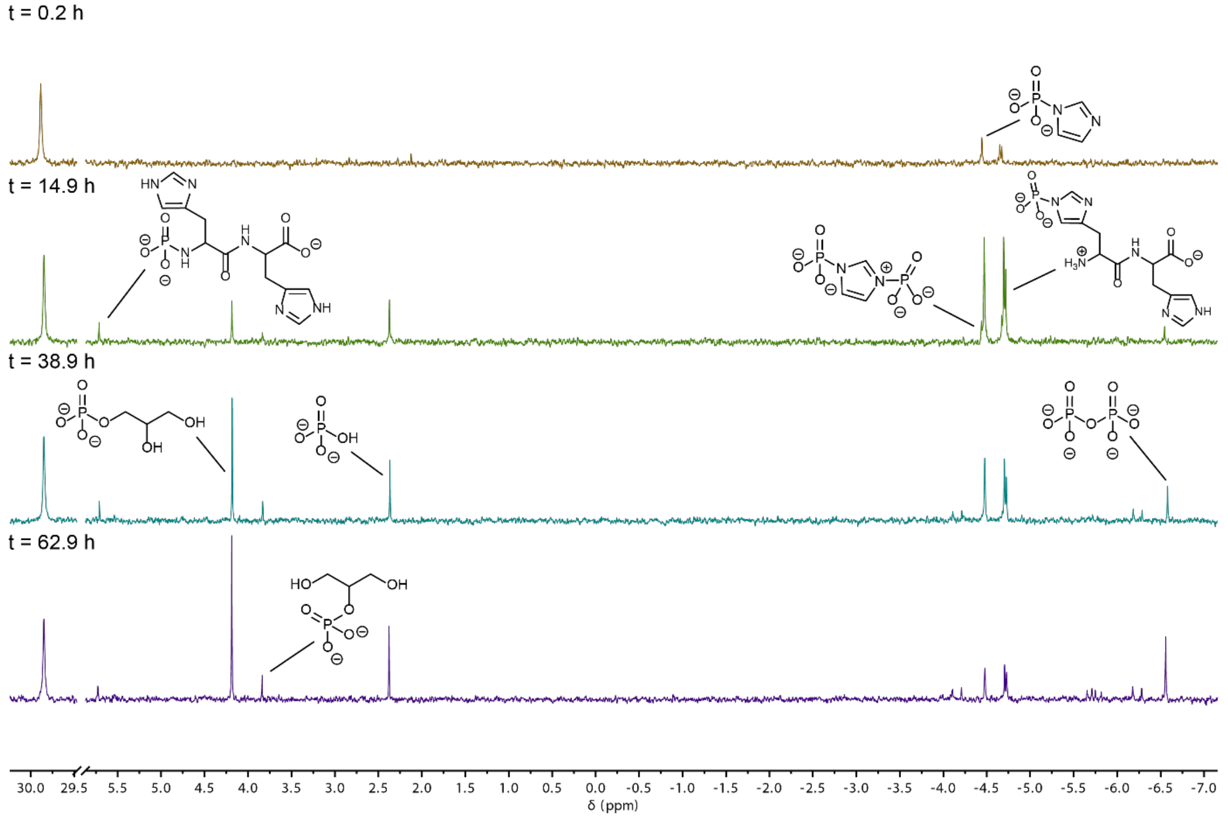

Supporting Figure 72: Representative ^{31}P-NMR spectra over time for the reaction of 0.13 mmol of calcium imidazole phosphate, 3.25 mmol of glycerol and 0.13 mmol of His-His at pH 7.5 and 22 °C.

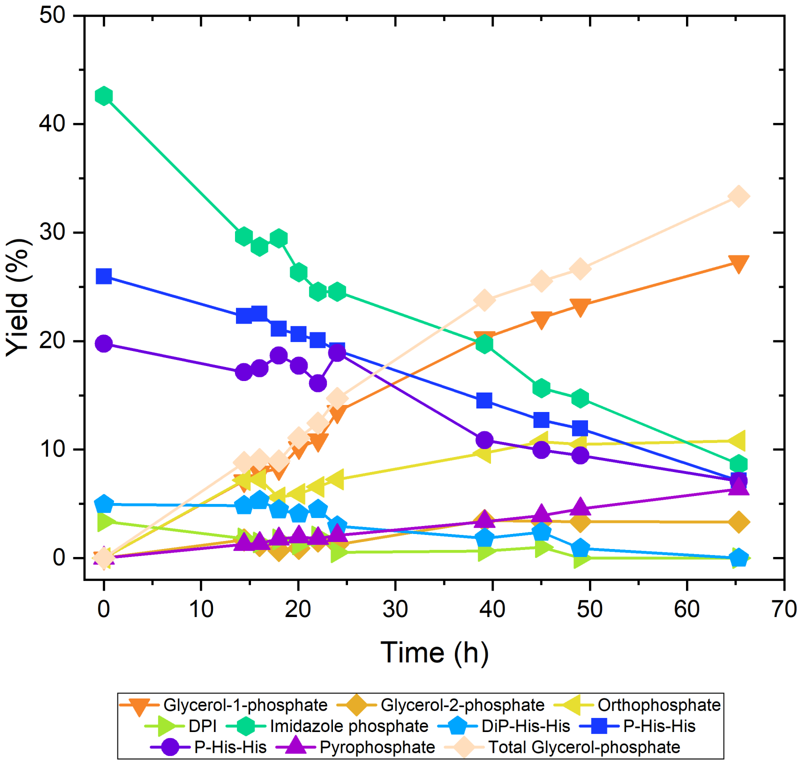

Supporting Figure 73: Changes in yield over time for the reaction of 0.13 mmol of calcium imidazole phosphate, 3.25 mmol of glycerol and 0.13 mmol of His-His at pH 7.5 and 22 °C. DPI = Diphosphoimidazole. The first time point includes phosphorylation that took place in the freezer at -20 °C and thus the yields of imidazole phosphate, diphosphoimidazole and P-His-His are for this time point off.

S3.7.4 Combined results for 3.25 mmol glycerol + 0.13 mmol imidazole phosphate + 0.13 mmol His-His

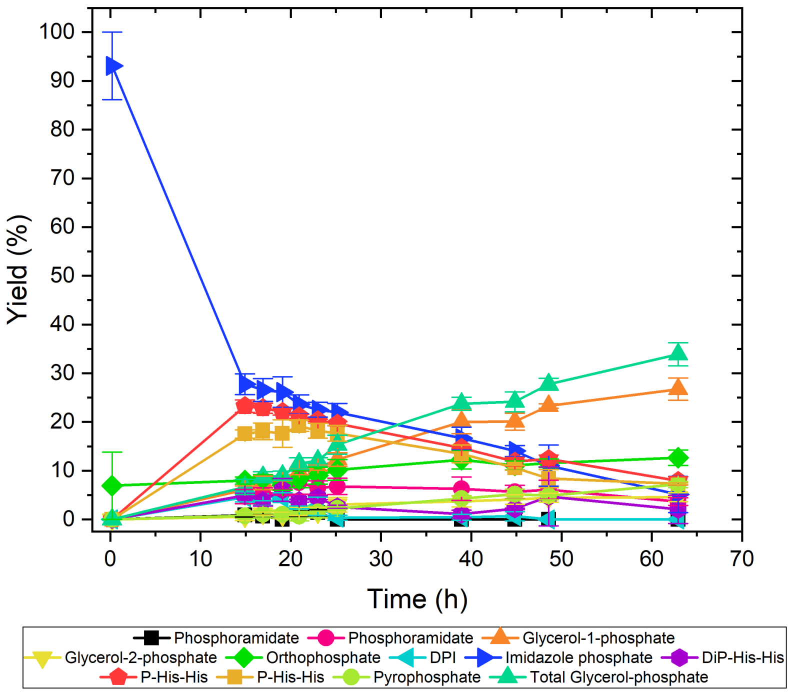

Supporting Figure 74: Changes in yield over time for the reaction of 0.13 mmol of calcium imidazole phosphate, 3.25 mmol of glycerol and 0.13 mmol of His-His at pH 7.5 and 22 °C. DPI = Diphosphoimidazole. These data are the mean values and standard deviation based upon triplicate experiments.

S3.8 Uncatalysed phosphorylation of glycerol by imidazole phosphate

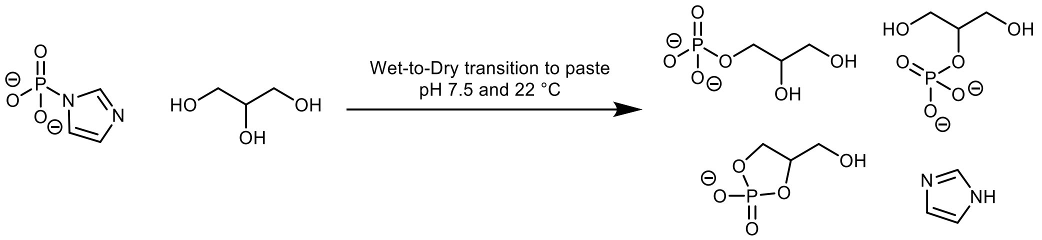

The experiment was carried out according to the procedure in S3.1 but with no 65 mM of histidine. The experiment was repeated in triplicate. Supporting Figures 75, 77 and 79 depict representative ^{31}P NMR spectra for the reaction over time. The changes in yield over time for all phosphate containing species are shown in Supporting Figures 76, 78 and 80. The mean experimental results with the standard deviation of each experimental data point from the triplicate experiments are shown in Supporting Figure 81.

S3.8.1 Experiment 1 - 3.25 mmol glycerol + 0.13 mmol imidazole phosphate

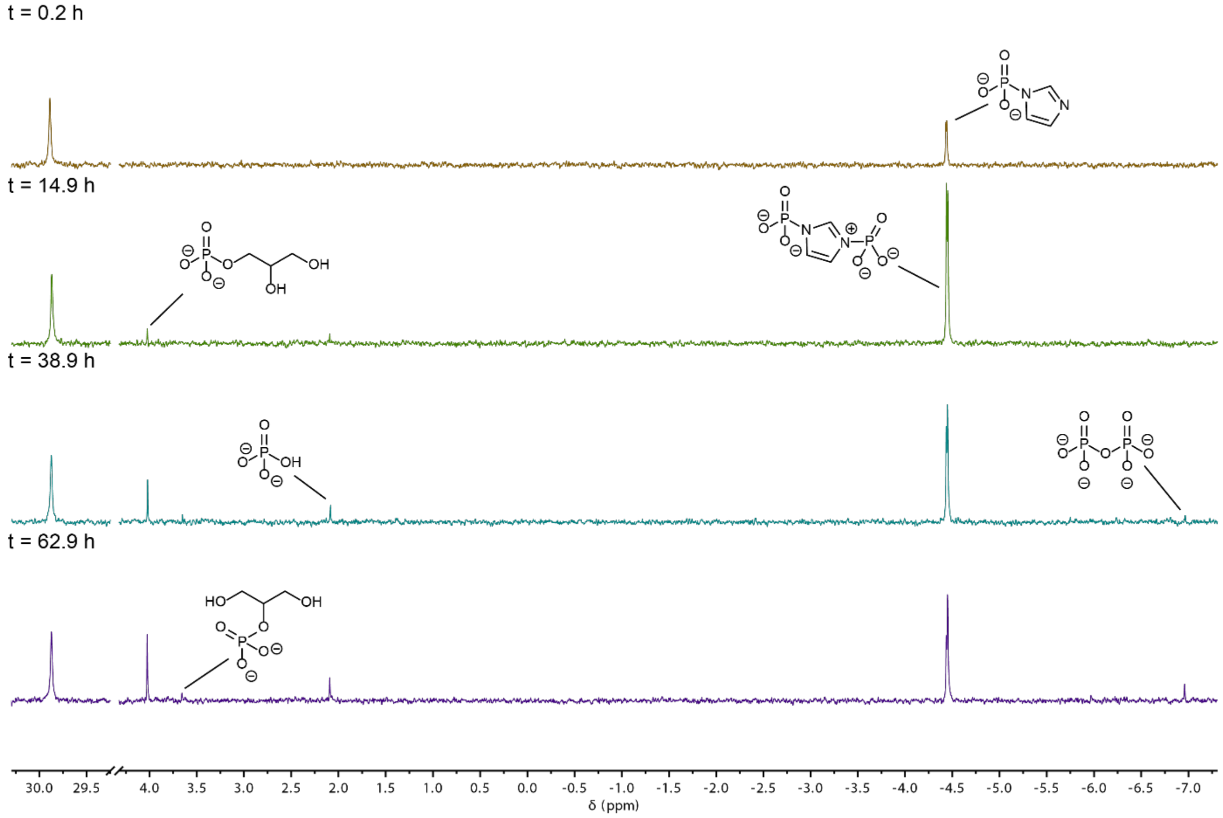

Supporting Figure 75: Representative ^{31}P-NMR spectra over time for the reaction of 0.13 mmol of calcium imidazole phosphate and 3.25 mmol of glycerol at pH 7.5 and 22 °C.

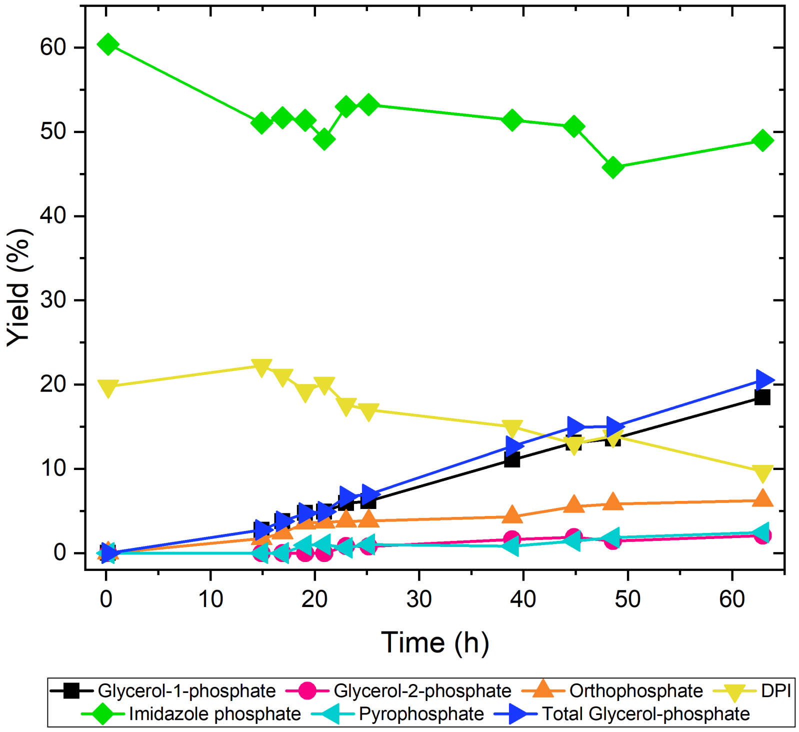

Supporting Figure 76: Changes in yield over time for the reaction of 0.13 mmol of calcium imidazole phosphate and 3.25 mmol of glycerol at pH 7.5 and 22 °C. DPI = Diphosphoimidazole. The first time point includes phosphorylation that took place in the freezer at -20 °C and thus the yields of imidazole phosphate and diphosphoimidazole are for this time point off.

S3.8.2 Experiment 2 - 3.25 mmol glycerol + 0.13 mmol imidazole phosphate

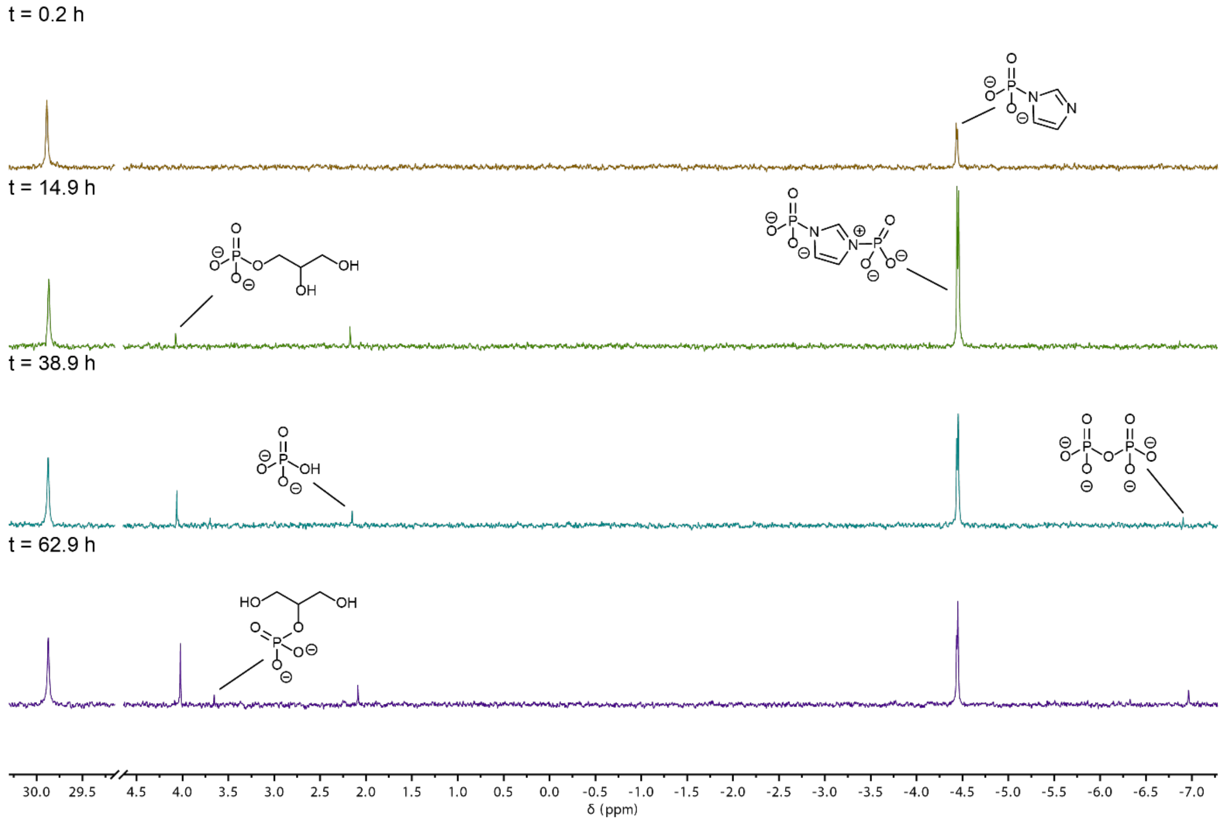

Supporting Figure 77: Representative ^{31}P-NMR spectra over time for the reaction of 0.13 mmol of calcium imidazole phosphate and 3.25 mmol of glycerol at pH 7.5 and 22 °C.

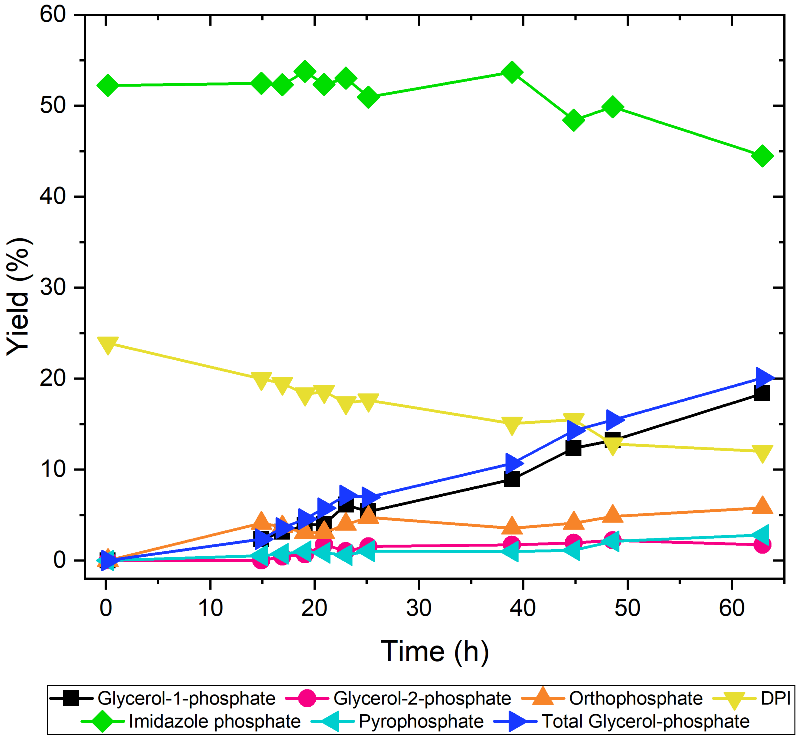

Supporting Figure 78: Changes in yield over time for the reaction of 0.13 mmol of calcium imidazole phosphate and 3.25 mmol of glycerol at pH 7.5 and 22 °C. DPI = Diphosphoimidazole. The first time point includes phosphorylation that took place in the freezer at -20 °C and thus the yields of imidazole phosphate and diphosphoimidazole are for this time point off.

S3.8.3 Experiment 3 - 3.25 mmol glycerol + 0.13 mmol imidazole phosphate

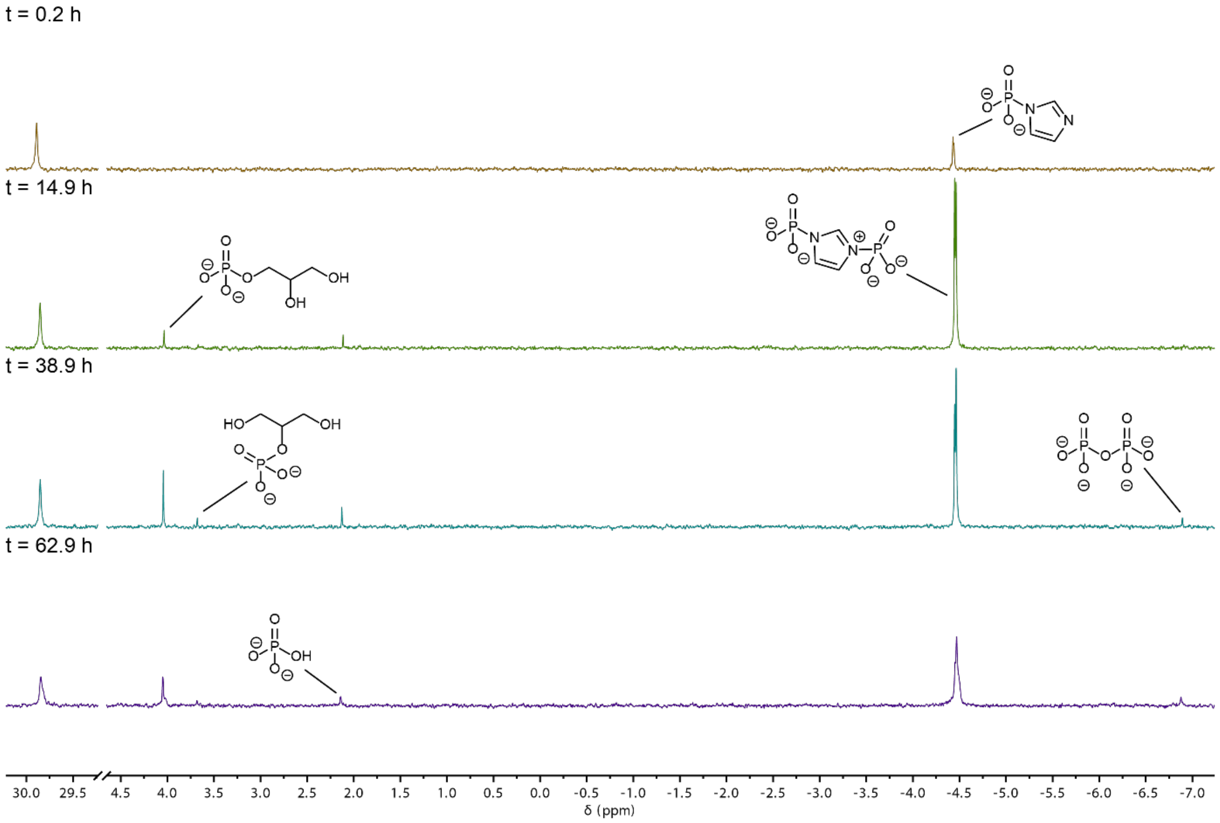

Supporting Figure 79: Representative ^{31}P-NMR spectra over time for the reaction of 0.13 mmol of calcium imidazole phosphate and 3.25 mmol of glycerol at pH 7.5 and 22 °C.

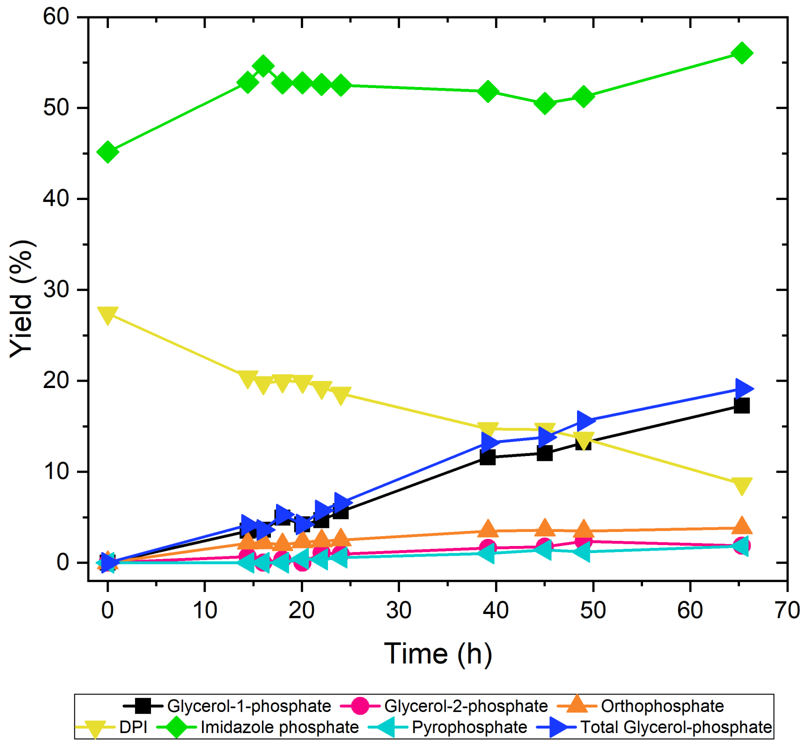

Supporting Figure 80: Changes in yield over time for the reaction of 0.13 mmol of calcium imidazole phosphate and 3.25 mmol of glycerol at pH 7.5 and 22 °C. DPI = Diphosphoimidazole. The first time point includes phosphorylation that took place in the freezer at -20 °C and thus the yields of imidazole phosphate and diphosphoimidazole are for this time point off.

S3.8.4 Combined results for 3.25 mmol glycerol + 0.13 mmol imidazole phosphate

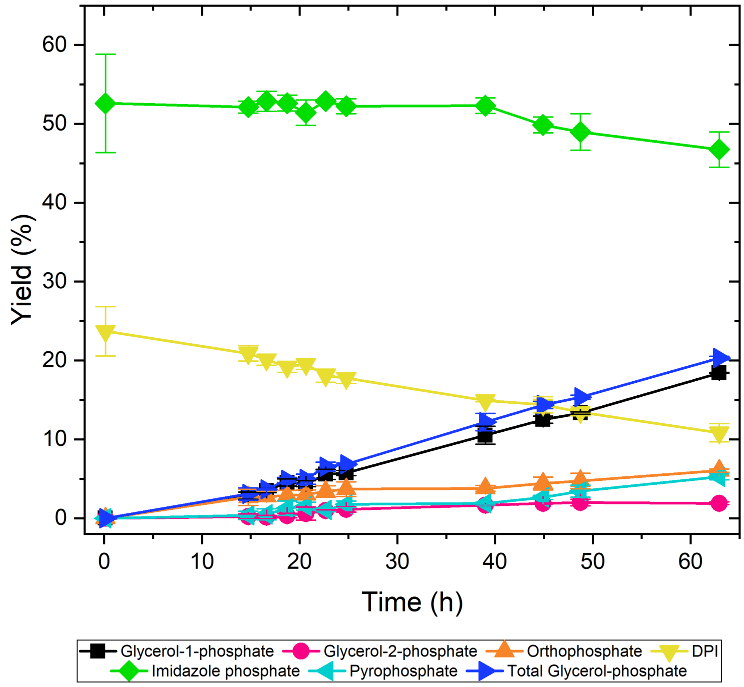

Supporting Figure 81: Changes in yield over time for the reaction of 0.13 mmol of calcium imidazole phosphate, 3.25 mmol of glycerol at pH 7.5 and 22 °C. DPI = Diphosphoimidazole. These data are the mean values and standard deviation based upon triplicate experiments. The first time point includes phosphorylation that took place in the freezer at -20 °C and thus the yields of imidazole phosphate and diphosphoimidazole are for this time point off.

S3.8.5 *Comparison between samples measured immediately and samples stored at -20 °C for 1 week for 3.25 mmol glycerol + 0.13 mmol imidazole phosphate*

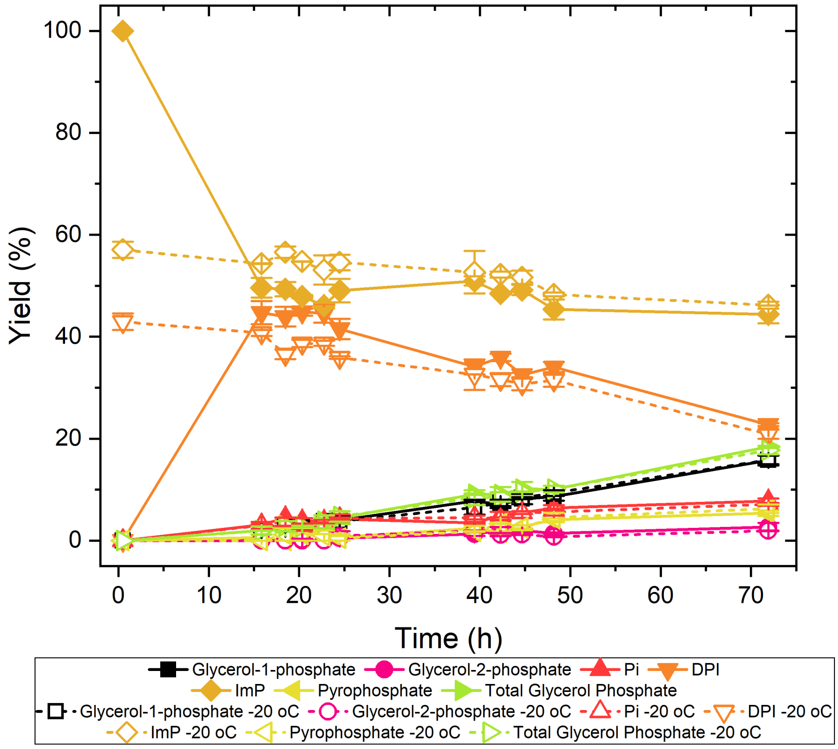

Supporting Figure 82: The changes in yield over time for the reaction of 0.13 mmol of calcium imidazole phosphate and 3.25 mmol of glycerol at pH 7.5 and 22 °C. Solid lines are for the samples measured immediately and dashed lines are for samples measured after being stored for 1 week at -20 °C. These data are the mean values and standard deviation based upon duplicate experiments.

S3.9 Phosphorylation of glycerol by imidazole phosphate with His-Gly catalyst

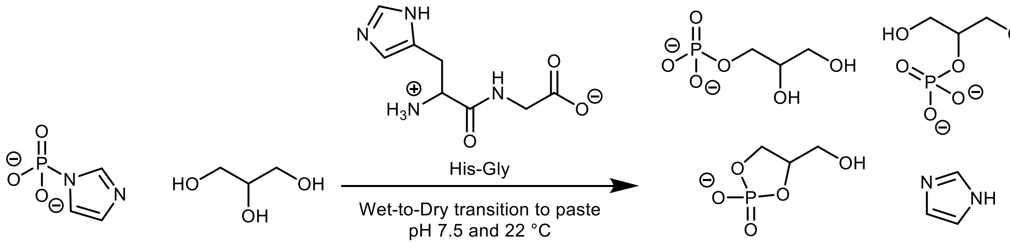

The experiment was carried out according to the procedure in S3.1 but with the 65 mM of histidine replaced by 65 mM His-Gly (27.6 mg, 0.13 mmol). The experiment was repeated in triplicate. Supporting Figures 83, 85 and 87 depict representative ^{31}P NMR spectra for the reaction over time. The changes in yield over time for all phosphate containing species are shown in Supporting Figures 84, 86 and 88. The mean experimental results with the standard deviation of each experimental data point from the triplicate experiments are shown in Supporting Figure 89.

S3.9.1 Experiment 1 - 3.25 mmol glycerol + 0.13 mmol imidazole phosphate + 0.13 mmol His-Gly

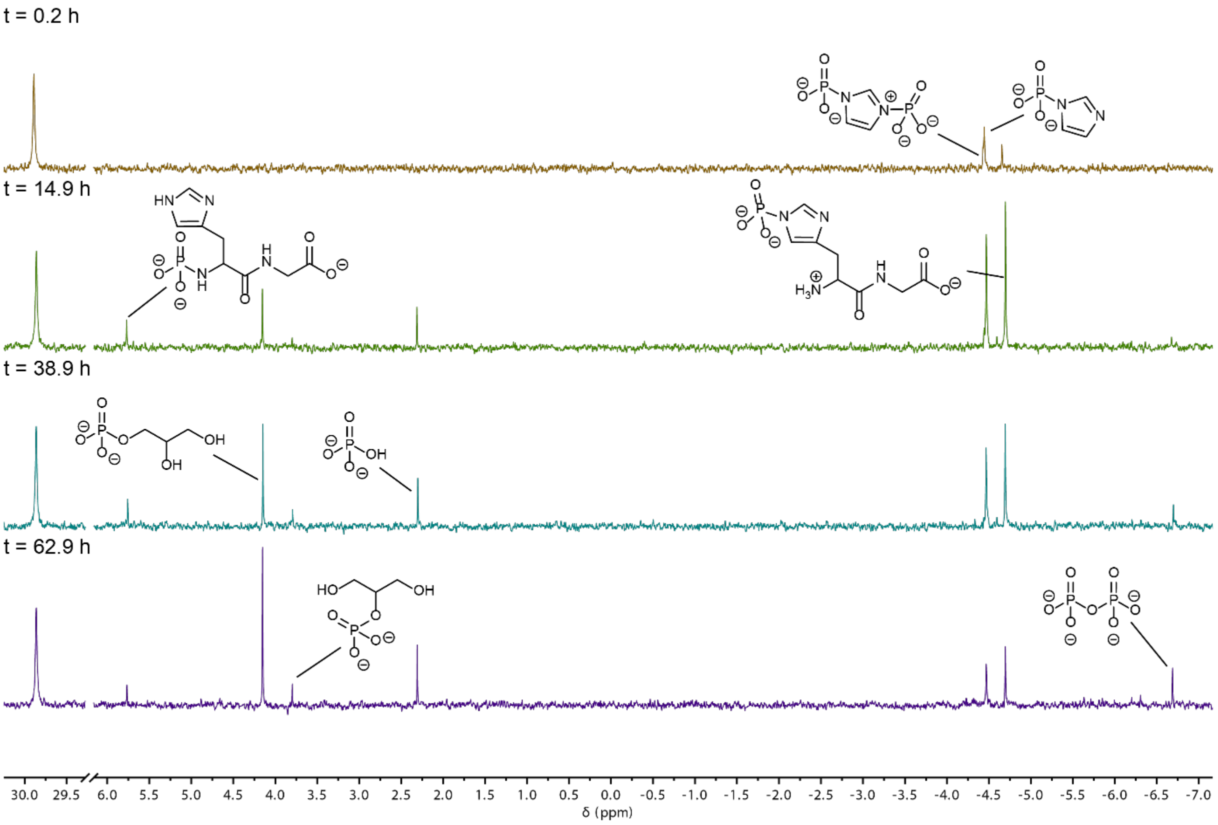

Supporting Figure 83: Representative ^{31}P-NMR spectra over time for the reaction of 0.13 mmol of calcium imidazole phosphate, 3.25 mmol of glycerol and 0.13 mmol of His-Gly at pH 7.5 and 22 °C.

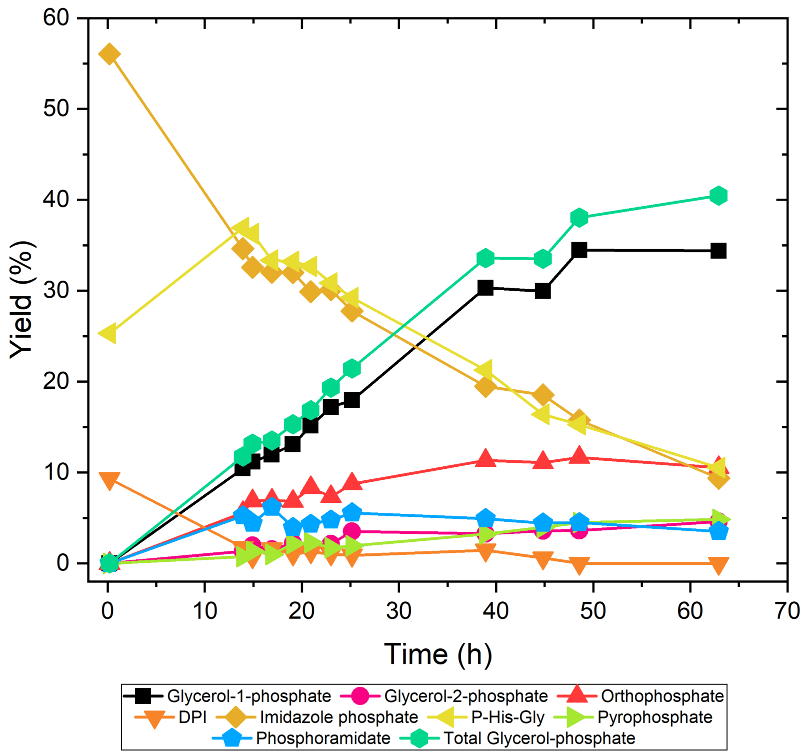

Supporting Figure 84: Changes in yield over time for the reaction of 0.13 mmol of calcium imidazole phosphate, 3.25 mmol of glycerol and 0.13 mmol of His-Gly at pH 7.5 and 22 °C. DPI = Diphosphoimidazole. The first time point includes phosphorylation that took place in the freezer at -20 °C and thus the yields of imidazole phosphate, diphosphoimidazole and P-His-Gly are for this time point off.

S3.9.2 Experiment 2 - 3.25 mmol glycerol + 0.13 mmol imidazole phosphate + 0.13 mmol His-Gly

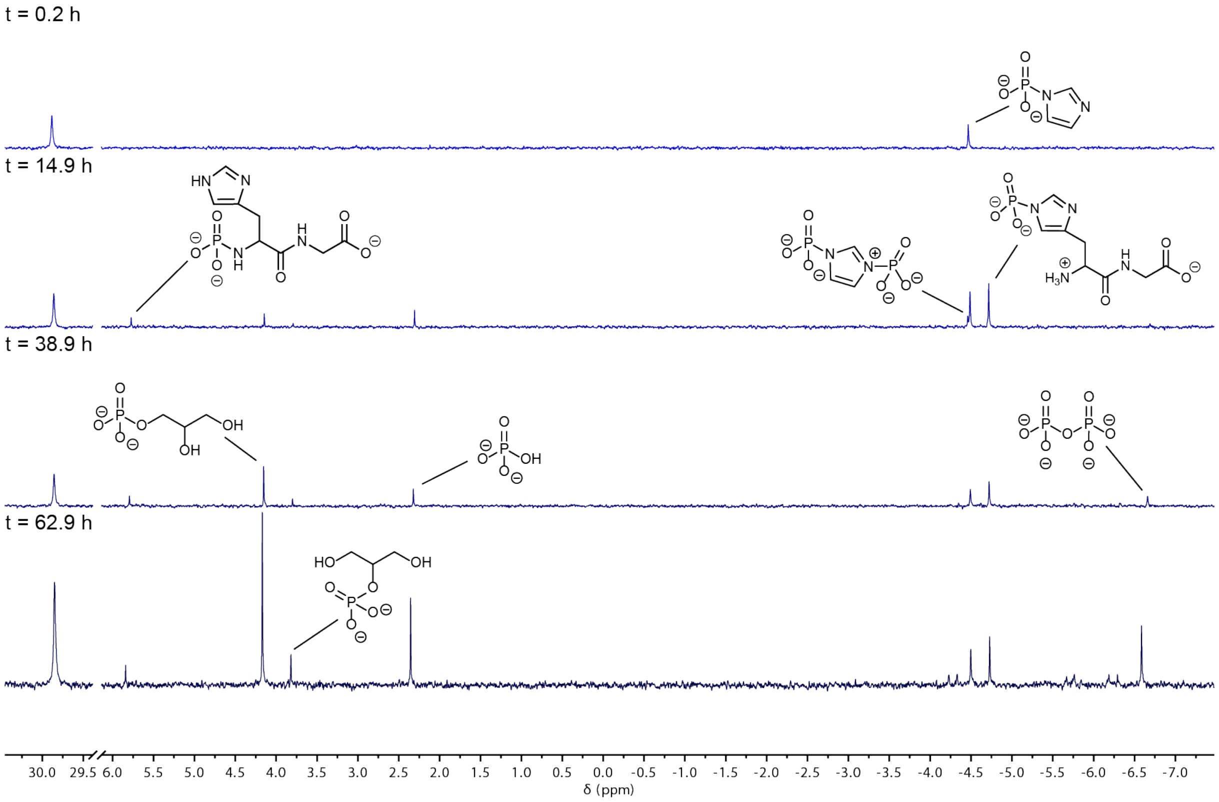

Supporting Figure 85: Representative ^{31}P-NMR spectra over time for the reaction of 0.13 mmol of calcium imidazole phosphate, 3.25 mmol of glycerol and 0.13 mmol of His-Gly at pH 7.5 and 22 °C.

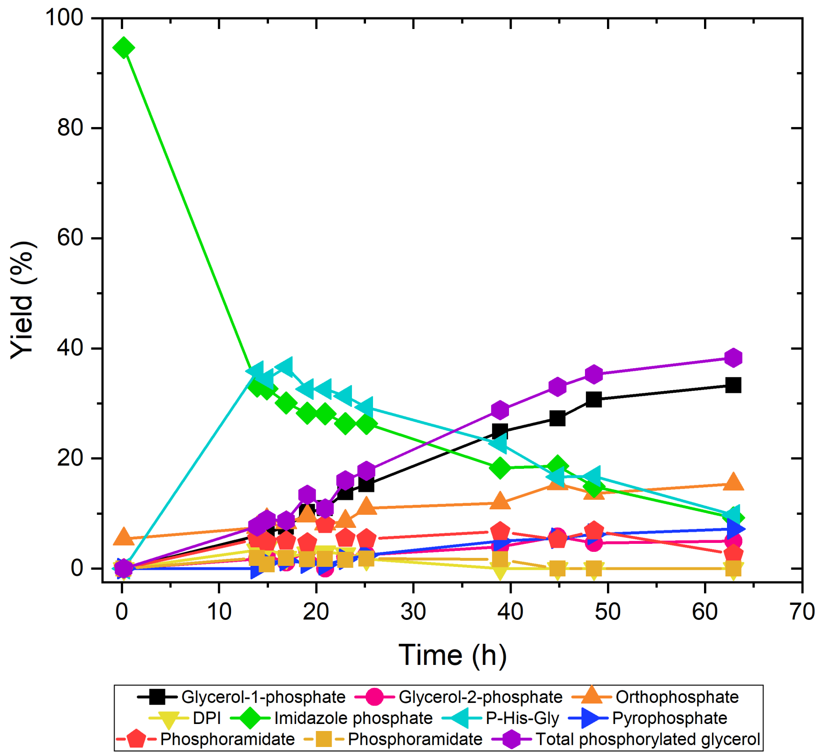

Supporting Figure 86: Changes in yield over time for the reaction of 0.13 mmol of calcium imidazole phosphate, 3.25 mmol of glycerol and 0.13 mmol of His-Gly at pH 7.5 and 22 °C. DPI = Diphosphoimidazole.

S3.9.3 *Experiment 3 - 3.25 mmol glycerol + 0.13 mmol imidazole phosphate + 0.13 mmol His-Gly*

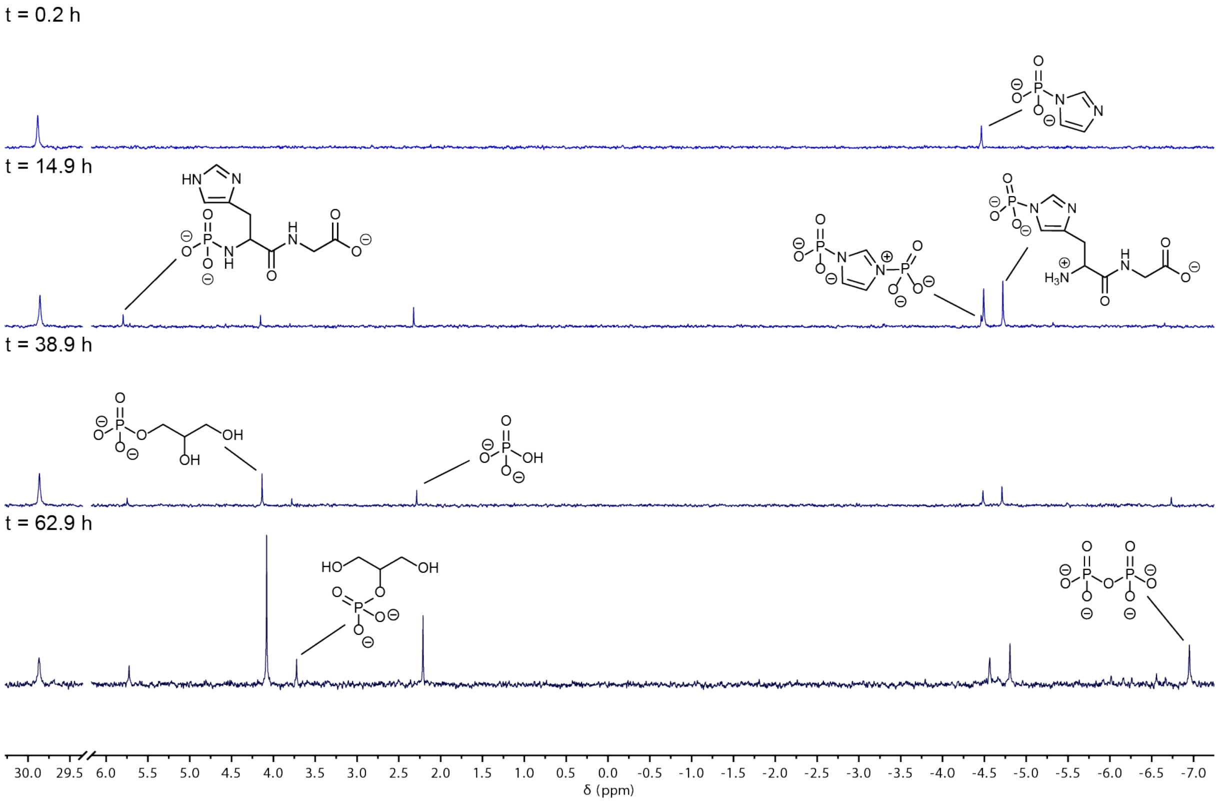

Supporting Figure 87: Representative ^{31}P-NMR spectra over time for the reaction of 0.13 mmol of calcium imidazole phosphate, 3.25 mmol of glycerol and 0.13 mmol of His-Gly at pH 7.5 and 22 °C.

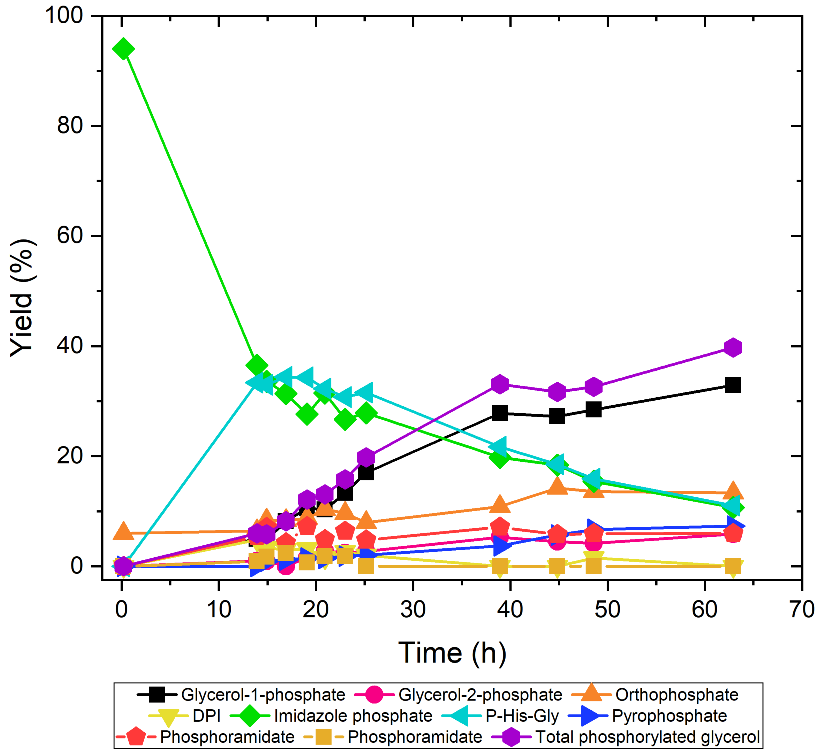

Supporting Figure 88: Changes in yield over time for the reaction of 0.13 mmol of calcium imidazole phosphate, 3.25 mmol of glycerol and 0.13 mmol of His-Gly at pH 7.5 and 22 °C. DPI = Diphosphoimidazole.

S3.9.4 Combined results for 3.25 mmol glycerol + 0.13 mmol imidazole phosphate + 0.13 mmol His-Gly

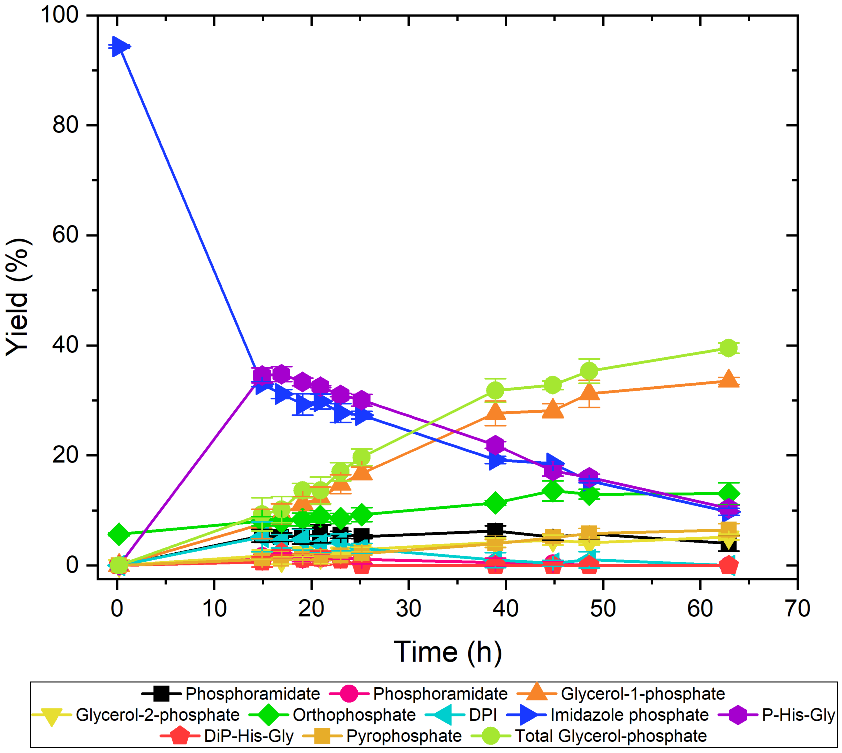

Supporting Figure 89: Changes in yield over time for the reaction of 0.13 mmol of calcium imidazole phosphate, 3.25 mmol of glycerol and 0.13 mmol of His-Gly at pH 7.5 and 22 °C. DPI = Diphosphoimidazole. These data are the mean values and standard deviation based upon triplicate experiments.

S3.9.5 Characterisation of phosphorylated His-Gly intermediate

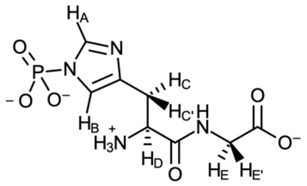

The phosphorylated His-Gly intermediate was characterised *in situ*. A solution of 50 mM His-Gly and 50 mM calcium imidazole phosphate in 0.5 mL 0.5 M MOPS buffer at pH 7.5 in 9 : 1 H_2O : D_2O containing 0.1 M citric acid and 50 mM HMPA internal standard was prepared according to the procedure in Section S2.2 and S2.4.

^{31}P NMR (202.46 MHz, 0.5 M MOPS + 0.1 M Citric Acid in 9 : 1 H_2O : D_2O at pH 7.5 and 22 °C): *phosphorylated His-Gly intermediate* δ (ppm) = - 4.88 (s, 1P). ^{1}H NMR (500.13 MHz, 0.5 M MOPS + 0.1 M Citric Acid in 9 : 1 H_2O : D_2O at pH 7.5 and 22 °C): *phosphorylated His-Gly intermediate* δ (ppm) = 7.92 (s, 1H, H_A), 7.13 (s, 1H, H_B), 3.98 (dd, 1H, H_D), 3.70 (s, 1H, H_E/$H_{E'}$), 3.65 (s, 1H, $H_{E'}$/H_E), 3.03 (1H, H_C), 2.98 (1H, $H_{C'}$). ^{13}C NMR (125.77 MHz, 0.5 M MOPS + 0.1 M Citric Acid in 9 : 1 H_2O : D_2O at pH 7.5 and 22 °C): *phosphorylated His-Gly intermediate* δ (ppm) = 176.5 (s, 1C, COOH), 17.2.2 (s, 1C, Amide N-C=O), 138.0 (s, 1C, imid C-H_A), 132.1 (s, 1C, imid), 119.2 (s, 1C, imid C-H_B), 53.7 (s, 1C, C-H_D), 43.4 (s, 1C, C-H_E), 29.7 (s, 1C, C-$H_{C+C'}$).

S3.10 Phosphorylation of glycerol by imidazole phosphate with c(His-Gly) catalyst

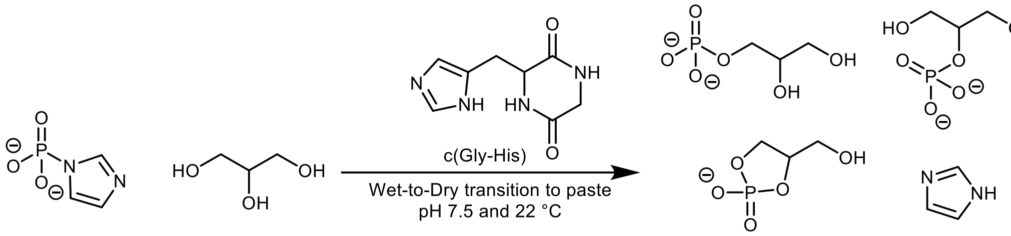

The experiment was carried out according to the procedure in S3.1 but with the 65 mM of histidine replaced by 65 mM c(His-Gly) (25.2 mg, 0.13 mmol). The experiment was repeated in triplicate. Supporting Figures 90, 92 and 94 depict representative ^{31}P NMR spectra for the reaction over time. The changes in yield over time for all phosphate containing species are shown in Supporting Figures 91, 93 and 95. The mean experimental results with the standard deviation of each experimental data point from the triplicate experiments are shown in Supporting Figure 96.

S3.10.1 Experiment 1 - 3.25 mmol glycerol + 0.13 mmol imidazole phosphate + 0.13 mmol c(His-Gly)

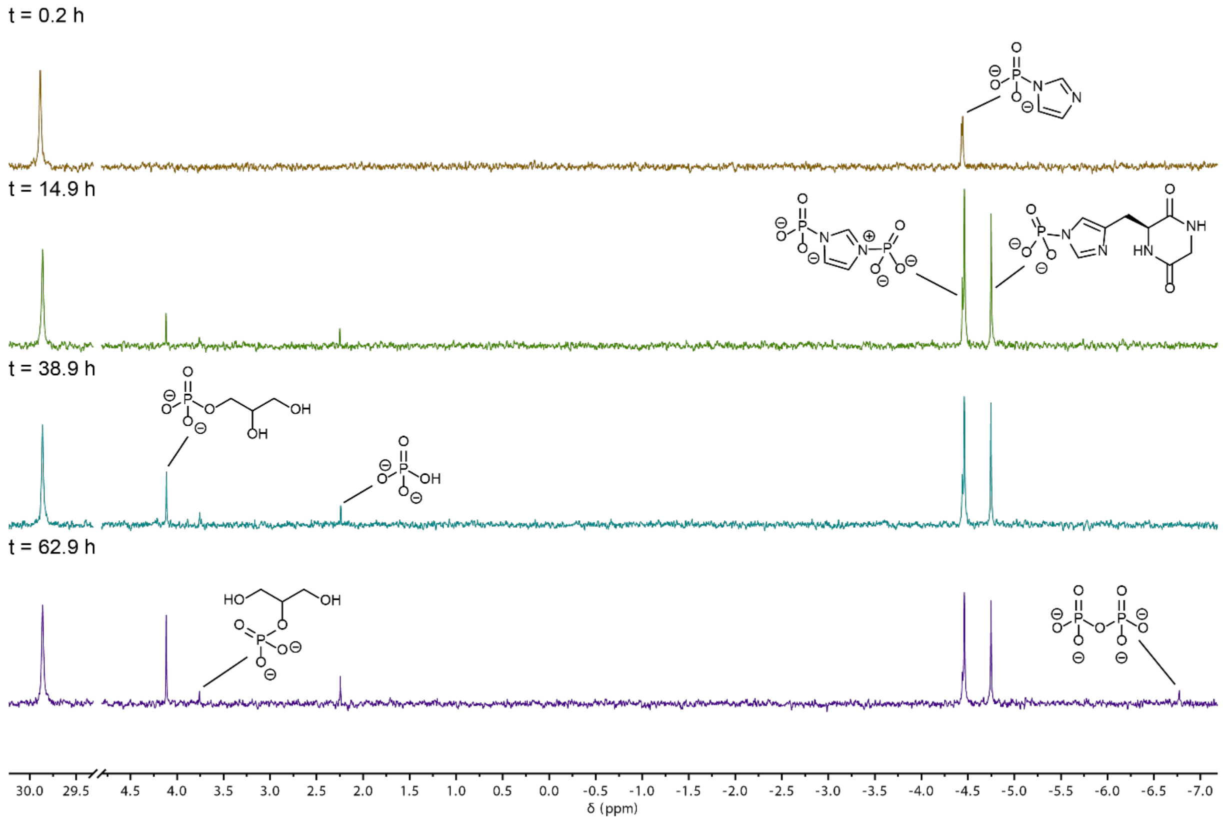

Supporting Figure 90: Representative ^{31}P-NMR spectra over time for the reaction of 0.13 mmol of calcium imidazole phosphate, 3.25 mmol of glycerol and 0.13 mmol of c(His-Gly) at pH 7.5 and 22 °C.

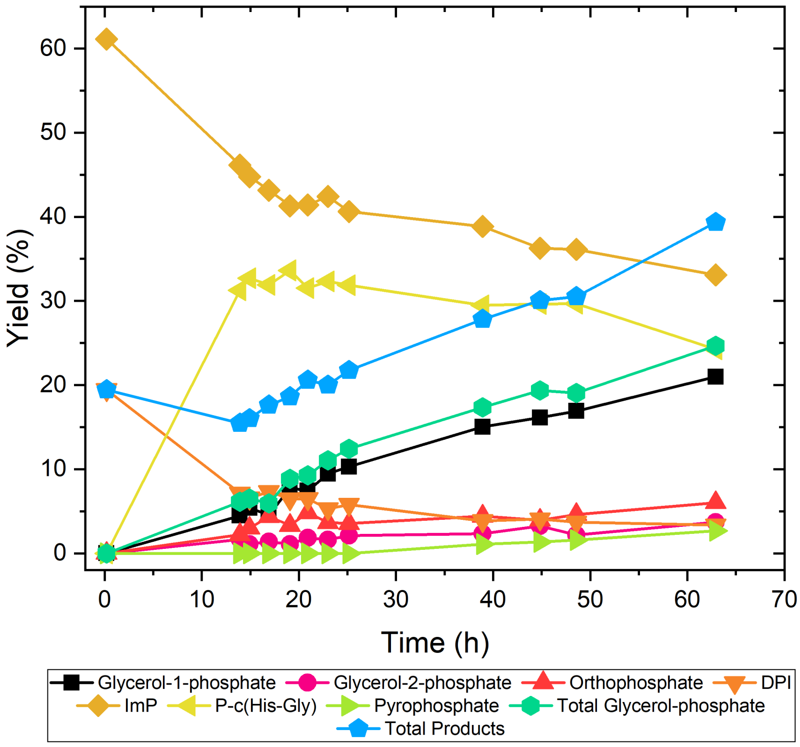

Supporting Figure 91: Changes in yield over time for the reaction of 0.13 mmol of calcium imidazole phosphate, 3.25 mmol of glycerol and 0.13 mmol of c(His-Gly) at pH 7.5 and 22 °C. DPI = Diphosphoimidazole. The first time point includes phosphorylation that took place in the freezer at -20 °C and thus the yields of imidazole phosphate, diphosphoimidazole and P-c(His-Gly) are for this time point off.

S3.10.2 Experiment 2 - 3.25 mmol glycerol + 0.13 mmol imidazole phosphate + 0.13 mmol c(His-Gly)

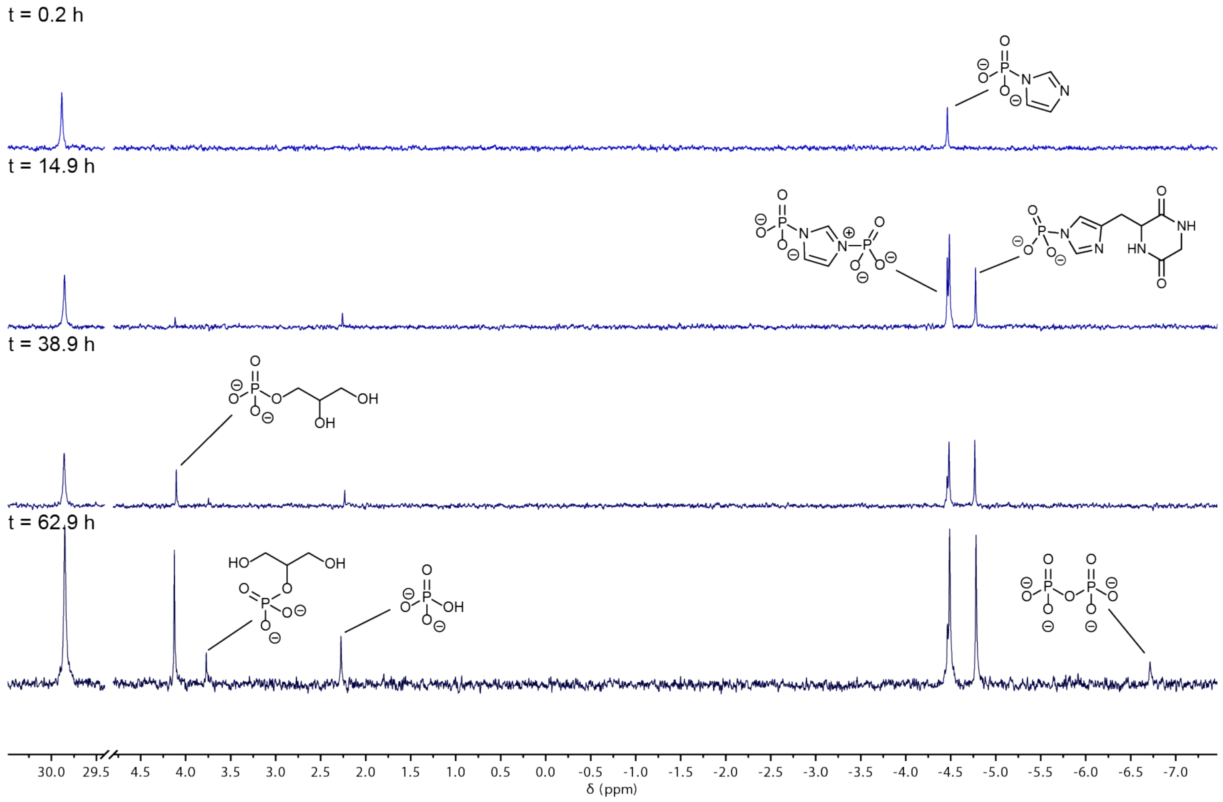

Supporting Figure 92: Representative ^{31}P-NMR spectra over time for the reaction of 0.13 mmol of calcium imidazole phosphate, 3.25 mmol of glycerol and 0.13 mmol of c(His-Gly) at pH 7.5 and 22 °C.

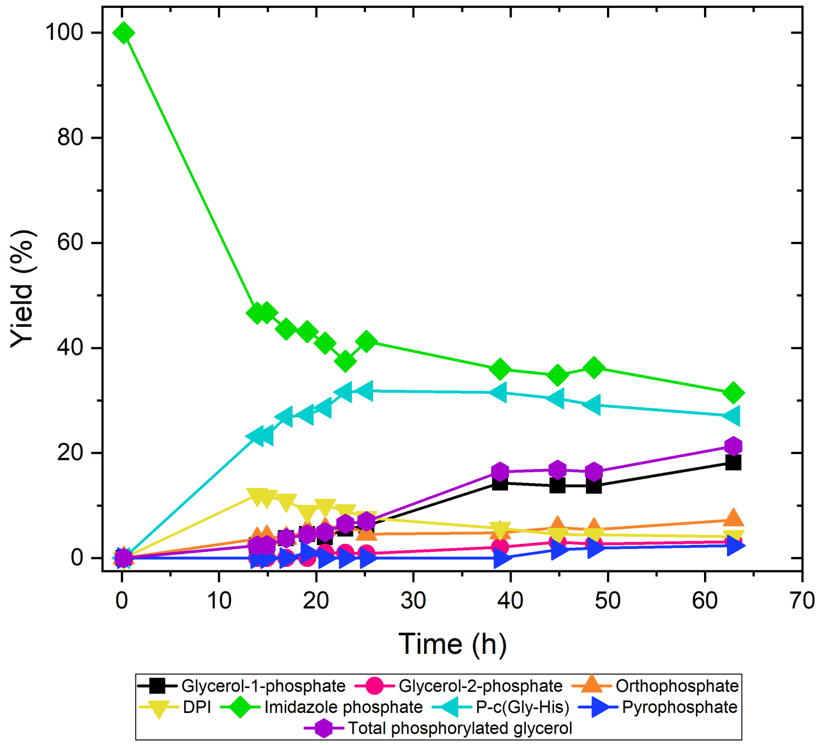

Supporting Figure 93: Changes in yield over time for the reaction of 0.13 mmol of calcium imidazole phosphate, 3.25 mmol of glycerol and 0.13 mmol of c(His-Gly) at pH 7.5 and 22 °C. DPI = Diphosphoimidazole.

S3.10.3 Experiment 3 - 3.25 mmol glycerol + 0.13 mmol imidazole phosphate + 0.13 mmol c(His-Gly)

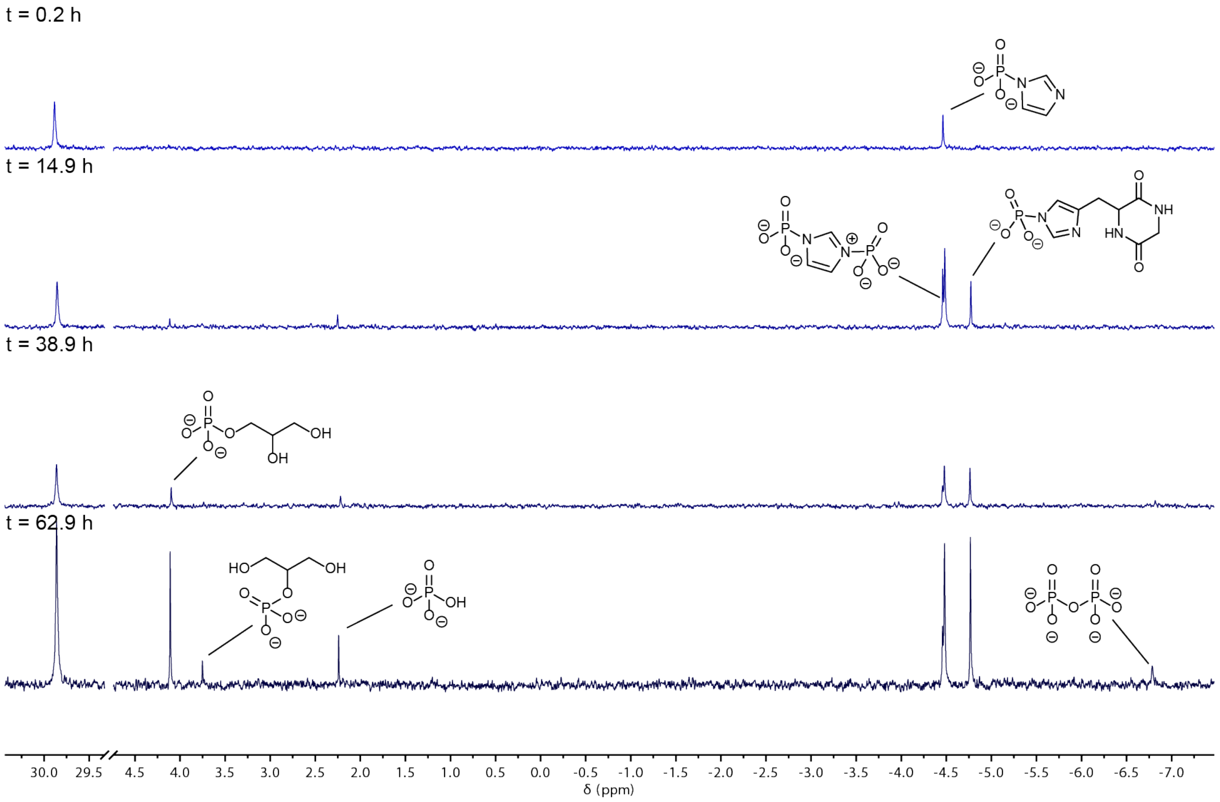

Supporting Figure 94: Representative ^{31}P-NMR spectra over time for the reaction of 0.13 mmol of calcium imidazole phosphate, 3.25 mmol of glycerol and 0.13 mmol of c(His-Gly)at pH 7.5 and 22 °C.

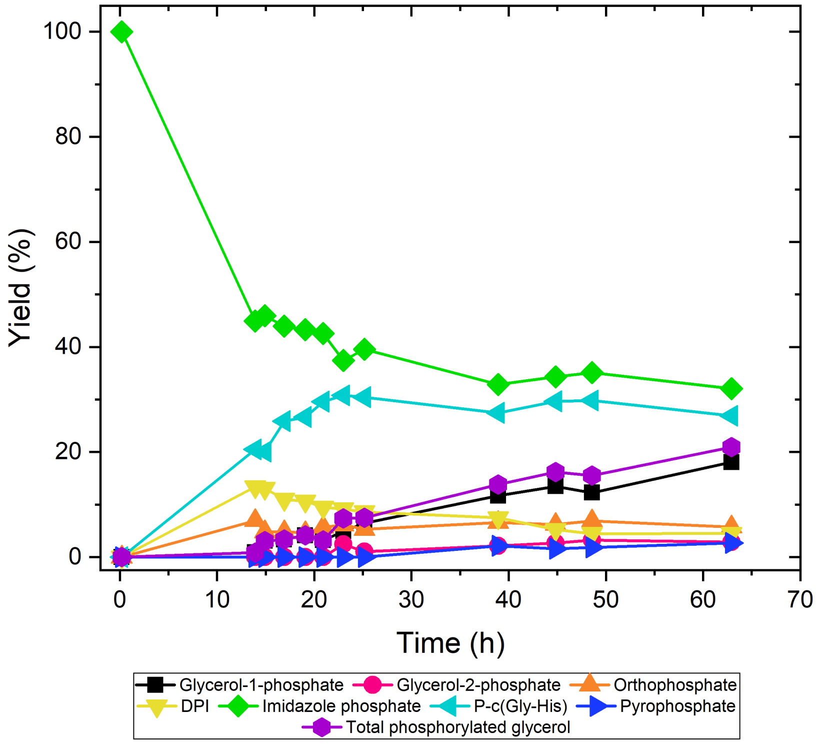

Supporting Figure 95: Changes in yield over time for the reaction of 0.13 mmol of calcium imidazole phosphate, 3.25 mmol of glycerol and 0.13 mmol of c(His-Gly)at pH 7.5 and 22 °C. DPI = Diphosphoimidazole.

S3.10.4 Combined results for 3.25 mmol glycerol + 0.13 mmol imidazole phosphate + 0.13 mmol c(His-Gly)

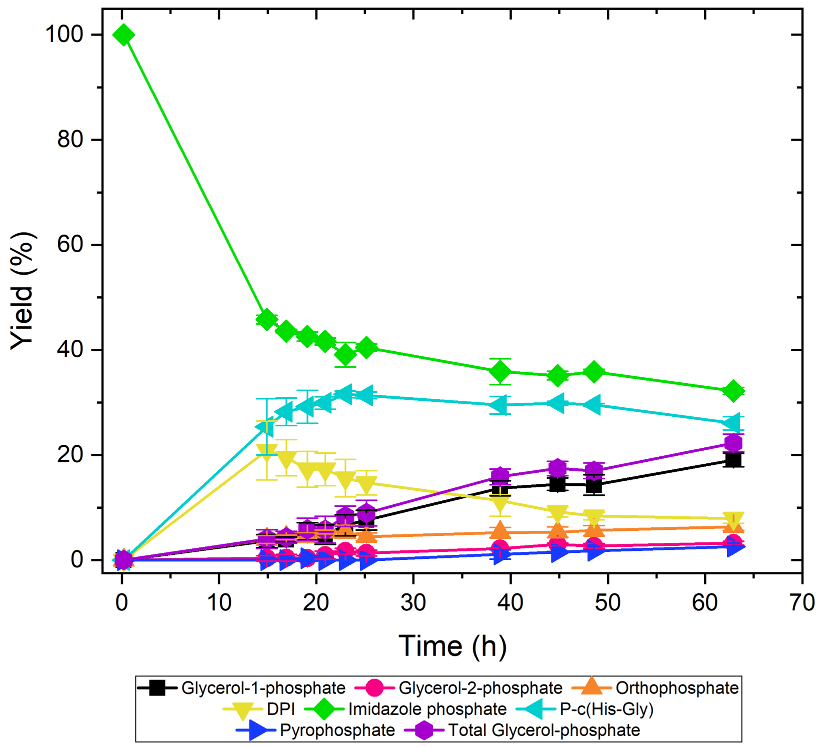

Supporting Figure 96: Changes in yield over time for the reaction of 0.13 mmol of calcium imidazole phosphate, 3.25 mmol of glycerol and 0.13 mmol of c(His-Gly) at pH 7.5 and 22 °C. DPI = Diphosphoimidazole. These data are the mean values and standard deviation based upon triplicate experiments.

S3.10.5 Characterisation of phosphorylated c(His-Gly) intermediate

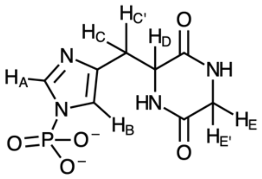

The phosphorylated c(His-Gly) intermediate was characterised *in situ*. A solution of 50 mM c(His-Gly) and 50 mM calcium imidazole phosphate in 0.5 mL 0.5 M MOPS buffer at pH 7.5 in 9 : 1 H_2O : D_2O containing 0.1 M citric acid and 50 mM HMPA internal standard was prepared with the procedure in Section S2.2 and S2.4.

^{31}P NMR (202.46 MHz, 0.5 M MOPS + 0.1 M Citric Acid in 9 : 1 H_2O : D_2O at pH 7.5 and 22 °C): *phosphorylated c(His-Gly) intermediate* δ (ppm) = - 4.96 (s, 1P). ^{1}H NMR (500.13 MHz, 0.5 M MOPS + 0.1 M Citric Acid in 9 : 1 H_2O : D_2O at pH 7.5 and 22 °C): *phosphorylated c(His-Gly) intermediate* δ (ppm) = 7.80 (s, 1H, H_A), 7.02 (s, 1H, H_B), 4.27 (dd, 1H, H_D), 3.68 (s, 1H, H_E/$H_{E'}$), 3.65 (s, 1H, $H_{E'}$/H_E), 3.14 (1H, H_C), 2.99 (1H, $H_{C'}$). ^{13}C NMR (125.77 MHz, 0.5 M MOPS + 0.1 M Citric Acid in 9 : 1 H_2O : D_2O at pH 7.5 and 22 °C): *phosphorylated c(His-Gly) intermediate* δ (ppm) = 169.5 (s, 1C, Amide N-C=O), 168.5 (s, 1C, Amide N-C=O), 138.2 (s, 1C, imid C-H_A), 133.0 (s, 1C, imid), 119.1 (s, 1C, imid C-H_B), 55.3 (s, 1C, C-H_D), 43.6 (s, 1C, C-H_E), 31.2 (s, 1C, C-$H_{C+C'}$).

S3.11 Phosphorylation of glycerol by imidazole phosphate with Ala-His-Lys catalyst

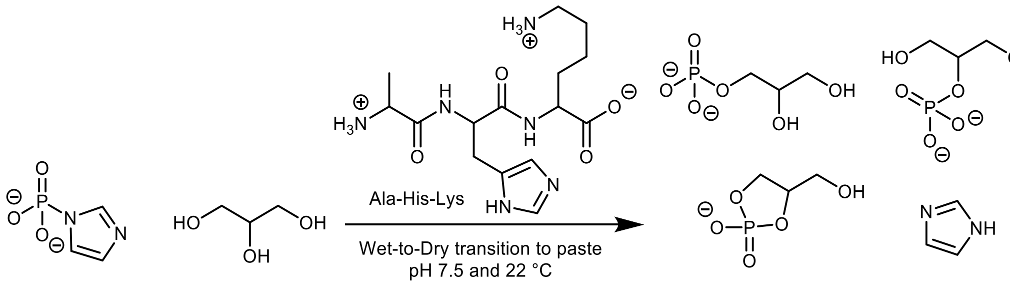

The experiment was carried out according to the procedure in S3.1 but with the 65 mM of histidine replaced by 65 mM Ala-His-Lys acetate salt (46.1 mg, 0.13 mmol). The experiment was repeated in triplicate. Supporting Figures 97, 99 and 101 depict representative ^{31}P NMR spectra for the reaction over time. The changes in yield over time for all phosphate containing species are shown in Supporting Figures 98, 100 and 102. The mean experimental results with the standard deviation of each experimental data point from the triplicate experiments are shown in Supporting Figure 103.

S3.11.1 Experiment 1 - 3.25 mmol glycerol + 0.13 mmol imidazole phosphate + 0.13 mmol Ala-His-Lys

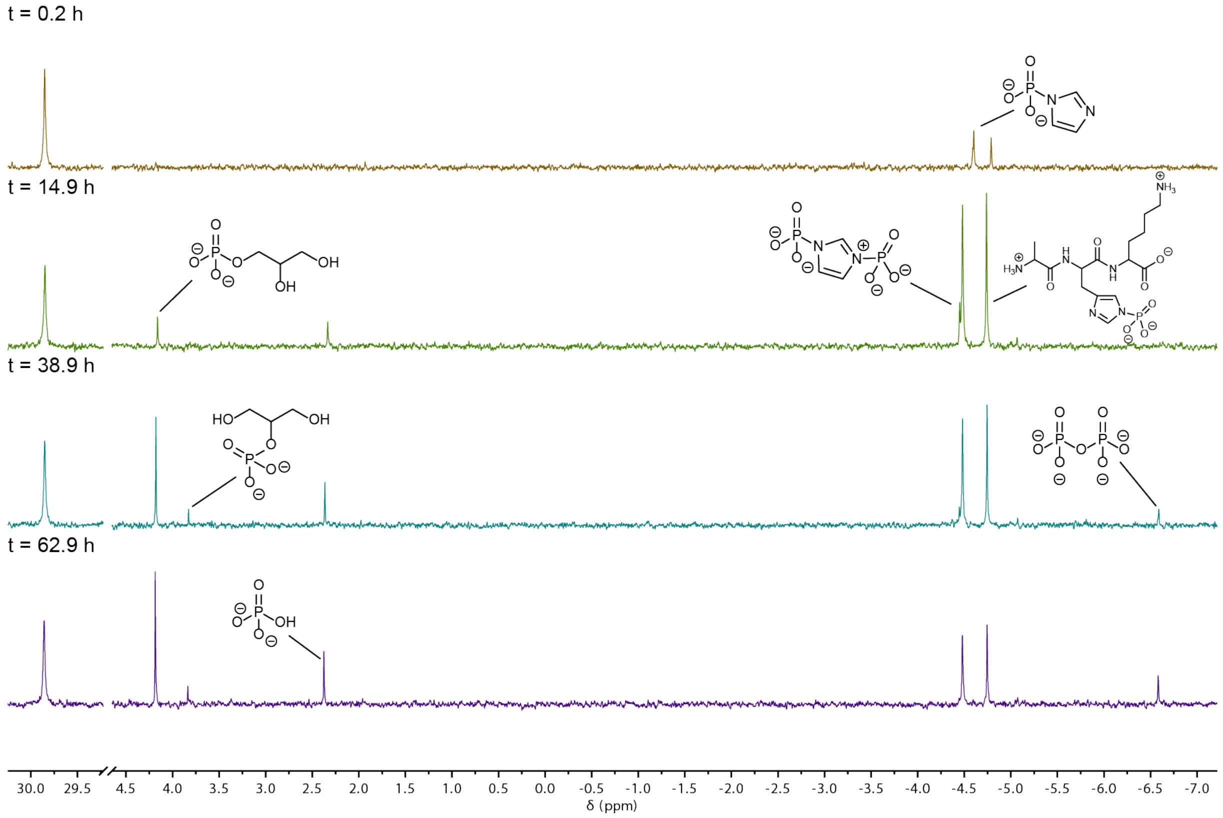

Supporting Figure 97: Representative ^{31}P-NMR spectra over time for the reaction of 0.13 mmol of calcium imidazole phosphate, 3.25 mmol of glycerol and 0.13 mmol of Ala-His-Lys at pH 7.5 and 22 °C.

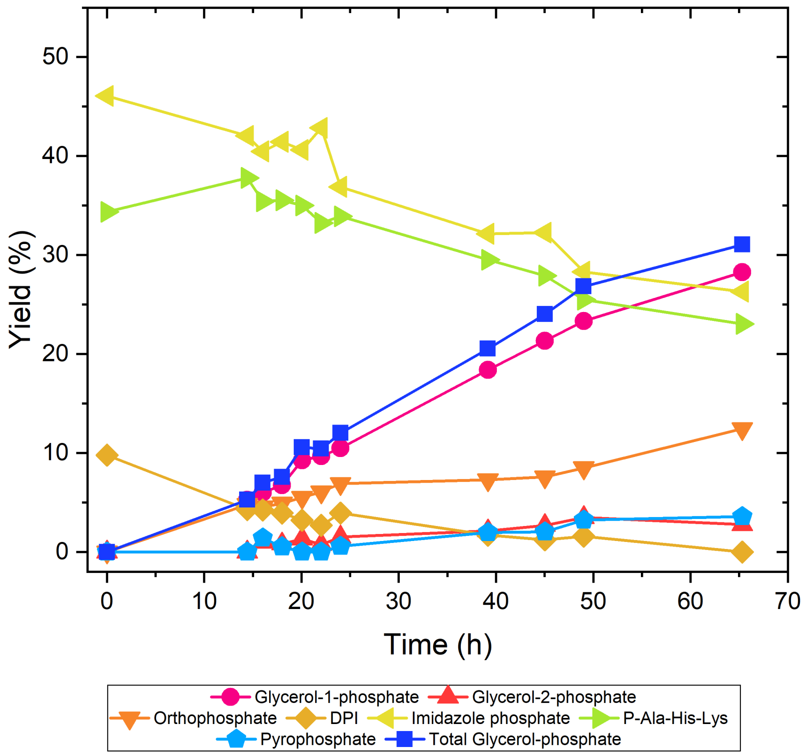

Supporting Figure 98: Changes in yield over time for the reaction of 0.13 mmol calcium imidazole phosphate, 3.25 mmol glycerol and 0.13 mmol Ala-His-Lys at pH 7.5 and 22 °C. DPI = Diphosphoimidazole. The first time point includes phosphorylation that took place in the freezer at -20 °C and thus the yields of imidazole phosphate, diphosphoimidazole and P-Ala-His-Lys are for this time point off.

S3.11.2 Experiment 2 - 3.25 mmol glycerol + 0.13 mmol imidazole phosphate + 0.13 mmol Ala-His-Lys

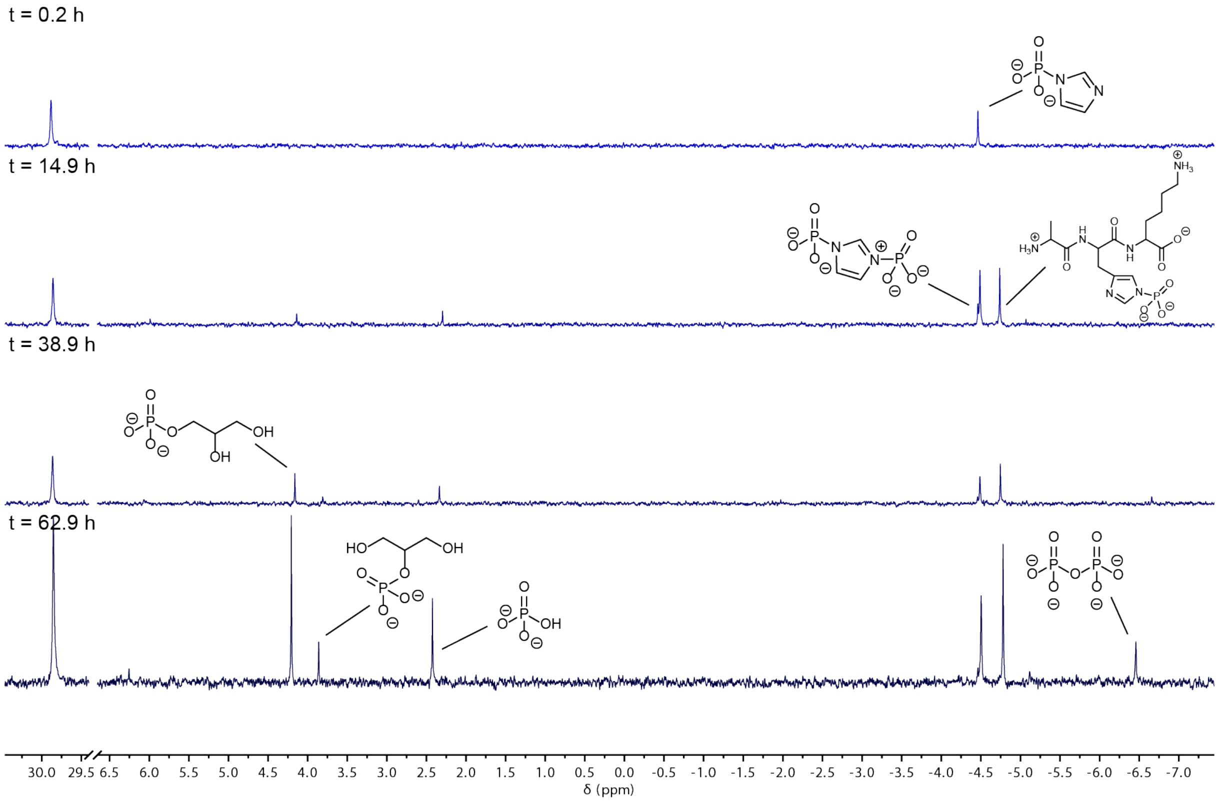

Supporting Figure 99: Representative ^{31}P-NMR spectra over time for the reaction of 0.13 mmol of calcium imidazole phosphate, 3.25 mmol of glycerol and 0.13 mmol of Ala-His-Lys at pH 7.5 and 22 °C.

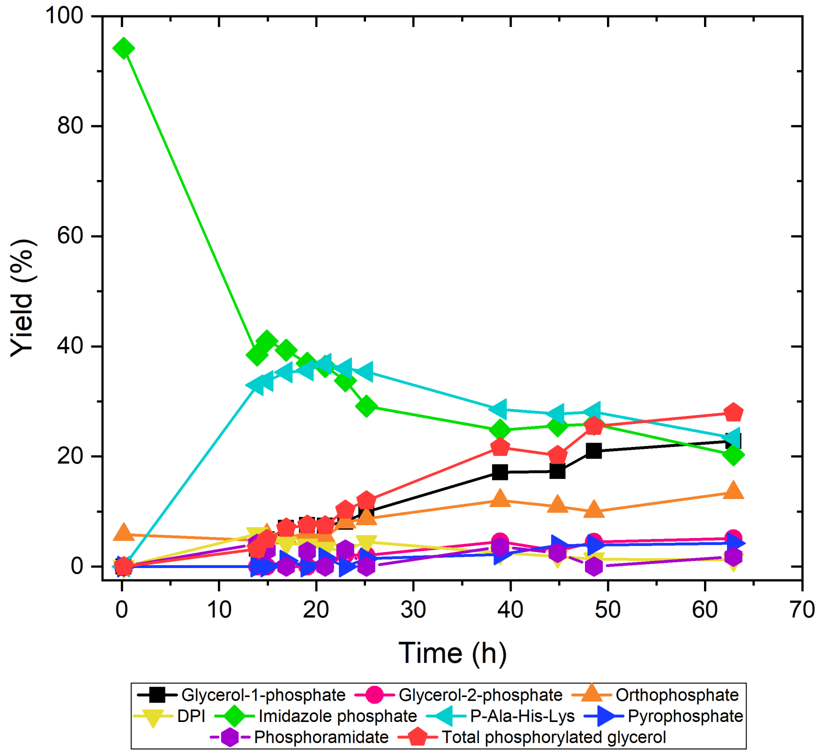

Supporting Figure 100: Changes in yield over time for the reaction of 0.13 mmol calcium imidazole phosphate, 3.25 mmol glycerol and 0.13 mmol Ala-His-Lys at pH 7.5 and 22 °C. DPI = Diphosphoimidazole.

S3.11.3 Experiment 3 - 3.25 mmol glycerol + 0.13 mmol imidazole phosphate + 0.13 mmol Ala-His-Lys

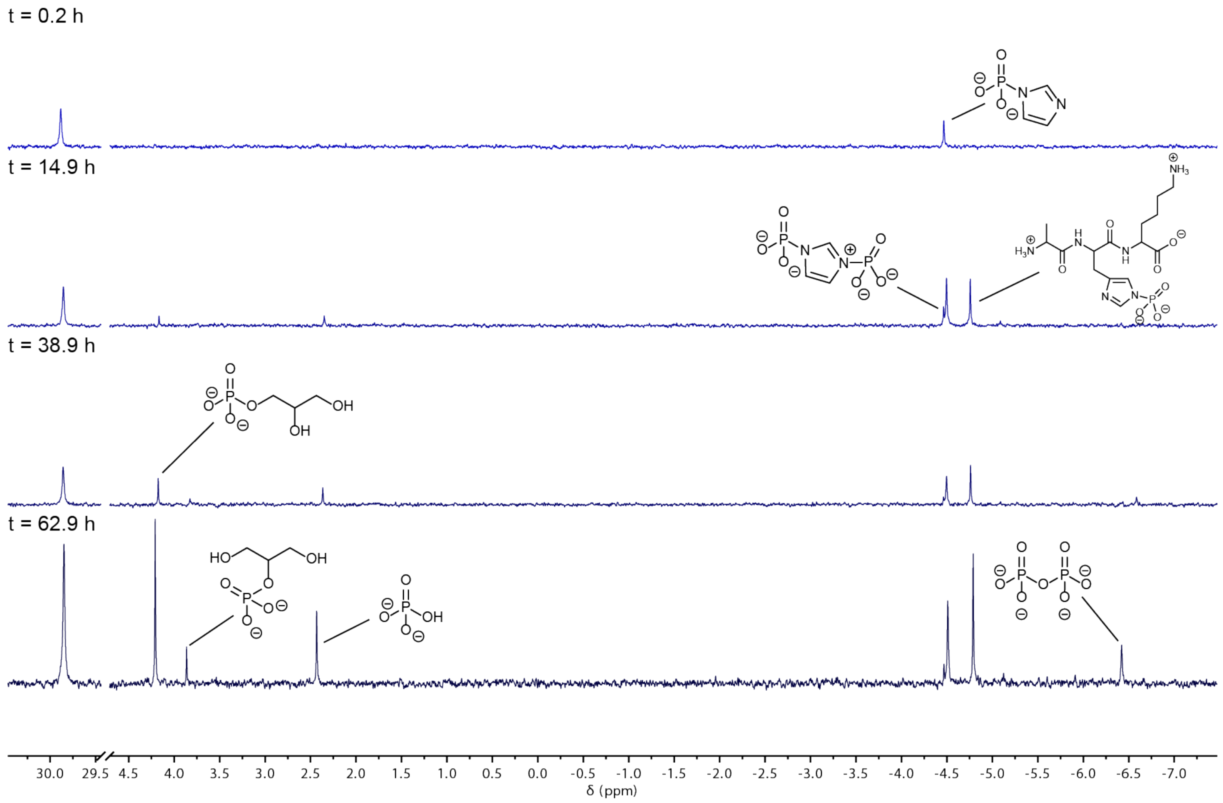

Supporting Figure 101: Representative ^{31}P-NMR spectra over time for the reaction of 0.13 mmol of calcium imidazole phosphate, 3.25 mmol of glycerol and 0.13 mmol of Ala-His-Lys at pH 7.5 and 22 °C.

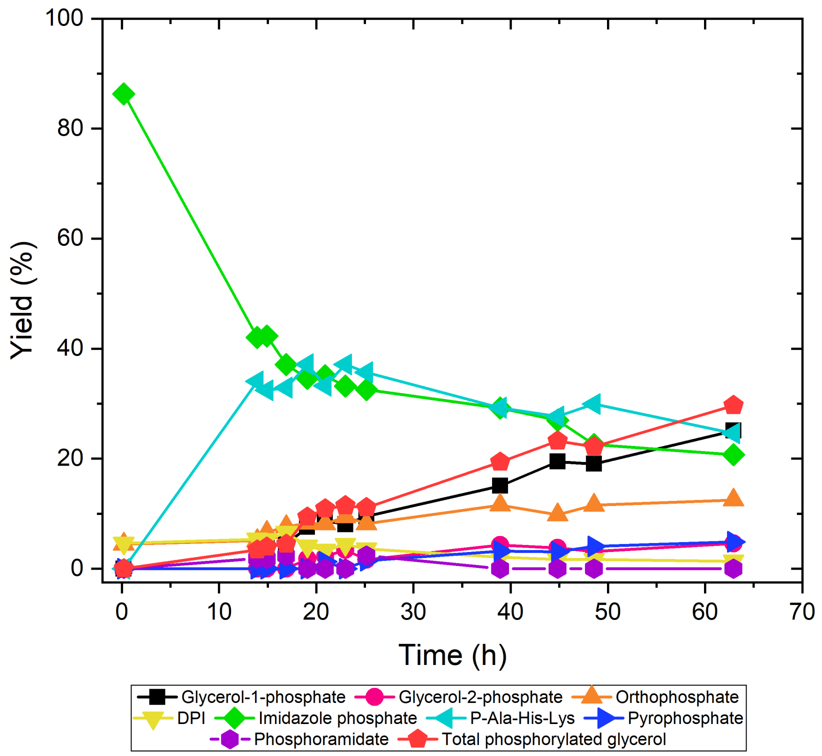

Supporting Figure 102: Changes in yield over time for the reaction of 0.13 mmol calcium imidazole phosphate, 3.25 mmol glycerol and 0.13 mmol Ala-His-Lys at pH 7.5 and 22 °C. DPI = Diphosphoimidazole.

S3.11.4 Combined results for 3.25 mmol glycerol + 0.13 mmol imidazole phosphate + 0.13 mmol Ala-His-Lys

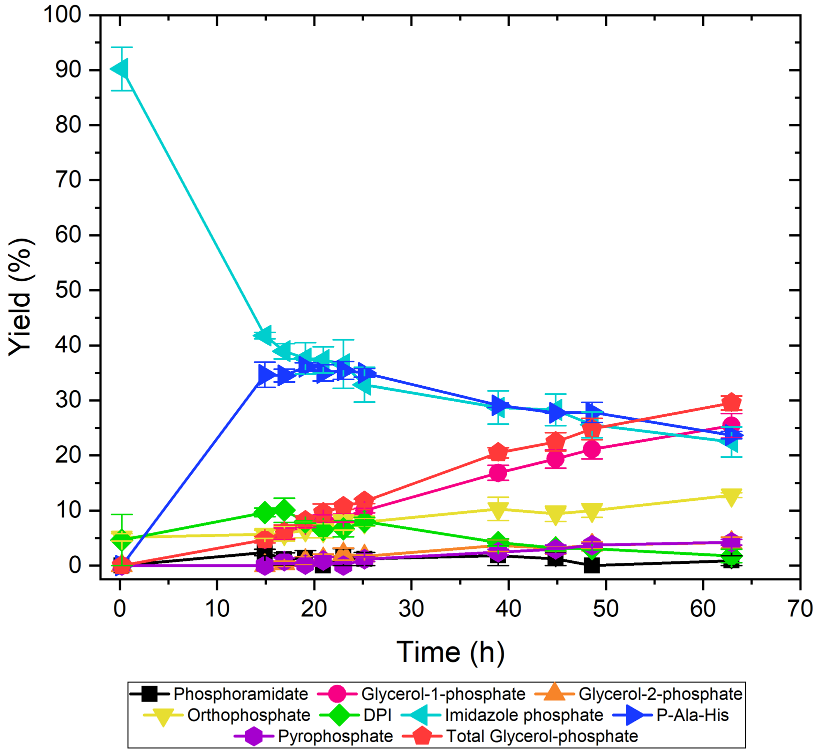

Supporting Figure 103: Changes in yield over time for the reaction of 0.13 mmol calcium imidazole phosphate, 3.25 mmol glycerol and 0.13 mmol Ala-His-Lys at pH 7.5 and 22 °C. DPI = Diphosphoimidazole. These data are the mean values and standard deviation based upon triplicate experiments.

S3.11.5 Characterisation of phosphorylated Ala-His-Lys intermediate

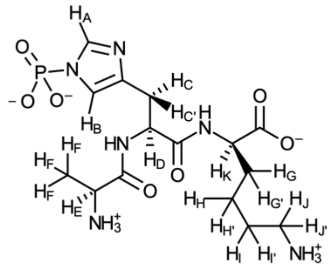

The phosphorylated Ala-His-Lys intermediate was characterised *in situ*. A solution of 50 mM Ala-His-Lys and 50 mM calcium imidazole phosphate in 0.5 mL 0.5 M MOPS buffer at pH 7.5 in 9 : 1 H_2O : D_2O containing 0.1 M citric acid and 50 mM HMPA internal standard was prepared according to the procedure in Section S2.2 and S2.4.

^{31}P NMR (202.46 MHz, 0.5 M MOPS + 0.1 M Citric Acid in 9 : 1 H_2O : D_2O at pH 7.5 and 22 °C): *phosphorylated Ala-His-Lys intermediate* δ (ppm) = - 4.92 (s, 1P). ^{1}H NMR (500.13 MHz, 0.5 M MOPS + 0.1 M Citric Acid in 9 : 1 H_2O : D_2O at pH 7.5 and 22 °C): *phosphorylated Ala-His-Lys intermediate* δ (ppm) = 7.95 (s, 1H, H_A), 7.08 (s, 1H, H_B), 4.55 (1H, H_D), 4.02 (1H, H_E), 3.92 (1H, H_K), 3.04 (2H, $H_{C+C'}$), 2.90 (s, 2H, $H_{J+J'}$), 1.68 (2H, $H_{G+G'}$), 1.57 (2H,

$H_{I+I'}$), 1.36 (d, 3H, H_F), 1.26 (2H, $H_{H+H'}$). ^{13}C NMR (125.77 MHz, 0.5 M MOPS + 0.1 M Citric Acid in 9 : 1 H_2O : D_2O at pH 7.5 and 22 °C): *phosphorylated Ala-His-Lys intermediate* δ (ppm) = 178.3 (s, 1C, COOH), 171.9 (s, 1C, Amide N-C=O), 171.5 (s, 1C, Amide N-C=O), 137.4 (s, 1C, imid C-H_A), 132.6 (s, 1C, imid), 119.0 (s, 1C, imid C-H_B), 55.1 (s, 1C, C-H_K), 53.6 (s, 1C, C-H_D), 49.2 (s, 1C, C-H_E), 39.5 (s, 1C, C-$H_{J+J'}$), 30.1 (s, 1C, C-$H_{G+G'}$), 28.1 (s, 1C, C-$H_{C+C'}$), 26.2 (s, 1C, C-$H_{I+I'}$), 21.9 (s, 1C, C-$H_{I+I'}$), 17.2 (s, 1C, C-H_F).

S3.12 Phosphorylation of glycerol by imidazole phosphate with Arg-His-NH_2 catalyst

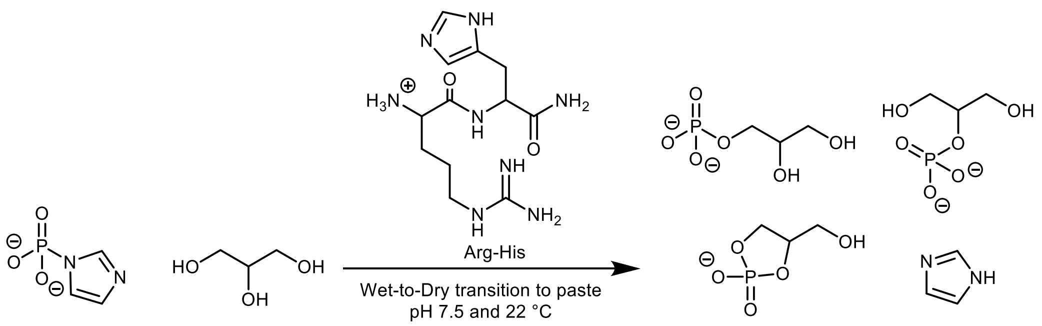

The experiment was carried out according to the procedure in S3.1 but with the 65 mM of histidine replaced by 65 mM Arg-His-NH_2 acetate salt (40.3 mg, 0.13 mmol). The experiment was repeated in triplicate. Supporting Figures 104, 106 and 108 depict representative ^{31}P NMR spectra for the reaction over time. The changes in yield over time for all phosphate containing species are shown in Supporting Figures 105, 107 and 109. The mean experimental results with the standard deviation of each experimental data point from the triplicate experiments are shown in Supporting Figure 110.

S3.12.1 *Experiment 1 - 3.25 mmol glycerol + 0.13 mmol imidazole phosphate + 0.13 mmol Arg-His-NH_2*

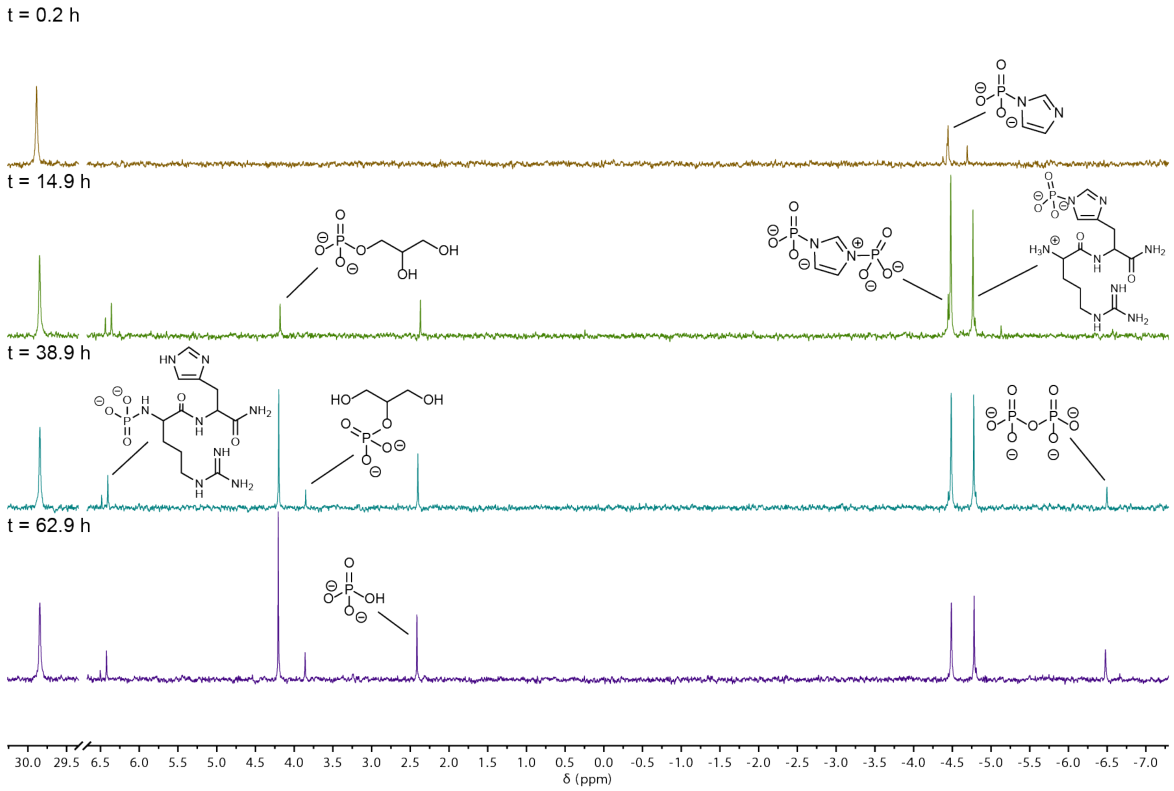

Supporting Figure 104: Representative ^{31}P-NMR spectra over time for the reaction of 0.13 mmol of calcium imidazole phosphate, 3.25 mmol of glycerol and 0.13 mmol of Arg-His-NH_2 at pH 7.5 and 22 °C.

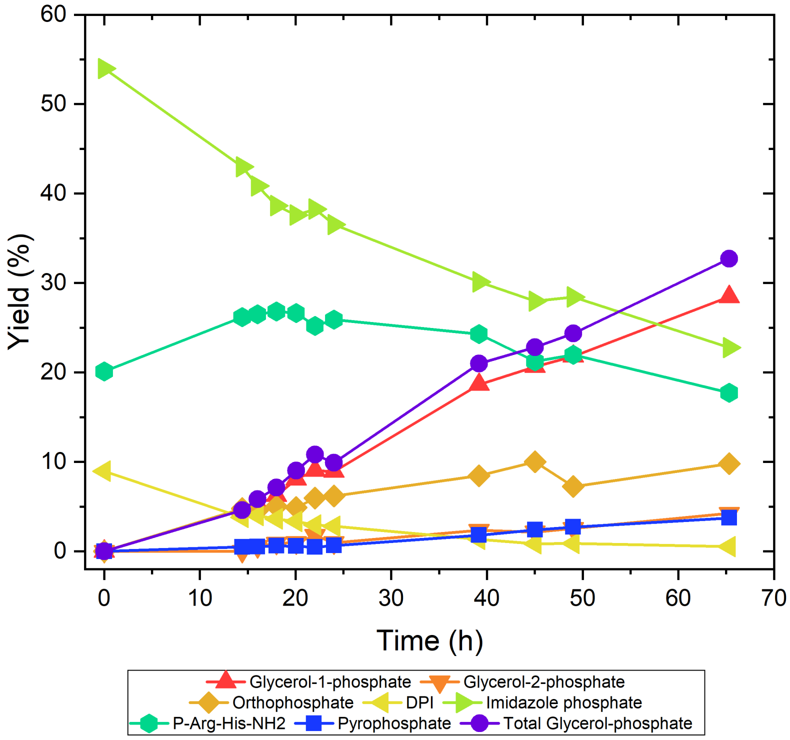

Supporting Figure 105: Changes in yield over time for the reaction of 0.13 mmol calcium imidazole phosphate, 3.25 mmol glycerol and 0.13 mmol Arg-His-NH_2 at pH 7.5 and 22 °C. DPI = Diphosphoimidazole. The first time point includes phosphorylation that took place in the freezer at -20 °C and thus the yields of imidazole phosphate, diphosphoimidazole and P-Arg-His-NH_2 are for this time point off.

S3.12.2 Experiment 2 - 3.25 mmol glycerol + 0.13 mmol imidazole phosphate + 0.13 mmol Arg-His-NH₂

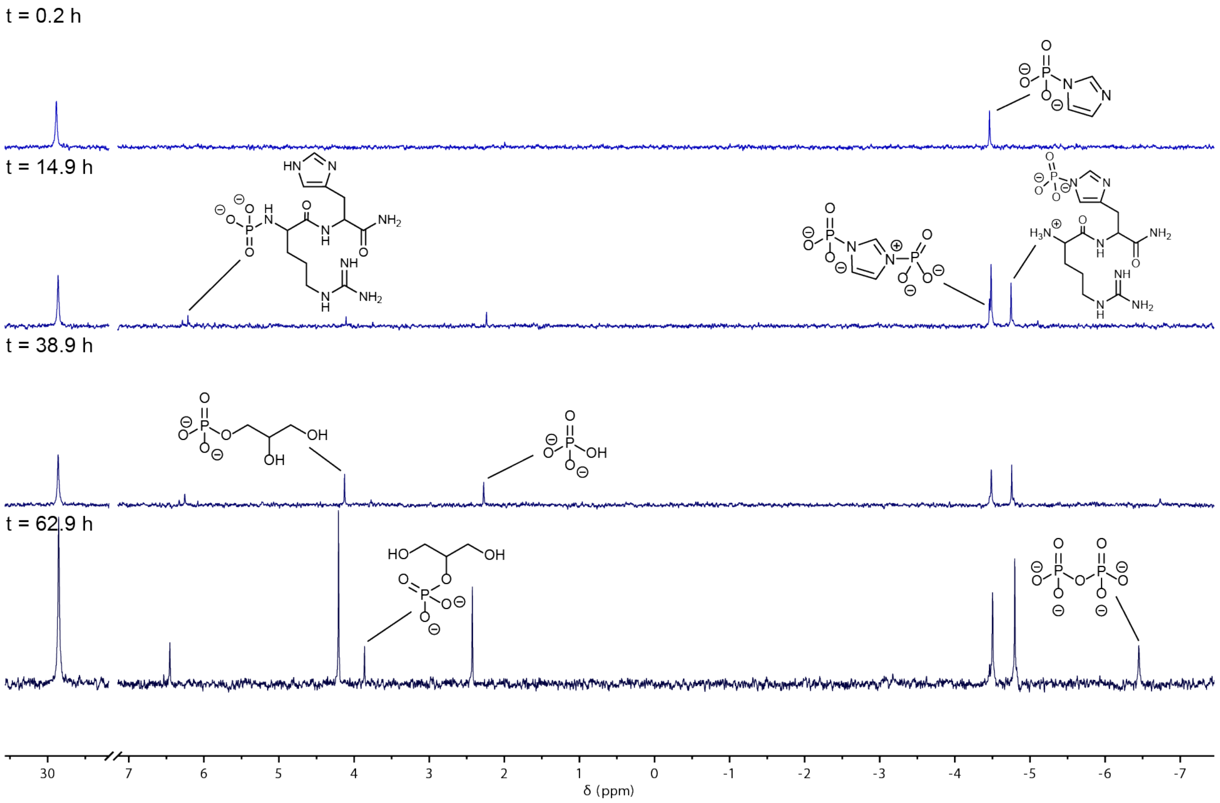

Supporting Figure 106: Representative ^{31}P-NMR spectra over time for the reaction of 0.13 mmol of calcium imidazole phosphate, 3.25 mmol of glycerol and 0.13 mmol of Arg-His-NH_2 at pH 7.5 and 22 °C.

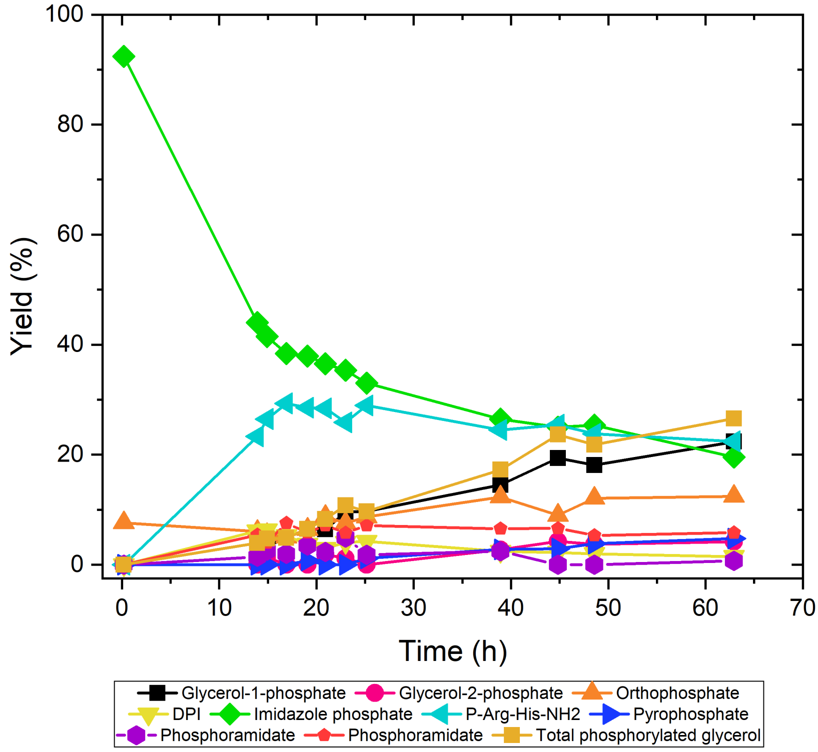

Supporting Figure 107: Changes in yield over time for the reaction of 0.13 mmol calcium imidazole phosphate, 3.25 mmol glycerol and 0.13 mmol Arg-His-NH_2 at pH 7.5 and 22 °C. DPI = Diphosphoimidazole.

S3.12.3 Experiment 3 - 3.25 mmol glycerol + 0.13 mmol imidazole phosphate + 0.13 mmol Arg-His-NH₂

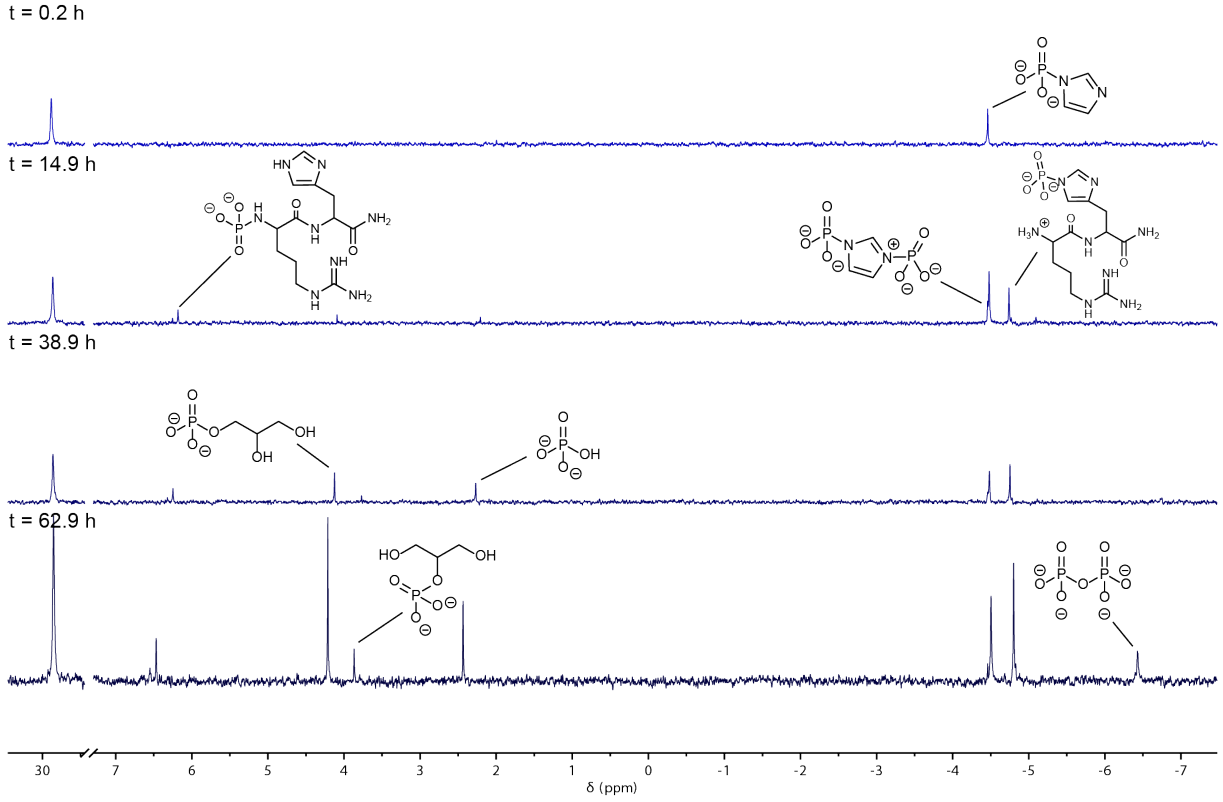

Supporting Figure 108: Representative ^{31}P-NMR spectra over time for the reaction of 0.13 mmol of calcium imidazole phosphate, 3.25 mmol of glycerol and 0.13 mmol of Arg-His-NH_2 at pH 7.5 and 22 °C.

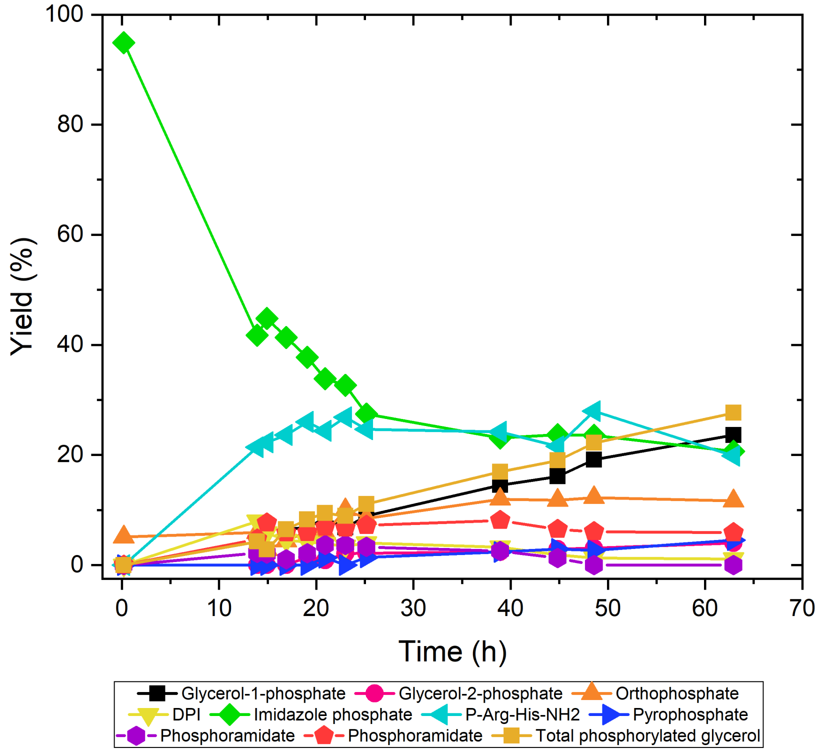

Supporting Figure 109: Changes in yield over time for the reaction of 0.13 mmol calcium imidazole phosphate, 3.25 mmol glycerol and 0.13 mmol Arg-His-NH_2 at pH 7.5 and 22 °C. DPI = Diphosphoimidazole.

S3.12.4 *Combined results for 3.25 mmol glycerol + 0.13 mmol imidazole phosphate + 0.13 mmol Arg-His-NH₂*

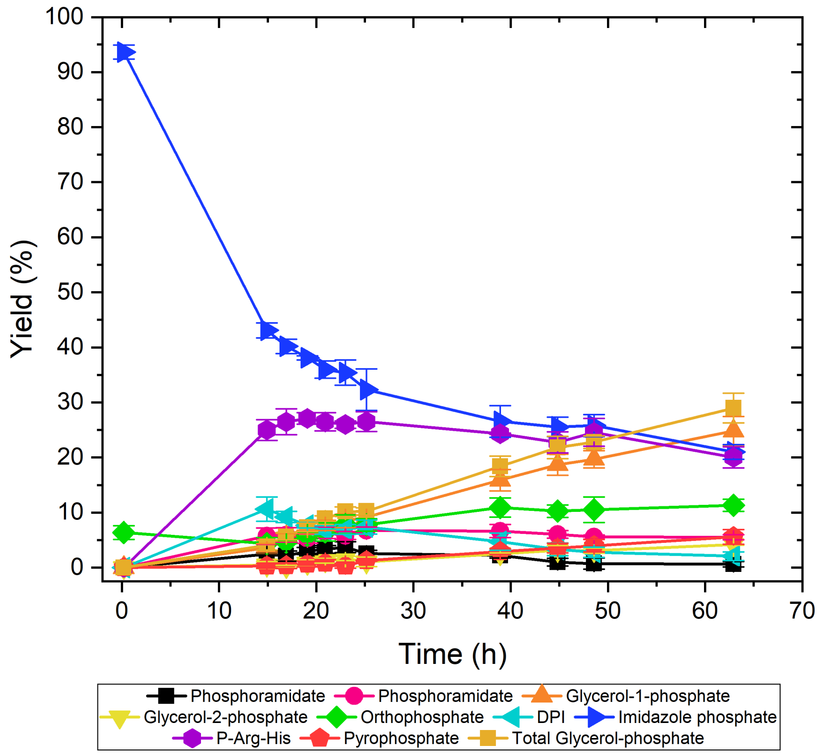

Supporting Figure 110: Changes in yield over time for the reaction of 0.13 mmol calcium imidazole phosphate, 3.25 mmol glycerol and 0.13 mmol Arg-His-NH_2 at pH 7.5 and 22 °C. DPI = Diphosphoimidazole. These data are the mean values and standard deviation based upon triplicate experiments.

S3.12.5 *Characterisation of phosphorylated Arg-His-NH_2 intermediate*

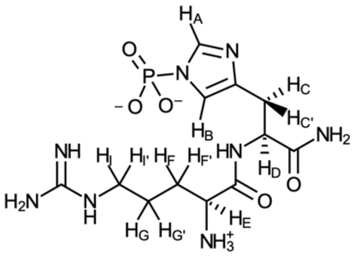

The phosphorylated Arg-His-NH_2 intermediate was characterised *in situ*. A solution of 50 mM Arg-His-NH_2 and 50 mM calcium imidazole phosphate in 0.5 mL 0.5 M MOPS buffer at pH 7.5 in 9 : 1 H_2O : D_2O containing 0.1 M citric acid and 50 mM HMPA internal standard was prepared according to the procedure in Section S2.2 and S2.4.

^{31}P NMR (202.46 MHz, 0.5 M MOPS + 0.1 M Citric Acid in 9 : 1 H_2O : D_2O at pH 7.5 and 22 °C): *phosphorylated Arg-His-NH_2 intermediate* δ (ppm) = - 4.75 (s, 1P). ^{1}H NMR (500.13 MHz, 0.5 M MOPS + 0.1 M Citric Acid in 9 : 1 H_2O : D_2O at pH 7.5 and 22 °C): *phosphorylated Arg-His-NH_2 intermediate* δ (ppm) = 7.84 (s, 1H, H_A), 7.06 (s, 1H, H_B), 4.53 (dd, 1H, H_D), 3.71 (dd, 1H, H_E), 3.09 (2H, $H_{I+I'}$), 3.04 (2H, $H_{C+C'}$), 1.68 (2H, $H_{F+F'}$), 1.46 (2H, $H_{G+G'}$). ^{13}C NMR (125.77 MHz, 0.5 M MOPS + 0.1 M Citric Acid in 9 : 1 H_2O : D_2O at pH 7.5 and 22 °C): *phosphorylated*

Arg-His-NH₂ intermediate δ (ppm) = 175.2 (s, 1C, $CONH_2$), 172.8 (s, 1C, Amide N-C=O), 137.9 (s, 1C, imid C-H_A), 133.8 (s, 1C, imid), 118.5 (s, 1C, imid C-H_B), 53.7 (s, 1C, C-H_D), 53.4 (s, 1C, C-H_E), 40.6 (s, 1C, C-H_I), 28.4 (s, 1C, C-H_C), 29.4 (s, 1C, C-H_F) 23.5 (s, 1C, C-H_G).

S3.13 Phosphorylation of glycerol by imidazole phosphate with Ala-His catalyst

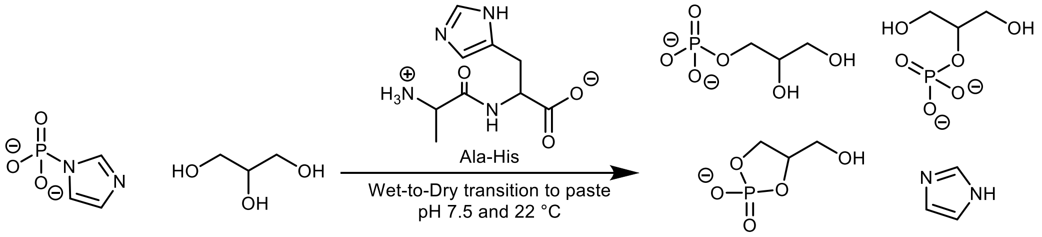

The experiment was carried out according to the procedure in S3.1 but with the 65 mM of histidine replaced by 65 mM Ala-His (29.4 mg, 0.13 mmol). The experiment was repeated in triplicate. Supporting Figures 111, 113 and 115 depict representative ^{31}P NMR spectra for the reaction over time. The changes in yield over time for all phosphate containing species are shown in Supporting Figure 112, 114 and 116. The mean experimental results with the standard deviation of each experimental data point from the triplicate experiments are shown in Supporting Figure 117.

S3.13.1 Experiment 1 - 3.25 mmol glycerol + 0.13 mmol imidazole phosphate + 0.13 mmol Ala-His

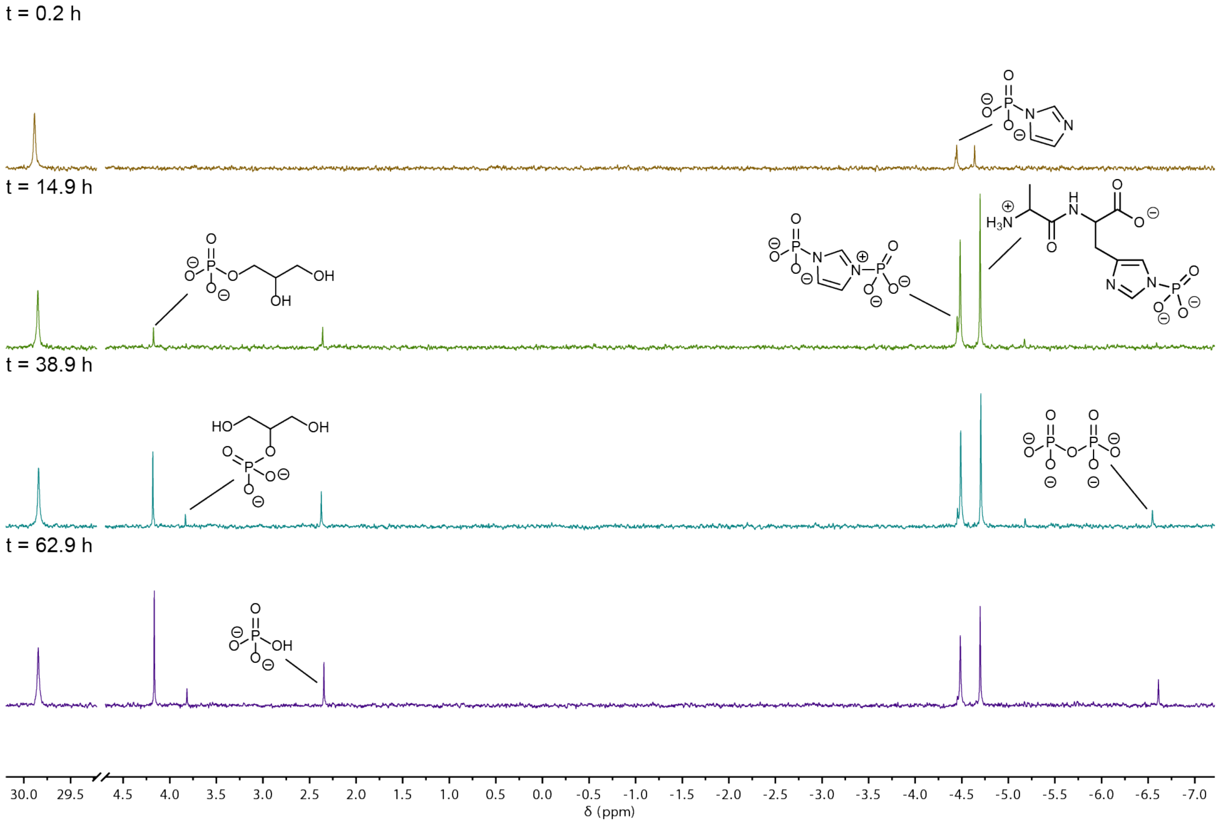

Supporting Figure 111: Representative ^{31}P-NMR spectra over time for the reaction of 0.13 mmol of calcium imidazole phosphate, 3.25 mmol of glycerol and 0.13 mmol of Ala-His at pH 7.5 and 22 °C.

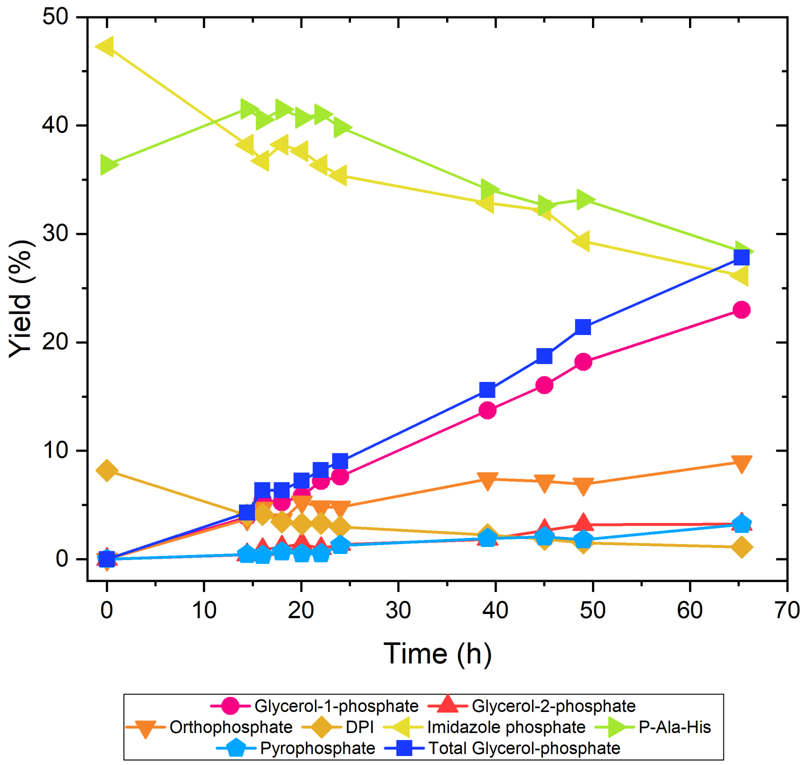

Supporting Figure 112: Changes in yield over time for the reaction of 0.13 mmol of calcium imidazole phosphate, 3.25 mmol of glycerol and 0.13 mmol of Ala-His at pH 7.5 and 22 °C. DPI = Diphosphoimidazole. The first time point includes phosphorylation that took place in the freezer at -20 °C and thus the yields of imidazole phosphate, diphosphoimidazole and P-Ala-His are for this time point off.

S3.13.2 Experiment 2 - 3.25 mmol glycerol + 0.13 mmol imidazole phosphate + 0.13 mmol Ala-His

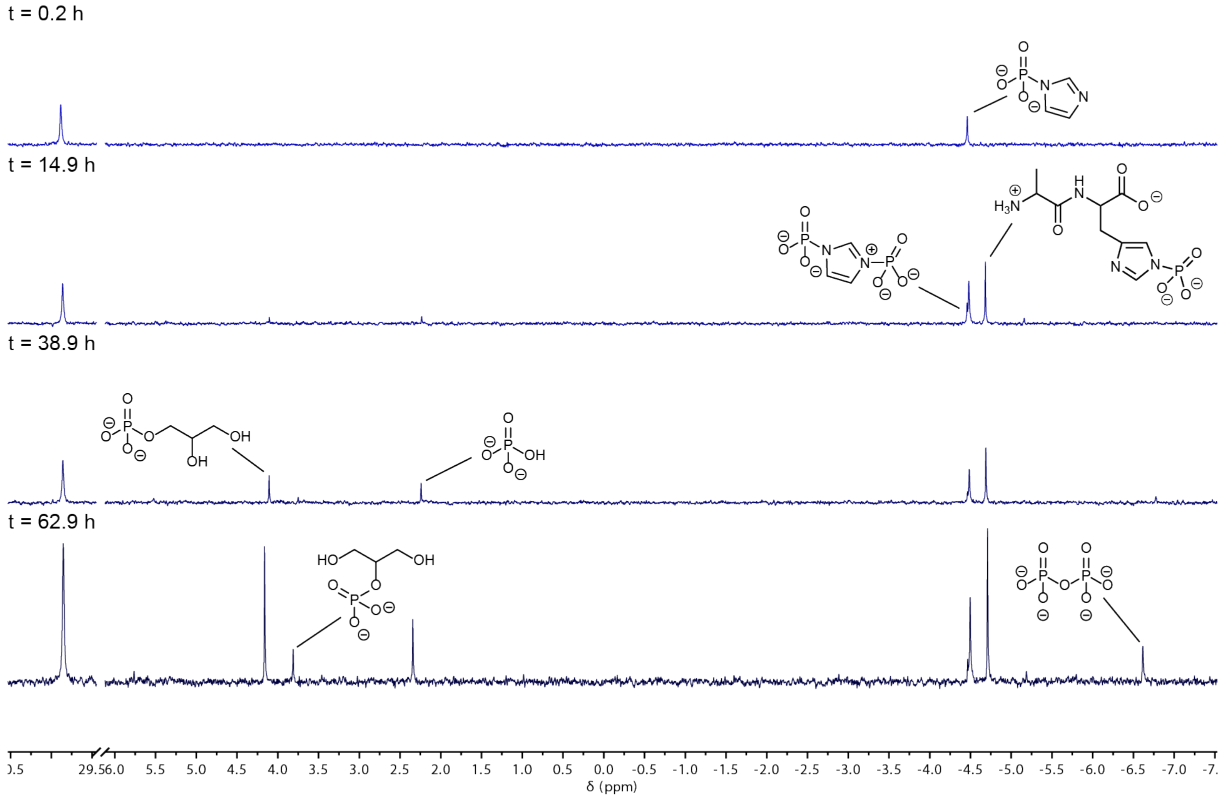

Supporting Figure 113: Representative ^{31}P-NMR spectra over time for the reaction of 0.13 mmol of calcium imidazole phosphate, 3.25 mmol of glycerol and 0.13 mmol of Ala-His at pH 7.5 and 22 °C.

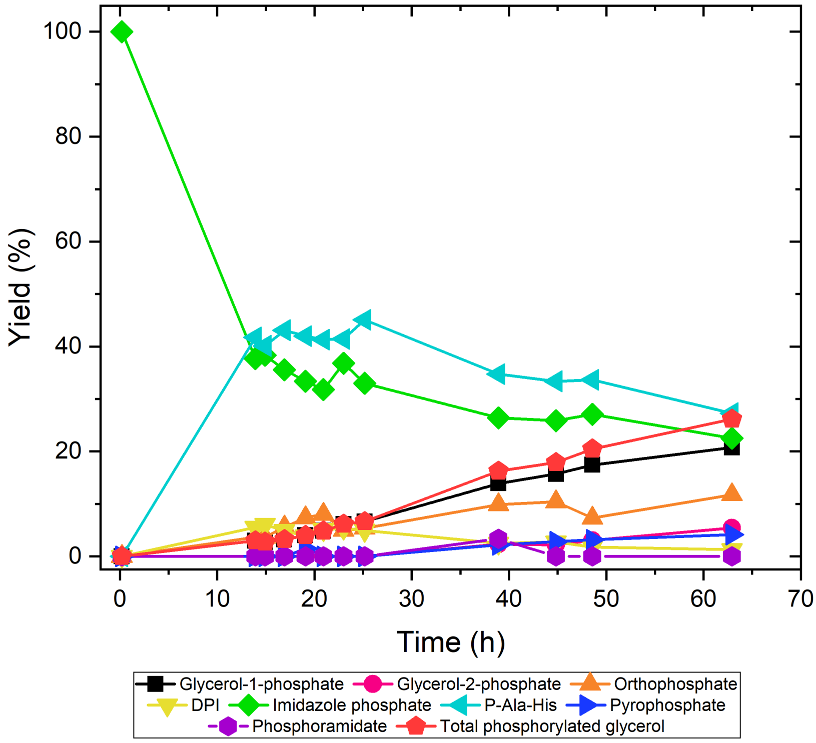

Supporting Figure 114: Changes in yield over time for the reaction of 0.13 mmol of calcium imidazole phosphate, 3.25 mmol of glycerol and 0.13 mmol of Ala-His at pH 7.5 and 22 °C. DPI = Diphosphoimidazole.

S3.13.3 Experiment 3 - 3.25 mmol glycerol + 0.13 mmol imidazole phosphate + 0.13 mmol Ala-His

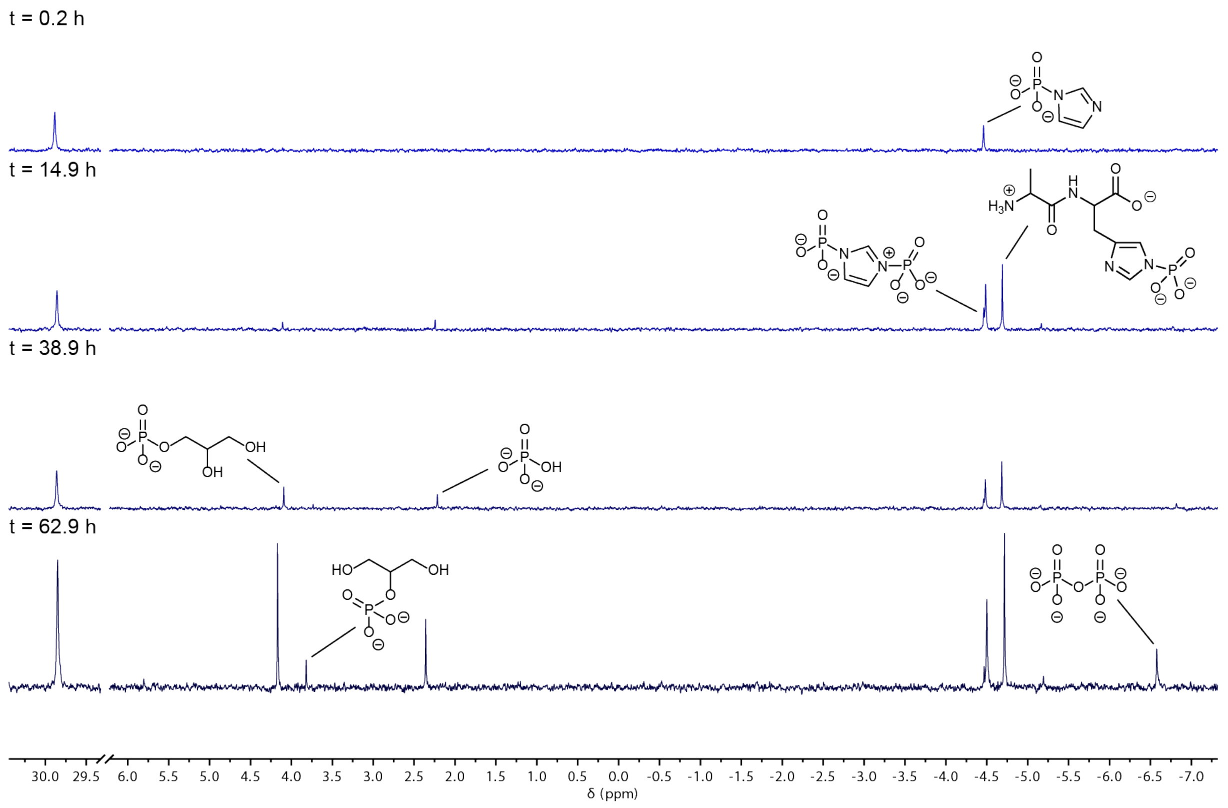

Supporting Figure 115: Representative ^{31}P-NMR spectra over time for the reaction of 0.13 mmol of calcium imidazole phosphate, 3.25 mmol of glycerol and 0.13 mmol of Ala-His at pH 7.5 and 22 °C.

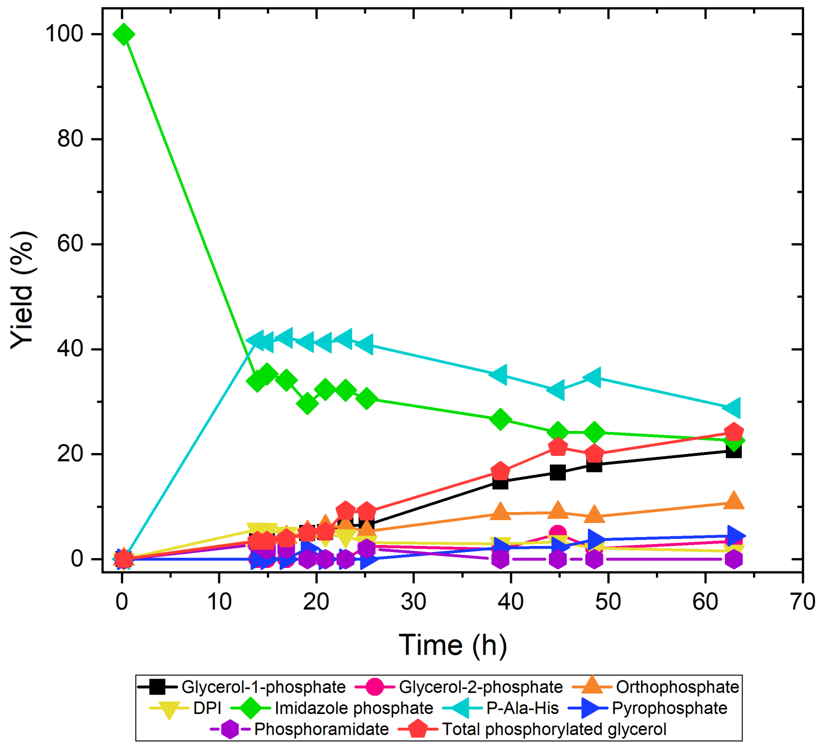

Supporting Figure 116: Changes in yield over time for the reaction of 0.13 mmol of calcium imidazole phosphate, 3.25 mmol of glycerol and 0.13 mmol of Ala-His at pH 7.5 and 22 °C. DPI = Diphosphoimidazole.

S3.13.4 Combined results for 3.25 mmol glycerol + 0.13 mmol imidazole phosphate + 0.13 mmol Ala-His

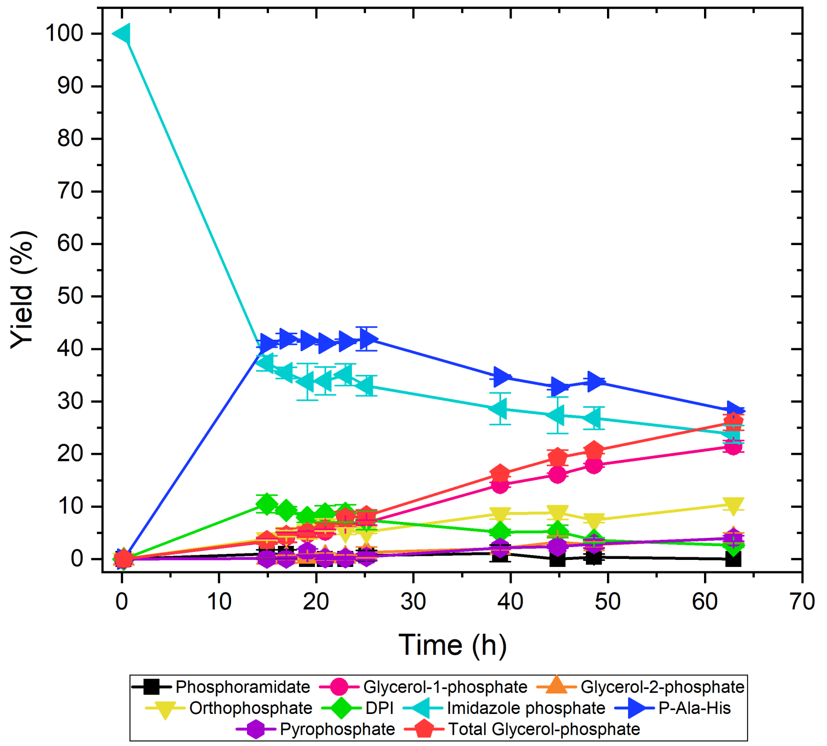

Supporting Figure 117: Changes in yield over time for the reaction of 0.13 mmol of calcium imidazole phosphate, 3.25 mmol of glycerol and 0.13 mmol of Ala-His at pH 7.5 and 22 °C. DPI = Diphosphoimidazole. These data are the mean values and standard deviation based upon triplicate experiments.

S3.13.5 Characterisation of phosphorylated Ala-His intermediate

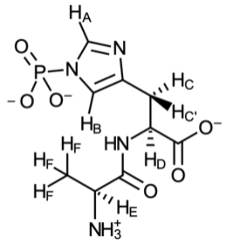

The phosphorylated Ala-His intermediate was characterised *in situ*. A solution of 50 mM Ala-His and 50 mM calcium imidazole phosphate in 0.5 mL 0.5 M MOPS buffer at pH 7.5 in 9 : 1 H_2O : D_2O containing 0.1 M citric acid and 50 mM HMPA internal standard was prepared according to the procedure in Section S2.2 and S2.4.

^{31}P NMR (202.46 MHz, 0.5 M MOPS + 0.1 M Citric Acid in 9 : 1 H_2O : D_2O at pH 7.5 and 22 °C): *phosphorylated Ala-His intermediate* δ (ppm) = - 4.87 (s, 1P). ^{1}H NMR (500.13 MHz, 0.5 M MOPS + 0.1 M Citric Acid in 9 : 1 H_2O : D_2O at pH 7.5 and 22 °C): *phosphorylated Ala-His intermediate* δ (ppm) = 7.97 (s, 1H, H_A), 7.04 (s, 1H, H_B), 4.34 (dd, 1H, H_D), 3.88 (q, 1H, H_E), 3.04 (1H, H_C), 2.94 (1H, $H_{C'}$), 1.39 (d, 3H, H_F). ^{13}C NMR (125.77 MHz, 0.5 M MOPS + 0.1 M Citric Acid in 9 : 1 H_2O : D_2O at pH 7.5 and 22 °C): *phosphorylated Ala-His intermediate* δ

(ppm) = 177.0 (s, 1C, COOH), 170.9 (s, 1C, Amide N-C=O), 136.9 (s, 1C, imid C-H$_A$), 133.6 (s, 1C, imid), 118.4 (s, 1C, imid C-H$_B$), 54.9 (s, 1C, C-H$_D$), 49.2 (s, 1C, C-H$_E$), 28.3 (s, 1C, C-H$_{C+C'}$), 17.0 (s, 1C, C-H$_F$).

S3.14 Phosphorylation of glycerol by imidazole phosphate with Ser-His catalyst

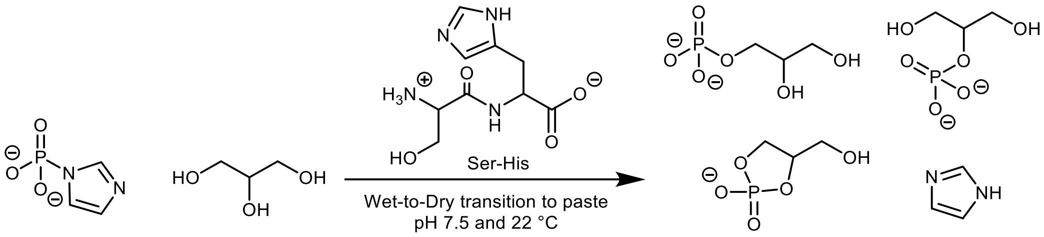

The experiment was carried out according to the procedure in S3.1 but with the 65 mM of histidine replaced by 65 mM Ser-His (31.5 mg, 0.13 mmol). The experiment was repeated in triplicate. Supporting Figures 118, 120 and 122 depict representative ^{31}P NMR spectra for the reaction over time. The changes in yield over time for all phosphate containing species are shown in Supporting Figures 119, 121 and 123. The mean experimental results with the standard deviation of each experimental data point from the triplicate experiments are shown in Supporting Figure 124.

S3.14.1 Experiment 1 - 3.25 mmol glycerol + 0.13 mmol imidazole phosphate + 0.13 mmol Ser-His

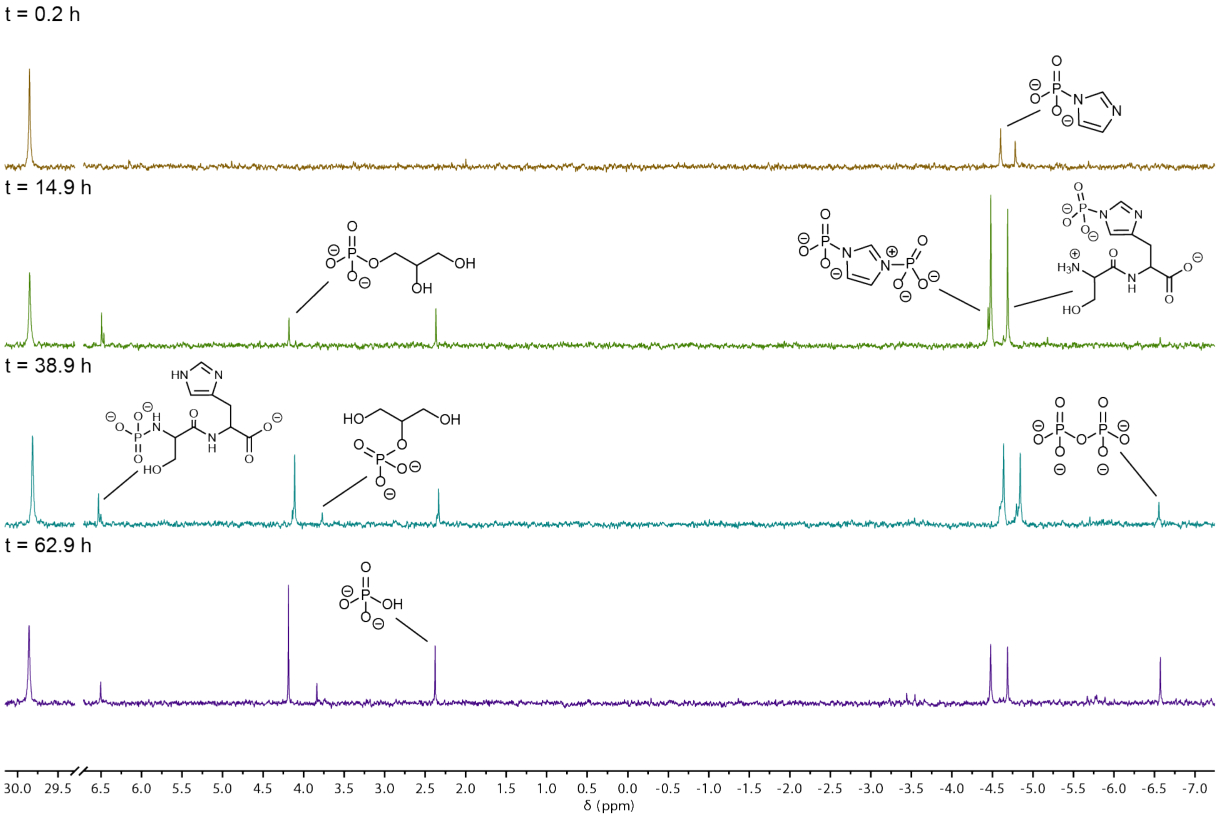

Supporting Figure 118: Representative ^{31}P-NMR spectra over time for the reaction of 0.13 mmol of calcium imidazole phosphate, 3.25 mmol of glycerol and 0.13 mmol of Ser-His at pH 7.5 and 22 °C.

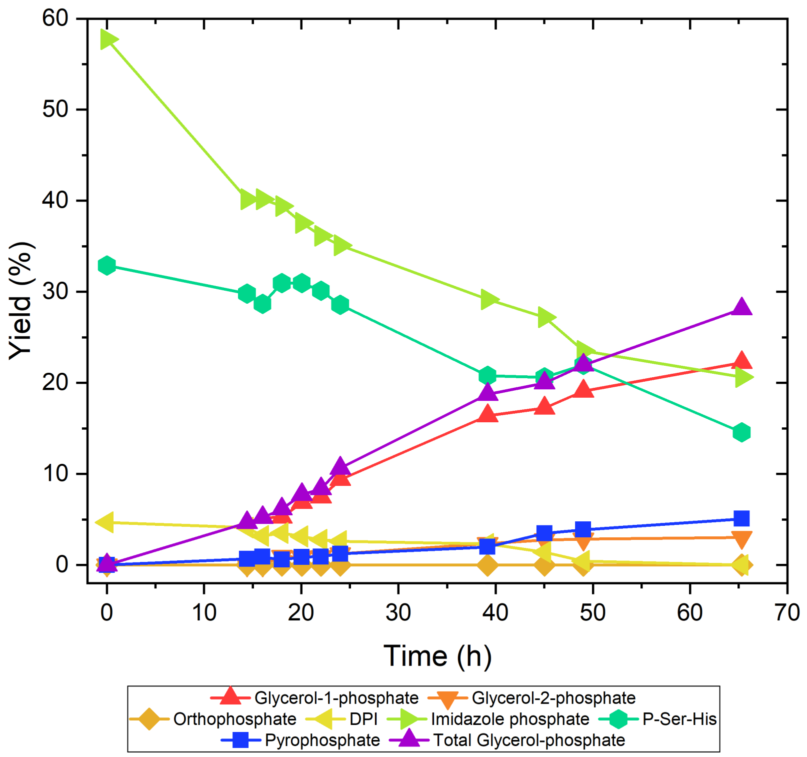

Supporting Figure 119: Changes in yield over time for the reaction of 0.13 mmol of calcium imidazole phosphate, 3.25 mmol of glycerol and 0.13 mmol of Ser-His at pH 7.5 and 22 °C. DPI = Diphosphoimidazole. The first time point includes phosphorylation that took place in the freezer at -20 °C and thus the yields of imidazole phosphate, diphosphoimidazole and P-Ala-His are for this time point off.

S3.14.2 Experiment 2 - 3.25 mmol glycerol + 0.13 mmol imidazole phosphate + 0.13 mmol Ser-His

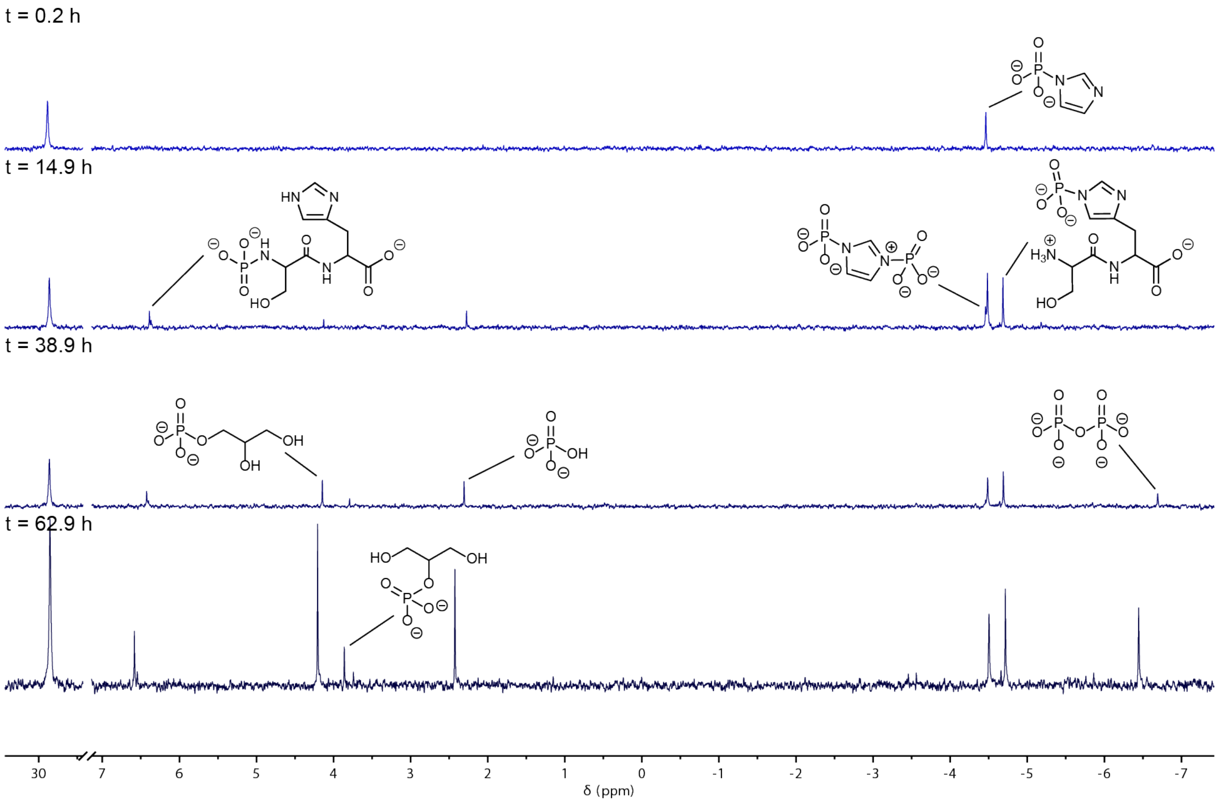

Supporting Figure 120: Representative ^{31}P-NMR spectra over time for the reaction of 0.13 mmol of calcium imidazole phosphate, 3.25 mmol of glycerol and 0.13 mmol of Ser-His at pH 7.5 and 22 °C.

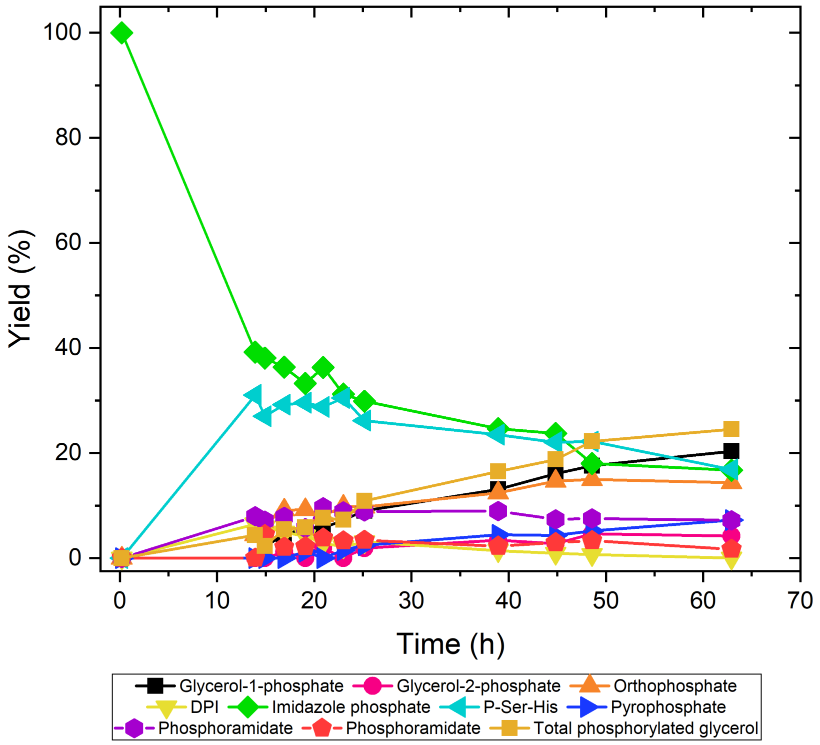

Supporting Figure 121: Changes in yield over time for the reaction of 0.13 mmol of calcium imidazole phosphate, 3.25 mmol of glycerol and 0.13 mmol of Ser-His at pH 7.5 and 22 °C. DPI = Diphosphoimidazole.

S3.14.3 Experiment 3 - 3.25 mmol glycerol + 0.13 mmol imidazole phosphate + 0.13 mmol Ser-His

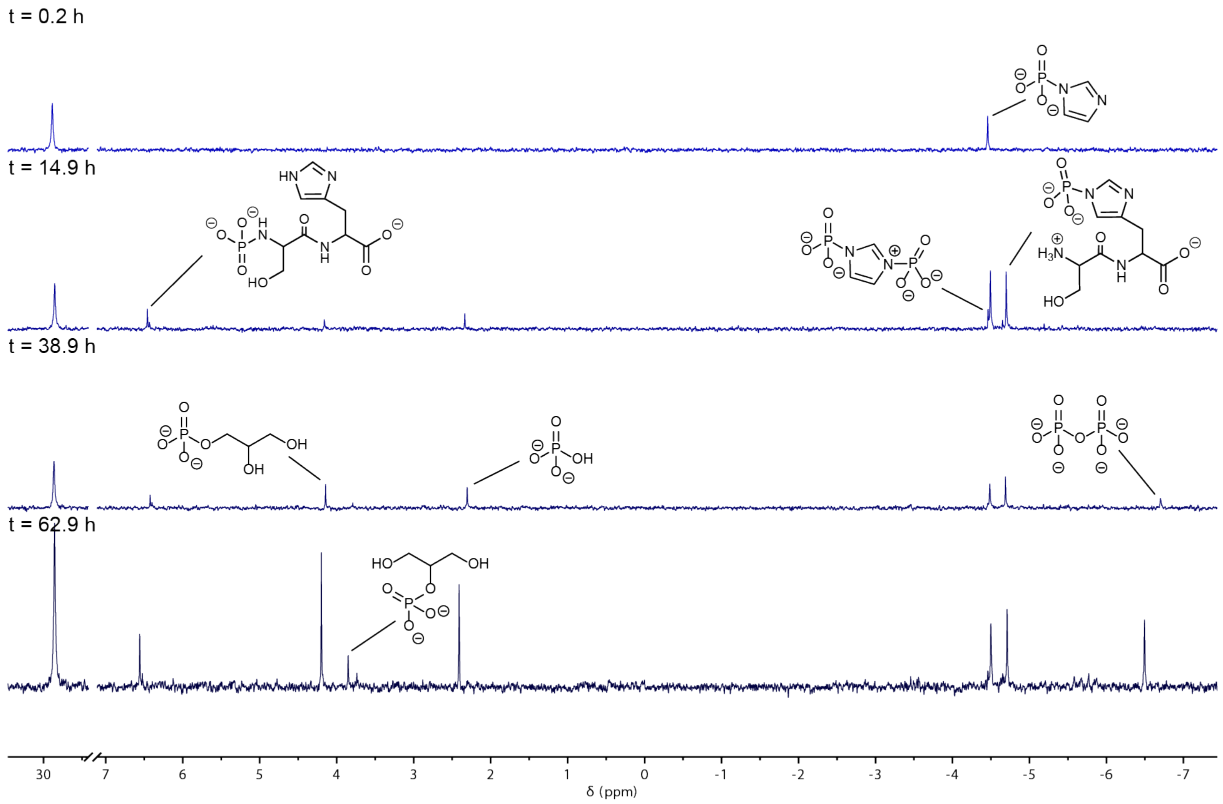

Supporting Figure 122: Representative ^{31}P-NMR spectra over time for the reaction of 0.13 mmol of calcium imidazole phosphate, 3.25 mmol of glycerol and 0.13 mmol of Ser-His at pH 7.5 and 22 °C.

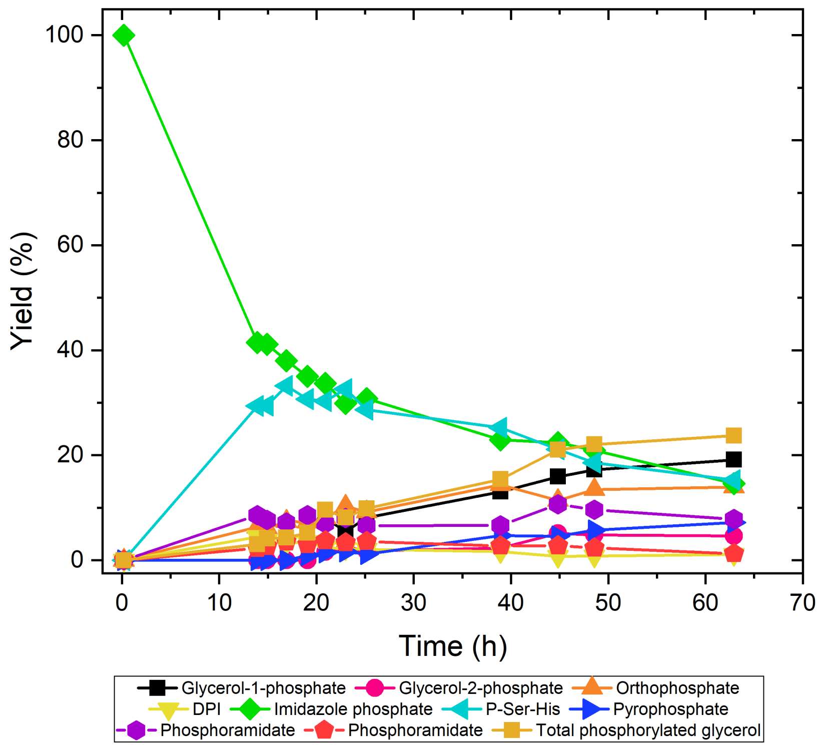

Supporting Figure 123: Changes in yield over time for the reaction of 0.13 mmol of calcium imidazole phosphate, 3.25 mmol of glycerol and 0.13 mmol of Ser-His at pH 7.5 and 22 °C. DPI = Diphosphoimidazole.

S3.14.4 *Combined results for 3.25 mmol glycerol + 0.13 mmol imidazole phosphate + 0.13 mmol Ser-His*

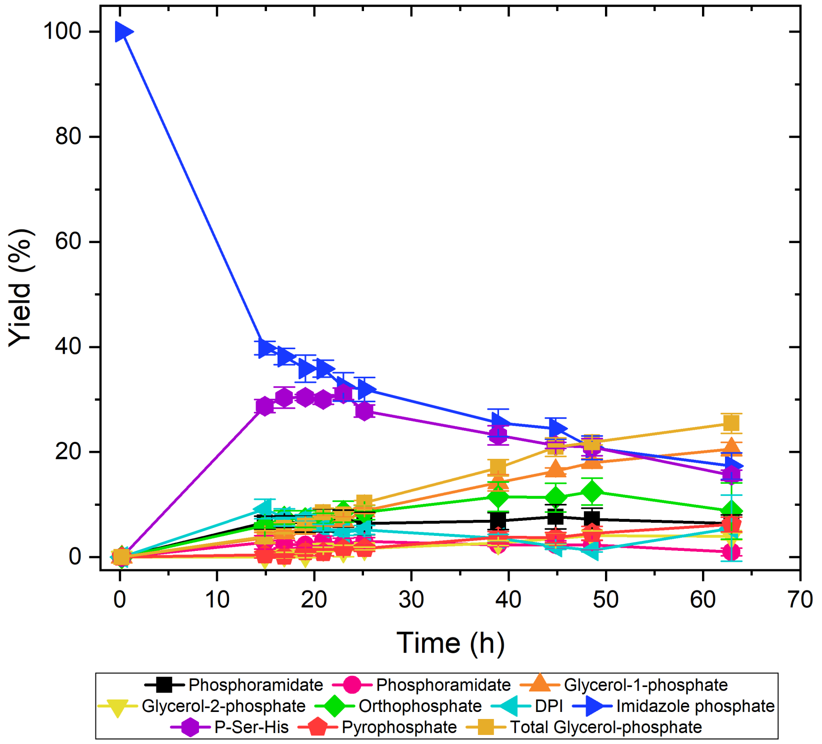

Supporting Figure 124: Changes in yield over time for the reaction of 0.13 mmol of calcium imidazole phosphate, 3.25 mmol of glycerol and 0.13 mmol of Ser-His at pH 7.5 and 22 °C. DPI = Diphosphoimidazole. These data are the mean values and standard deviation based upon triplicate experiments.

S3.14.5 *Characterisation of phosphorylated Ser-His intermediate*

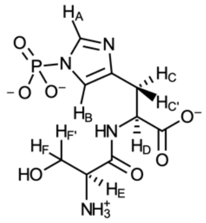

The phosphorylated Ser-His intermediate was characterised *in situ*. A solution of 50 mM Ser-His and 50 mM calcium imidazole phosphate in 0.5 mL 0.5 M MOPS buffer at pH 7.5 in 9 : 1 H_2O : D_2O containing 0.1 M citric acid and 50 mM HMPA internal standard was prepared according to the procedure in Section S2.2 and S2.4.

^{31}P NMR (202.46 MHz, 0.5 M MOPS + 0.1 M Citric Acid in 9 : 1 H_2O : D_2O at pH 7.5 and 22 °C): *phosphorylated Ser-His intermediate* δ (ppm) = - 4.82 (s, 1P). ^{1}H NMR (500.13 MHz, 0.5 M MOPS + 0.1 M Citric Acid in 9 : 1 H_2O : D_2O at pH 7.5 and 22 °C): *phosphorylated Ser-His intermediate* δ (ppm) = 8.02 (s, 1H, H_A), 7.07 (s, 1H, H_B), 4.39 (dd, 1H, H_D), 3.81 (s, 1H, $H_{F+F'}$), 3.75 (s, 1H, H_E), 3.08 (1H, H_C), 2.96 (1H, $H_{C'}$). ^{13}C NMR (125.77 MHz, 0.5 M MOPS + 0.1 M Citric Acid in 9 : 1 H_2O : D_2O at pH 7.5 and 22 °C): *phosphorylated Ser-His intermediate* δ (ppm) = 177.0 (s, 1C, COOH), 170.5 (s, 1C, Amide N-C=O), 136.7 (s, 1C, imid C-H_A), 133.2 (s, 1C, imid), 118.5 (s, 1C, imid C-H_B), 61.9 (s, 1C, C-H_E), 55.2 (s, 1C, C-$H_{F+F'}$), 55.3 (s, 1C, C-H_D), 28.6 (s, 1C, C-$H_{C+C'}$).

S3.15 Phosphorylation of glycerol by imidazole phosphate with Gly-Lys-His catalyst

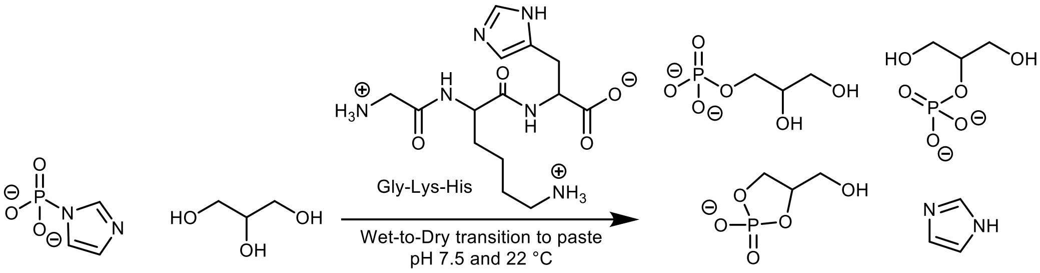

The experiment was carried out according to the procedure in S3.1 but with the 65 mM of histidine replaced by 65 mM Gly-Lys-His acetate salt (44.2 mg, 0.13 mmol). The experiment was repeated in triplicate. Supporting Figures 125, 127 and 129 depict representative ^{31}P NMR spectra for the reaction over time. The changes in yield over time for all phosphate containing species are shown in Supporting Figure 126, 128 and 130. The mean experimental results with the standard deviation of each experimental data point from the triplicate experiments are shown in Supporting Figure 131.

S3.15.1 Experiment 1 - 3.25 mmol glycerol + 0.13 mmol imidazole phosphate + 0.13 mmol Gly-Lys-His

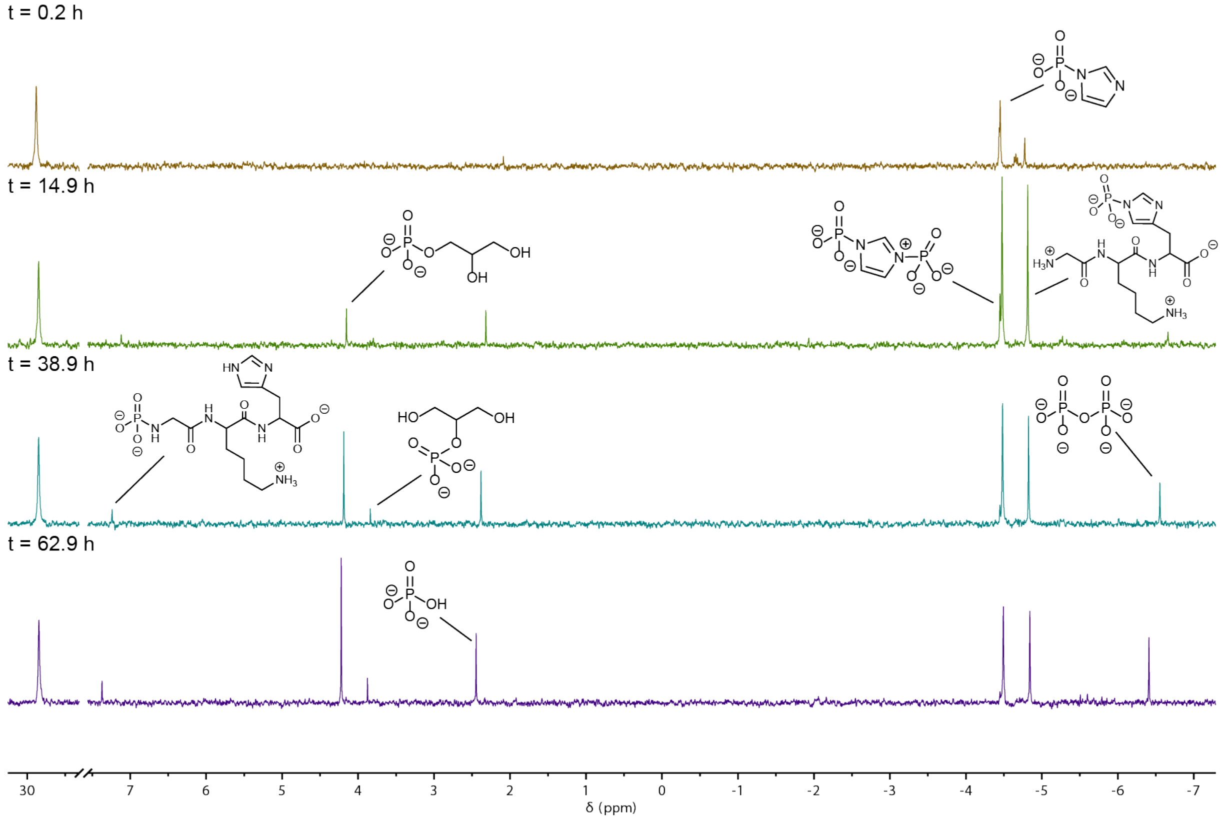

Supporting Figure 125: Representative ^{31}P-NMR spectra over time for the reaction of 0.13 mmol of calcium imidazole phosphate, 3.25 mmol of glycerol and 0.13 mmol of Gly-Lys-His at pH 7.5 and 22 °C.

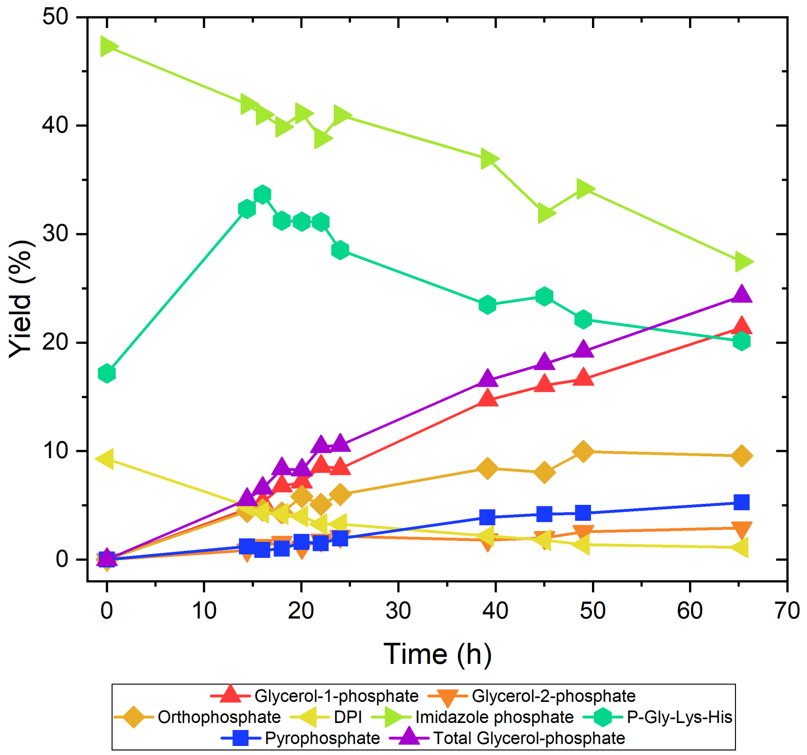

Supporting Figure 126: Changes in yield over time for the reaction of 0.13 mmol calcium imidazole phosphate, 3.25 mmol glycerol and 0.13 mmol Gly-Lys-His at pH 7.5 and 22 °C. DPI = Diphosphoimidazole. The first time point includes phosphorylation that took place in the freezer at -20 °C and thus the yields of imidazole phosphate, diphosphoimidazole and P-Gly-Lys-His are for this time point off.

S3.15.2 *Experiment 2 - 3.25 mmol glycerol + 0.13 mmol imidazole phosphate + 0.13 mmol Gly-Lys-His*

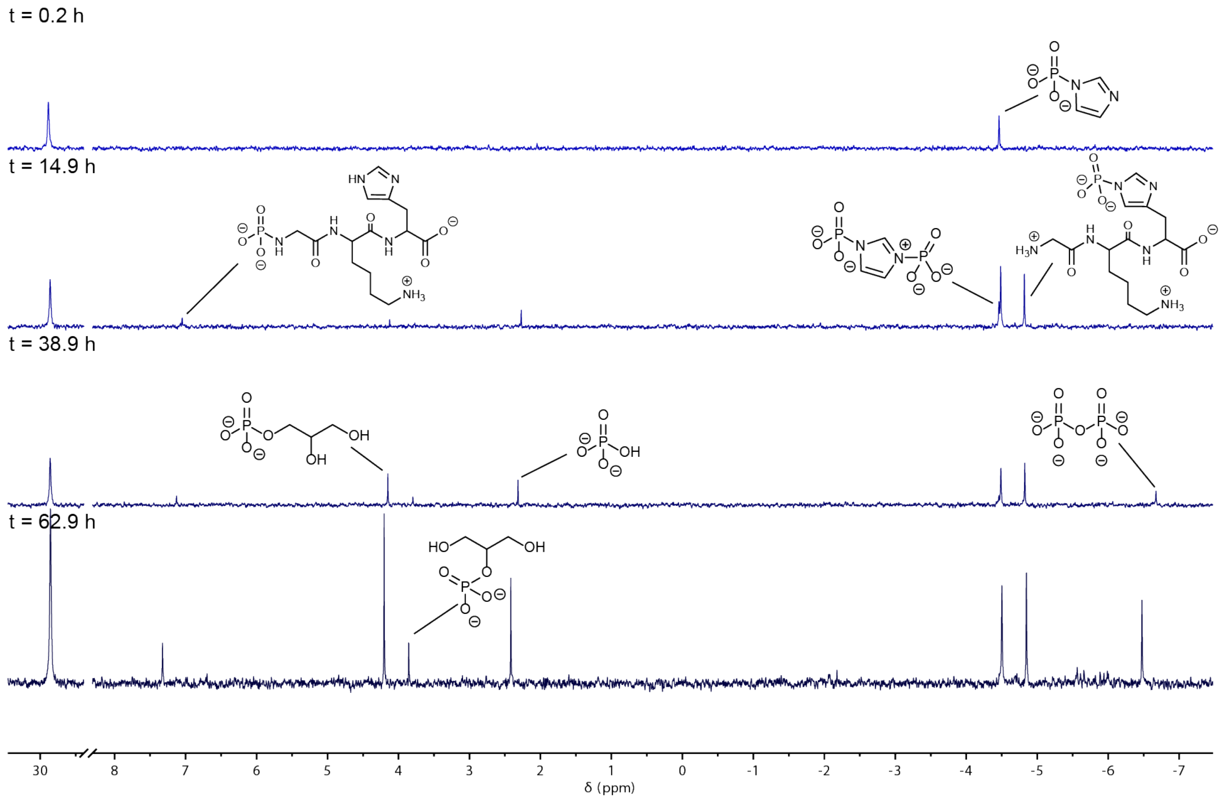

Supporting Figure 127: Representative ^{31}P-NMR spectra over time for the reaction of 0.13 mmol of calcium imidazole phosphate, 3.25 mmol of glycerol and 0.13 mmol of Gly-Lys-His at pH 7.5 and 22 °C.

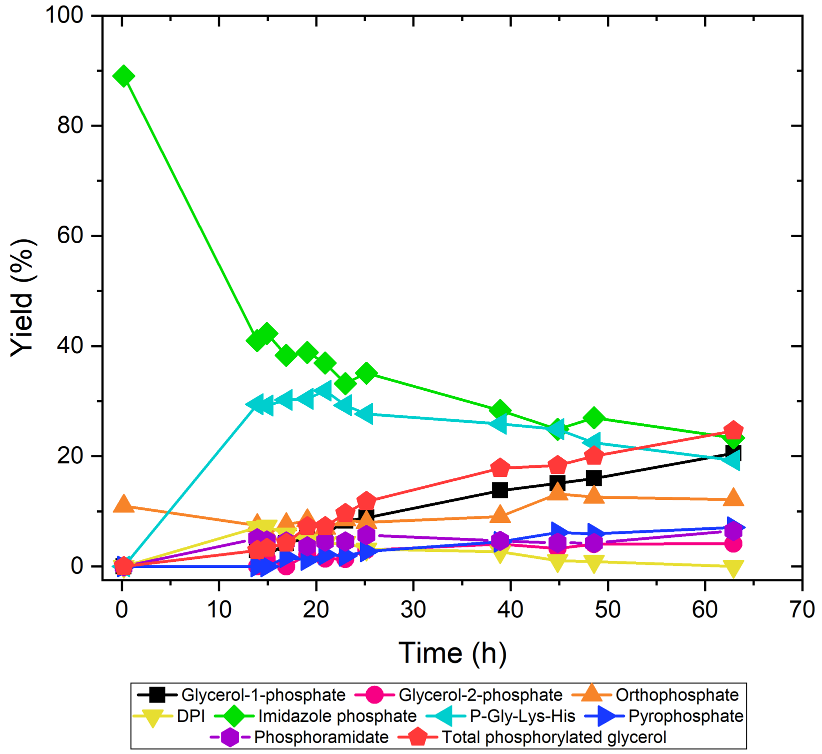

Supporting Figure 128: Changes in yield over time for the reaction of 0.13 mmol calcium imidazole phosphate, 3.25 mmol glycerol and 0.13 mmol Gly-Lys-His at pH 7.5 and 22 °C. DPI = Diphosphoimidazole.

S3.15.3 Experiment 3 - 3.25 mmol glycerol + 0.13 mmol imidazole phosphate + 0.13 mmol Gly-Lys-His

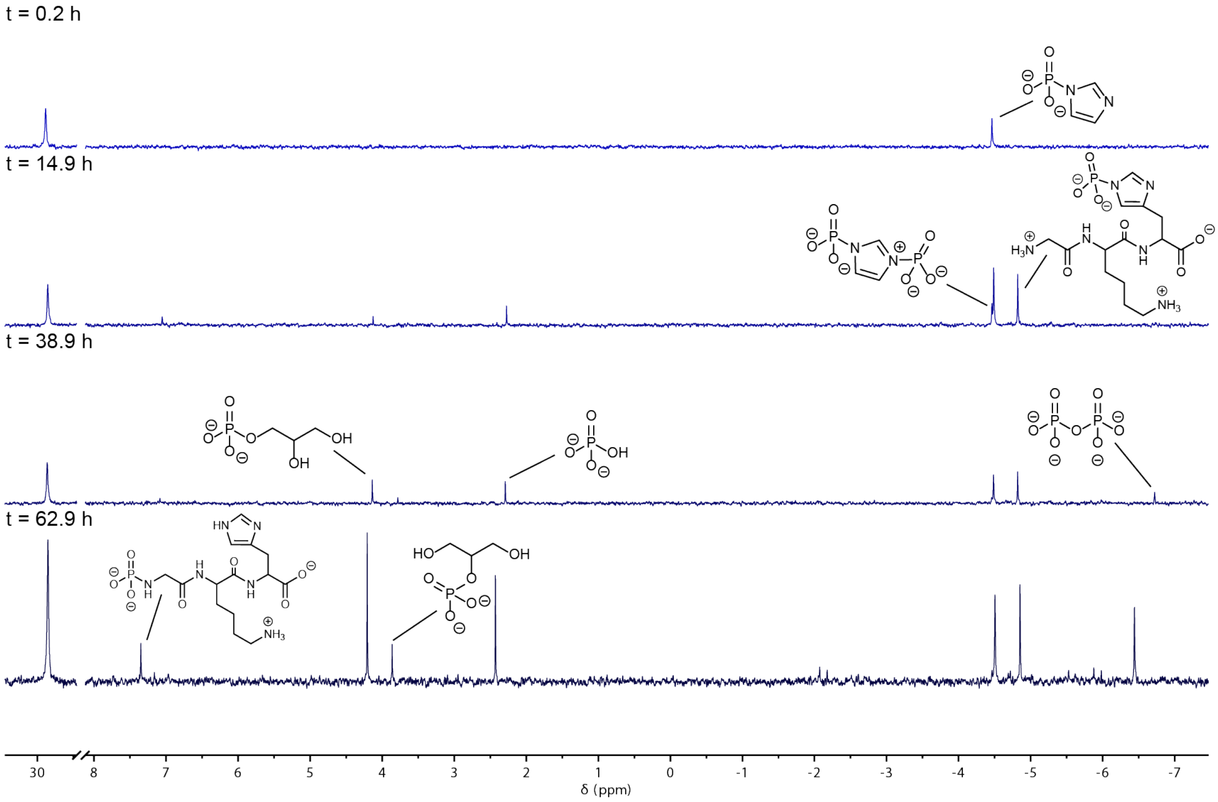

Supporting Figure 129: Representative ^{31}P-NMR spectra over time for the reaction of 0.13 mmol of calcium imidazole phosphate, 3.25 mmol of glycerol and 0.13 mmol of Gly-Lys-His at pH 7.5 and 22 °C.

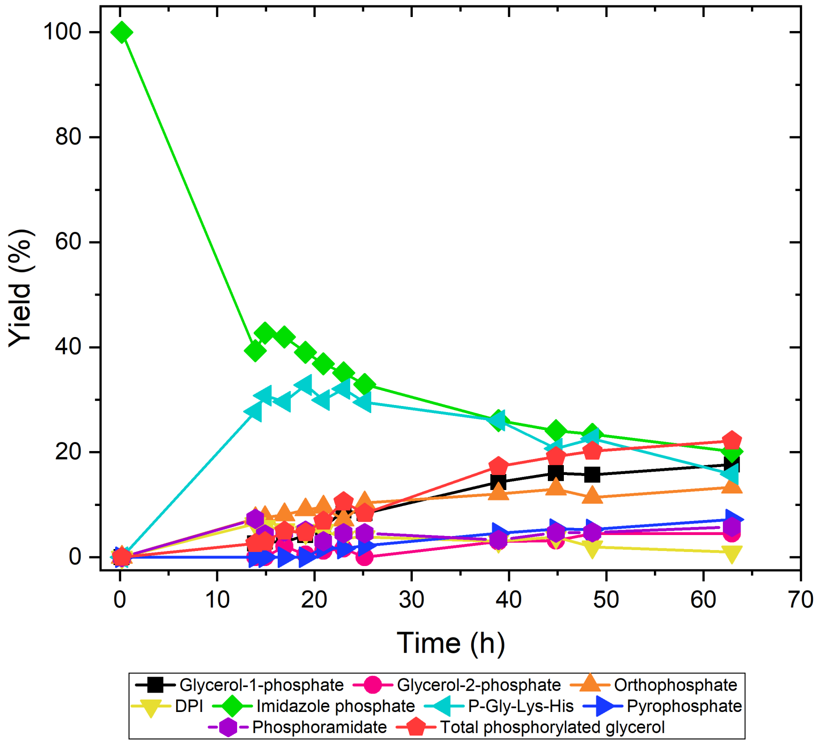

Supporting Figure 130: Changes in yield over time for the reaction of 0.13 mmol calcium imidazole phosphate, 3.25 mmol glycerol and 0.13 mmol Gly-Lys-His at pH 7.5 and 22 °C. DPI = Diphosphoimidazole.

S3.15.4 Combined results for 3.25 mmol glycerol + 0.13 mmol imidazole phosphate + 0.13 mmol Gly-Lys-His

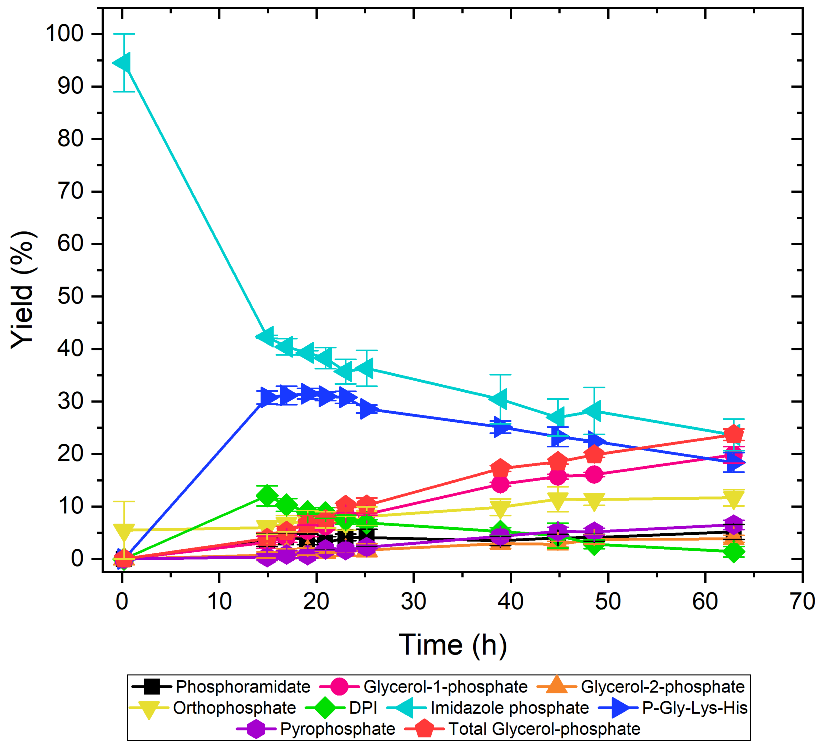

Supporting Figure 131: Changes in yield over time for the reaction of 0.13 mmol calcium imidazole phosphate, 3.25 mmol glycerol and 0.13 mmol Gly-Lys-His at pH 7.5 and 22 °C. DPI = Diphosphoimidazole. These data are the mean values and standard deviation based upon triplicate experiments.

S3.15.5 Characterisation of phosphorylated Gly-Lys-His intermediate

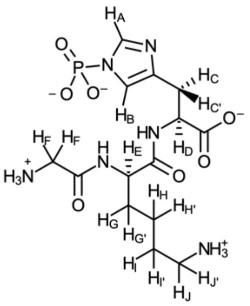

The phosphorylated Gly-Lys-His intermediate was characterised *in situ*. A solution of 50 mM Gly-Lys-His and 50 mM calcium imidazole phosphate in 0.5 mL 0.5 M MOPS buffer at pH 7.5 in 9 : 1 H_2O : D_2O containing 0.1 M citric acid and 50 mM HMPA internal standard was prepared according to the procedure in Section S2.2 and S2.4.

^{31}P NMR (202.46 MHz, 0.5 M MOPS + 0.1 M Citric Acid in 9 : 1 H_2O : D_2O at pH 7.5 and 22 °C): *phosphorylated Gly-Lys-His intermediate* δ (ppm) = - 4.98 (s, 1P). ^{1}H NMR (500.13 MHz, 0.5 M MOPS + 0.1 M Citric Acid in 9 : 1 H_2O : D_2O at pH 7.5 and 22 °C): *phosphorylated Gly-Lys-His intermediate* δ (ppm) = 8.07 (s, 1H, H_A), 7.05 (s, 1H, H_B), 4.35 (dd, 1H, H_D), 4.20 (dd, 1H, H_E), 3.72 (s, 2H, H_F), 3.07 (1H, H_C), 2.91 (1H, $H_{C'}$), 2.87 (2H, $H_{J+J'}$), 1.65

(m, 2H, $H_{G+G'}$), 1.55 (m, 2H, $H_{I+I'}$), 1.27 (m, 2H, $H_{H+H'}$). ^{13}C NMR (125.77 MHz, 0.5 M MOPS + 0.1 M Citric Acid in 9 : 1 H_2O : D_2O at pH 7.5 and 22 °C): *phosphorylated Gly-Lys-His intermediate* δ (ppm) = 176.9 (s, 1C, COOH), 173.0 (s, 1C, Amide N-C=O), 167.6 (s, 1C, Amide N-C=O), 136.1 (s, 1C, imid C-H_A), 132.5 (s, 1C, imid), 118.7 (s, 1C, imid C-H_B), 54.7 (s, 1C, C-H_D), 53.9 (s, 1C, C-H_E), 40.7 (s, 1C, C-H_F), 39.2 (s, 1C, C-H_J), 30.3 (s, 1C, C-H_G), 28.5 (s, 1C, C-H_C), 26.3 (s, 1C, C-H_I), 21.8 (s, 1C, C-H_H).

S3.16 Phosphorylation of glycerol by imidazole phosphate with Gly-Gly-His catalyst

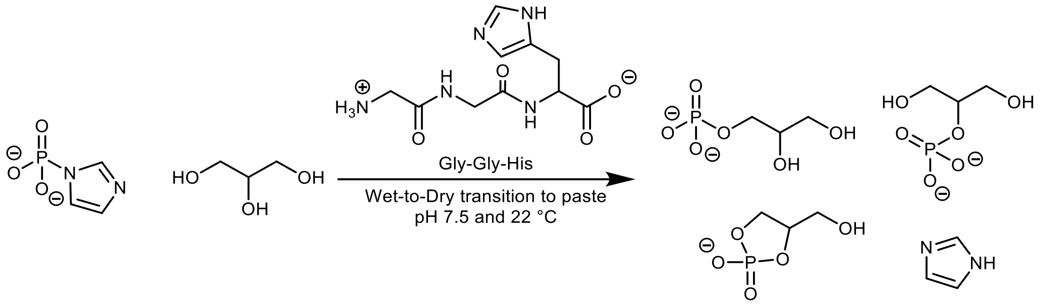

The experiment was carried out according to the procedure in S3.1 but with the 65 mM of histidine replaced by 65 mM Gly-Gly-His (35.0 mg, 0.13 mmol). The experiment was repeated in triplicate. Supporting Figures 132, 134 and 136 depict representative ^{31}P NMR spectra for the reaction over time. The changes in yield over time for all phosphate containing species are shown in Supporting Figure 133, 135 and 137. The mean experimental results with the standard deviation of each experimental data point from the triplicate experiments are shown in Supporting Figure 138.

S3.16.1 Experiment 1 - 3.25 mmol glycerol + 0.13 mmol imidazole phosphate + 0.13 mmol Gly-Gly-His

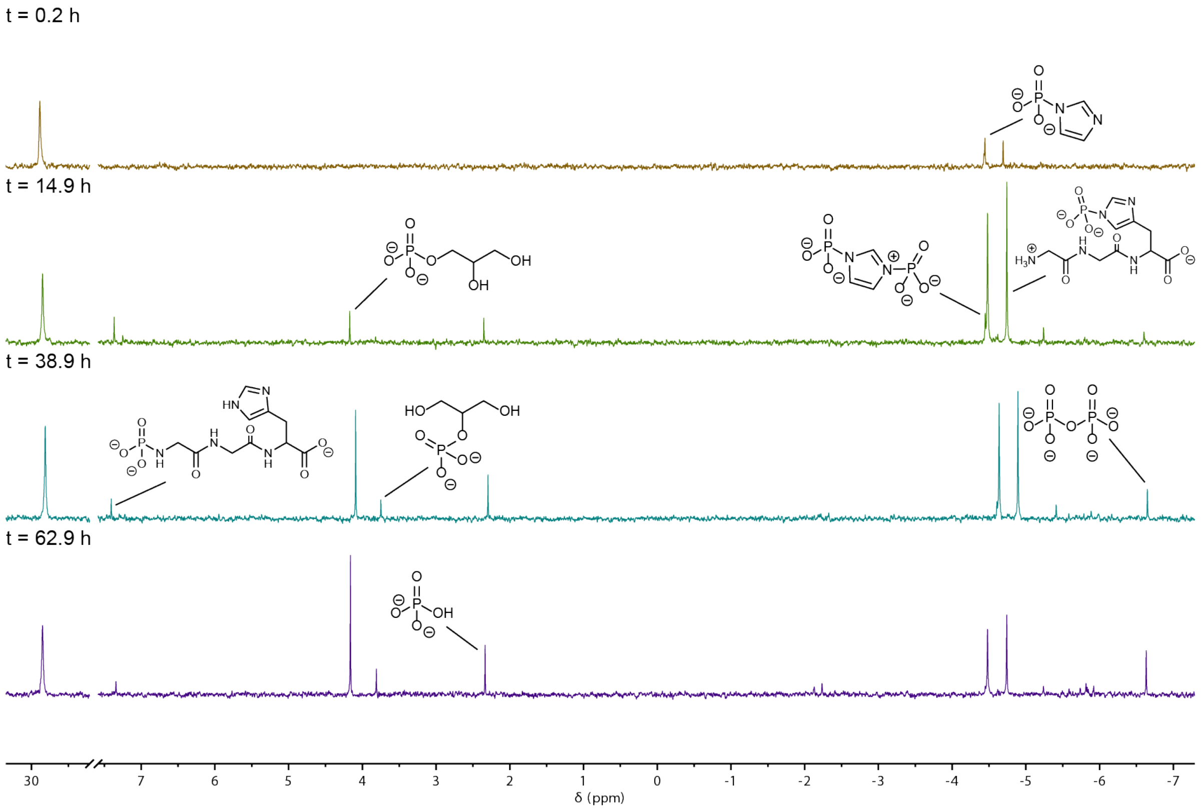

Supporting Figure 132: Representative ^{31}P-NMR spectra over time for the reaction of 0.13 mmol of calcium imidazole phosphate, 3.25 mmol of glycerol and 0.13 mmol of Gly-Gly-His at pH 7.5 and 22 °C.

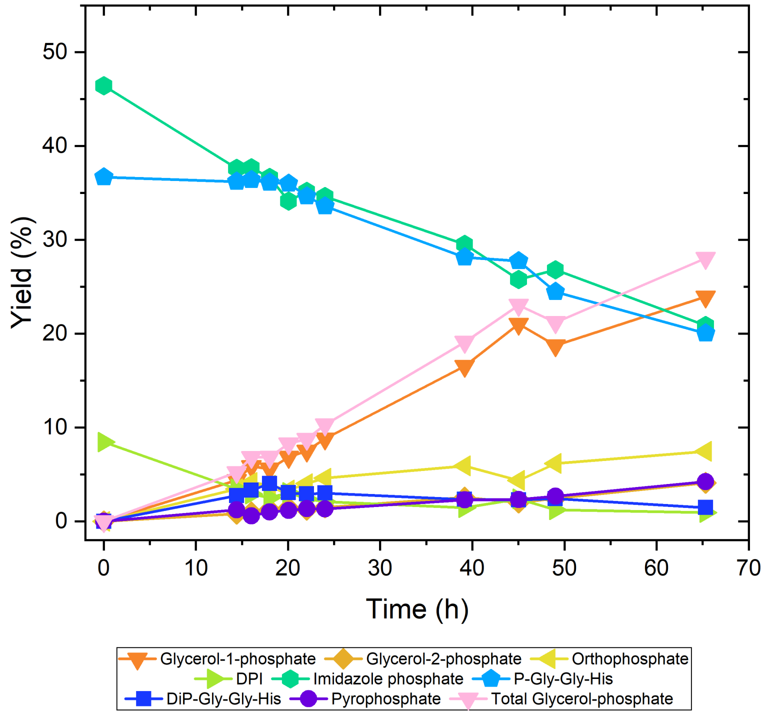

Supporting Figure 133: Changes in yield over time for the reaction of 0.13 mmol calcium imidazole phosphate, 3.25 mmol glycerol and 0.13 mmol Gly-Gly-His at pH 7.5 and 22 °C. DPI = Diphosphoimidazole. The first time point includes phosphorylation that took place in the freezer at -20 °C and thus the yields of imidazole phosphate, diphosphoimidazole and P-Gly-Gly-His are for this time point off.

S3.16.2 *Experiment 2 - 3.25 mmol glycerol + 0.13 mmol imidazole phosphate + 0.13 mmol Gly-Gly-His*

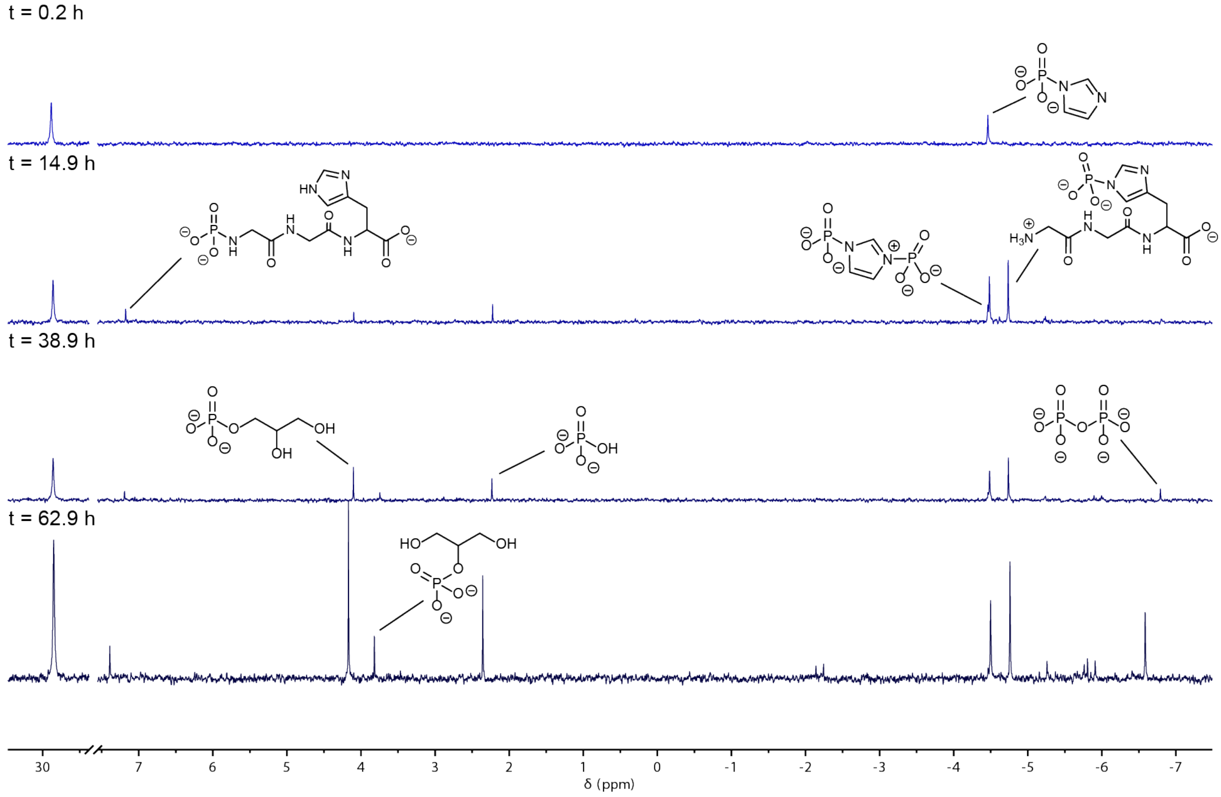

Supporting Figure 134: Representative ^{31}P-NMR spectra over time for the reaction of 0.13 mmol of calcium imidazole phosphate, 3.25 mmol of glycerol and 0.13 mmol of Gly-Gly-His at pH 7.5 and 22 °C.

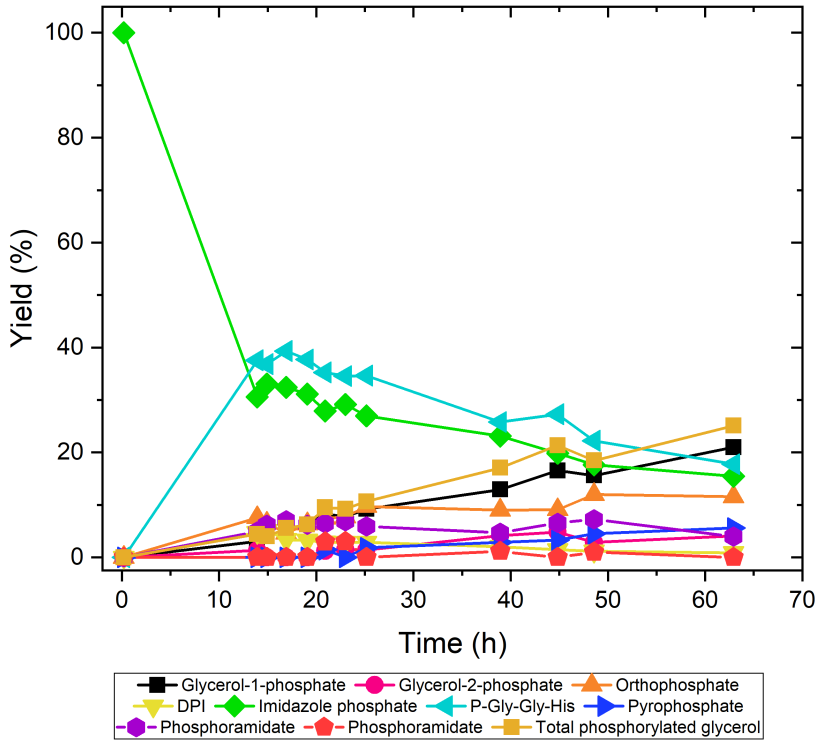

Supporting Figure 135: Changes in yield over time for the reaction of 0.13 mmol calcium imidazole phosphate, 3.25 mmol glycerol and 0.13 mmol Gly-Gly-His at pH 7.5 and 22 °C. DPI = Diphosphoimidazole.

S3.16.3 *Experiment 3 - 3.25 mmol glycerol + 0.13 mmol imidazole phosphate + 0.13 mmol Gly-Gly-His*

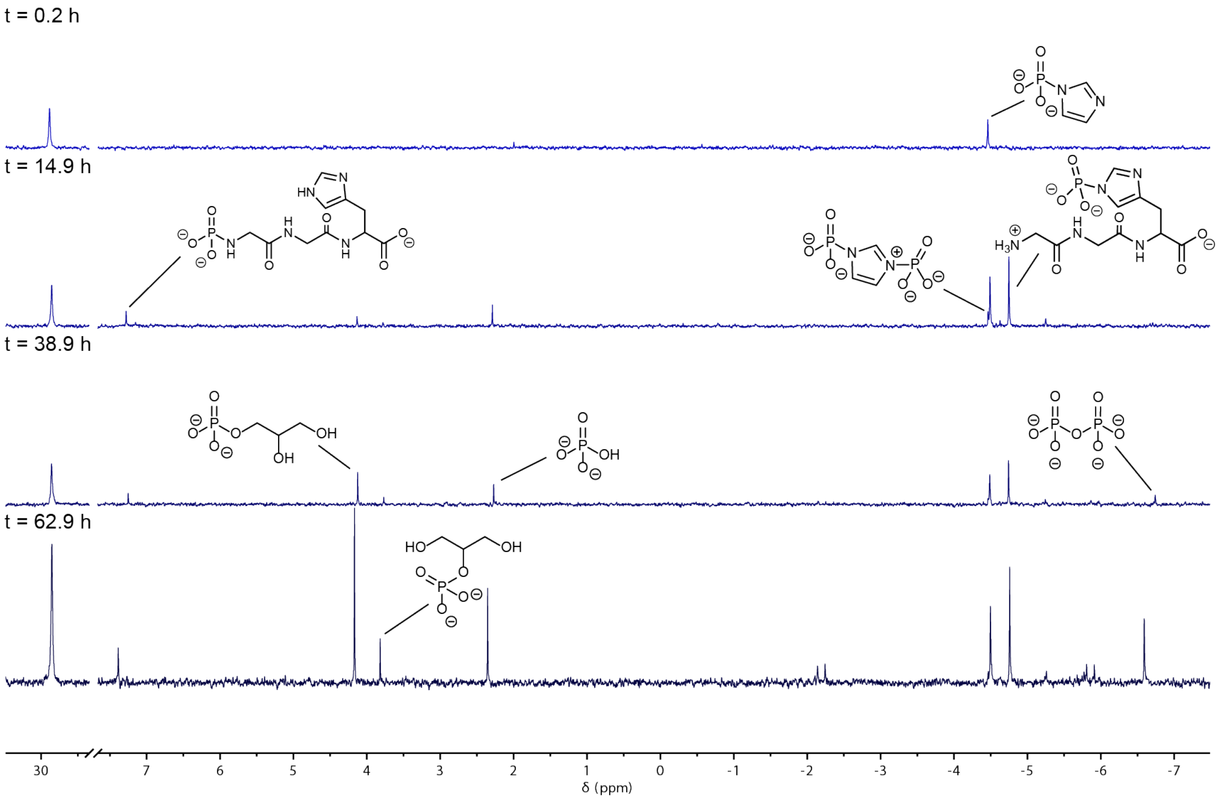

Supporting Figure 136: Representative ^{31}P-NMR spectra over time for the reaction of 0.13 mmol of calcium imidazole phosphate, 3.25 mmol of glycerol and 0.13 mmol of Gly-Gly-His at pH 7.5 and 22 °C.

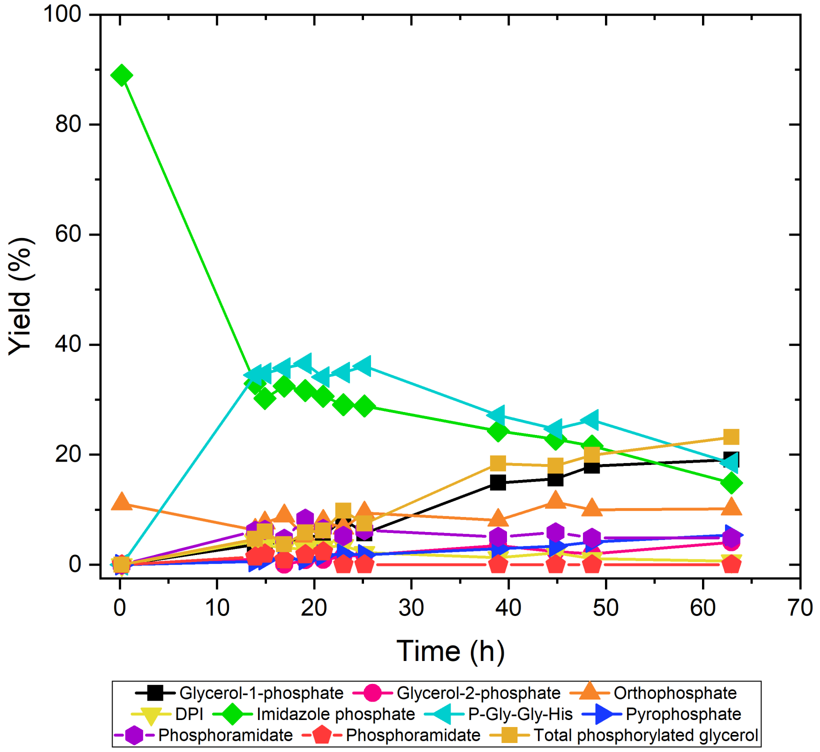

Supporting Figure 137: Changes in yield over time for the reaction of 0.13 mmol calcium imidazole phosphate, 3.25 mmol glycerol and 0.13 mmol Gly-Gly-His at pH 7.5 and 22 °C. DPI = Diphosphoimidazole.

S3.16.4 *Combined results for 3.25 mmol glycerol + 0.13 mmol imidazole phosphate + 0.13 mmol Gly-Gly-His*

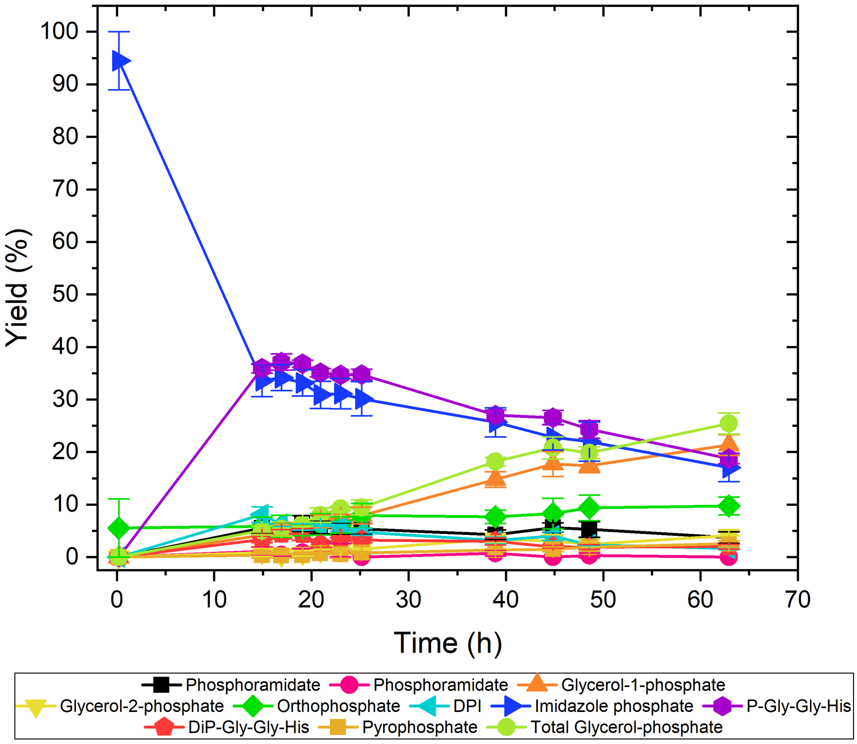

Supporting Figure 138: Changes in yield over time for the reaction of 0.13 mmol calcium imidazole phosphate, 3.25 mmol glycerol and 0.13 mmol Gly-Gly-His at pH 7.5 and 22 °C. DPI = Diphosphoimidazole. These data are the mean values and standard deviation based upon triplicate experiments.

S3.16.5 *Characterisation of phosphorylated Gly-Gly-His intermediate*

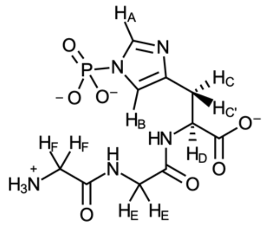

The phosphorylated Gly-Gly-His intermediate was characterised *in situ*. A solution of 50 mM Gly-Gly-His and 50 mM calcium imidazole phosphate in 0.5 mL 0.5 M MOPS buffer at pH 7.5 in 9 : 1 H_2O : D_2O containing 0.1 M citric acid and 50 mM HMPA internal standard was prepared according to the procedure in Section S2.2 and S2.4.

^{31}P NMR (202.46 MHz, 0.5 M MOPS + 0.1 M Citric Acid in 9 : 1 H_2O : D_2O at pH 7.5 and 22 °C): *phosphorylated Gly-Gly-His intermediate* δ (ppm) = - 4.89 (s, 1P). ^{1}H NMR (500.13 MHz, 0.5 M MOPS + 0.1 M Citric Acid in 9 : 1 H_2O : D_2O at pH 7.5 and 22 °C): *phosphorylated Gly-Gly-His intermediate* δ (ppm) = 8.00 (s, 1H, H_A), 7.04 (s, 1H, H_B), 4.37 (dd, 1H, H_D), 3.88 (s, 1H, H_E), 3.72 (s, 1H, H_F), 3.05 (1H, H_C), 2.91 (1H, $H_{C'}$). ^{13}C NMR (125.77 MHz, 0.5 M MOPS + 0.1 M Citric Acid in 9 : 1 H_2O : D_2O at pH 7.5 and 22 °C): *phosphorylated Gly-Gly-His intermediate*

δ (ppm) = 177.2 (s, 1C, COOH), 170.6 (s, 1C, Amide N-C=O), 169.5 (s, 1C, Amide N-C=O), 136.8 (s, 1C, imid C-H_A), 133.3 (s, 1C, imid), 118.6 (s, 1C, imid C-H_B), 54.6 (s, 1C, C-H_D), 42.7 (s, 1C, C-H_E), 41.1 (s, 1C, C-H_F), 28.6 (s, 1C, C-$H_{C+C'}$).

S3.17 Comparison of histidyl catalysts for phosphate transfer reactions

S3.17.1 Comparison of phosphorylation of glycerol by histidyl catalysts.

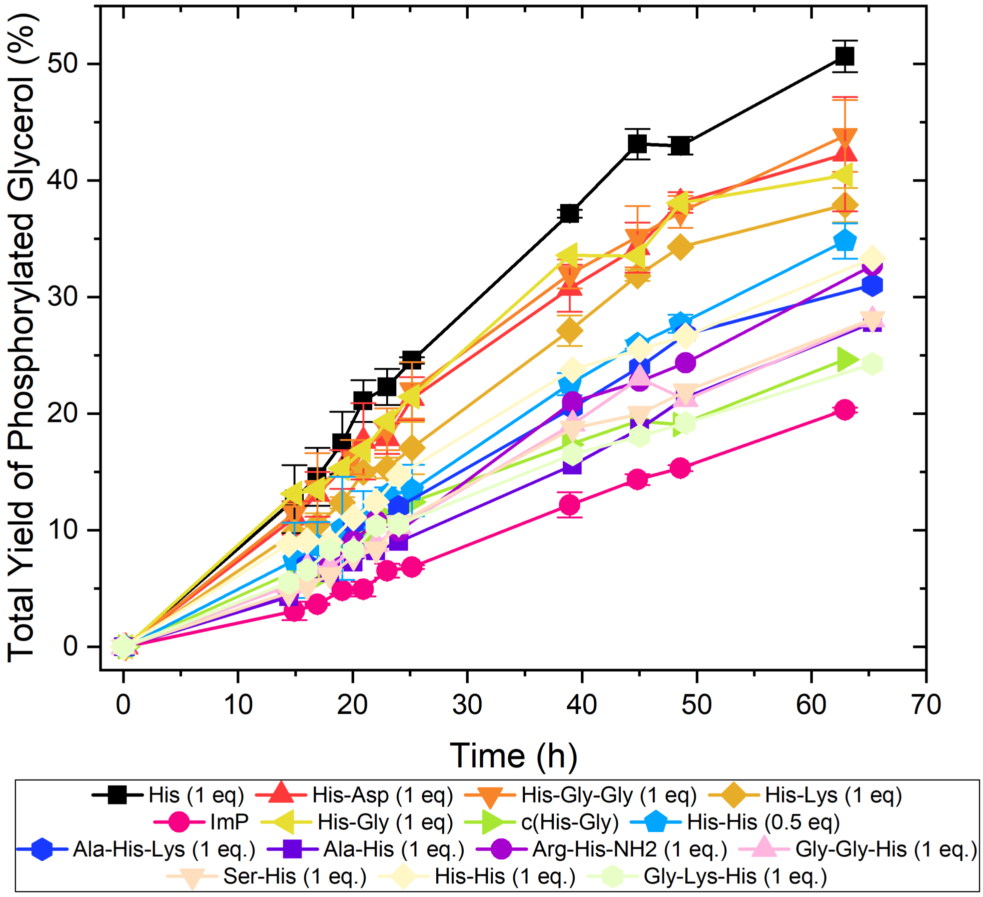

Supporting Figure 139: Changes in the yield of phosphorylated glycerol over time for the reaction of 0.13 mmol calcium imidazole phosphate, 3.25 mmol glycerol and 0.13 mmol histidyl catalyst at pH 7.5 and 22 °C.

S3.17.2 Comparison of orthophosphate production by histidyl catalysts

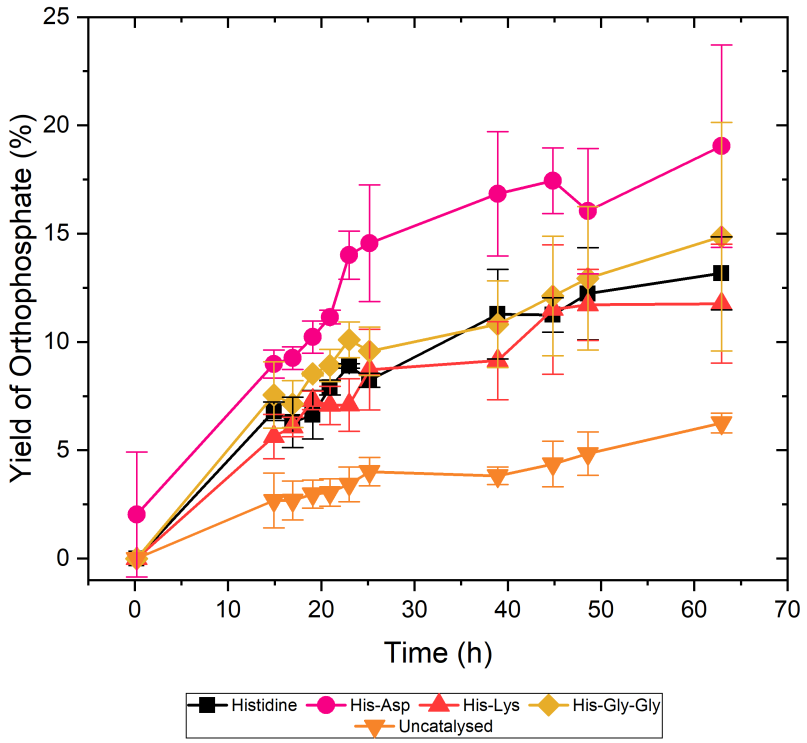

Supporting Figure 140: Changes in the yield of orthophosphate over time for the reaction of 0.13 mmol of calcium imidazole phosphate, 3.25 mmol of glycerol and 0.13 mmol of histidyl catalyst at pH 7.5 and 22 °C.

S3.17.3 Comparison of pyrophosphate production by histidyl catalysts

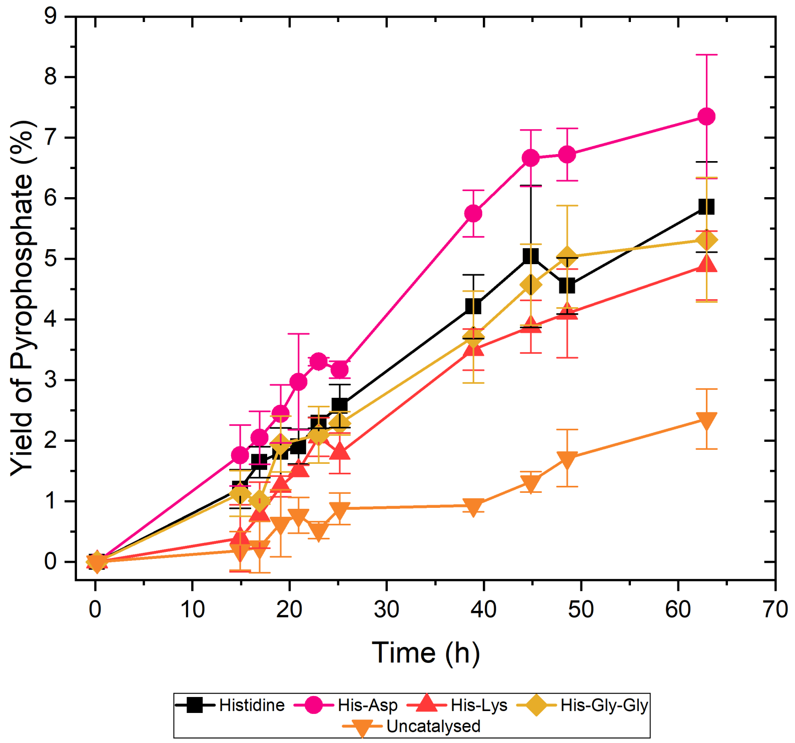

Supporting Figure 141: Changes in the yield of pyrophosphate over time for the reaction of 0.13 mmol of calcium imidazole phosphate, 3.25 mmol of glycerol and 0.13 mmol of histidyl catalyst at pH 7.5 and 22 °C.

S3.18 The quality of repeatability of the phosphate transfer reactions in the paste reactions

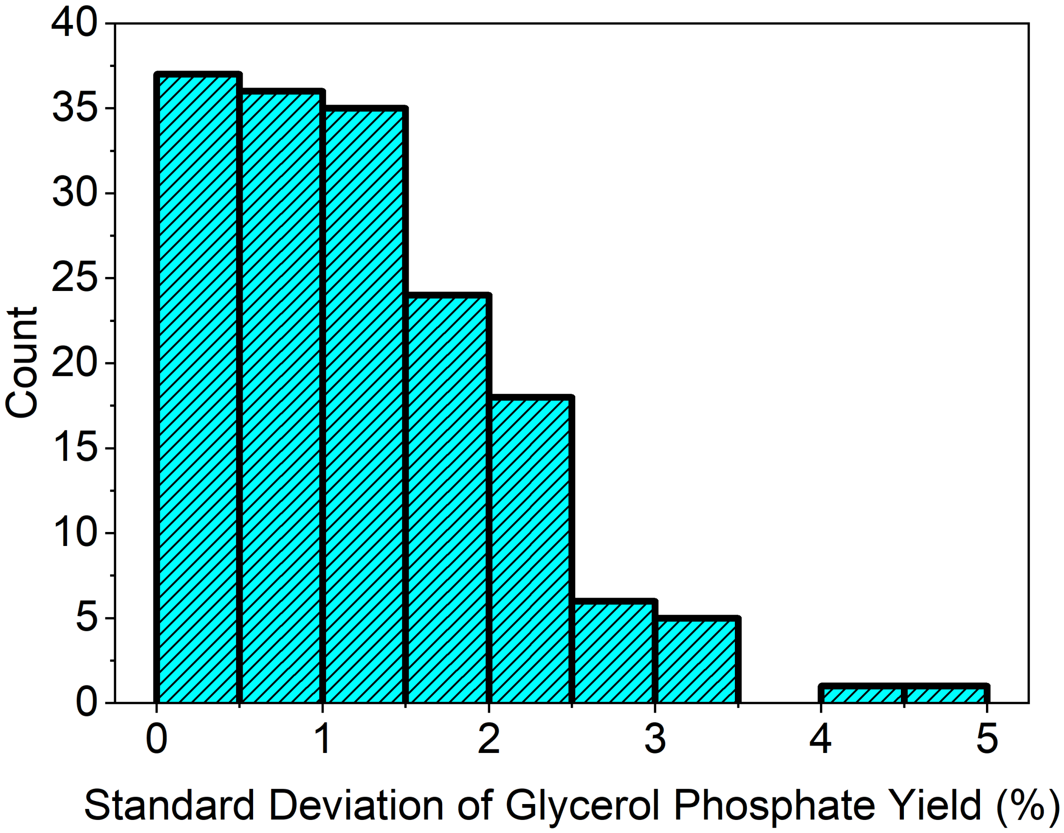

Supporting Figure 142: Histogram of the standard deviation of glycerol phosphate yield for the experiments with 0.13 mmol imidazole phosphate, 3.25 mmol of glycerol and 0.13 mmol of histidyl peptides at pH 7.5 and 22 °C.

S3.19 Comparison of histidyl catalysts for phosphate transfer reactions with 10-fold excess of Glycerol

S3.19.1 Experimental Method

24.2 mg (0.13 mmol) of calcium imidazole phosphate and 119.7 mg (1.30 mmol, 10 eq) of glycerol were dissolved in 2.0 mL of MilliQ water to give a 65 mM calcium imidazole phosphate and a 650 mM glycerol solution. The pH of the solution was adjusted to pH 7.5 with 5.0 M HCl and 5.0 M KOH solution. 20.2 mg (0.13 mmol, 1 eq) of histidine were dissolved in this solution to give a 65 mM histidine solution and again the pH of the solution was adjusted to pH 7.5 with 5.0 M HCl and 5.0 M KOH solution. The solution was then added to a petri dish and left with the lid off to dry at 22 °C for 3.5 days. The reaction was followed by periodically removing an approximately 10-20 mg sample from the paste and the sample as then placed into a -80 °C freezer. The samples were then analysed with ^{31}P-NMR and ^{1}H-NMR and ^{1}H ^{31}P HMBC NMR spectroscopy by

dissolving them in 0.5 mL of 0.5 M citric acid buffer in 9 : 1 H_2O : D_2O at pH 6.85. NMR spectra of the samples were measured within 1.0 h of dissolution in order to prevent hydrolysis from interfering with the reliability of the results.

Beyond maintaing pH of the solutions for NMR analysis, the citric acid buffer also chelated calcium ions and therefore ensured full solubilisation of all calcium phosphate salts in the sample.

S3.19.2 Phosphorylation of glycerol by imidazole phosphate with 1.00 eq. histidine catalyst

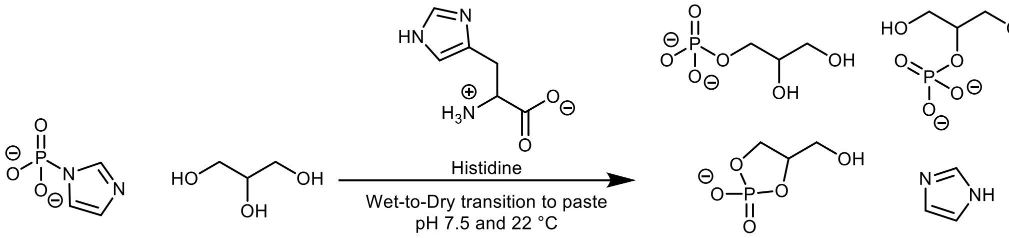

The procedure was used as detailed in Section S3.19. The experiment was repeated in duplicate. Supporting Figures 143 depicts representative ^{31}P NMR spectra for the reaction over time. The changes in yield over time for all phosphate containing species are shown in Supporting Figures 144 and 145. The mean experimental results with the standard deviation of each experimental data point from the duplicate experiments are shown in Supporting Figure 156.

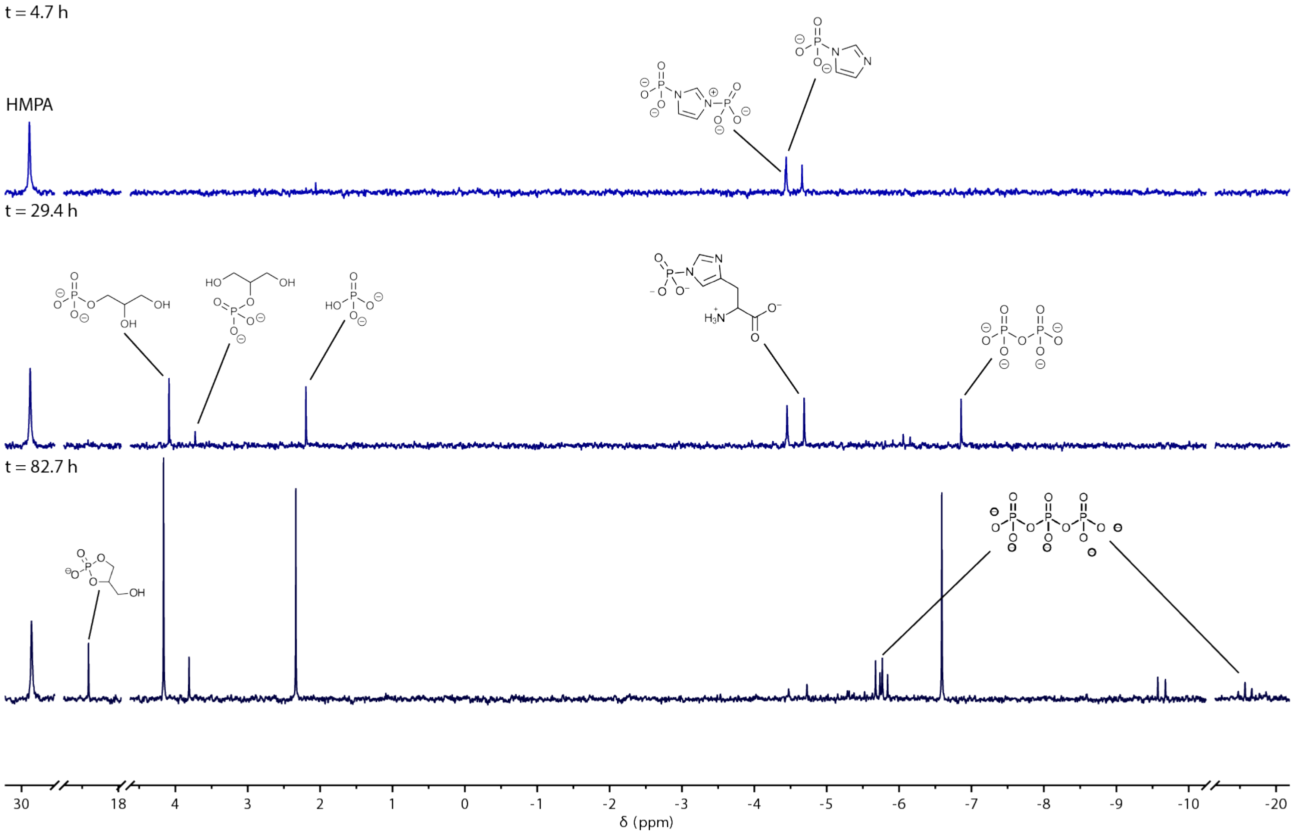

Supporting Figure 143: Representative ^{31}P-NMR spectra over time for the reaction of 0.13 mmol of calcium imidazole phosphate, 1.30 mmol of glycerol and 0.13 mmol of histidine at pH 7.5 and 22 °C.

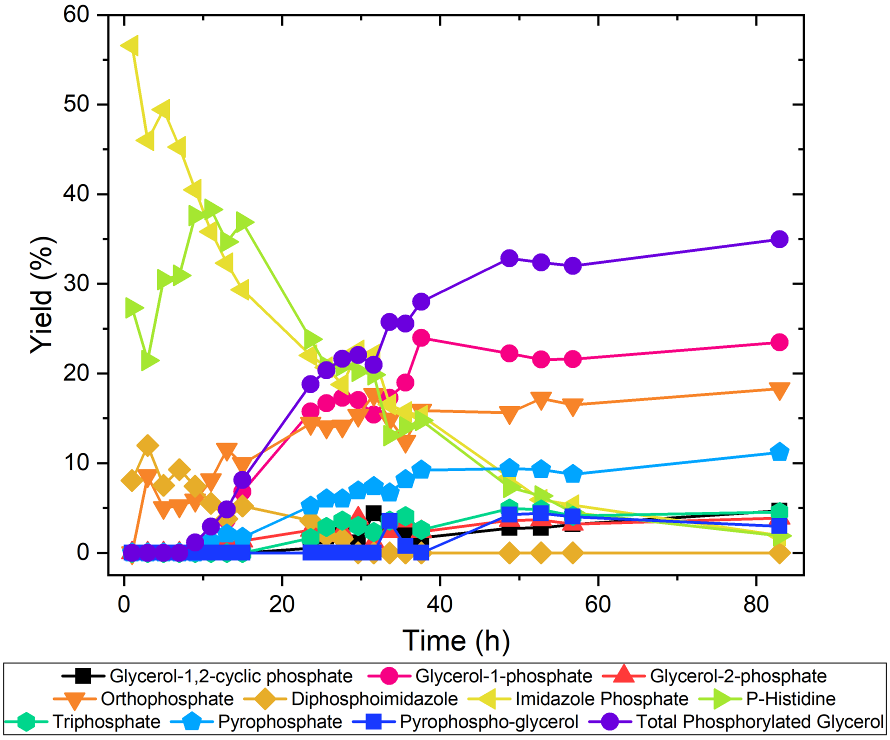

Supporting Figure 144: Changes in yield over time for the reaction of 0.13 mmol calcium imidazole phosphate, 1.30 mmol glycerol and 0.13 mmol histidine at pH 7.5 and 22 °C. The first time point includes phosphorylation that took place in the freezer at -20 °C and thus the yields of imidazole phosphate, diphosphoimidazole and P-His are for this time point off.

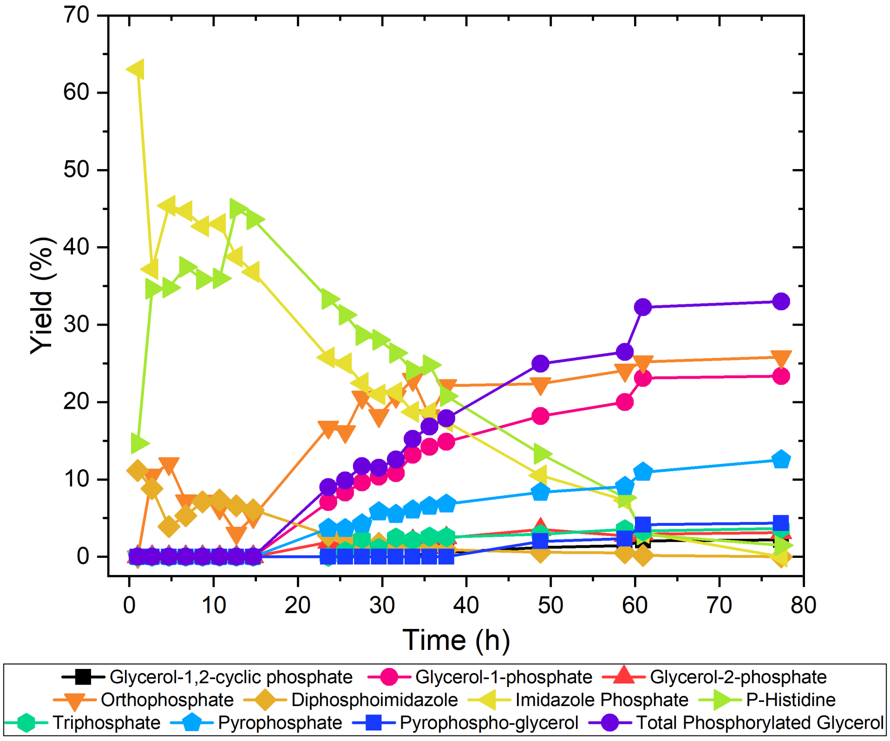

Supporting Figure 145: Repeat of changes in yield over time for the reaction of 0.13 mmol calcium imidazole phosphate, 1.30 mmol glycerol and 0.13 mmol histidine at pH 7.5 and 22 °C. The first time point includes phosphorylation that took place in the freezer at -20 °C and thus the yields of imidazole phosphate, diphosphoimidazole and P-His are for this time point off.

S3.19.3 Phosphorylation of glycerol by imidazole phosphate with 0.75 eq. histidine catalyst

An identical procedure was used as detailed in Section S3.19 but with 0.098 mmol histidine instead of 0.130 mmol histidine and prepared from 15.1 mg (0.098 mmol, 0.75 eq) of histidine. The experiment was repeated in duplicate. The changes in yield over time for all phosphate containing species are shown in Supporting Figure 146 and 147. The mean experimental results with the standard deviation of each experimental data point from the duplicate experiments are shown in Supporting Figure 156.

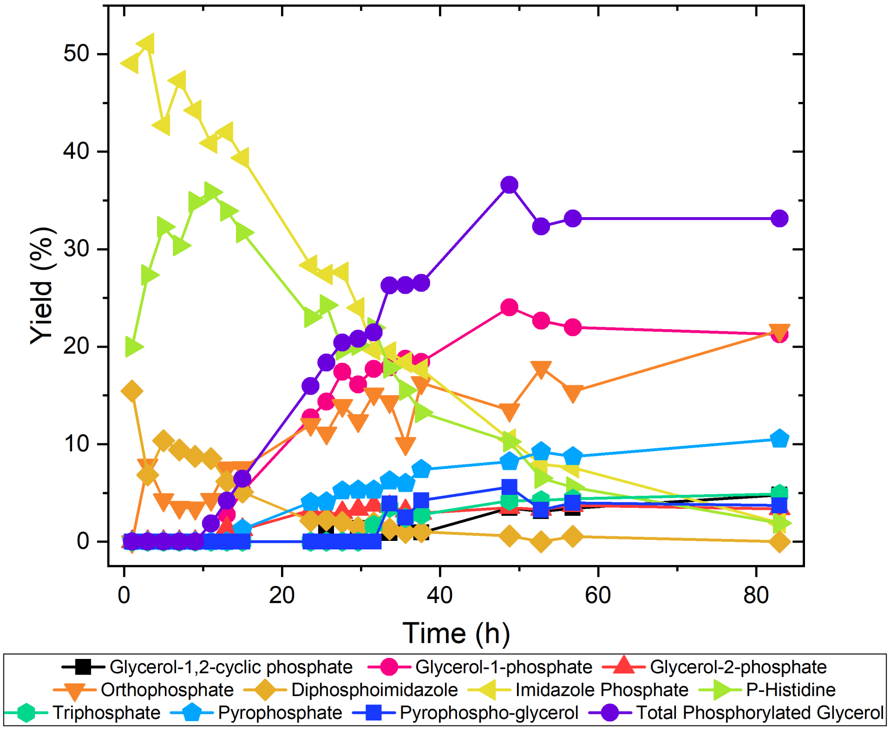

Supporting Figure 146: Changes in yield over time for the reaction of 0.13 mmol calcium imidazole phosphate, 1.30 mmol glycerol and 0.098 mmol histidine at pH 7.5 and 22 °C. The first time point includes phosphorylation that took place in the freezer at -20 °C and thus the yields of imidazole phosphate, diphosphoimidazole and P-His are for this time point off.

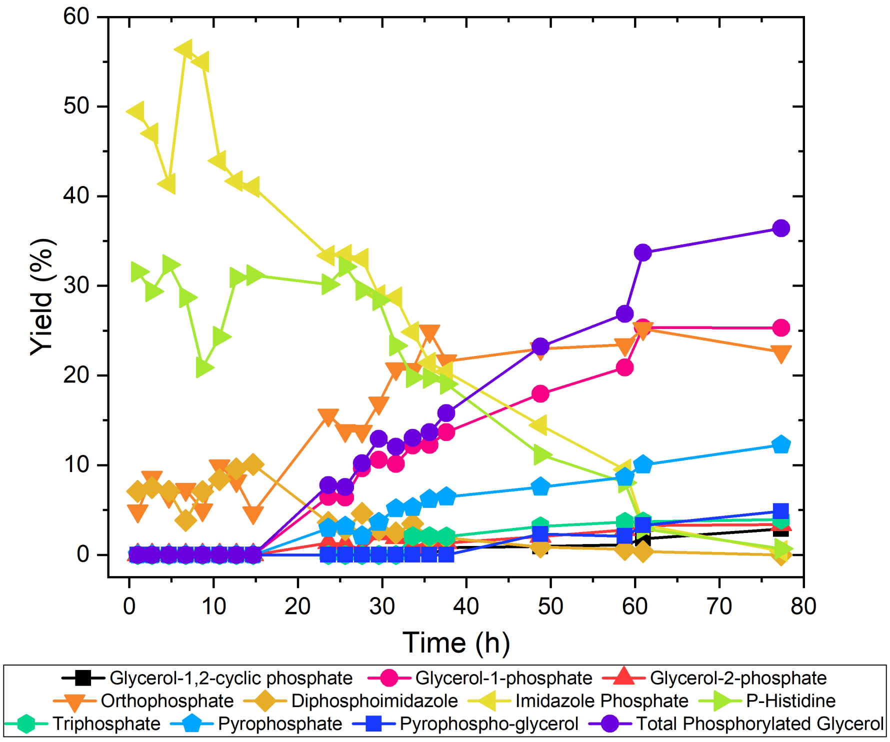

Supporting Figure 147: Repeat of changes in yield over time for the reaction of 0.13 mmol calcium imidazole phosphate, 1.30 mmol glycerol and 0.098 mmol histidine at pH 7.5 and 22 °C. The first time point includes phosphorylation that took place in the freezer at -20 °C and thus the yields of imidazole phosphate, diphosphoimidazole and P-His are for this time point off.

S3.19.4 Phosphorylation of glycerol by imidazole phosphate with 0.50 eq. histidine catalyst

An identical procedure was used as detailed in Section S3.19 but with 0.065 mmol histidine instead of 0.130 mmol histidine and prepared from 10.1 mg (0.065 mmol, 0.50 eq) of histidine. The experiment was repeated in duplicate. The changes in yield over time for all phosphate containing species are shown in Supporting Figure 148 and 149. The mean experimental results with the standard deviation of each experimental data point from the duplicate experiments are shown in Supporting Figure 156.

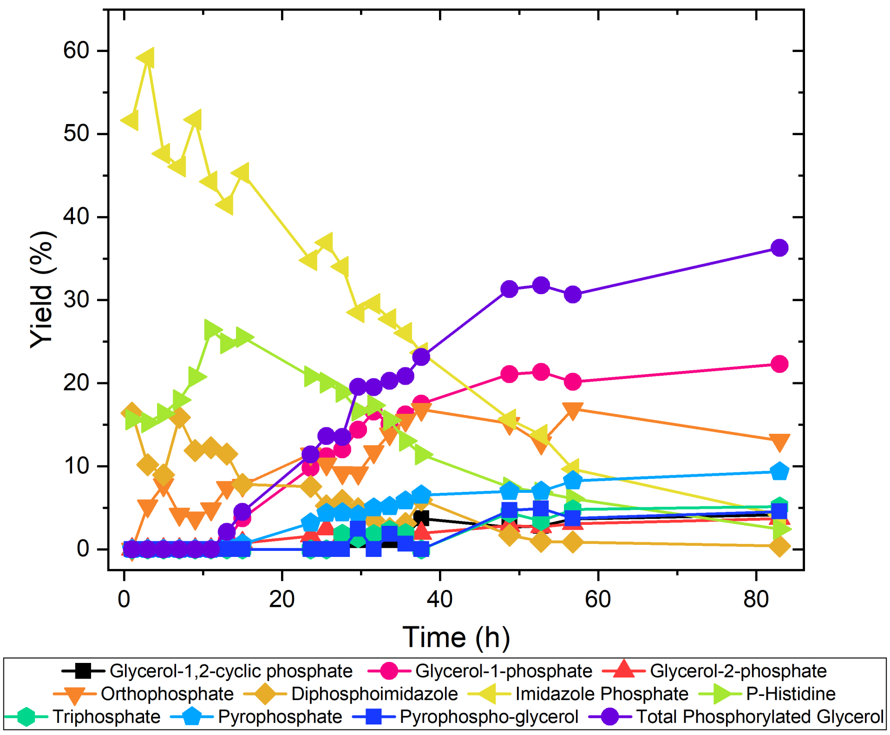

Supporting Figure 148: Changes in yield over time for the reaction of 0.13 mmol calcium imidazole phosphate, 1.30 mmol glycerol and 0.065 mmol histidine at pH 7.5 and 22 °C. The first time point includes phosphorylation that took place in the freezer at -20 °C and thus the yields of imidazole phosphate, diphosphoimidazole and P-His are for this time point off.

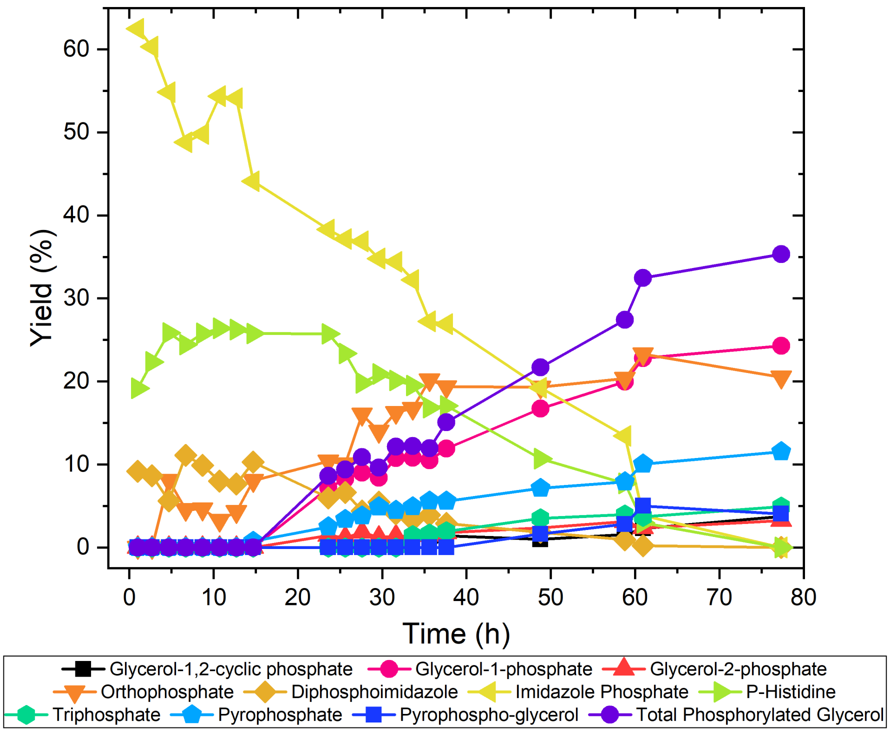

Supporting Figure 149: Repeat of changes in yield over time for the reaction of 0.13 mmol calcium imidazole phosphate, 1.30 mmol glycerol and 0.065 mmol histidine at pH 7.5 and 22 °C. The first time point includes phosphorylation that took place in the freezer at -20 °C and thus the yields of imidazole phosphate, diphosphoimidazole and P-His are for this time point off.

S3.19.5 Phosphorylation of glycerol by imidazole phosphate with 0.25 eq. histidine catalyst

An identical procedure was used as detailed in Section S3.19 but with 0.033 mmol histidine instead of 0.130 mmol histidine and prepared from 5.0 mg (0.033 mmol, 0.25 eq) of histidine. The experiment was repeated in duplicate. The changes in yield over time for all phosphate containing species are shown in Supporting Figure 150 and 151. The mean experimental results with the standard deviation of each experimental data point from the duplicate experiments are shown in Supporting Figure 156.

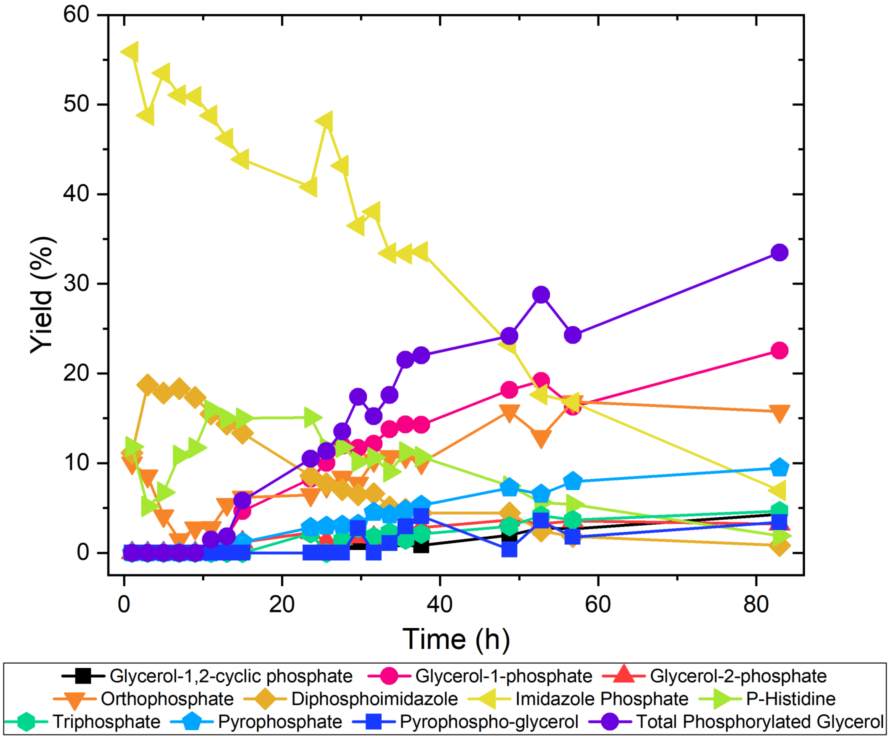

Supporting Figure 150: Changes in yield over time for the reaction of 0.13 mmol calcium imidazole phosphate, 1.30 mmol glycerol and 0.033 mmol histidine at pH 7.5 and 22 °C. The first time point includes phosphorylation that took place in the freezer at -20 °C and thus the yields of imidazole phosphate, diphosphoimidazole and P-His are for this time point off.

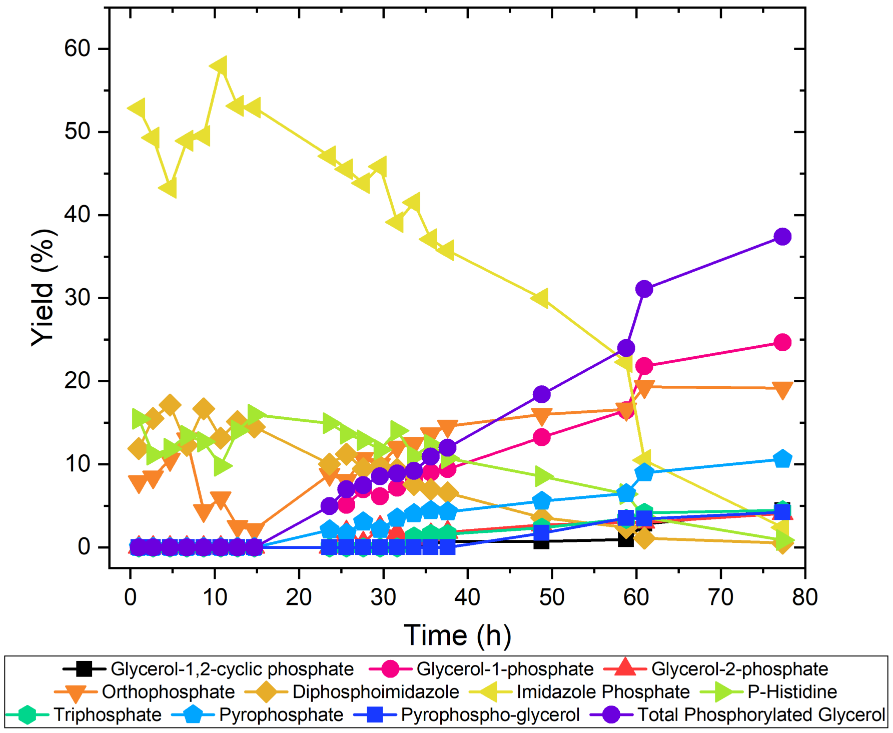

Supporting Figure 151: Repeat of changes in yield over time for the reaction of 0.13 mmol calcium imidazole phosphate, 1.30 mmol glycerol and 0.033 mmol histidine at pH 7.5 and 22 °C. The first time point includes phosphorylation that took place in the freezer at -20 °C and thus the yields of imidazole phosphate, diphosphoimidazole and P-His are for this time point off.

S3.19.6 Uncatalysed phosphorylation of glycerol by imidazole phosphate

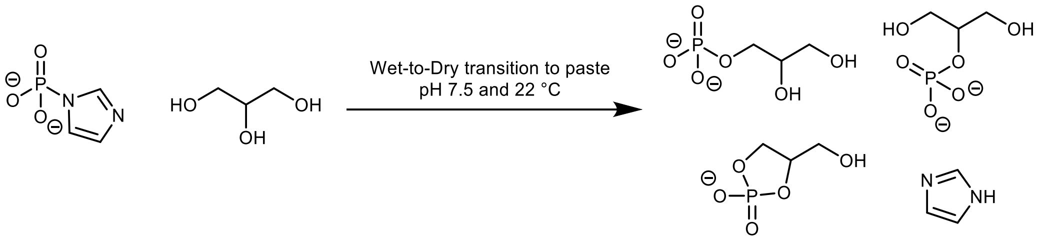

An identical procedure was used as detailed in Section S3.19 but with no histidine catalyst added. The experiment was repeated in quadruplicate. The changes in yield over time for all phosphate containing species are shown in Supporting Figure 152, 153, 154 and 155. The mean experimental results with the standard deviation of each experimental data point from the duplicate experiments are shown in Supporting Figure 156.

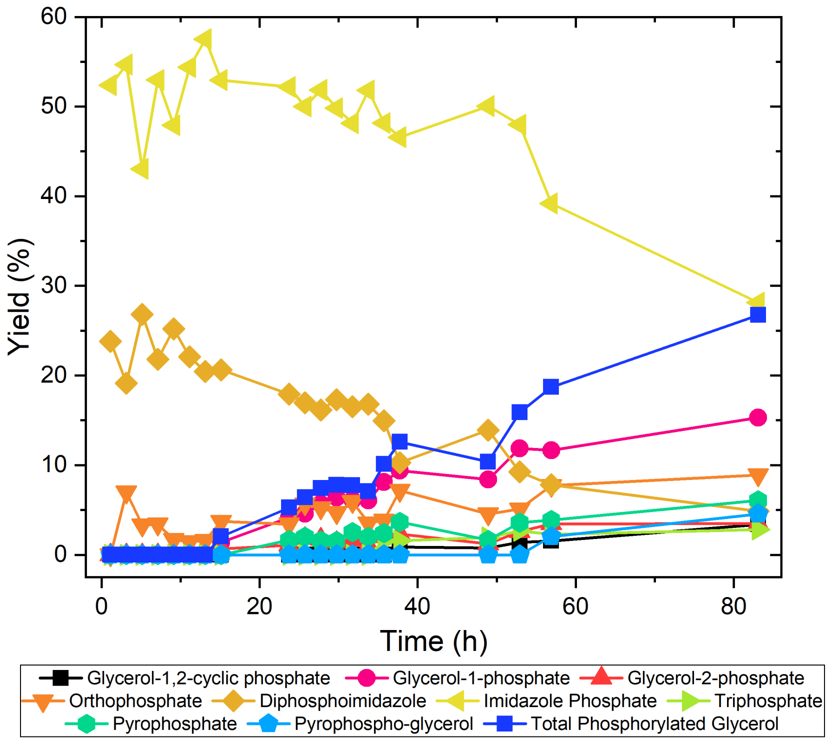

Supporting Figure 152: Changes in yield over time for the reaction of 0.13 mmol calcium imidazole phosphate and 1.30 mmol glycerol at pH 7.5 and 22 °C. The first time point includes phosphorylation that took place in the freezer at -20 °C and thus the yields of imidazole phosphate, diphosphoimidazole and P-His are for this time point off.

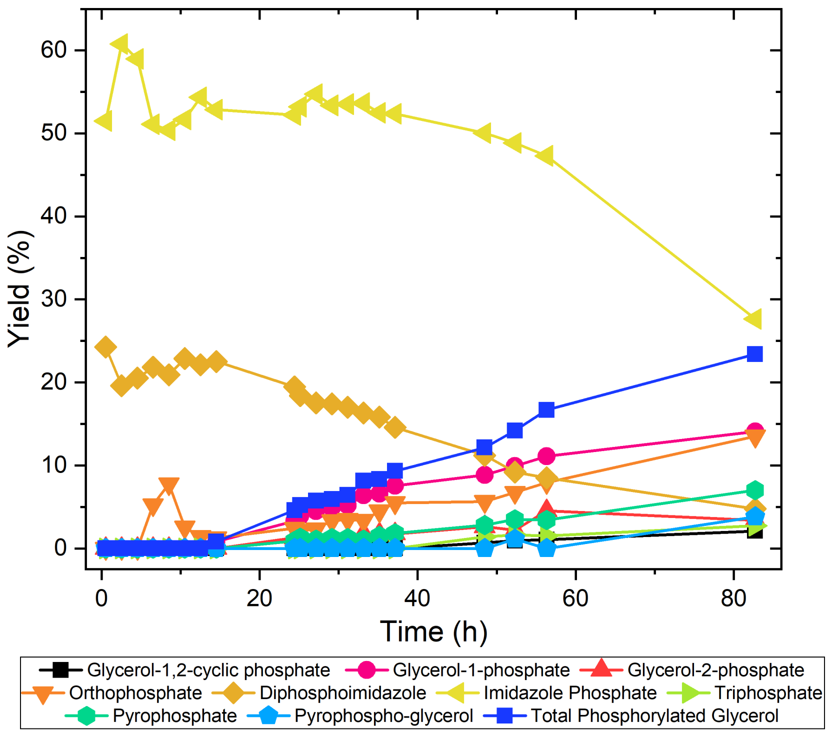

Supporting Figure 153: Repeat 1 of changes in yield over time for the reaction of 0.13 mmol calcium imidazole phosphate and 1.30 mmol glycerol at pH 7.5 and 22 °C. The first time point includes phosphorylation that took place in the freezer at -20 °C and thus the yields of imidazole phosphate, diphosphoimidazole and P-His are for this time point off.

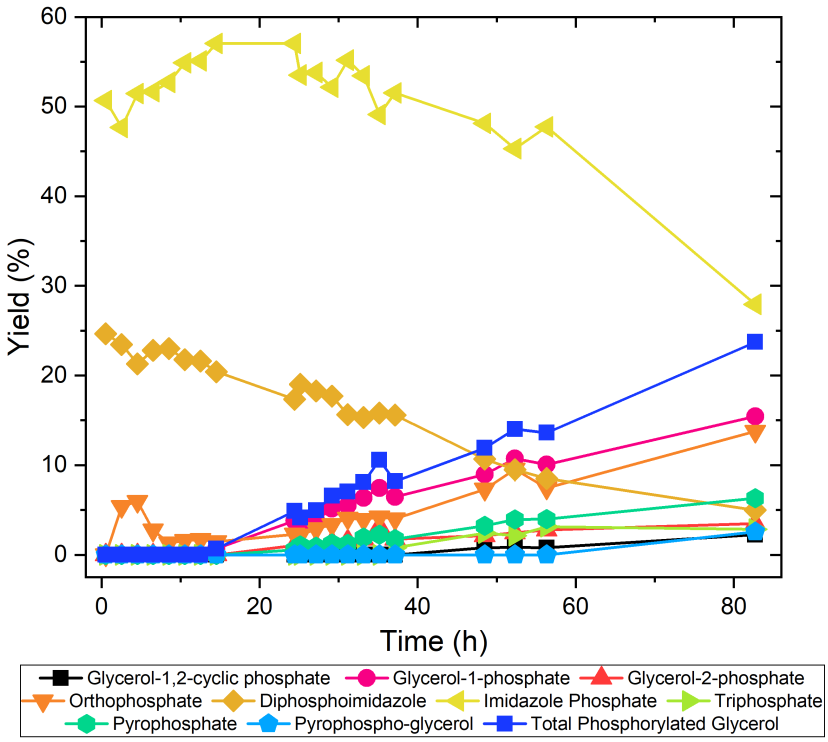

Supporting Figure 154: Repeat 2 of changes in yield over time for the reaction of 0.13 mmol calcium imidazole phosphate and 1.30 mmol glycerol at pH 7.5 and 22 °C. The first time point includes phosphorylation that took place in the freezer at -20 °C and thus the yields of imidazole phosphate, diphosphoimidazole and P-His are for this time point off.

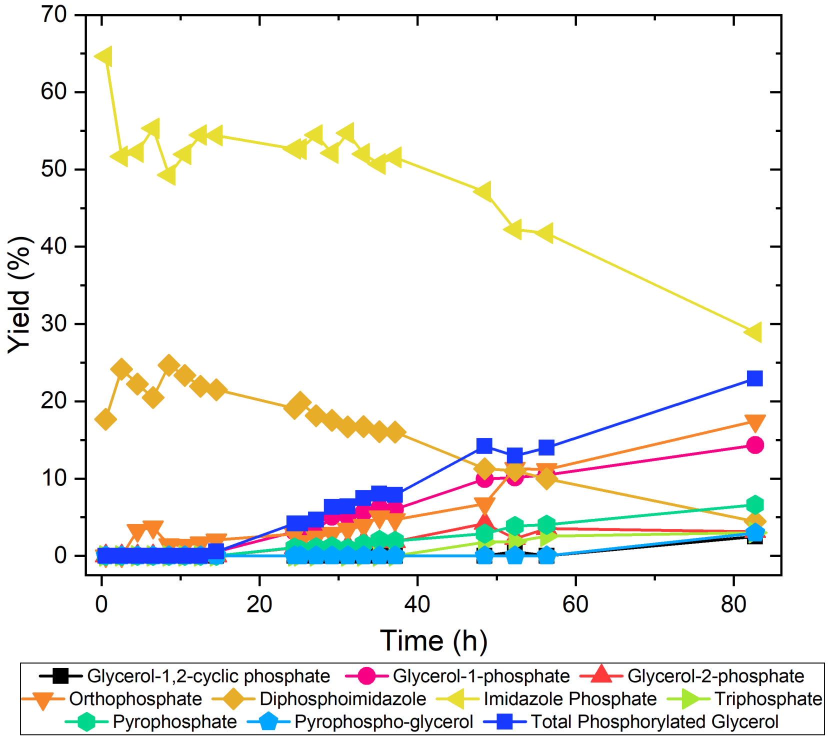

Supporting Figure 155: Repeat 3 of changes in yield over time for the reaction of 0.13 mmol calcium imidazole phosphate and 1.30 mmol glycerol at pH 7.5 and 22 °C. The first time point includes phosphorylation that took place in the freezer at -20 °C and thus the yields of imidazole phosphate, diphosphoimidazole and P-His are for this time point off.

S3.19.7 Comparison of different equivalents of histidine catalyst upon the phosphorylation of glycerol by imidazole phosphate.

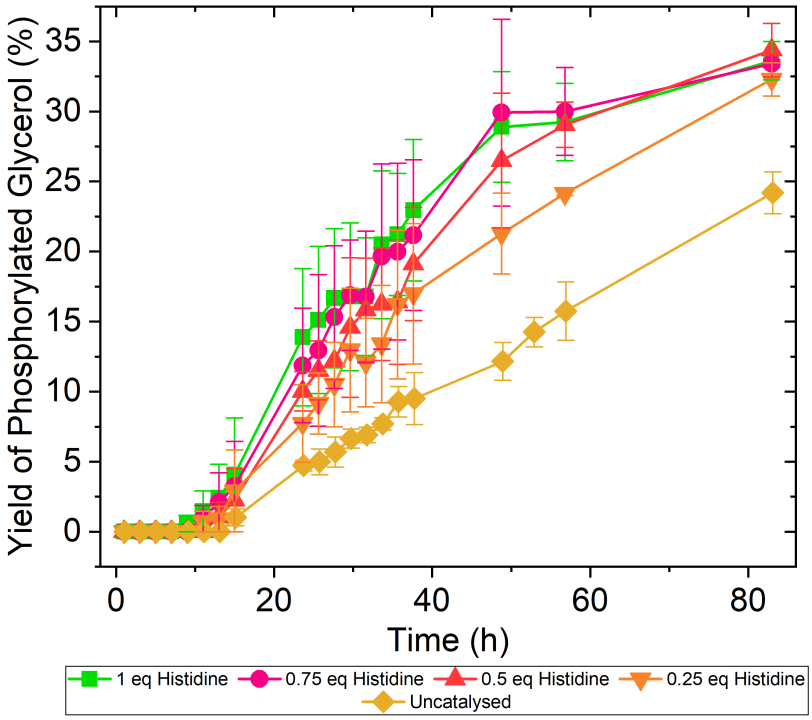

Supporting Figure 156: The effect of different equivalents of histidine catalyst upon the catalysed phosphorylation of glycerol by imidazole phosphate. The changes in yield of phosphorylated glycerol over time whereby the yields of all phosphorylated glycerol species (glycerol-1-phosphate, glycerol-2-phopsphate and cyclo-glycerol-1,2-phosphate) were summated. The reaction was performed by starting from solutions containing 0.130 mmol imidazole phosphate, 1.30 mmol of glycerol (10 eq.) and 0.130 mmol (1.00 eq.) / 0.098 mmol (0.75 eq.) / 0.065 mmol (0.50 eq.) / 0.033 mmol (0.25 eq.) histidine at pH 7.5 and 22 °C. The uncatalysed reaction is also shown. The reactions were repeated in duplicate or quadruplicate with each data point being the mean value and error bars represent the standard deviation.

S3.20 Comparison of histidyl catalysts for phosphate transfer reactions with 5-fold excess of Glycerol

S3.20.1 Experimental Method

24.2 mg (0.13 mmol) of calcium imidazole phosphate and 59.9 mg (0.65 mmol, 5 eq.) of glycerol were dissolved in 2.0 mL of MilliQ water to give a 65 mM calcium imidazole phosphate and a 325 mM glycerol solution. The pH of the solution was adjusted to pH 7.5 with 5.0 M HCl and 5.0 M KOH solution. 20.2 mg (0.13 mmol, 1 eq.) of histidine were dissolved in this solution to give a 65 mM histidine solution and again the pH of the solution was adjusted to pH 7.5 with 5.0 M HCl and 5.0 M KOH solution. The solution was then added to a petri dish and left with the lid off to dry at 22 °C for 41 h. The reaction was followed by periodically

removing an approximately 10-20 mg sample from the paste and then analysing the sample with ^{31}P-NMR and ^{1}H-NMR spectroscopy by dissolving them in 0.5 mL of 0.5 M citric acid buffer in 9 : 1 H_2O : D_2O at pH 6.85. NMR spectra of the samples were measured within 1.0 h of dissolution in order to prevent hydrolysis from interfering with the reliability of the results.

Beyond maintaing pH of the solutions for NMR analysis, the citric acid buffer also chelated calcium ions and therefore ensured full solubilisation of all calcium phosphate salts in the sample.

S3.20.2 Phosphorylation of glycerol by imidazole phosphate with 1.0 eq. histidine catalyst and 5.0 eq. of glycerol

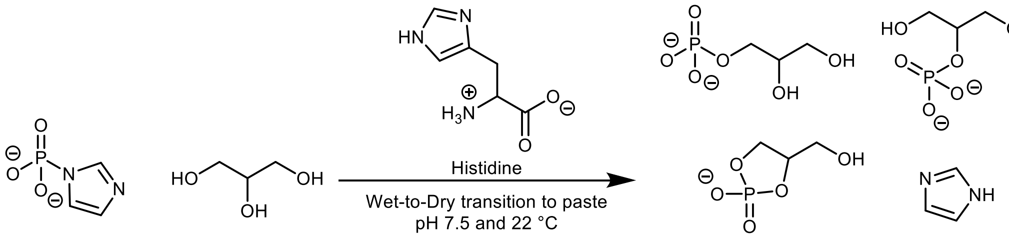

The procedure was used as detailed in Section S3.20.1. Supporting Figures 157 depicts representative ^{31}P NMR spectra for the reaction over time. The changes in yield over time for all phosphate containing species are shown in Supporting Figures 158.

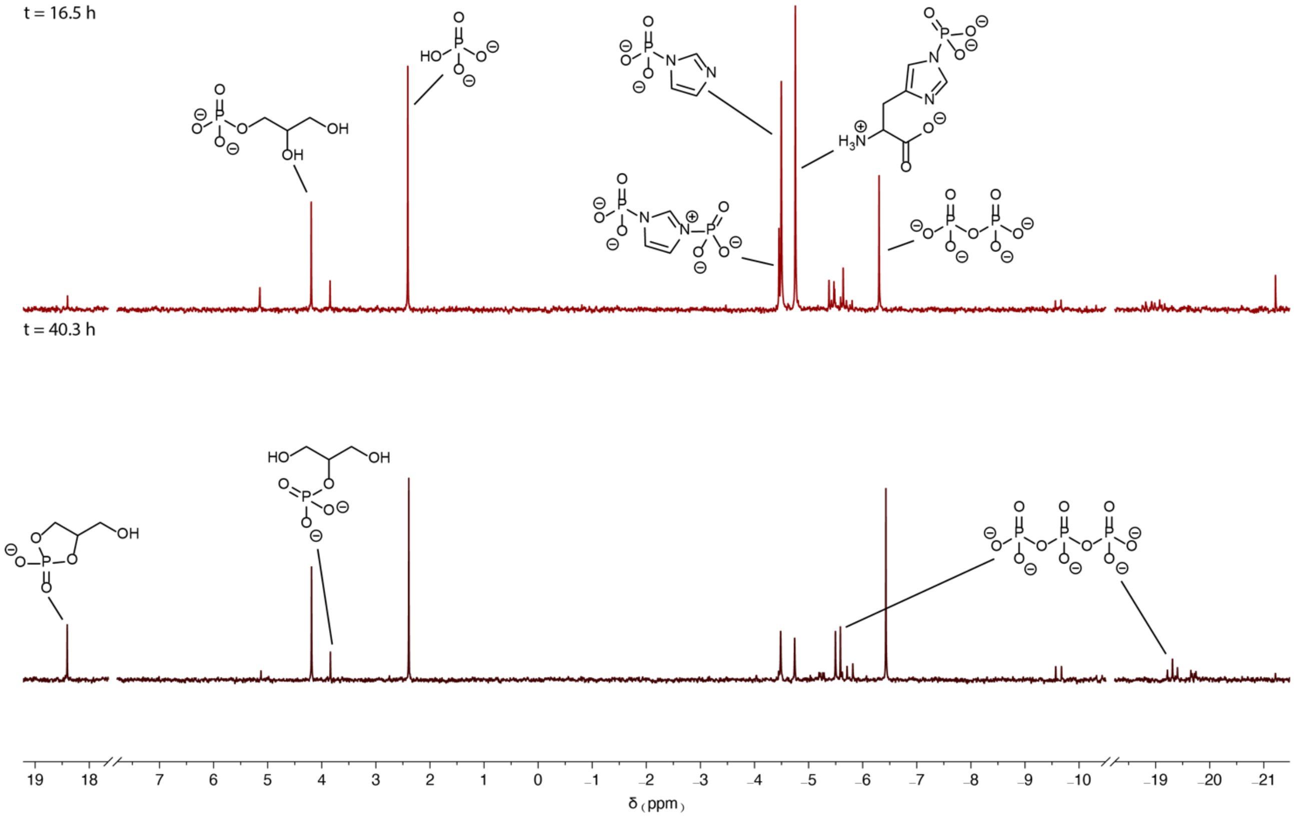

Supporting Figure 157: Representative ^{31}P-NMR spectra over time for the reaction of 0.13 mmol of calcium imidazole phosphate, 0.65 mmol of glycerol and 0.13 mmol of histidine at pH 7.5 and 22 °C.

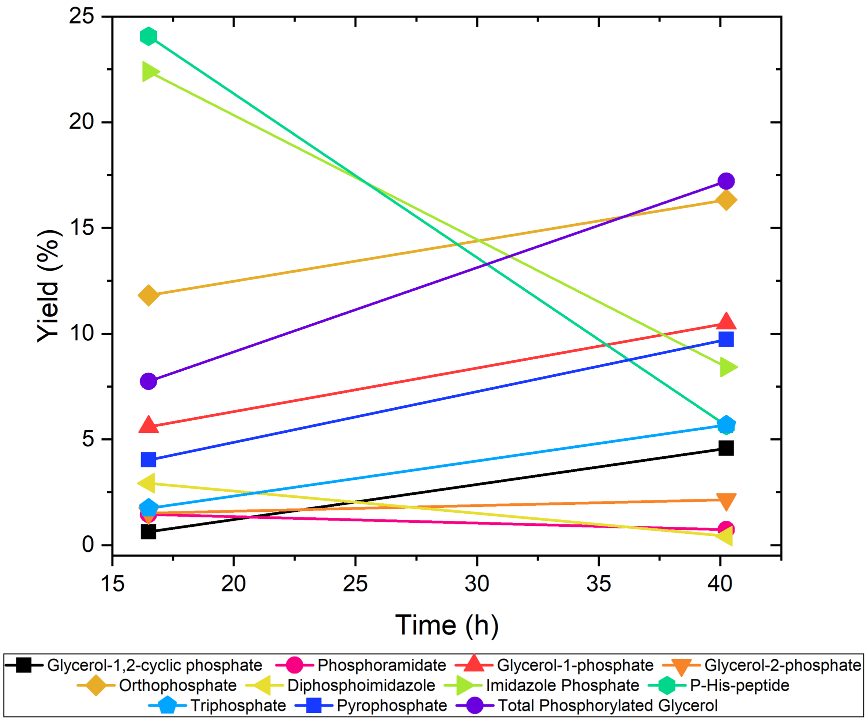

Supporting Figure 158: Changes in yield over time for the reaction of 0.13 mmol calcium imidazole phosphate, 0.65 mmol glycerol and 0.13 mmol histidine at pH 7.5 and 22 °C.

S3.20.3 Uncatalysed phosphorylation of glycerol by imidazole phosphate with 5 eq. of glycerol

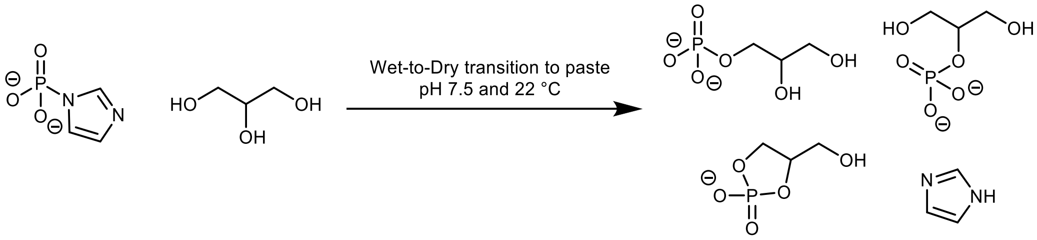

The procedure was used as detailed in Section S3.20.1 but without histidine catalyst added. Supporting Figures 159 depicts representative ^{31}P NMR spectra for the reaction over time. The changes in yield over time for all phosphate containing species are shown in Supporting Figures 160.

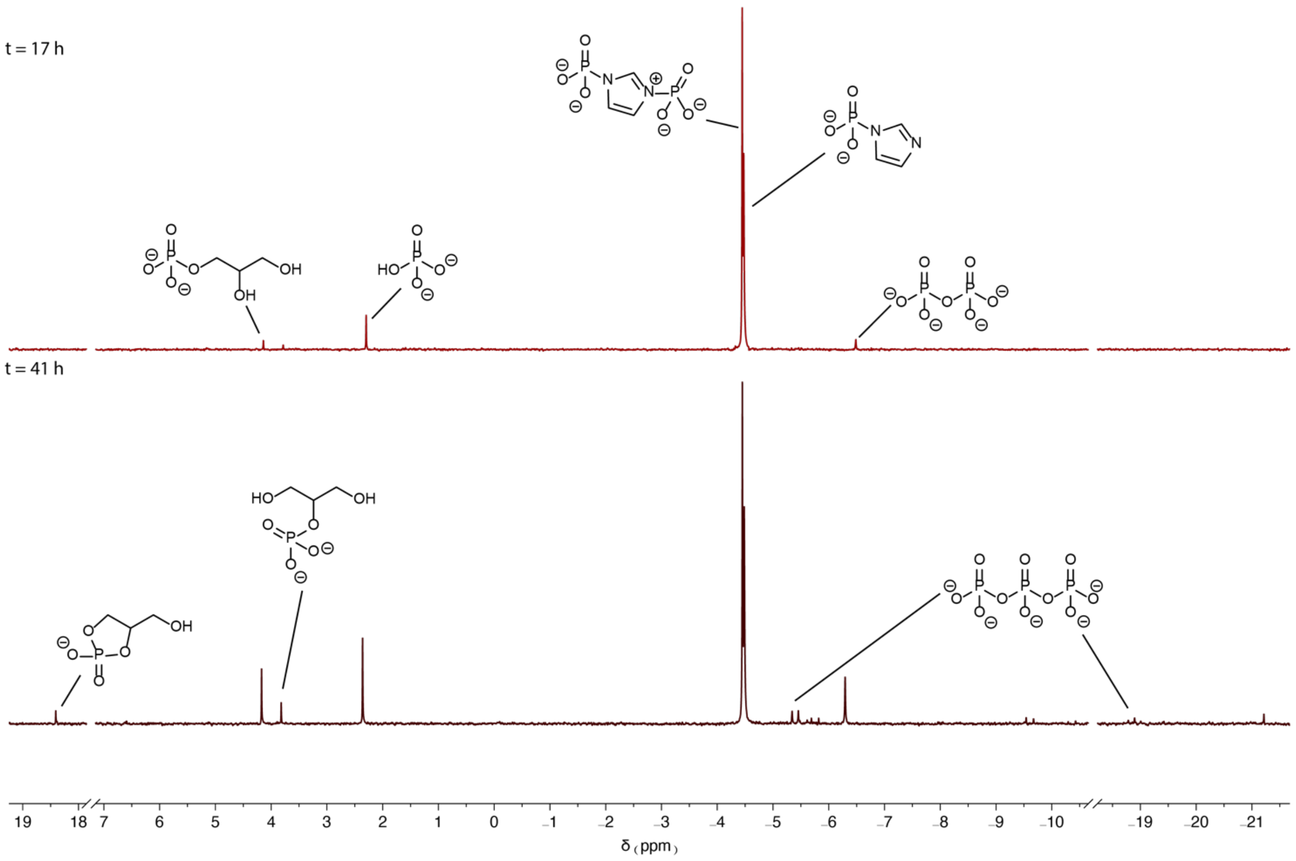

Supporting Figure 159: Representative ^{31}P-NMR spectra over time for the reaction of 0.13 mmol of calcium imidazole phosphate, 0.65 mmol of glycerol and 0.13 mmol of histidine at pH 7.5 and 22 °C.

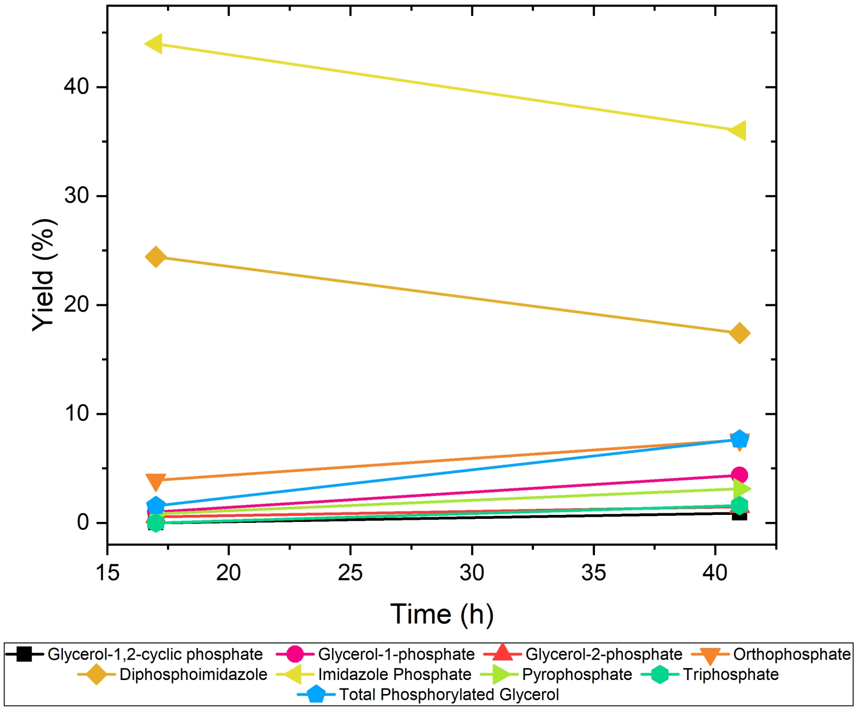

Supporting Figure 160: Changes in yield over time for the reaction of 0.13 mmol calcium imidazole phosphate, 0.65 mmol glycerol and 0.13 mmol histidine at pH 7.5 and 22 °C.

S4 Physicochemical orthophosphate cycles with histidyl catalysts (Main Text Figure 4)

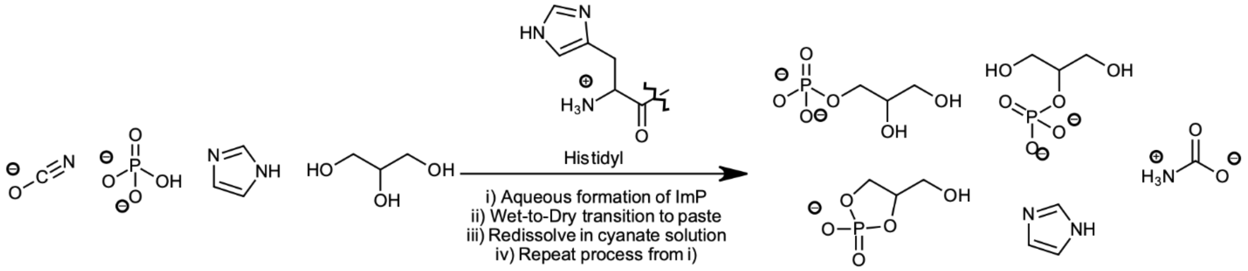

S4.1 Experimental Method

A 4 mL solution of 20 mM sodium phosphate dibasic + 500 mM glycerol + 230 mM potassium cyanate + 100 mM imidazole + 100 mM histidine was prepared by dissolving 14.2 mg (0.08 mmol) of sodium phosphate dibasic dihydrate, 184.2 mg (2.00 mmol) of glycerol, 74.4 mg (0.92 mmol) of potassium cyanate, 36.8 mg (0.40 mmol) of imidazole and 62.1 mg (0.40 mmol) of histidine in 9 : 1 H_2O : D_2O. The pH of the solution was adjusted to pH 7.3 with 5.0 M HCl and 5.0 M KOH solution. For the first wet-dry cycle, the solution was left for 24 h and then added to a petri dish and left with the lid off to dry at 22 °C for 48 h in a fume cupboard. To begin the second wet-dry cycle, the resulting paste was dissolved in 4 mL of 230 mM cyanate solution (prepared as above) in 9 : 1 H_2O : D_2O. The pH of the solution was adjusted to pH 7.3 with 5.0 M HCl and 5.0 M KOH solution. Immediately after this, the ^{31}P and ^{1}H NMR spectra were measured using 0.5 mL of this solution in order to determine the quantity of phosphorylated species formed in the first wet-dry cycle. After which the 0.5 mL of solution was recombined with the rest of the solution. The second wet-dry cycle followed the same procedure as for the first wet-dry cycle. Once the second wet-dry cycle was complete a third wet-dry cycle was initiated again using the procedure of the first wet-dry cycle.

S4.2 Wet/Dry Cycle for the phosphorylation of glycerol by imidazole phosphate with a histidine catalyst

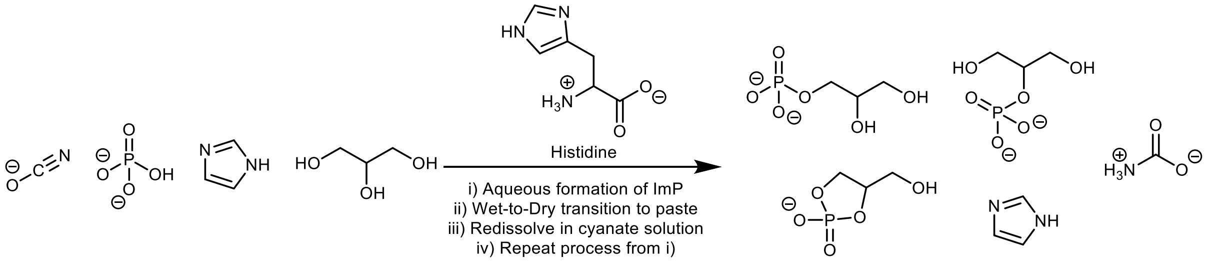

The experiment was carried out according to the procedure in S4.1. The experiment was repeated in triplicate. Supporting Figures 161, 163 and 165 depict representative ^{31}P NMR spectra after each wet-dry cycle. The changes in yield after each wet-dry cycle for all phosphate containing species are shown in Supporting Figure 162a, 164a and 166a. The change in yield of glycerol-1-phosphate and glycerol-2-phosphate after each wet-dry cycle are shown in Supporting Figure 162b, 164b and 166b.

S4.2.1 Wet/Dry cycle for the phosphorylation of glycerol catalysed by 100 mM histidine catalyst

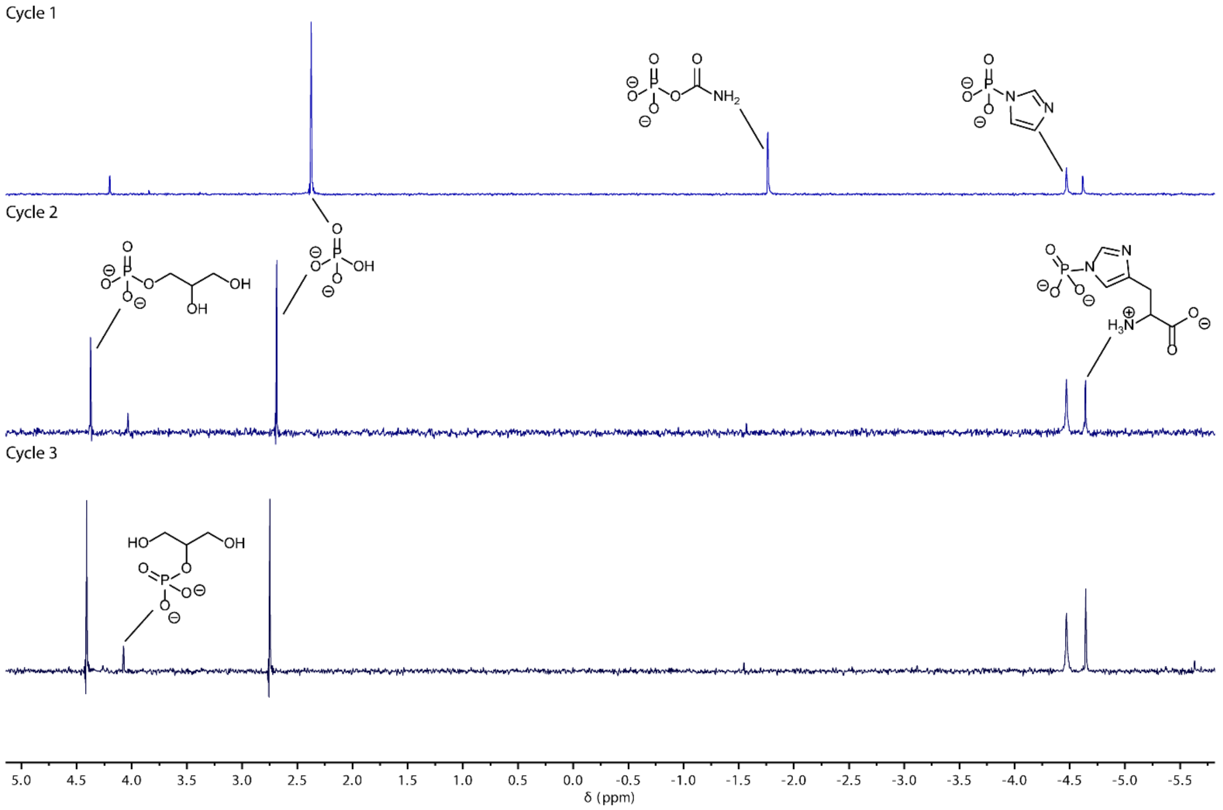

Supporting Figure 161: Representative ^{31}P-NMR spectra at the end of each cycle for the reaction of 20 mM sodium phosphate dibasic + 500 mM glycerol + 230 mM potassium cyanate + 100 mM imidazole + 100 mM histidine at pH 7.3 and 22 °C.

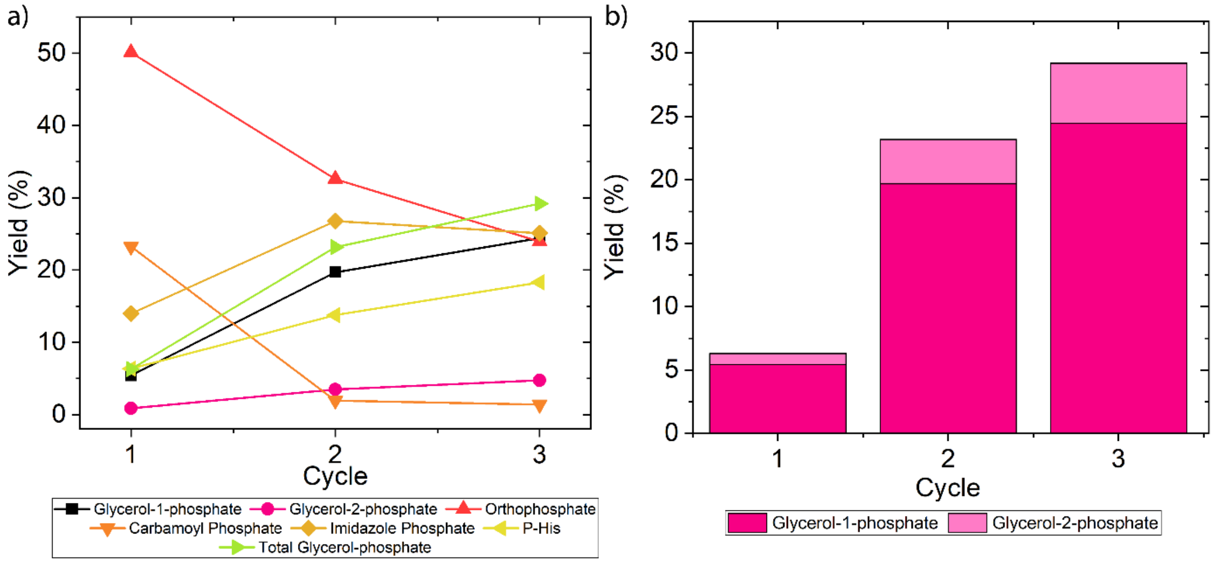

Supporting Figure 162: a) Changes in yield for all phosphate-containing products over three cycles for the reaction of for the reaction of 20 mM sodium phosphate dibasic + 500 mM glycerol + 230 mM potassium cyanate + 100 mM imidazole + 100 mM histidine at pH 7.3 and 22 °C. b) Change in yield for glycerol-1-phosphate and glycerol-2-phosphate over the course of three cycles.

S4.2.2 Wet/Dry cycle for the phosphorylation of glycerol catalysed by 100 mM histidine catalyst 1st repeat

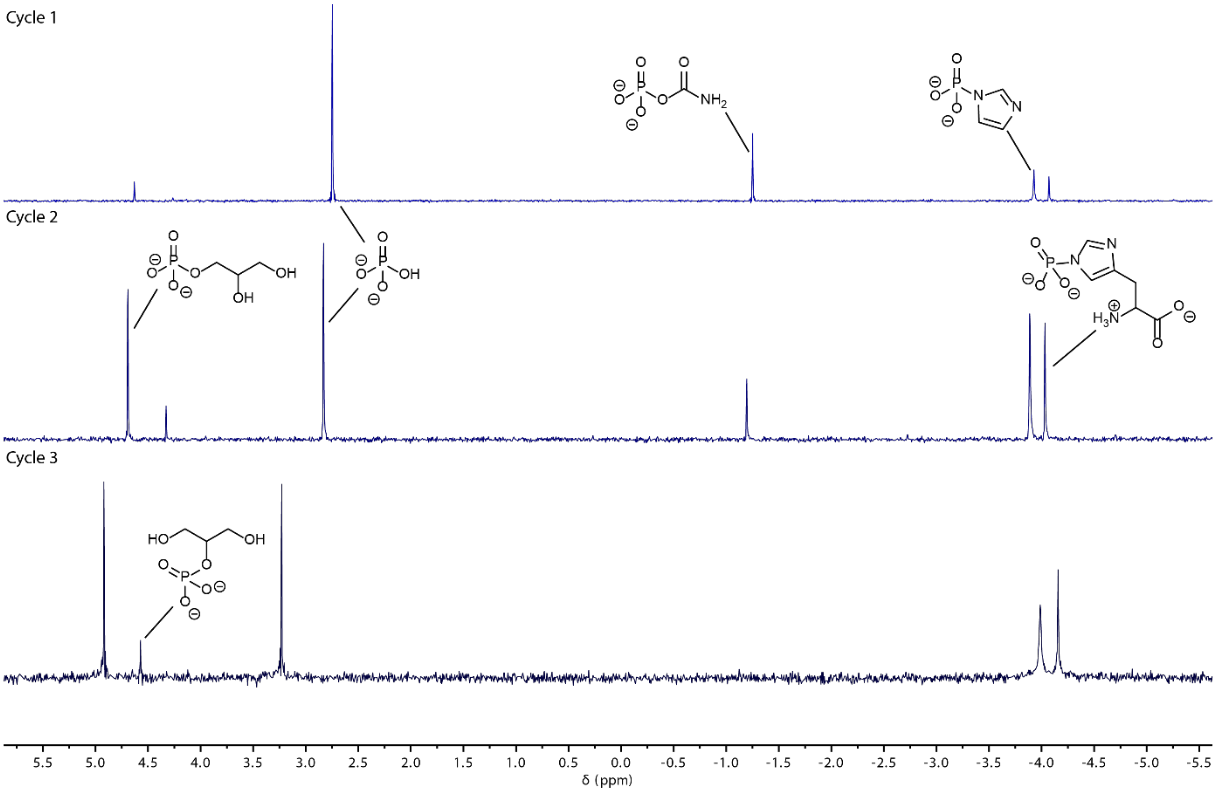

Supporting Figure 163: Representative ^{31}P-NMR spectra at the end of each cycle for the reaction of 20 mM sodium phosphate dibasic + 500 mM glycerol + 230 mM potassium cyanate + 100 mM imidazole + 100 mM histidine at pH 7.3 and 22 °C.

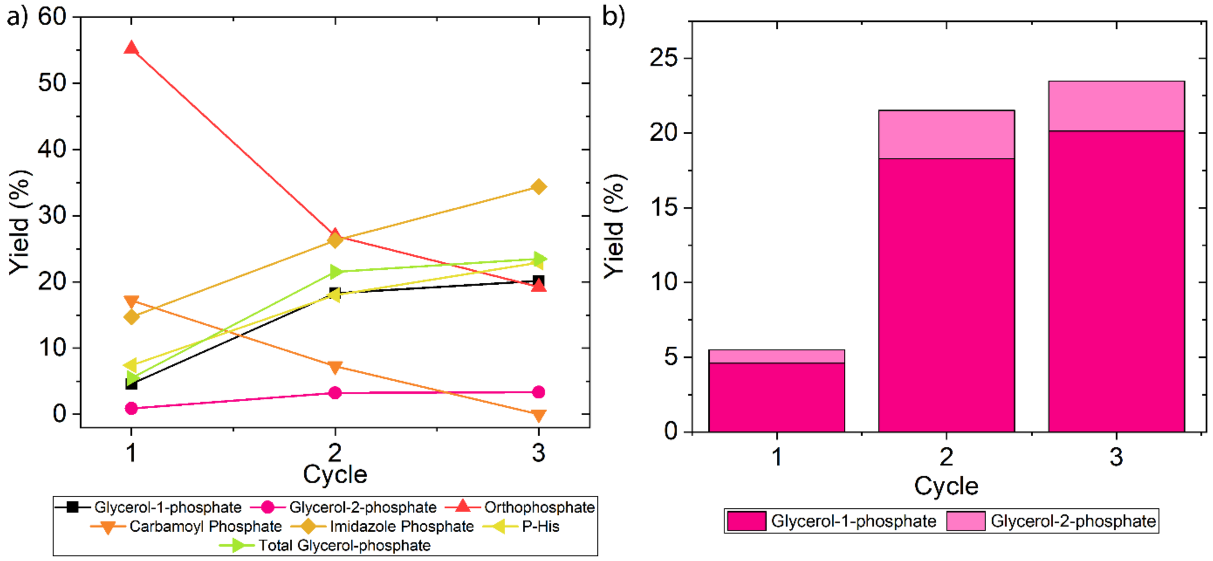

Supporting Figure 164: a) Changes in yield for all phosphate-containing products over three cycles for the reaction of 20 mM sodium phosphate dibasic + 500 mM glycerol + 230 mM potassium cyanate + 100 mM imidazole + 100 mM histidine at pH 7.3 and 22 °C. b) Change in yield for glycerol-1-phosphate and glycerol-2-phosphate over the course of three cycles.

S4.2.3 Wet/Dry cycle for the phosphorylation of glycerol catalysed by 100 mM histidine catalyst 2nd repeat

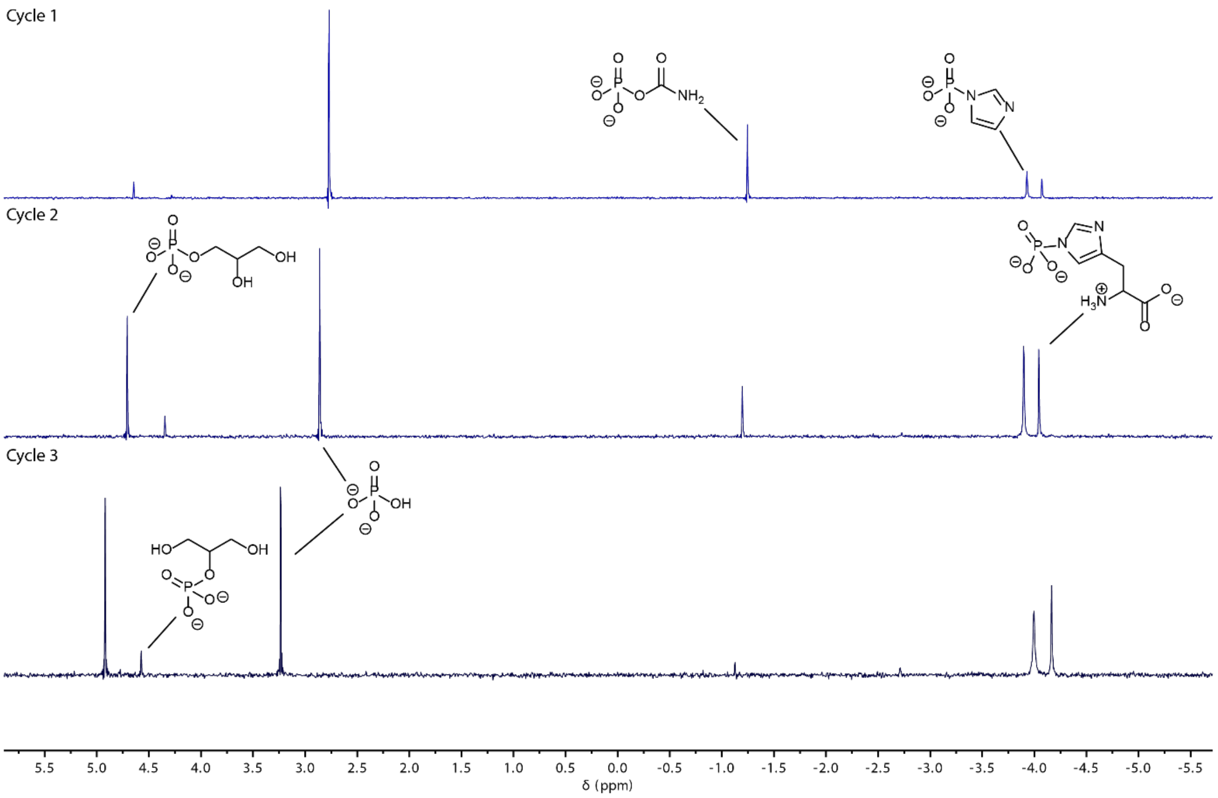

Supporting Figure 165: Representative ^{31}P-NMR spectra at the end of each cycle for the reaction of 20 mM sodium phosphate dibasic + 500 mM glycerol + 230 mM potassium cyanate + 100 mM imidazole + 100 mM histidine at pH 7.3 and 22 °C.

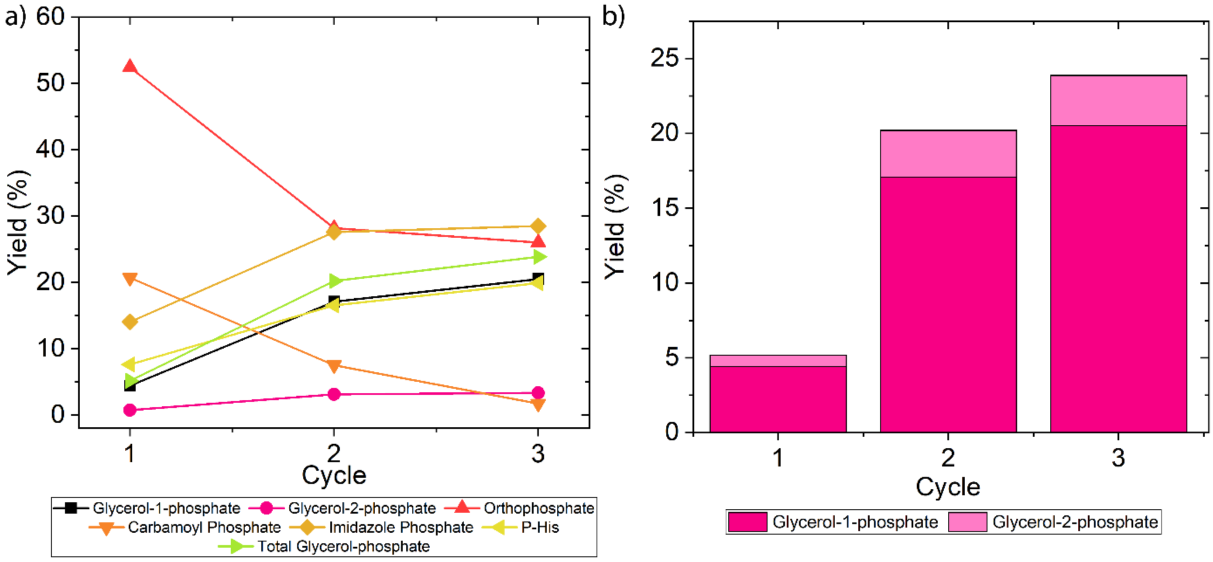

Supporting Figure 166: a) Changes in yield for all phosphate-containing products over three cycles for the reaction of 20 mM sodium phosphate dibasic + 500 mM glycerol + 230 mM potassium cyanate + 100 mM imidazole + 100 mM histidine at pH 7.3 and 22 °C. b) Change in yield for glycerol-1-phosphate and glycerol-2-phosphate over the course of three cycles.

S4.3 Wet/Dry Cycle for the phosphorylation of glycerol by imidazole phosphate with 50 mM histidine catalyst

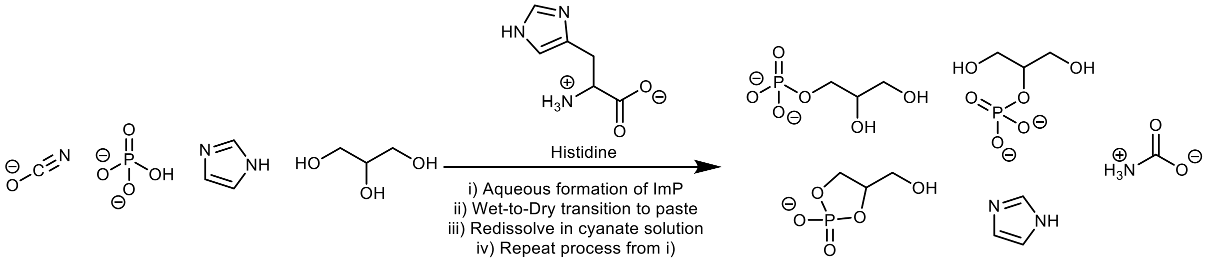

The experiment was carried out according to the procedure in S4.1 but with the 100 mM of histidine replaced by 50 mM histidine (31.0 mg, 0.2 mmol). The experiment was repeated in triplicate. Supporting Figures 167, 169, 171 and 173 depict representative ^{31}P NMR spectra after each wet-dry cycle. The changes in yield after each wet-dry cycle for all phosphate containing species are shown in Supporting Figure 168a, 170a, 172a and 174a. The change in yield of glycerol-1-phosphate and glycerol-2-phosphate after each wet-dry cycle are shown in Supporting Figure 168b, 170b, 172b and 174b.

S4.3.1 Wet/Dry cycle for the phosphorylation of glycerol catalysed by 50 mM histidine catalyst

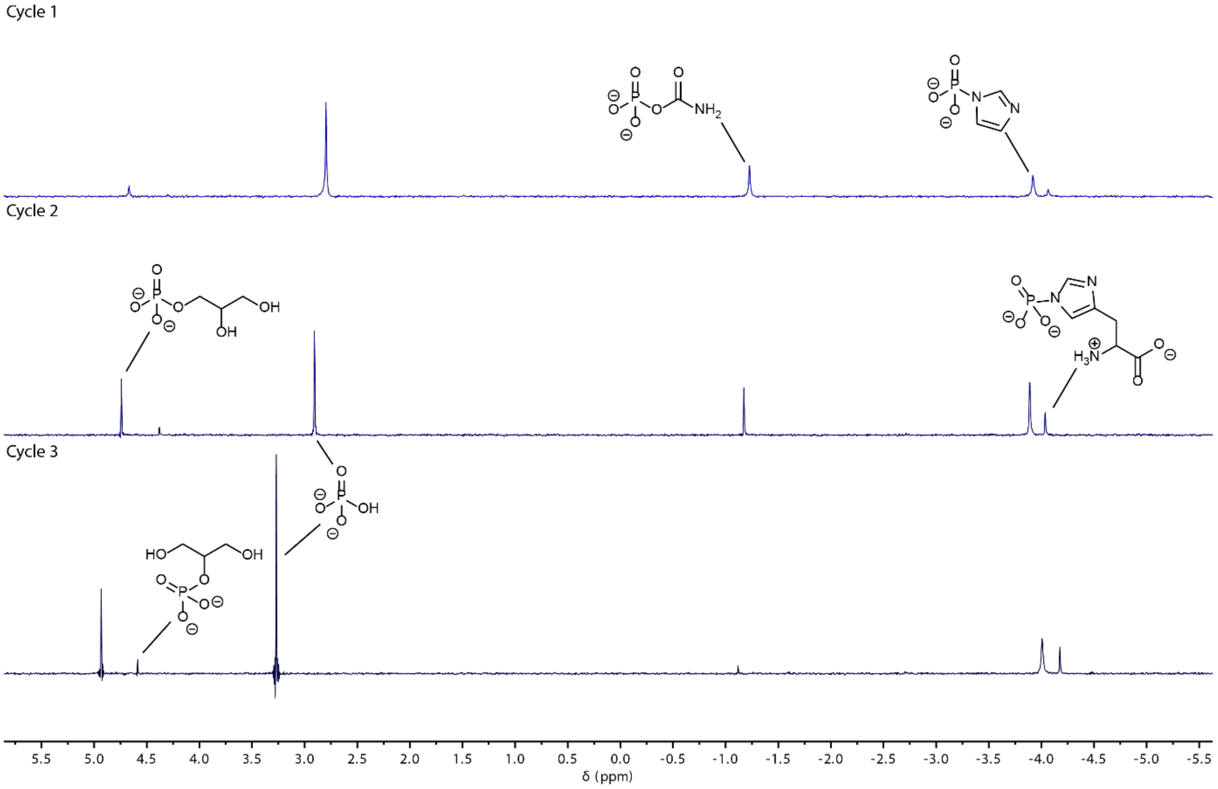

Supporting Figure 167: Representative ^{31}P-NMR spectra at the end of each cycle for the reaction of 20 mM sodium phosphate dibasic + 500 mM glycerol + 230 mM potassium cyanate + 100 mM imidazole + 50 mM histidine at pH 7.3 and 22 °C.

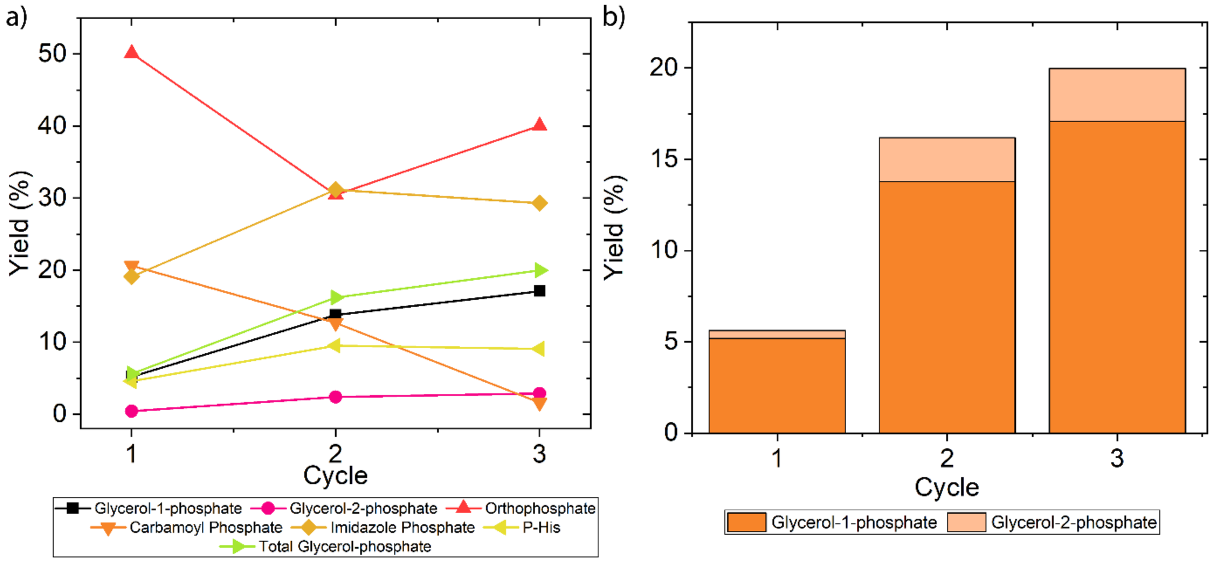

Supporting Figure 168: a) Changes in yield for all phosphate-containing products over three cycles for the reaction of 20 mM sodium phosphate dibasic + 500 mM glycerol + 230 mM potassium cyanate + 100 mM imidazole + 50 mM histidine at pH 7.3 and 22 °C. b) Change in yield for glycerol-1-phosphate and glycerol-2-phosphate over the course of three cycles.

S4.3.2 Wet/Dry cycle for the phosphorylation of glycerol catalysed by 50 mM histidine catalyst 1st repeat

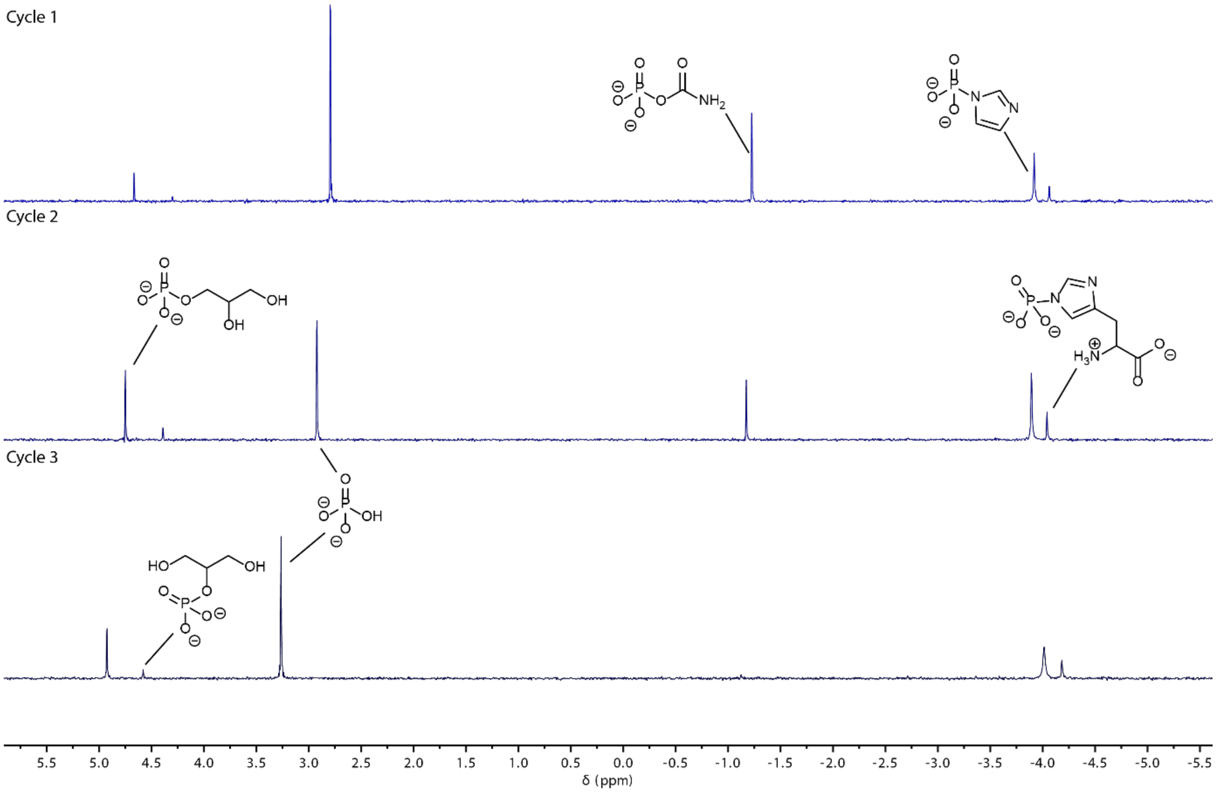

Supporting Figure 169: Representative ^{31}P-NMR spectra at the end of each cycle for the reaction of 20 mM sodium phosphate dibasic + 500 mM glycerol + 230 mM potassium cyanate + 100 mM imidazole + 50 mM histidine at pH 7.3 and 22 °C.

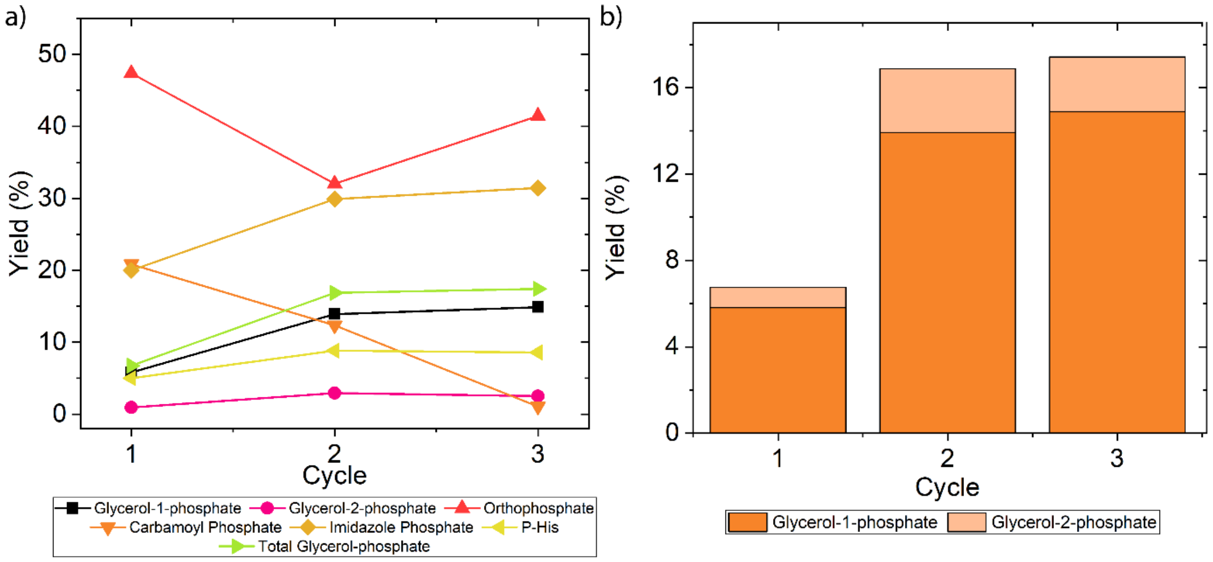

Supporting Figure 170: a) Changes in yield for all phosphate-containing products over three cycles for the reaction of 20 mM sodium phosphate dibasic + 500 mM glycerol + 230 mM potassium cyanate + 100 mM imidazole + 50 mM histidine at pH 7.3 and 22 °C. b) Change in yield for glycerol-1-phosphate and glycerol-2-phosphate over the course of three cycles.

S4.3.3 Wet/Dry cycle for the phosphorylation of glycerol catalysed by 50 mM histidine catalyst 2nd repeat

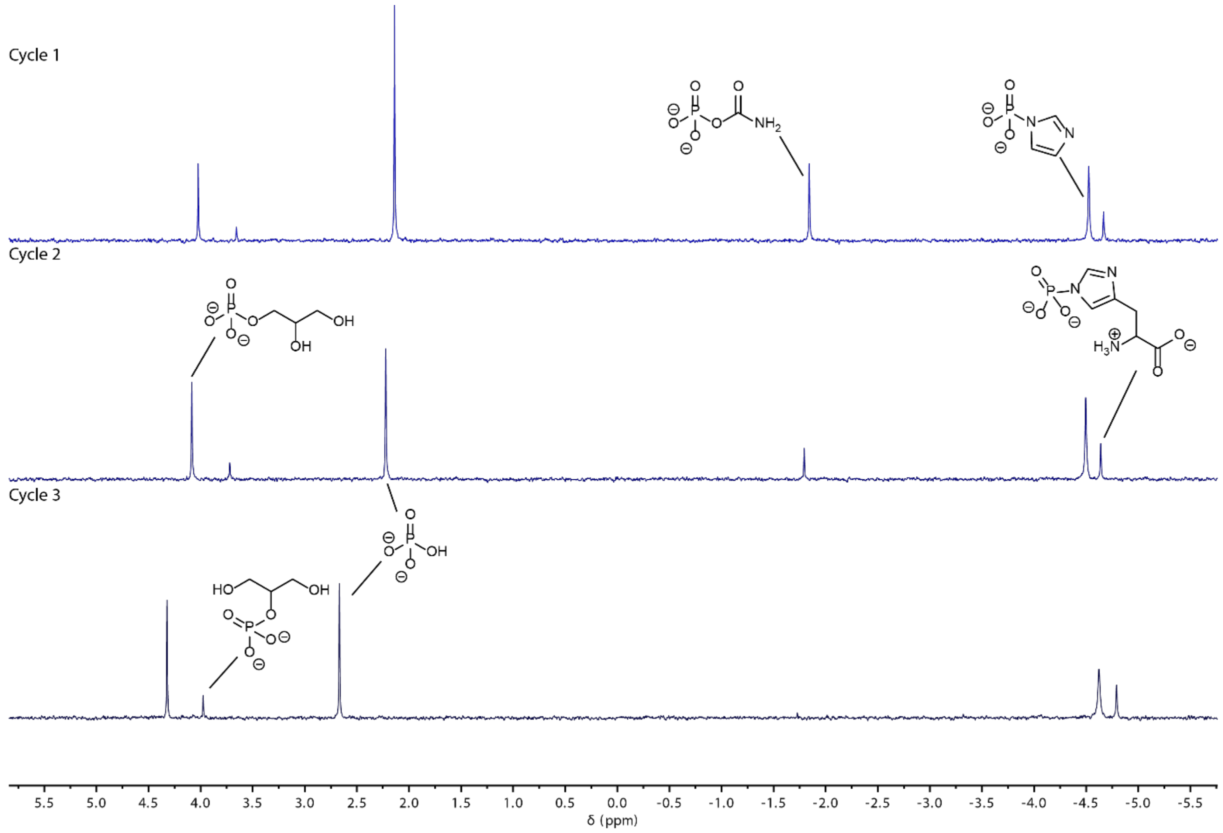

Supporting Figure 171: Representative ^{31}P-NMR spectra at the end of each cycle for the reaction of 20 mM sodium phosphate dibasic + 500 mM glycerol + 230 mM potassium cyanate + 100 mM imidazole + 50 mM histidine at pH 7.3 and 22 °C.

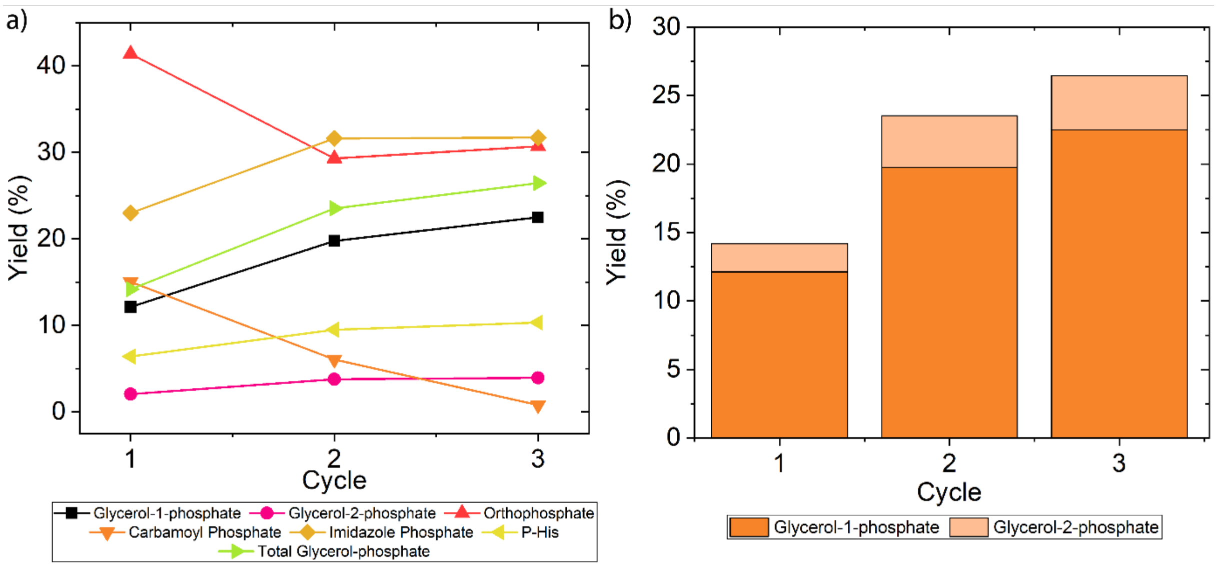

Supporting Figure 172: a) Changes in yield for all phosphate-containing products over three cycles for the reaction of 20 mM sodium phosphate dibasic + 500 mM glycerol + 230 mM potassium cyanate + 100 mM imidazole + 50 mM histidine at pH 7.3 and 22 °C. b) Change in yield for glycerol-1-phosphate and glycerol-2-phosphate over the course of three cycles.

S4.3.4 Wet/Dry cycle for the phosphorylation of glycerol catalysed by 50 mM histidine catalyst 3rd repeat

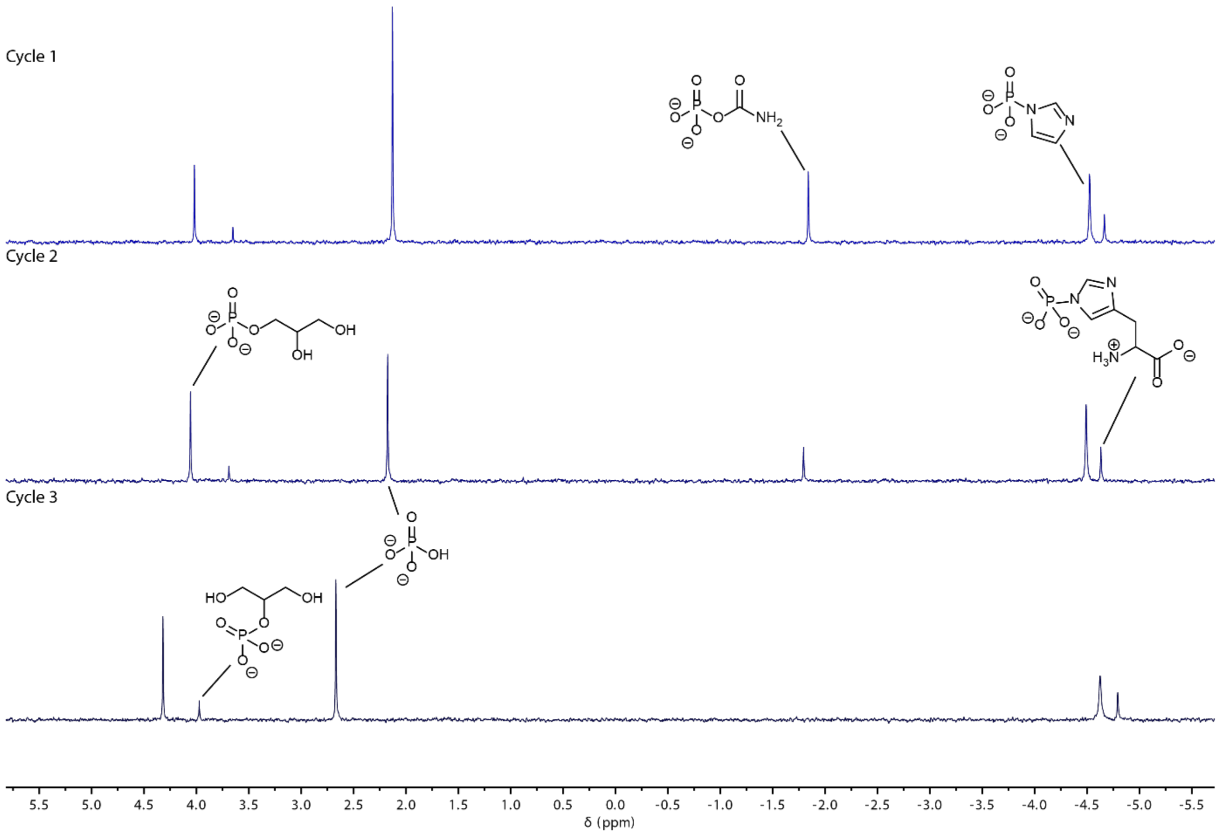

Supporting Figure 173: Representative ^{31}P-NMR spectra at the end of each cycle for the reaction of 20 mM sodium phosphate dibasic + 500 mM glycerol + 230 mM potassium cyanate + 100 mM imidazole + 50 mM histidine at pH 7.3 and 22 °C.

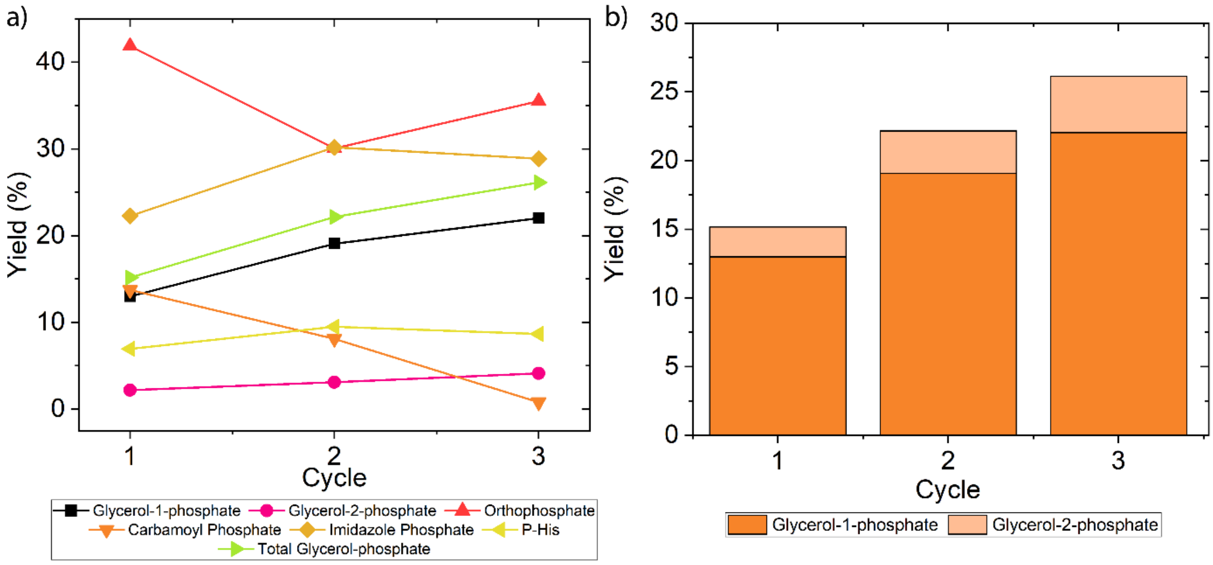

Supporting Figure 174: a) Changes in yield for all phosphate-containing products over three cycles for the reaction of 20 mM sodium phosphate dibasic + 500 mM glycerol + 230 mM potassium cyanate + 100 mM imidazole + 50 mM histidine at pH 7.3 and 22 °C. b) Change in yield for glycerol-1-phosphate and glycerol-2-phosphate over the course of three cycles.

S4.4 Wet/Dry Cycle for the phosphorylation of glycerol by imidazole phosphate with 100 mM His-NH_2 catalyst

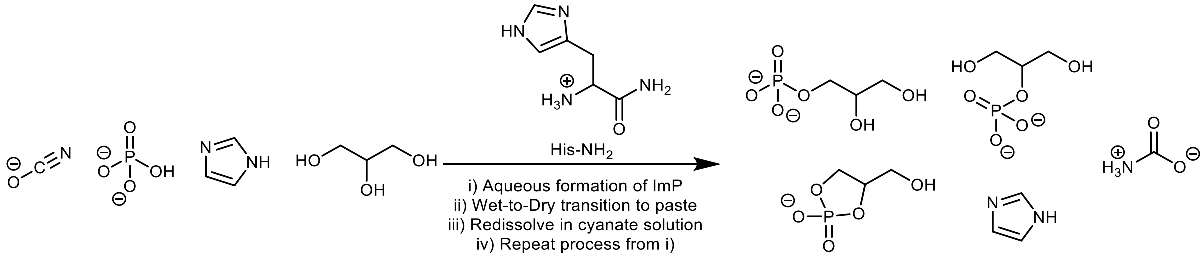

The experiment was carried out according to the procedure in S4.1 but with the 100 mM of histidine replaced by 100 mM His-NH_2.2HCl (90.8 mg, 0.4 mmol). The experiment was repeated in triplicate. Supporting Figures 175, 177 and 179 depict representative ^{31}P NMR spectra after each wet-dry cycle. The changes in yield after each wet-dry cycle for all phosphate containing species are shown in Supporting Figure 176a, 178a and 180a. The change in yield of glycerol-1-phosphate and glycerol-2-phosphate after each wet-dry cycle are shown in Supporting Figure 176b, 178b and 180b.

S4.4.1 Wet/Dry cycle for the phosphorylation of glycerol catalysed by 100 mM His-NH_2 catalyst

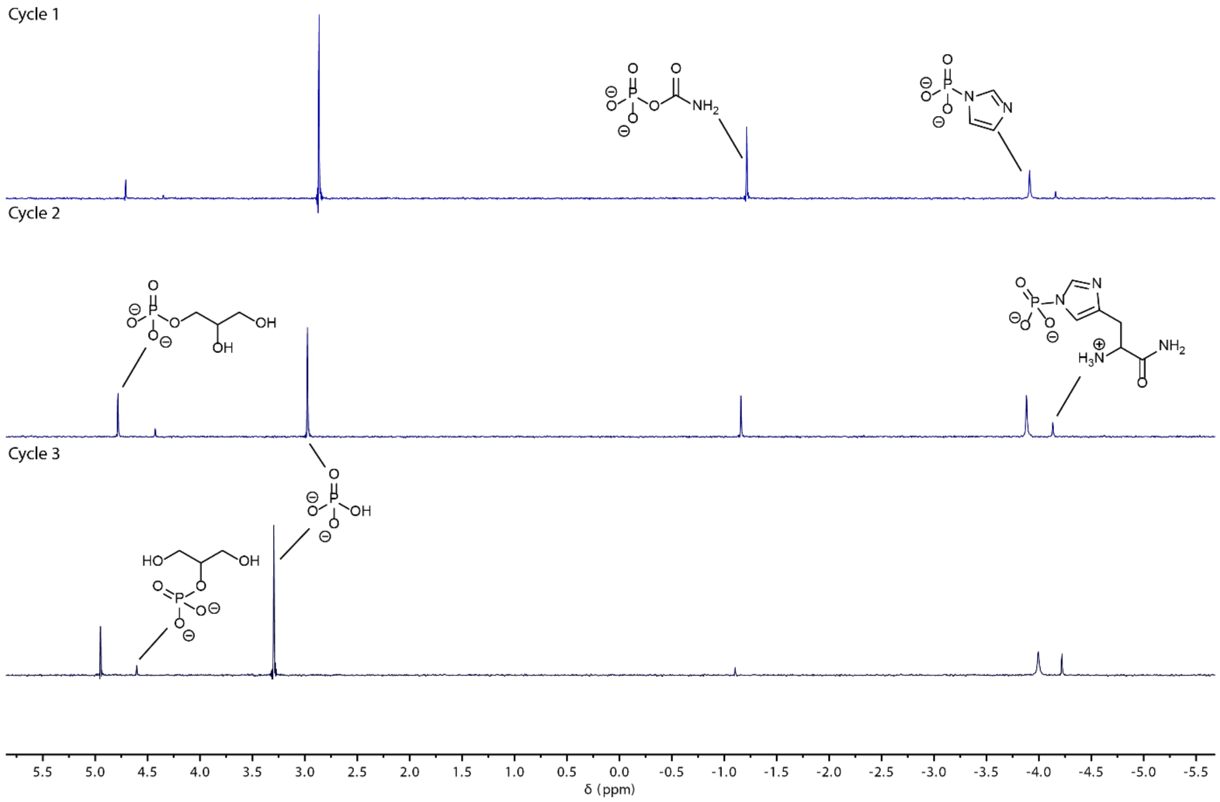

Supporting Figure 175: Representative ^{31}P-NMR spectra at the end of each cycle for the reaction of 20 mM sodium phosphate dibasic + 500 mM glycerol + 230 mM potassium cyanate + 100 mM imidazole + 100 mM His-NH_2 at pH 7.3 and 22 °C.

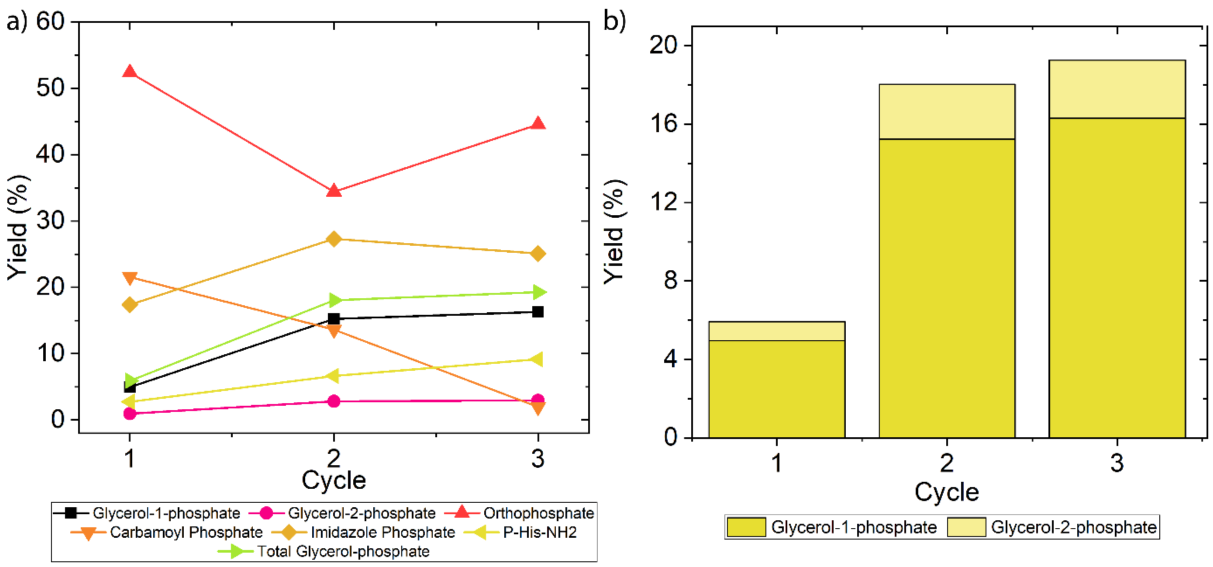

Supporting Figure 176: a) Changes in yield for all phosphate-containing products over three cycles for the reaction of 20 mM sodium phosphate dibasic + 500 mM glycerol + 230 mM potassium cyanate + 100 mM imidazole + 100 mM His-NH_2 at pH 7.3 and 22 °C. b) Change in yield for glycerol-1-phosphate and glycerol-2-phosphate over the course of three cycles.

S4.4.2 Wet/Dry cycle for the phosphorylation of glycerol catalysed by 100 mM His-NH_2 catalyst 1st repeat

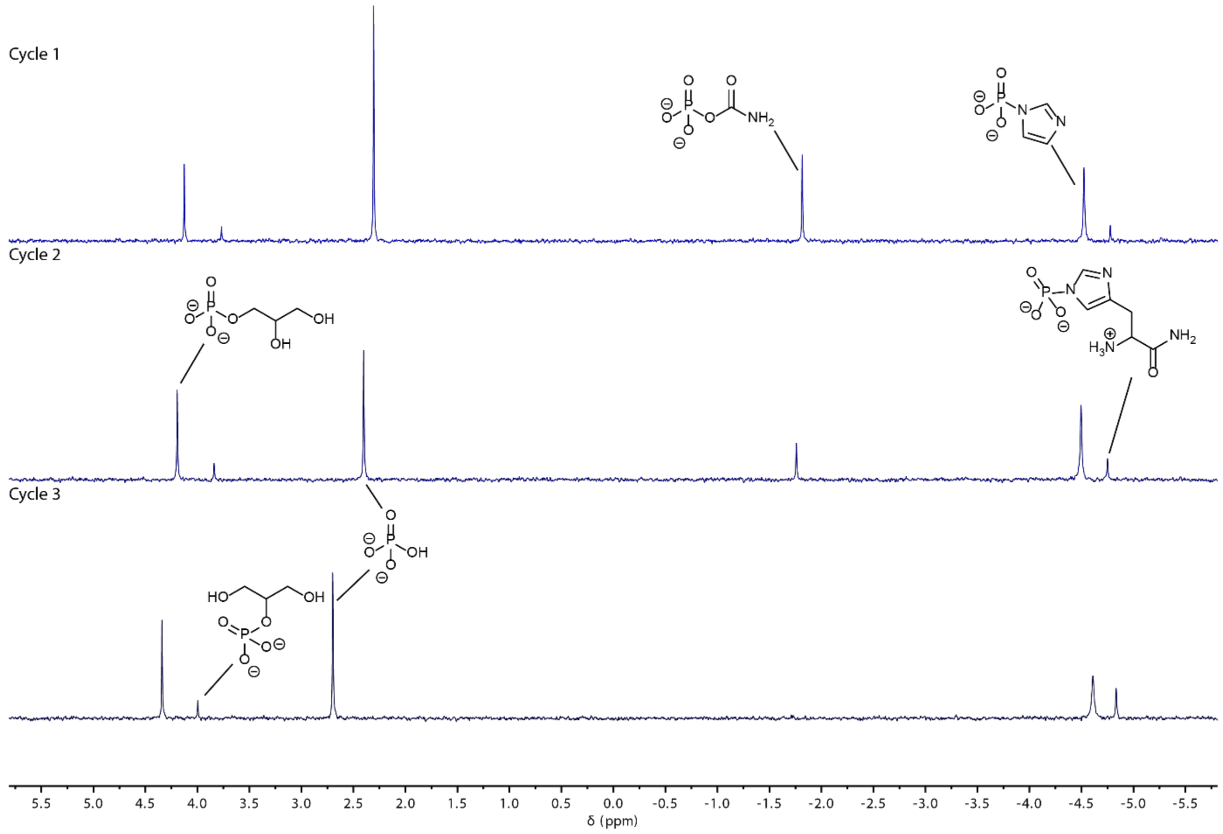

Supporting Figure 177: Representative ^{31}P-NMR spectra at the end of each cycle for the reaction of 20 mM sodium phosphate dibasic + 500 mM glycerol + 230 mM potassium cyanate + 100 mM imidazole + 100 mM His-NH_2 at pH 7.3 and 22 °C.

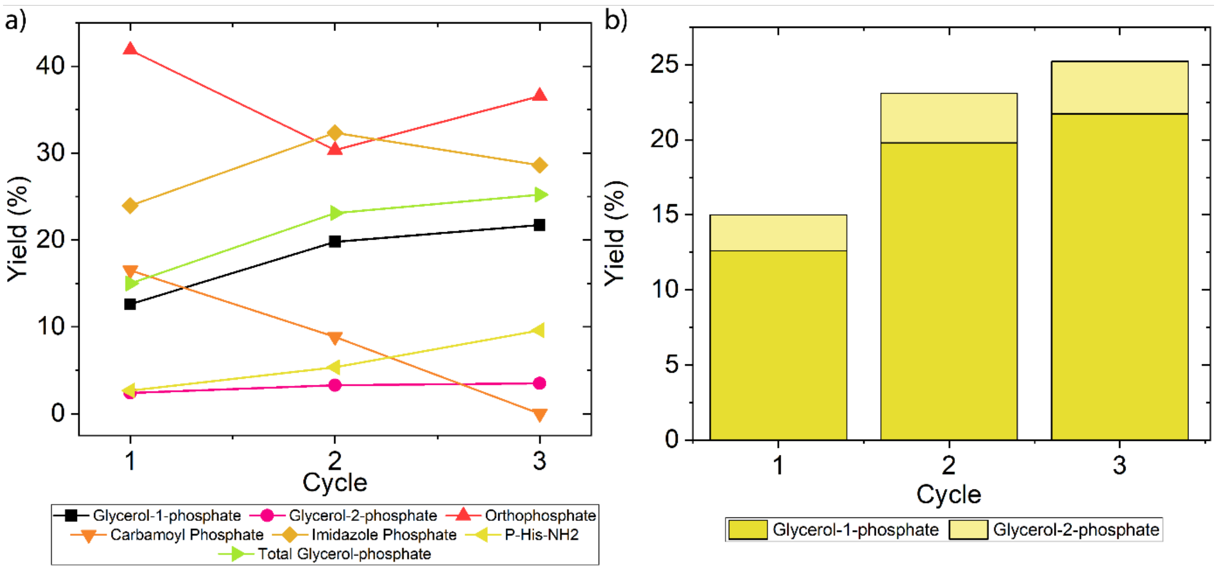

Supporting Figure 178: a) Changes in yield for all phosphate-containing products over three cycles for the reaction of 20 mM sodium phosphate dibasic + 500 mM glycerol + 230 mM potassium cyanate + 100 mM imidazole + 100 mM His-NH_2 at pH 7.3 and 22 °C. b) Change in yield for glycerol-1-phosphate and glycerol-2-phosphate over the course of three cycles.

S4.4.3 Wet/Dry cycle for the phosphorylation of glycerol catalysed by 100 mM His-NH₂ catalyst 2nd repeat

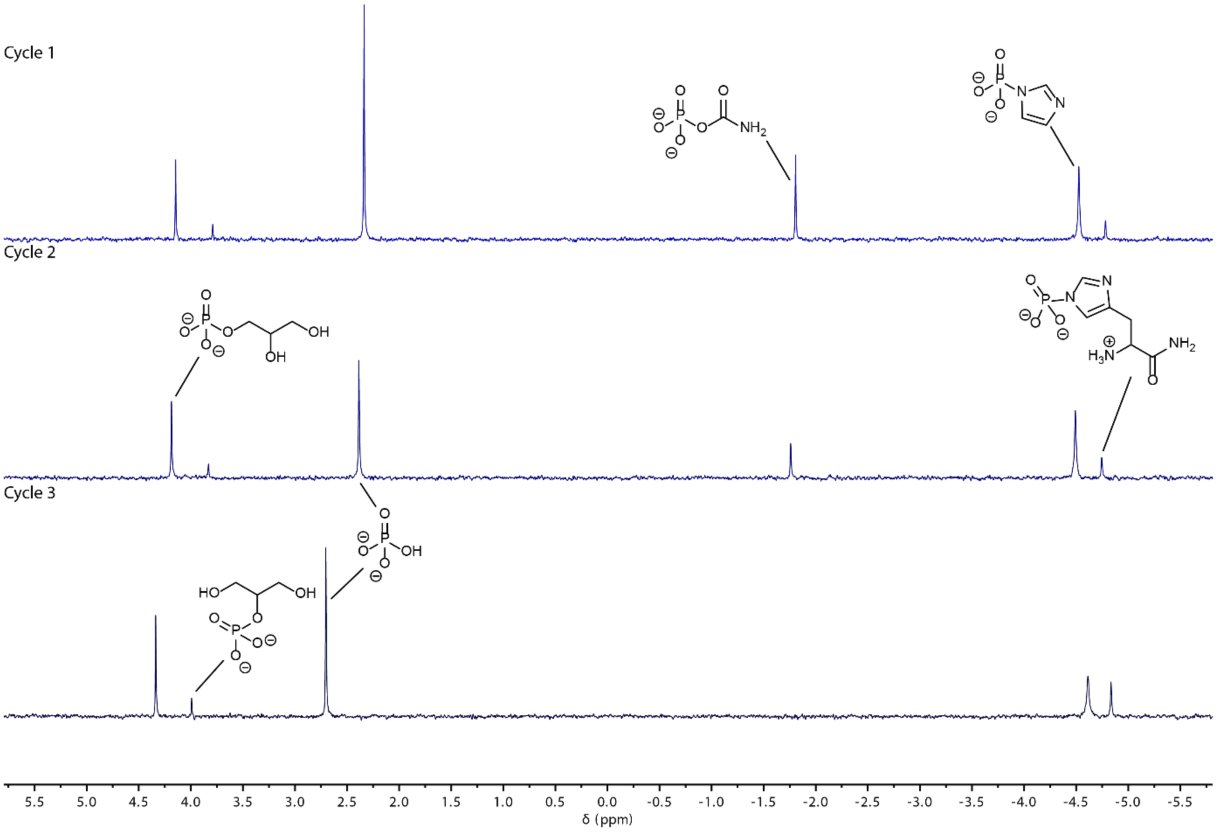

Supporting Figure 179: Representative ^{31}P-NMR spectra at the end of each cycle for the reaction of 20 mM sodium phosphate dibasic + 500 mM glycerol + 230 mM potassium cyanate + 100 mM imidazole + 100 mM His-NH_2 at pH 7.3 and 22 °C.

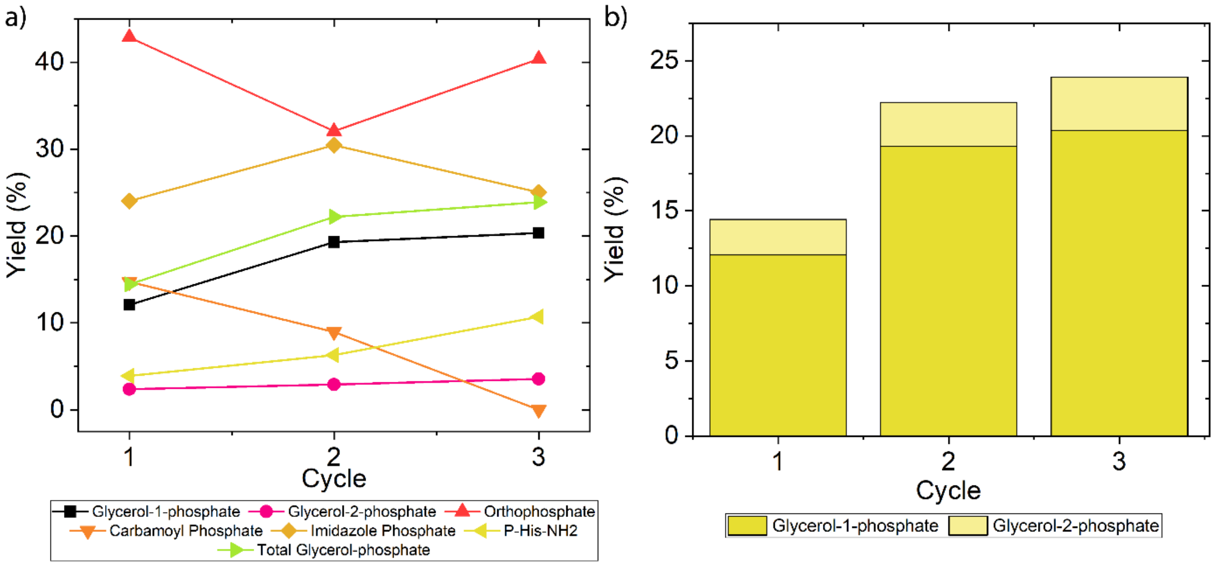

Supporting Figure 180: a) Changes in yield for all phosphate-containing products over three cycles for the reaction of 20 mM sodium phosphate dibasic + 500 mM glycerol + 230 mM potassium cyanate + 100 mM imidazole + 100 mM His-NH_2 at pH 7.3 and 22 °C. b) Change in yield for glycerol-1-phosphate and glycerol-2-phosphate over the course of three cycles.

S4.5 Wet/Dry Cycle for the phosphorylation of glycerol by imidazole phosphate with 50 mM His-NH$_2$ catalyst

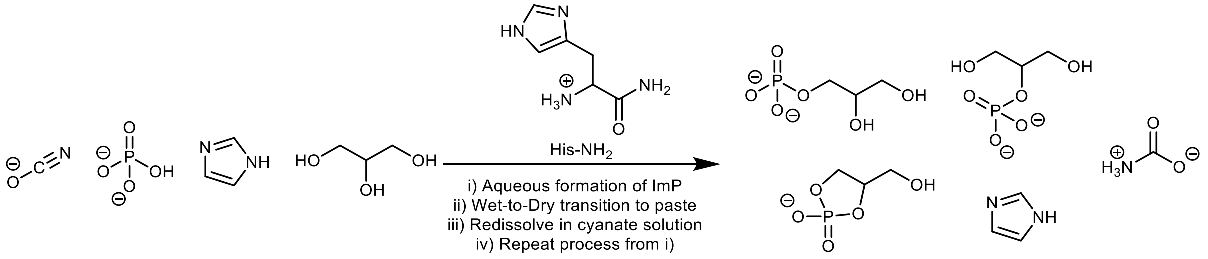

The experiment was carried out according to the procedure in S4.1 but with the 100 mM of histidine replaced by 50 mM His-NH$_2$.2HCl (45.4 mg, 0.2 mmol). The experiment was repeated in triplicate. Supporting Figures 181, 183 and 185 depict representative ^{31}P NMR spectra after each wet-dry cycle. The changes in yield after each wet-dry cycle for all phosphate containing species are shown in Supporting Figure 182a, 184a and 186a. The change in yield of glycerol-1-phosphate and glycerol-2-phosphate after each wet-dry cycle are shown in Supporting Figure 182b, 184b and 186b.

S4.5.1 Wet/Dry cycle for the phosphorylation of glycerol catalysed by 50 mM His-NH$_2$ catalyst

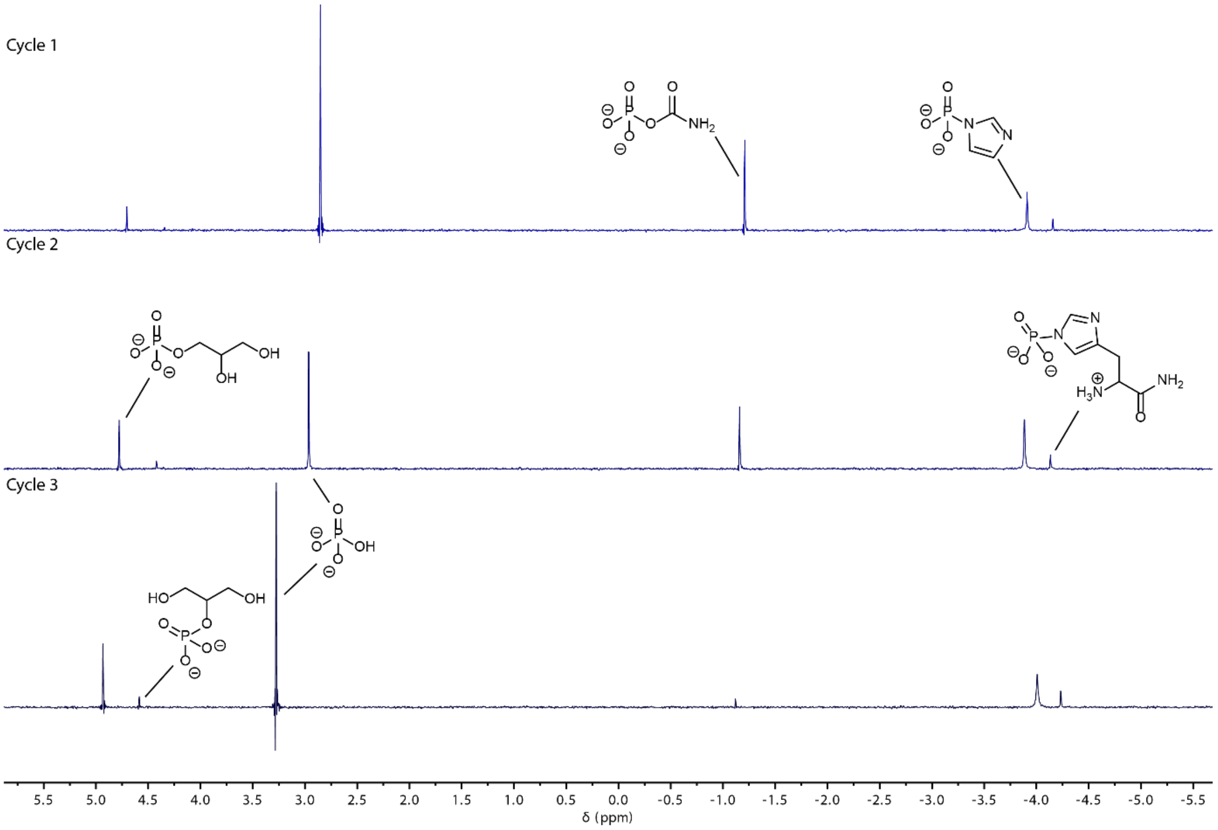

Supporting Figure 181: Representative ^{31}P-NMR spectra at the end of each cycle for the reaction of 20 mM sodium phosphate dibasic + 500 mM glycerol + 230 mM potassium cyanate + 100 mM imidazole + 50 mM His-NH$_2$ at pH 7.3 and 22 °C.

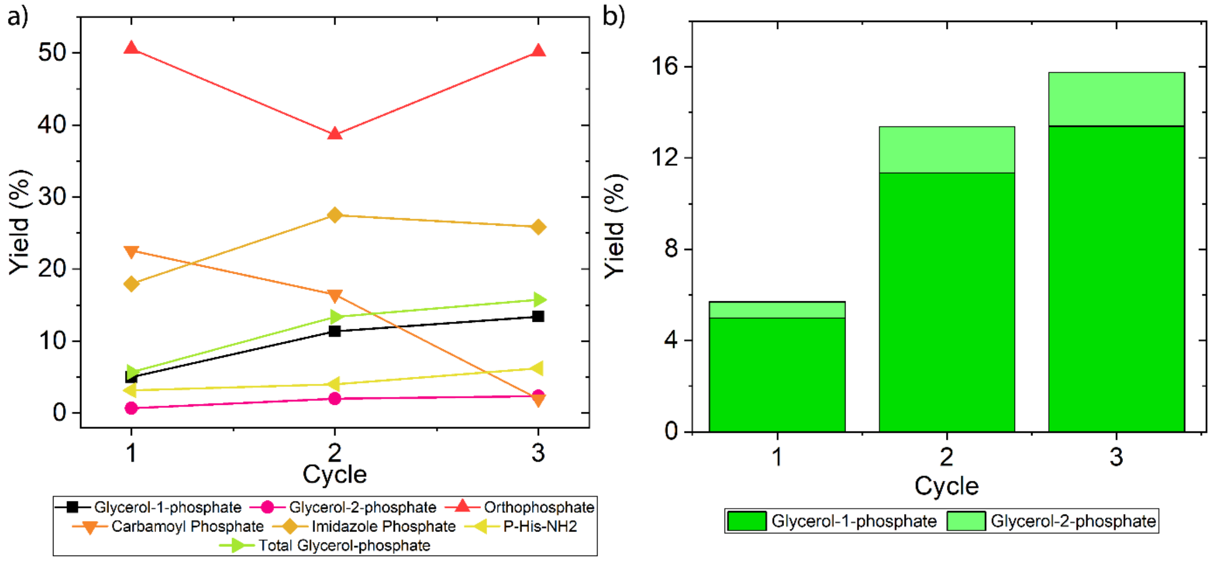

Supporting Figure 182: a) Changes in yield for all phosphate-containing products over three cycles for the reaction of 20 mM sodium phosphate dibasic + 500 mM glycerol + 230 mM potassium cyanate + 100 mM imidazole + 50 mM His-NH_2 at pH 7.3 and 22 °C. b) Change in yield for glycerol-1-phosphate and glycerol-2-phosphate over the course of three cycles.

S4.5.2 Wet/Dry cycle for the phosphorylation of glycerol catalysed by 50 mM His-NH_2 catalyst 1st repeat

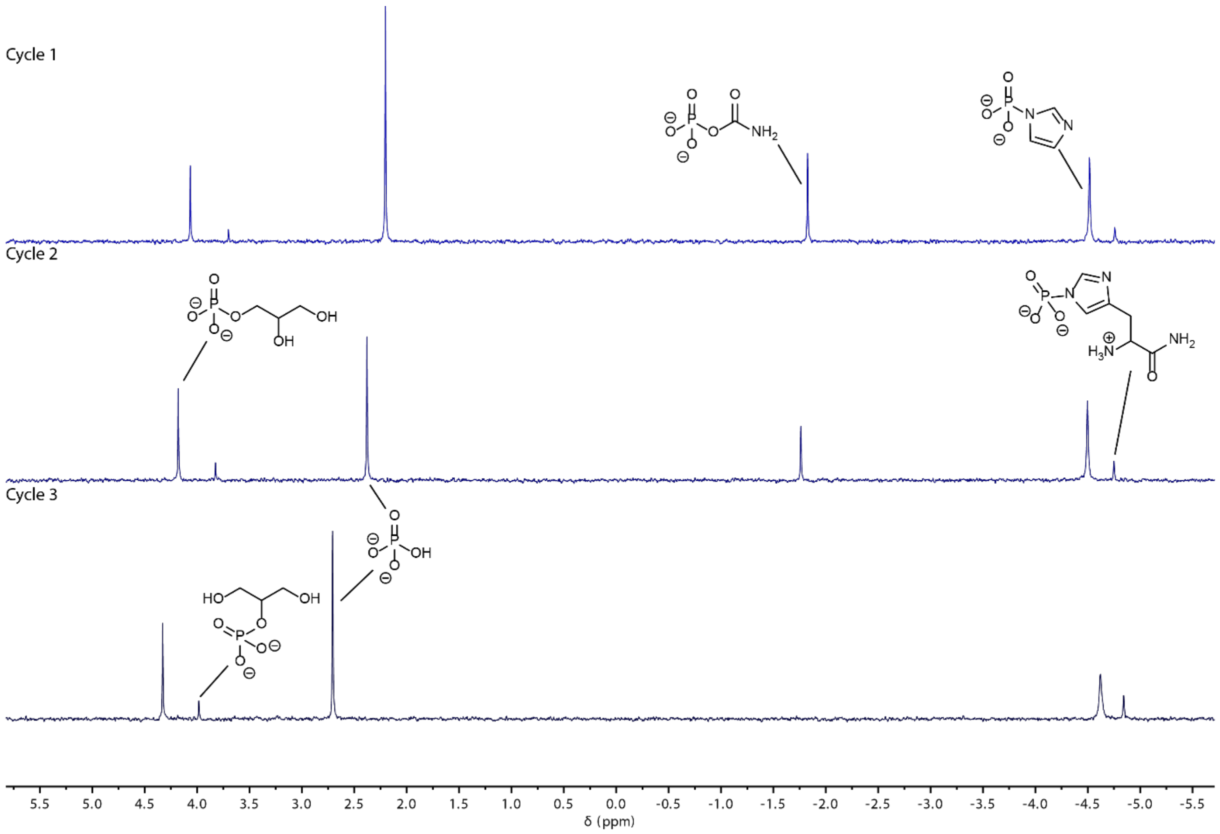

Supporting Figure 183: Representative ^{31}P-NMR spectra at the end of each cycle for the reaction of 20 mM sodium phosphate dibasic + 500 mM glycerol + 230 mM potassium cyanate + 100 mM imidazole + 50 mM His-NH_2 at pH 7.3 and 22 °C.

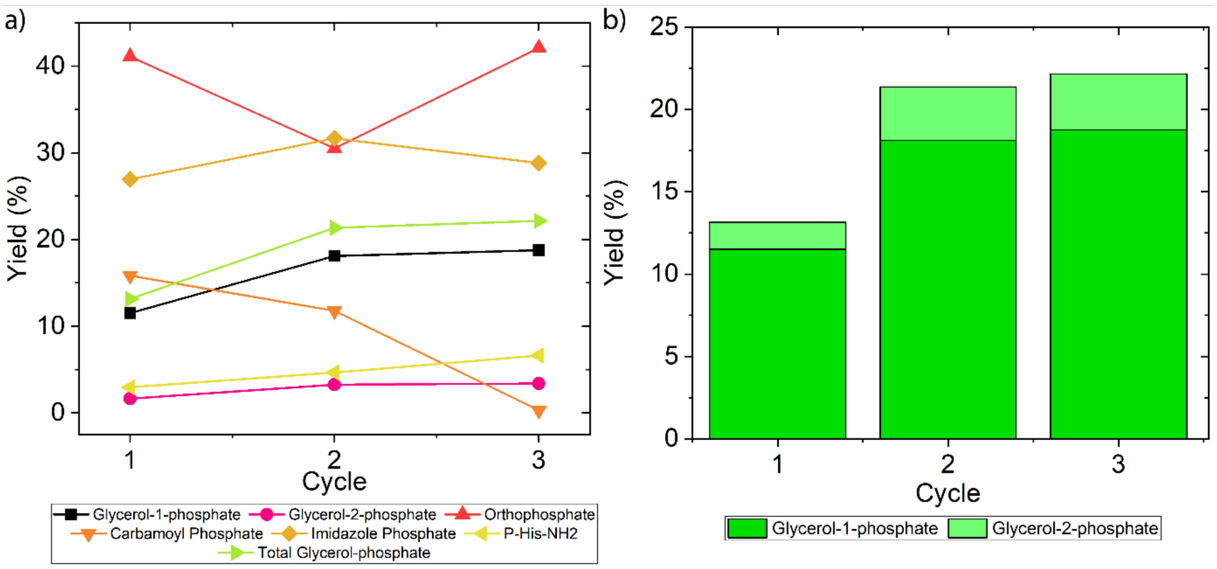

Supporting Figure 184: a) Changes in yield for all phosphate-containing products over three cycles for the reaction of 20 mM sodium phosphate dibasic + 500 mM glycerol + 230 mM potassium cyanate + 100 mM imidazole + 50 mM His-NH_2 at pH 7.3 and 22 °C. b) Change in yield for glycerol-1-phosphate and glycerol-2-phosphate over the course of three cycles.

S4.5.3 Wet/Dry cycle for the phosphorylation of glycerol catalysed by 50 mM His-NH_2 catalyst 2nd repeat

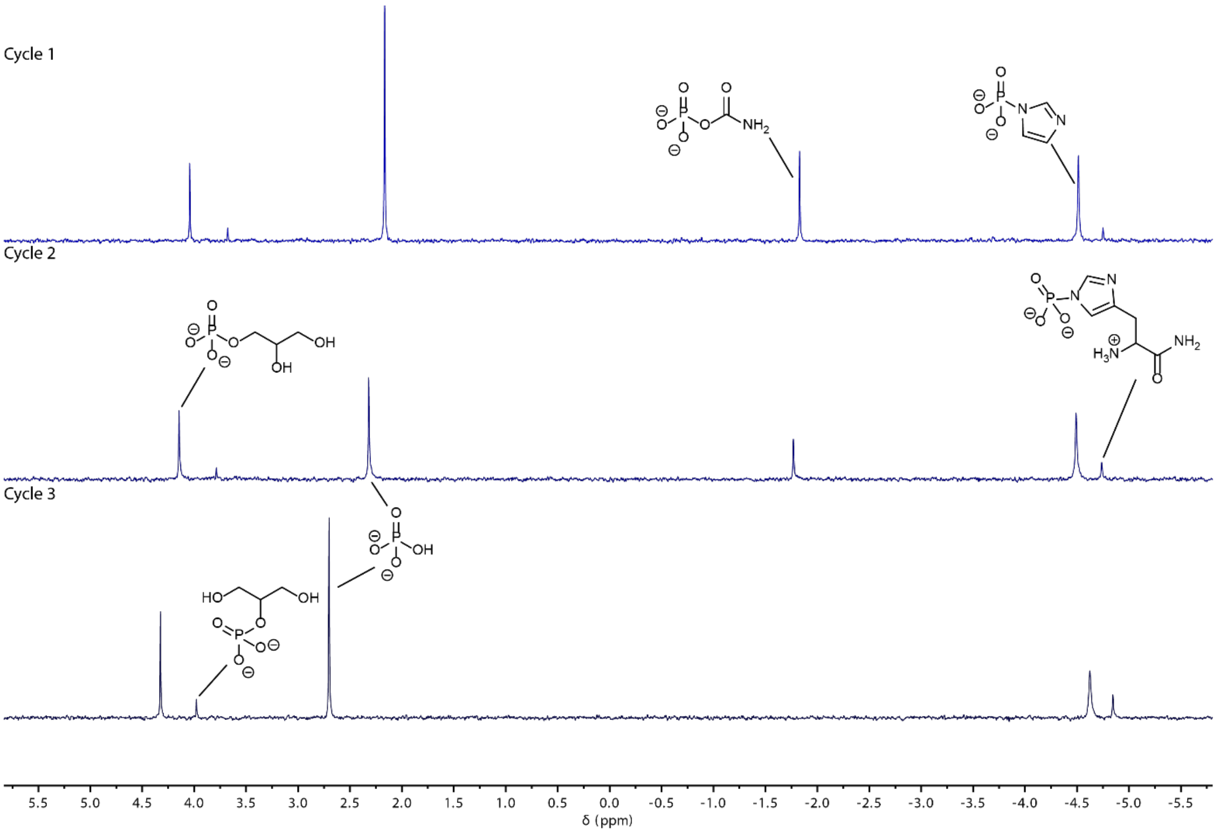

Supporting Figure 185: Representative ^{31}P-NMR spectra at the end of each cycle for the reaction of 20 mM sodium phosphate dibasic + 500 mM glycerol + 230 mM potassium cyanate + 100 mM imidazole + 50 mM His-NH_2 at pH 7.3 and 22 °C.

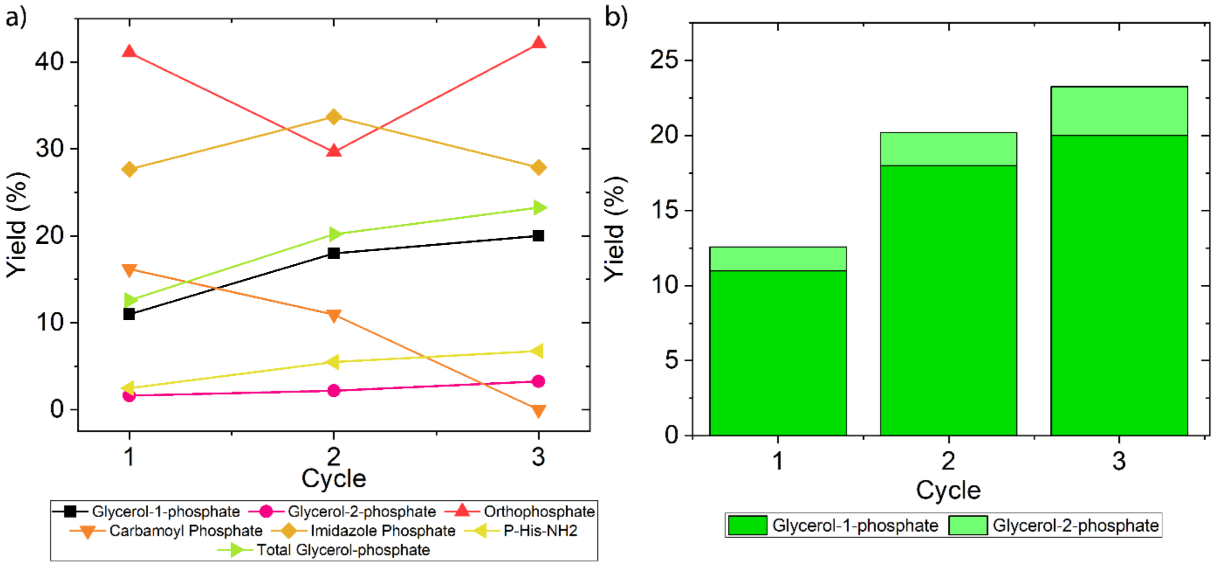

Supporting Figure 186: a) Changes in yield for all phosphate-containing products over three cycles for the reaction of 20 mM sodium phosphate dibasic + 500 mM glycerol + 230 mM potassium cyanate + 100 mM imidazole + 50 mM His-NH_2 at pH 7.3 and 22 °C. b) Change in yield for glycerol-1-phosphate and glycerol-2-phosphate over the course of three cycles.

S4.6 Wet/Dry Cycle for the phosphorylation of glycerol by imidazole phosphate with a His-Asp catalyst

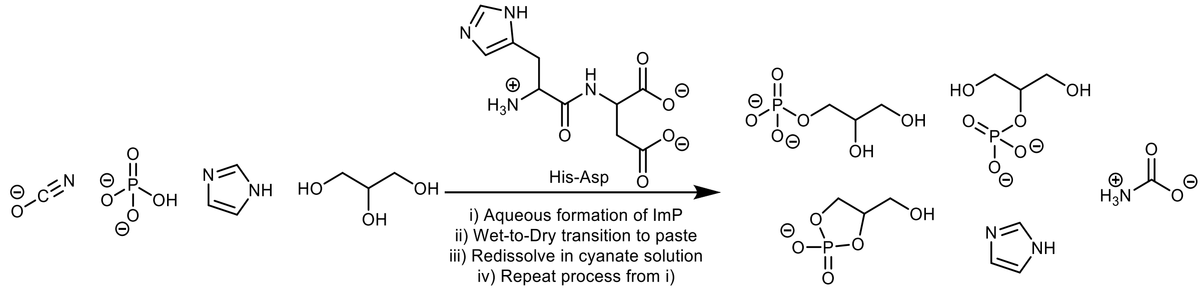

The experiment was carried out according to the procedure in S4.1 but with the 100 mM of histidine replaced by 100 mM His-Asp (108.1 mg, 0.4 mmol). The experiment was repeated in triplicate. Supporting Figures 187, 189 and 191 depict representative ^{31}P NMR spectra after each wet-dry cycle. The changes in yield after each wet-dry cycle for all phosphate containing species are shown in Supporting Figure 188a, 190a and 192a. The change in yield of glycerol-1-phosphate and glycerol-2-phosphate after each wet-dry cycle are shown in Supporting Figure 188b, 190b and 192b.

S4.6.1 Wet/Dry cycle for the phosphorylation of glycerol catalysed by 100 mM His-Asp catalyst

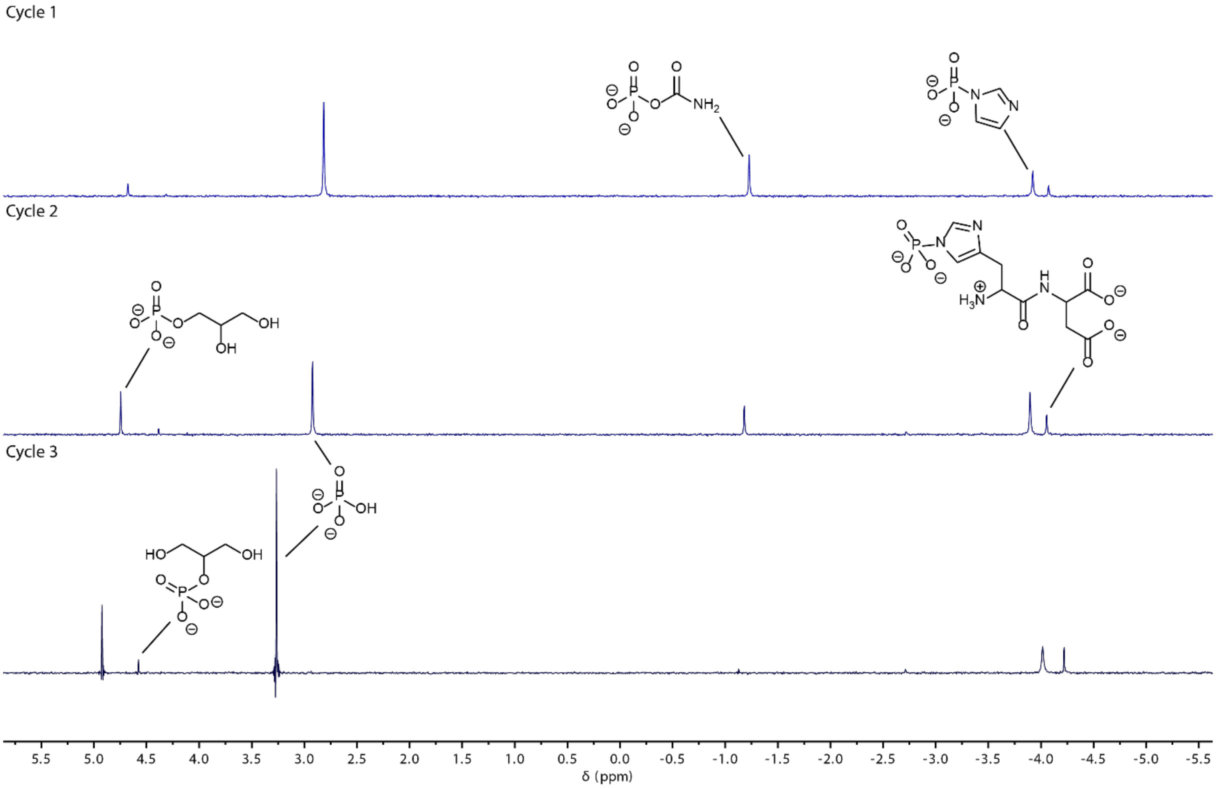

Supporting Figure 187: Representative ^{31}P-NMR spectra at the end of each cycle for the reaction of 20 mM sodium phosphate dibasic + 500 mM glycerol + 230 mM potassium cyanate + 100 mM imidazole + 100 mM His-Asp at pH 7.3 and 22 °C.

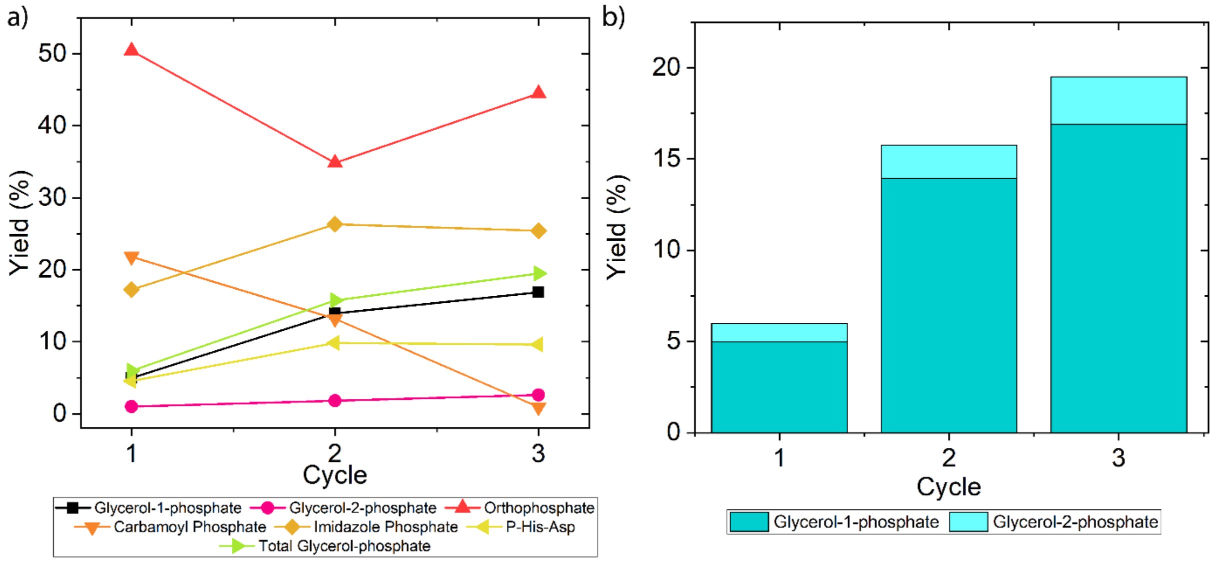

Supporting Figure 188: a) Changes in yield for all phosphate-containing products over three cycles for the reaction of 20 mM sodium phosphate dibasic + 500 mM glycerol + 230 mM potassium cyanate + 100 mM imidazole + 100 mM His-Asp at pH 7.3 and 22 °C. b) Change in yield for glycerol-1-phosphate and glycerol-2-phosphate over the course of three cycles.

S4.6.2 Wet/Dry cycle for the phosphorylation of glycerol catalysed by 100 mM His-Asp catalyst 1st repeat

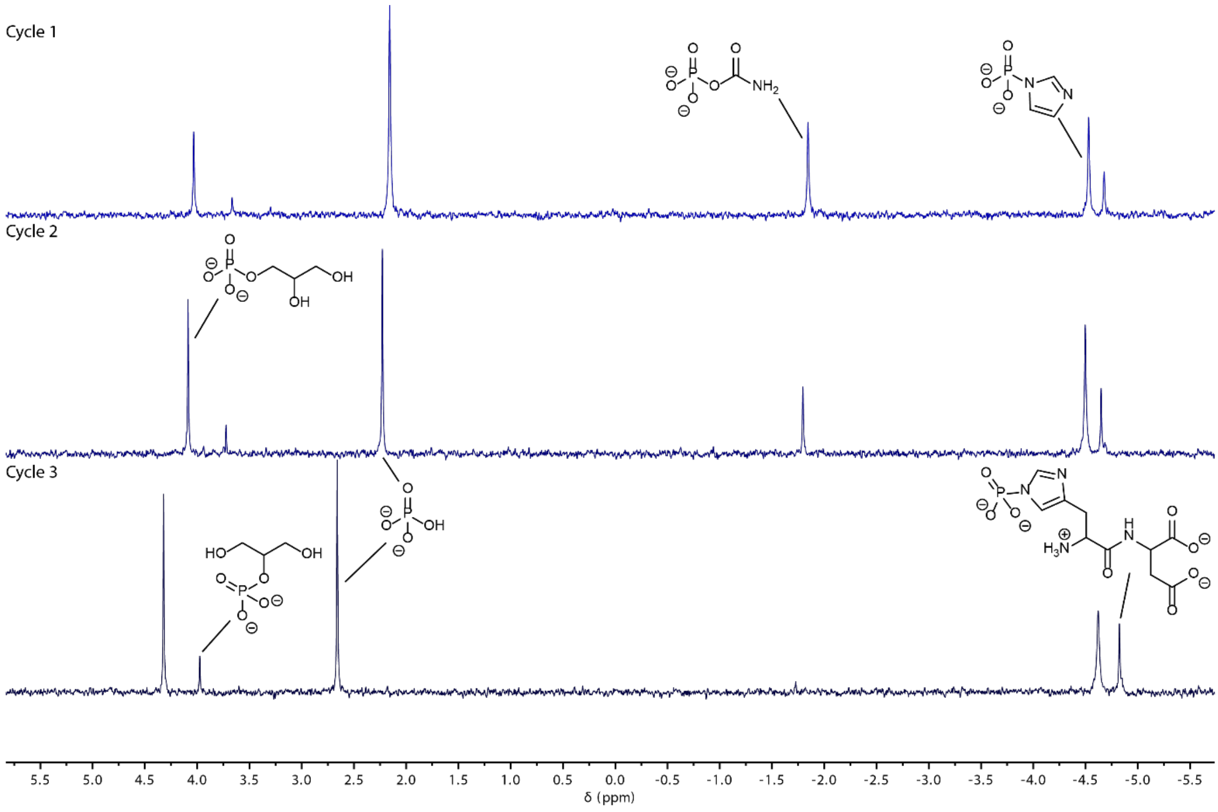

Supporting Figure 189: Representative ^{31}P-NMR spectra at the end of each cycle for the reaction of 20 mM sodium phosphate dibasic + 500 mM glycerol + 230 mM potassium cyanate + 100 mM imidazole + 100 mM His-Asp at pH 7.3 and 22 °C.

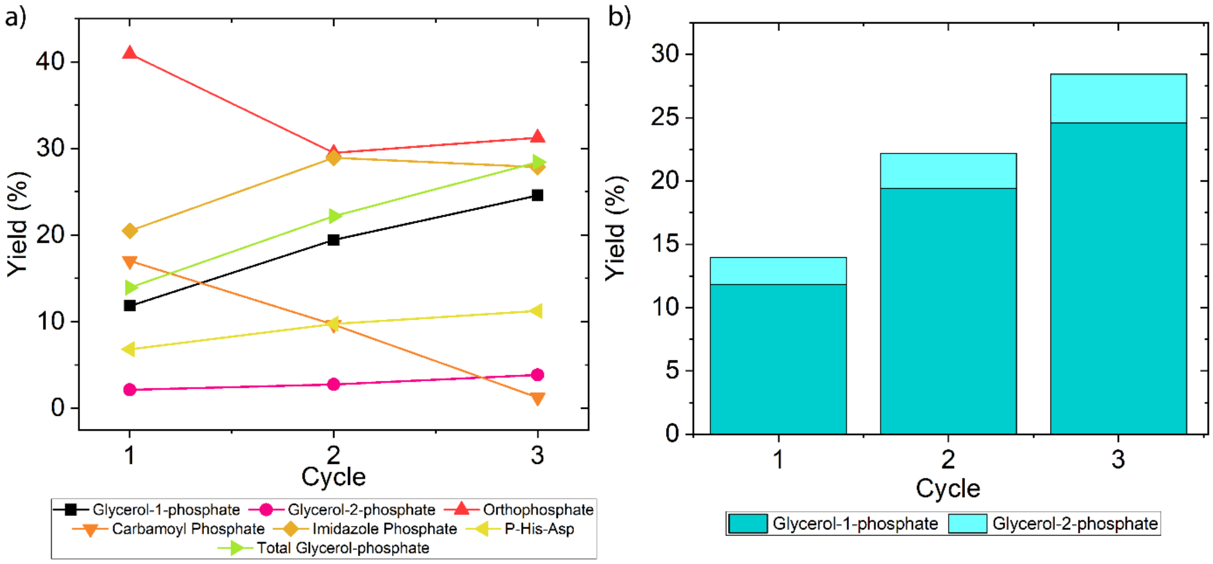

Supporting Figure 190: a) Changes in yield for all phosphate-containing products over three cycles for the reaction of 20 mM sodium phosphate dibasic + 500 mM glycerol + 230 mM potassium cyanate + 100 mM imidazole + 100 mM His-Asp at pH 7.3 and 22 °C. b) Change in yield for glycerol-1-phosphate and glycerol-2-phosphate over the course of three cycles.

S4.6.3 Wet/Dry cycle for the phosphorylation of glycerol catalysed by 100 mM His-Asp catalyst 2nd repeat

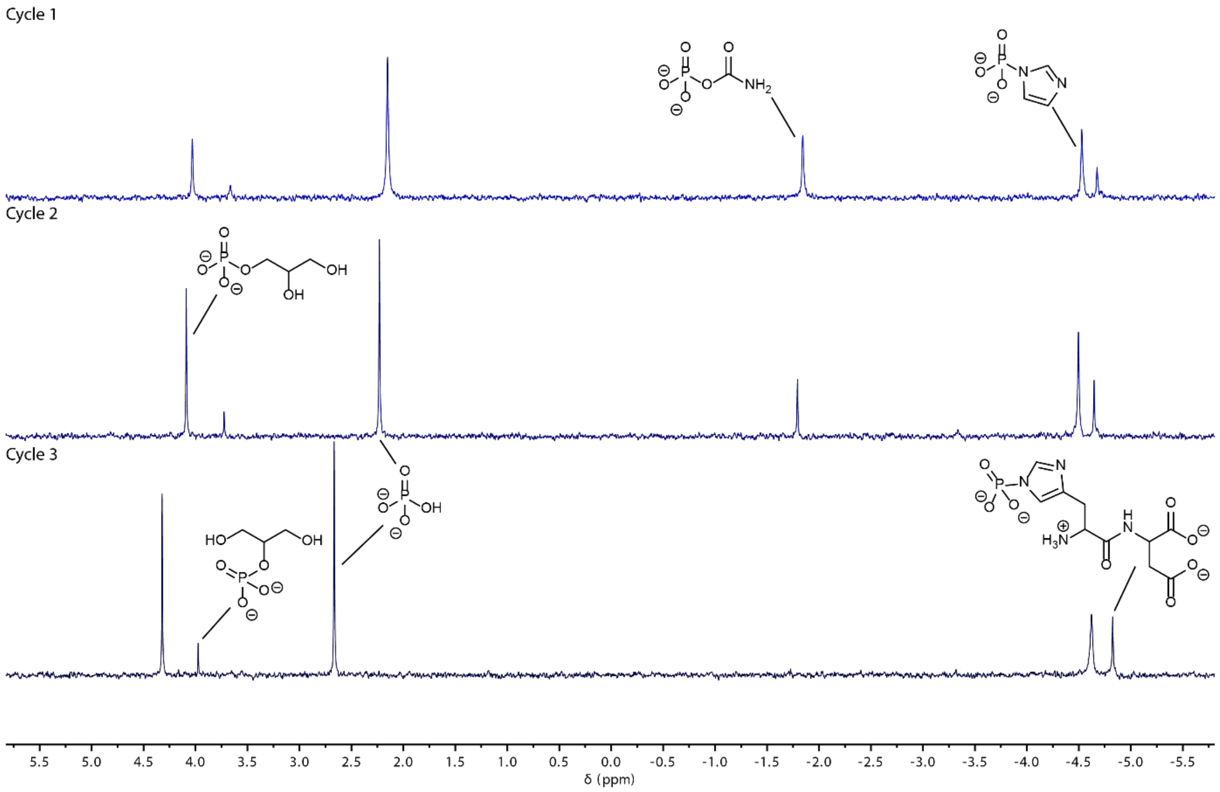

Supporting Figure 191: Representative ^{31}P-NMR spectra at the end of each cycle for the reaction of 20 mM sodium phosphate dibasic + 500 mM glycerol + 230 mM potassium cyanate + 100 mM imidazole + 100 mM His-Asp at pH 7.3 and 22 °C.

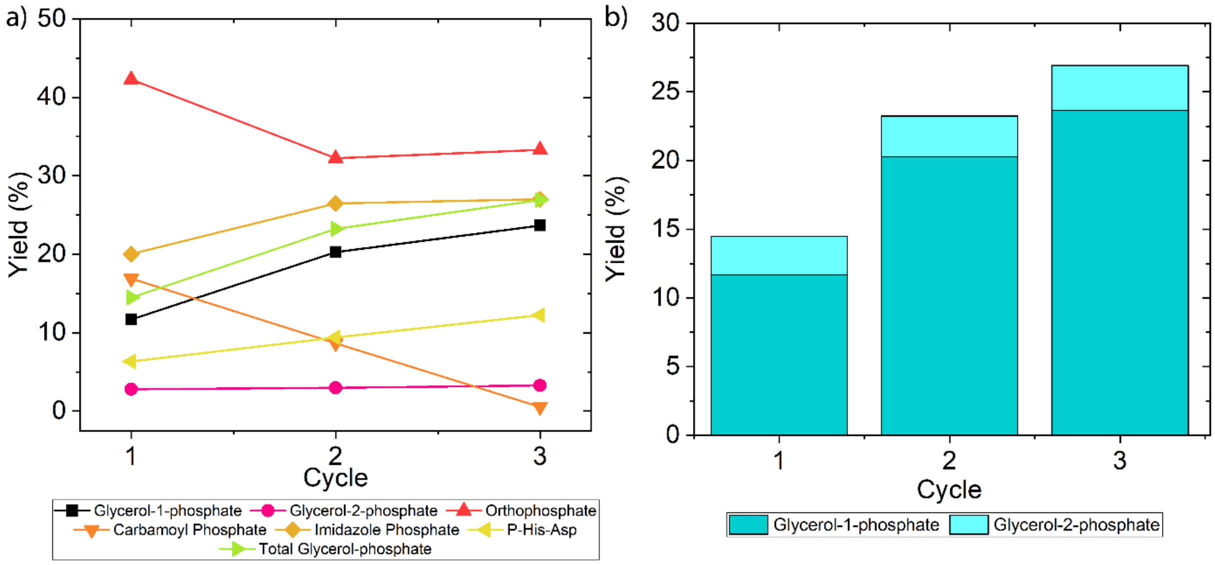

Supporting Figure 192: a) Changes in yield for all phosphate-containing products over three cycles for the reaction of 20 mM sodium phosphate dibasic + 500 mM glycerol + 230 mM potassium cyanate + 100 mM imidazole + 100 mM His-Asp at pH 7.3 and 22 °C. b) Change in yield for glycerol-1-phosphate and glycerol-2-phosphate over the course of three cycles.

S4.7 Wet/Dry Cycle for the phosphorylation of glycerol by imidazole phosphate with a His-Lys catalyst

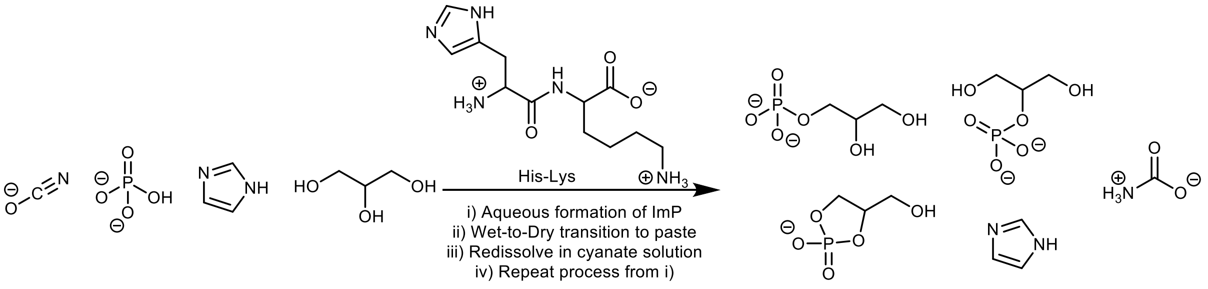

The experiment was carried out according to the procedure in S4.1 but with the 100 mM of histidine replaced by 100 mM His-Lys.HBr (145.7 mg, 0.4 mmol). The experiment was repeated in triplicate. Supporting Figures 193, 195 and 197 depict representative ^{31}P NMR spectra after each wet-dry cycle. The changes in yield after each wet-dry cycle for all phosphate containing species are shown in Supporting Figure 194a, 196a and 198a. The change in yield of glycerol-1-phosphate and glycerol-2-phosphate after each wet-dry cycle are shown in Supporting Figure 194b, 196b and 198b.

S4.7.1 Wet/Dry cycle for the phosphorylation of glycerol catalysed by 100 mM His-Lys catalyst

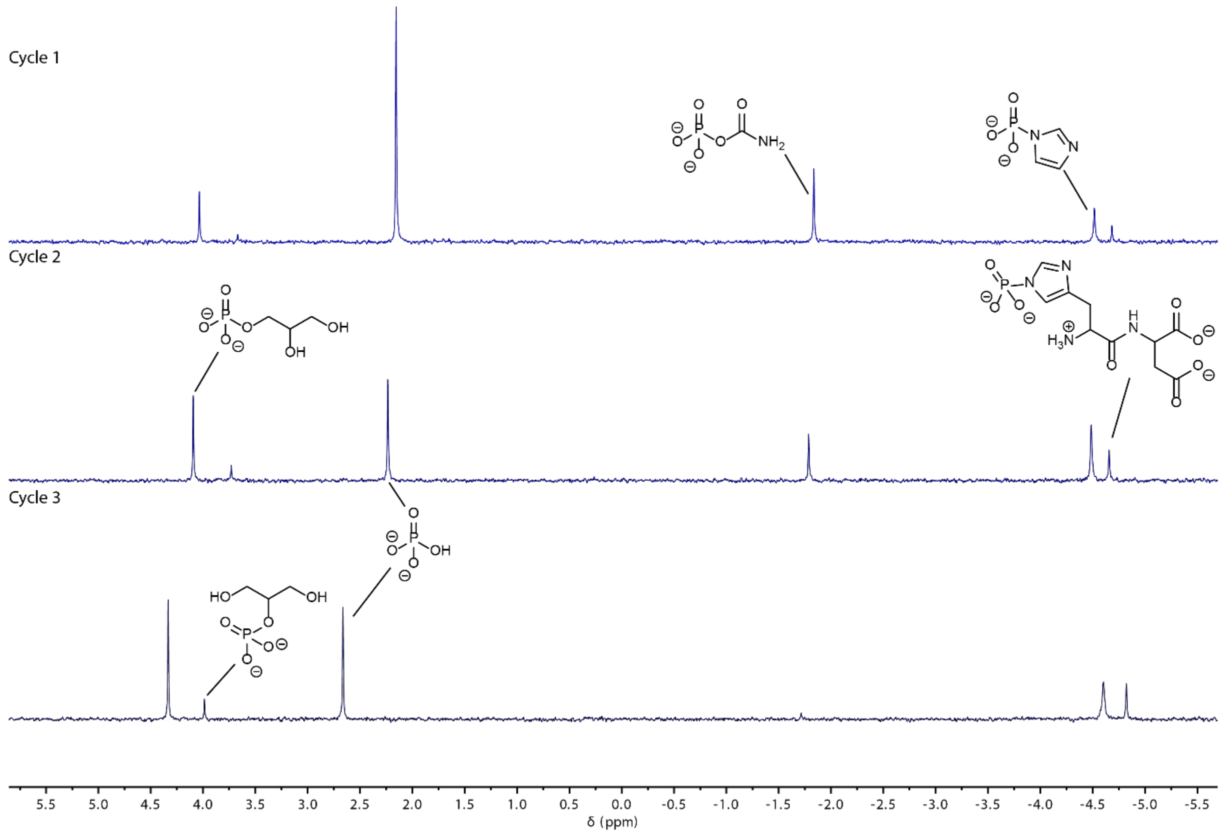

Supporting Figure 193: Representative ^{31}P-NMR spectra at the end of each cycle for the reaction of 20 mM sodium phosphate dibasic + 500 mM glycerol + 230 mM potassium cyanate + 100 mM imidazole + 100 mM His-Lys at pH 7.3 and 22 °C.

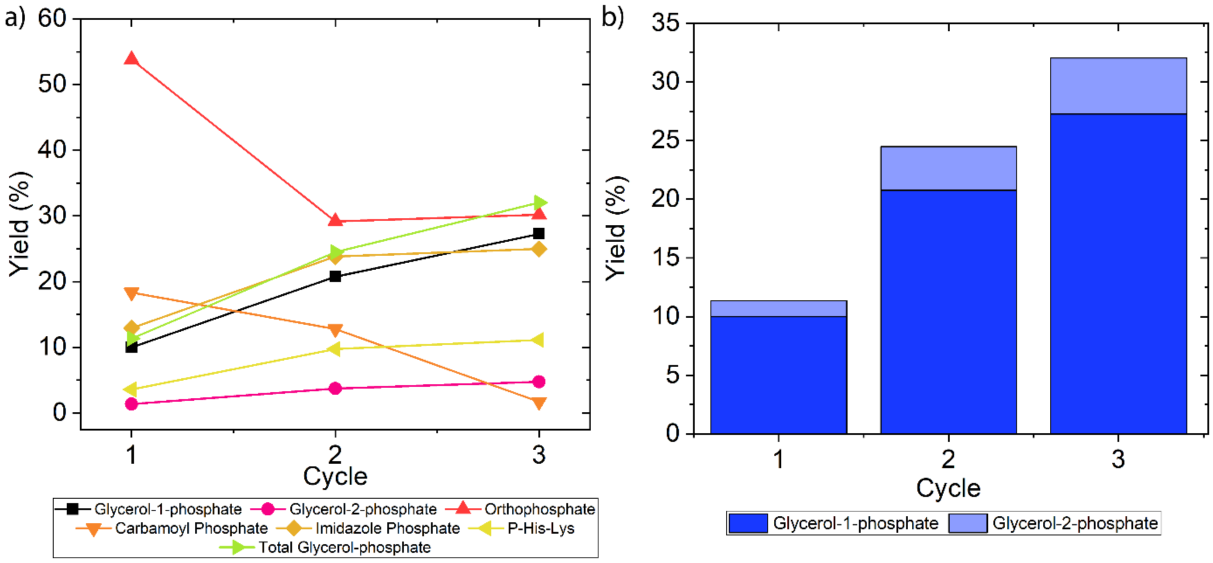

Supporting Figure 194: a) Changes in yield for all phosphate-containing products over three cycles for the reaction of 20 mM sodium phosphate dibasic + 500 mM glycerol + 230 mM potassium cyanate + 100 mM imidazole + 100 mM His-Lys at pH 7.3 and 22 °C. b) Change in yield for glycerol-1-phosphate and glycerol-2-phosphate over the course of three cycles.

S4.7.2 Wet/Dry cycle for the phosphorylation of glycerol catalysed by 100 mM His-Lys catalyst 1st repeat

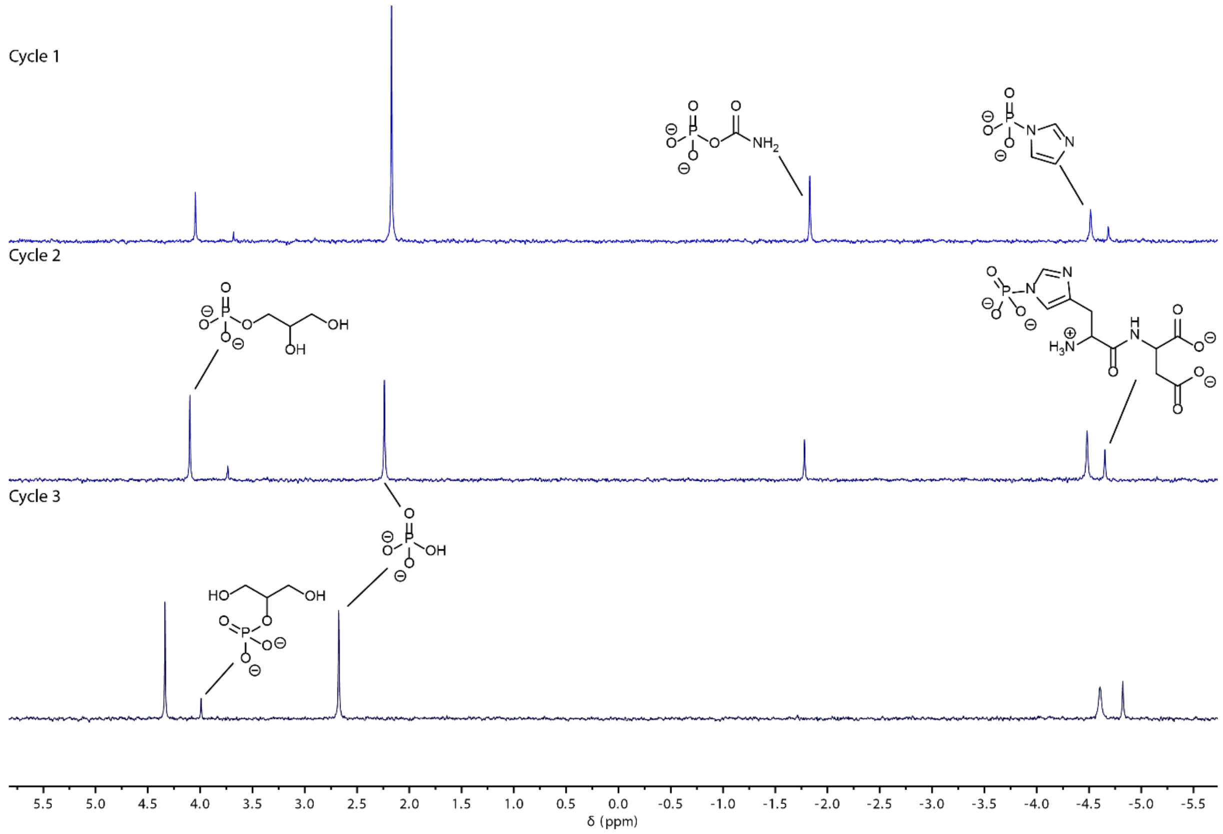

Supporting Figure 195: Representative ^{31}P-NMR spectra at the end of each cycle for the reaction of 20 mM sodium phosphate dibasic + 500 mM glycerol + 230 mM potassium cyanate + 100 mM imidazole + 100 mM His-Lys at pH 7.3 and 22 °C.

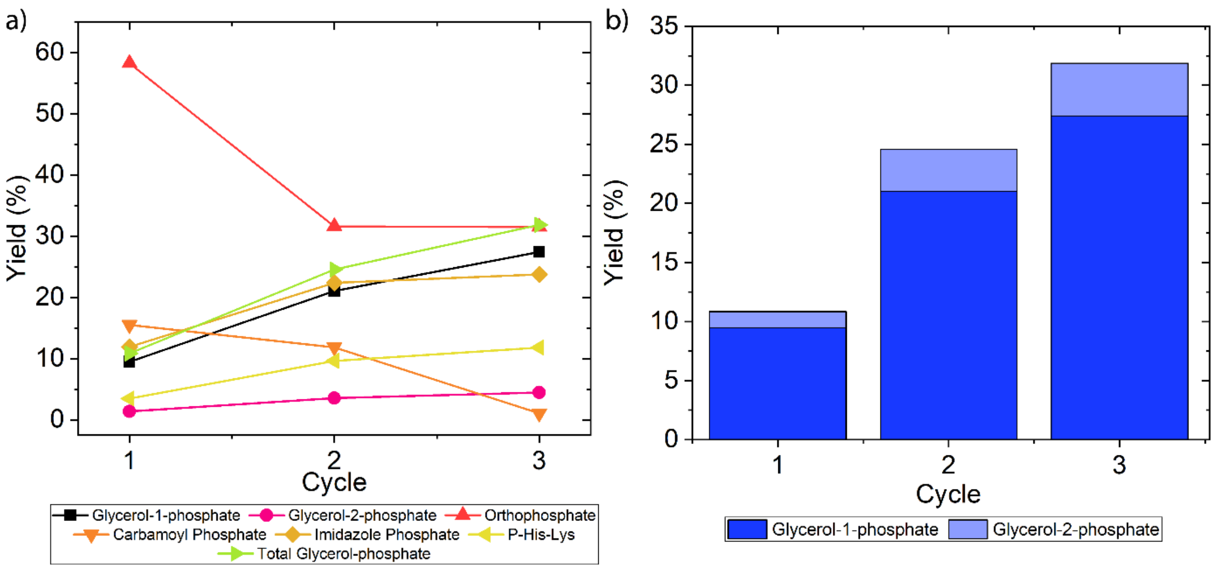

Supporting Figure 196: a) Changes in yield for all phosphate-containing products over three cycles for the reaction of 20 mM sodium phosphate dibasic + 500 mM glycerol + 230 mM potassium cyanate + 100 mM imidazole + 100 mM His-Lys at pH 7.3 and 22 °C. b) Change in yield for glycerol-1-phosphate and glycerol-2-phosphate over the course of three cycles.

S4.7.3 Wet/Dry cycle for the phosphorylation of glycerol catalysed by 100 mM His-Lys catalyst 2nd repeat

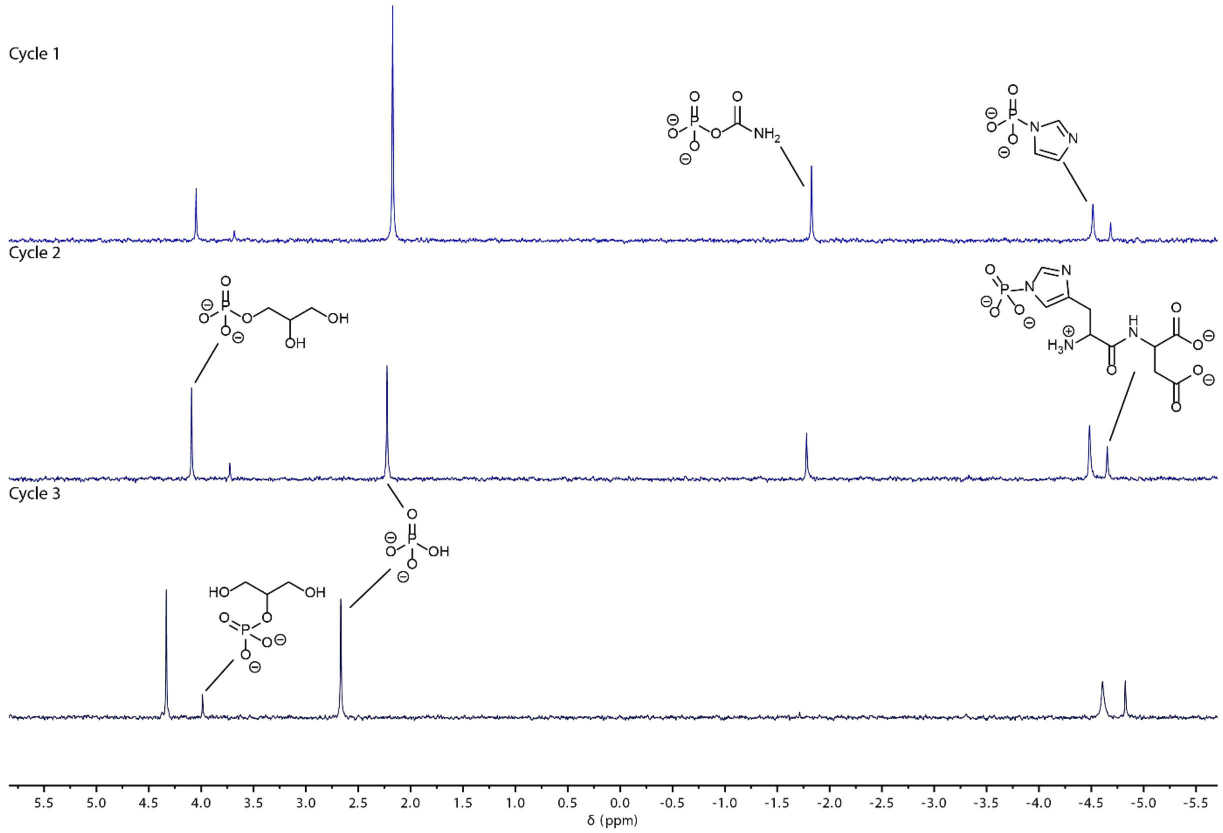

Supporting Figure 197: Representative ^{31}P-NMR spectra at the end of each cycle for the reaction of 20 mM sodium phosphate dibasic + 500 mM glycerol + 230 mM potassium cyanate + 100 mM imidazole + 100 mM His-Lys at pH 7.3 and 22 °C.

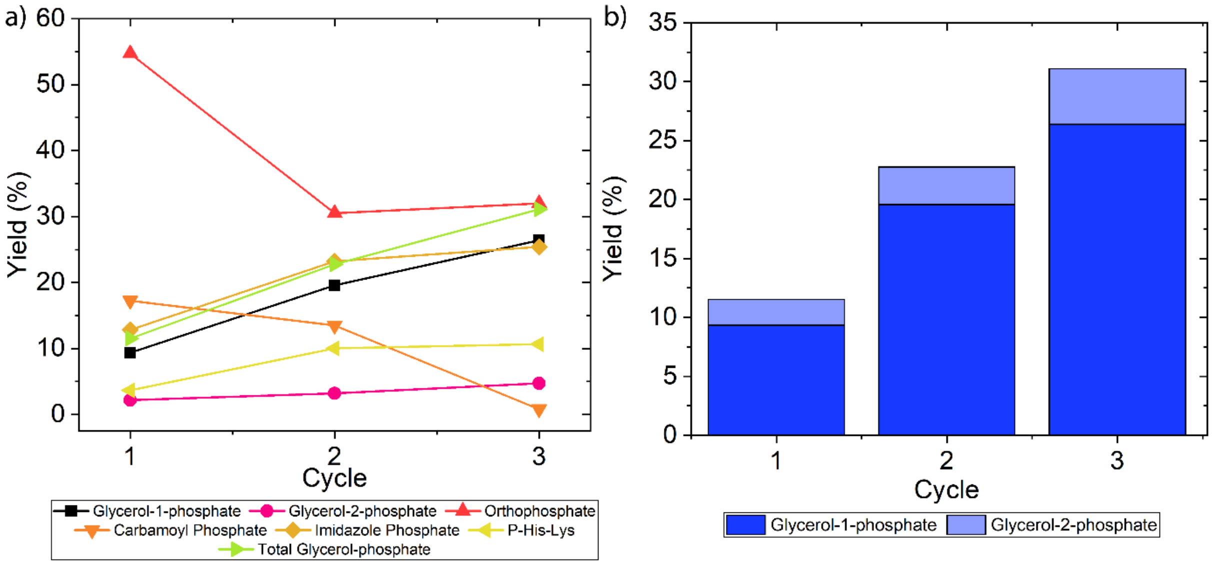

Supporting Figure 198: a) Changes in yield for all phosphate-containing products over three cycles for the reaction of 20 mM sodium phosphate dibasic + 500 mM glycerol + 230 mM potassium cyanate + 100 mM imidazole + 100 mM His-Lys at pH 7.3 and 22 °C. b) Change in yield for glycerol-1-phosphate and glycerol-2-phosphate over the course of three cycles.

S4.8 Wet/Dry Cycle for the phosphorylation of glycerol by imidazole phosphate with a His-Gly-Gly catalyst

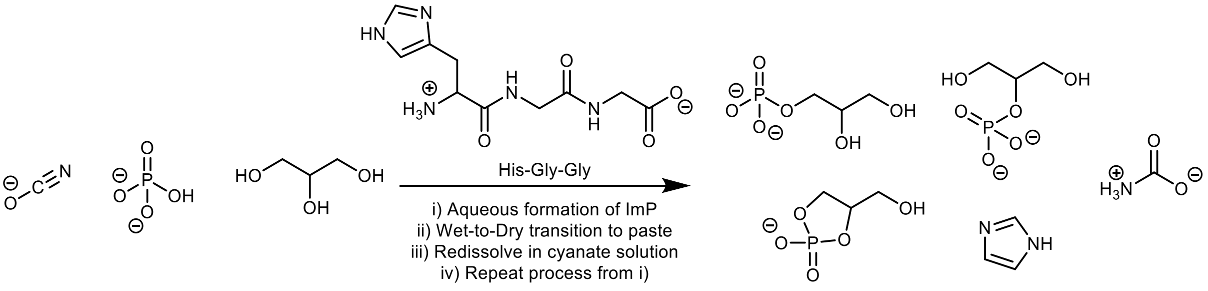

The experiment was carried out according to the procedure in S4.1 but with the 100 mM of histidine replaced by 100 mM His-Gly-Gly (107.7 mg, 0.4 mmol). The experiment was repeated in duplicate. Supporting Figures 199 and 201 depict representative ^{31}P NMR spectra after each wet-dry cycle. The changes in yield after each wet-dry cycle for all phosphate containing species are shown in Supporting Figure 200a and 202a. The change in yield of glycerol-1-phosphate and glycerol-2-phosphate after each wet-dry cycle are shown in Supporting Figure 200b and 202b.

S4.8.1 Wet/Dry cycle for the phosphorylation of glycerol catalysed by 100 mM His-Gly-Gly catalyst

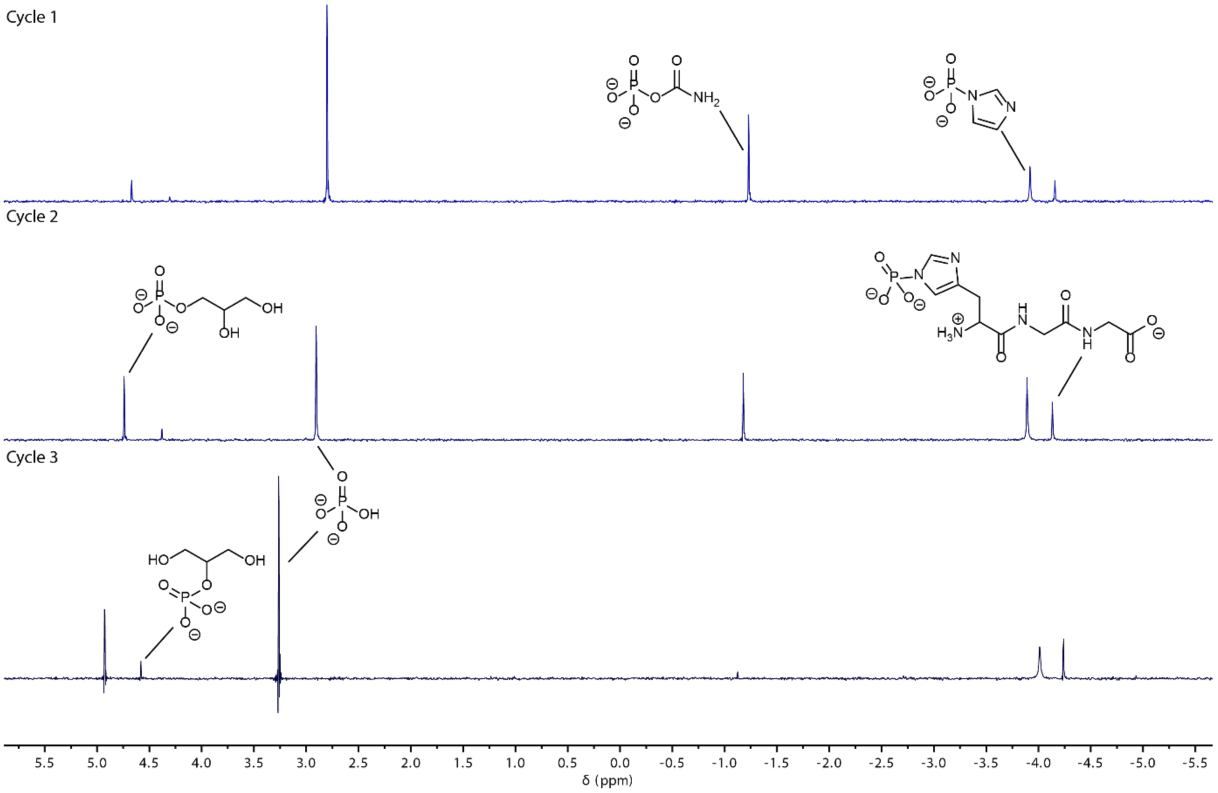

Supporting Figure 199: Representative ^{31}P-NMR spectra at the end of each cycle for the reaction of 20 mM sodium phosphate dibasic + 500 mM glycerol + 230 mM potassium cyanate + 100 mM imidazole + 100 mM His-Gly-Gly at pH 7.3 and 22 °C.

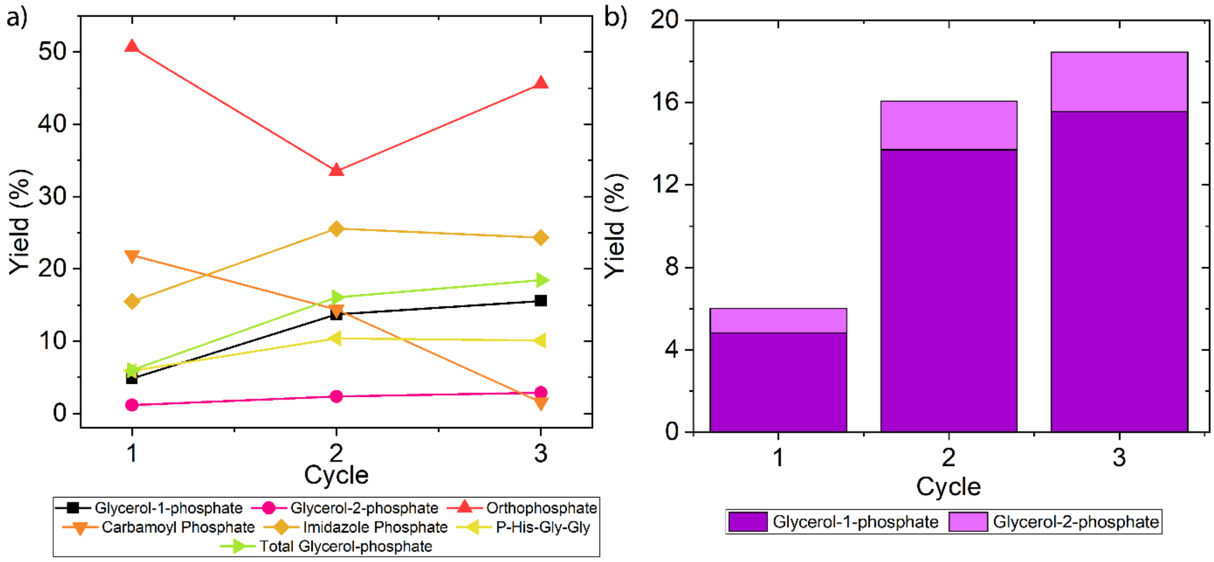

Supporting Figure 200: a) Changes in yield for all phosphate-containing products over three cycles for the reaction of 20 mM sodium phosphate dibasic + 500 mM glycerol + 230 mM potassium cyanate + 100 mM imidazole + 100 mM His-Gly-Gly at pH 7.3 and 22 °C. b) Change in yield for glycerol-1-phosphate and glycerol-2-phosphate over the course of three cycles.

S4.8.2 Wet/Dry cycle for the phosphorylation of glycerol catalysed by 100 mM His-Gly-Gly catalyst 1st repeat

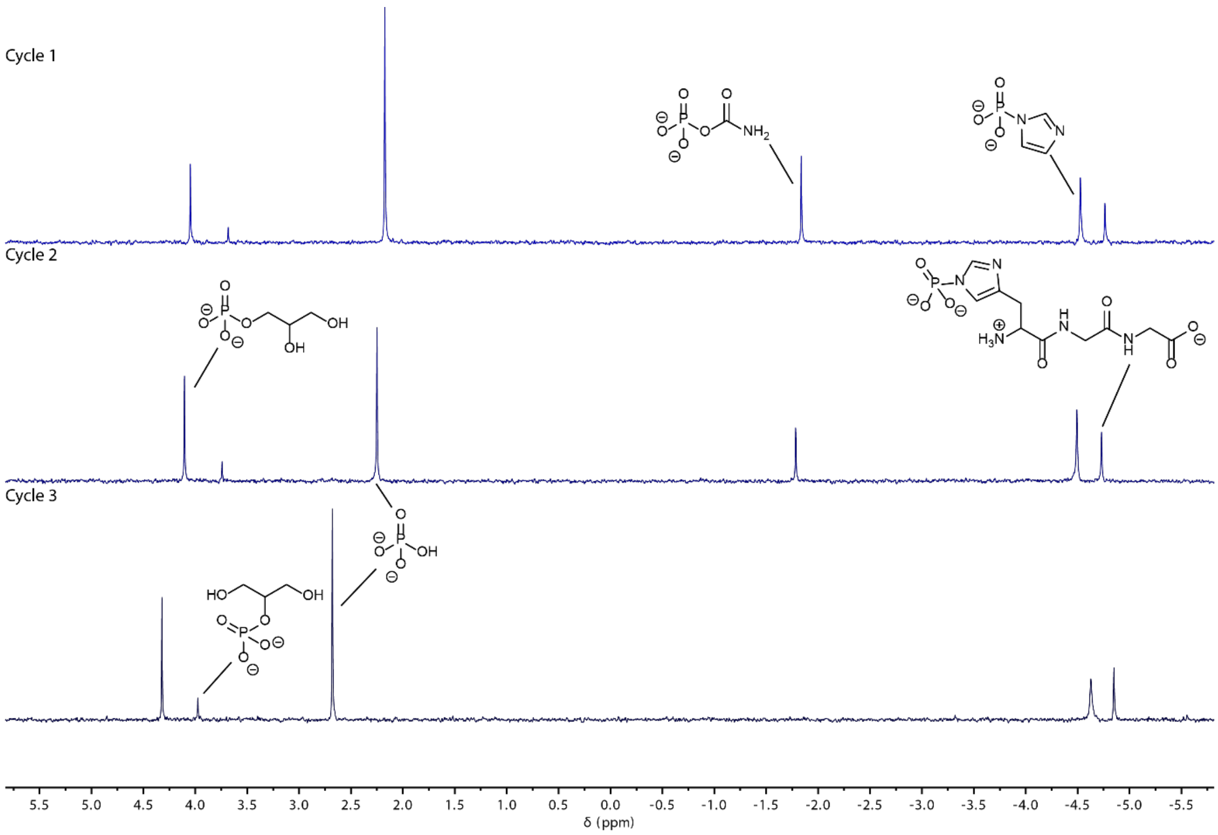

Supporting Figure 201: Representative ^{31}P-NMR spectra at the end of each cycle for the reaction of 20 mM sodium phosphate dibasic + 500 mM glycerol + 230 mM potassium cyanate + 100 mM imidazole + 100 mM His-Gly-Gly at pH 7.3 and 22 °C.

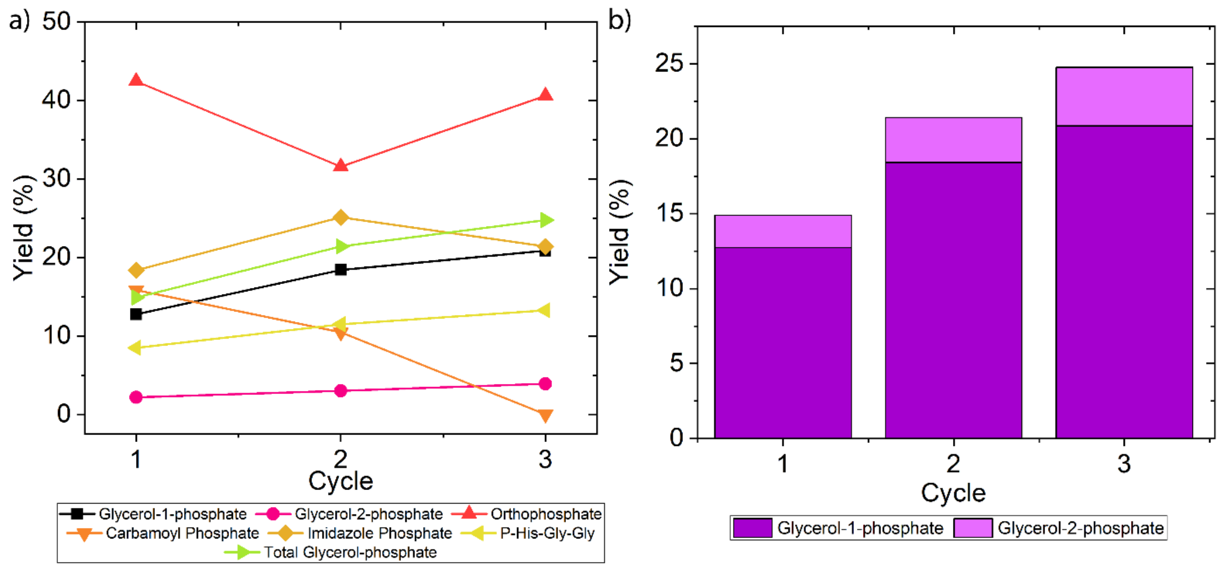

Supporting Figure 202: a) Changes in yield for all phosphate-containing products over three cycles for the reaction of 20 mM sodium phosphate dibasic + 500 mM glycerol + 230 mM potassium cyanate + 100 mM imidazole + 100 mM His-Gly-Gly at pH 7.3 and 22 °C. b) Change in yield for glycerol-1-phosphate and glycerol-2-phosphate over the course of three cycles.

S4.9 Wet/Dry Cycle with phosphorylation of glycerol by imidazole phosphate – uncatalysed reaction

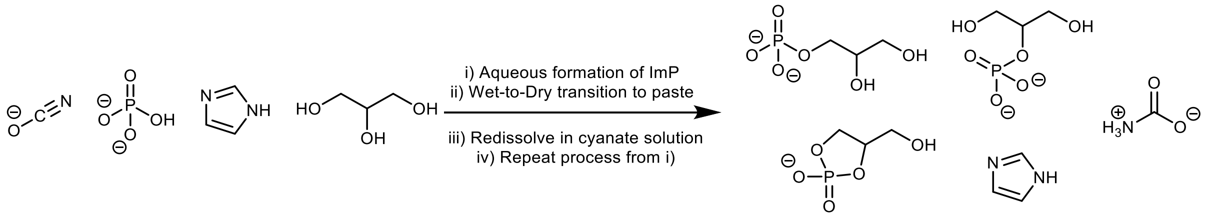

The experiment was carried out according to the procedure in S4.1 but with no 100 mM of histidine. The experiment was repeated in triplicate. Supporting Figures 203, 205 and 207 depict representative ^{31}P NMR spectra after each wet-dry cycle. The changes in yield after each wet-dry cycle for all phosphate containing species are shown in Supporting Figure 204a, 206a and 208a. The change in yield of glycerol-1-phosphate and glycerol-2-phosphate after each wet-dry cycle are shown in Supporting Figure 204b, 206b and 208b.

S4.9.1 Wet/Dry cycle for the phosphorylation of glycerol catalysed uncatalysed reaction

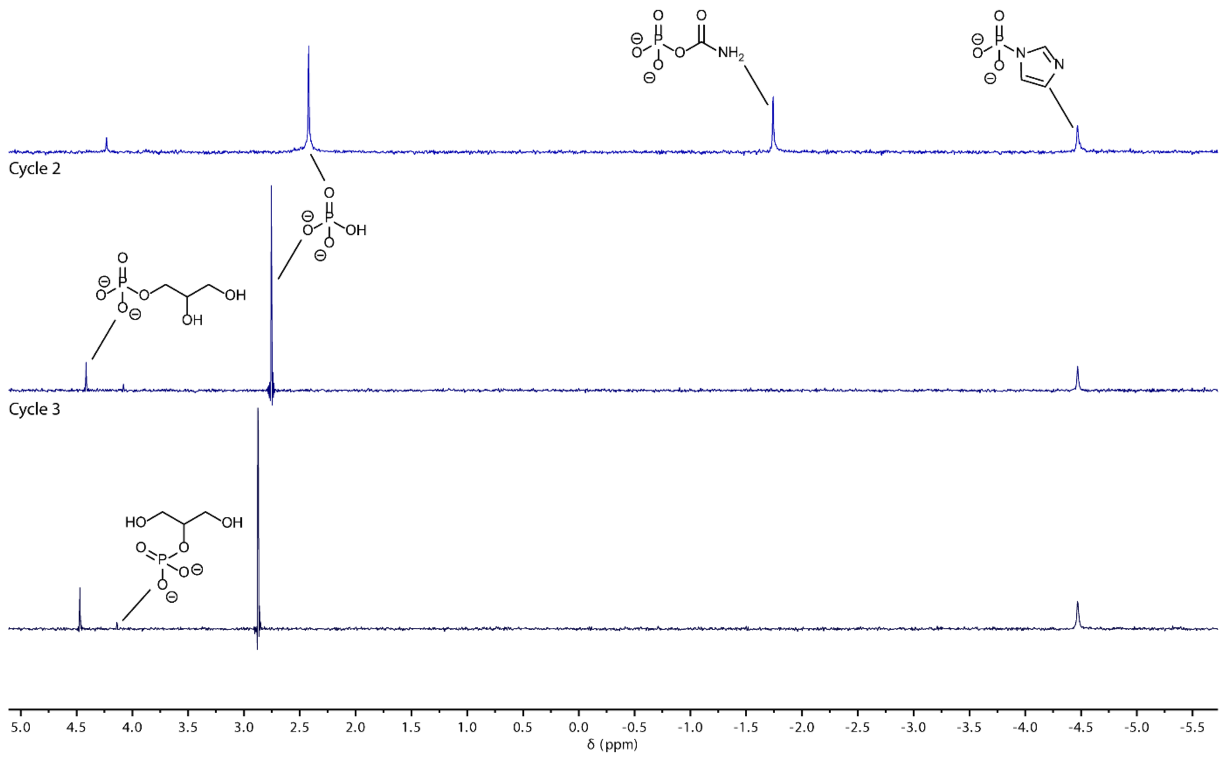

Supporting Figure 203: Representative ^{31}P-NMR spectra at the end of each cycle for the reaction of 20 mM sodium phosphate dibasic + 500 mM glycerol + 230 mM potassium cyanate + 100 mM imidazole at pH 7.3 and 22 °C.

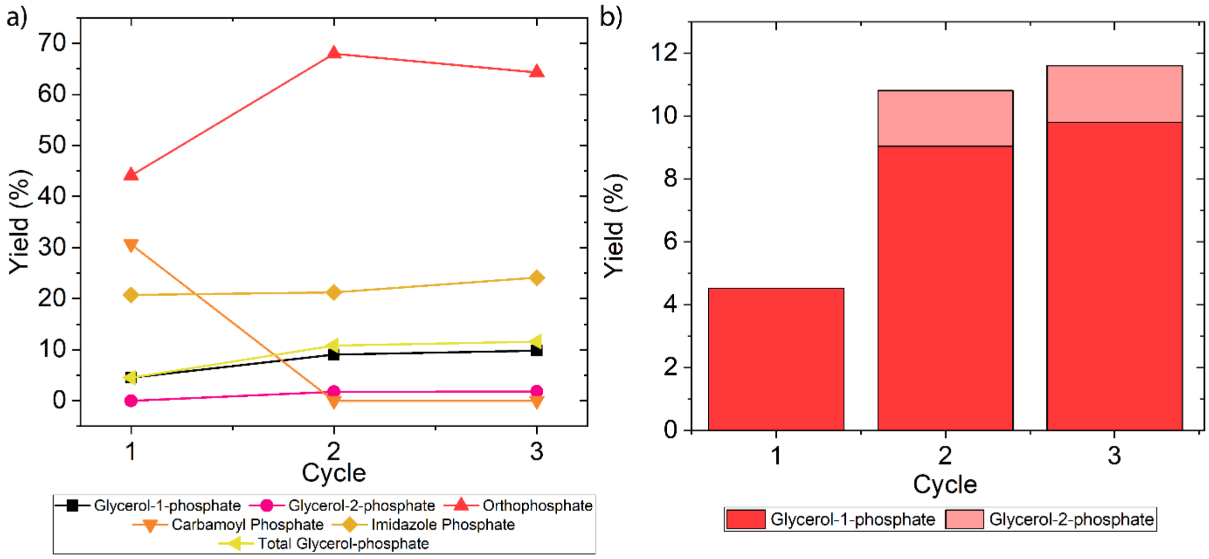

Supporting Figure 204: a) Changes in yield for all phosphate-containing products over three cycles for the reaction of 20 mM sodium phosphate dibasic + 500 mM glycerol + 230 mM potassium cyanate + 100 mM imidazole at pH 7.3 and 22 °C. b) Change in yield for glycerol-1-phosphate and glycerol-2-phosphate over the course of three cycles.

S4.9.2 Wet/Dry cycle for the phosphorylation of glycerol catalysed uncatalysed reaction 1st repeat

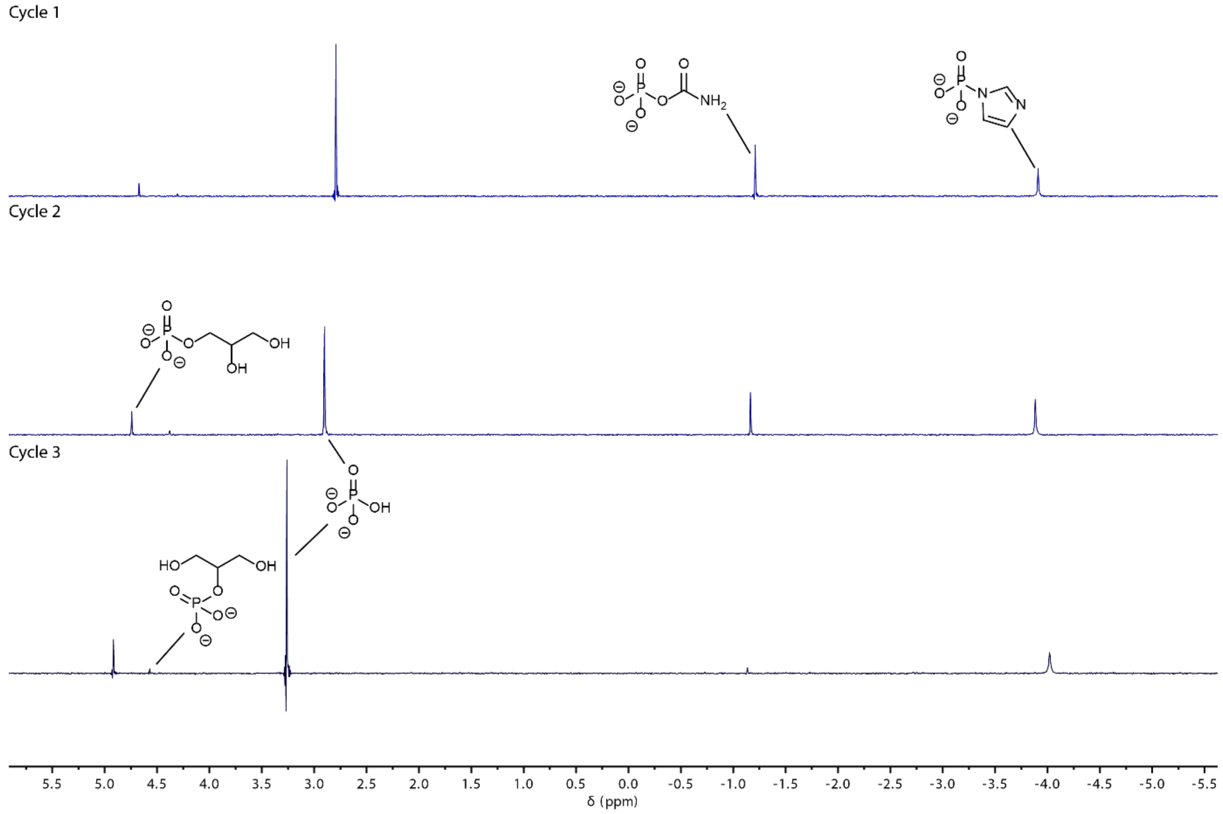

Supporting Figure 205: Representative ^{31}P-NMR spectra at the end of each cycle for the reaction of 20 mM sodium phosphate dibasic + 500 mM glycerol + 230 mM potassium cyanate + 100 mM imidazole at pH 7.3 and 22 °C.

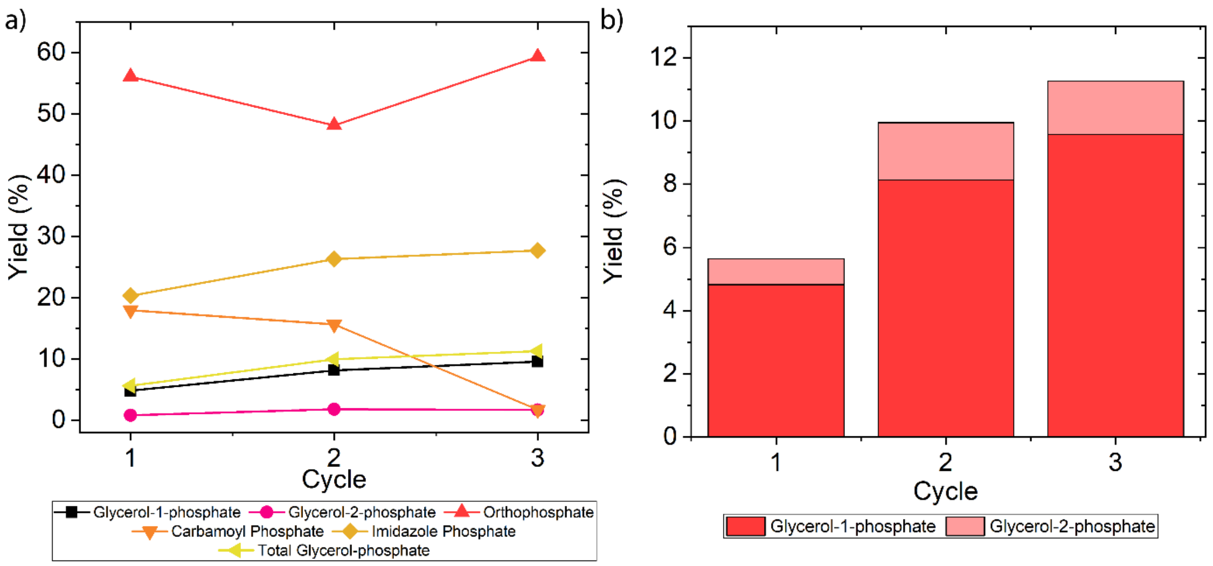

Supporting Figure 206: a) Changes in yield for all phosphate-containing products over three cycles for the reaction of 20 mM sodium phosphate dibasic + 500 mM glycerol + 230 mM potassium cyanate + 100 mM imidazole at pH 7.3 and 22 °C. b) Change in yield for glycerol-1-phosphate and glycerol-2-phosphate over the course of three cycles.

S4.9.3 Wet/Dry cycle for the phosphorylation of glycerol catalysed uncatalysed reaction 2nd repeat

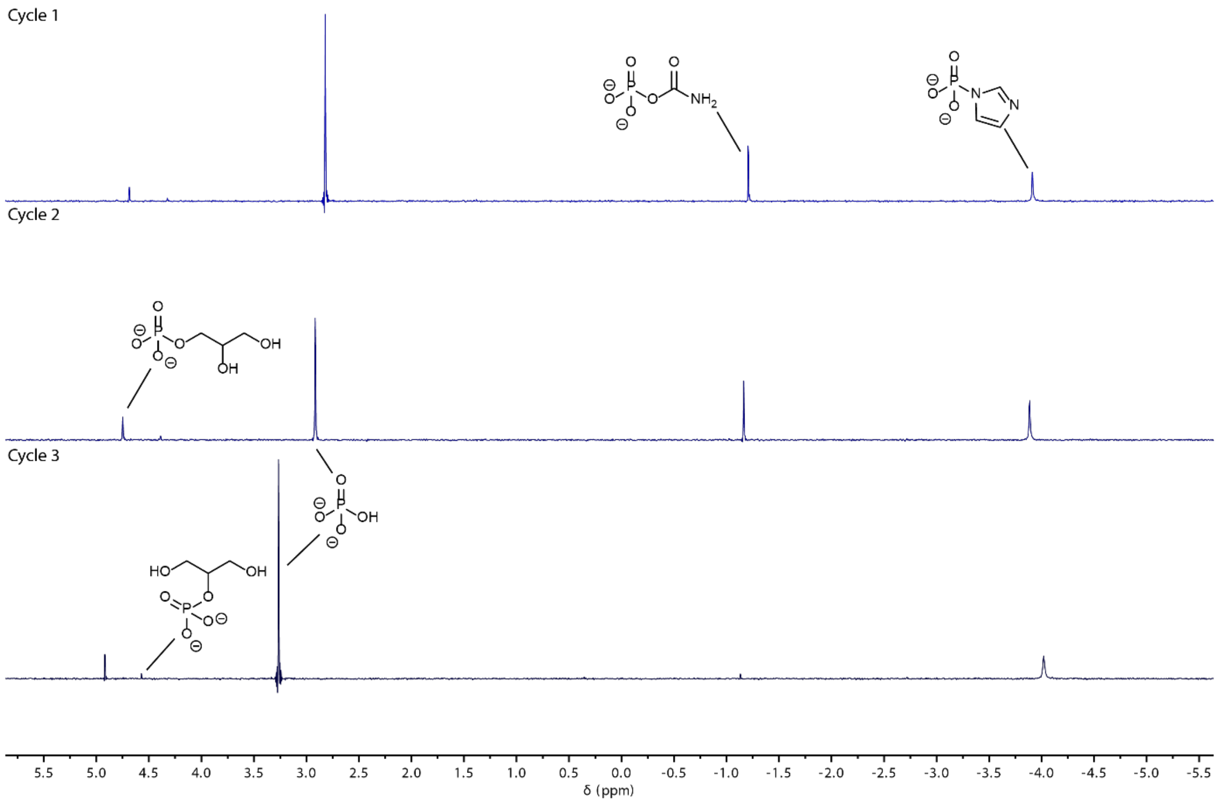

Supporting Figure 207: Representative ^{31}P-NMR spectra at the end of each cycle for the reaction of 20 mM sodium phosphate dibasic + 500 mM glycerol + 230 mM potassium cyanate + 100 mM imidazole at pH 7.3 and 22 °C.

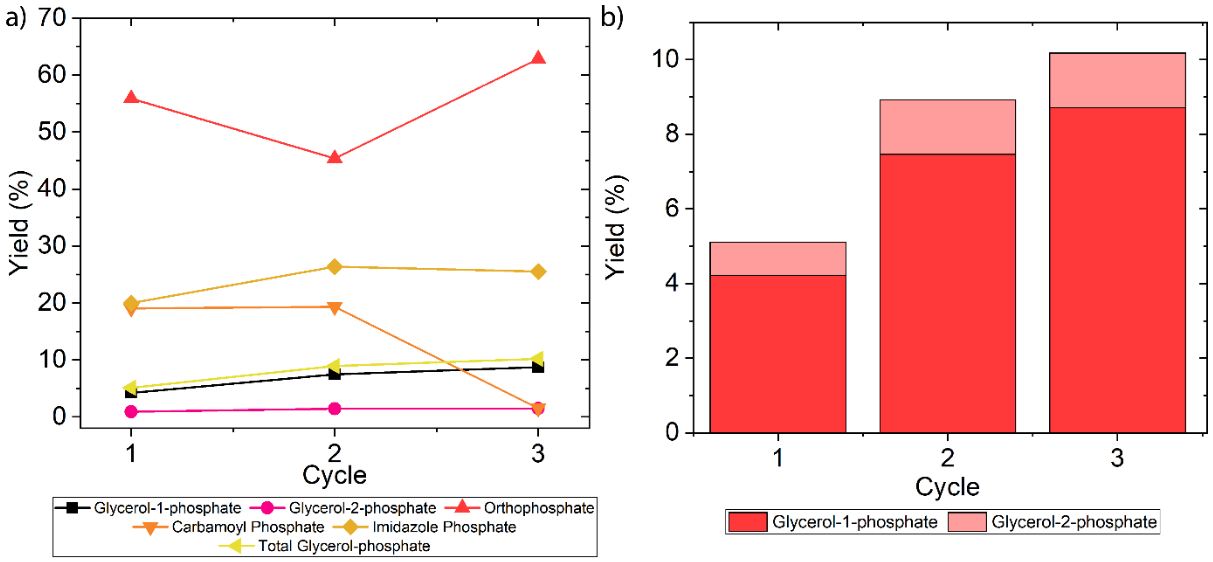

Supporting Figure 208: a) Changes in yield for all phosphate-containing products over three cycles for the reaction of 20 mM sodium phosphate dibasic + 500 mM glycerol + 230 mM potassium cyanate + 100 mM imidazole at pH 7.3 and 22 °C. b) Change in yield for glycerol-1-phosphate and glycerol-2-phosphate over the course of three cycles.

S4.10 Wet/Dry Cycle for the phosphorylation of glycerol with 100 mM histidine catalyst and no imidazole present

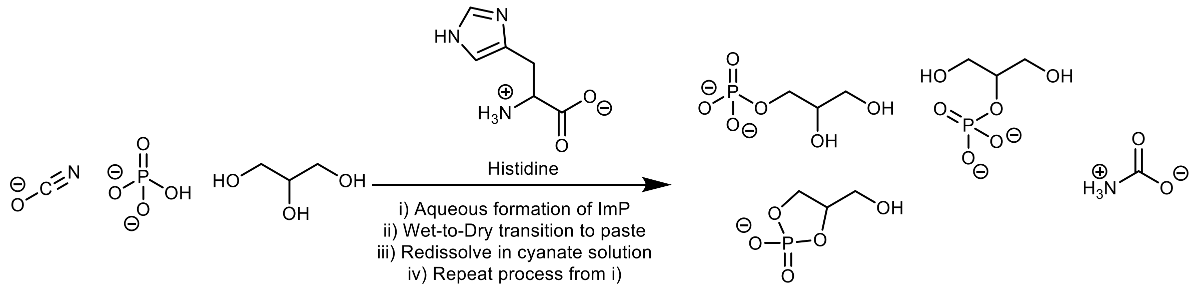

The experiment was carried out according to the procedure in S4.1 but with the 100 mM of imidazole removed. The experiment was repeated in triplicate. Supporting Figures 209, 211 and 213 depict representative ^{31}P NMR spectra after each wet-dry cycle. The changes in yield after each wet-dry cycle for all phosphate containing species are shown in Supporting Figure 210a, 212a and 214a. The change in yield of glycerol-1-phosphate and glycerol-2-phosphate after each wet-dry cycle are shown in Supporting Figure 210b, 212b and 214b.

S4.10.1 Wet/Dry cycle for the phosphorylation of glycerol catalysed by 100 mM histidine catalyst and no imidazole present

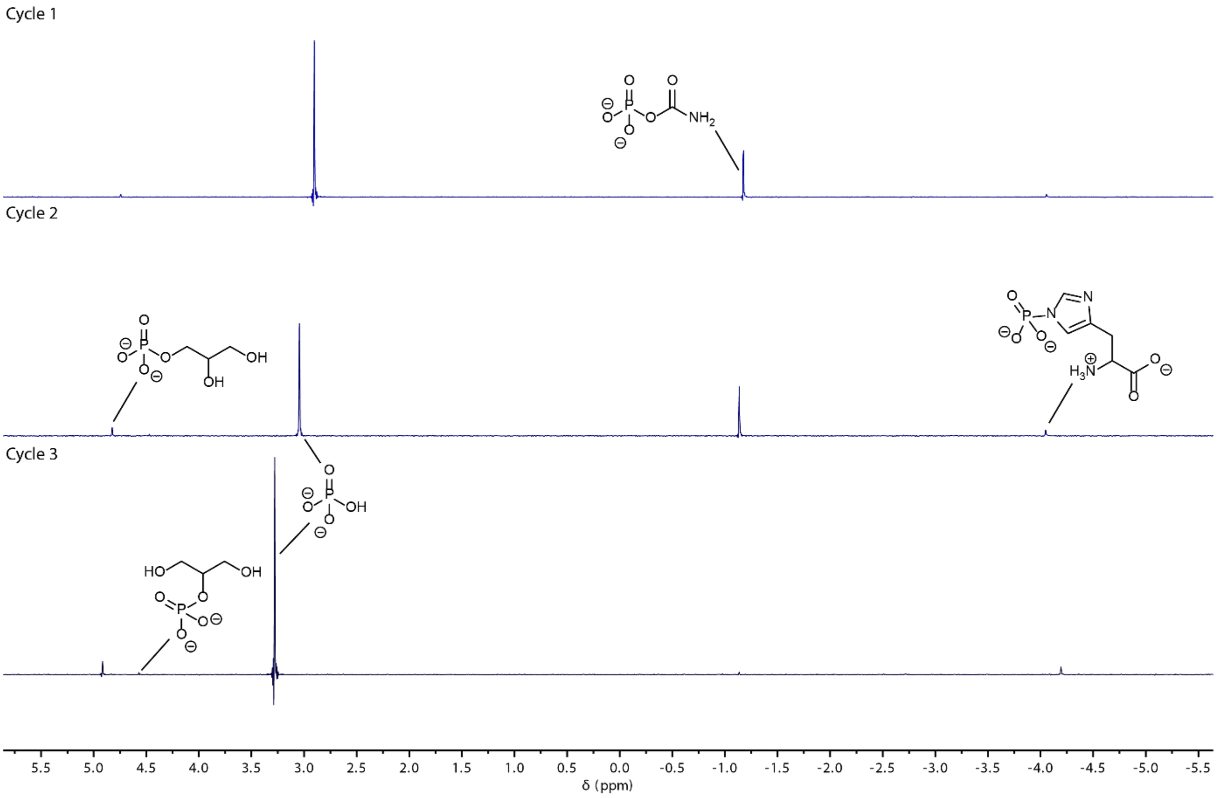

Supporting Figure 209: Representative ^{31}P-NMR spectra at the end of each cycle for the reaction of 20 mM sodium phosphate dibasic + 500 mM glycerol + 230 mM potassium cyanate + 100 mM histidine at pH 7.3 and 22 °C.

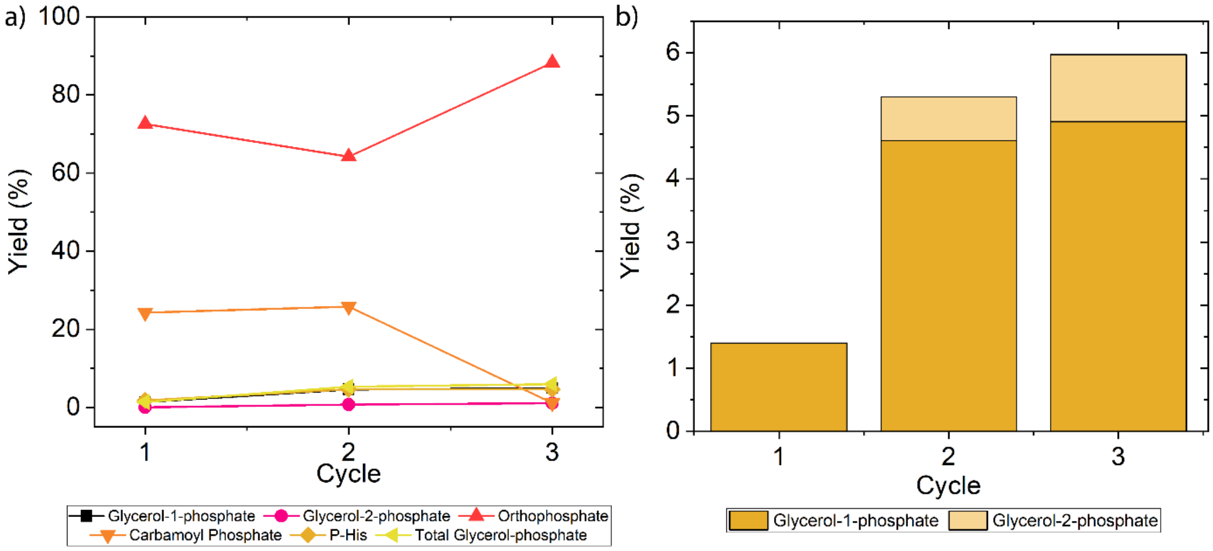

Supporting Figure 210: a) Changes in yield for all phosphate-containing products over three cycles for the reaction of 20 mM sodium phosphate dibasic + 500 mM glycerol + 230 mM potassium cyanate + 100 mM histidine at pH 7.3 and 22 °C. b) Change in yield for glycerol-1-phosphate and glycerol-2-phosphate over the course of three cycles.

S4.10.2 Wet/Dry cycle for the phosphorylation of glycerol catalysed by 100 mM histidine catalyst and no imidazole present

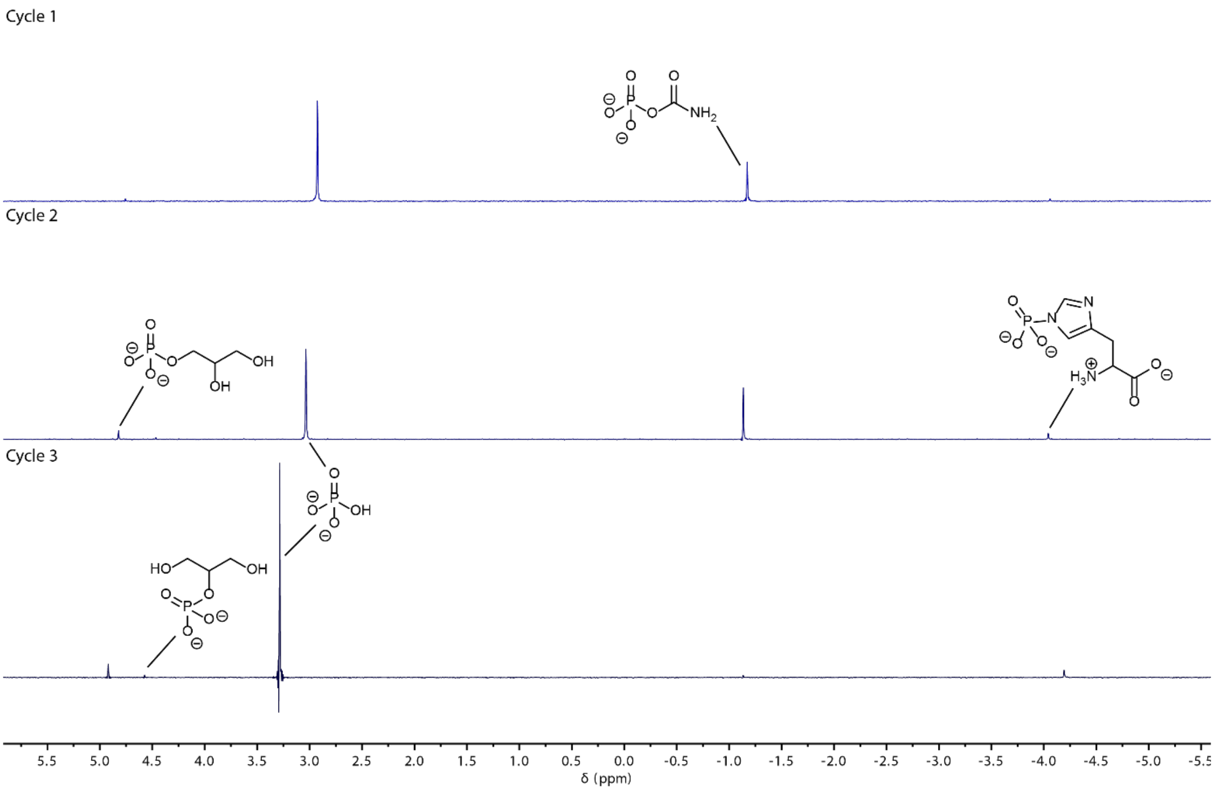

Supporting Figure 211: Representative ^{31}P-NMR spectra at the end of each cycle for the reaction of 20 mM sodium phosphate dibasic + 500 mM glycerol + 230 mM potassium cyanate + 100 mM histidine at pH 7.3 and 22 °C.

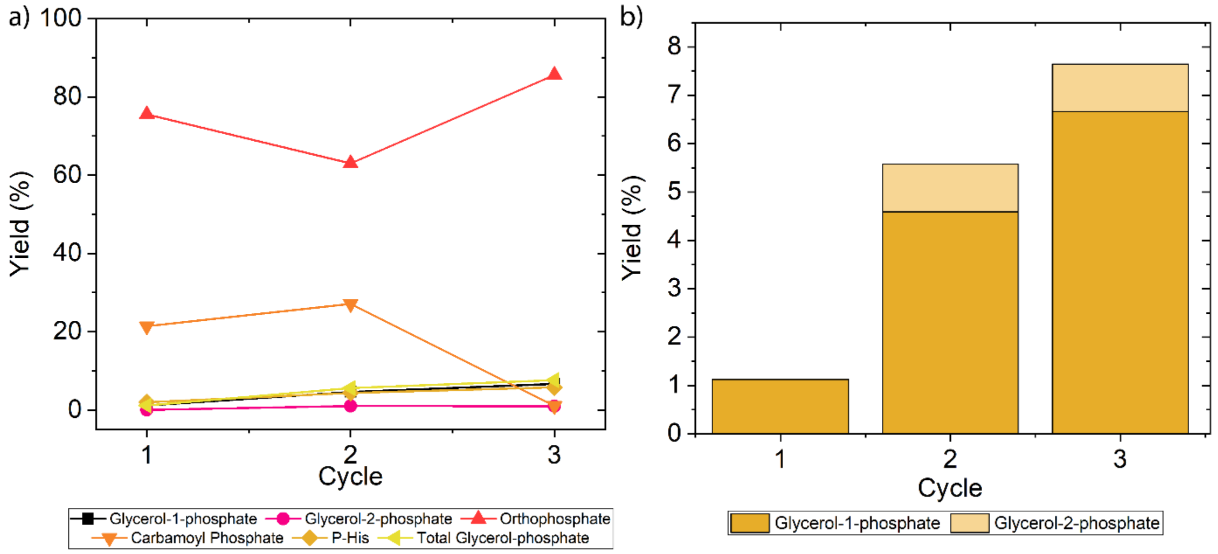

Supporting Figure 212: a) Changes in yield for all phosphate-containing products over three cycles for the reaction of 20 mM sodium phosphate dibasic + 500 mM glycerol + 230 mM potassium cyanate + 100 mM histidine at pH 7.3 and 22 °C. b) Change in yield for glycerol-1-phosphate and glycerol-2-phosphate over the course of three cycles.

S4.10.3 Wet/Dry cycle for the phosphorylation of glycerol catalysed by 100 mM histidine catalyst and no imidazole present

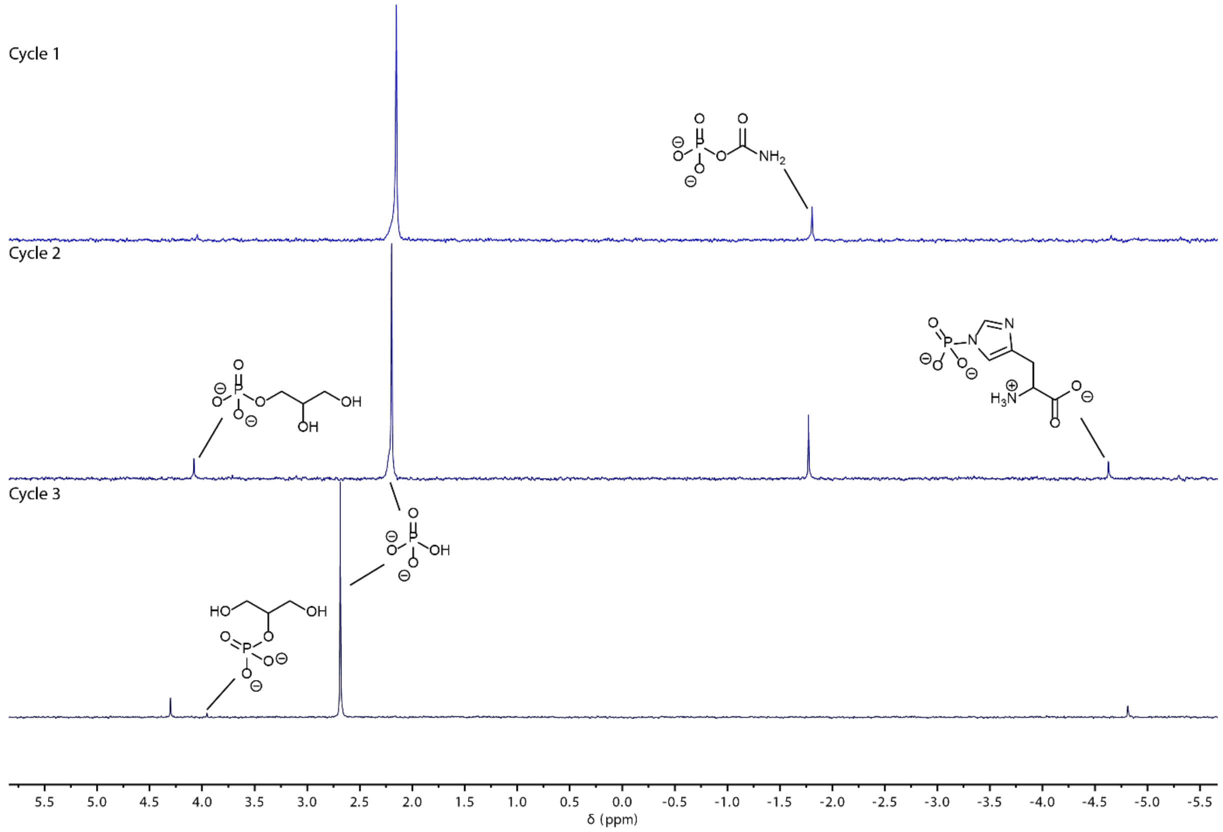

Supporting Figure 213: Representative ^{31}P-NMR spectra at the end of each cycle for the reaction of 20 mM sodium phosphate dibasic + 500 mM glycerol + 230 mM potassium cyanate + 100 mM histidine at pH 7.3 and 22 °C.

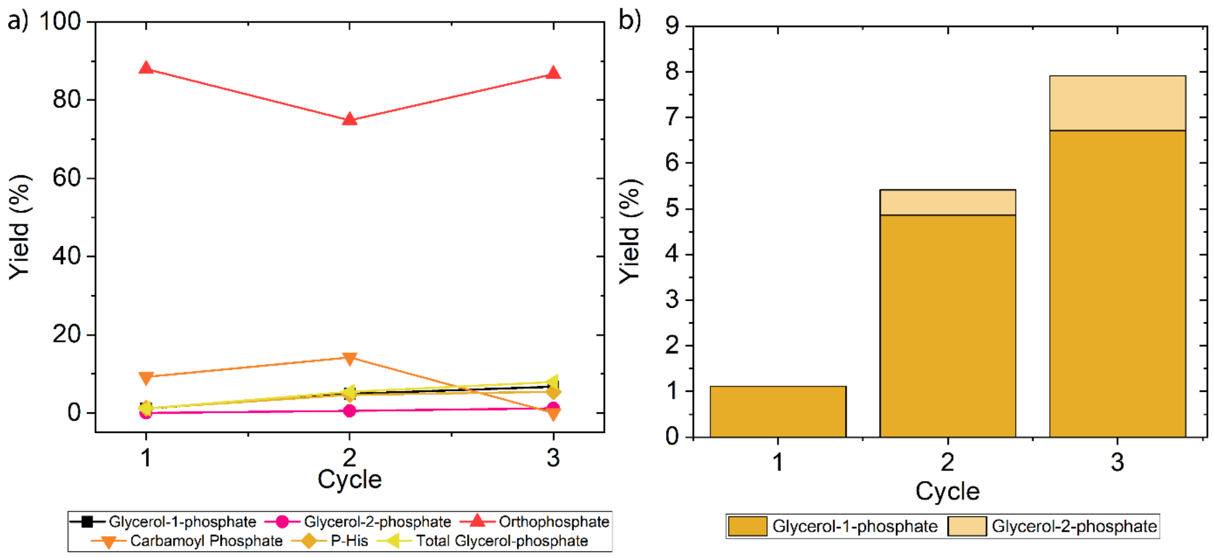

Supporting Figure 214: a) Changes in yield for all phosphate-containing products over three cycles for the reaction of 20 mM sodium phosphate dibasic + 500 mM glycerol + 230 mM potassium cyanate + 100 mM histidine at pH 7.3 and 22 °C. b) Change in yield for glycerol-1-phosphate and glycerol-2-phosphate over the course of three cycles.

S4.11 Experimental Method with glycerate

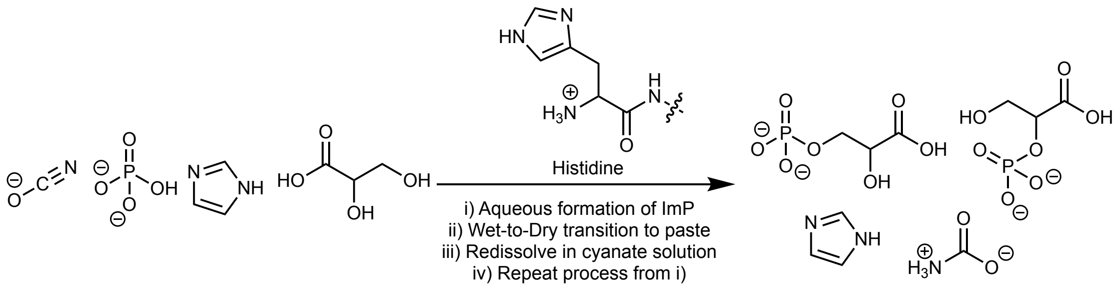

A 4 mL solution of 20 mM sodium phosphate dibasic + 500 mM glycerate + 230 mM potassium cyanate + 100 mM imidazole + 100 mM histidine was prepared by dissolving 14.2 mg (0.08 mmol) of sodium phosphate dibasic dihydrate, 212.2 mg (2.00 mmol) of glyceric acid, 74.4 mg (0.92 mmol) of potassium cyanate, 36.8 mg (0.40 mmol) of imidazole and 62.1 mg (0.40 mmol) of histidine in 9 : 1 H_2O : D_2O. The pH of the solution was adjusted to pH 7.3 with 5.0 M HCl and 5.0 M KOH solution. For the first wet-dry cycle, the solution was left for 24 h and then added to a petri dish and left with the lid off to dry at 22 °C for 24 h in a fume cupboard. To begin the second wet-dry cycle, the resulting paste was dissolved in 4 mL of 230 mM cyanate solution (prepared as above) in 9 : 1 H_2O : D_2O. The pH of the solution was adjusted to pH 7.3 with 5.0 M HCl and 5.0 M KOH solution. Immediately after this, the ^{31}P and ^{1}H NMR spectra were measured using 0.5 mL of this solution in order to determine the quantity of phosphorylated species formed in the first wet-dry cycle. After which the 0.5 mL of solution was recombined with the rest of the solution. The second wet-dry cycle followed the same procedure as for the first wet-dry cycle except that the drying of the solution was left for 48 h. Once the second wet-dry cycle was complete a third wet-dry cycle was initiated again using the procedure of the first wet-dry cycle except that the drying of the solution was left for 48 h.

S4.12 Wet/Dry Cycle for the phosphorylation of glycerate by imidazole phosphate with a histidine catalyst

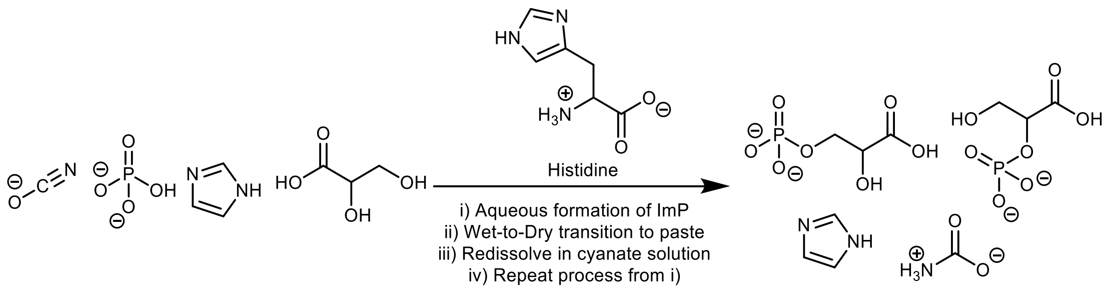

The experiment was carried out according to the procedure in S4.11. The experiment was repeated in duplicate. Supporting Figures 215 and 217 depict representative ^{31}P NMR spectra after each wet-dry cycle. The changes in yield after each wet-dry cycle for all phosphate containing species are shown in Supporting Figure 216a and 218a. The change in yield of glycerate-2-phosphate and glycerate-3-phosphate after each wet-dry cycle are shown in Supporting Figure 216b and 218b.

S4.12.1 Wet/Dry cycle for the phosphorylation of glycerate catalysed by 100 mM histidine catalyst

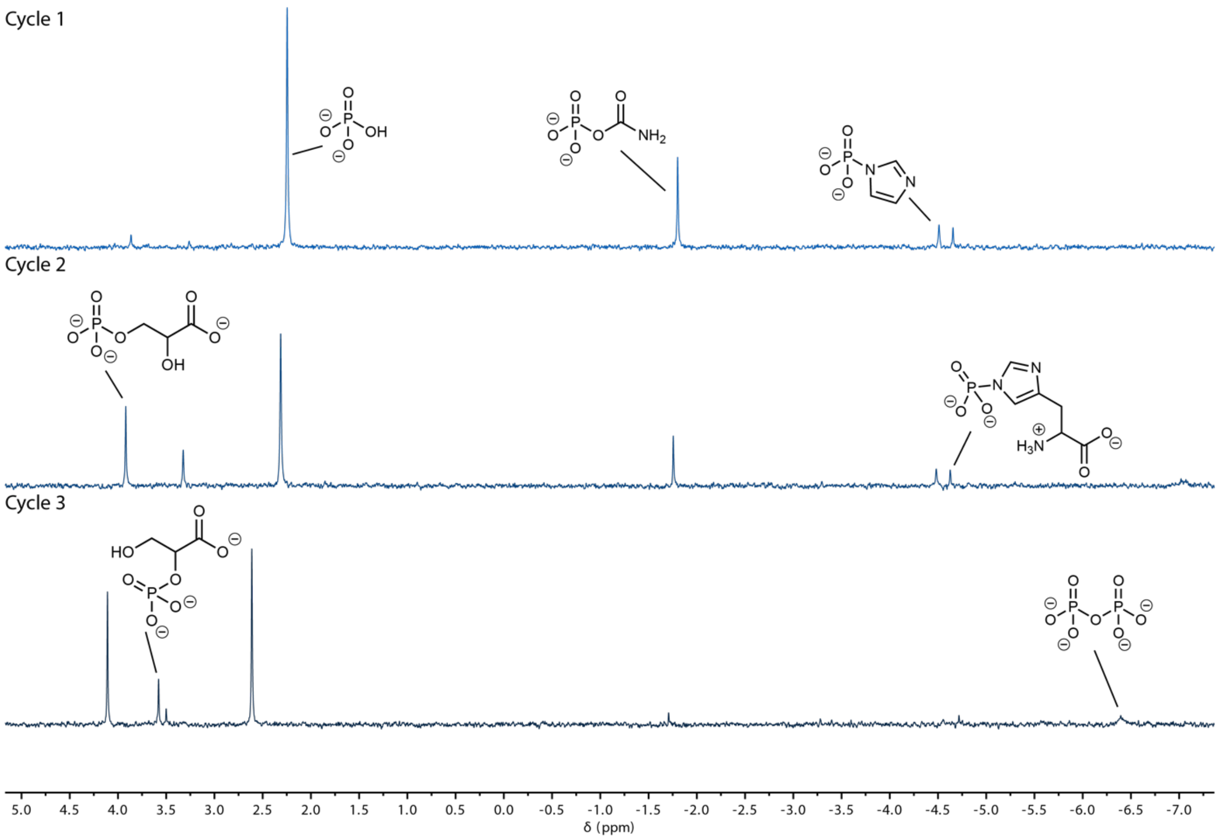

Supporting Figure 215: Representative ^{31}P-NMR spectra at the end of each cycle for the reaction of 20 mM sodium phosphate dibasic + 500 mM glycerate + 230 mM potassium cyanate + 100 mM imidazole + 100 mM histidine at pH 7.3 and 22 °C.

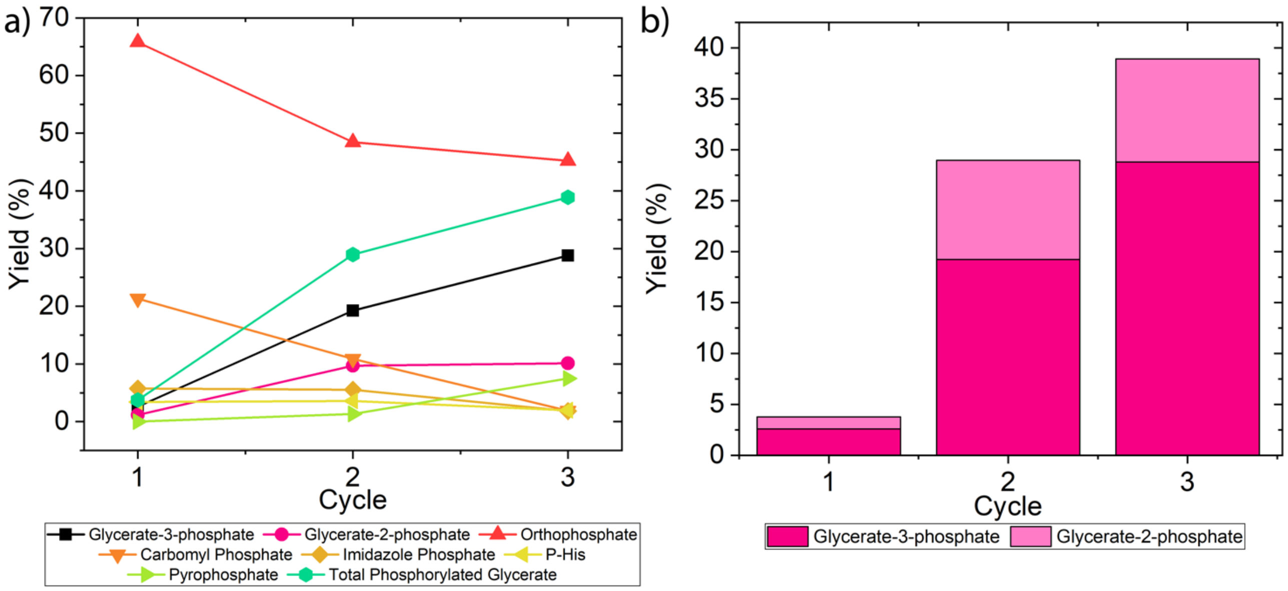

Supporting Figure 216: a) Changes in yield for all phosphate-containing products over three cycles for the reaction of for the reaction of 20 mM sodium phosphate dibasic + 500 mM glycerate + 230 mM potassium cyanate + 100 mM imidazole + 100 mM histidine at pH 7.3 and 22 °C. b) Change in yield for glycerate-2-phosphate and glycerate-3-phosphate over the course of three cycles.

S4.12.2 *Wet/Dry cycle for the phosphorylation of glycerate catalysed by 100 mM histidine catalyst 1st repeat*

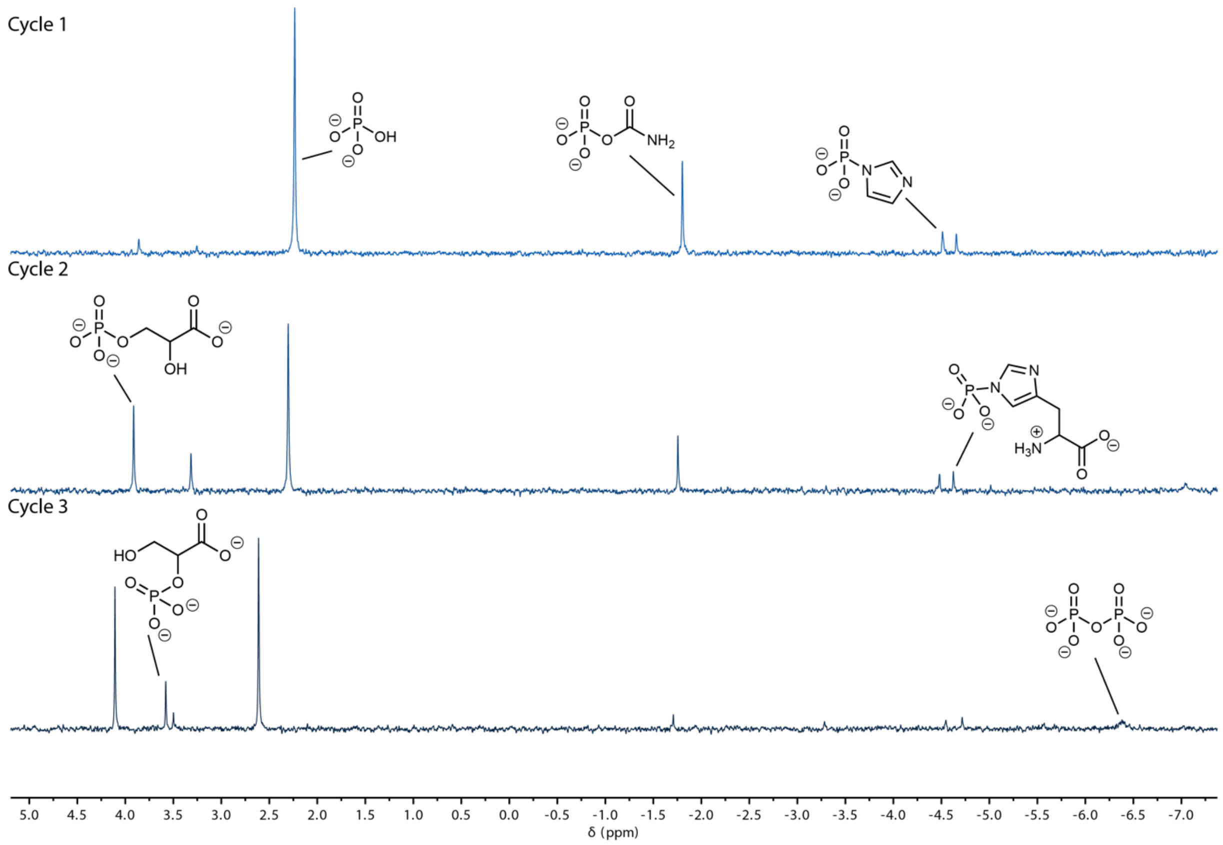

Supporting Figure 217: Representative ^{31}P-NMR spectra at the end of each cycle for the reaction of 20 mM sodium phosphate dibasic + 500 mM glycerate + 230 mM potassium cyanate + 100 mM imidazole + 100 mM histidine at pH 7.3 and 22 °C.

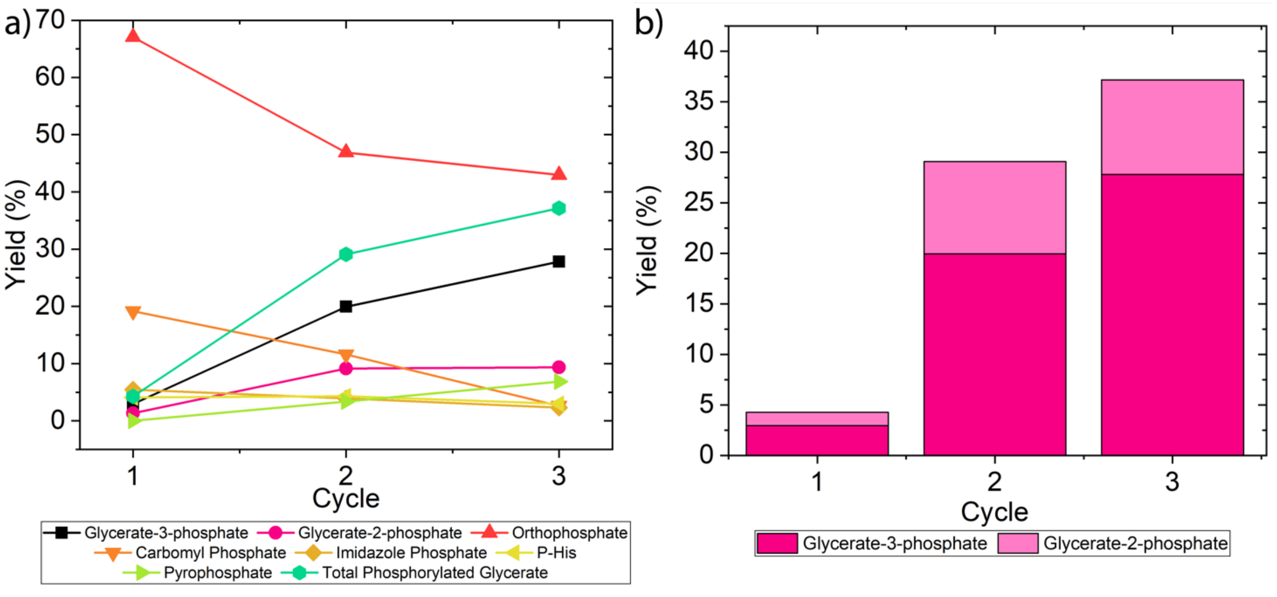

Supporting Figure 218: a) Changes in yield for all phosphate-containing products over three cycles for the reaction of 20 mM sodium phosphate dibasic + 500 mM glycerate + 230 mM potassium cyanate + 100 mM imidazole + 100 mM histidine at pH 7.3 and 22 °C. b) Change in yield for glycerate-2-phosphate and glycerate-3-phosphate over the course of three cycles.

S4.13 Wet/Dry Cycle for the phosphorylation of glycerate by imidazole phosphate with a His-Lys catalyst

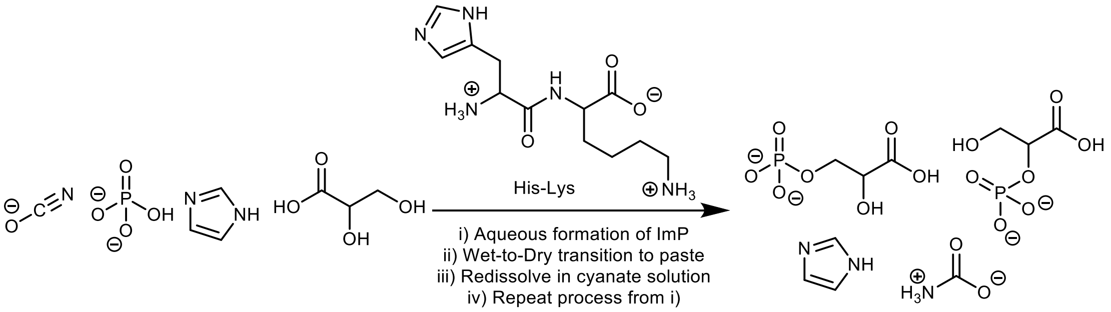

The experiment was carried out according to the procedure in S4.11 but with the 100 mM of histidine replaced by 100 mM His-Lys.HBr (145.7 mg, 0.4 mmol). The experiment was repeated in duplicate. Supporting Figures 219 and 221 depict representative ^{31}P NMR spectra after each wet-dry cycle. The changes in yield after each wet-dry cycle for all phosphate containing species are shown in Supporting Figure 220a and 222a. The change in yield of glycerate-2-phosphate and glycerate-3-phosphate after each wet-dry cycle are shown in Supporting Figure 220b and 222b.

S4.13.1 Wet/Dry cycle for the phosphorylation of glycerate catalysed by 100 mM His-Lys catalyst

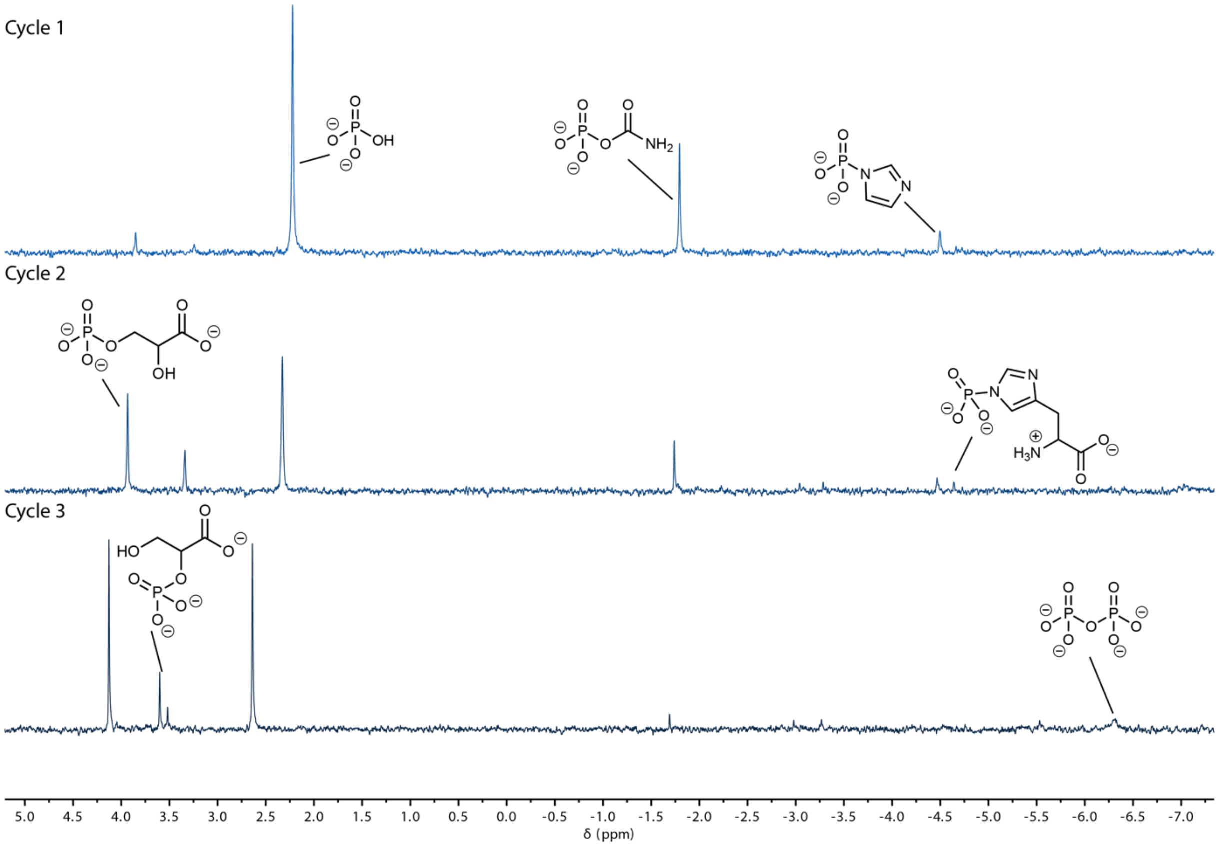

Supporting Figure 219: Representative ^{31}P-NMR spectra at the end of each cycle for the reaction of 20 mM sodium phosphate dibasic + 500 mM glycerate + 230 mM potassium cyanate + 100 mM imidazole + 100 mM His-Lys at pH 7.3 and 22 °C.

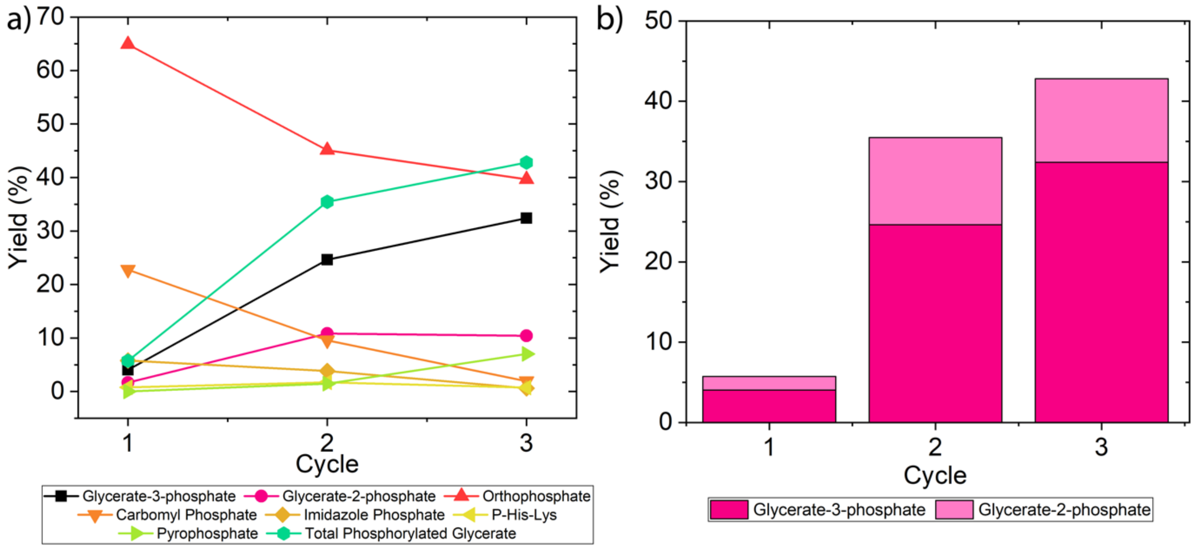

Supporting Figure 220: a) Changes in yield for all phosphate-containing products over three cycles for the reaction of 20 mM sodium phosphate dibasic + 500 mM glycerate + 230 mM potassium cyanate + 100 mM imidazole + 100 mM His-Lys at pH 7.3 and 22 °C. b) Change in yield for glycerate-2-phosphate and glycerate-3-phosphate over the course of three cycles.

S4.13.2 Wet/Dry cycle for the phosphorylation of glycerate catalysed by 100 mM His-Lys catalyst 1st repeat

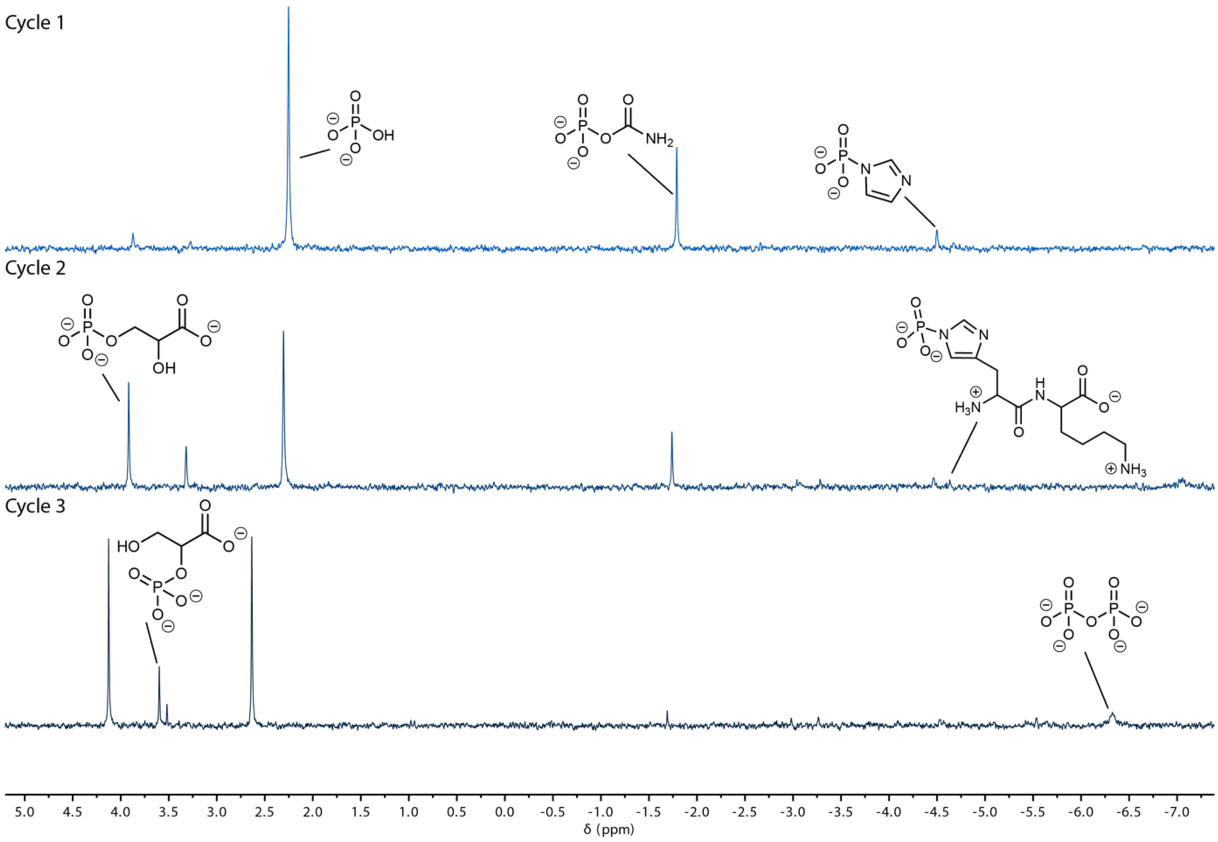

Supporting Figure 221: Representative ^{31}P-NMR spectra at the end of each cycle for the reaction of 20 mM sodium phosphate dibasic + 500 mM glycerate + 230 mM potassium cyanate + 100 mM imidazole + 100 mM His-Lys at pH 7.3 and 22 °C.

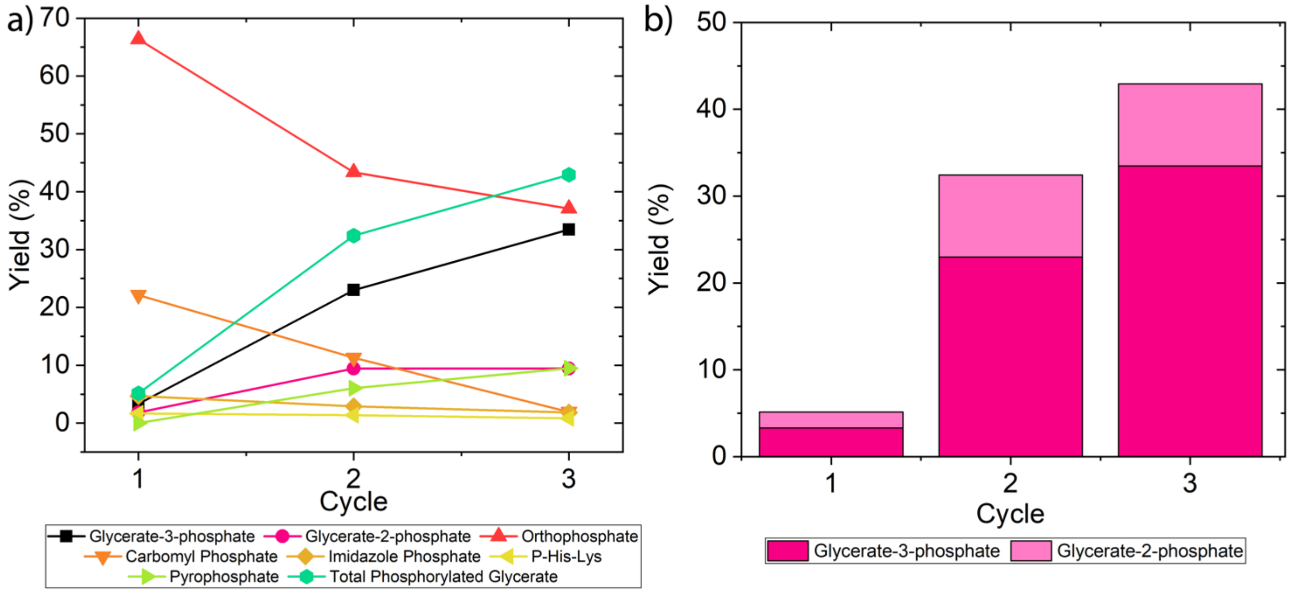

Supporting Figure 222: a) Changes in yield for all phosphate-containing products over three cycles for the reaction of 20 mM sodium phosphate dibasic + 500 mM glycerate + 230 mM potassium cyanate + 100 mM imidazole + 100 mM His-Lys at pH 7.3 and 22 °C. b) Change in yield for glycerate-2-phosphate and glycerate-3-phosphate over the course of three cycles.

S4.14 Wet/Dry Cycle with phosphorylation of glycerate by imidazole phosphate – uncatalysed reaction

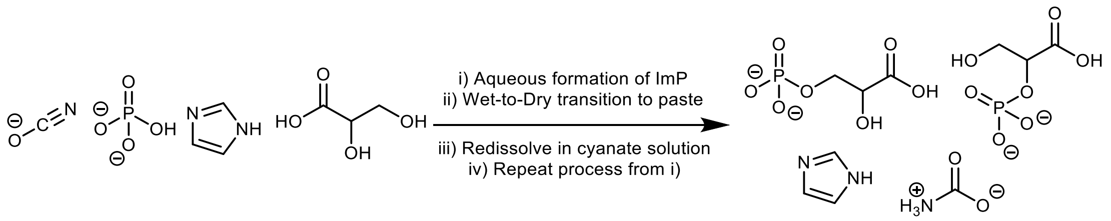

The experiment was carried out according to the procedure in S4.11 but with no 100 mM of histidine. The experiment was repeated in duplicate. Supporting Figures 223 and 225 depict representative ^{31}P NMR spectra after each wet-dry cycle. The changes in yield after each wet-dry cycle for all phosphate containing species are shown in Supporting Figure 224a and 226a. The change in yield of glycerate-2-phosphate and glycerate-3-phosphate after each wet-dry cycle are shown in Supporting Figure 224b and 226b.

S4.14.1 Wet/Dry cycle for the phosphorylation of glycerate uncatalysed reaction

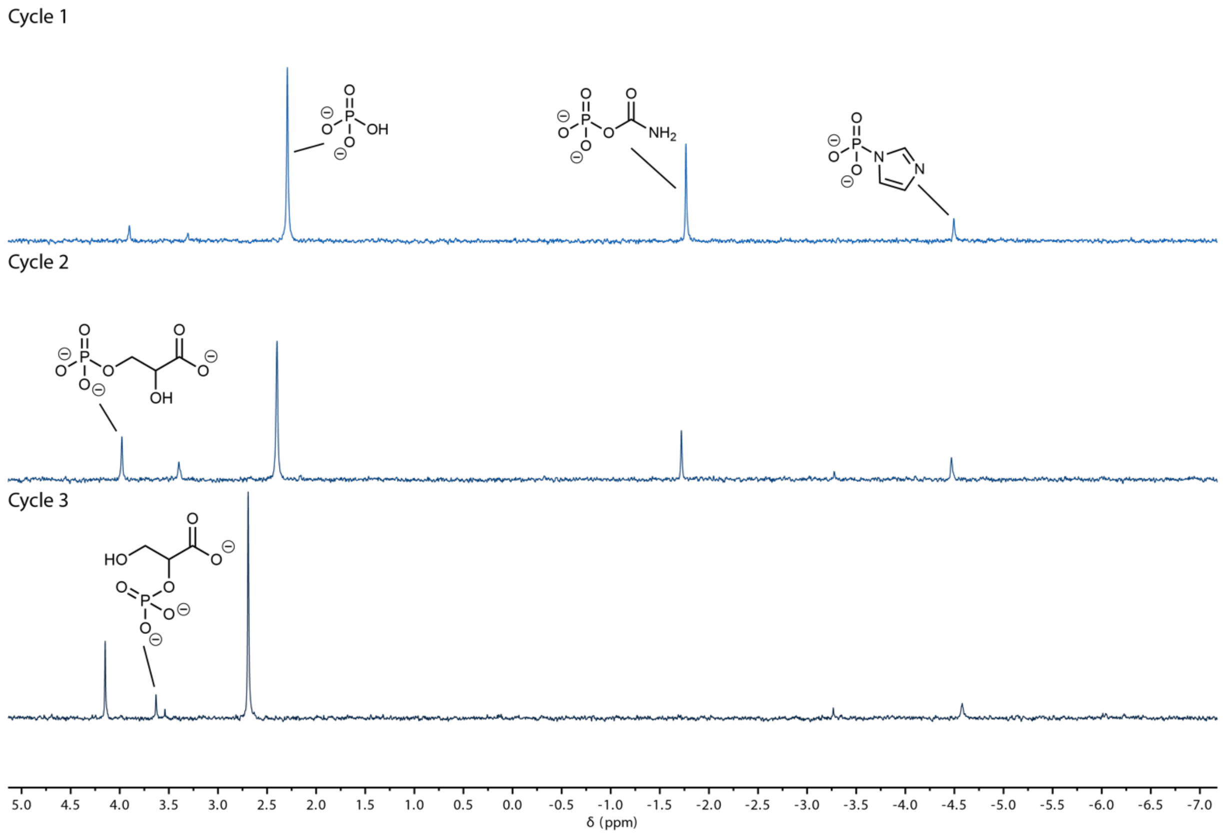

Supporting Figure 223: Representative ^{31}P-NMR spectra at the end of each cycle for the reaction of 20 mM sodium phosphate dibasic + 500 mM glycerate + 230 mM potassium cyanate + 100 mM imidazole at pH 7.3 and 22 °C.

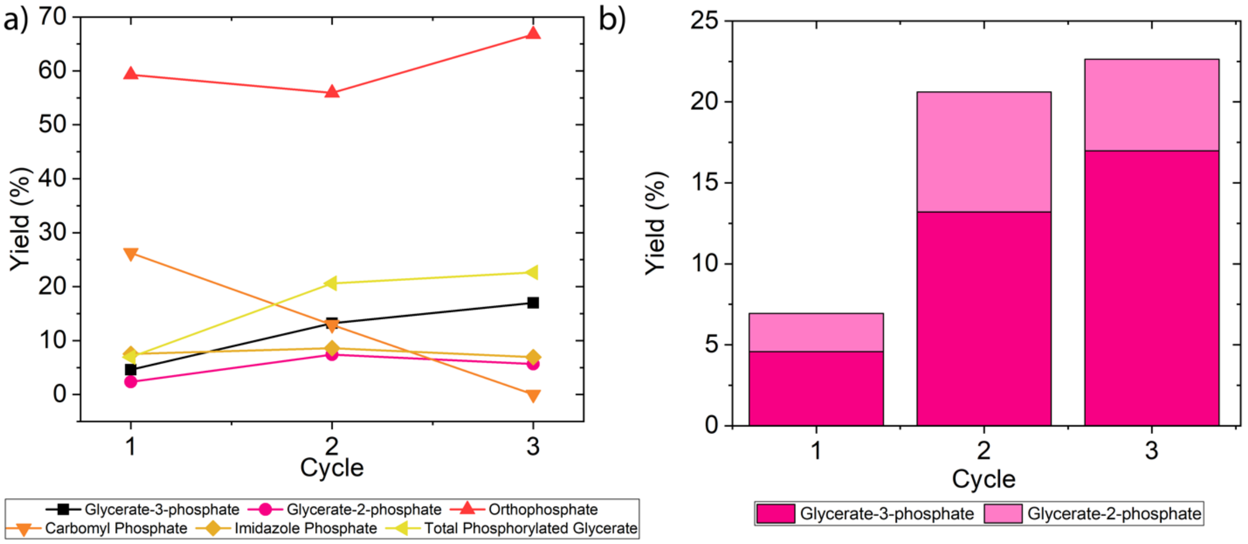

Supporting Figure 224: a) Changes in yield for all phosphate-containing products over three cycles for the reaction of 20 mM sodium phosphate dibasic + 500 mM glycerate + 230 mM potassium cyanate + 100 mM imidazole at pH 7.3 and 22 °C. b) Change in yield for glycerate-2-phosphate and glycerate-3-phosphate over the course of three cycles.

S4.14.2 Wet/Dry cycle for the phosphorylation of glycerol uncatalysed reaction 1st repeat

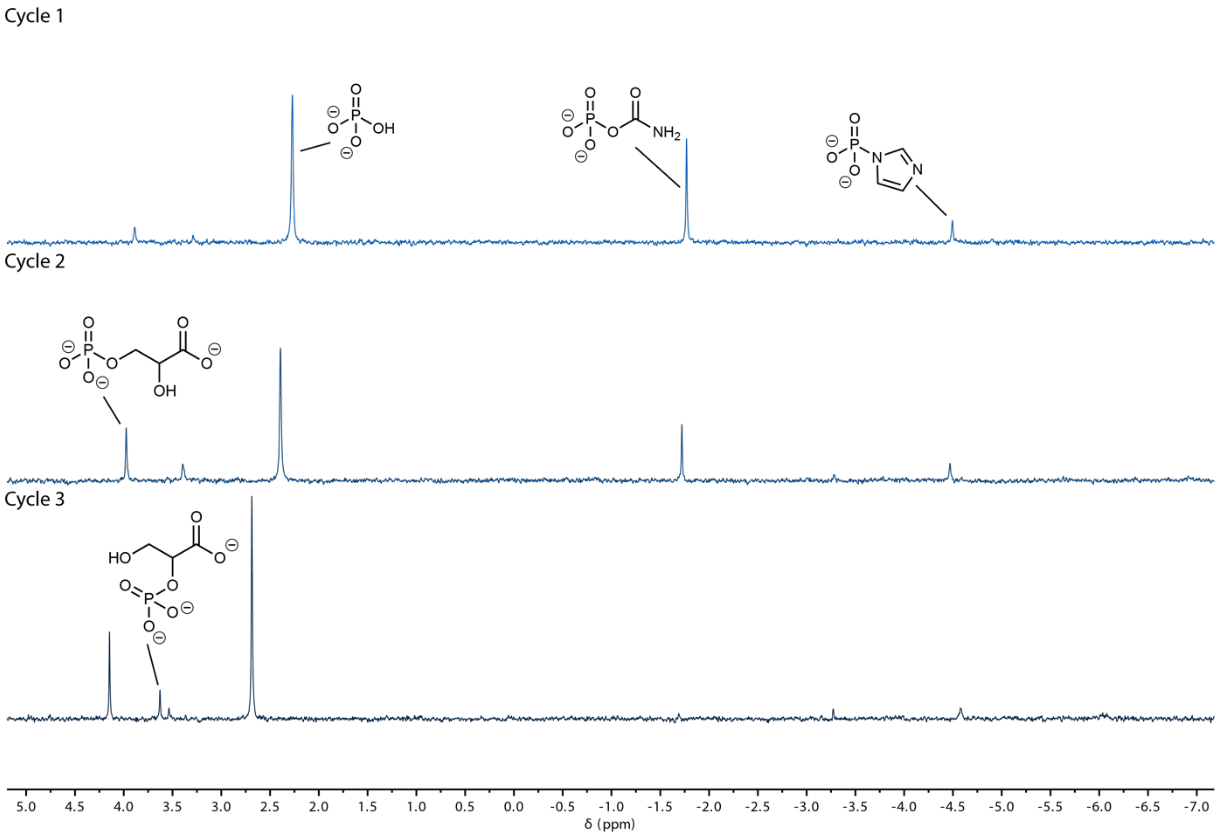

Supporting Figure 225: Representative ^{31}P-NMR spectra at the end of each cycle for the reaction of 20 mM sodium phosphate dibasic + 500 mM glycerate + 230 mM potassium cyanate + 100 mM imidazole at pH 7.3 and 22 °C.

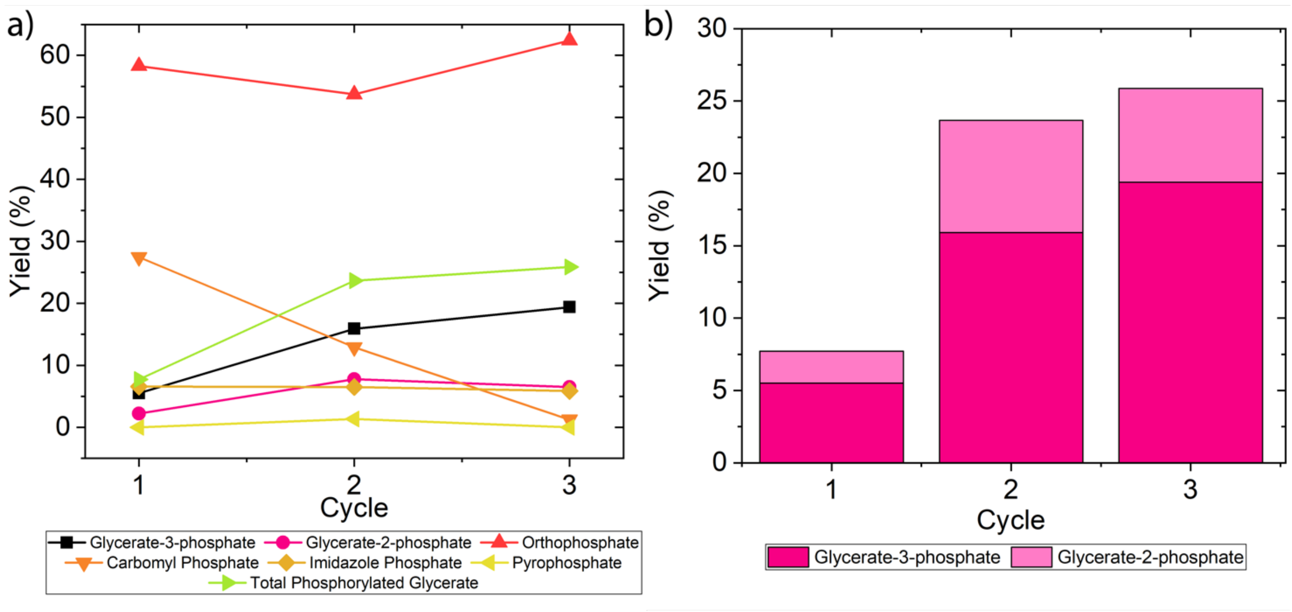

Supporting Figure 226: a) Changes in yield for all phosphate-containing products over three cycles for the reaction of 20 mM sodium phosphate dibasic + 500 mM glycerate + 230 mM potassium cyanate + 100 mM imidazole at pH 7.3 and 22 °C. b) Change in yield for glycerate-2-phosphate and glycerate-3-phosphate over the course of three cycles.

S4.15 Wet/Dry Cycle for the phosphorylation of glycerol by imidazole phosphate with 50 mM histidine catalyst at 4 °C

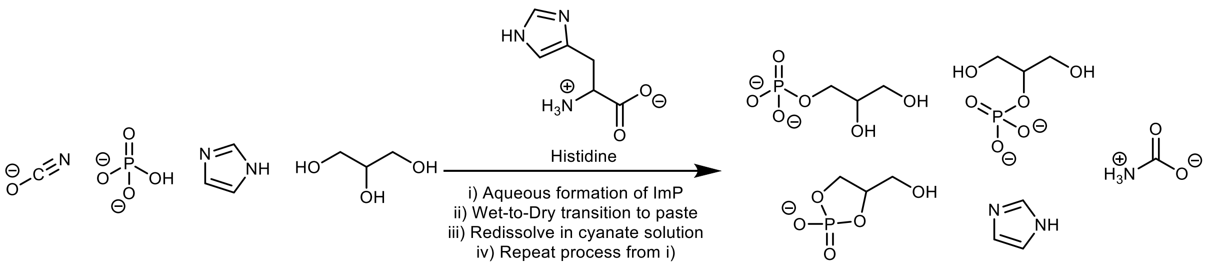

The experiment was carried out according to the procedure in S4.1 but with the 100 mM of histidine replaced by 50 mM histidine (31.0 mg, 0.2 mmol) and after drying the solution into a paste for 24 h the paste was transferred to a fridge and kept at 4 °C for 24 h. The experiment was repeated in duplicate. Supporting Figures 227 depict representative ^{31}P NMR spectra after each wet-dry cycle. The changes in yield after each wet-dry cycle for all phosphate containing species are shown in Supporting Figure 228a. The change in yield of glycerol-1-phosphate and glycerol-2-phosphate after each wet-dry cycle are shown in Supporting Figure 228b.

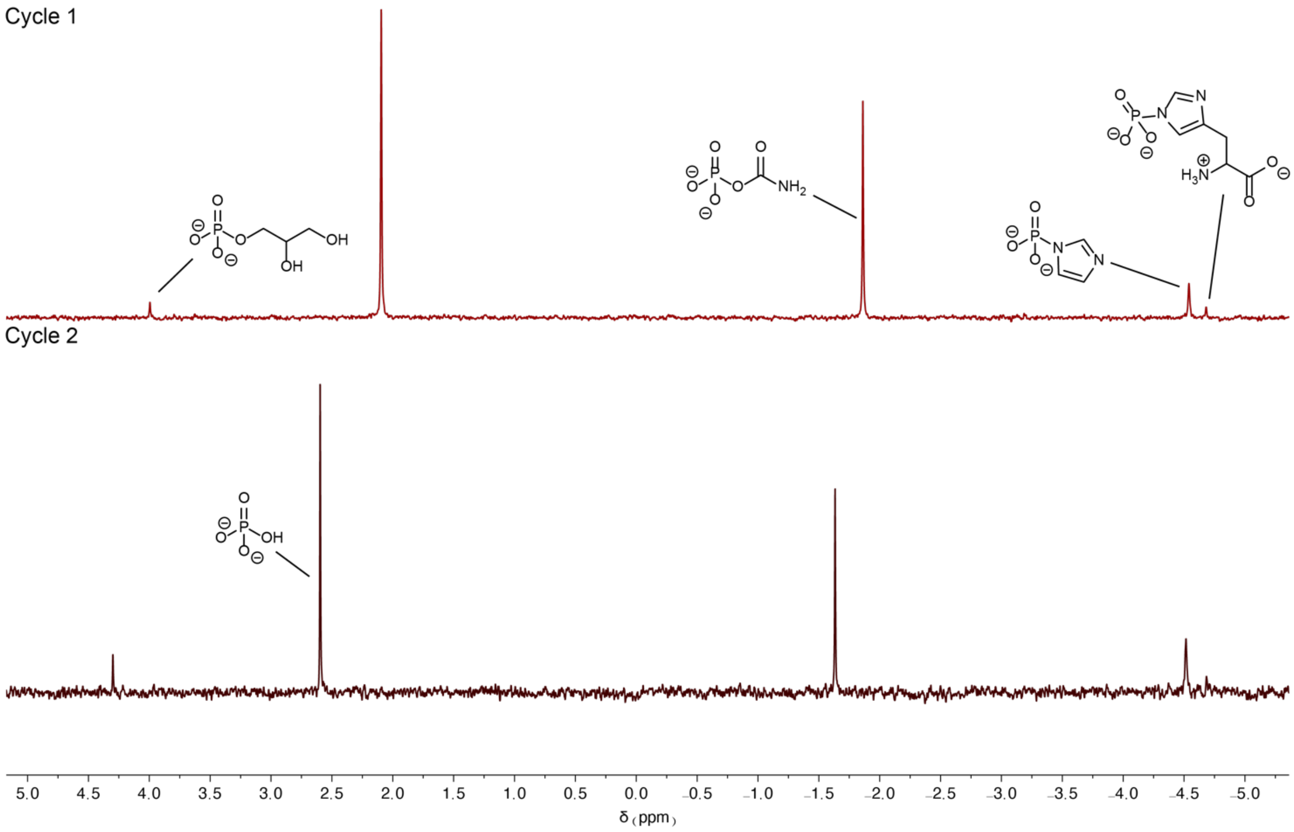

Supporting Figure 227: Representative ^{31}P-NMR spectra at the end of each cycle for the reaction of 20 mM sodium phosphate dibasic + 500 mM glycerol + 230 mM potassium cyanate + 100 mM imidazole + 50 mM histidine at pH 7.3 and phosphorylation at 4 °C.

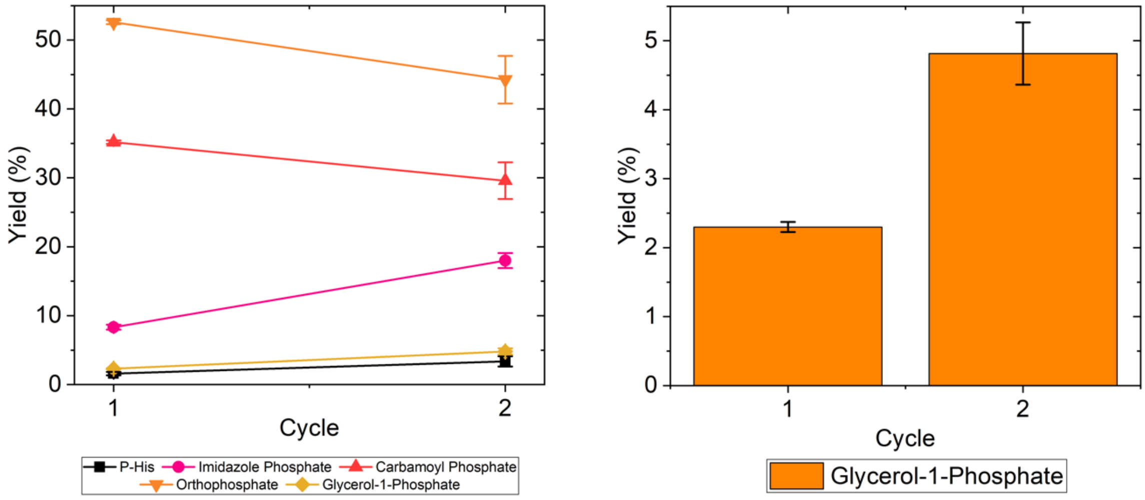

Supporting Figure 228: a) Changes in yield for all phosphate-containing products over two cycles for the reaction of 20 mM sodium phosphate dibasic + 500 mM glycerol + 230 mM potassium cyanate + 100 mM imidazole + 50 mM histidine at pH 7.3 and phosphorylation at 4 °C. b) Change in yield for glycerol-1-phosphate and glycerol-2-phosphate over the course of two cycles.

S4.16 Wet/Dry Cycle for the phosphorylation of glycerol by imidazole phosphate at 4 °C

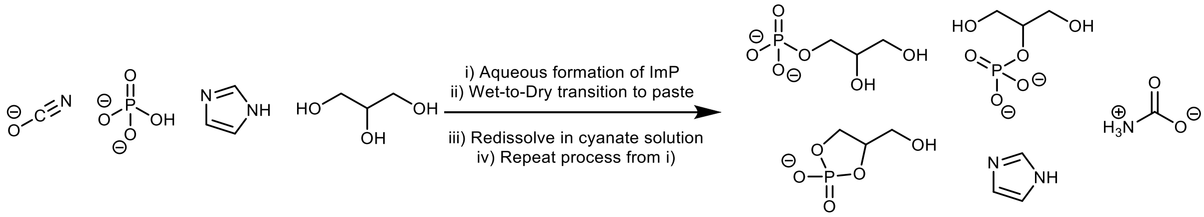

The experiment was carried out according to the procedure in S4.1 but without 100 mM of histidine and after drying the solution into a paste for 24 h the paste was transferred to a fridge and kept at 4 °C for 24 h. The experiment was repeated in duplicate. Supporting Figures 229 depict representative ^{31}P NMR spectra after each wet-dry cycle. The changes in yield after each wet-dry cycle for all phosphate containing species are shown in Supporting Figure 230a. The change in yield of glycerol-1-phosphate and glycerol-2-phosphate after each wet-dry cycle are shown in Supporting Figure 230b.

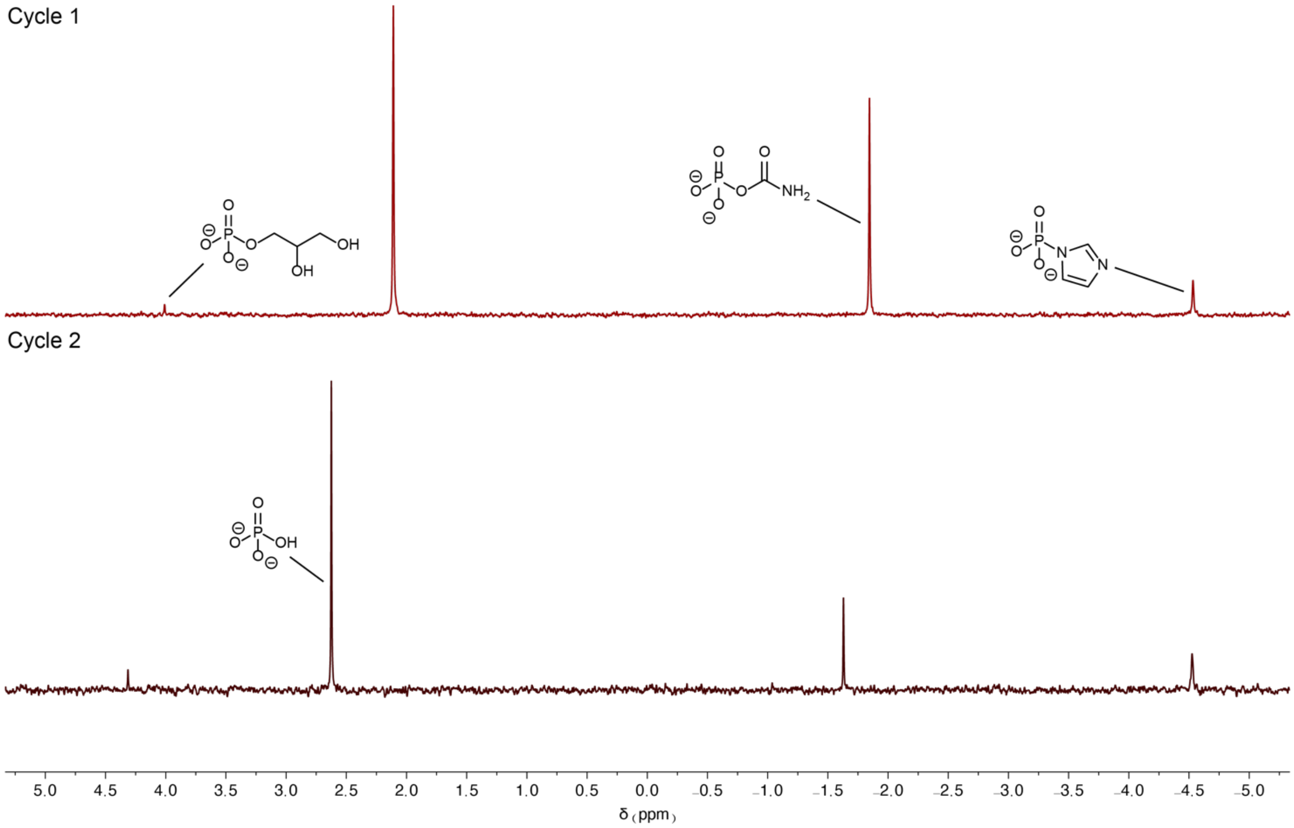

Supporting Figure 229: Representative ^{31}P-NMR spectra at the end of each cycle for the reaction of 20 mM sodium phosphate dibasic + 500 mM glycerol + 230 mM potassium cyanate + 100 mM imidazole at pH 7.3 and phosphorylation at 4 °C.

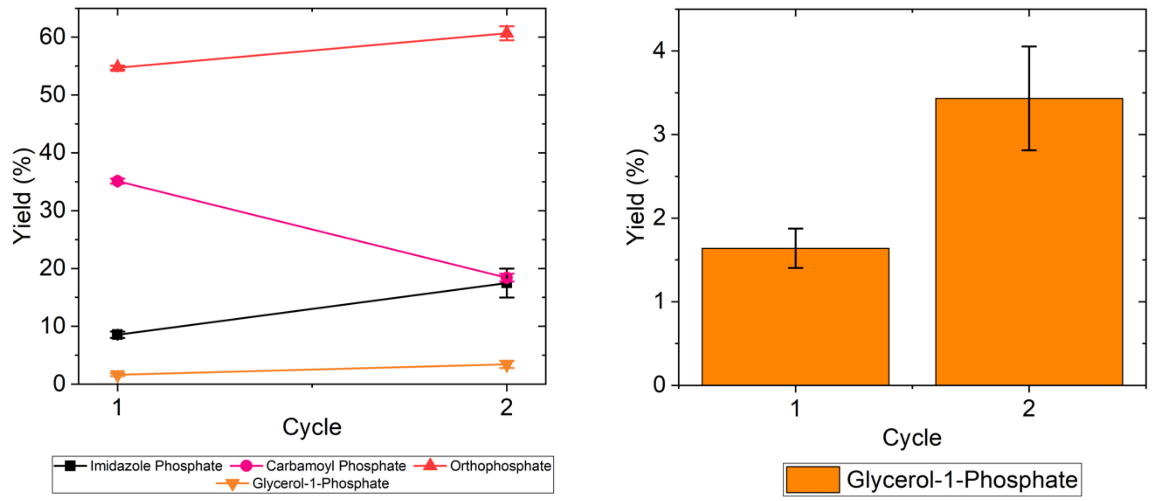

Supporting Figure 230: a) Changes in yield for all phosphate-containing products over two cycles for the reaction of 20 mM sodium phosphate dibasic + 500 mM glycerol + 230 mM potassium cyanate + 100 mM imidazole at pH 7.3 and phosphorylation at 4 °C. b) Change in yield for glycerol-1-phosphate and glycerol-2-phosphate over the course of two cycles.

S4.17 Wet/Dry Cycle for the phosphorylation of glycerol by imidazole phosphate with 50 mM histidine catalyst at 35 °C

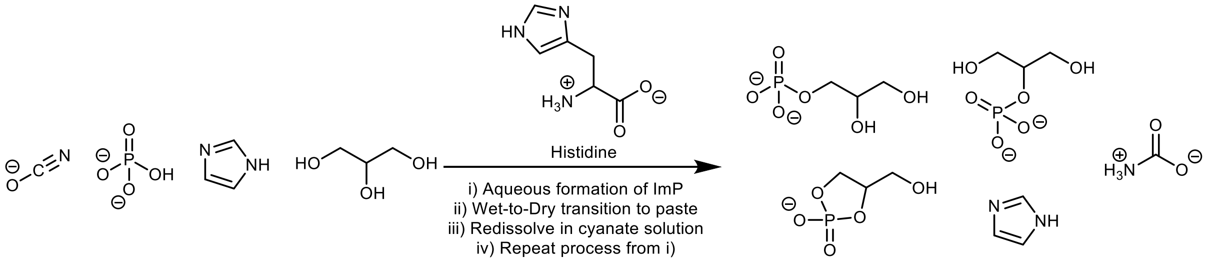

The experiment was carried out according to the procedure in S4.1 but with the 100 mM of histidine replaced by 50 mM histidine (31.0 mg, 0.2 mmol) and after drying the solution into a paste for 24 h the paste was transferred to an oven and kept at 35 °C for 24 h. The experiment was repeated in duplicate. Supporting Figure 231 depict representative ^{31}P NMR spectra after each wet-dry cycle. The changes in yield after each wet-dry cycle for all phosphate containing species are shown in Supporting Figure 232a. The change in yield of glycerol-1-phosphate and glycerol-2-phosphate after each wet-dry cycle are shown in Supporting Figure 232b.

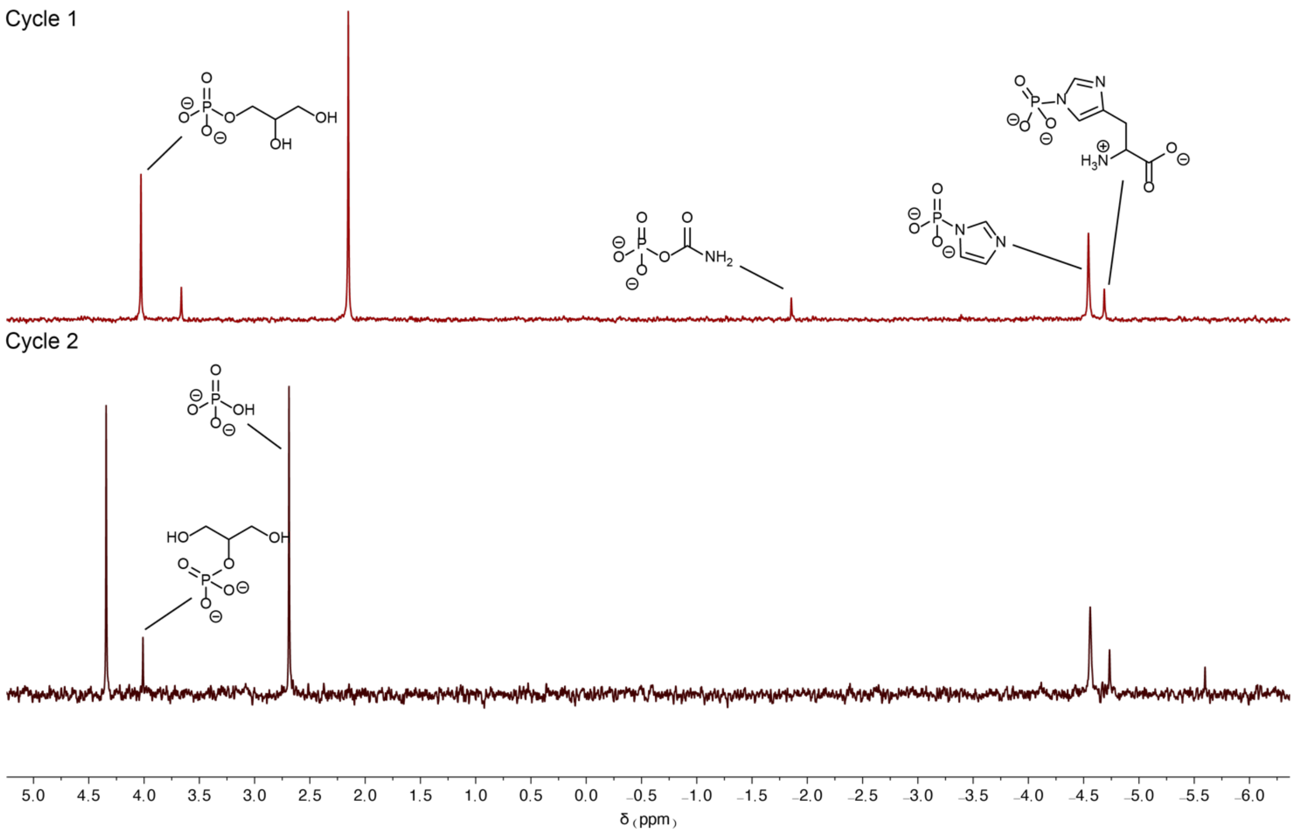

Supporting Figure 231: Representative ^{31}P-NMR spectra at the end of each cycle for the reaction of 20 mM sodium phosphate dibasic + 500 mM glycerol + 230 mM potassium cyanate + 100 mM imidazole + 50 mM histidine at pH 7.3 and phosphorylation at 35 °C.

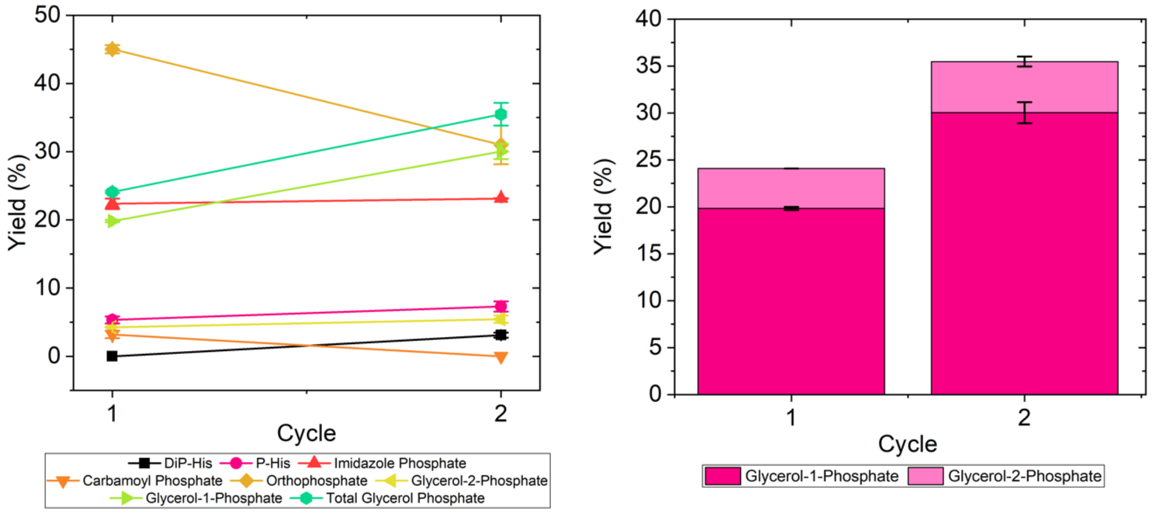

Supporting Figure 232: a) Changes in yield for all phosphate-containing products over two cycles for the reaction of 20 mM sodium phosphate dibasic + 500 mM glycerol + 230 mM potassium cyanate + 100 mM imidazole + 50 mM histidine at pH 7.3 and phosphorylation at 35 °C. b) Change in yield for glycerol-1-phosphate and glycerol-2-phosphate over the course of two cycles.

S4.18 Wet/Dry Cycle for the phosphorylation of glycerol by imidazole phosphate at 35 °C

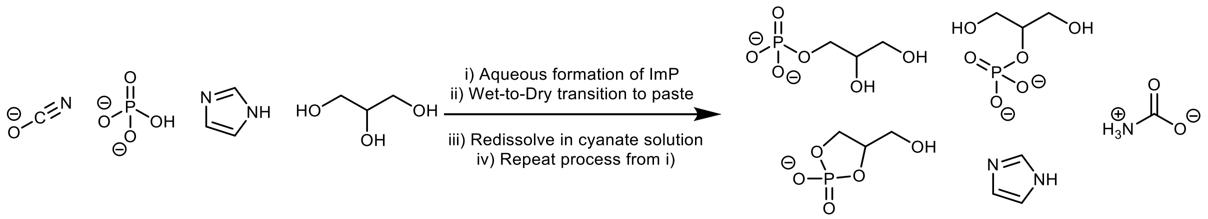

The experiment was carried out according to the procedure in S4.1 but without 100 mM of histidine and after drying the solution into a paste for 24 h the paste was transferred to an oven and kept at 35 °C for 24 h. The experiment was repeated in duplicate. Supporting Figure 233 depict representative ^{31}P NMR spectra after each wet-dry cycle. The changes in yield after each wet-dry cycle for all phosphate containing species are shown in Supporting Figure 234a. The change in yield of glycerol-1-phosphate and glycerol-2-phosphate after each wet-dry cycle are shown in Supporting Figure 234b.

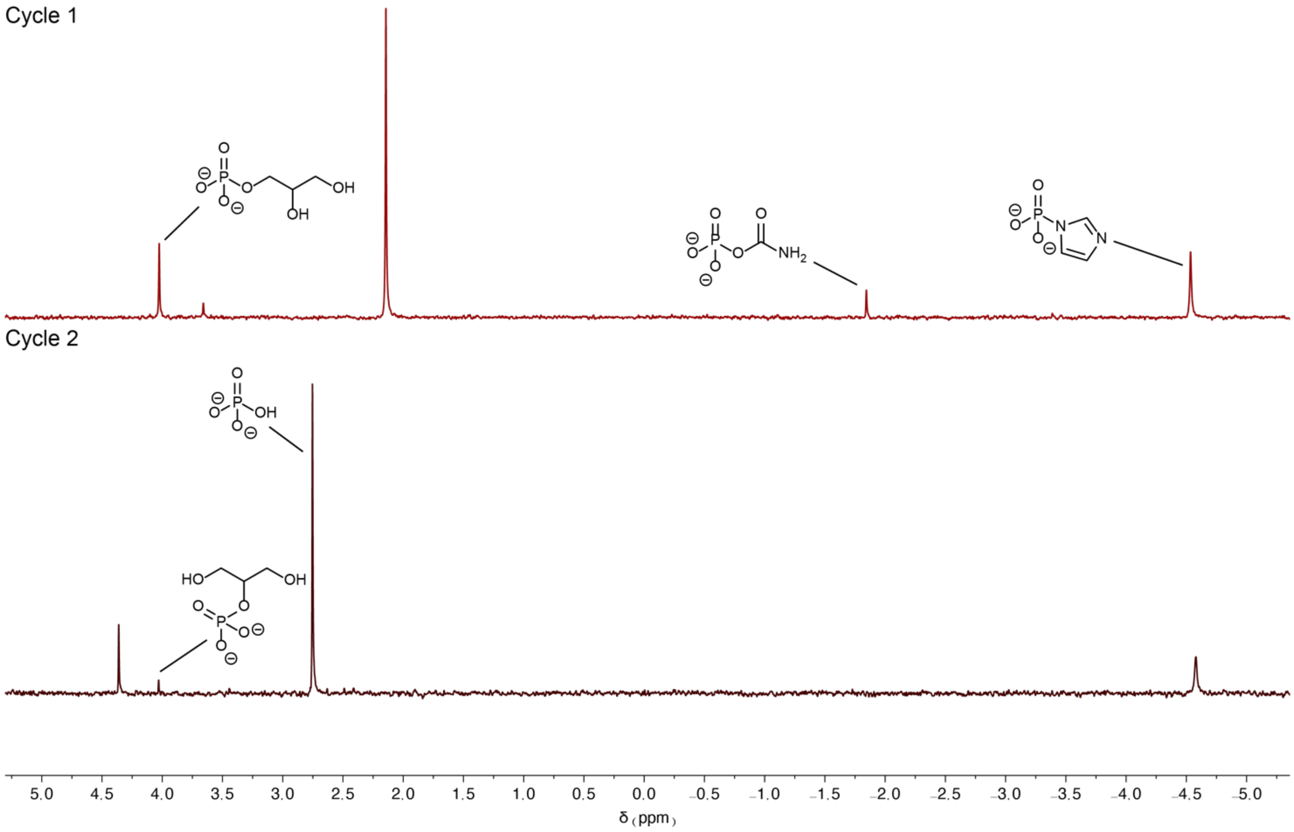

Supporting Figure 233: Representative ^{31}P-NMR spectra at the end of each cycle for the reaction of 20 mM sodium phosphate dibasic + 500 mM glycerol + 230 mM potassium cyanate + 100 mM imidazole at pH 7.3 and phosphorylation at 35 °C.

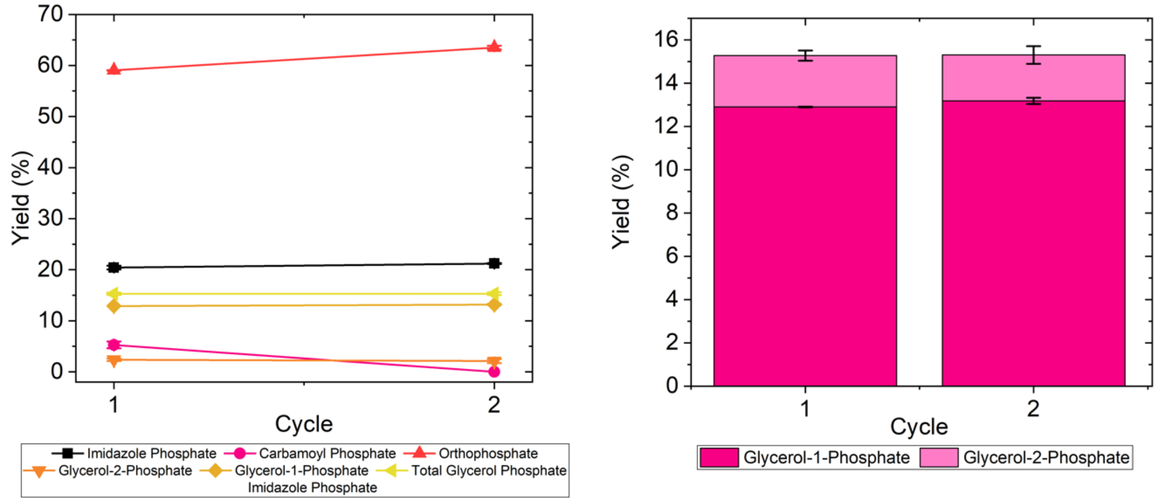

Supporting Figure 234: a) Changes in yield for all phosphate-containing products over two cycles for the reaction of 20 mM sodium phosphate dibasic + 500 mM glycerol + 230 mM potassium cyanate + 100 mM imidazole at pH 7.3 and phosphorylation at 35 °C. b) Change in yield for glycerol-1-phosphate and glycerol-2-phosphate over the course of two cycles.

S4.19 Wet/Dry Cycle for the phosphorylation of glycerol by imidazole phosphate with 50 mM histidine catalyst at 50 °C

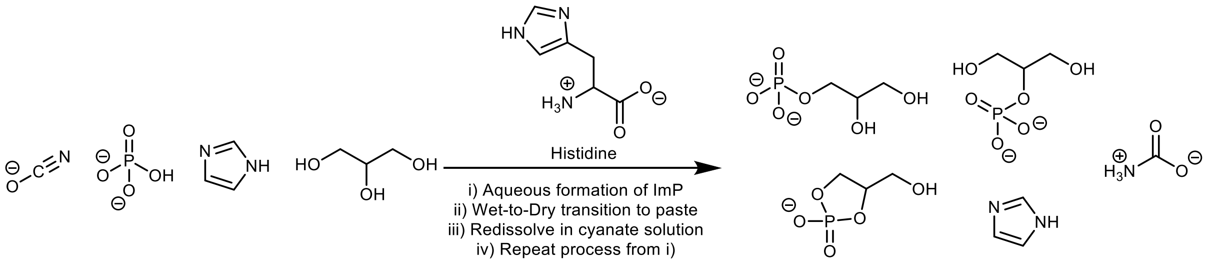

The experiment was carried out according to the procedure in S4.1 but with the 100 mM of histidine replaced by 50 mM histidine (31.0 mg, 0.2 mmol) and after drying the solution into a paste for 24 h the paste was transferred to an oven and kept at 50 °C for 24 h. The experiment was repeated in duplicate. Supporting Figure 235 depict representative ^{31}P NMR spectra after each wet-dry cycle. The changes in yield after each wet-dry cycle for all phosphate containing species are shown in Supporting Figure 236a. The change in yield of glycerol-1-phosphate and glycerol-2-phosphate after each wet-dry cycle are shown in Supporting Figure 236b.

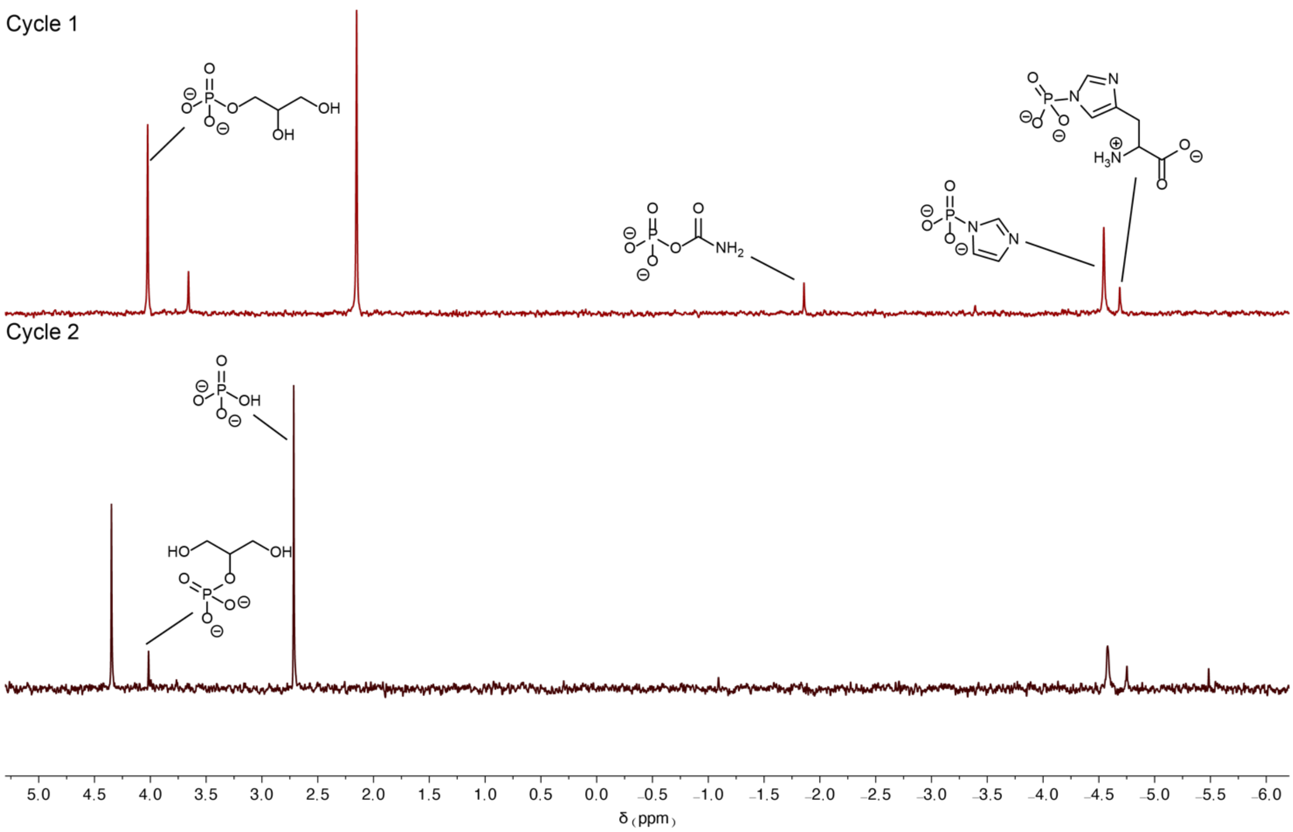

Supporting Figure 235: Representative ^{31}P-NMR spectra at the end of each cycle for the reaction of 20 mM sodium phosphate dibasic + 500 mM glycerol + 230 mM potassium cyanate + 100 mM imidazole + 50 mM histidine at pH 7.3 and phosphorylation at 50 °C.

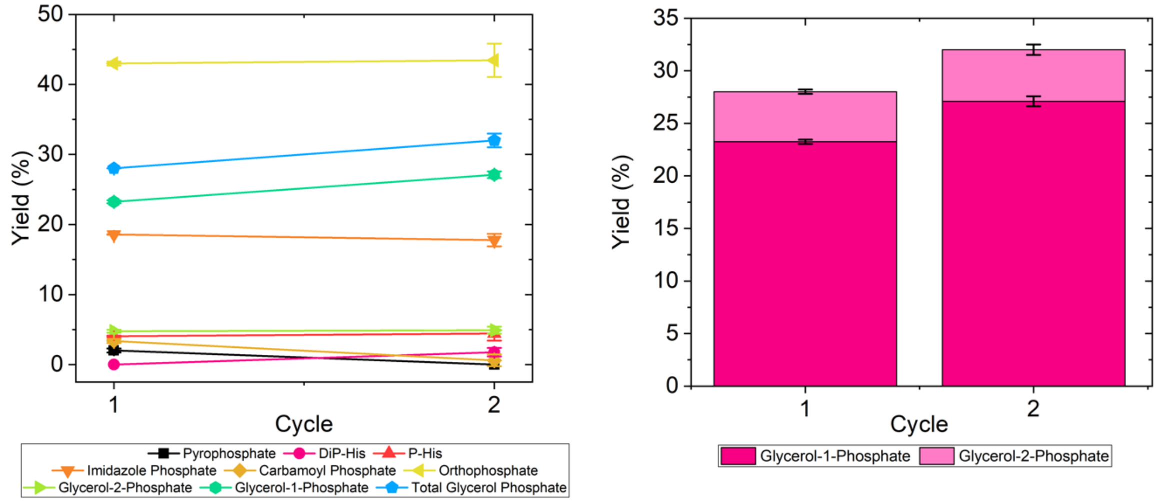

Supporting Figure 236: a) Changes in yield for all phosphate-containing products over two cycles for the reaction of 20 mM sodium phosphate dibasic + 500 mM glycerol + 230 mM potassium cyanate + 100 mM imidazole + 50 mM histidine at pH 7.3 and phosphorylation at 50 °C. b) Change in yield for glycerol-1-phosphate and glycerol-2-phosphate over the course of two cycles.

S4.20 Wet/Dry Cycle for the phosphorylation of glycerol by imidazole phosphate at 50 °C

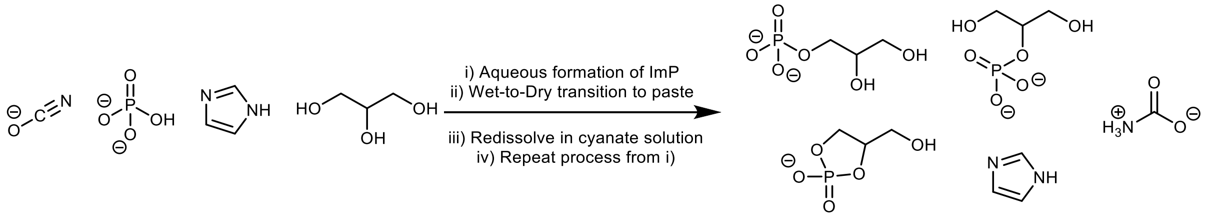

The experiment was carried out according to the procedure in S4.1 but without 100 mM of histidine and after drying the solution into a paste for 24 h the paste was transferred to an oven and kept at 50 °C for 24 h. The experiment was repeated in duplicate. Supporting Figure 237 depict representative ^{31}P NMR spectra after each wet-dry cycle. The changes in yield after each wet-dry cycle for all phosphate containing species are shown in Supporting Figure 238a. The change in yield of glycerol-1-phosphate and glycerol-2-phosphate after each wet-dry cycle are shown in Supporting Figure 238b.

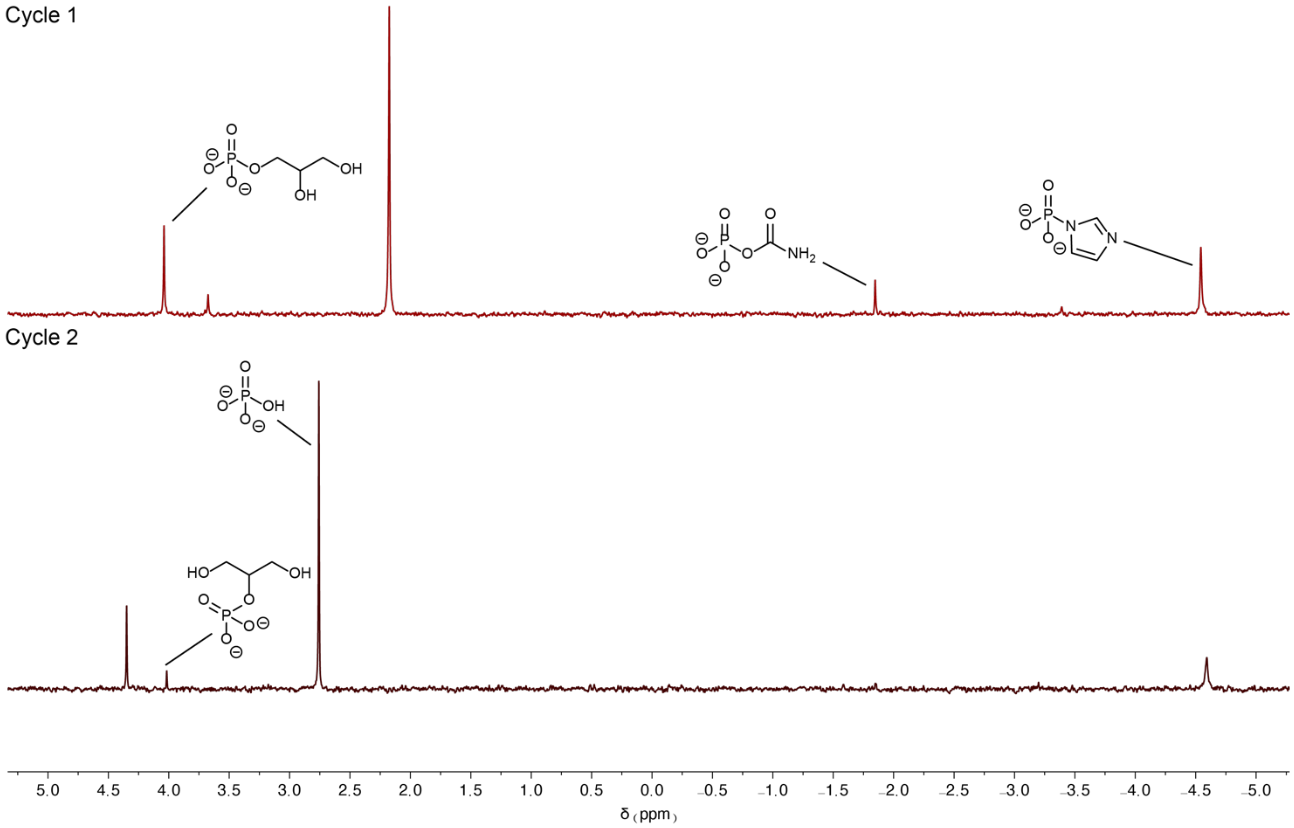

Supporting Figure 237: Representative ^{31}P-NMR spectra at the end of each cycle for the reaction of 20 mM sodium phosphate dibasic + 500 mM glycerol + 230 mM potassium cyanate + 100 mM imidazole at pH 7.3 and phosphorylation at 50 °C.

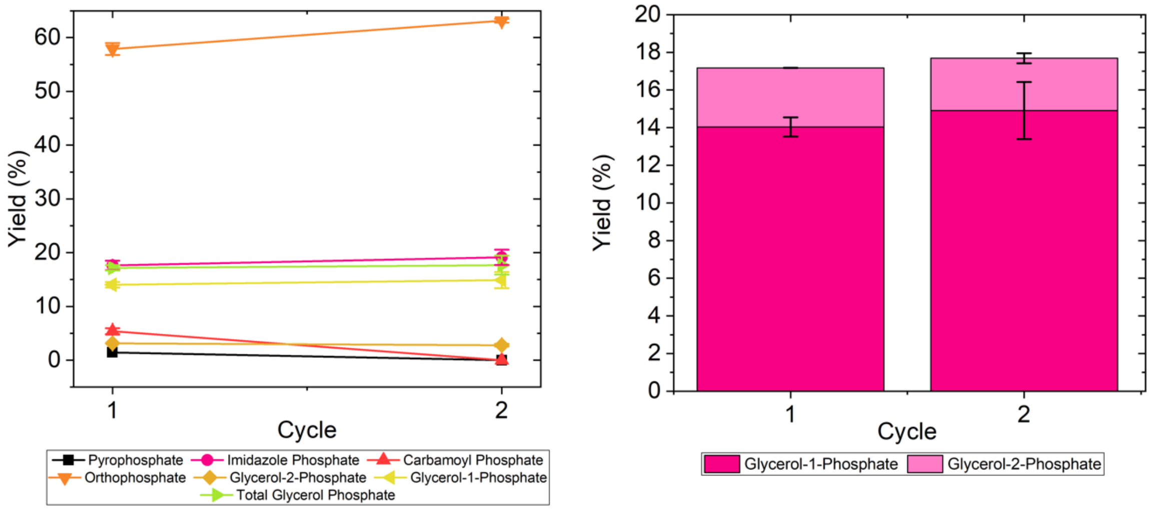

Supporting Figure 238: a) Changes in yield for all phosphate-containing products over two cycles for the reaction of 20 mM sodium phosphate dibasic + 500 mM glycerol + 230 mM potassium cyanate + 100 mM imidazole at pH 7.3 and phosphorylation at 50 °C. b) Change in yield for glycerol-1-phosphate and glycerol-2-phosphate over the course of two cycles.

S4.21 Wet/Dry Cycle for the phosphorylation of glycerol by imidazole phosphate with 50 mM histidine catalyst at pH 6.5

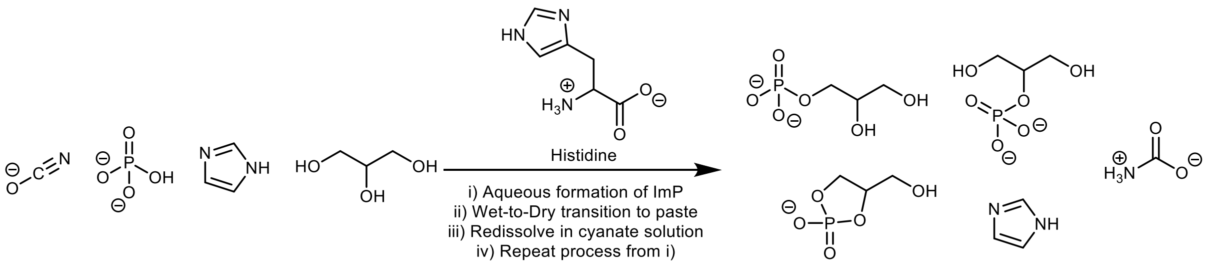

The experiment was carried out according to the procedure in S4.1 but with the 100 mM of histidine replaced by 50 mM histidine (31.0 mg, 0.2 mmol) and at pH 6.5. The pH rose to around pH 7.2 during the wet part of the cycle and was corrected back to pH 6.5 just before the drying of solution began. The experiment was repeated in duplicate. Supporting Figure 239 depict representative ^{31}P NMR spectra after each wet-dry cycle. The changes in yield after each wet-dry cycle for all phosphate containing species are shown in Supporting Figure 240a. The change in yield of glycerol-1-phosphate and glycerol-2-phosphate after each wet-dry cycle are shown in Supporting Figure 240b.

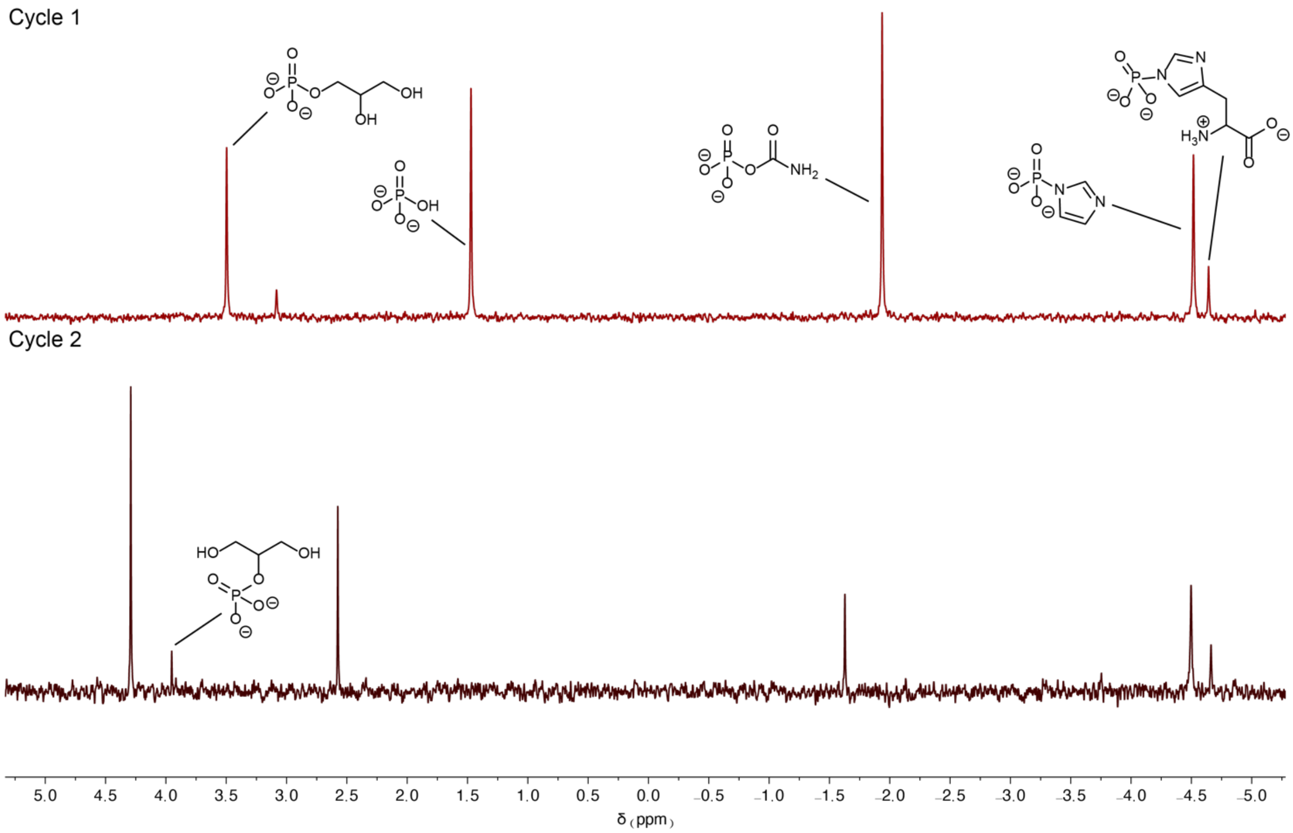

Supporting Figure 239: Representative ^{31}P-NMR spectra at the end of each cycle for the reaction of 20 mM sodium phosphate dibasic + 500 mM glycerol + 230 mM potassium cyanate + 100 mM imidazole + 50 mM histidine at pH 6.5 and 22 °C.

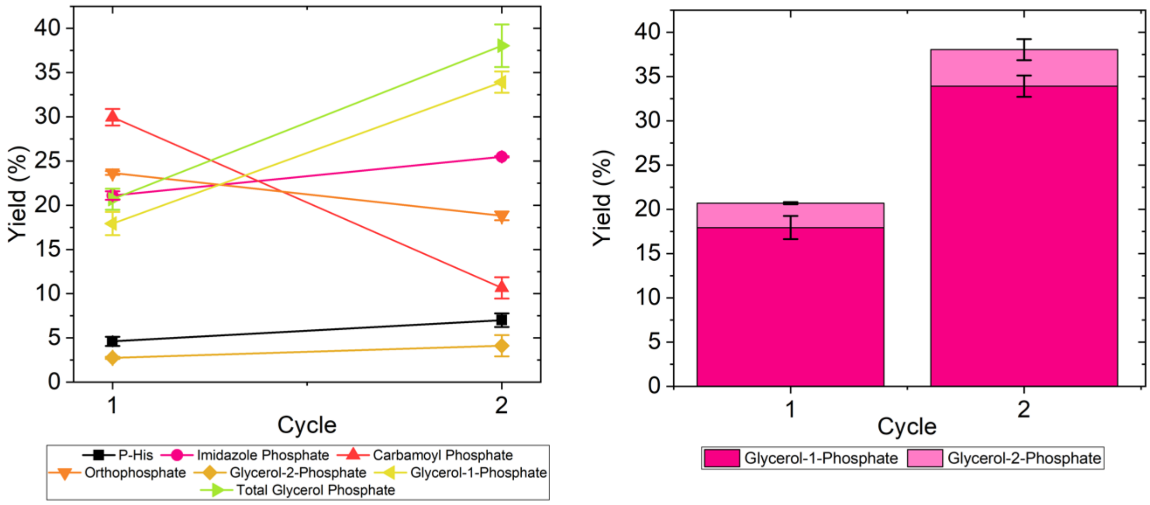

Supporting Figure 240: a) Changes in yield for all phosphate-containing products over two cycles for the reaction of 20 mM sodium phosphate dibasic + 500 mM glycerol + 230 mM potassium cyanate + 100 mM imidazole + 50 mM histidine at pH 6.5 and 22 °C. b) Change in yield for glycerol-1-phosphate and glycerol-2-phosphate over the course of two cycles.

S4.22 Wet/Dry Cycle for the phosphorylation of glycerol by imidazole phosphate at pH 6.5

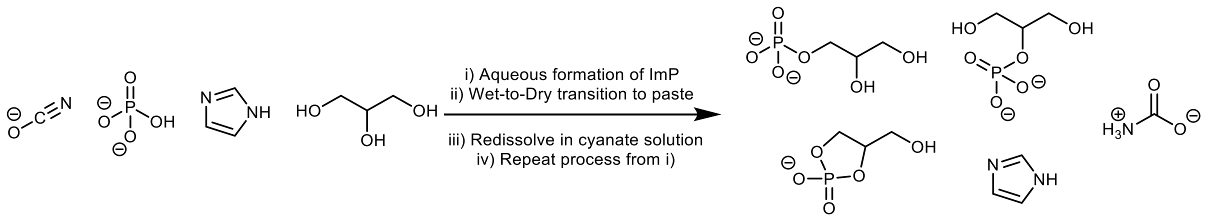

The experiment was carried out according to the procedure in S4.1 but without 100 mM of histidine and at pH 6.5. The pH rose to around pH 7.2 during the wet part of the cycle and was corrected back to pH 6.5 just before the drying of solution began. The experiment was repeated in duplicate. Supporting Figure 241 depict representative ^{31}P NMR spectra after each wet-dry cycle. The changes in yield after each wet-dry cycle for all phosphate containing species are shown in Supporting Figure 242a. The change in yield of glycerol-1-phosphate and glycerol-2-phosphate after each wet-dry cycle are shown in Supporting Figure 242b.

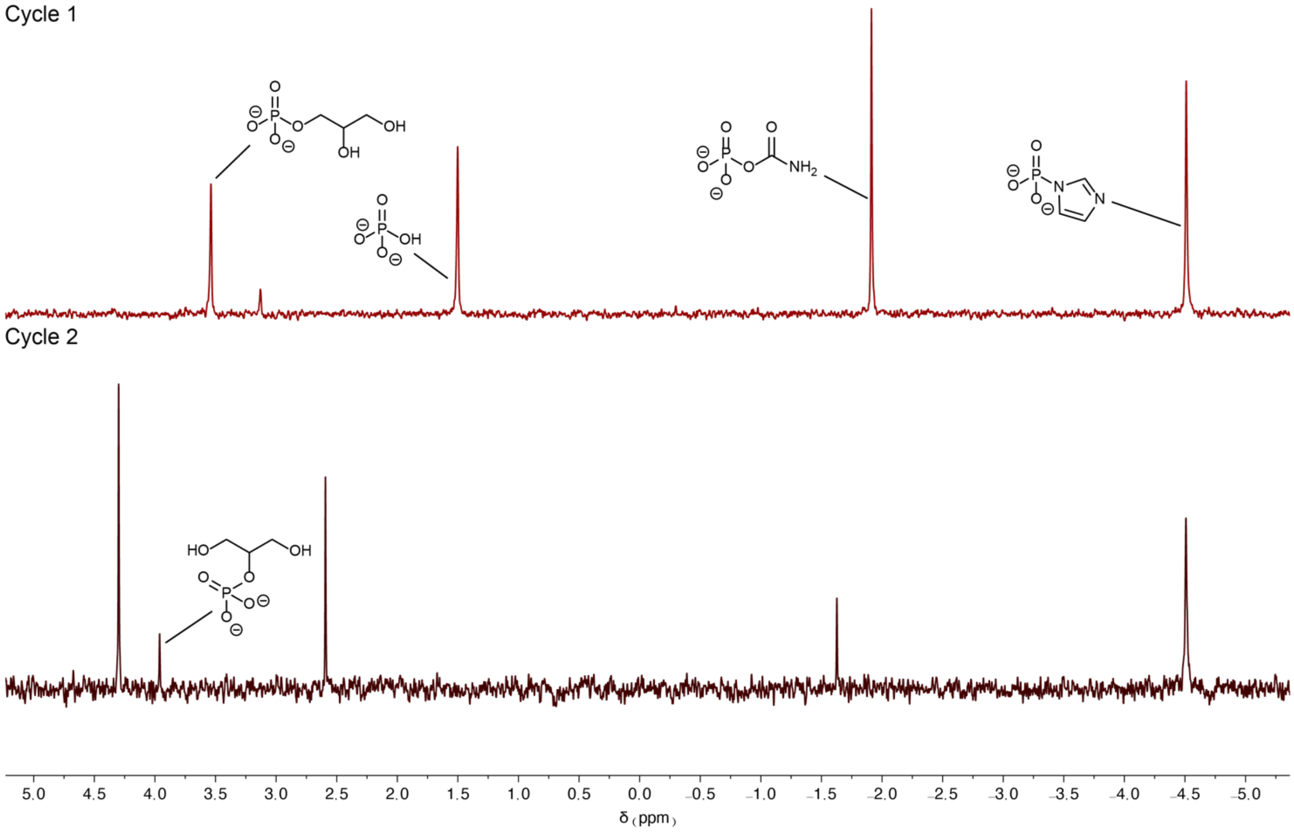

Supporting Figure 241: Representative ^{31}P-NMR spectra at the end of each cycle for the reaction of 20 mM sodium phosphate dibasic + 500 mM glycerol + 230 mM potassium cyanate + 100 mM imidazole at pH 6.5 and 22 °C.

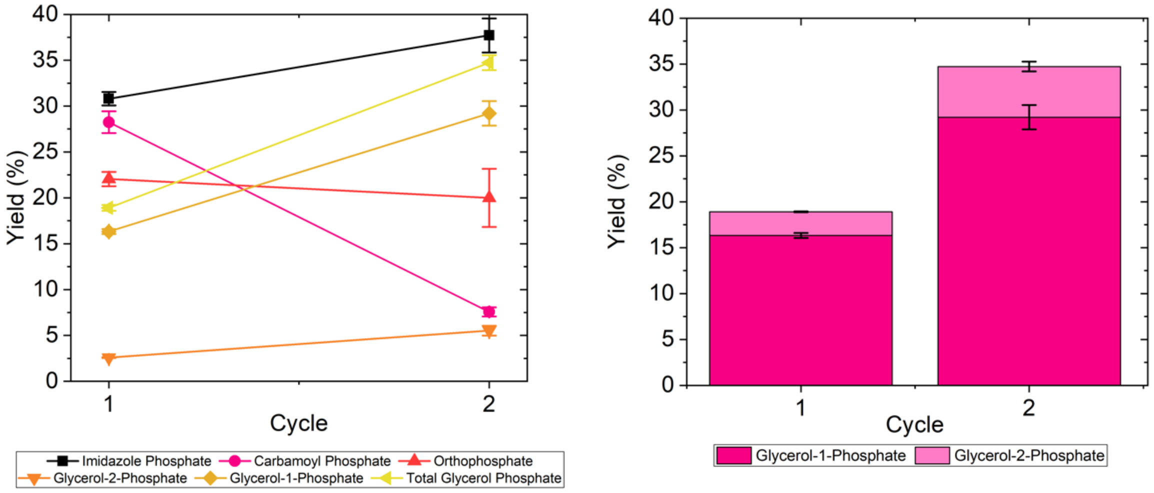

Supporting Figure 242: a) Changes in yield for all phosphate-containing products over two cycles for the reaction of 20 mM sodium phosphate dibasic + 500 mM glycerol + 230 mM potassium cyanate + 100 mM imidazole at pH 6.5 and 22 °C. b) Change in yield for glycerol-1-phosphate and glycerol-2-phosphate over the course of two cycles.

S4.23 Wet/Dry Cycle for the phosphorylation of glycerol by imidazole phosphate with 50 mM histidine catalyst at pH 8.0

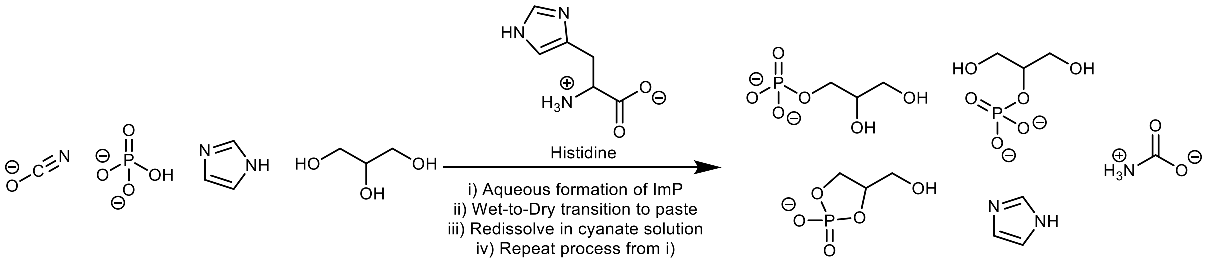

The experiment was carried out according to the procedure in S4.1 but with the 100 mM of histidine replaced by 50 mM histidine (31.0 mg, 0.2 mmol) and at pH 8.0. The pH rose to around pH 8.4 during the wet part of the cycle and was corrected back to pH 8.0 just before the drying of solution began. The experiment was repeated in duplicate. Supporting Figures 243 depict representative ^{31}P NMR spectra after each wet-dry cycle. The changes in yield after each wet-dry cycle for all phosphate containing species are shown in Supporting Figure 244a. The change in yield of glycerol-1-phosphate and glycerol-2-phosphate after each wet-dry cycle are shown in Supporting Figure 244b.

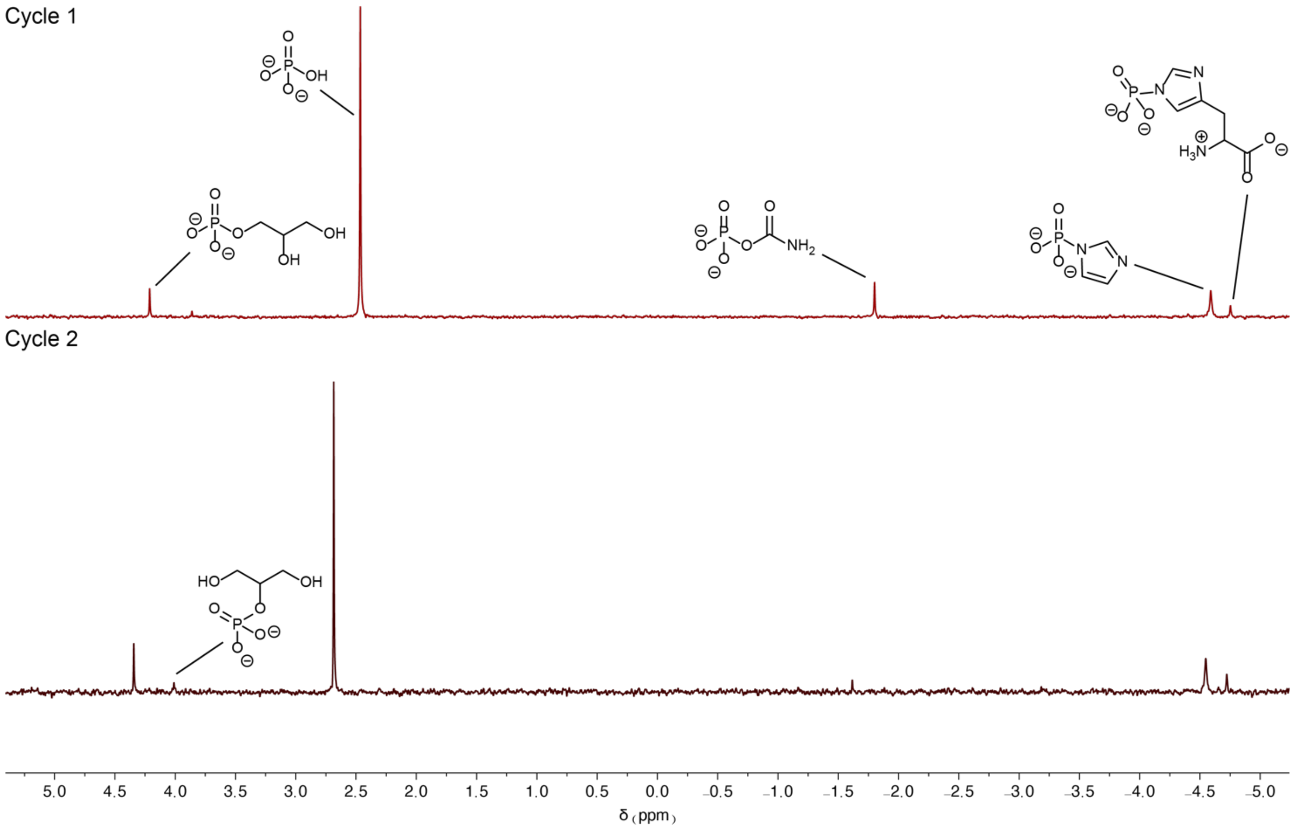

Supporting Figure 243: Representative ^{31}P-NMR spectra at the end of each cycle for the reaction of 20 mM sodium phosphate dibasic + 500 mM glycerol + 230 mM potassium cyanate + 100 mM imidazole + 50 mM histidine at pH 8.0 and 22 °C.

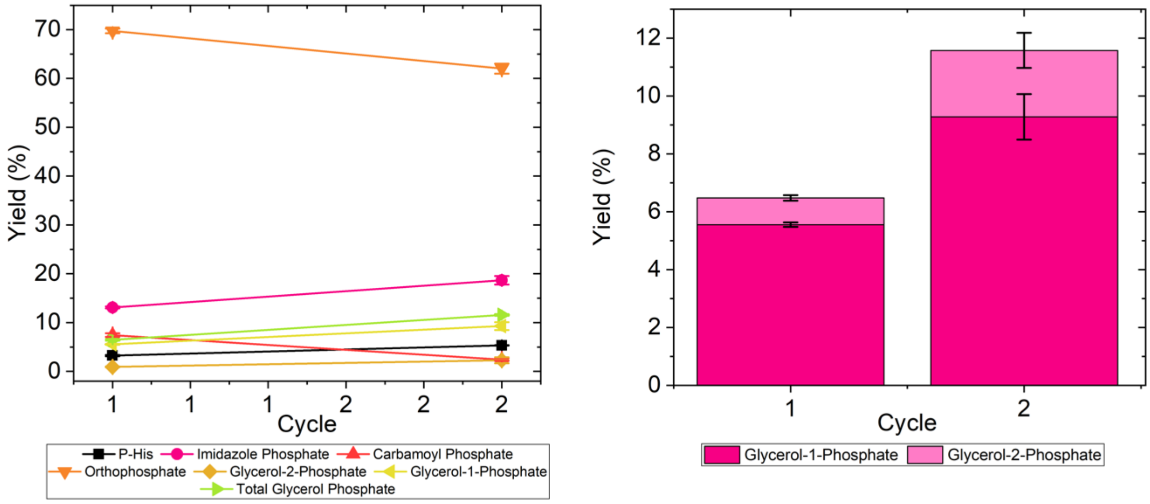

Supporting Figure 244: a) Changes in yield for all phosphate-containing products over two cycles for the reaction of 20 mM sodium phosphate dibasic + 500 mM glycerol + 230 mM potassium cyanate + 100 mM imidazole + 50 mM histidine at pH 8.0 and 22 °C. b) Change in yield for glycerol-1-phosphate and glycerol-2-phosphate over the course of two cycles.

S4.24 Wet/Dry Cycle for the phosphorylation of glycerol by imidazole phosphate at pH 8.0

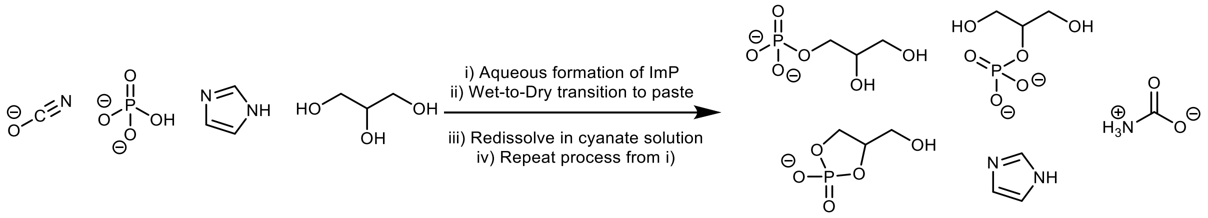

The experiment was carried out according to the procedure in S4.1 but without 100 mM of histidine and at pH 8.0. The pH rose to around pH 8.4 during the wet part of the cycle and was corrected back to pH 8.0 just before the drying of solution began. The experiment was repeated in duplicate. Supporting Figures 245 depict representative ^{31}P NMR spectra after each wet-dry cycle. The changes in yield after each wet-dry cycle for all phosphate containing species are shown in Supporting Figure 246a. The change in yield of glycerol-1-phosphate and glycerol-2-phosphate after each wet-dry cycle are shown in Supporting Figure 246b.

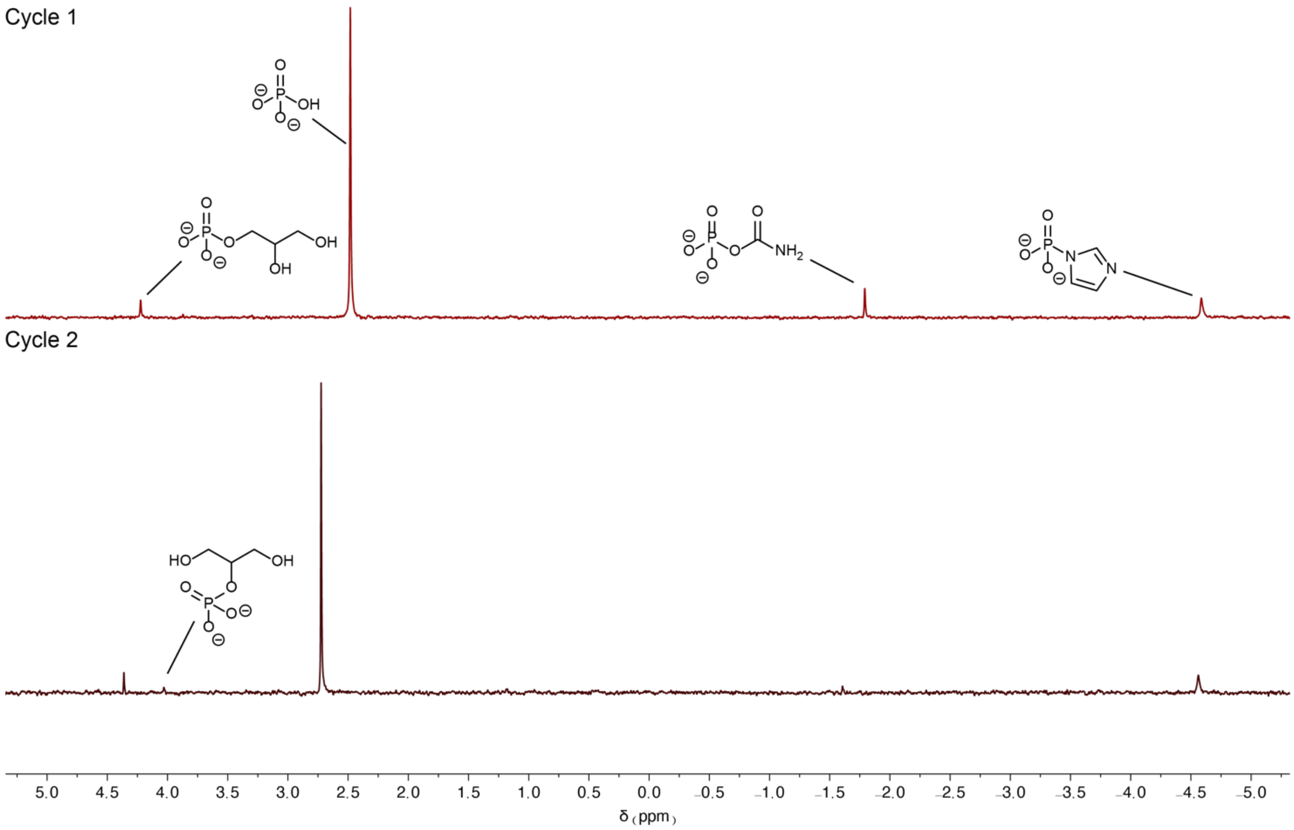

Supporting Figure 245: Representative ^{31}P-NMR spectra at the end of each cycle for the reaction of 20 mM sodium phosphate dibasic + 500 mM glycerol + 230 mM potassium cyanate + 100 mM imidazole at pH 8.0 and 22 °C.

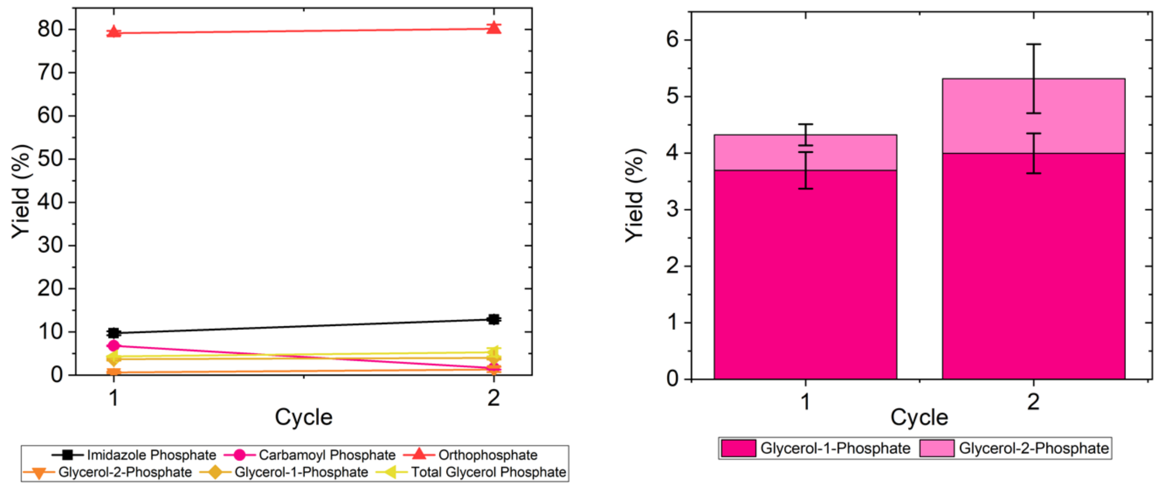

Supporting Figure 246: a) Changes in yield for all phosphate-containing products over two cycles for the reaction of 20 mM sodium phosphate dibasic + 500 mM glycerol + 230 mM potassium cyanate + 100 mM imidazole at pH 8.0 and 22 °C. b) Change in yield for glycerol-1-phosphate and glycerol-2-phosphate over the course of two cycles.

S4.25 Wet/Dry Cycle for the phosphorylation of glycerol by imidazole phosphate with 1 % weight/volume Montmorillonite

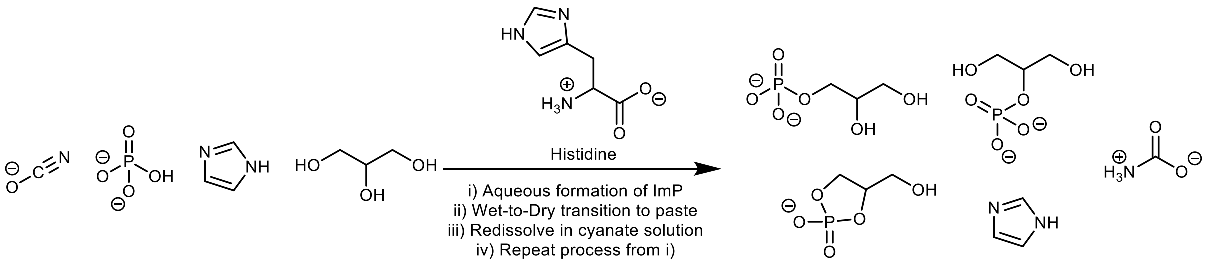

The experiment was carried out according to the procedure in S4.1 but with the 100 mM of histidine replaced by 50 mM histidine (31.0 mg, 0.2 mmol) with 1 % weight/volume Montmorillonite (40 mg). In the first cycle the Montmorillonite was added when the sample was put out to dry and in the second cycle the Montmorillonite was *in situ* for the whole wet/dry cycle. The experiment was repeated in duplicate. Supporting Figures 247 depict representative ^{31}P NMR spectra after each wet-dry cycle. The changes in yield after each wet-dry cycle for all phosphate containing species are shown in Supporting Figure 248a. The change in yield of glycerol-1-phosphate and glycerol-2-phosphate after each wet-dry cycle are shown in Supporting Figure 248b.

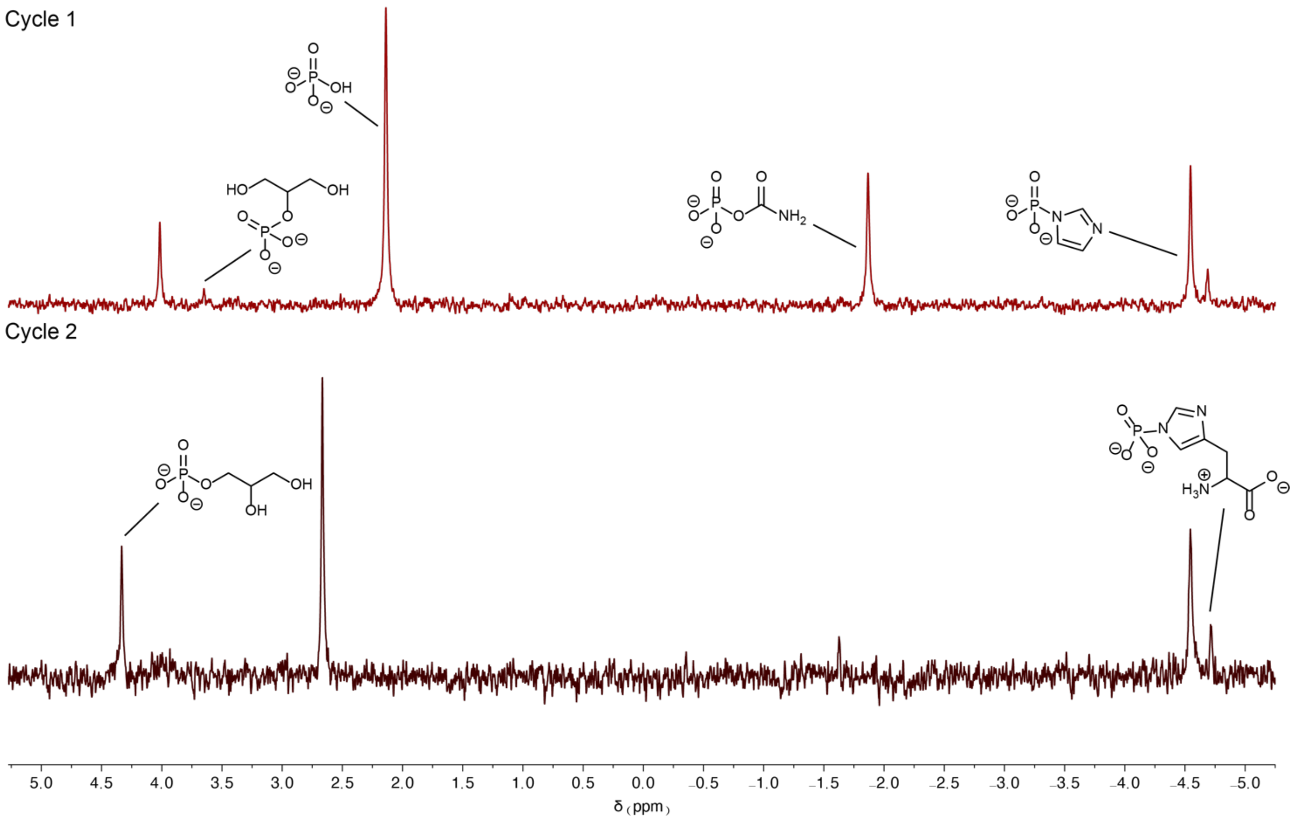

Supporting Figure 247: Representative ^{31}P-NMR spectra at the end of each cycle for the reaction of 20 mM sodium phosphate dibasic + 500 mM glycerol + 230 mM potassium cyanate + 100 mM imidazole + 50 mM histidine at pH 7.3 and 22 °C with 1 % weight/volume Montmorillonite.

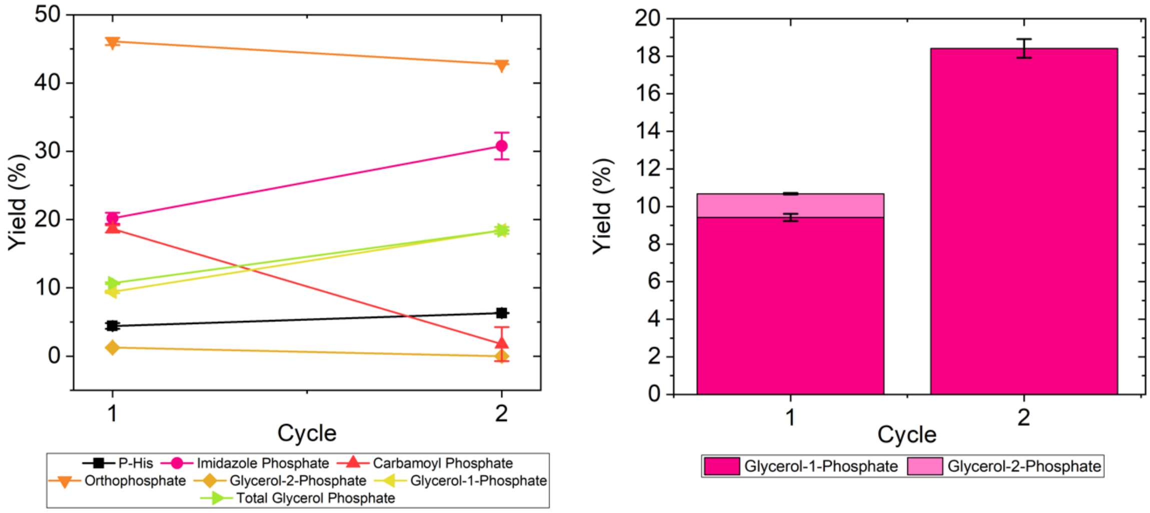

Supporting Figure 248: a) Changes in yield for all phosphate-containing products over two cycles for the reaction of 20 mM sodium phosphate dibasic + 500 mM glycerol + 230 mM potassium cyanate + 100 mM imidazole + 50 mM histidine at pH 7.3 and 22 °C with 1 % weight/volume Montmorillonite. b) Change in yield for glycerol-1-phosphate and glycerol-2-phosphate over the course of two cycles.

S4.26 Wet/Dry Cycle for the phosphorylation of glycerol by imidazole phosphate with 1 % weight/volume Montmorillonite

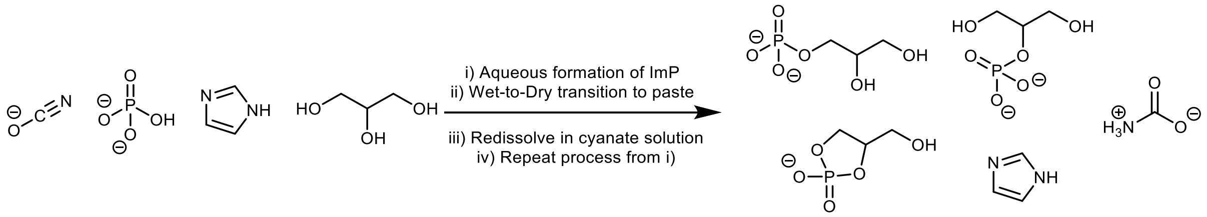

The experiment was carried out according to the procedure in S4.1 but without 100 mM of histidine and with 1 % weight/volume Montmorillonite (40 mg). In the first cycle the Montmorillonite was added when the sample was put out to dry and in the second cycle the Montmorillonite was *in situ* for the whole wet/dry cycle. The experiment was repeated in duplicate. Supporting Figure 249 depict representative ^{31}P NMR spectra after each wet-dry cycle. The changes in yield after each wet-dry cycle for all phosphate containing species are shown in Supporting Figure 250a. The change in yield of glycerol-1-phosphate and glycerol-2-phosphate after each wet-dry cycle are shown in Supporting Figure 250b.

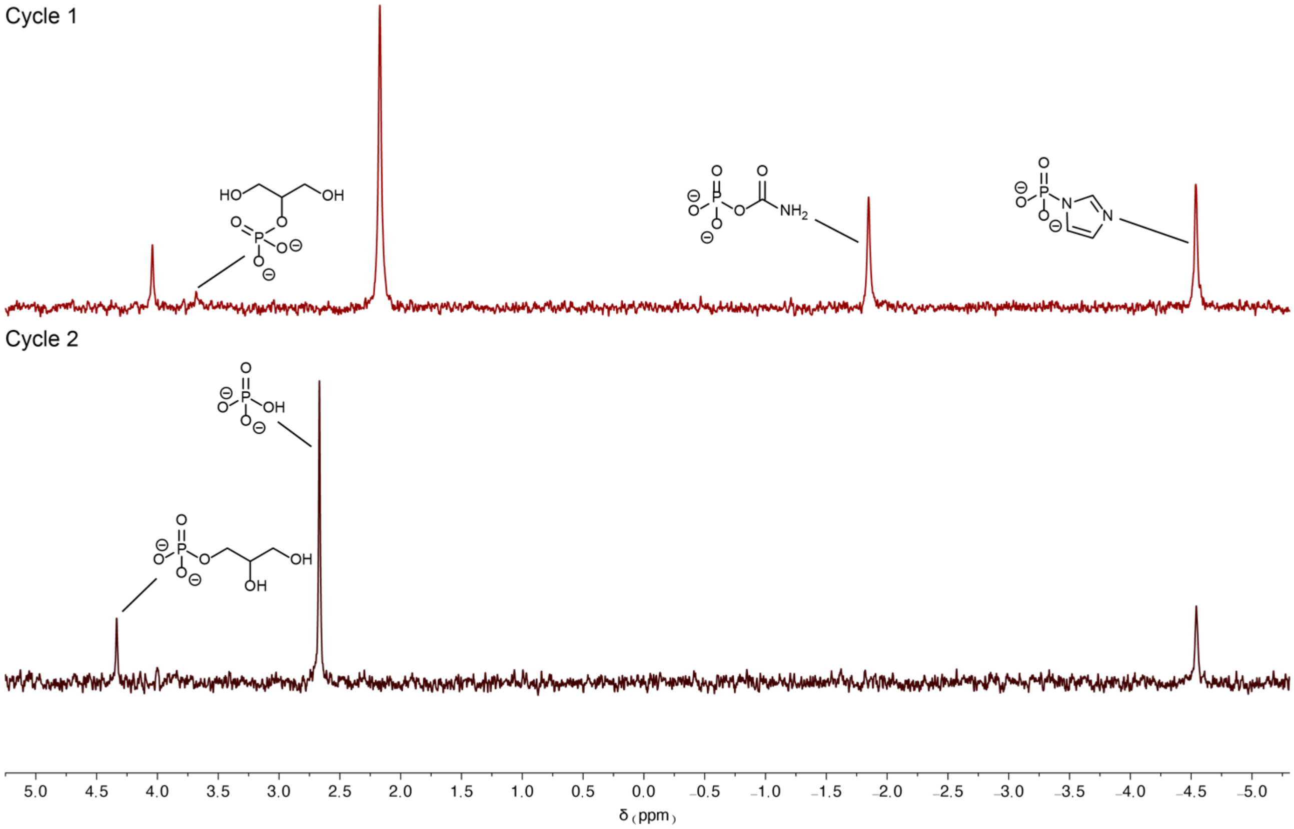

Supporting Figure 249: Representative ^{31}P-NMR spectra at the end of each cycle for the reaction of 20 mM sodium phosphate dibasic + 500 mM glycerol + 230 mM potassium cyanate + 100 mM imidazole at pH 7.3 and 22 °C with 1 % weight/volume Montmorillonite.

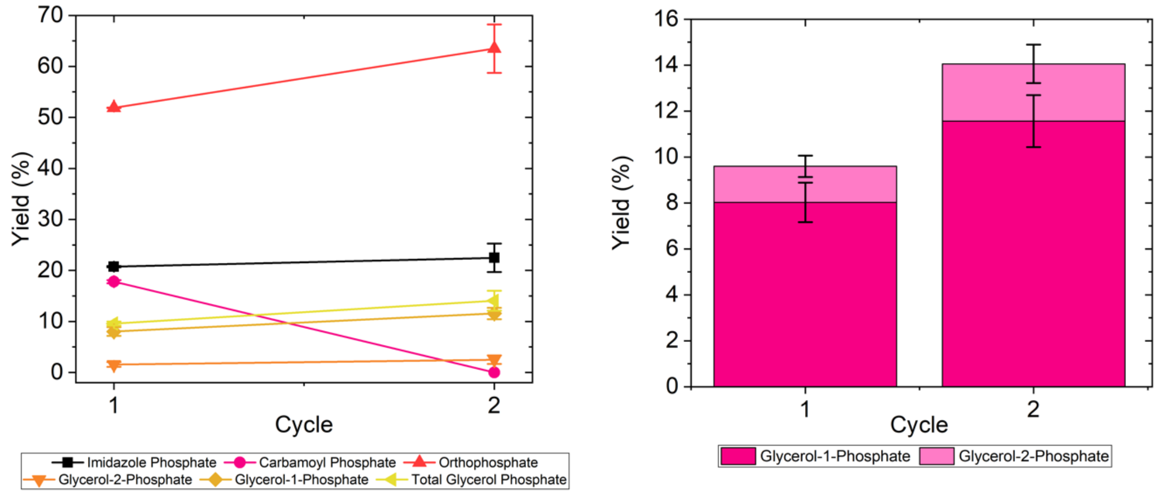

Supporting Figure 250: a) Changes in yield for all phosphate-containing products over two cycles for the reaction of 20 mM sodium phosphate dibasic + 500 mM glycerol + 230 mM potassium cyanate + 100 mM imidazole at pH 7.3 and 22 °C with 1 % weight/volume Montmorillonite. b) Change in yield for glycerol-1-phosphate and glycerol-2-phosphate over the course of two cycles.

S4.27 Wet/Dry Cycle for the phosphorylation of glycerol by imidazole phosphate with 1 % weight/weight Montmorillonite

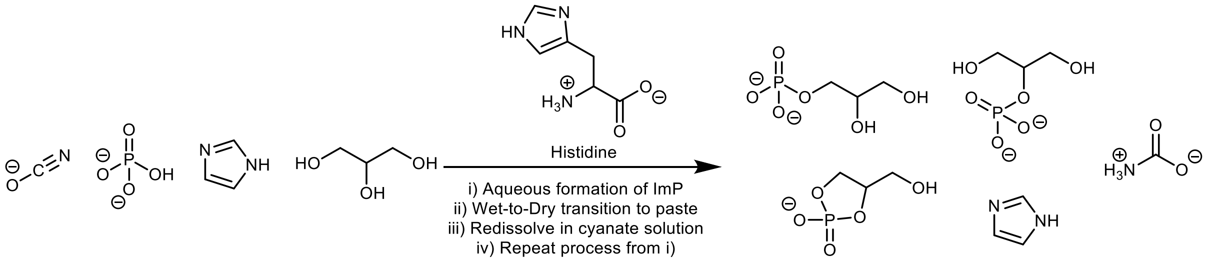

The experiment was carried out according to the procedure in S4.1 but with the 100 mM of histidine replaced by 50 mM histidine (31.0 mg, 0.2 mmol) with 1 % weight/weight Montmorillonite (3 mg). In the first cycle the Montmorillonite was added when the sample was put out to dry and in the second cycle the Montmorillonite was *in situ* for the whole wet/dry cycle. The experiment was repeated in duplicate. Supporting Figure 251 depict representative ^{31}P NMR spectra after each wet-dry cycle. The changes in yield after each wet-dry cycle for all phosphate containing species are shown in Supporting Figure 252a. The change in yield of glycerol-1-phosphate and glycerol-2-phosphate after each wet-dry cycle are shown in Supporting Figure 252b.

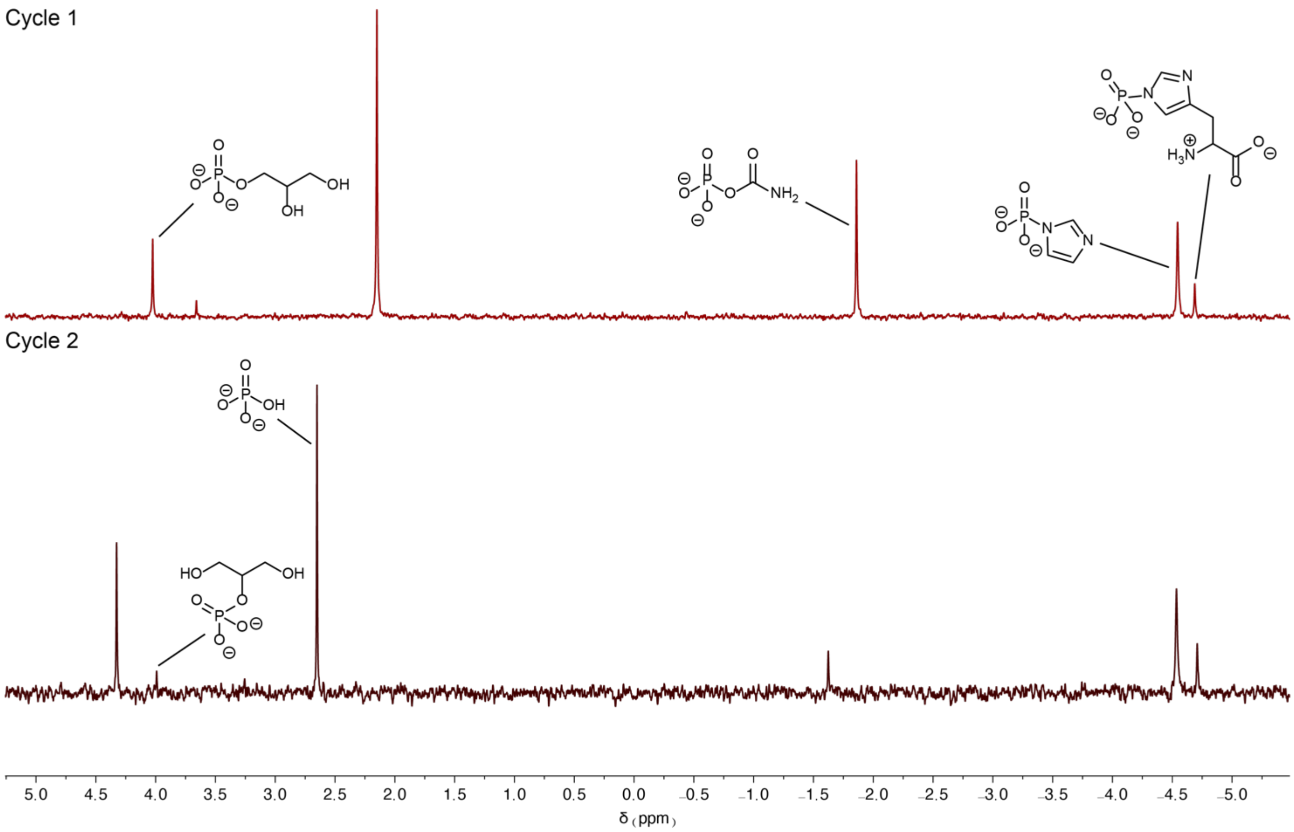

Supporting Figure 251: Representative ^{31}P-NMR spectra at the end of each cycle for the reaction of 20 mM sodium phosphate dibasic + 500 mM glycerol + 230 mM potassium cyanate + 100 mM imidazole + 50 mM histidine at pH 7.3 and 22 °C with 1 % weight/weight Montmorillonite.

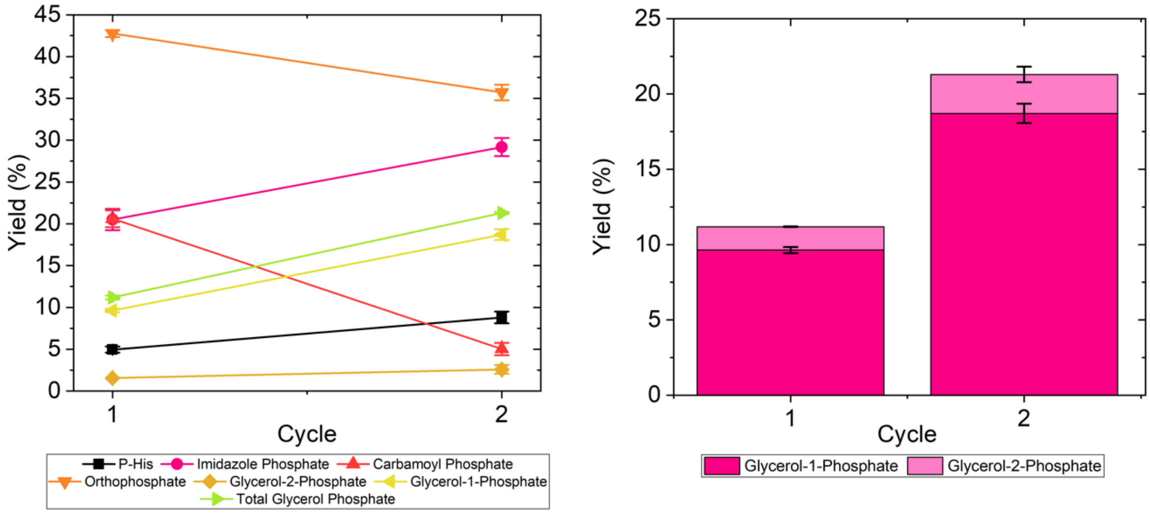

Supporting Figure 252: a) Changes in yield for all phosphate-containing products over two cycles for the reaction of 20 mM sodium phosphate dibasic + 500 mM glycerol + 230 mM potassium cyanate + 100 mM imidazole + 50 mM histidine at pH 7.3 and 22 °C with 1 % weight/weight Montmorillonite. b) Change in yield for glycerol-1-phosphate and glycerol-2-phosphate over the course of two cycles.

S4.28 Wet/Dry Cycle for the phosphorylation of glycerol by imidazole phosphate with 1 % weight/weight Montmorillonite

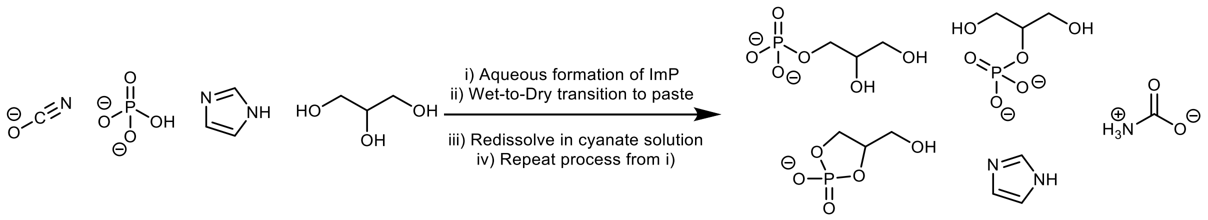

The experiment was carried out according to the procedure in S4.1 but without 100 mM of histidine and with 1 % weight/weight Montmorillonite (3 mg). In the first cycle the Montmorillonite was added when the sample was put out to dry and in the second cycle the Montmorillonite was *in situ* for the whole wet/dry cycle. The experiment was repeated in duplicate. Supporting Figure 253 depict representative ^{31}P NMR spectra after each wet-dry cycle. The changes in yield after each wet-dry cycle for all phosphate containing species are shown in Supporting Figure 254a. The change in yield of glycerol-1-phosphate and glycerol-2-phosphate after each wet-dry cycle are shown in Supporting Figure 254b.

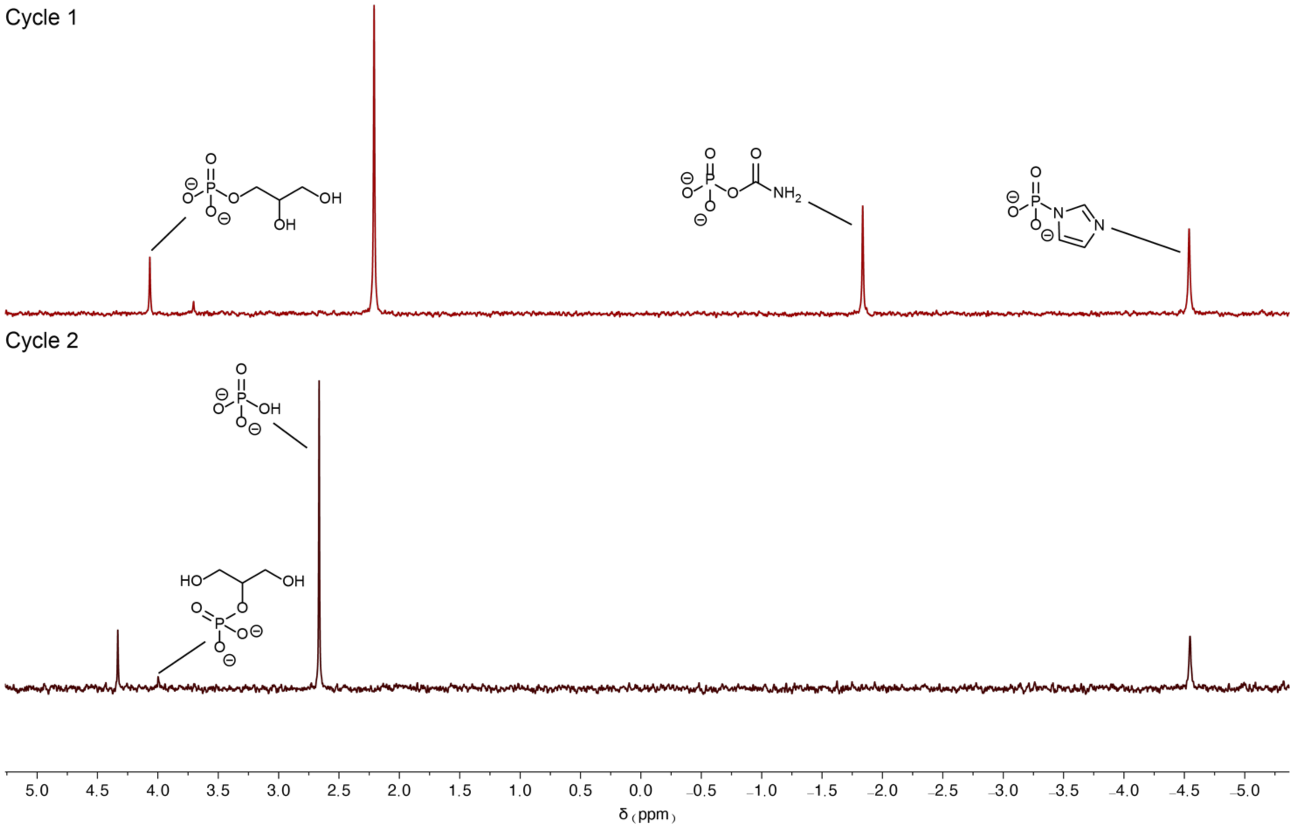

Supporting Figure 253: Representative ^{31}P-NMR spectra at the end of each cycle for the reaction of 20 mM sodium phosphate dibasic + 500 mM glycerol + 230 mM potassium cyanate + 100 mM imidazole at pH 7.3 and 22 °C with 1 % weight/weight Montmorillonite.

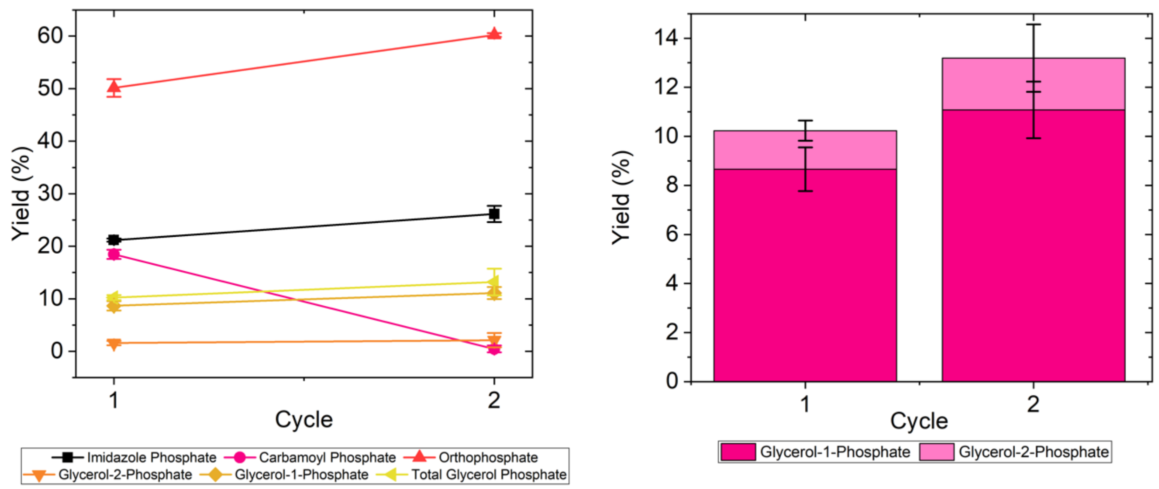

Supporting Figure 254: a) Changes in yield for all phosphate-containing products over two cycles for the reaction of 20 mM sodium phosphate dibasic + 500 mM glycerol + 230 mM potassium cyanate + 100 mM imidazole at pH 7.3 and 22 °C with 1 % weight/weight Montmorillonite. b) Change in yield for glycerol-1-phosphate and glycerol-2-phosphate over the course of two cycles.

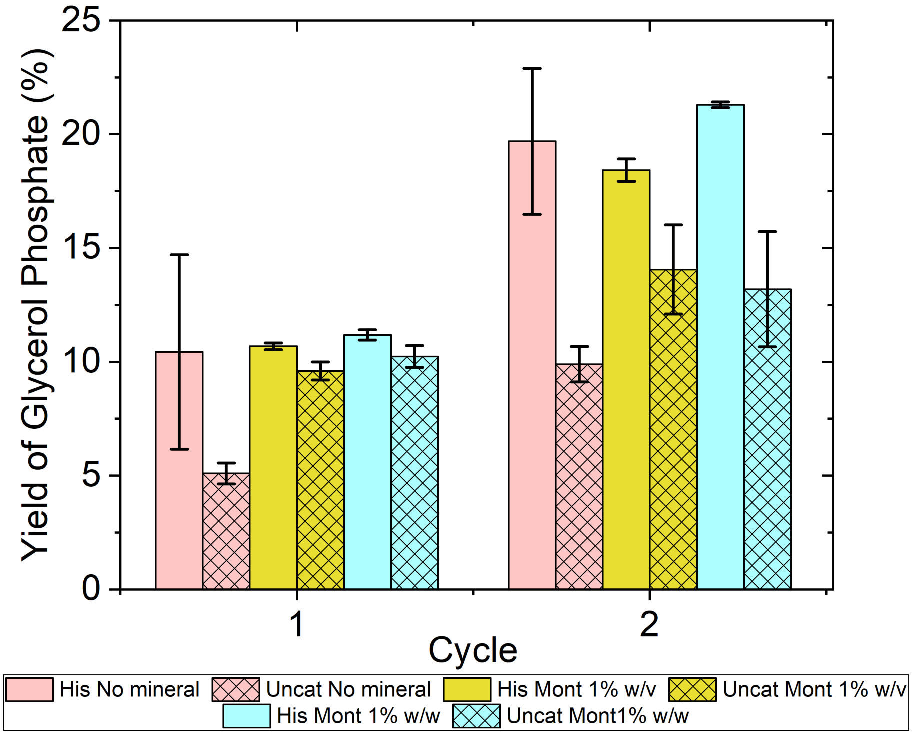

Supporting Figure 255: The effect of the minerals montmorillonite on the uncatalysed and histidine-catalysed phosphorylation of glycerol in the physicochemical orthophosphate cycle. The changes in glycerol-phosphate yield (summation of the yields of glycerol-1-phosphate and glycerol-2-phosphate) over two cycles for the reaction of 50 mM histidine, 20 mM sodium phosphate dibasic + 500 mM glycerol + 230 mM potassium cyanate + 100 mM imidazole at pH 7.3 and 22 °C.

Supporting Figure 255 shows that in the presence of the mineral montmorillonite the histidyl-catalysed phosphorylations still occur.

S4.29 Wet/Dry Cycle for the phosphorylation of glycerol by imidazole phosphate with 1 % weight/volume Hydroxyapatite

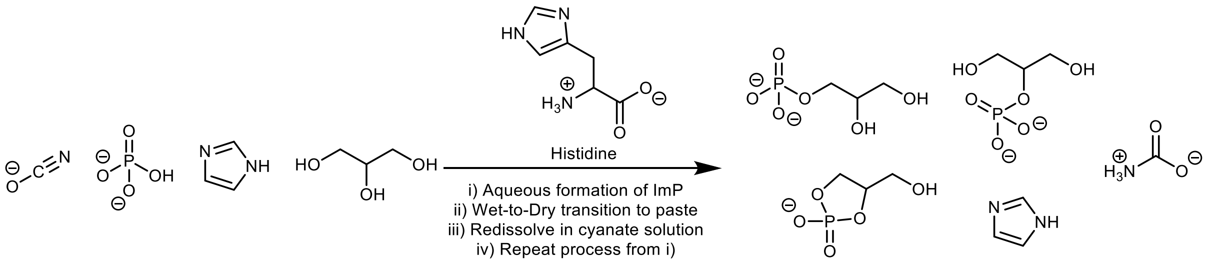

The experiment was carried out according to the procedure in S4.1 but with the 100 mM of histidine replaced by 50 mM histidine (31.0 mg, 0.2 mmol) with 1 % weight/volume Hydroxyapatite (40 mg). The experiment was repeated in duplicate. Supporting Figures 256 depict representative ^{31}P NMR spectra after each wet-dry cycle. The changes in yield after each wet-dry cycle for all phosphate containing species are shown in Supporting Figure 257a. The change in yield of glycerol-1-phosphate and glycerol-2-phosphate after each wet-dry cycle are shown in Supporting Figure 257b.

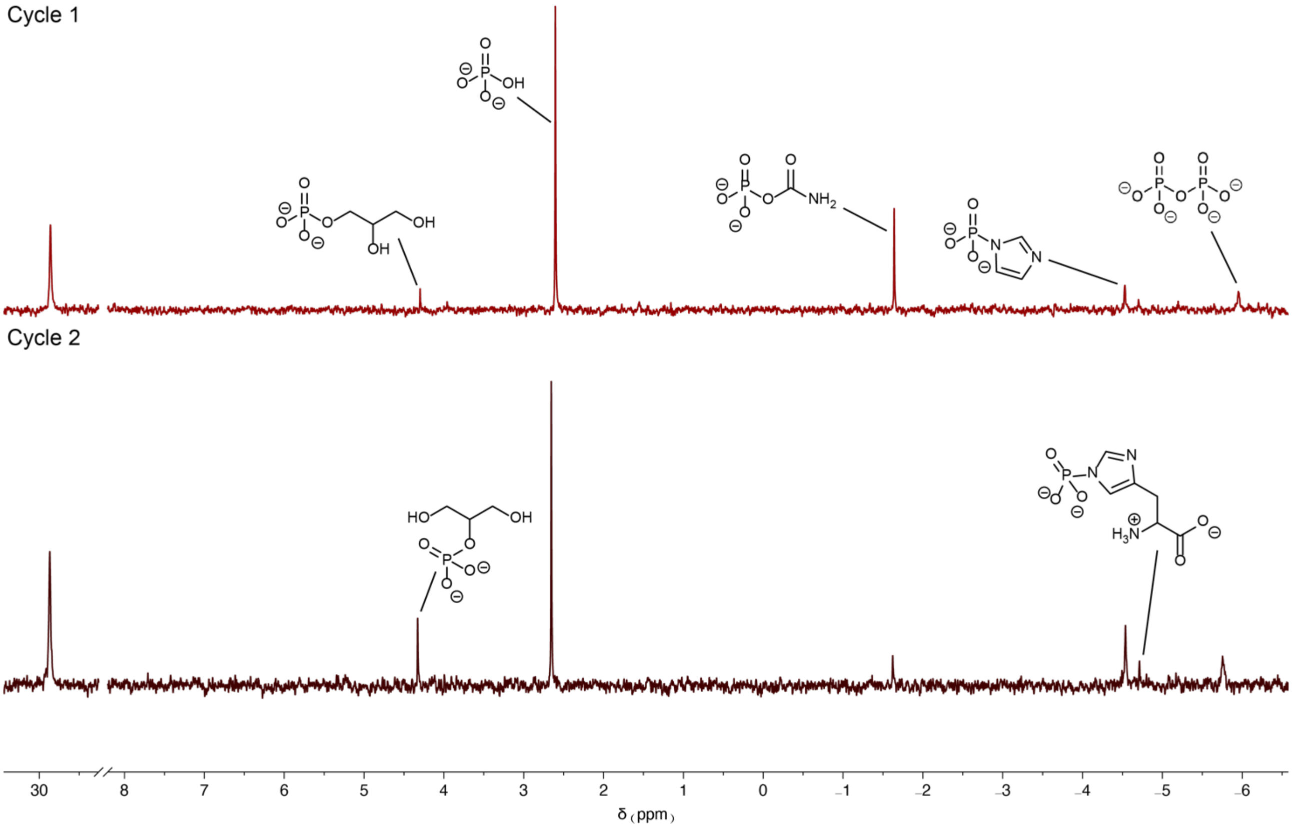

Supporting Figure 256: Representative ^{31}P-NMR spectra at the end of each cycle for the reaction of 20 mM sodium phosphate dibasic + 500 mM glycerol + 230 mM potassium cyanate + 100 mM imidazole + 50 mM histidine at pH 7.3 and 22 °C with 1 % weight/volume Hydroxyapatite.

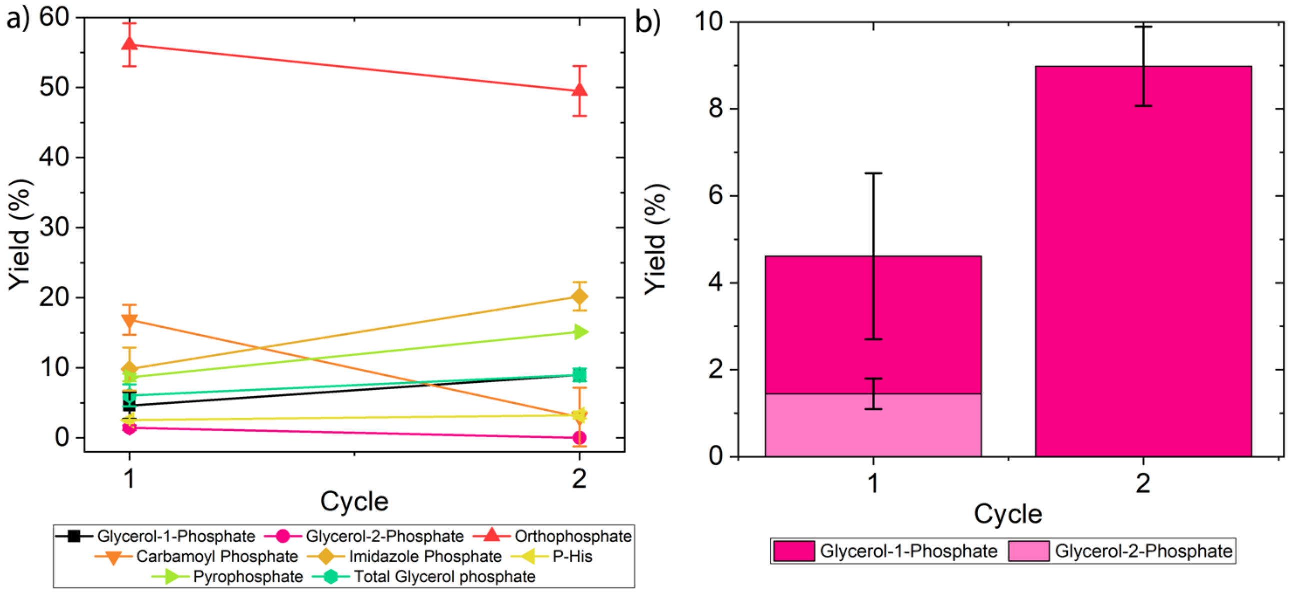

Supporting Figure 257: a) Changes in yield for all phosphate-containing products over two cycles for the reaction of 20 mM sodium phosphate dibasic + 500 mM glycerol + 230 mM potassium cyanate + 100 mM imidazole + 50 mM histidine at pH 7.3 and 22 °C with 1 % weight/volume Hydroxyapatite. b) Change in yield for glycerol-1-phosphate and glycerol-2-phosphate over the course of two cycles.

S4.30 Wet/Dry Cycle for the phosphorylation of glycerol by imidazole phosphate with 1 % weight/volume Hydroxyapatite

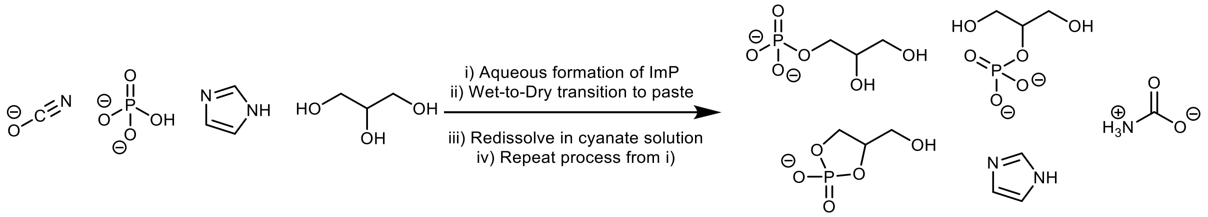

The experiment was carried out according to the procedure in S4.1 but without 100 mM of histidine and with 1 % weight/volume Hydroxyapatite (40 mg). The experiment was repeated in duplicate. Supporting Figure 258 depict representative ^{31}P NMR spectra after each wet-dry cycle. The changes in yield after each wet-dry cycle for all phosphate containing species are shown in Supporting Figure 259a. The change in yield of glycerol-1-phosphate and glycerol-2-phosphate after each wet-dry cycle are shown in Supporting Figure 259b.

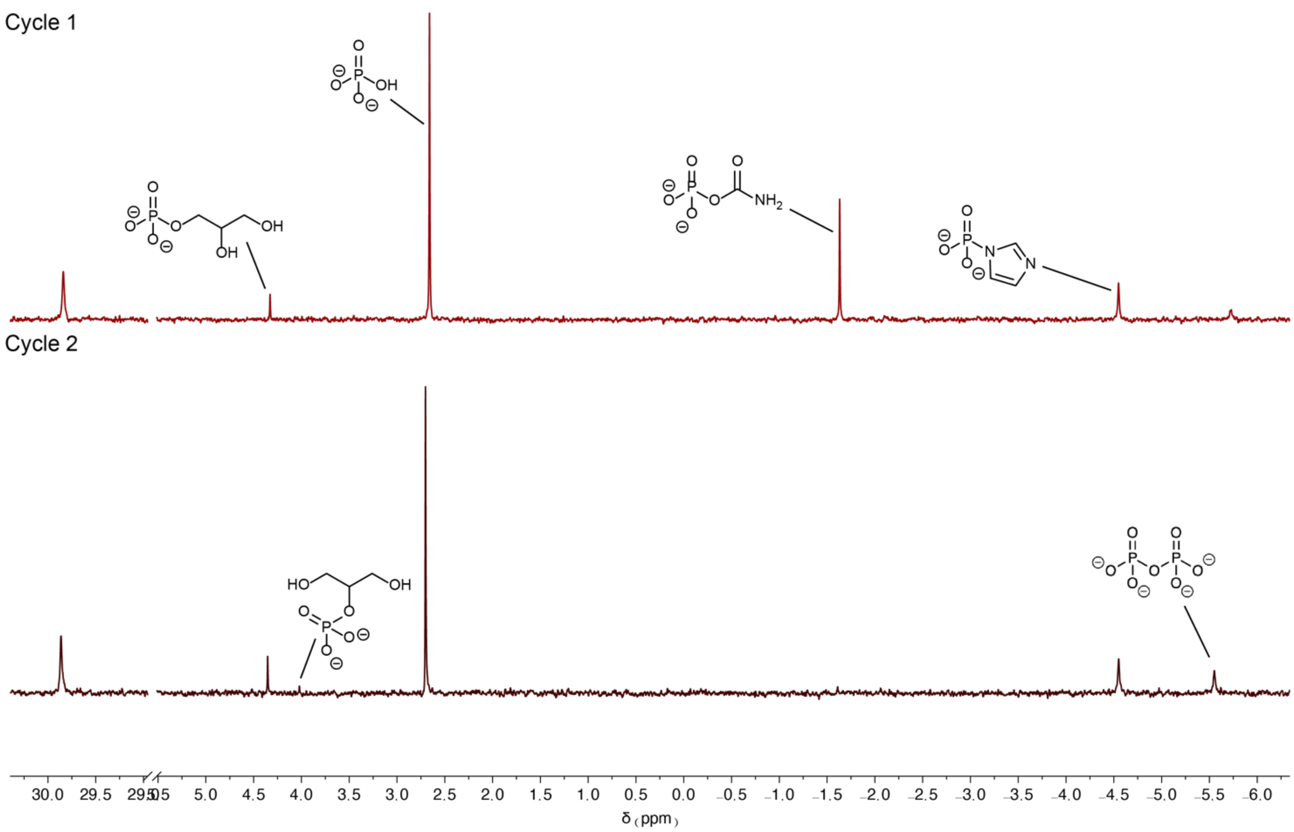

Supporting Figure 258: Representative ^{31}P-NMR spectra at the end of each cycle for the reaction of 20 mM sodium phosphate dibasic + 500 mM glycerol + 230 mM potassium cyanate + 100 mM imidazole at pH 7.3 and 22 °C with 1 % weight/volume Hydroxyapatite.

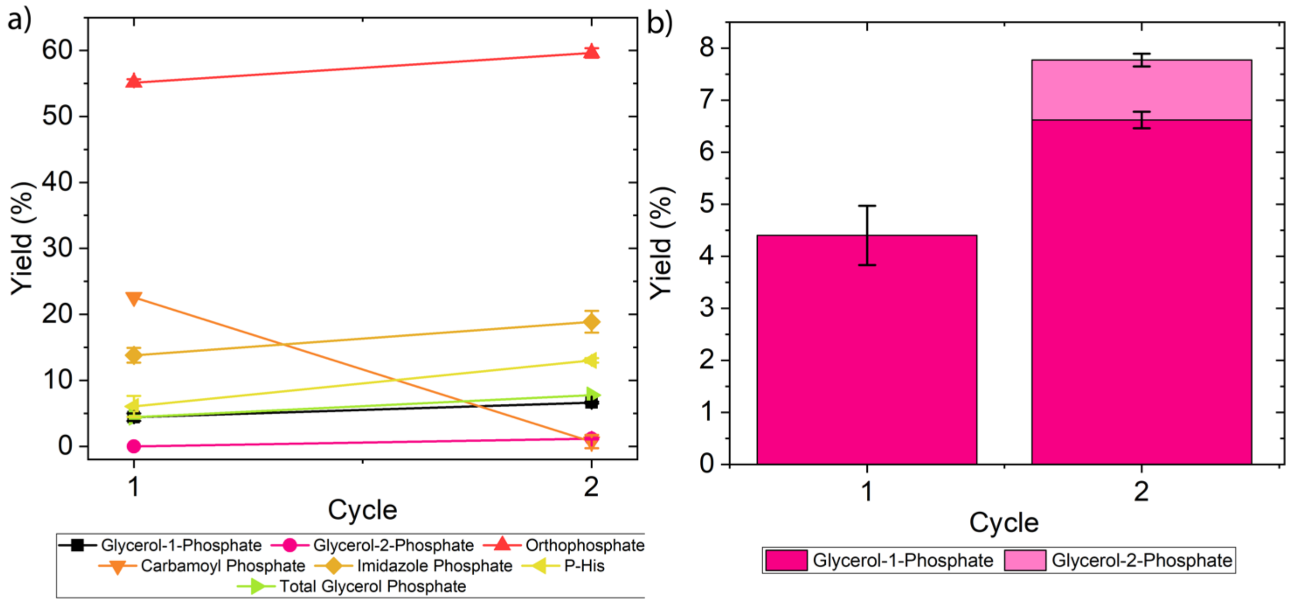

Supporting Figure 259: a) Changes in yield for all phosphate-containing products over two cycles for the reaction of 20 mM sodium phosphate dibasic + 500 mM glycerol + 230 mM potassium cyanate + 100 mM imidazole at pH 7.3 and 22 °C with 1 % weight/volume Hydroxyapatite. b) Change in yield for glycerol-1-phosphate and glycerol-2-phosphate over the course of two cycles.

S4.31 Wet/Dry Cycle for the phosphorylation of glycerol by imidazole phosphate with 1 % weight/weight Hydroxyapatite

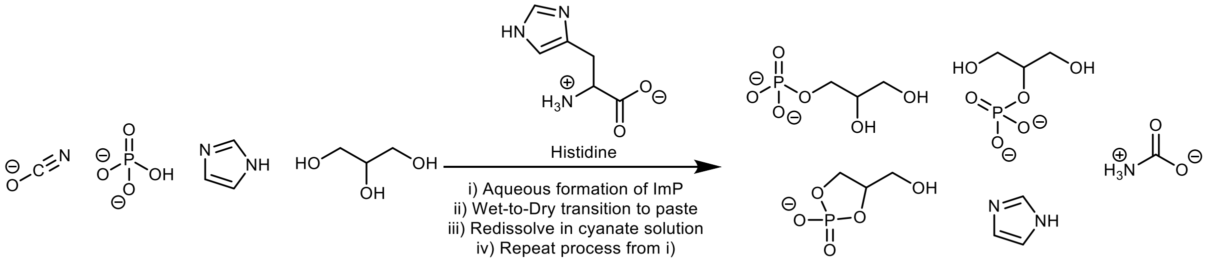

The experiment was carried out according to the procedure in S4.1 but with the 100 mM of histidine replaced by 50 mM histidine (31.0 mg, 0.2 mmol) with 1 % weight/weight Hydroxyapatite (3 mg). The experiment was repeated in duplicate. Supporting Figure 260 depict representative ^{31}P NMR spectra after each wet-dry cycle. The changes in yield after each wet-dry cycle for all phosphate containing species are shown in Supporting Figure 261a. The change in yield of glycerol-1-phosphate and glycerol-2-phosphate after each wet-dry cycle are shown in Supporting Figure 261b.

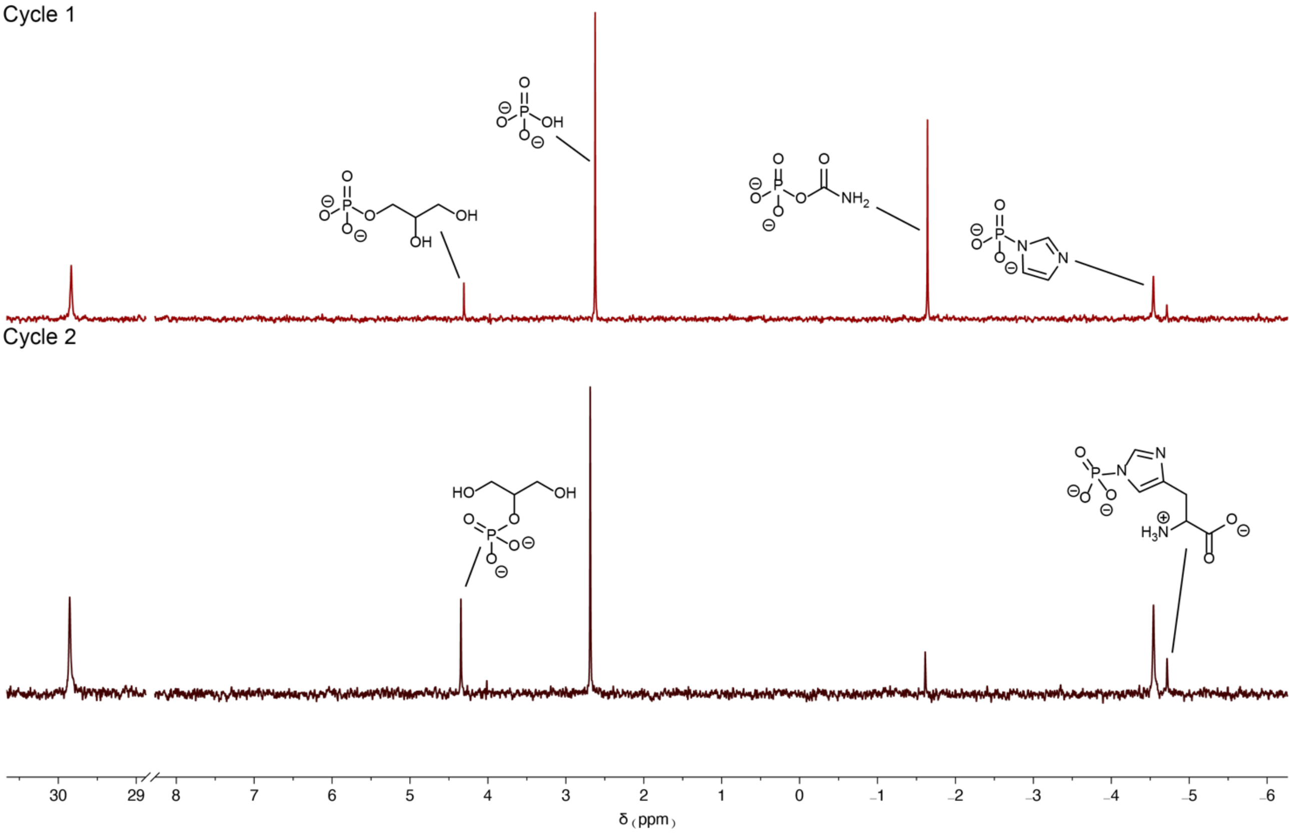

Supporting Figure 260: Representative ^{31}P-NMR spectra at the end of each cycle for the reaction of 20 mM sodium phosphate dibasic + 500 mM glycerol + 230 mM potassium cyanate + 100 mM imidazole + 50 mM histidine at pH 7.3 and 22 °C with 1 % weight/weight Hydroxyapatite.

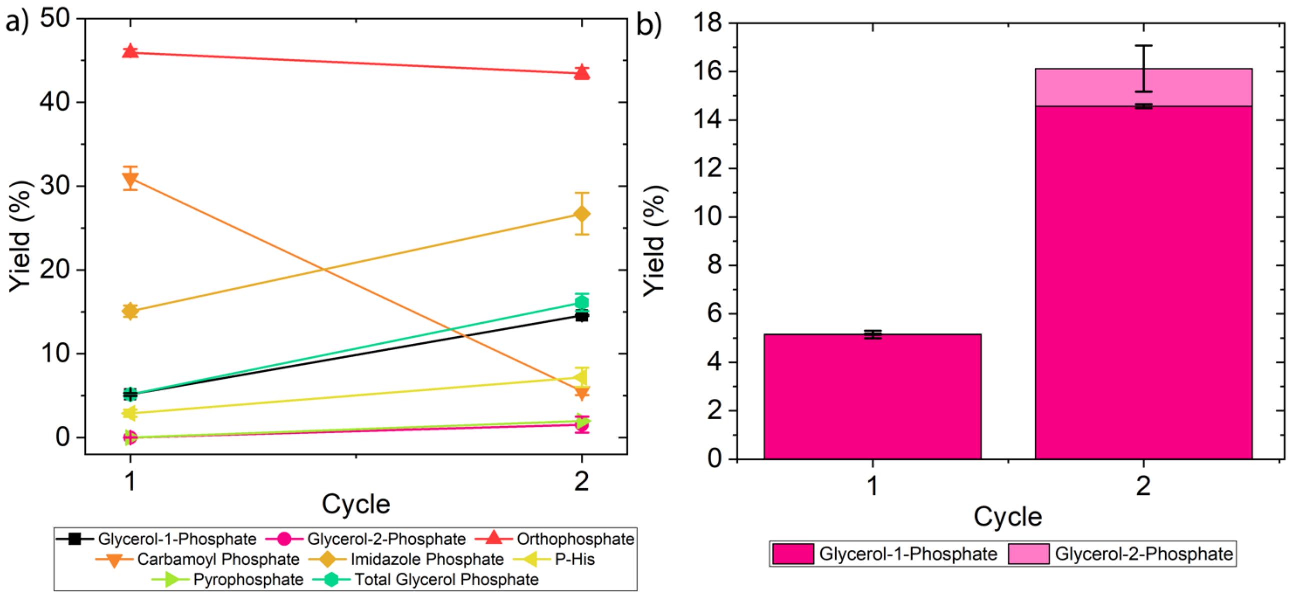

Supporting Figure 261: a) Changes in yield for all phosphate-containing products over two cycles for the reaction of 20 mM sodium phosphate dibasic + 500 mM glycerol + 230 mM potassium cyanate + 100 mM imidazole + 50 mM histidine at pH 7.3 and 22 °C with 1 % weight/weight Hydroxyapatite. b) Change in yield for glycerol-1-phosphate and glycerol-2-phosphate over the course of two cycles.

S4.32 Wet/Dry Cycle for the phosphorylation of glycerol by imidazole phosphate with 1 % weight/weight Hydroxyapatite

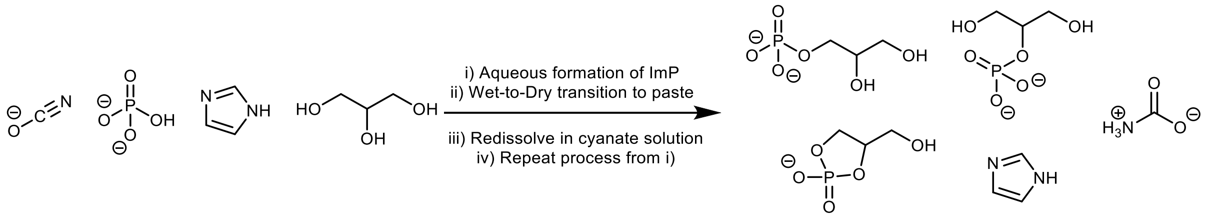

The experiment was carried out according to the procedure in S4.1 but without 100 mM of histidine and with 1 % weight/weight Hydroxyapatite (3 mg). The experiment was repeated in duplicate. Supporting Figure 262 depict representative ^{31}P NMR spectra after each wet-dry cycle. The changes in yield after each wet-dry cycle for all phosphate containing species are shown in Supporting Figure 263a. The change in yield of glycerol-1-phosphate and glycerol-2-phosphate after each wet-dry cycle are shown in Supporting Figure 263b.

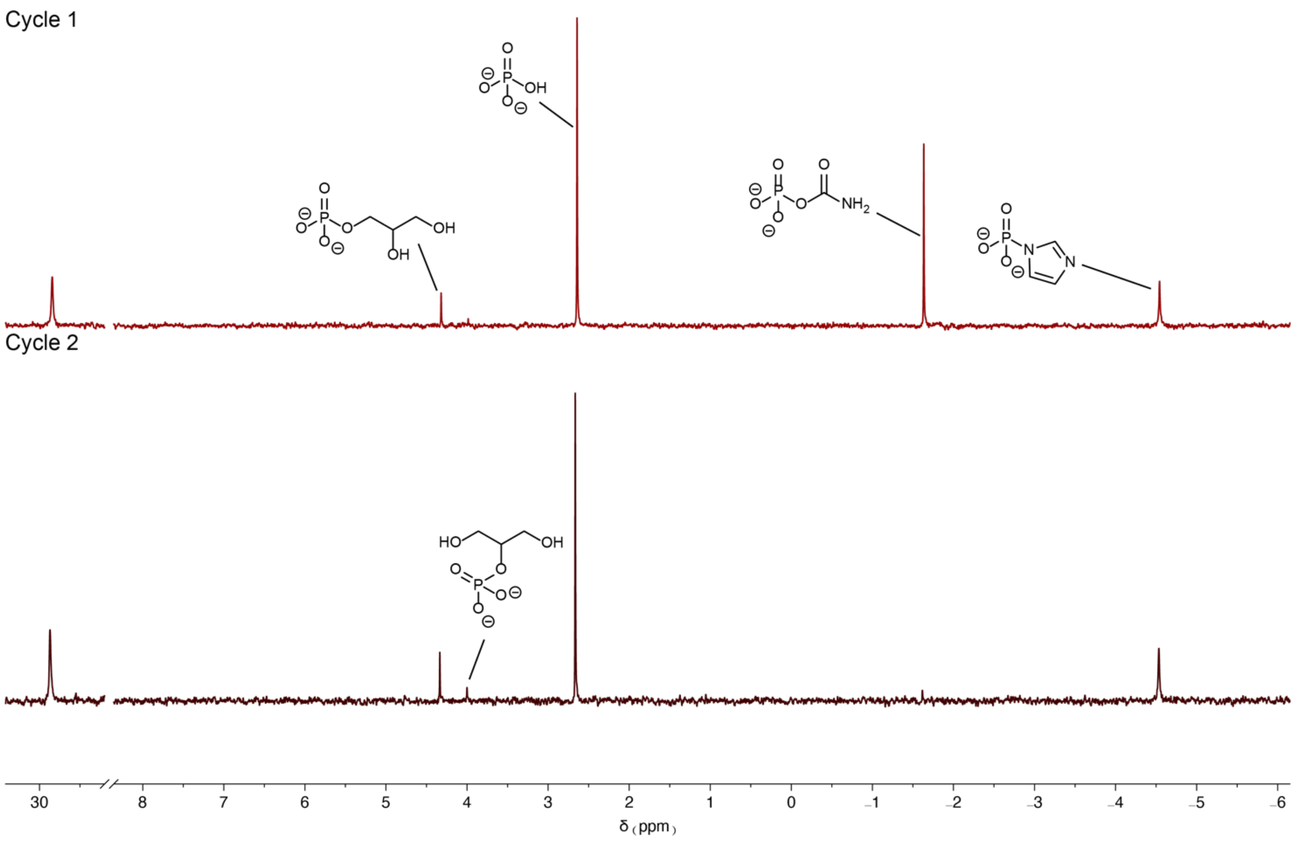

Supporting Figure 262: Representative ^{31}P-NMR spectra at the end of each cycle for the reaction of 20 mM sodium phosphate dibasic + 500 mM glycerol + 230 mM potassium cyanate + 100 mM imidazole at pH 7.3 and 22 °C with 1 % weight/weight Hydroxyapatite.

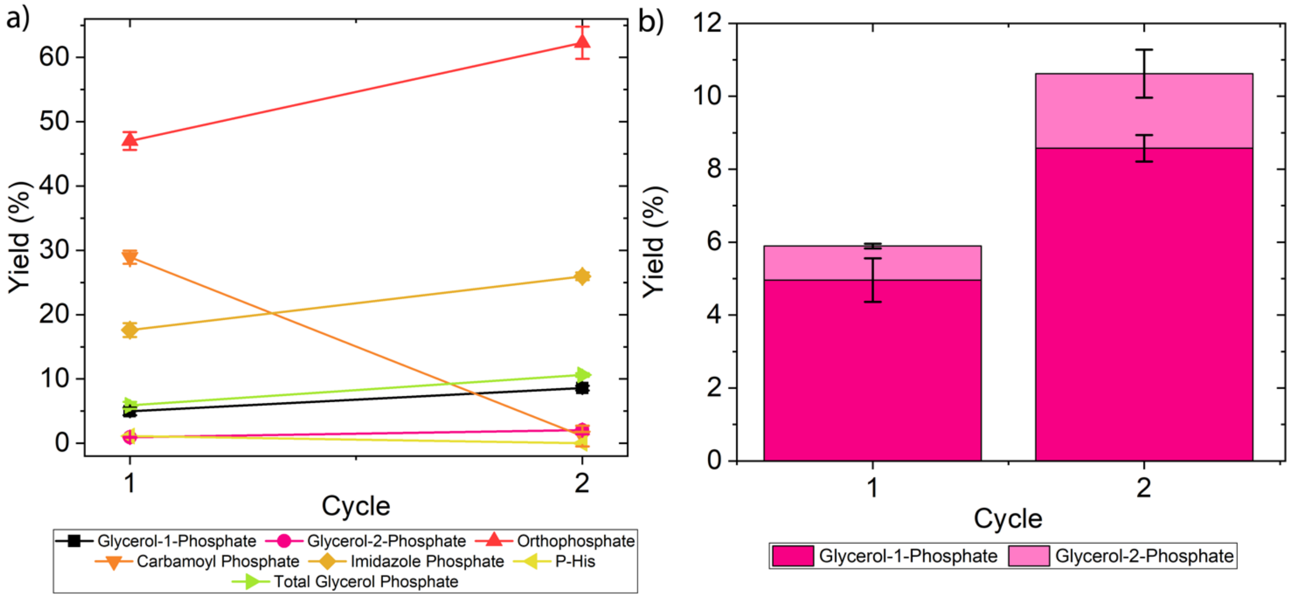

Supporting Figure 263: a) Changes in yield for all phosphate-containing products over two cycles for the reaction of 20 mM sodium phosphate dibasic + 500 mM glycerol + 230 mM potassium cyanate + 100 mM imidazole at pH 7.3 and 22 °C with 1 % weight/weight Hydroxyapatite. b) Change in yield for glycerol-1-phosphate and glycerol-2-phosphate over the course of two cycles.

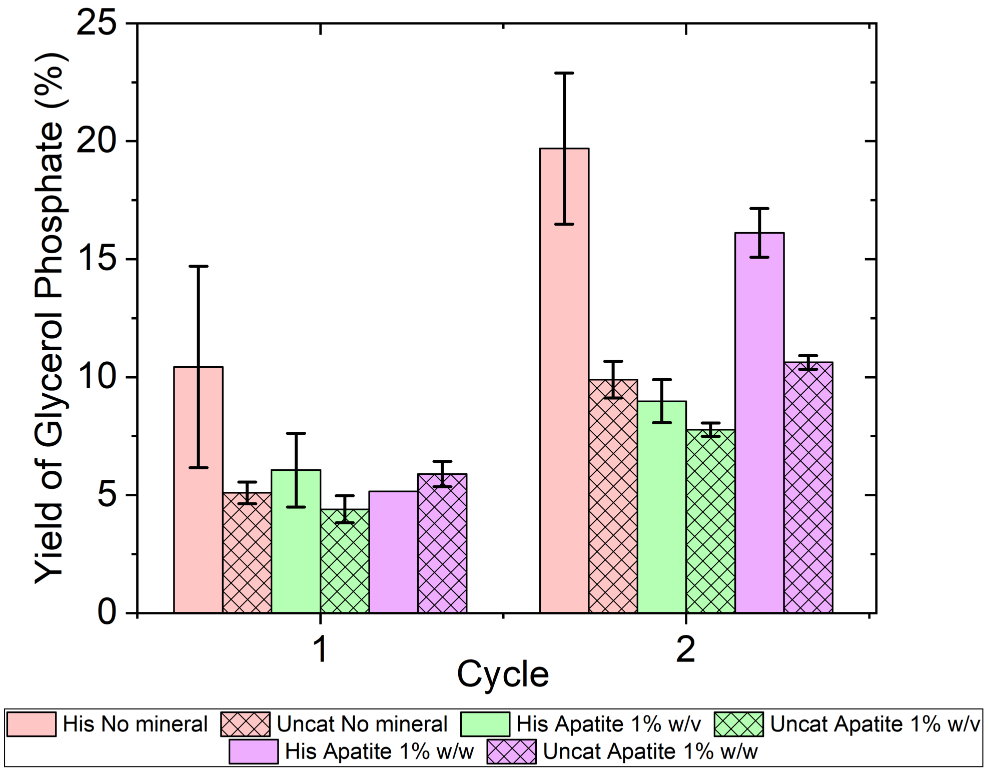

Supporting Figure 264: The effect of the minerals Hydroxyapatite on the uncatalysed and histidine-catalysed phosphorylation of glycerol in the physicochemical orthophosphate cycle. The changes in glycerol-phosphate yield (summation of the yields of glycerol-1-phosphate and glycerol-2-phosphate) over two cycles for the reaction of 50 mM histidine, 20 mM sodium phosphate dibasic + 500 mM glycerol + 230 mM potassium cyanate + 100 mM imidazole at pH 7.3 and 22 °C.

Supporting Figure 264 shows that in the presence of the mineral Hydroxyapatite the histidyl-catalysed phosphorylations still occur in the second cycle. In the first cycle, the histidyl catalysts do not show catalysis compared to the uncatalysed reaction. However, after the second cycle the histidyl catalysed reactions do show an increased yield of phosphorylated glycerol.

S5 *In situ* NMR Spectroscopic Characterisation of Phosphorylated Histidyls

S5.1 Phosphorylated Histidine Intermediate

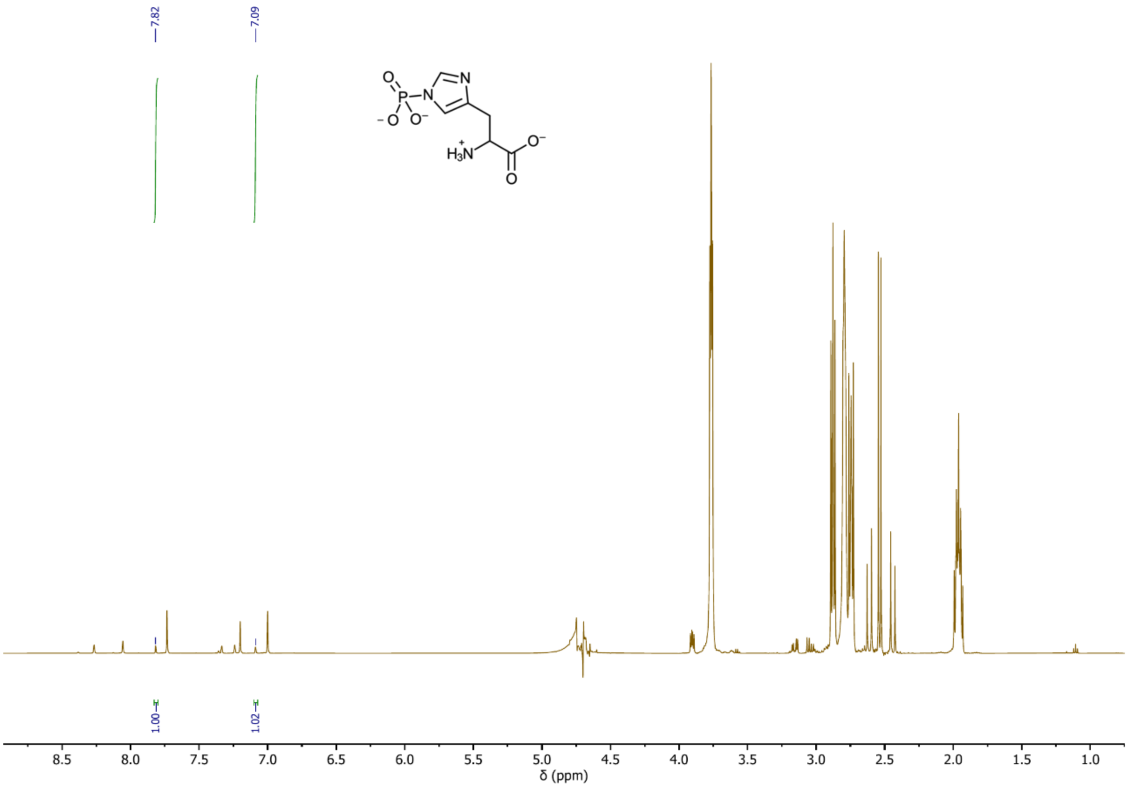

Supporting Figure 265: ^{1}H NMR spectrum of the phosphorylated histidine intermediate. Characterised *in situ* after 48 h starting from a solution of 50 mM histidine and 50 mM calcium imidazole phosphate in 0.5 M MOPS buffer at pH 7.5 in 9 : 1 H_2O : D_2O containing 0.1 M citric acid and 50 mM HMPA internal standard.

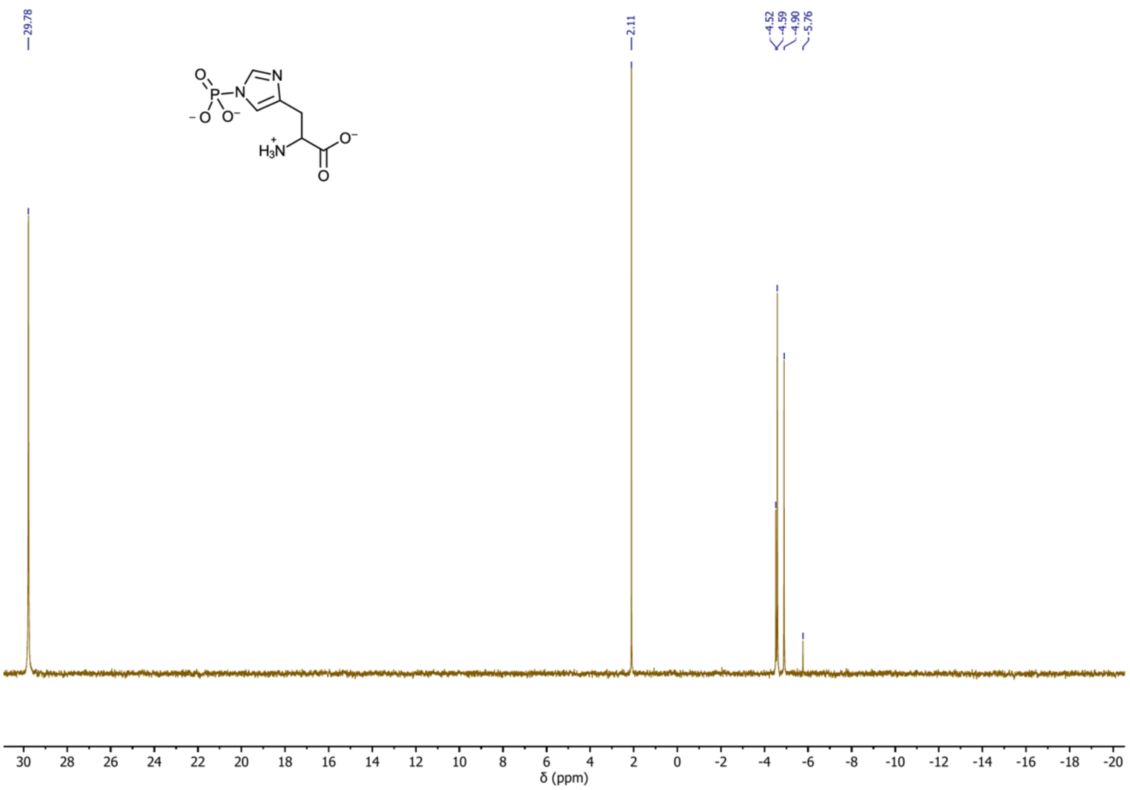

Supporting Figure 266: ^{31}P NMR spectrum of the phosphorylated histidine intermediate. Characterised *in situ* after 48 h starting from a solution of 50 mM histidine and 50 mM calcium imidazole phosphate in 0.5 M MOPS buffer at pH 7.5 in 9 : 1 H_2O : D_2O containing 0.1 M citric acid and 50 mM HMPA internal standard.

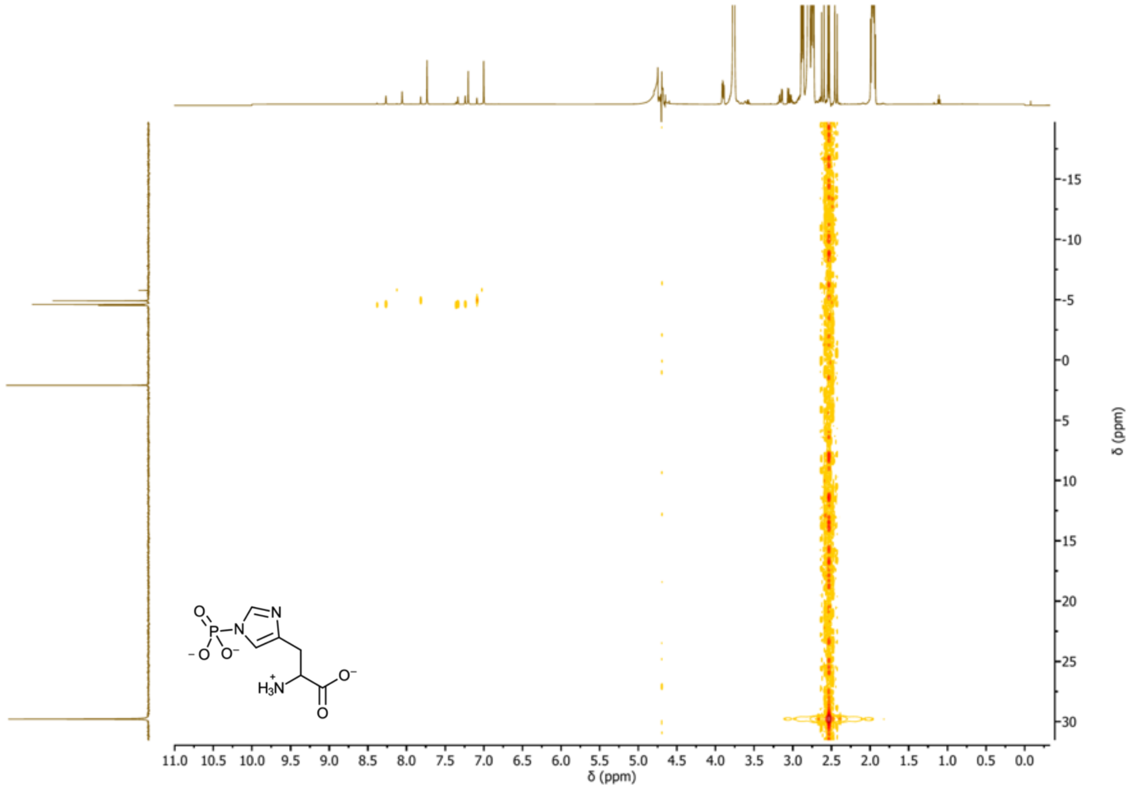

Supporting Figure 267: ^{1}H ^{31}P HMBC spectrum of the phosphorylated histidine intermediate. Characterised *in situ* after 48 h starting from a solution of 50 mM histidine and 50 mM calcium imidazole phosphate in 0.5 M MOPS buffer at pH 7.5 in 9 : 1 H_2O : D_2O containing 0.1 M citric acid and 50 mM HMPA internal standard.

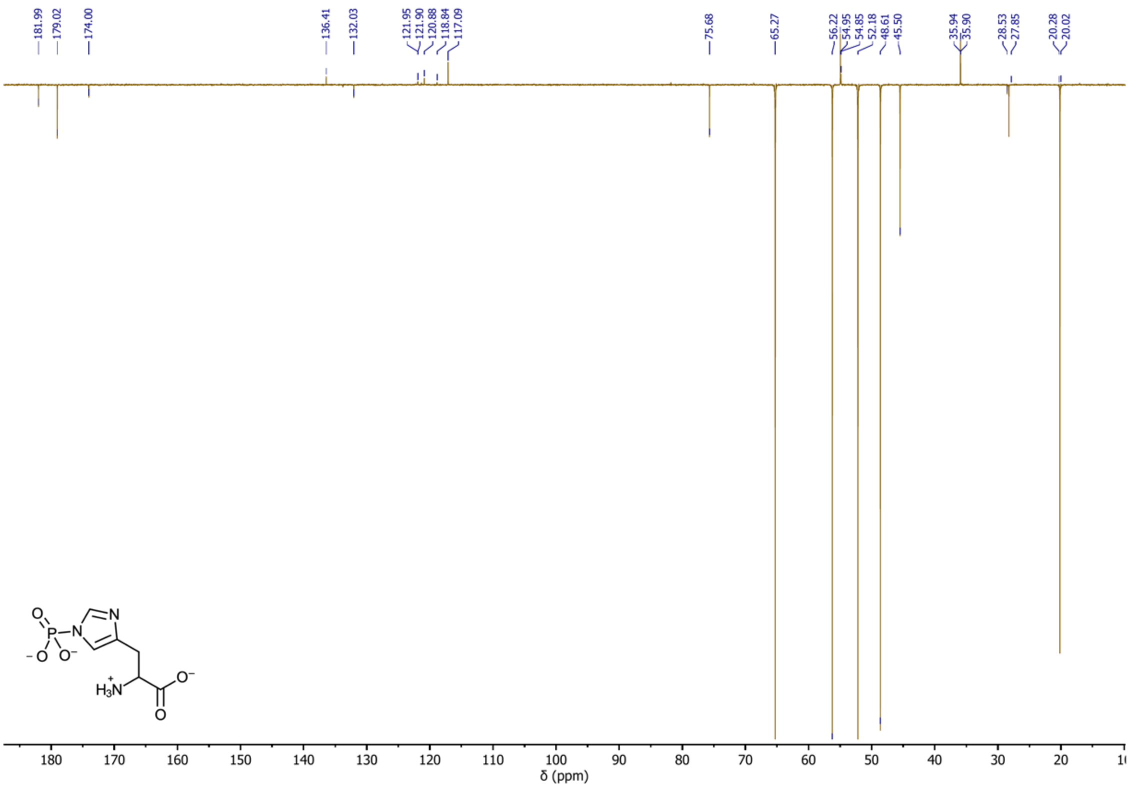

Supporting Figure 268: ^{13}C NMR spectrum of the phosphorylated histidine intermediate. Characterised *in situ* after 48 h starting from a solution of 50 mM histidine and 50 mM calcium imidazole phosphate in 0.5 M MOPS buffer at pH 7.5 in 9 : 1 H_2O : D_2O containing 0.1 M citric acid and 50 mM HMPA internal standard.

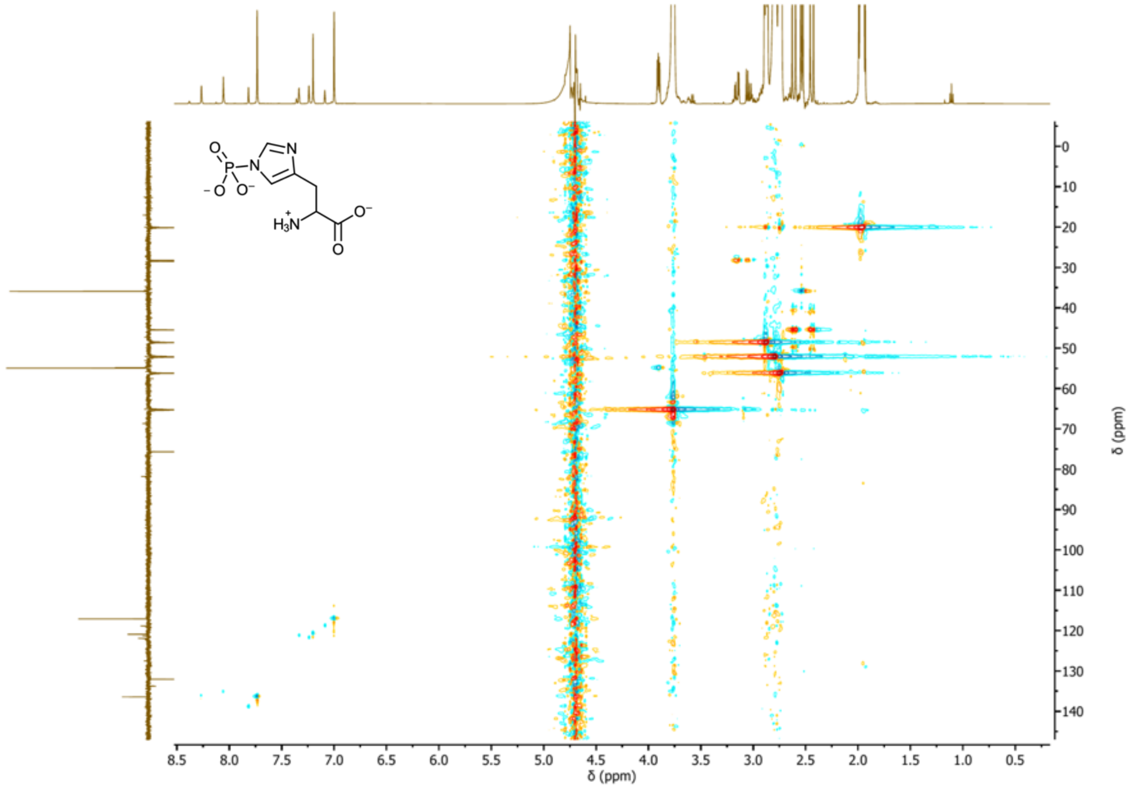

Supporting Figure 269: ^{1}H ^{13}C HSQC spectrum of the phosphorylated histidine intermediate. Characterised *in situ* after 48 h starting from a solution of 50 mM histidine and 50 mM calcium imidazole phosphate in 0.5 M MOPS buffer at pH 7.5 in 9 : 1 H_2O : D_2O containing 0.1 M citric acid and 50 mM HMPA internal standard.

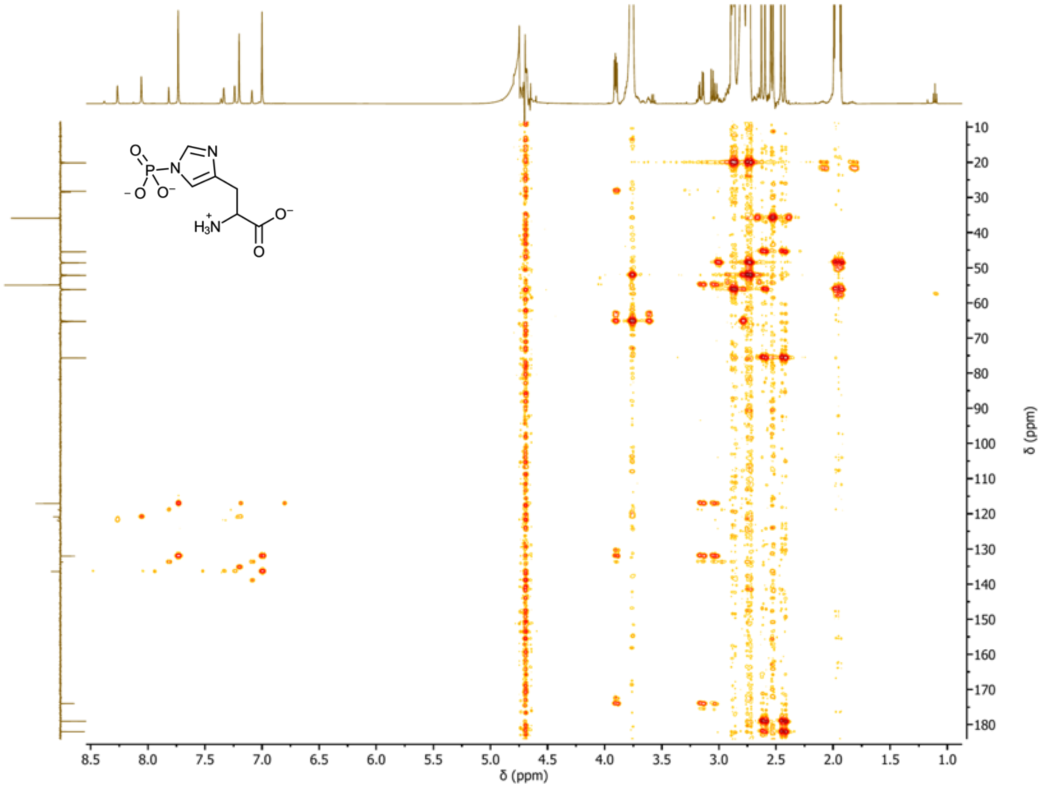

Supporting Figure 270: ^{1}H ^{13}C HMBC spectrum of the phosphorylated histidine intermediate. Characterised *in situ* after 48 h starting from a solution of 50 mM histidine and 50 mM calcium imidazole phosphate in 0.5 M MOPS buffer at pH 7.5 in 9 : 1 H_2O : D_2O containing 0.1 M citric acid and 50 mM HMPA internal standard.

S5.2 Phosphorylated Acetyl-Histidine Intermediate

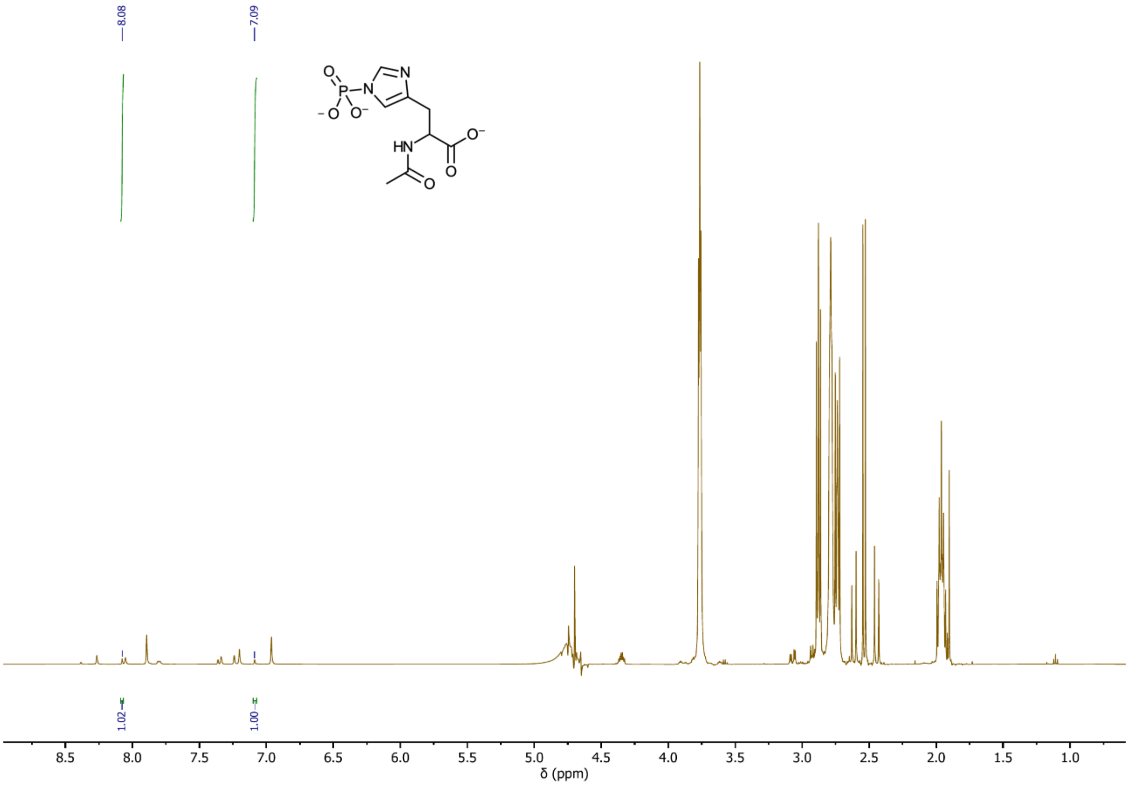

Supporting Figure 271: ^{1}H NMR spectrum of the phosphorylated acetyl histidine intermediate. Characterised *in situ* after 48 h starting from a solution of 50 mM acetyl histidine and 50 mM calcium imidazole phosphate in 0.5 M MOPS buffer at pH 7.5 in 9 : 1 H_2O : D_2O containing 0.1 M citric acid and 50 mM HMPA internal standard.

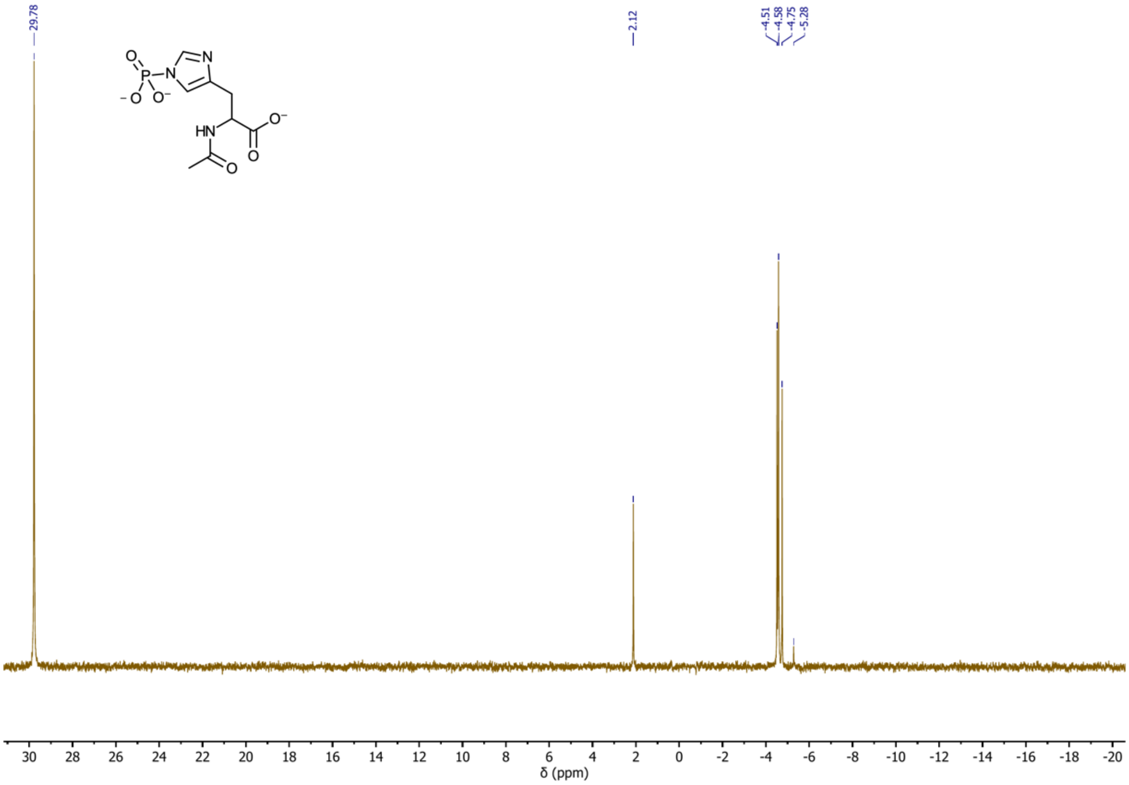

Supporting Figure 272: ^{31}P NMR spectrum of the phosphorylated acetyl histidine intermediate. Characterised *in situ* after 48 h starting from a solution of 50 mM acetyl histidine and 50 mM calcium imidazole phosphate in 0.5 M MOPS buffer at pH 7.5 in 9 : 1 H_2O : D_2O containing 0.1 M citric acid and 50 mM HMPA internal standard.

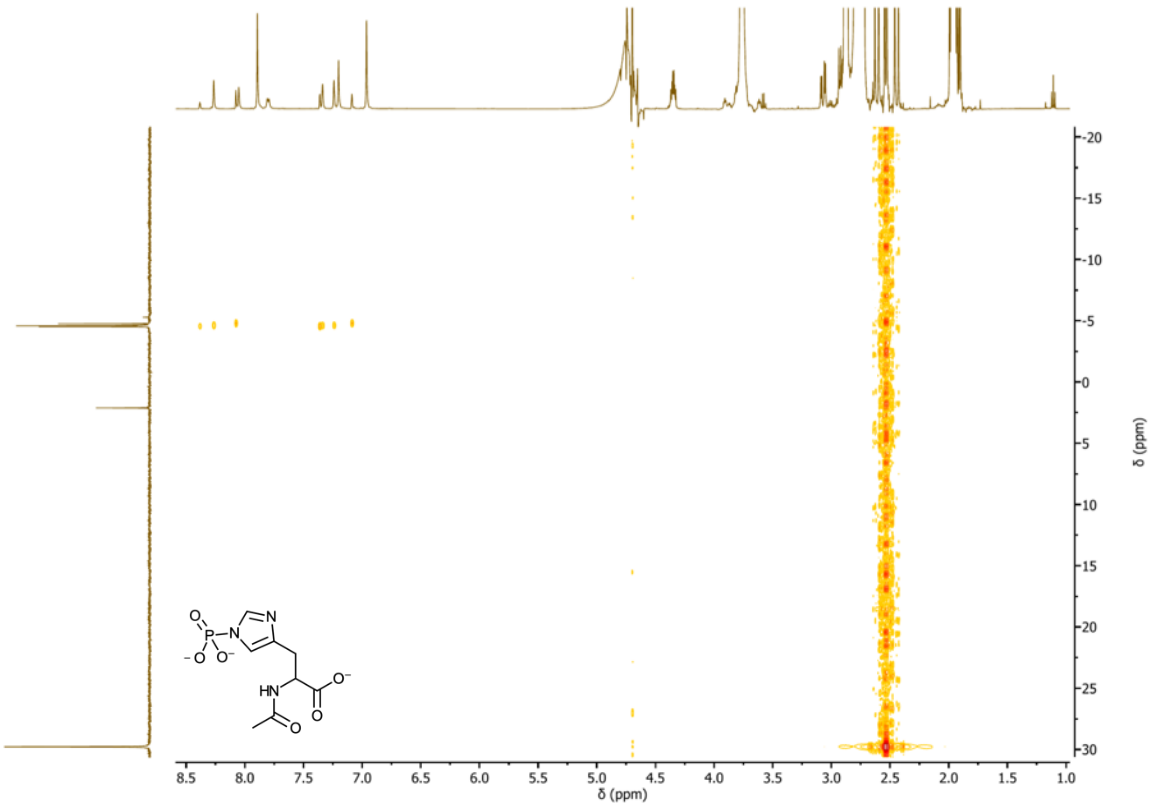

Supporting Figure 273: ^{1}H ^{31}P HMBC spectrum of the phosphorylated acetyl histidine intermediate. Characterised *in situ* after 48 h starting from a solution of 50 mM acetyl histidine and 50 mM calcium imidazole phosphate in 0.5 M MOPS buffer at pH 7.5 in 9 : 1 H_2O : D_2O containing 0.1 M citric acid and 50 mM HMPA internal standard.

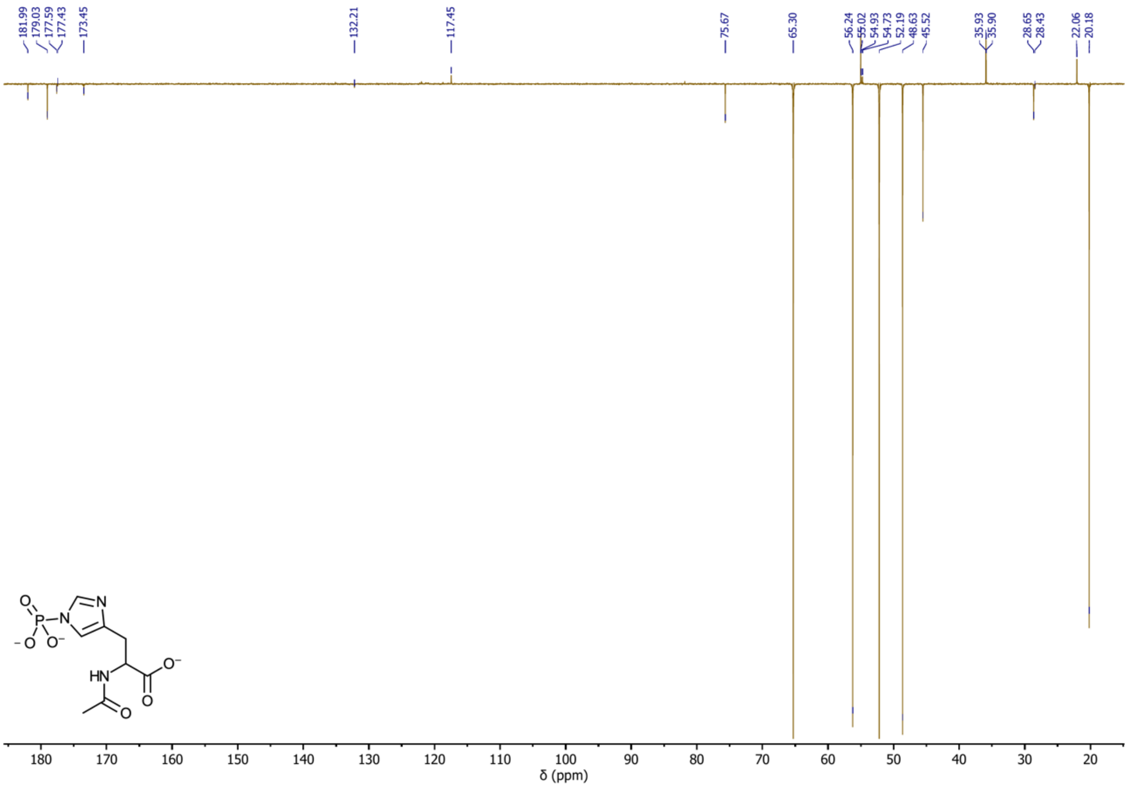

Supporting Figure 274: ^{13}C NMR spectrum of the phosphorylated acetyl histidine intermediate. Characterised *in situ* after 48 h starting from a solution of 50 mM acetyl histidine and 50 mM calcium imidazole phosphate in 0.5 M MOPS buffer at pH 7.5 in 9 : 1 H_2O : D_2O containing 0.1 M citric acid and 50 mM HMPA internal standard.

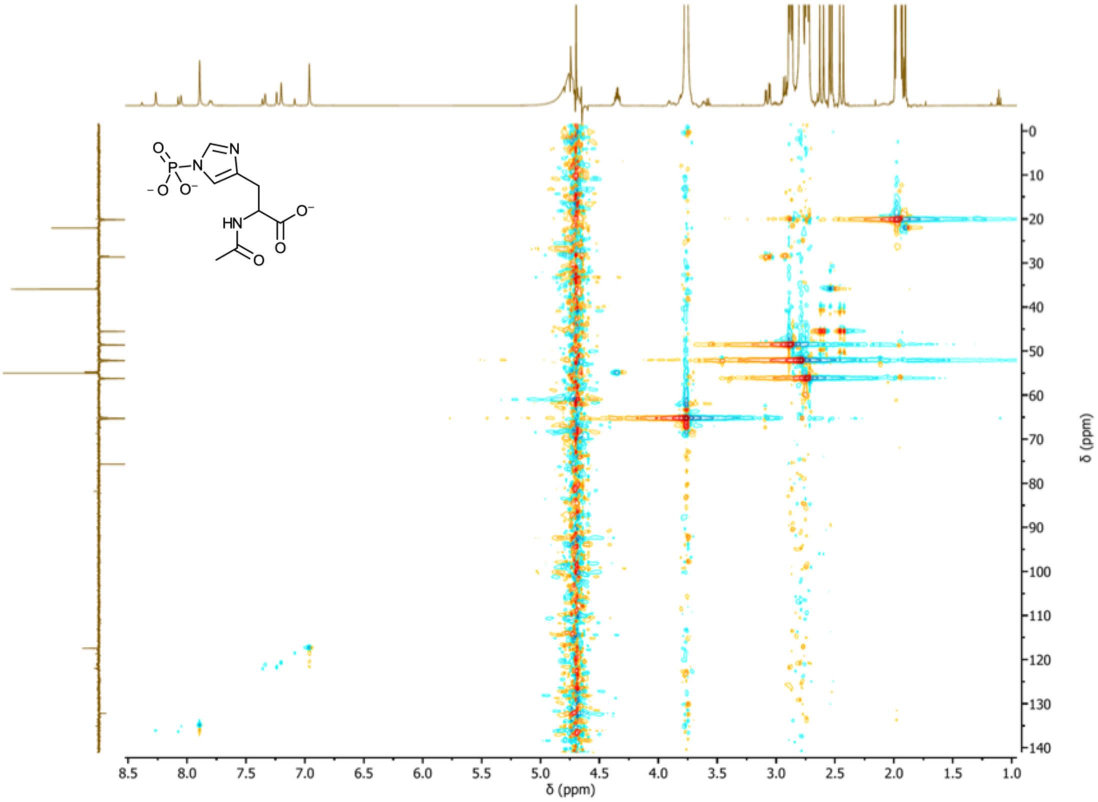

Supporting Figure 275: ^{1}H ^{13}C HSQC spectrum of the phosphorylated acetyl histidine intermediate. Characterised *in situ* after 48 h starting from a solution of 50 mM acetyl histidine and 50 mM calcium imidazole phosphate in 0.5 M MOPS buffer at pH 7.5 in 9 : 1 H_2O : D_2O containing 0.1 M citric acid and 50 mM HMPA internal standard.

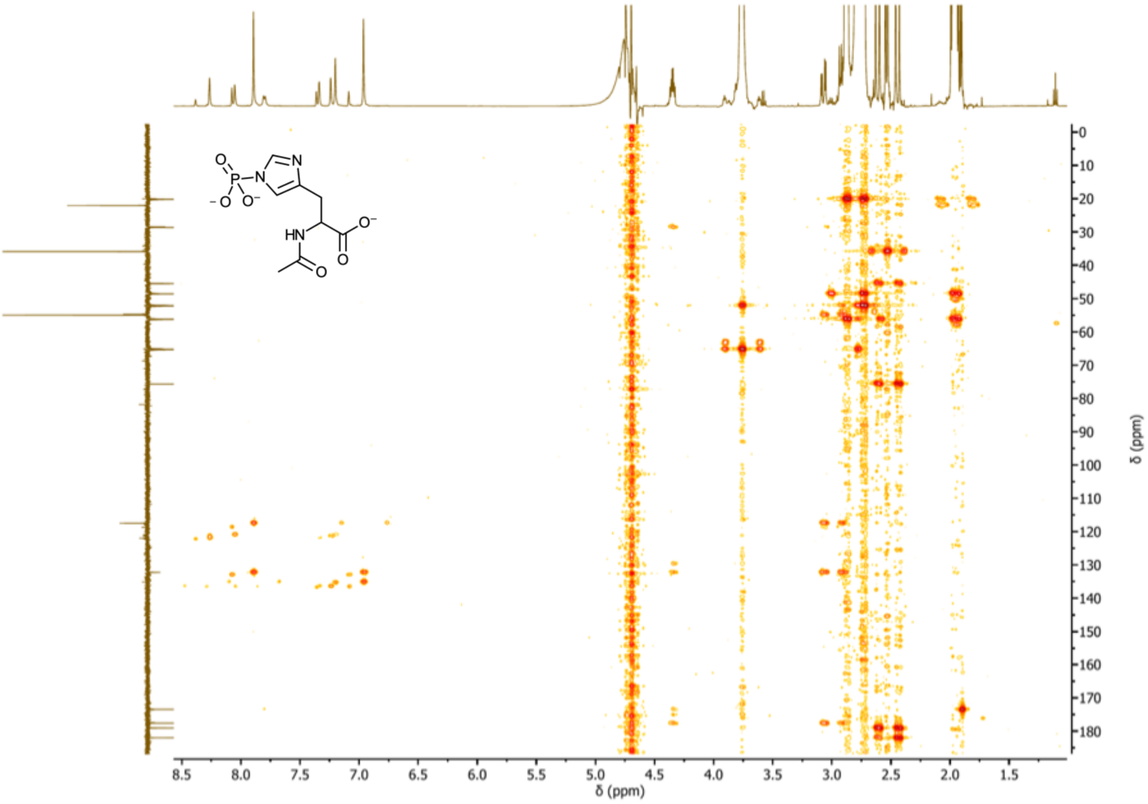

Supporting Figure 276: ^{1}H ^{13}C HMBC spectrum of the phosphorylated acetyl histidine intermediate. Characterised *in situ* after 48 h starting from a solution of 50 mM acetyl histidine and 50 mM calcium imidazole phosphate in 0.5 M MOPS buffer at pH 7.5 in 9 : 1 H_2O : D_2O containing 0.1 M citric acid and 50 mM HMPA internal standard.

S5.3 Phosphorylated His-Asp Intermediate

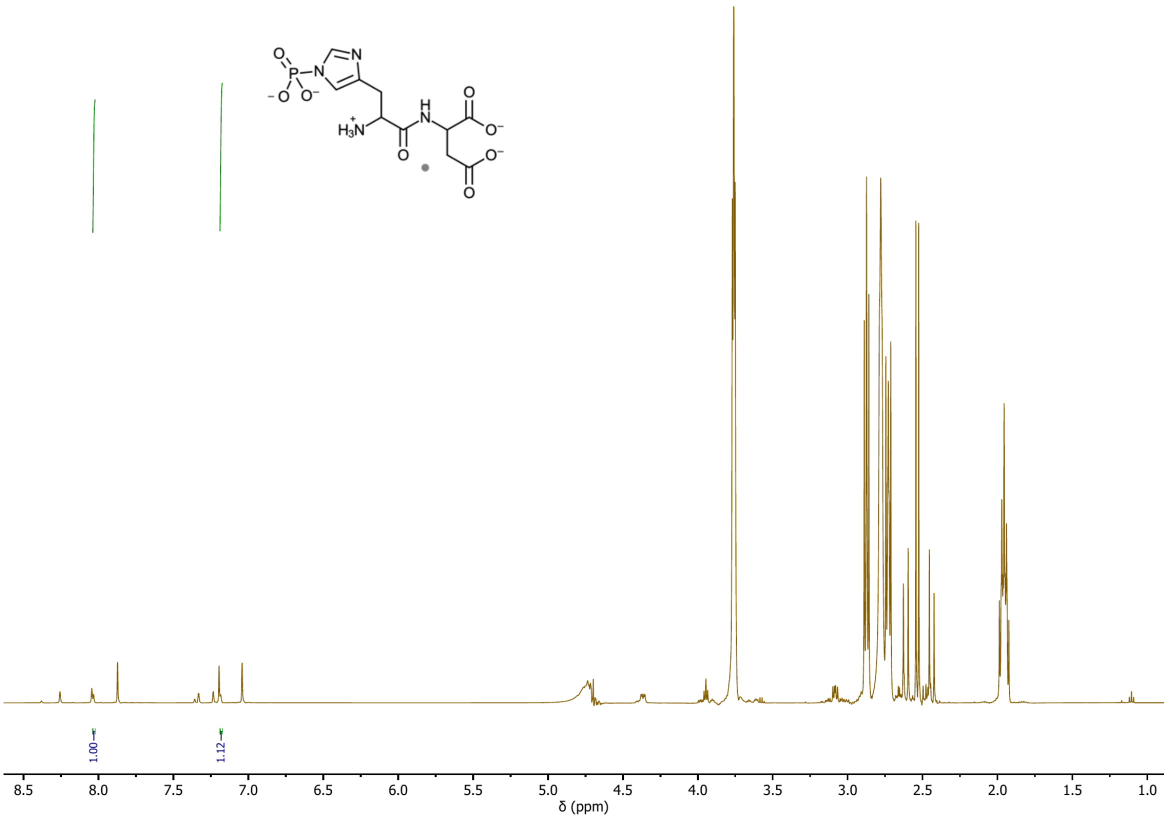

Supporting Figure 277: ^{1}H NMR spectrum of the phosphorylated His-Asp intermediate. Characterised *in situ* after 48 h starting from a solution of 50 mM His-Asp and 50 mM calcium imidazole phosphate in 0.5 M MOPS buffer at pH 7.5 in 9 : 1 H_2O : D_2O containing 0.1 M citric acid and 50 mM HMPA internal standard.

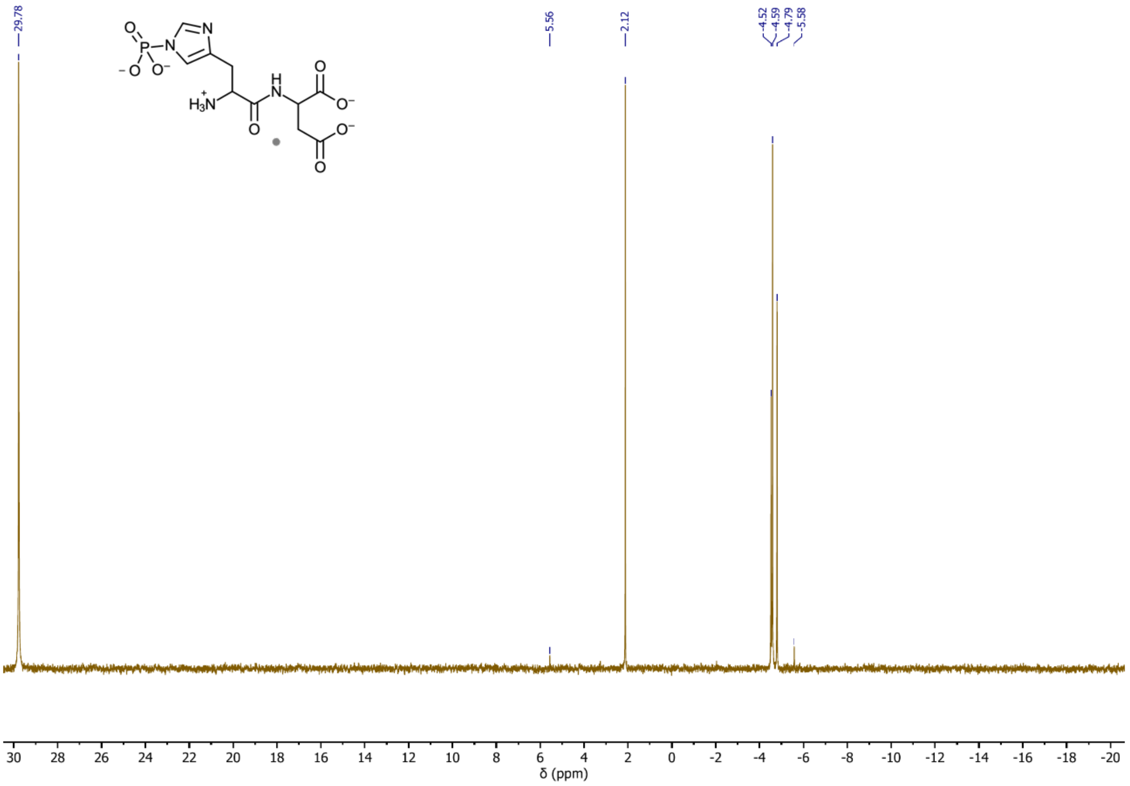

Supporting Figure 278: ^{31}P NMR spectrum of the phosphorylated His-Asp intermediate. Characterised *in situ* after 48 h starting from a solution of 50 mM His-Asp and 50 mM calcium imidazole phosphate in 0.5 M MOPS buffer at pH 7.5 in 9 : 1 H_2O : D_2O containing 0.1 M citric acid and 50 mM HMPA internal standard.

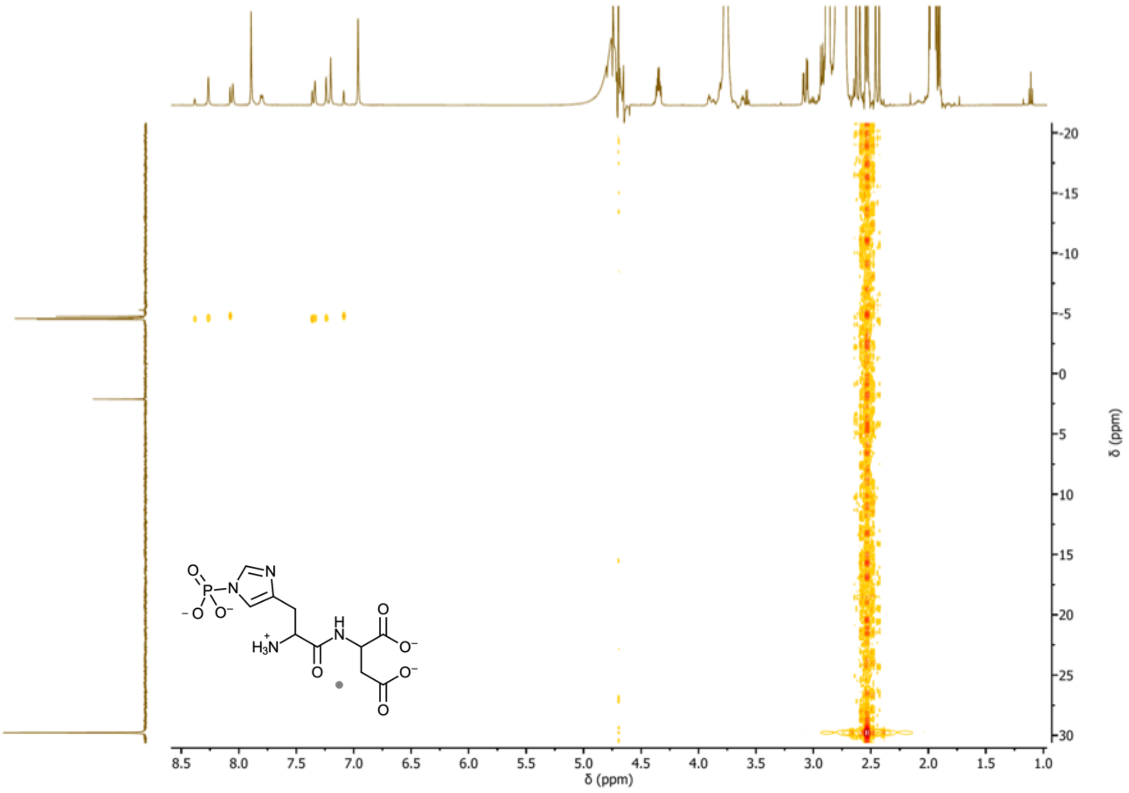

Supporting Figure 279: ^{1}H ^{31}P HMBC spectrum of the phosphorylated His-Asp intermediate. Characterised *in situ* after 48 h starting from a solution of 50 mM His-Asp and 50 mM calcium imidazole phosphate in 0.5 M MOPS buffer at pH 7.5 in 9 : 1 H_2O : D_2O containing 0.1 M citric acid and 50 mM HMPA internal standard.

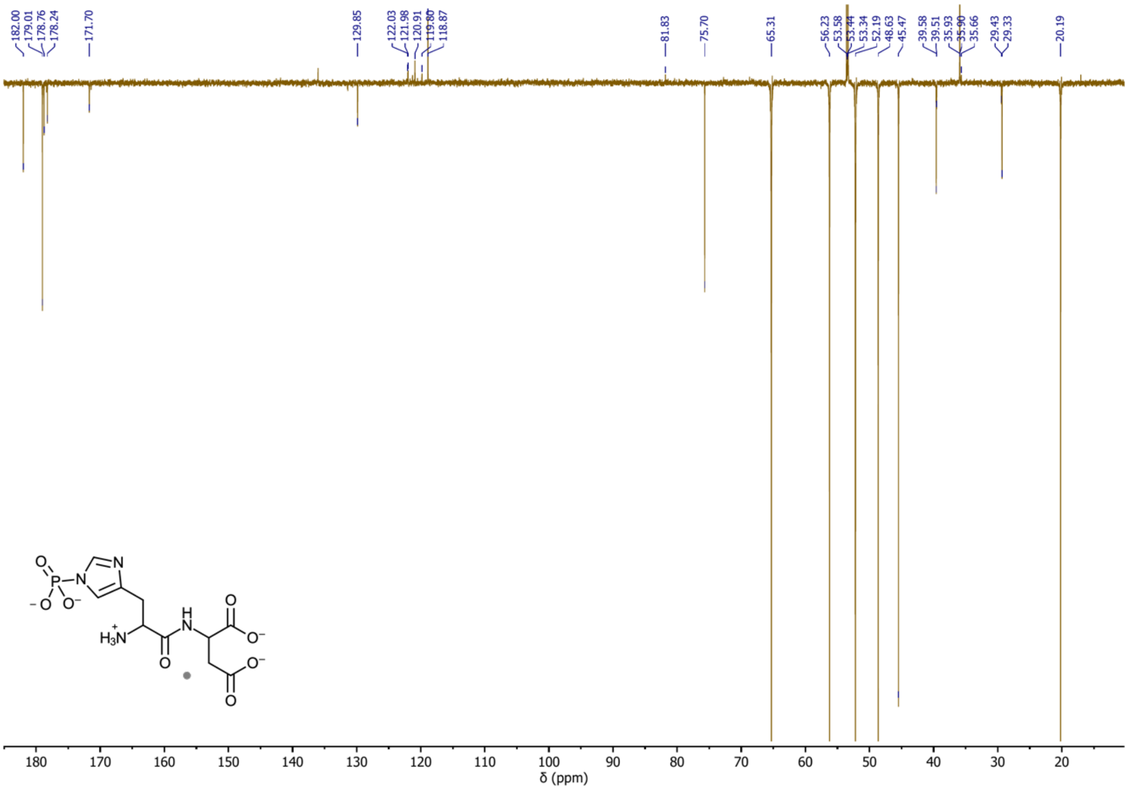

Supporting Figure 280: ^{13}C NMR spectrum of the phosphorylated His-Asp intermediate. Characterised *in situ* after 48 h starting from a solution of 50 mM His-Asp and 50 mM calcium imidazole phosphate in 0.5 M MOPS buffer at pH 7.5 in 9 : 1 H_2O : D_2O containing 0.1 M citric acid and 50 mM HMPA internal standard.

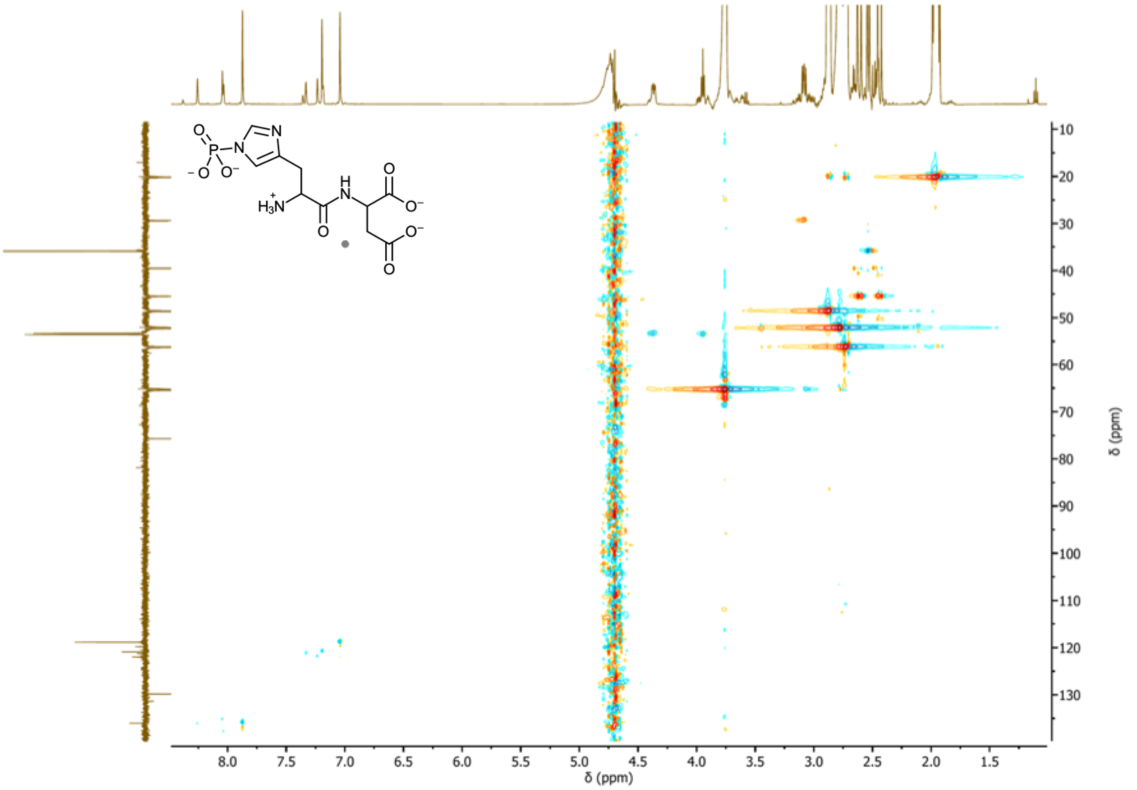

Supporting Figure 281: ^{1}H ^{13}C HSQC spectrum of the phosphorylated His-Asp intermediate. Characterised *in situ* after 48 h starting from a solution of 50 mM His-Asp and 50 mM calcium imidazole phosphate in 0.5 M MOPS buffer at pH 7.5 in 9 : 1 H_2O : D_2O containing 0.1 M citric acid and 50 mM HMPA internal standard.

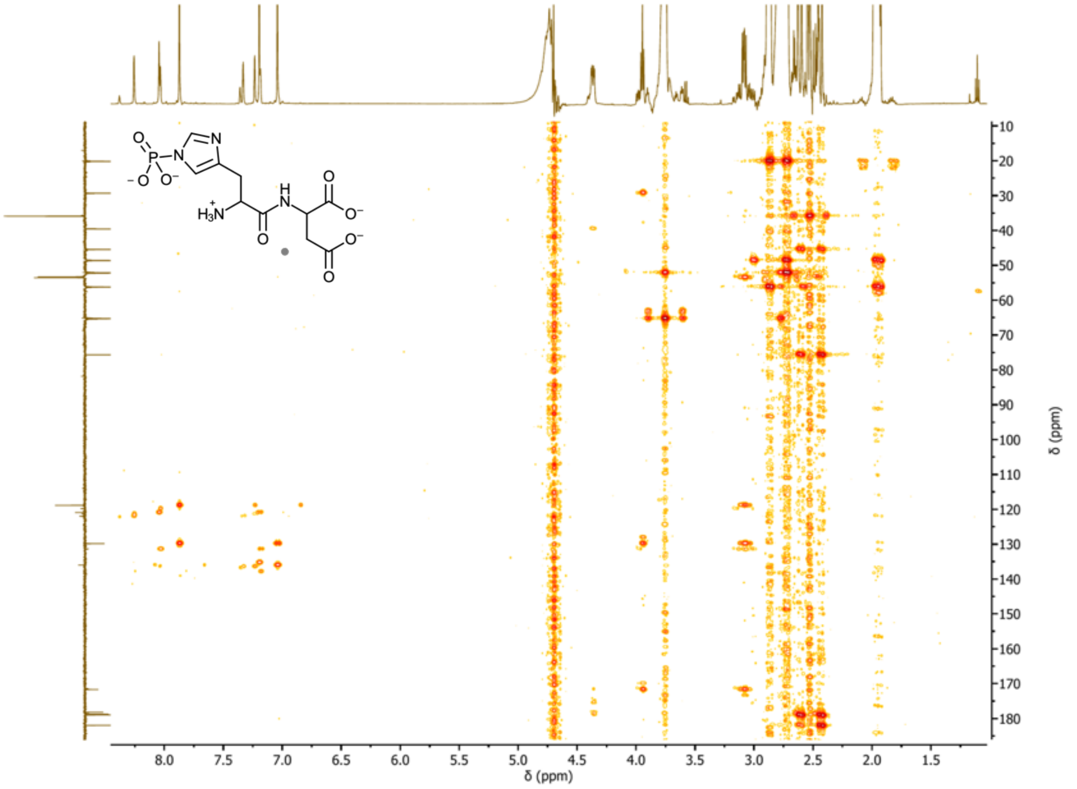

Supporting Figure 282: ^{1}H ^{13}C HMBC spectrum of the phosphorylated His-Asp intermediate. Characterised *in situ* after 48 h starting from a solution of 50 mM His-Asp and 50 mM calcium imidazole phosphate in 0.5 M MOPS buffer at pH 7.5 in 9 : 1 H_2O : D_2O containing 0.1 M citric acid and 50 mM HMPA internal standard.

S5.4 Phosphorylated His-Lys Intermediate

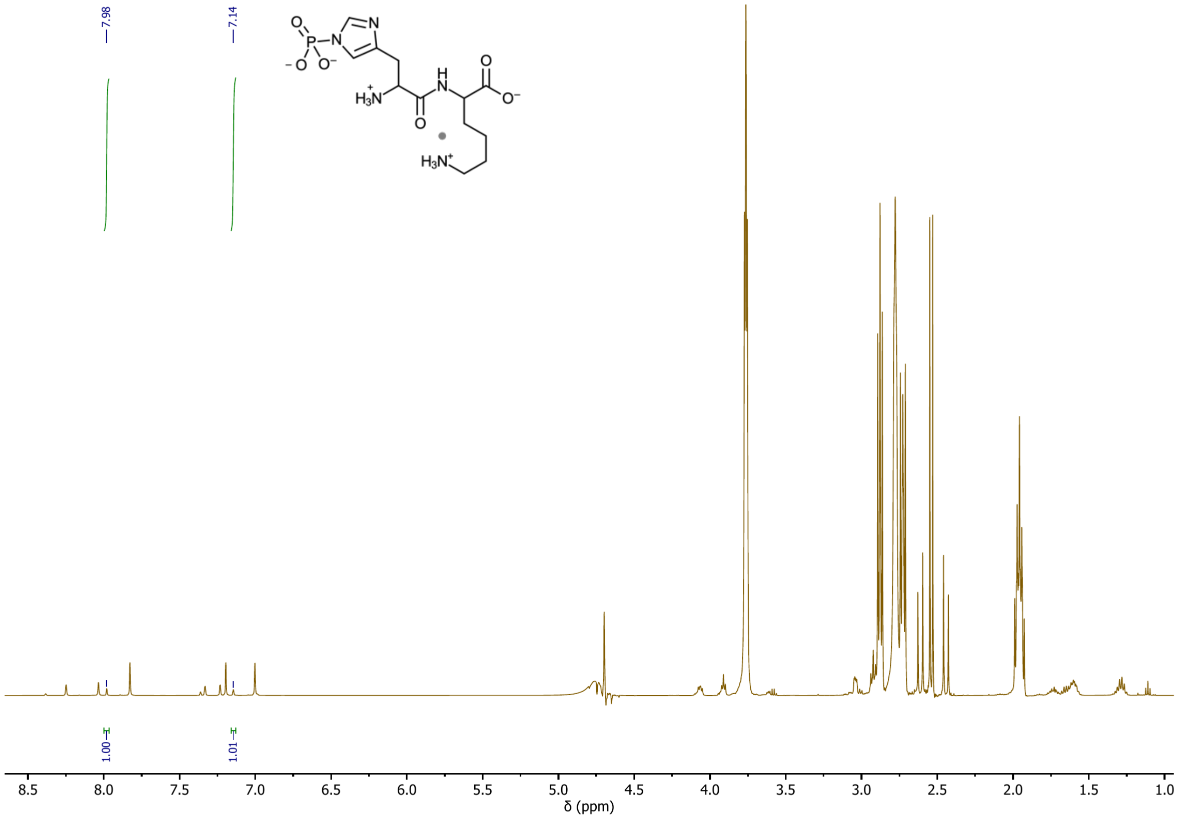

Supporting Figure 283: ^{1}H NMR spectrum of the phosphorylated His-Lys intermediate. Characterised *in situ* after 48 h starting from a solution of 50 mM His-Lys and 50 mM calcium imidazole phosphate in 0.5 M MOPS buffer at pH 7.5 in 9 : 1 H_2O : D_2O containing 0.1 M citric acid and 50 mM HMPA internal standard.

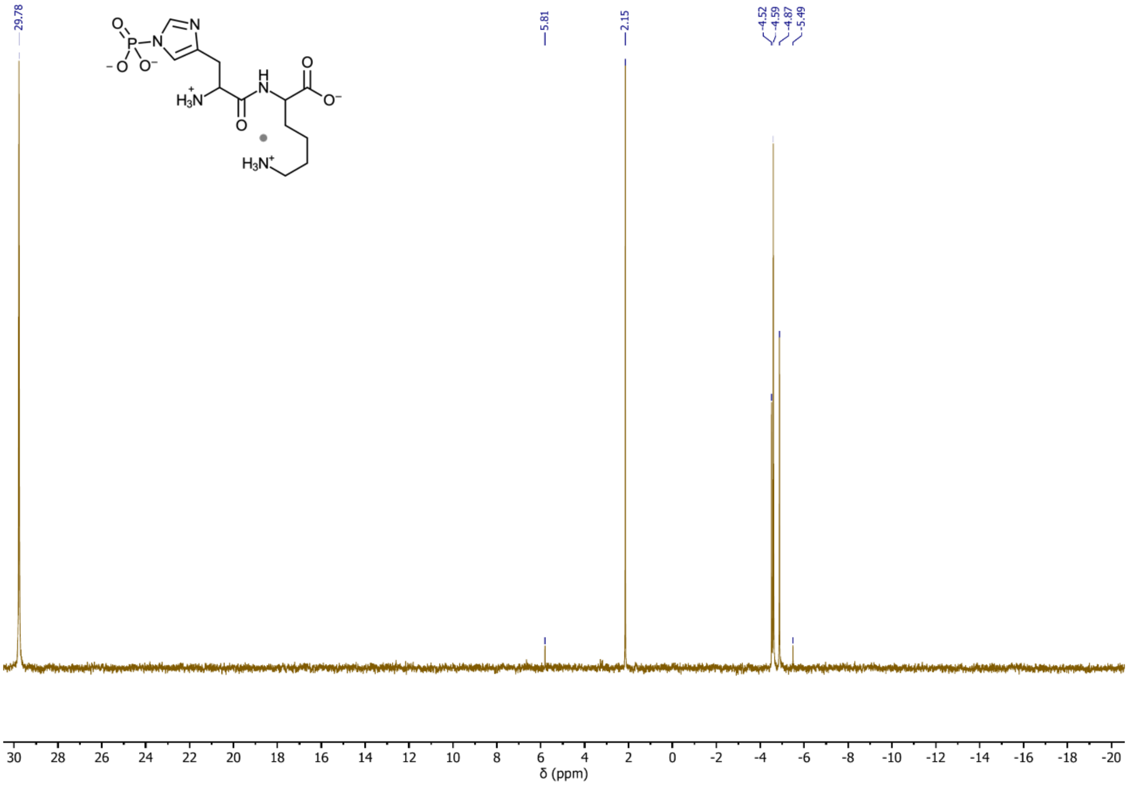

Supporting Figure 284: ^{31}P NMR spectrum of the phosphorylated His-Lys intermediate. Characterised *in situ* after 48 h starting from a solution of 50 mM His-Lys and 50 mM calcium imidazole phosphate in 0.5 M MOPS buffer at pH 7.5 in 9 : 1 H_2O : D_2O containing 0.1 M citric acid and 50 mM HMPA internal standard.

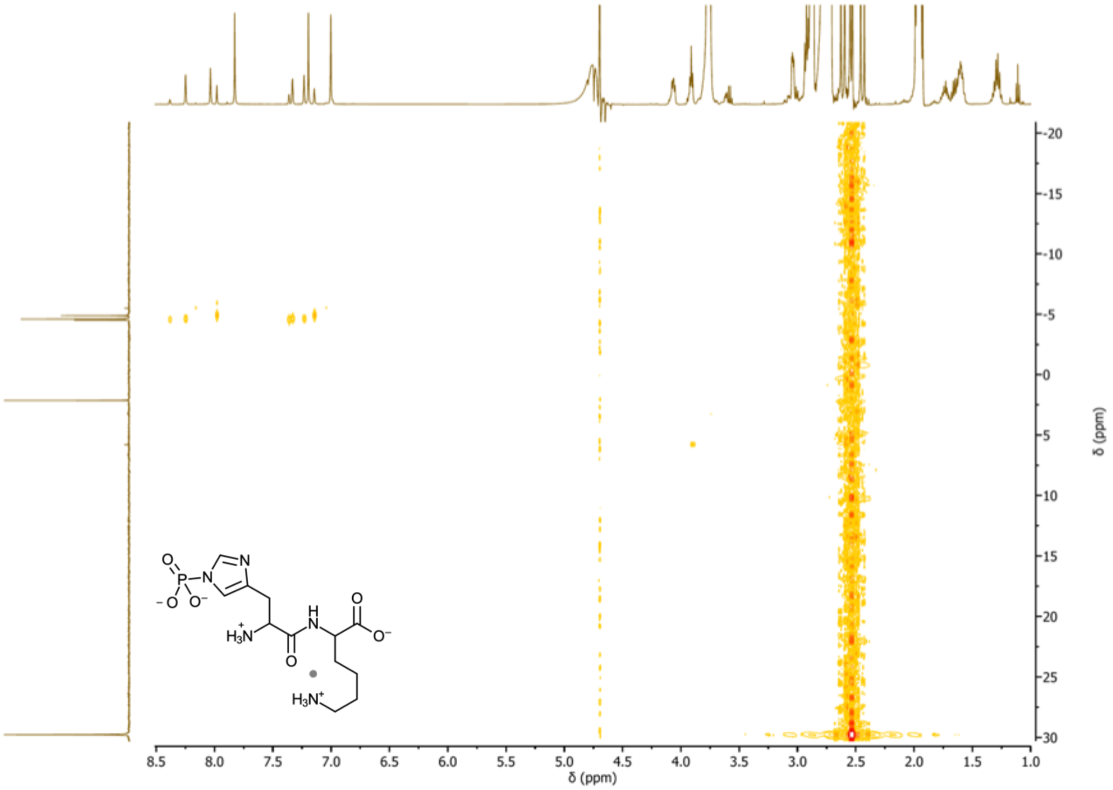

Supporting Figure 285: ^{1}H ^{31}P HMBC spectrum of the phosphorylated His-Lys intermediate. Characterised *in situ* after 48 h starting from a solution of 50 mM His-Lys and 50 mM calcium imidazole phosphate in 0.5 M MOPS buffer at pH 7.5 in 9 : 1 H_2O : D_2O containing 0.1 M citric acid and 50 mM HMPA internal standard.

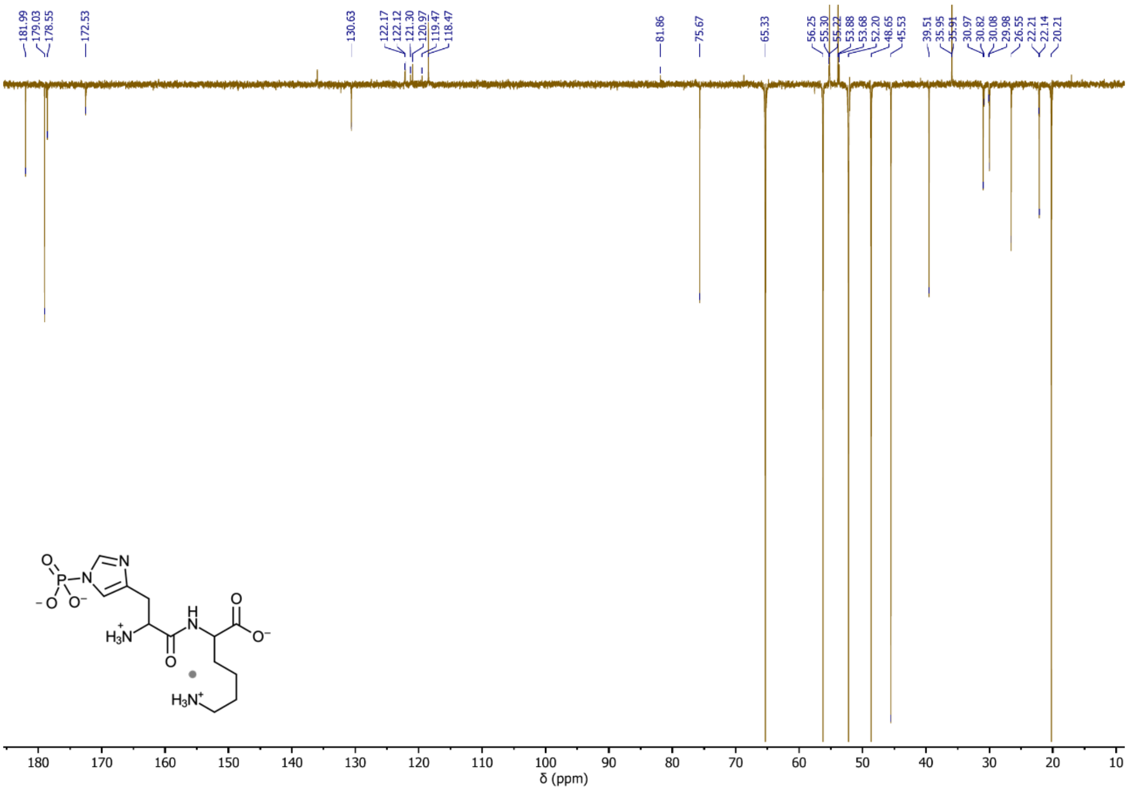

Supporting Figure 286: ^{13}C NMR spectrum of the phosphorylated His-Lys intermediate. Characterised *in situ* after 48 h starting from a solution of 50 mM His-Lys and 50 mM calcium imidazole phosphate in 0.5 M MOPS buffer at pH 7.5 in 9 : 1 H_2O : D_2O containing 0.1 M citric acid and 50 mM HMPA internal standard.

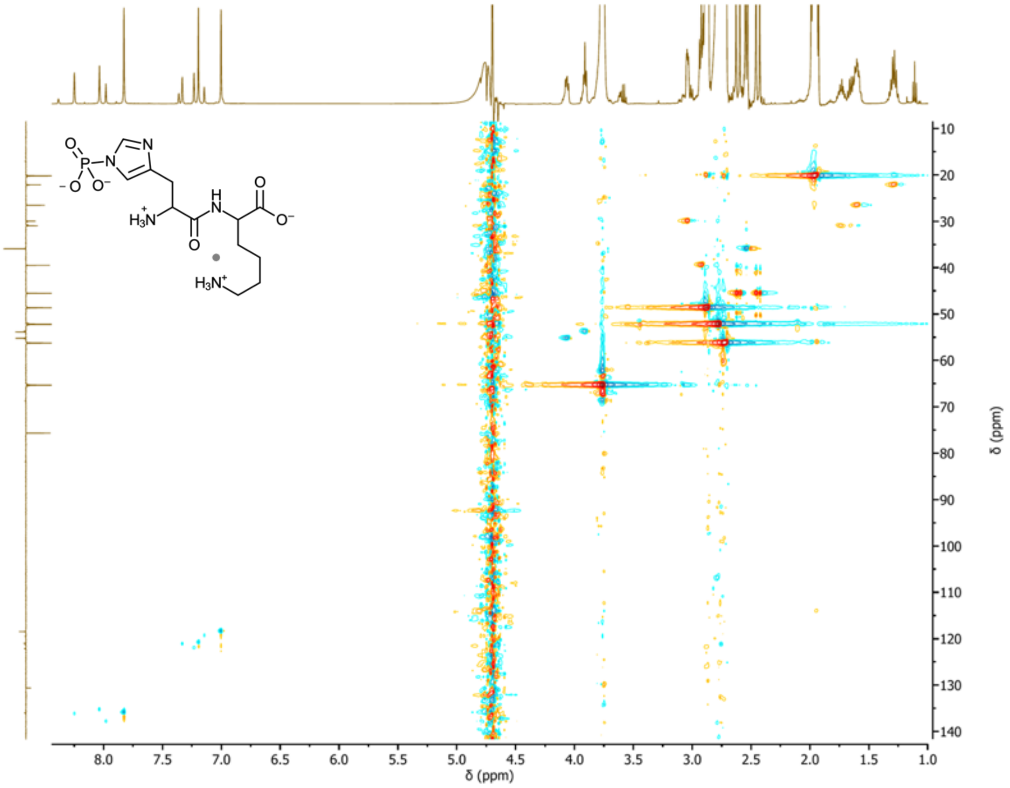

Supporting Figure 287: ^{1}H ^{13}C HSQC spectrum of the phosphorylated His-Lys intermediate. Characterised *in situ* after 48 h starting from a solution of 50 mM His-Lys and 50 mM calcium imidazole phosphate in 0.5 M MOPS buffer at pH 7.5 in 9 : 1 H_2O : D_2O containing 0.1 M citric acid and 50 mM HMPA internal standard.

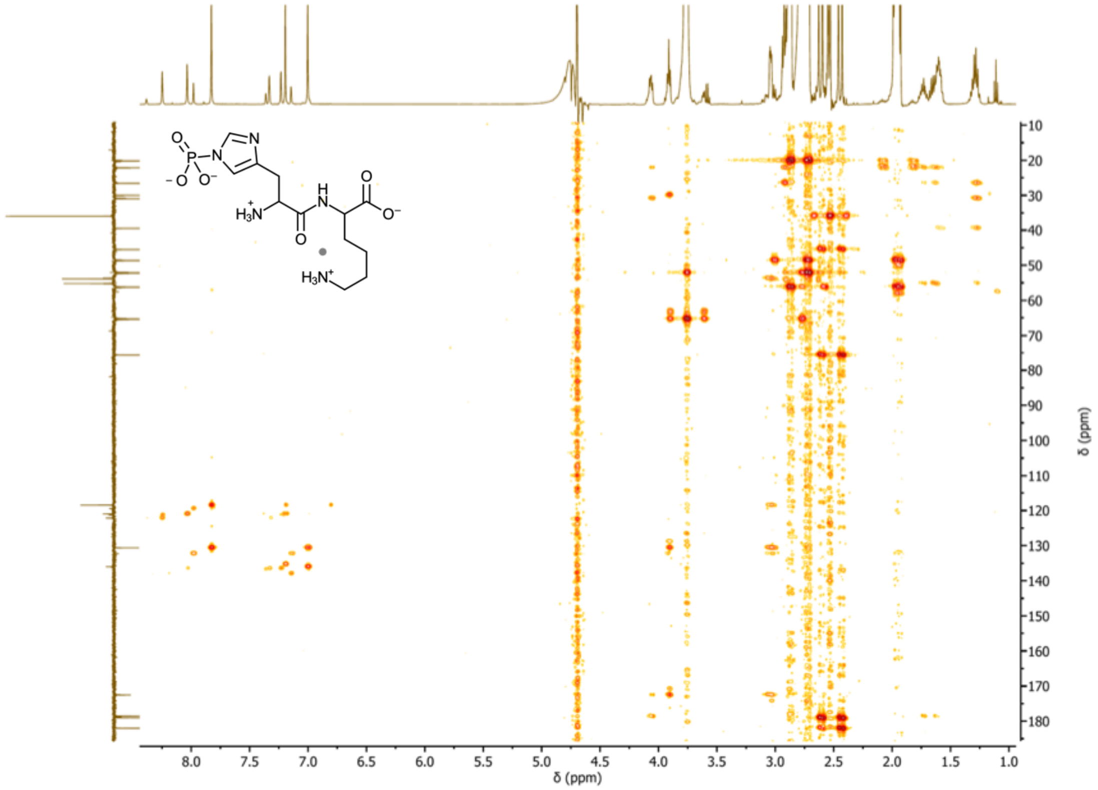

Supporting Figure 288: ^{1}H ^{13}C HMBC spectrum of the phosphorylated His-Lys intermediate. Characterised *in situ* after 48 h starting from a solution of 50 mM His-Lys and 50 mM calcium imidazole phosphate in 0.5 M MOPS buffer at pH 7.5 in 9 : 1 H_2O : D_2O containing 0.1 M citric acid and 50 mM HMPA internal standard.

S5.5 Phosphorylated His-Gly-Gly Intermediate

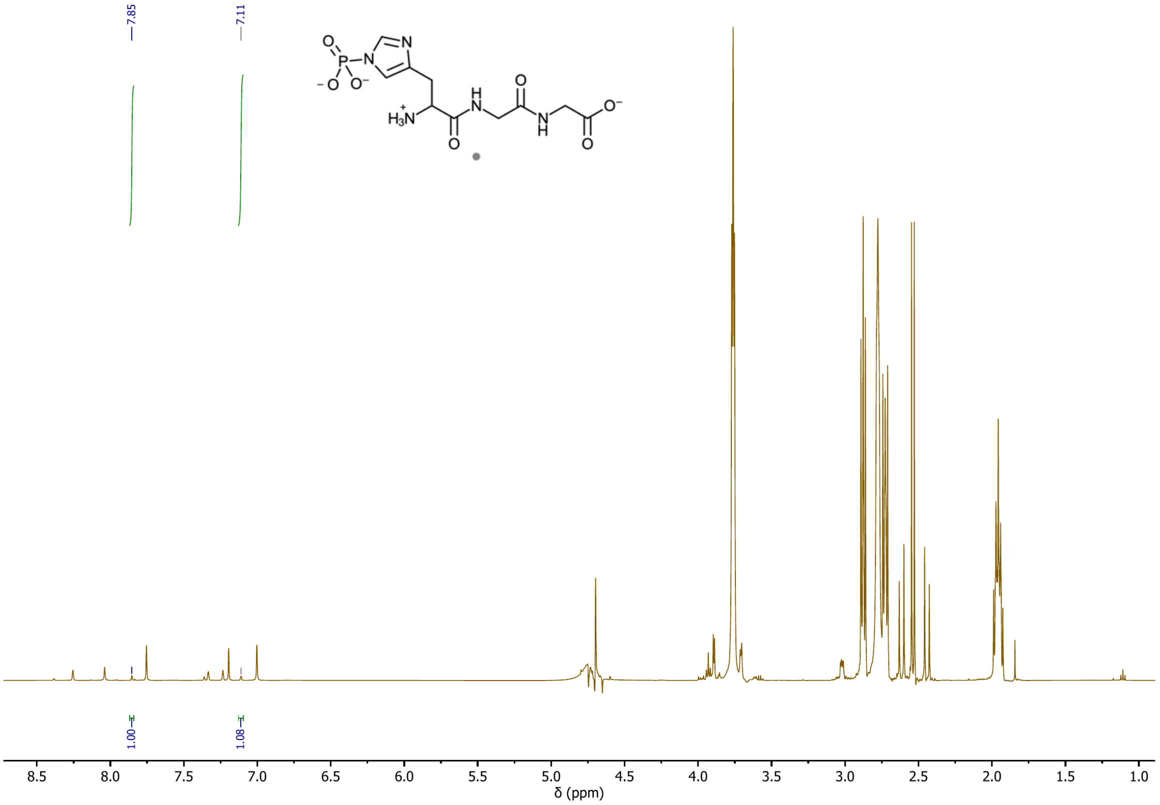

Supporting Figure 289: ^{1}H NMR spectrum of the phosphorylated His-Gly-Gly intermediate. Characterised *in situ* after 48 h starting from a solution of 50 mM His-Gly-Gly and 50 mM calcium imidazole phosphate in 0.5 M MOPS buffer at pH 7.5 in 9 : 1 H_2O : D_2O containing 0.1 M citric acid and 50 mM HMPA internal standard.

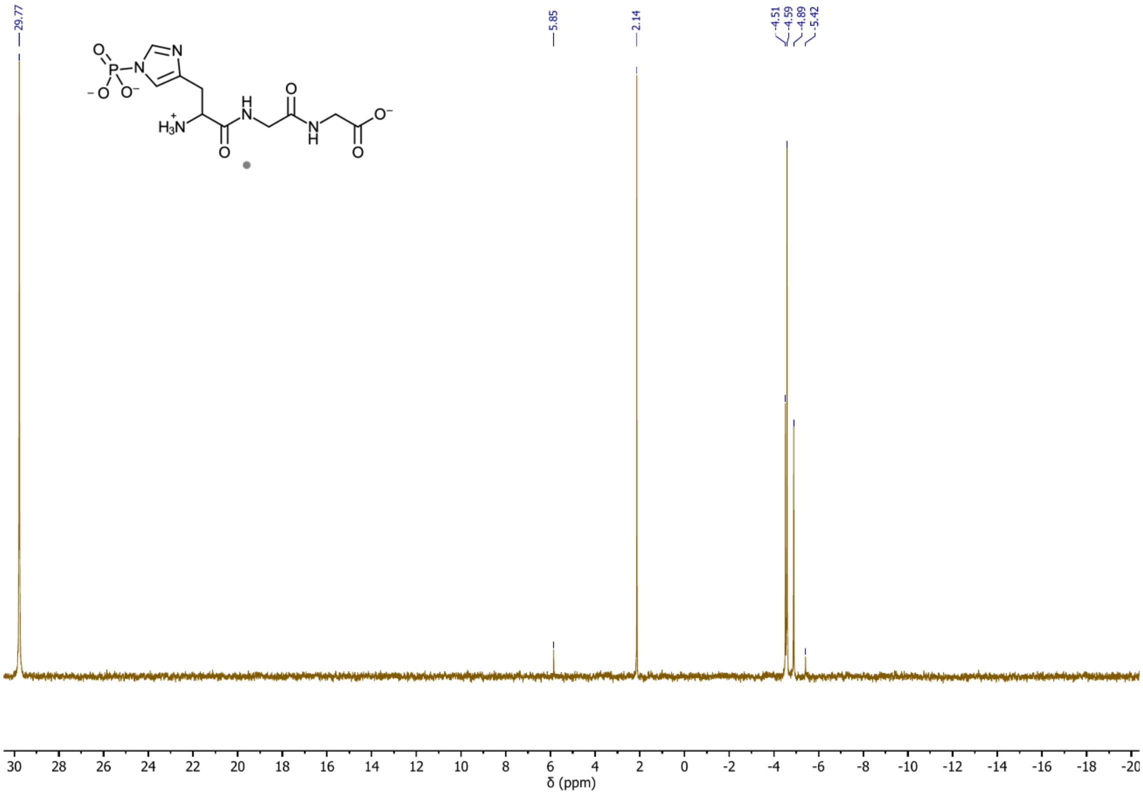

Supporting Figure 290: ^{31}P NMR spectrum of the phosphorylated His-Gly-Gly intermediate. Characterised *in situ* after 48 h starting from a solution of 50 mM His-Gly-Gly and 50 mM calcium imidazole phosphate in 0.5 M MOPS buffer at pH 7.5 in 9 : 1 H_2O : D_2O containing 0.1 M citric acid and 50 mM HMPA internal standard.

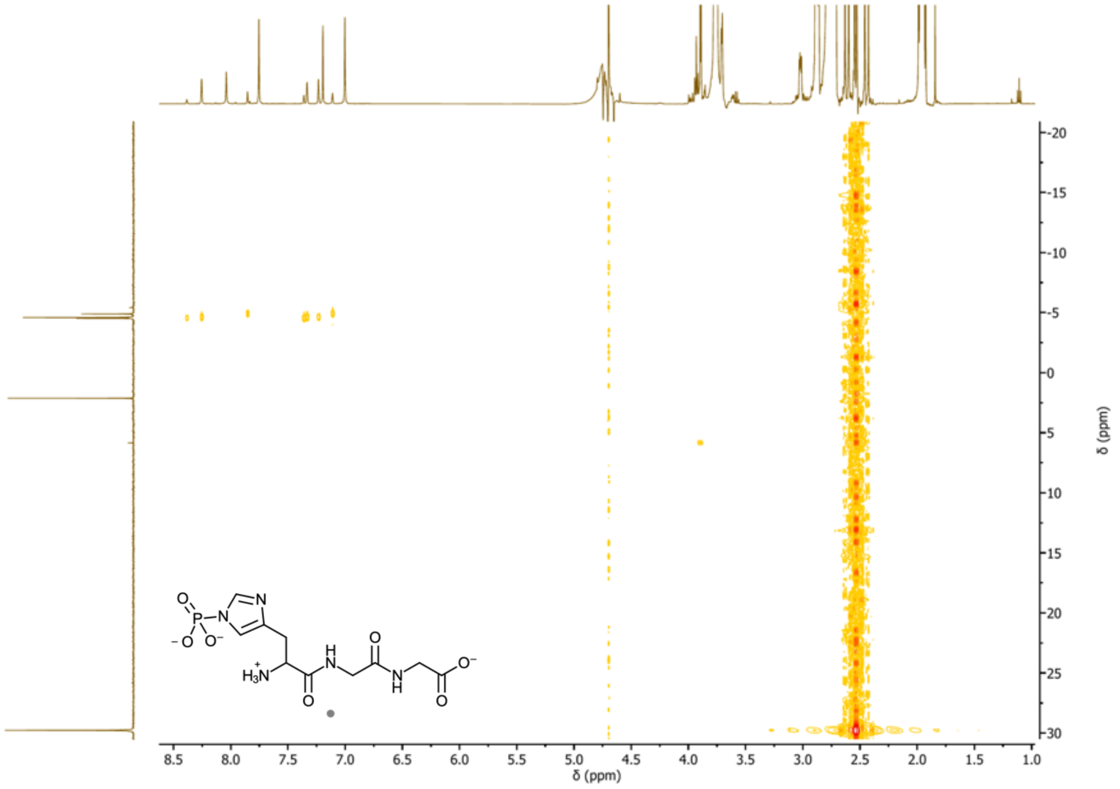

Supporting Figure 291: ^{1}H ^{31}P HMBC spectrum of the phosphorylated His-Gly-Gly intermediate. Characterised *in situ* after 48 h starting from a solution of 50 mM His-Gly-Gly and 50 mM calcium imidazole phosphate in 0.5 M MOPS buffer at pH 7.5 in 9 : 1 H_2O : D_2O containing 0.1 M citric acid and 50 mM HMPA internal standard.

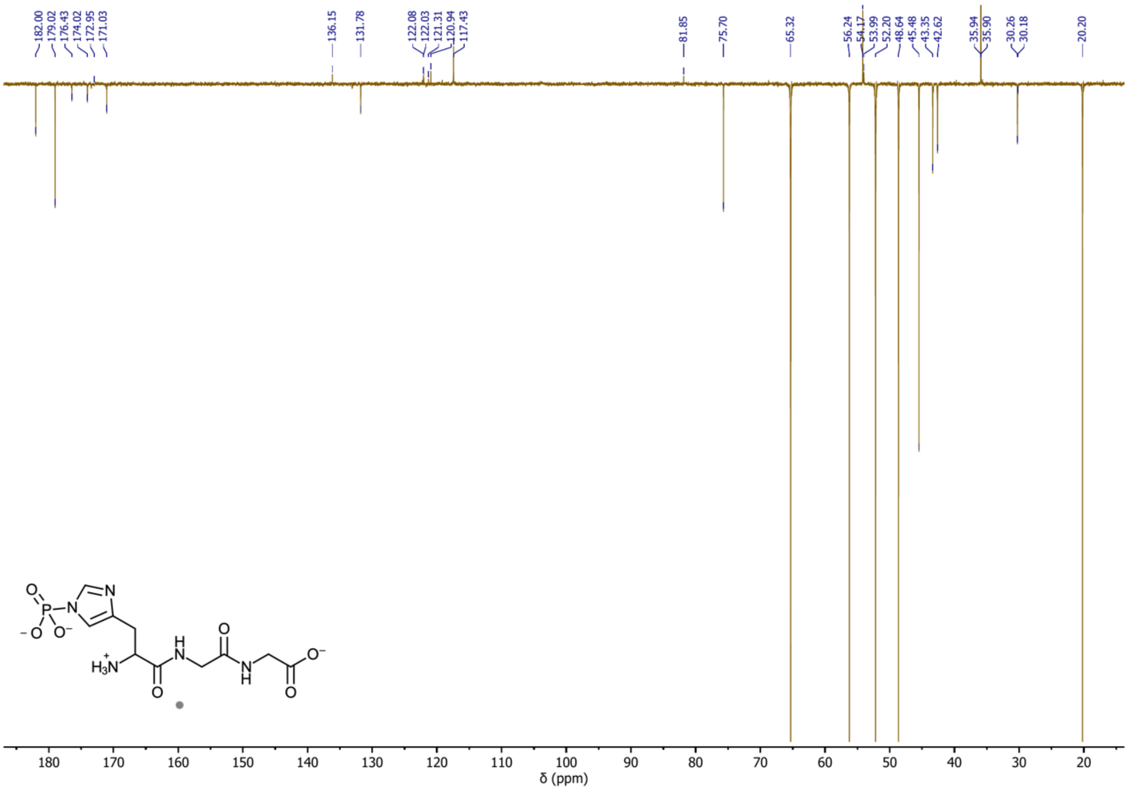

Supporting Figure 292: ^{13}C NMR spectrum of the phosphorylated His-Gly-Gly intermediate. Characterised *in situ* after 48 h starting from a solution of 50 mM His-Gly-Gly and 50 mM calcium imidazole phosphate in 0.5 M MOPS buffer at pH 7.5 in 9 : 1 H_2O : D_2O containing 0.1 M citric acid and 50 mM HMPA internal standard.

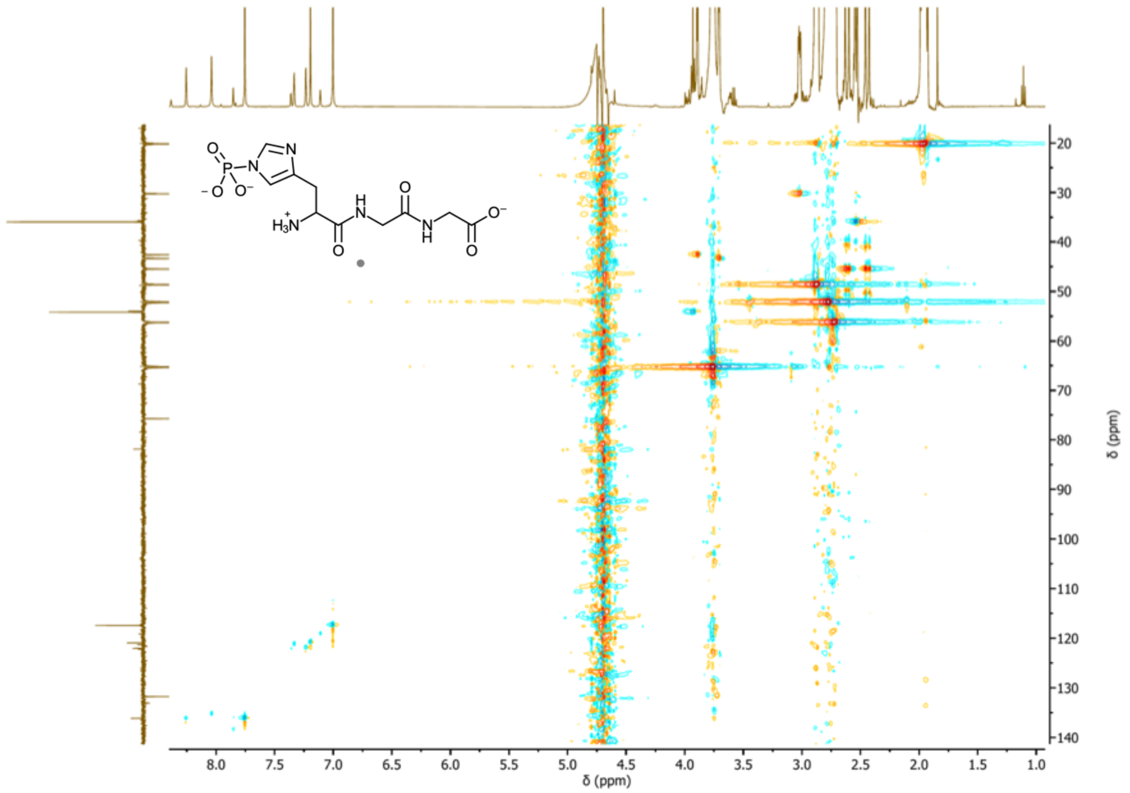

Supporting Figure 293: ^{1}H ^{13}C HSQC spectrum of the phosphorylated His-Gly-Gly intermediate. Characterised *in situ* after 48 h starting from a solution of 50 mM His-Gly-Gly and 50 mM calcium imidazole phosphate in 0.5 M MOPS buffer at pH 7.5 in 9 : 1 H_2O : D_2O containing 0.1 M citric acid and 50 mM HMPA internal standard.

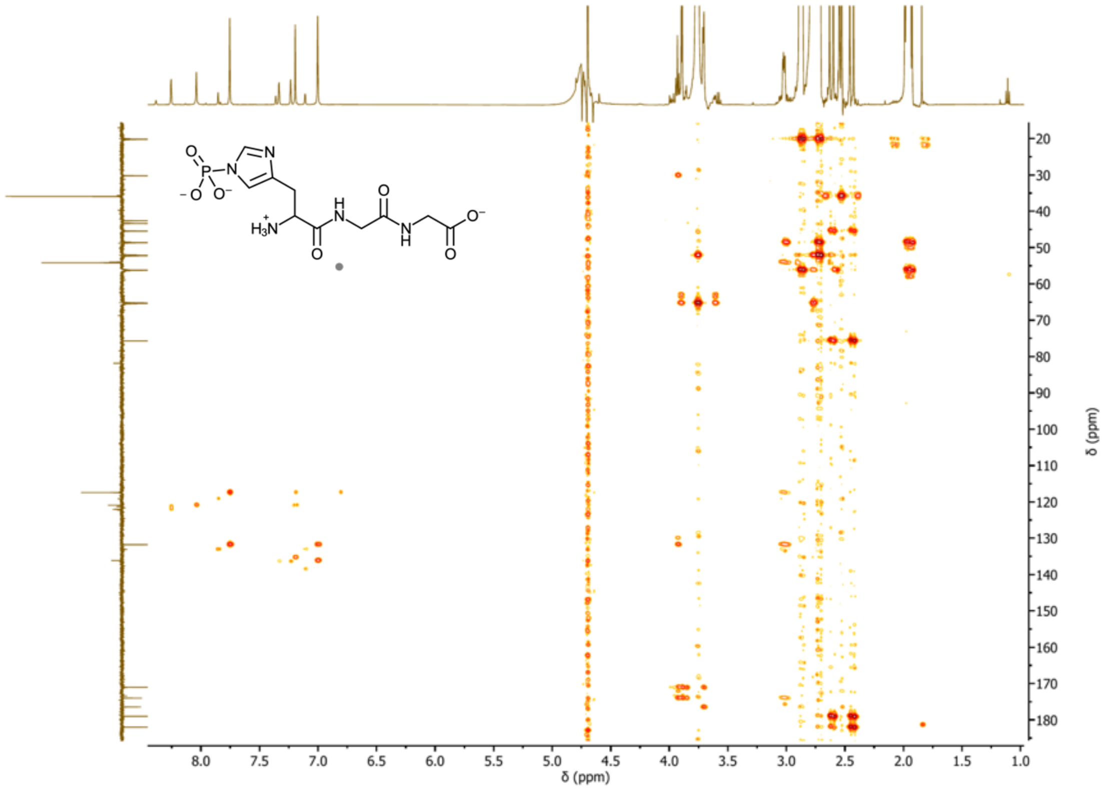

Supporting Figure 294: ^{1}H ^{13}C HMBC spectrum of the phosphorylated His-Gly-Gly intermediate. Characterised *in situ* after 48 h starting from a solution of 50 mM His-Gly-Gly and 50 mM calcium imidazole phosphate in 0.5 M MOPS buffer at pH 7.5 in 9 : 1 H_2O : D_2O containing 0.1 M citric acid and 50 mM HMPA internal standard.

S5.6 Phosphorylated Hercynine Intermediate

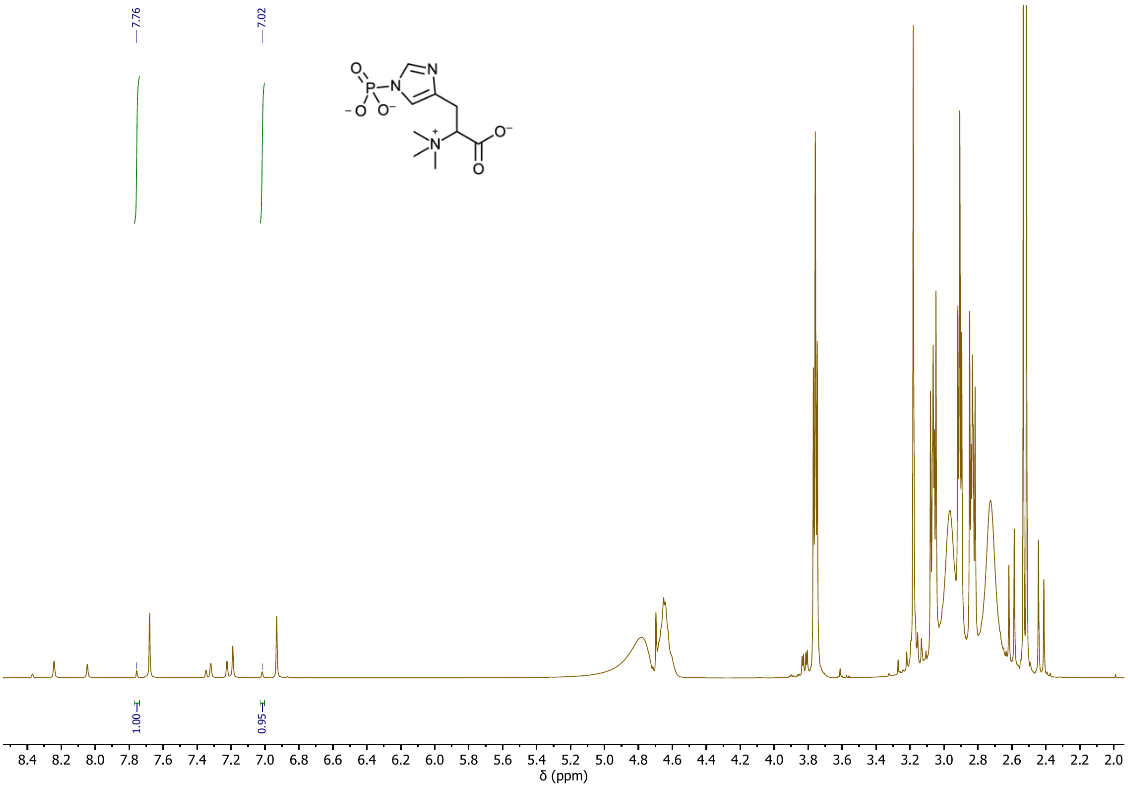

Supporting Figure 295: ^{1}H NMR spectrum of the phosphorylated hercynine intermediate. Characterised *in situ* after 48 h starting from a solution of 50 mM hercynine and 50 mM calcium imidazole phosphate in 0.5 M MOPS buffer at pH 7.5 in 9 : 1 H_2O : D_2O containing 0.1 M citric acid and 50 mM HMPA internal standard.

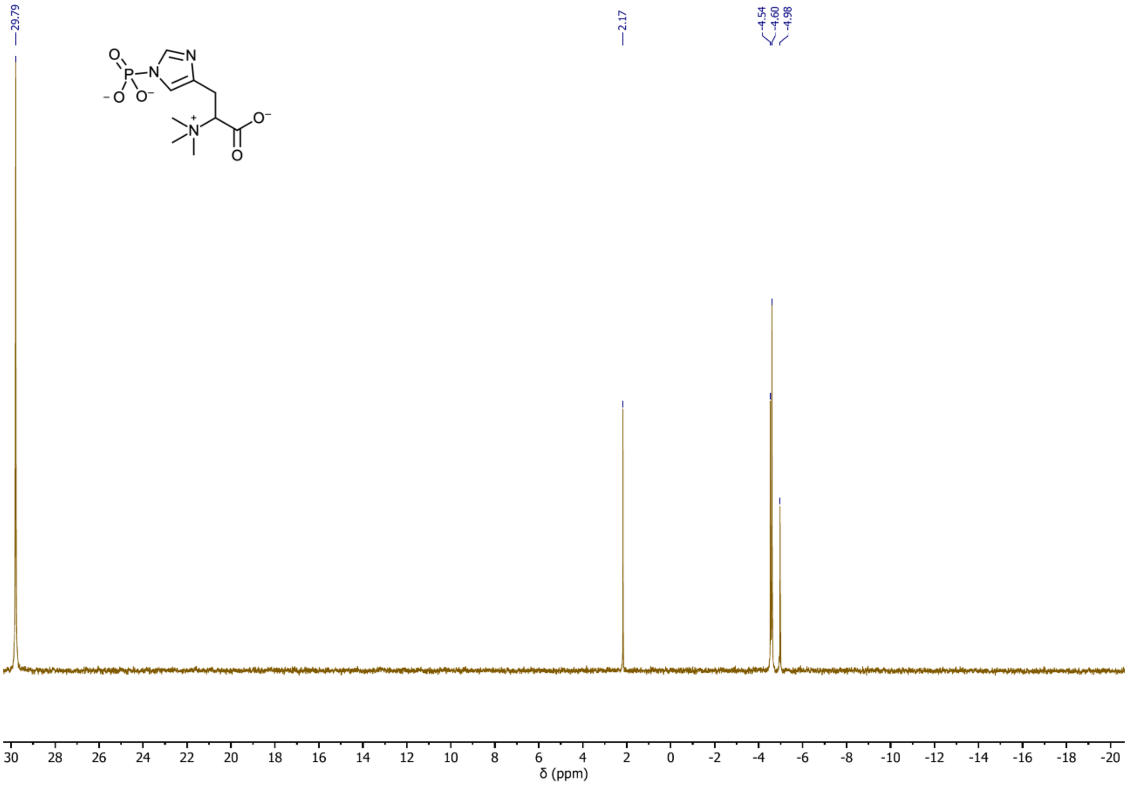

Supporting Figure 296: ^{31}P NMR spectrum of the phosphorylated hercynine intermediate. Characterised *in situ* after 48 h starting from a solution of 50 mM hercynine and 50 mM calcium imidazole phosphate in 0.5 M MOPS buffer at pH 7.5 in 9 : 1 H_2O : D_2O containing 0.1 M citric acid and 50 mM HMPA internal standard.

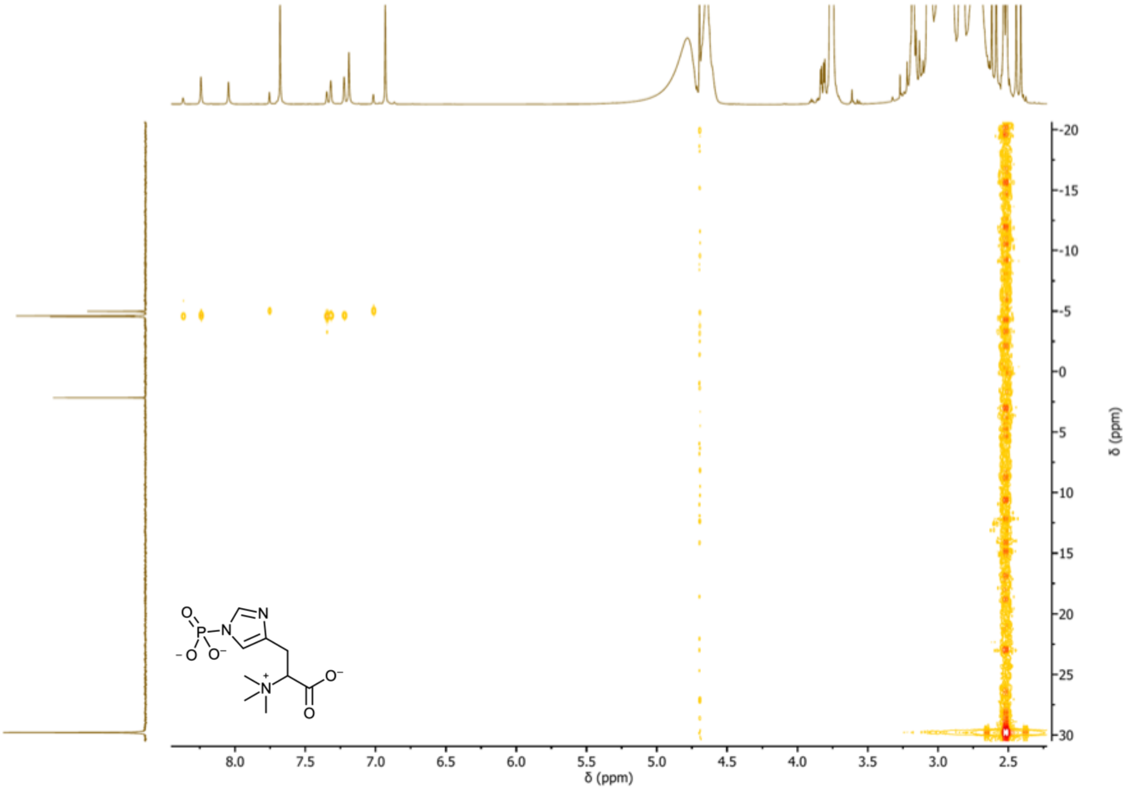

Supporting Figure 297: ^{1}H ^{31}P HMBC spectrum of the phosphorylated hercynine intermediate. Characterised *in situ* after 48 h starting from a solution of 50 mM hercynine and 50 mM calcium imidazole phosphate in 0.5 M MOPS buffer at pH 7.5 in 9 : 1 H_2O : D_2O containing 0.1 M citric acid and 50 mM HMPA internal standard.

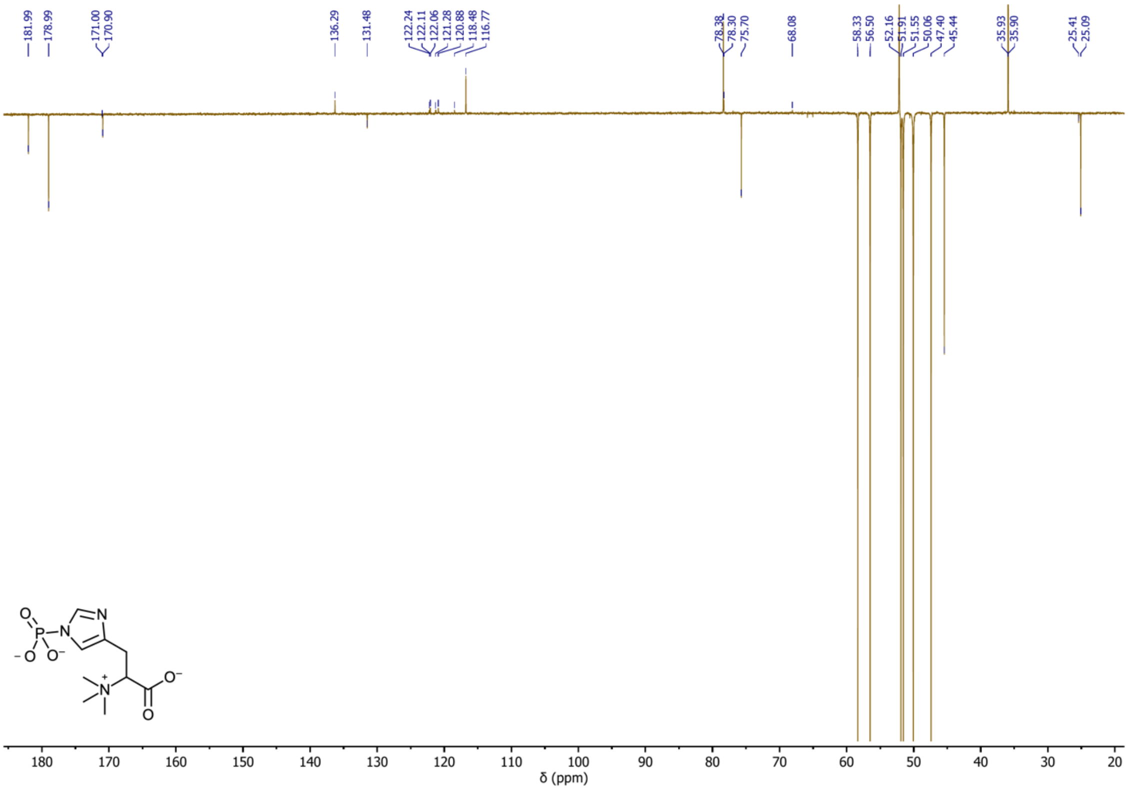

Supporting Figure 298: ^{13}C NMR spectrum of the phosphorylated hercynine intermediate. Characterised *in situ* after 48 h starting from a solution of 50 mM hercynine and 50 mM calcium imidazole phosphate in 0.5 M MOPS buffer at pH 7.5 in 9 : 1 H_2O : D_2O containing 0.1 M citric acid and 50 mM HMPA internal standard.

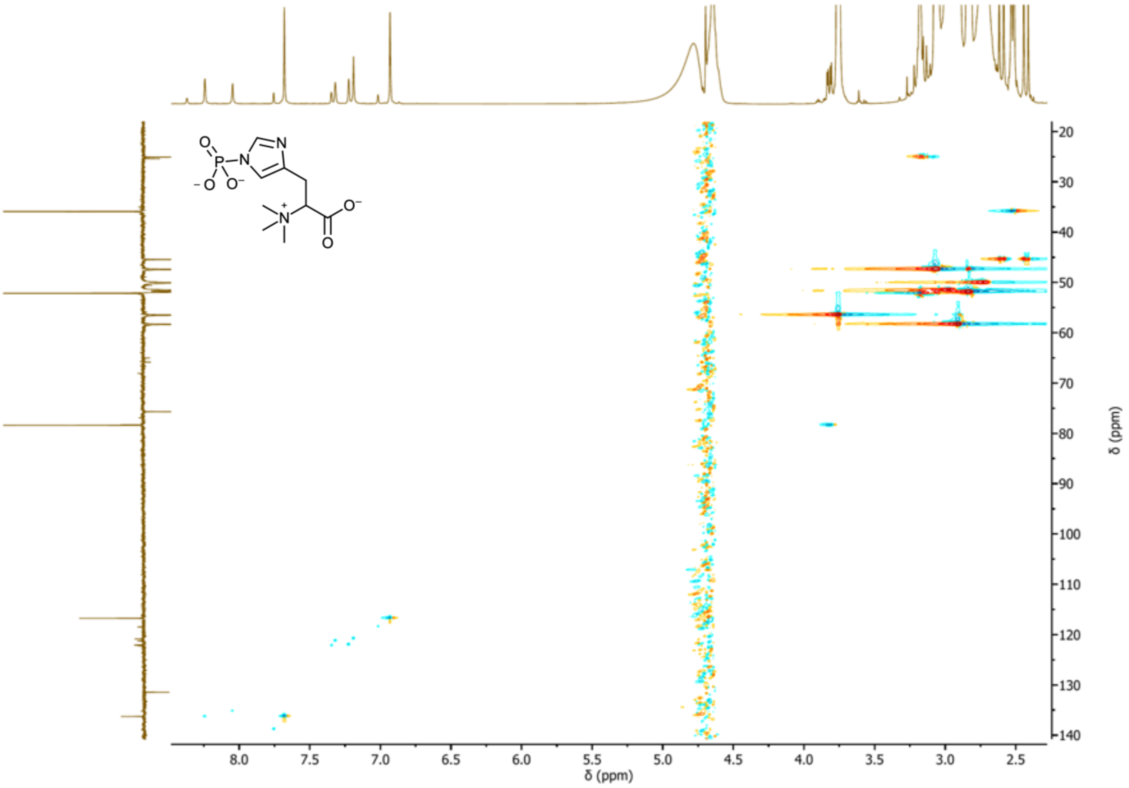

Supporting Figure 299: ^{1}H ^{13}C HSQC spectrum of the phosphorylated hercynine intermediate. Characterised *in situ* after 48 h starting from a solution of 50 mM hercynine and 50 mM calcium imidazole phosphate in 0.5 M MOPS buffer at pH 7.5 in 9 : 1 H_2O : D_2O containing 0.1 M citric acid and 50 mM HMPA internal standard.

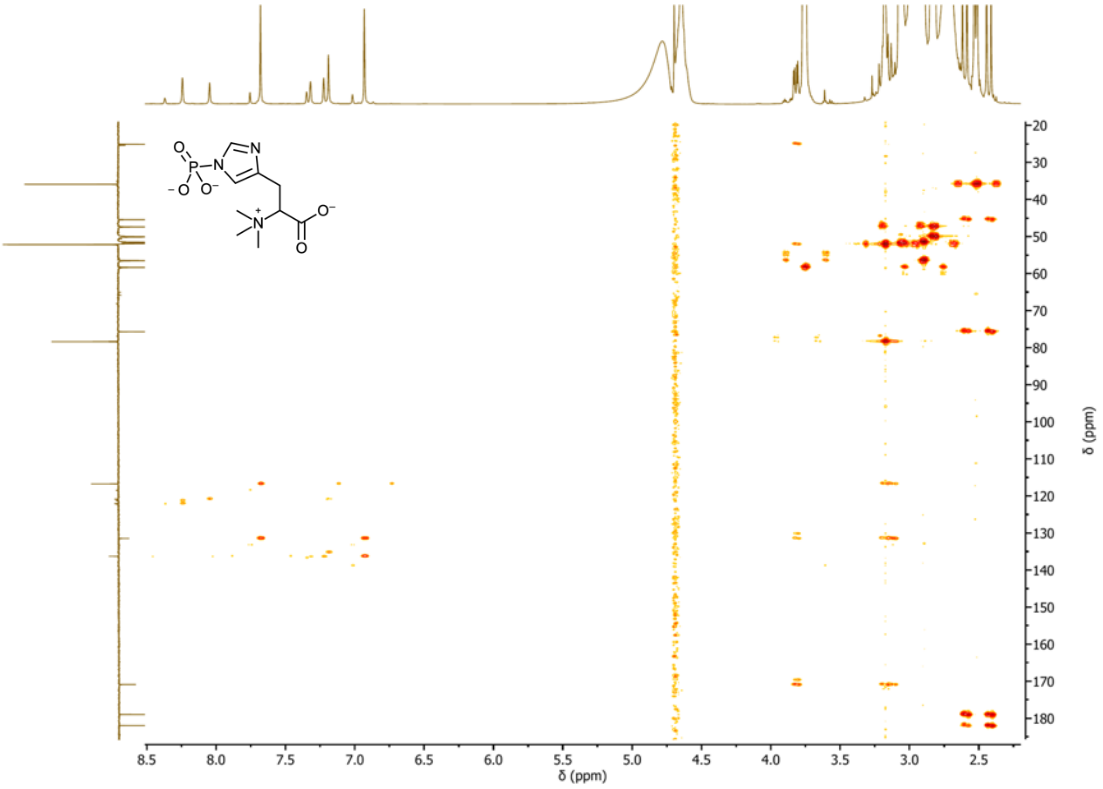

Supporting Figure 300: ^{1}H ^{13}C HMBC spectrum of the phosphorylated hercynine intermediate. Characterised *in situ* after 48 h starting from a solution of 50 mM hercynine and 50 mM calcium imidazole phosphate in 0.5 M MOPS buffer at pH 7.5 in 9 : 1 H_2O : D_2O containing 0.1 M citric acid and 50 mM HMPA internal standard.

S5.7 Phosphorylated His-Gly Intermediate

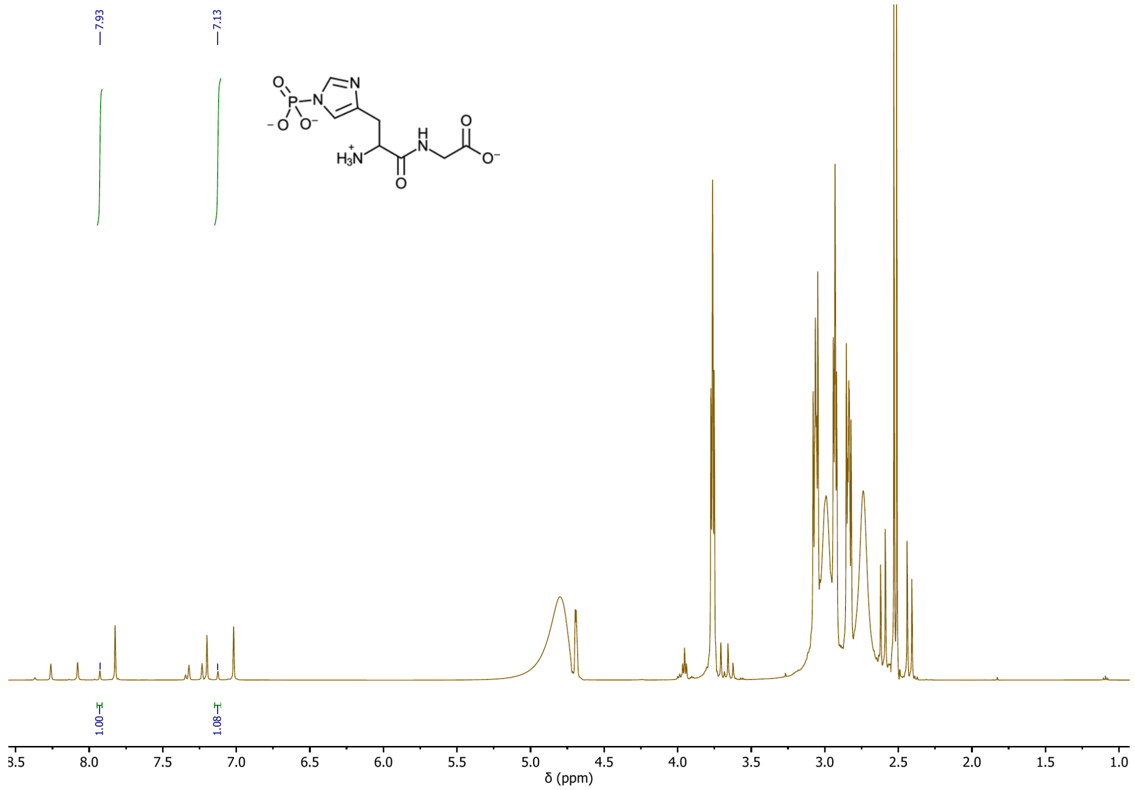

Supporting Figure 301: ^{1}H NMR spectrum of the phosphorylated His-Gly intermediate. Characterised *in situ* after 48 h starting from a solution of 50 mM His-Gly and 50 mM calcium imidazole phosphate in 0.5 M MOPS buffer at pH 7.5 in 9 : 1 H_2O : D_2O containing 0.1 M citric acid and 50 mM HMPA internal standard.

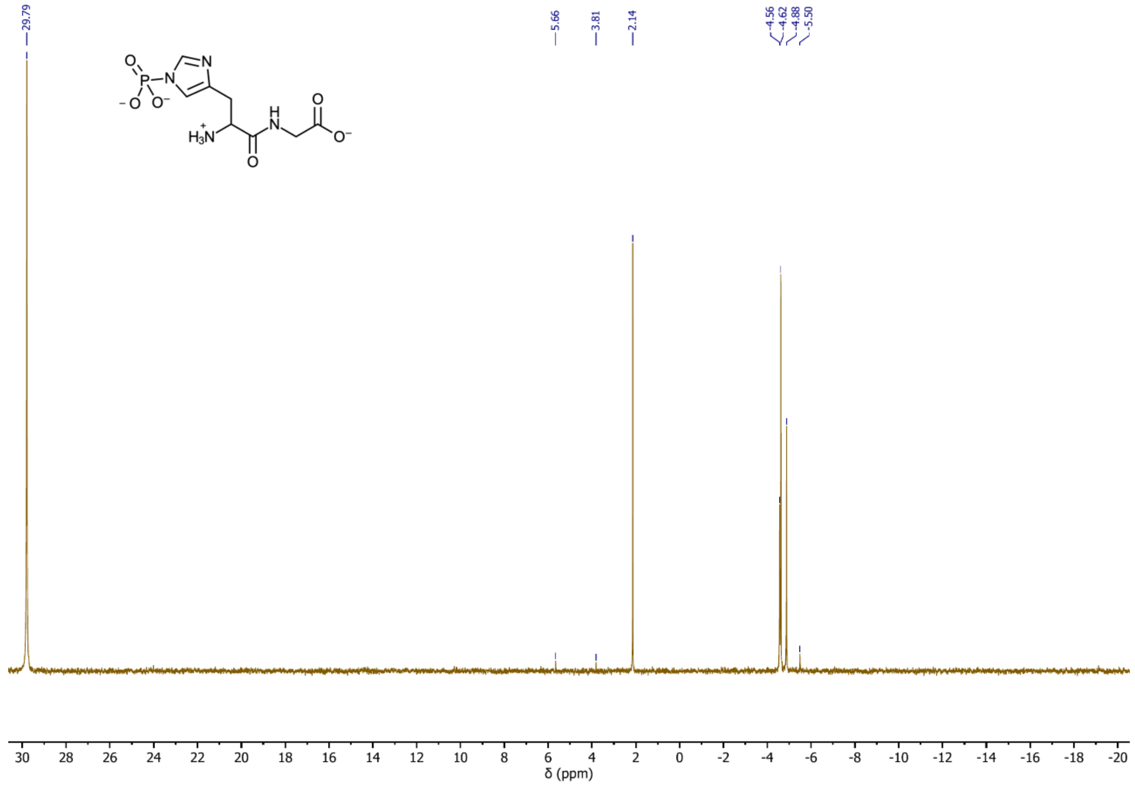

Supporting Figure 302: ^{31}P NMR spectrum of the phosphorylated His-Gly intermediate. Characterised *in situ* after 48 h starting from a solution of 50 mM His-Gly and 50 mM calcium imidazole phosphate in 0.5 M MOPS buffer at pH 7.5 in 9 : 1 H_2O : D_2O containing 0.1 M citric acid and 50 mM HMPA internal standard.

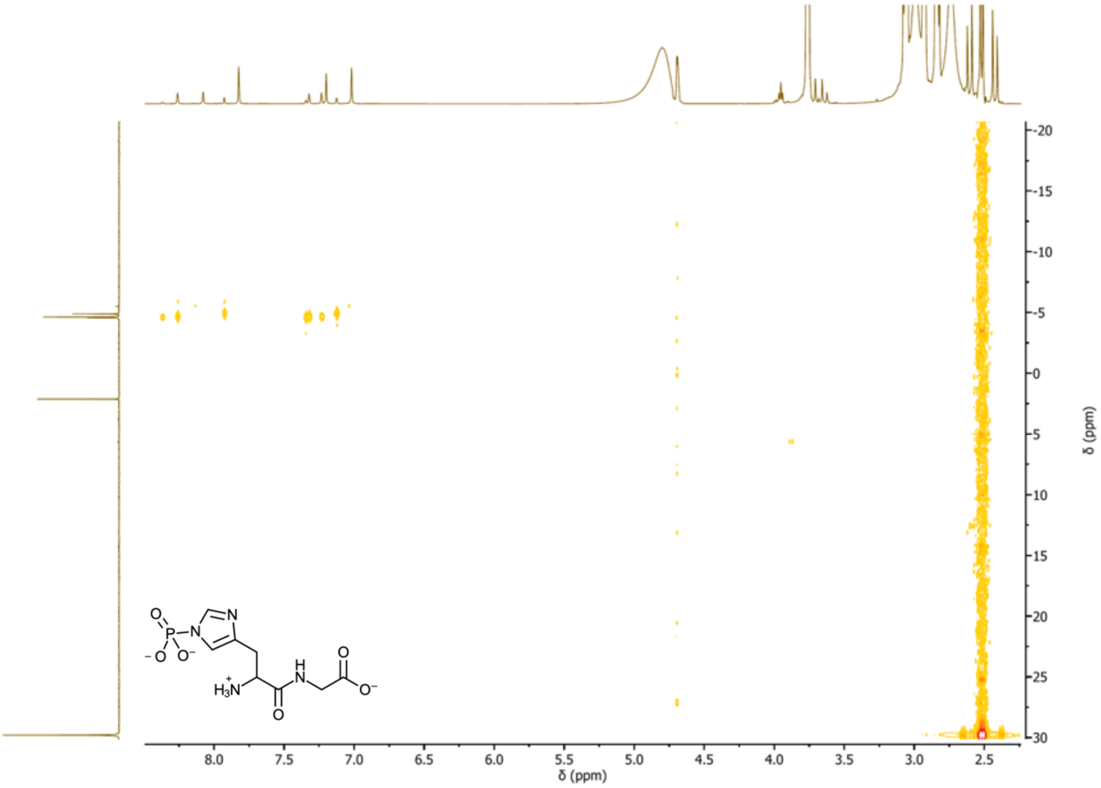

Supporting Figure 303: ^{1}H ^{31}P HMBC spectrum of the phosphorylated His-Gly intermediate. Characterised *in situ* after 48 h starting from a solution of 50 mM His-Gly and 50 mM calcium imidazole phosphate in 0.5 M MOPS buffer at pH 7.5 in 9 : 1 H_2O : D_2O containing 0.1 M citric acid and 50 mM HMPA internal standard.

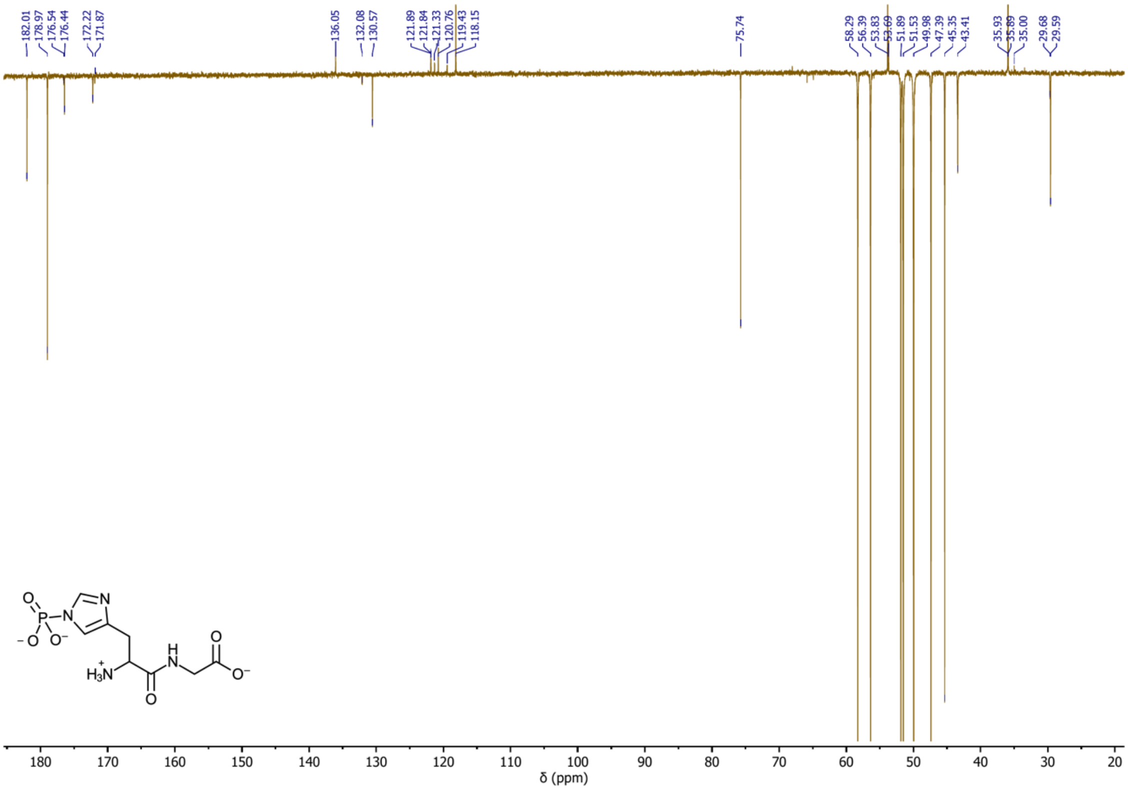

Supporting Figure 304: ^{13}C NMR spectrum of the phosphorylated His-Gly intermediate. Characterised *in situ* after 48 h starting from a solution of 50 mM His-Gly and 50 mM calcium imidazole phosphate in 0.5 M MOPS buffer at pH 7.5 in 9 : 1 H_2O : D_2O containing 0.1 M citric acid and 50 mM HMPA internal standard.

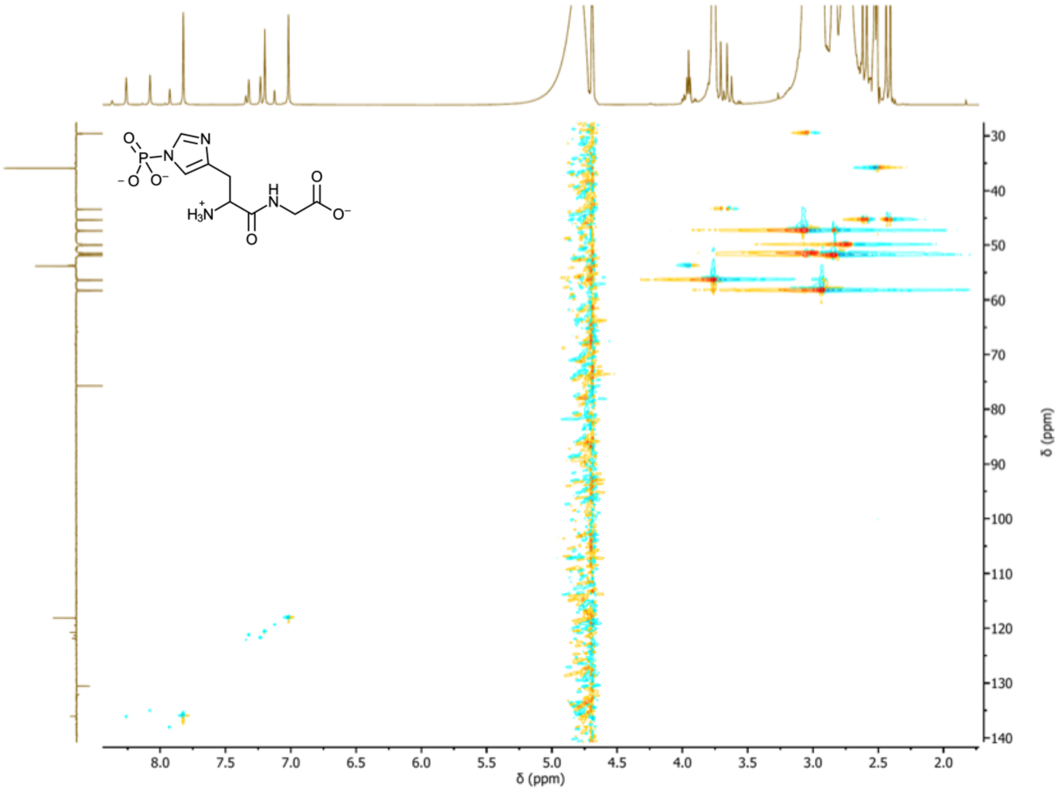

Supporting Figure 305: ^{1}H ^{13}C HSQC spectrum of the phosphorylated His-Gly intermediate. Characterised *in situ* after 48 h starting from a solution of 50 mM His-Gly and 50 mM calcium imidazole phosphate in 0.5 M MOPS buffer at pH 7.5 in 9 : 1 H_2O : D_2O containing 0.1 M citric acid and 50 mM HMPA internal standard.

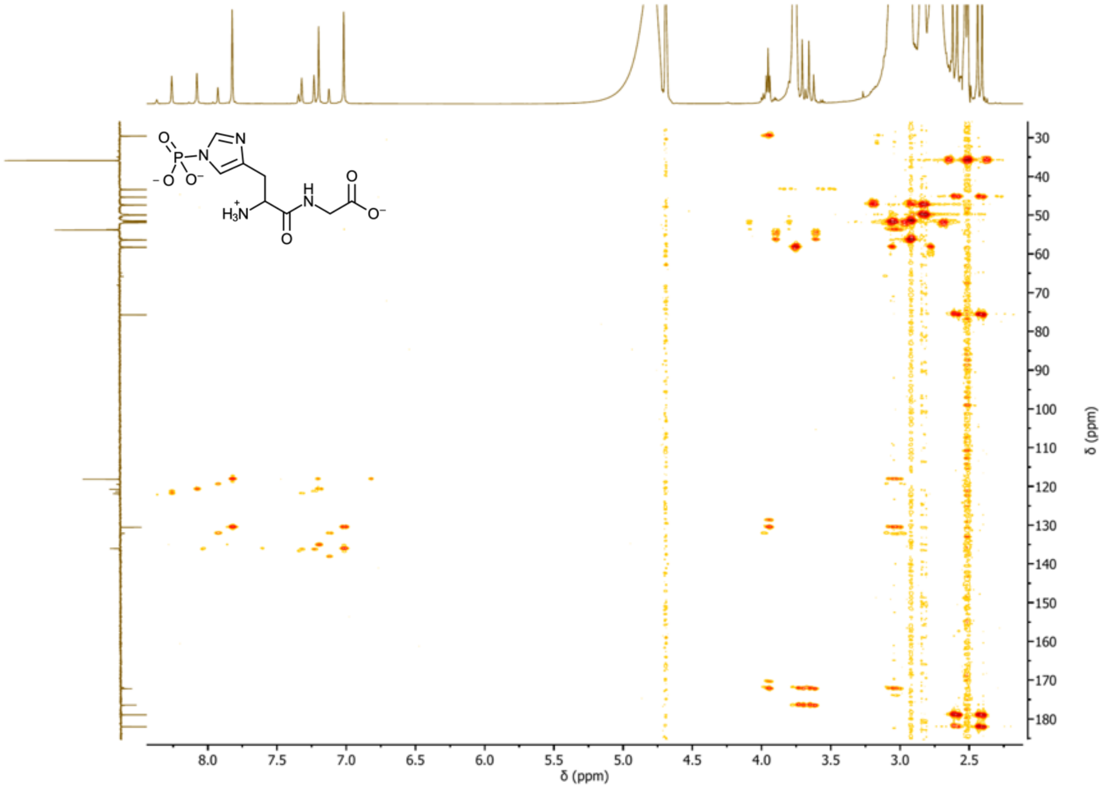

Supporting Figure 306: ^{1}H ^{13}C HMBC spectrum of the phosphorylated His-Gly intermediate. Characterised *in situ* after 48 h starting from a solution of 50 mM His-Gly and 50 mM calcium imidazole phosphate in 0.5 M MOPS buffer at pH 7.5 in 9 : 1 H_2O : D_2O containing 0.1 M citric acid and 50 mM HMPA internal standard.

S5.8 Phosphorylated c(His-Gly) Intermediate

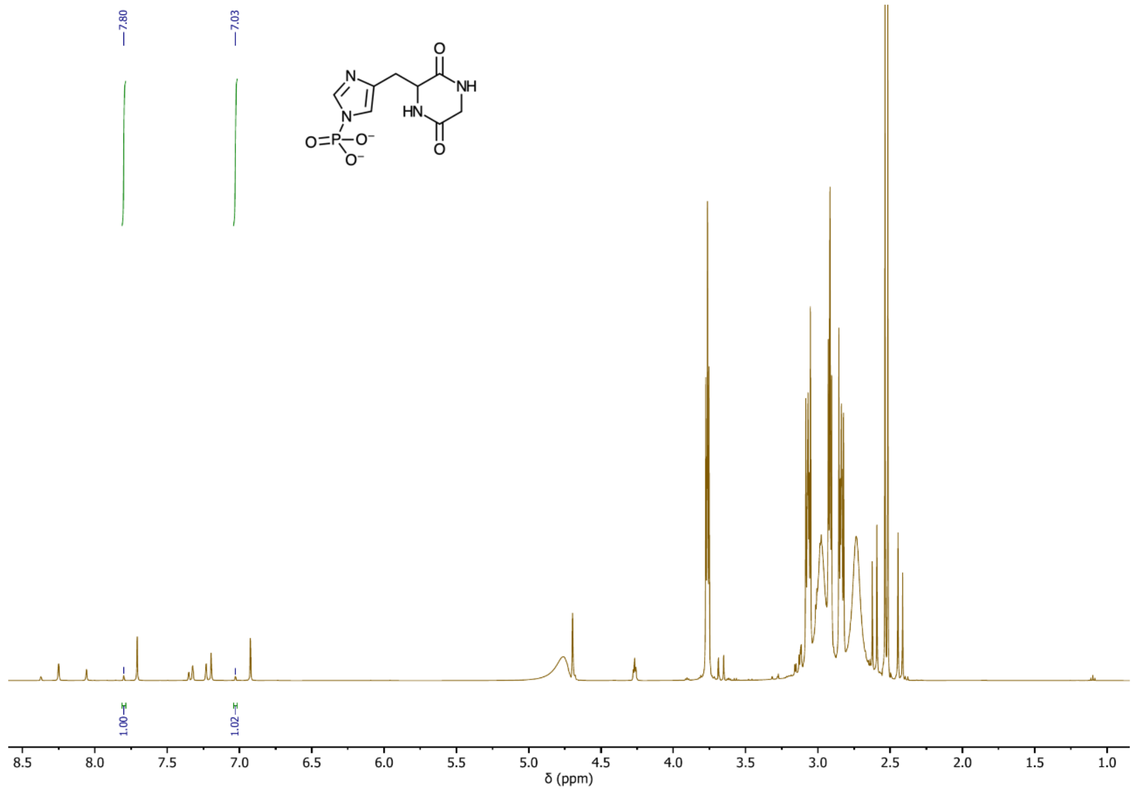

Supporting Figure 307: ^{1}H NMR spectrum of the phosphorylated c(His-Gly) intermediate. Characterised *in situ* after 48 h starting from a solution of 50 mM c(His-Gly) and 50 mM calcium imidazole phosphate in 0.5 M MOPS buffer at pH 7.5 in 9 : 1 H_2O : D_2O containing 0.1 M citric acid and 50 mM HMPA internal standard.

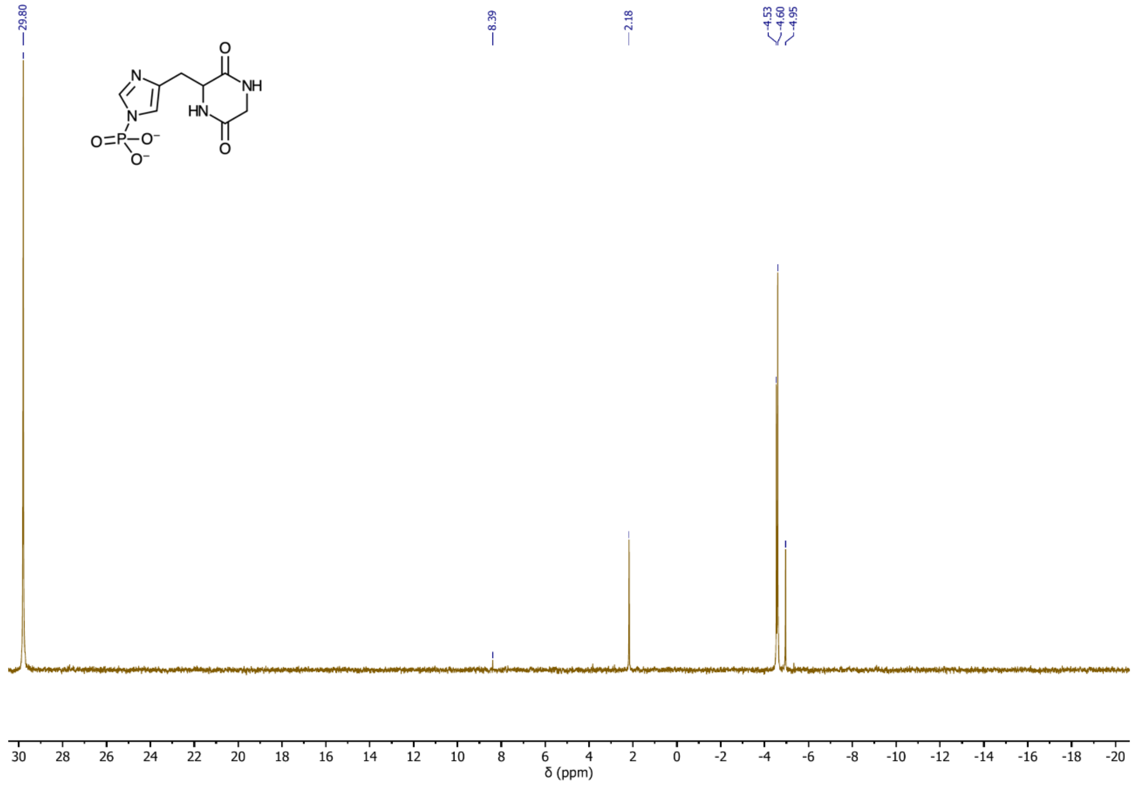

Supporting Figure 308: ^{31}P NMR spectrum of the phosphorylated c(His-Gly) intermediate. Characterised *in situ* after 48 h starting from a solution of 50 mM c(His-Gly) and 50 mM calcium imidazole phosphate in 0.5 M MOPS buffer at pH 7.5 in 9 : 1 H_2O : D_2O containing 0.1 M citric acid and 50 mM HMPA internal standard.

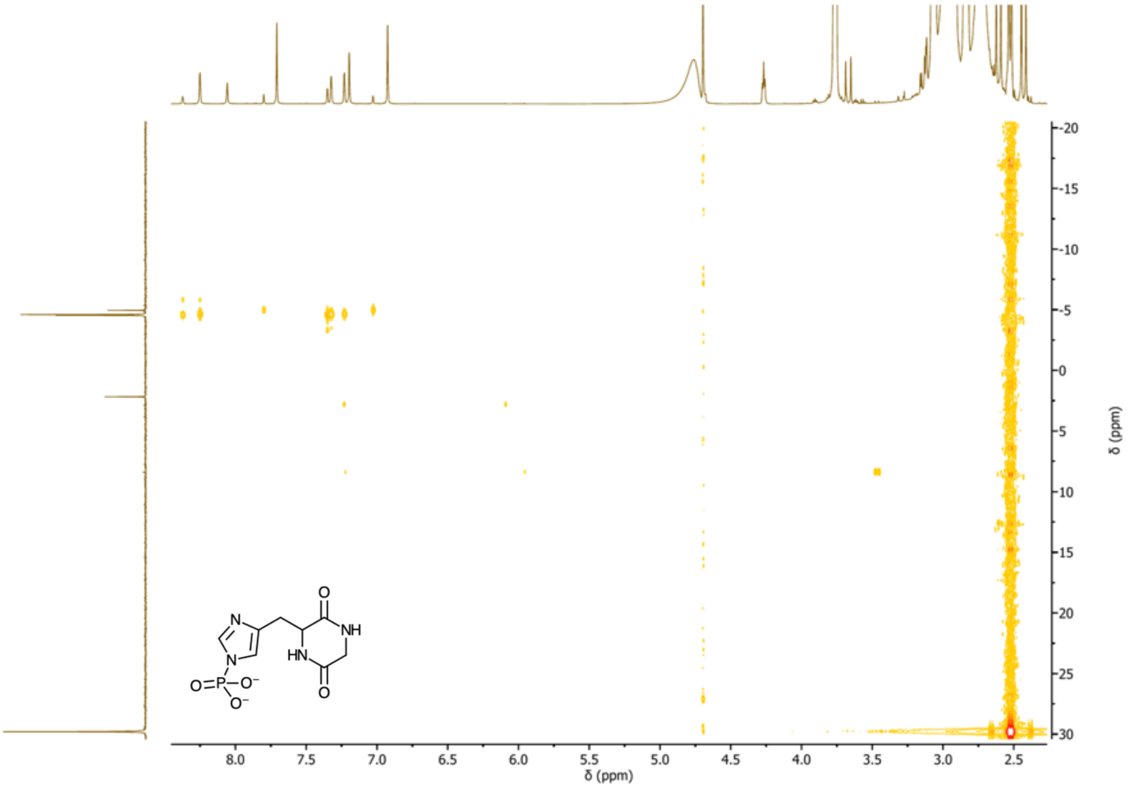

Supporting Figure 309: ^{1}H ^{31}P HMBC spectrum of the phosphorylated c(His-Gly) intermediate. Characterised *in situ* after 48 h starting from a solution of 50 mM c(His-Gly) and 50 mM calcium imidazole phosphate in 0.5 M MOPS buffer at pH 7.5 in 9 : 1 H_2O : D_2O containing 0.1 M citric acid and 50 mM HMPA internal standard.

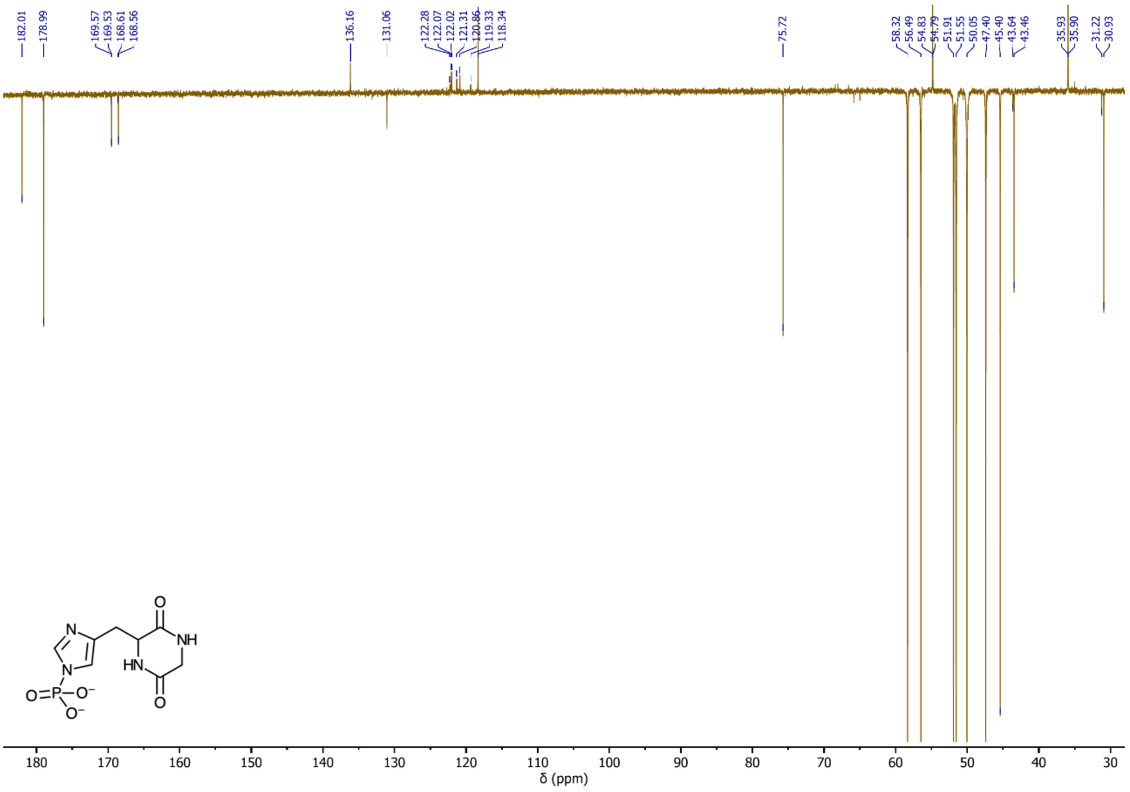

Supporting Figure 310: ^{13}C NMR spectrum of the phosphorylated c(His-Gly) intermediate. Characterised *in situ* after 48 h starting from a solution of 50 mM c(His-Gly) and 50 mM calcium imidazole phosphate in 0.5 M MOPS buffer at pH 7.5 in 9 : 1 H_2O : D_2O containing 0.1 M citric acid and 50 mM HMPA internal standard.

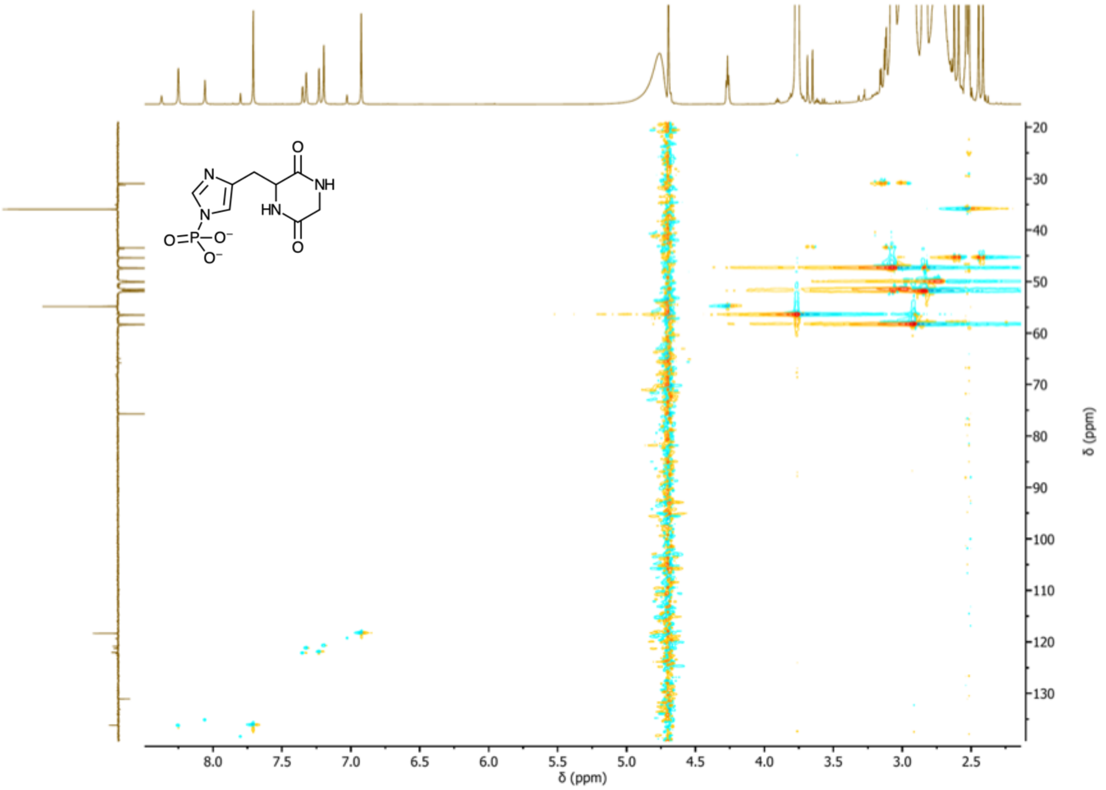

Supporting Figure 311: ^1H ^{13}C HSQC spectrum of the phosphorylated c(His-Gly) intermediate. Characterised *in situ* after 48 h starting from a solution of 50 mM c(His-Gly) and 50 mM calcium imidazole phosphate in 0.5 M MOPS buffer at pH 7.5 in 9 : 1 H_2O : D_2O containing 0.1 M citric acid and 50 mM HMPA internal standard.

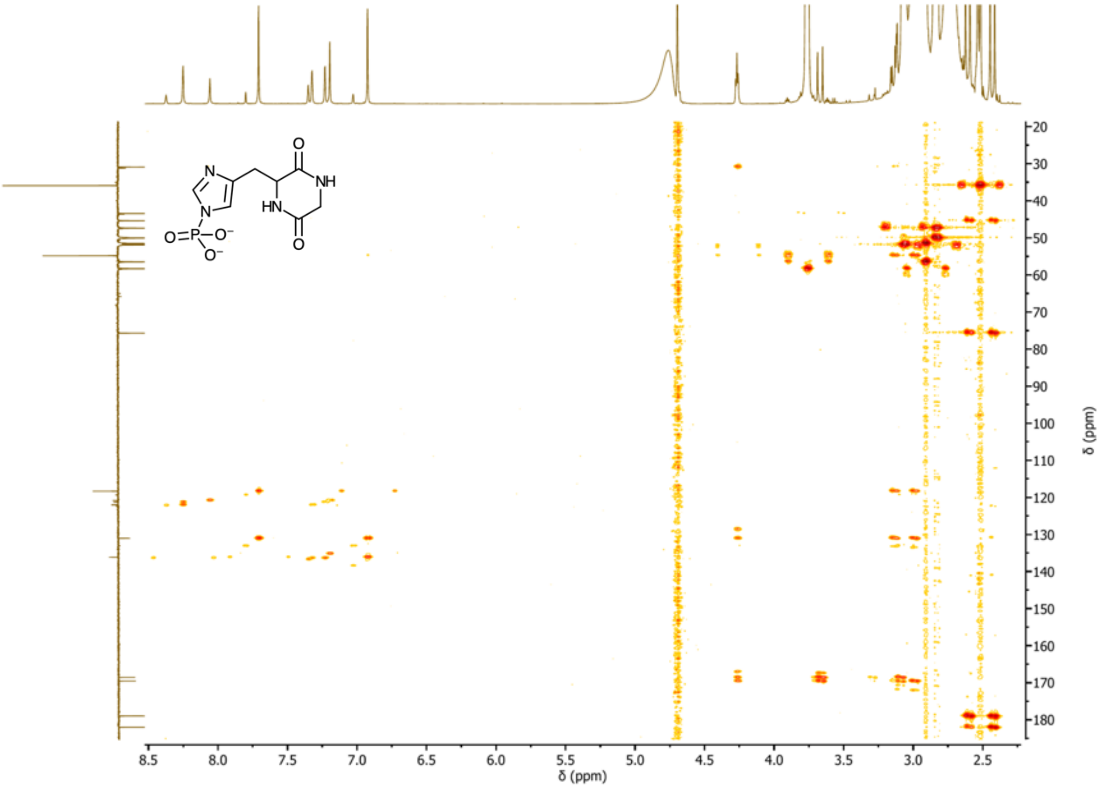

Supporting Figure 312: ^1H ^{13}C HMBC spectrum of the phosphorylated c(His-Gly) intermediate. Characterised *in situ* after 48 h starting from a solution of 50 mM c(His-Gly) and 50 mM calcium imidazole phosphate in 0.5 M MOPS buffer at pH 7.5 in 9 : 1 H_2O : D_2O containing 0.1 M citric acid and 50 mM HMPA internal standard.

S5.9 Phosphorylated Ala-His Intermediate

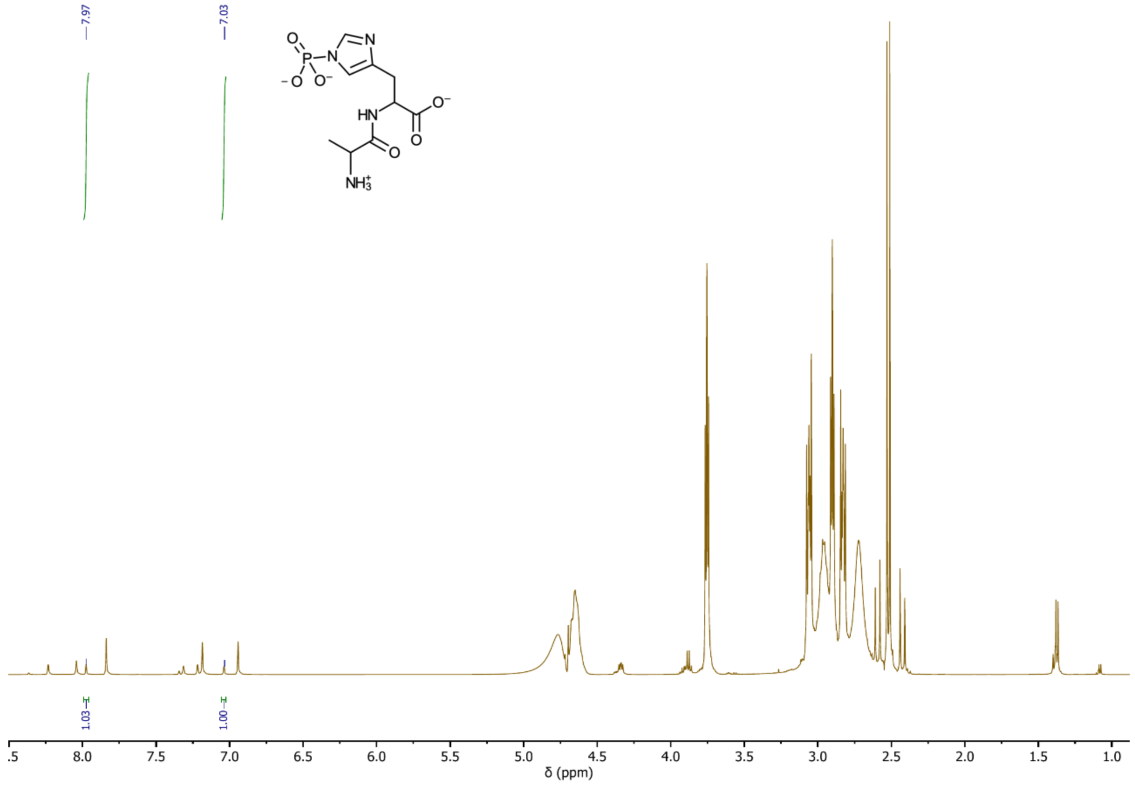

Supporting Figure 313: ^{1}H NMR spectrum of the phosphorylated Ala-His intermediate. Characterised *in situ* after 48 h starting from a solution of 50 mM Ala-His and 50 mM calcium imidazole phosphate in 0.5 M MOPS buffer at pH 7.5 in 9 : 1 H_2O : D_2O containing 0.1 M citric acid and 50 mM HMPA internal standard.

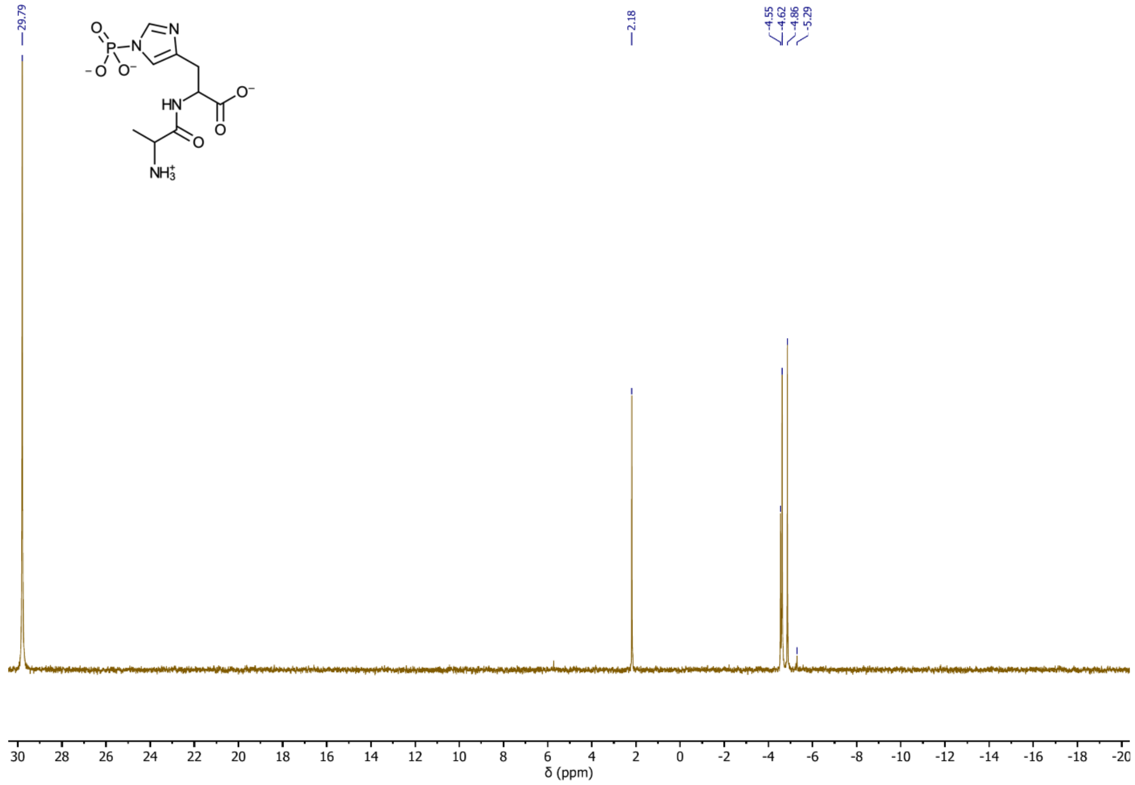

Supporting Figure 314: ^{31}P NMR spectrum of the phosphorylated Ala-His intermediate. Characterised *in situ* after 48 h starting from a solution of 50 mM Ala-His and 50 mM calcium imidazole phosphate in 0.5 M MOPS buffer at pH 7.5 in 9 : 1 H_2O : D_2O containing 0.1 M citric acid and 50 mM HMPA internal standard.

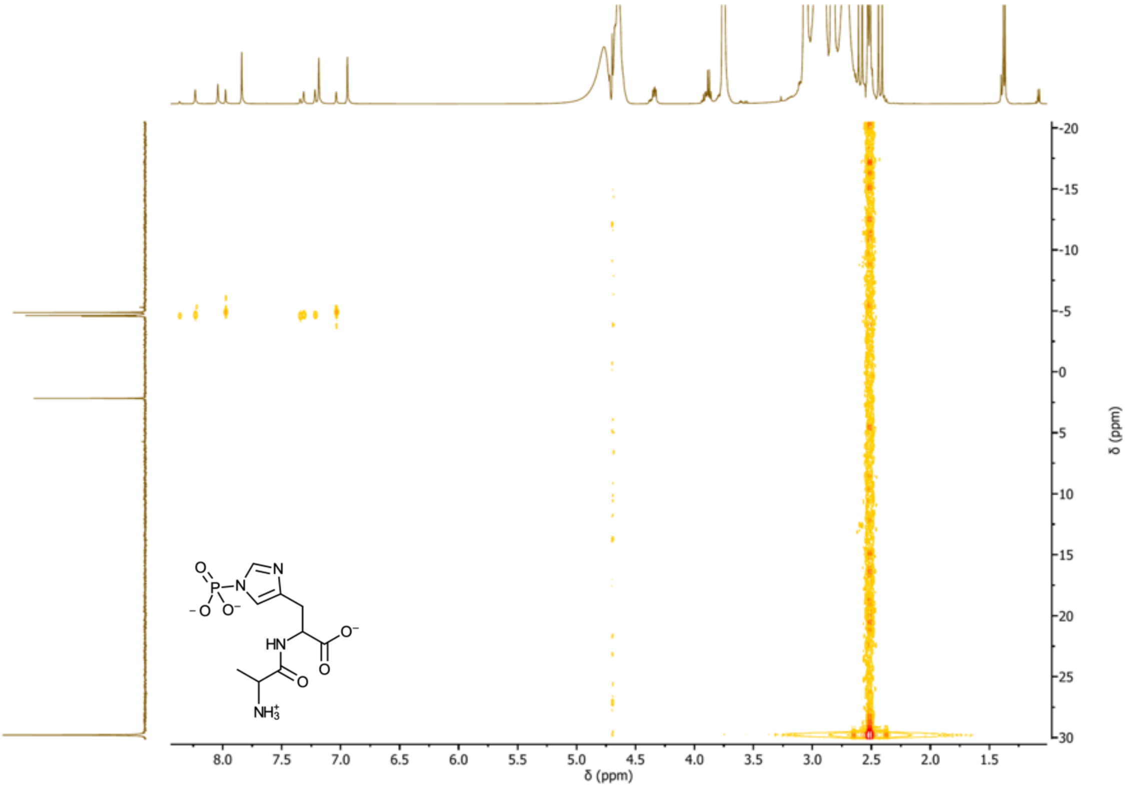

Supporting Figure 315: ^{1}H ^{31}P HMBC spectrum of the phosphorylated Ala-His intermediate. Characterised *in situ* after 48 h starting from a solution of 50 mM Ala-His and 50 mM calcium imidazole phosphate in 0.5 M MOPS buffer at pH 7.5 in 9 : 1 H_2O : D_2O containing 0.1 M citric acid and 50 mM HMPA internal standard.

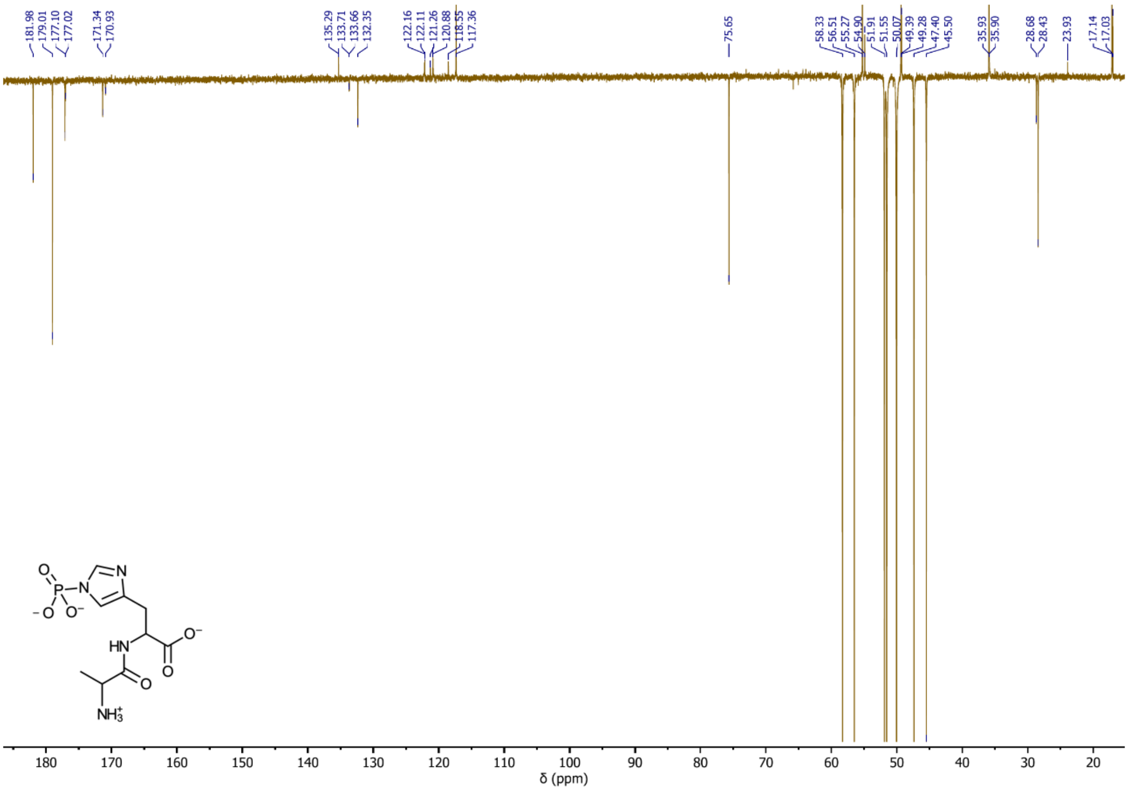

Supporting Figure 316: ^{13}C NMR spectrum of the phosphorylated Ala-His intermediate. Characterised *in situ* after 48 h starting from a solution of 50 mM Ala-His and 50 mM calcium imidazole phosphate in 0.5 M MOPS buffer at pH 7.5 in 9 : 1 H_2O : D_2O containing 0.1 M citric acid and 50 mM HMPA internal standard.

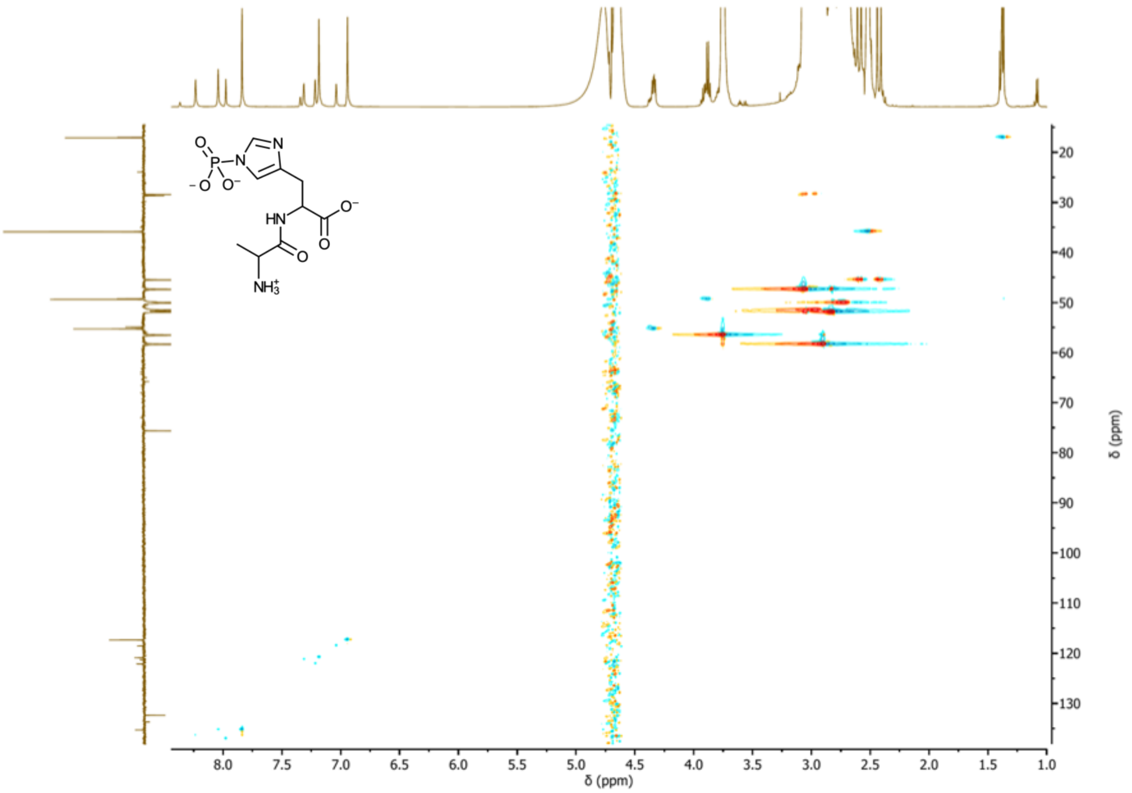

Supporting Figure 317: ^{1}H ^{13}C HSQC spectrum of the phosphorylated Ala-His intermediate. Characterised *in situ* after 48 h starting from a solution of 50 mM Ala-His and 50 mM calcium imidazole phosphate in 0.5 M MOPS buffer at pH 7.5 in 9 : 1 H_2O : D_2O containing 0.1 M citric acid and 50 mM HMPA internal standard.

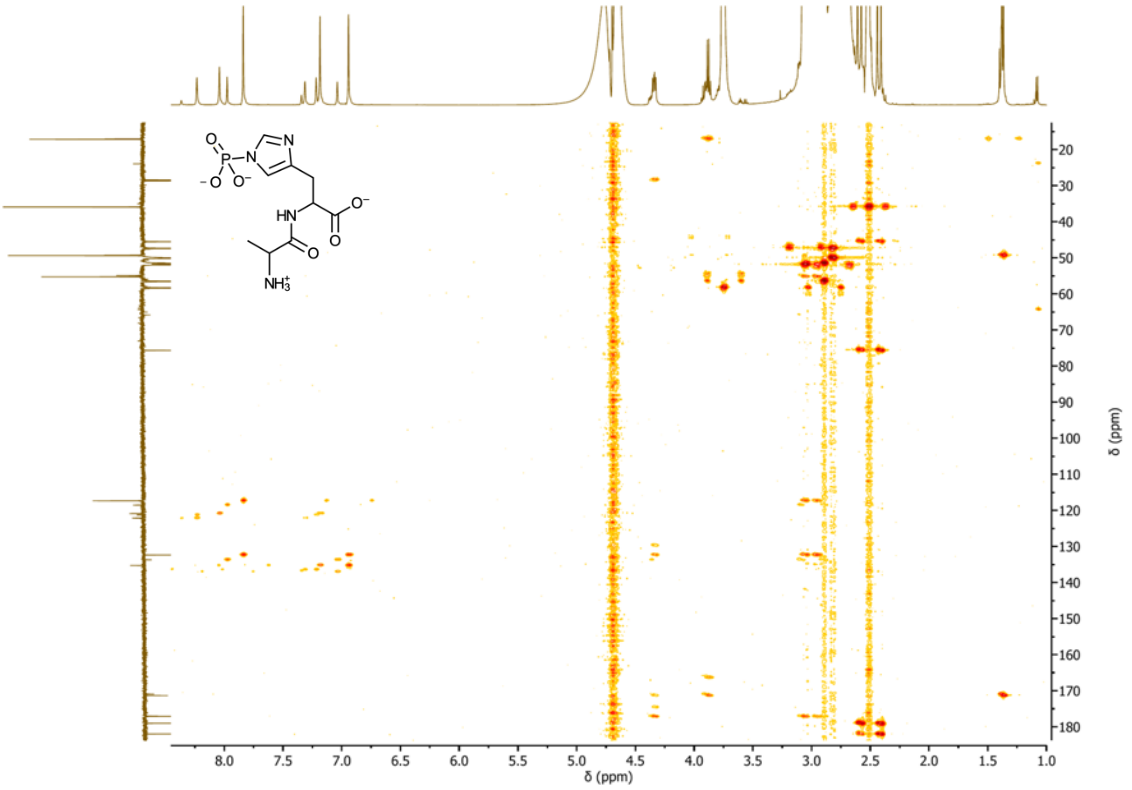

Supporting Figure 318: ^{1}H ^{13}C HMBC spectrum of the phosphorylated Ala-His intermediate. Characterised *in situ* after 48 h starting from a solution of 50 mM Ala-His and 50 mM calcium imidazole phosphate in 0.5 M MOPS buffer at pH 7.5 in 9 : 1 H_2O : D_2O containing 0.1 M citric acid and 50 mM HMPA internal standard.

S5.10 Phosphorylated Ser-His Intermediate

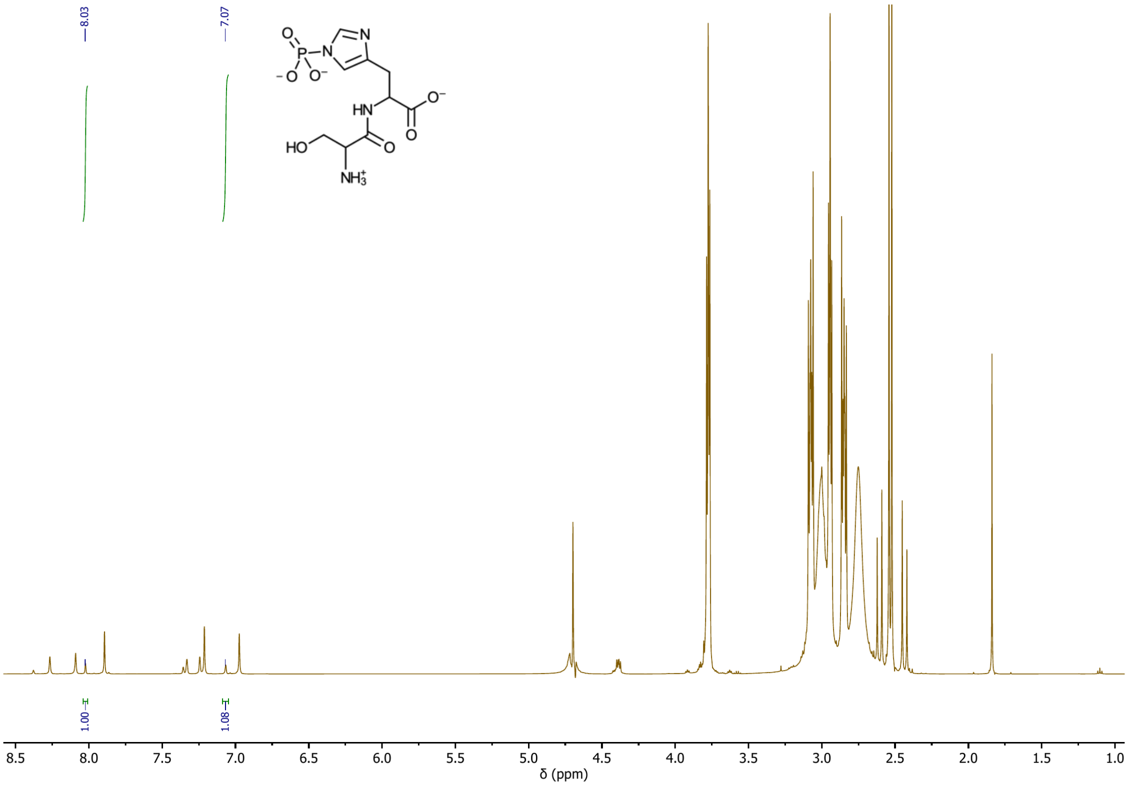

Supporting Figure 319: ^{1}H NMR spectrum of the phosphorylated Ser-His intermediate. Characterised *in situ* after 48 h starting from a solution of 50 mM Ser-His and 50 mM calcium imidazole phosphate in 0.5 M MOPS buffer at pH 7.5 in 9 : 1 H_2O : D_2O containing 0.1 M citric acid and 50 mM HMPA internal standard.

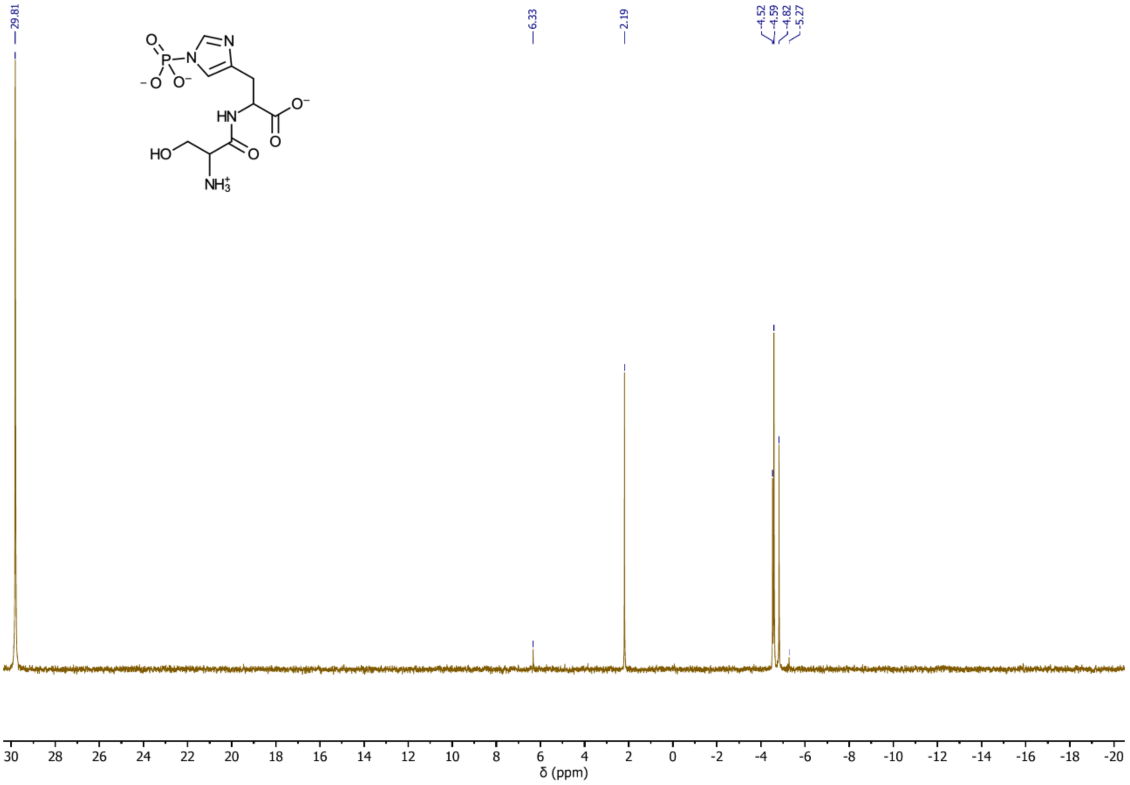

Supporting Figure 320: ^{31}P NMR spectrum of the phosphorylated Ser-His intermediate. Characterised *in situ* after 48 h starting from a solution of 50 mM Ser-His and 50 mM calcium imidazole phosphate in 0.5 M MOPS buffer at pH 7.5 in 9 : 1 H_2O : D_2O containing 0.1 M citric acid and 50 mM HMPA internal standard.

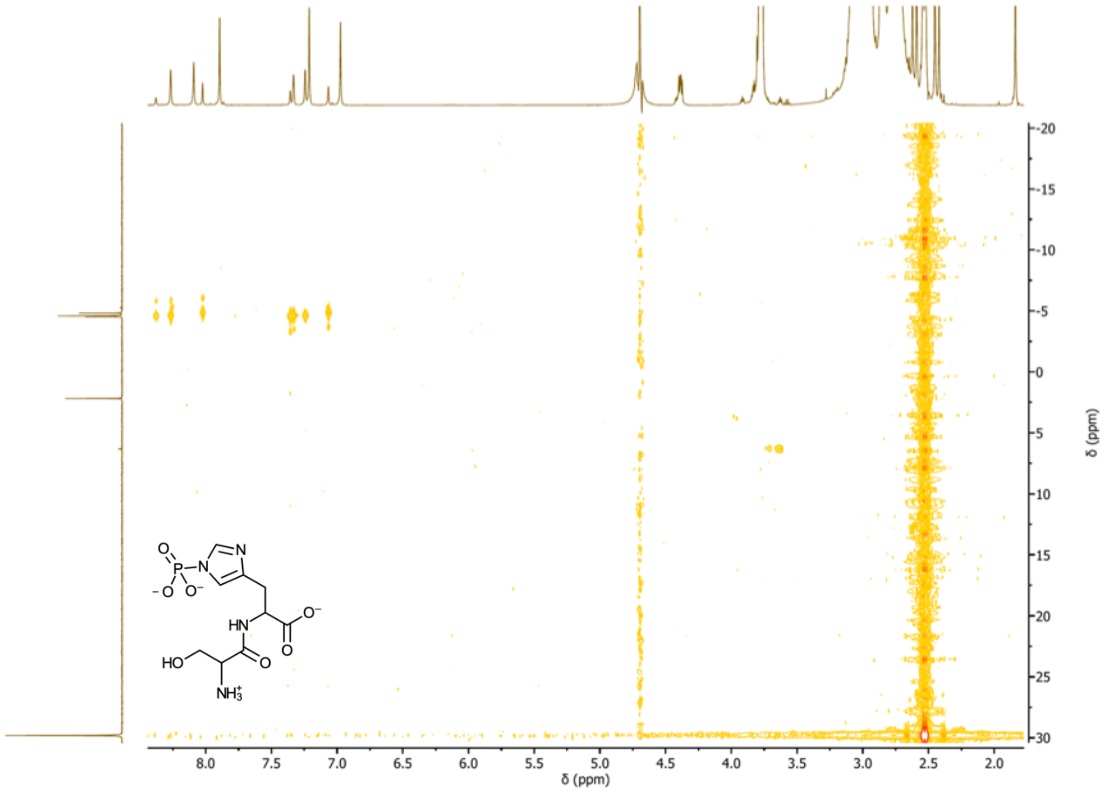

Supporting Figure 321: ^{1}H ^{31}P HMBC spectrum of the phosphorylated Ser-His intermediate. Characterised *in situ* after 48 h starting from a solution of 50 mM Ser-His and 50 mM calcium imidazole phosphate in 0.5 M MOPS buffer at pH 7.5 in 9 : 1 H_2O : D_2O containing 0.1 M citric acid and 50 mM HMPA internal standard.

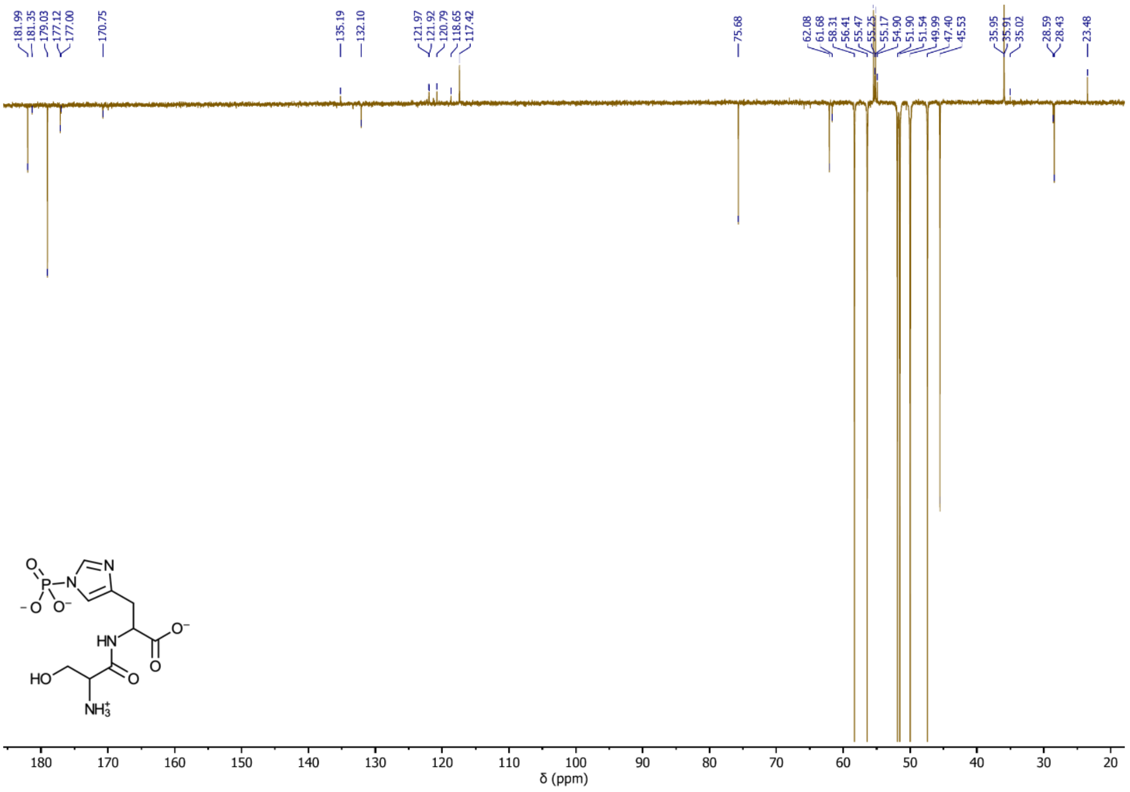

Supporting Figure 322: ^{13}C NMR spectrum of the phosphorylated Ser-His intermediate. Characterised *in situ* after 48 h starting from a solution of 50 mM Ser-His and 50 mM calcium imidazole phosphate in 0.5 M MOPS buffer at pH 7.5 in 9 : 1 H_2O : D_2O containing 0.1 M citric acid and 50 mM HMPA internal standard.

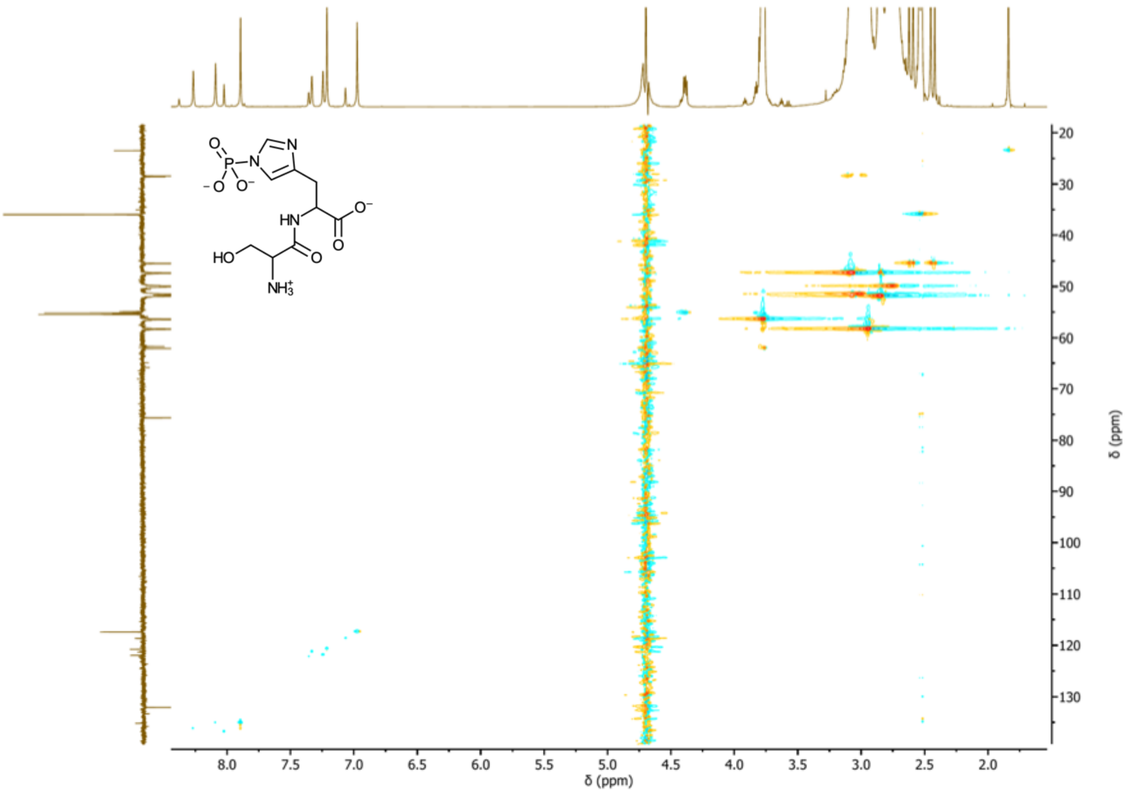

Supporting Figure 323: ^{1}H ^{13}C HSQC spectrum of the phosphorylated Ser-His intermediate. Characterised *in situ* after 48 h starting from a solution of 50 mM Ser-His and 50 mM calcium imidazole phosphate in 0.5 M MOPS buffer at pH 7.5 in 9 : 1 H_2O : D_2O containing 0.1 M citric acid and 50 mM HMPA internal standard.

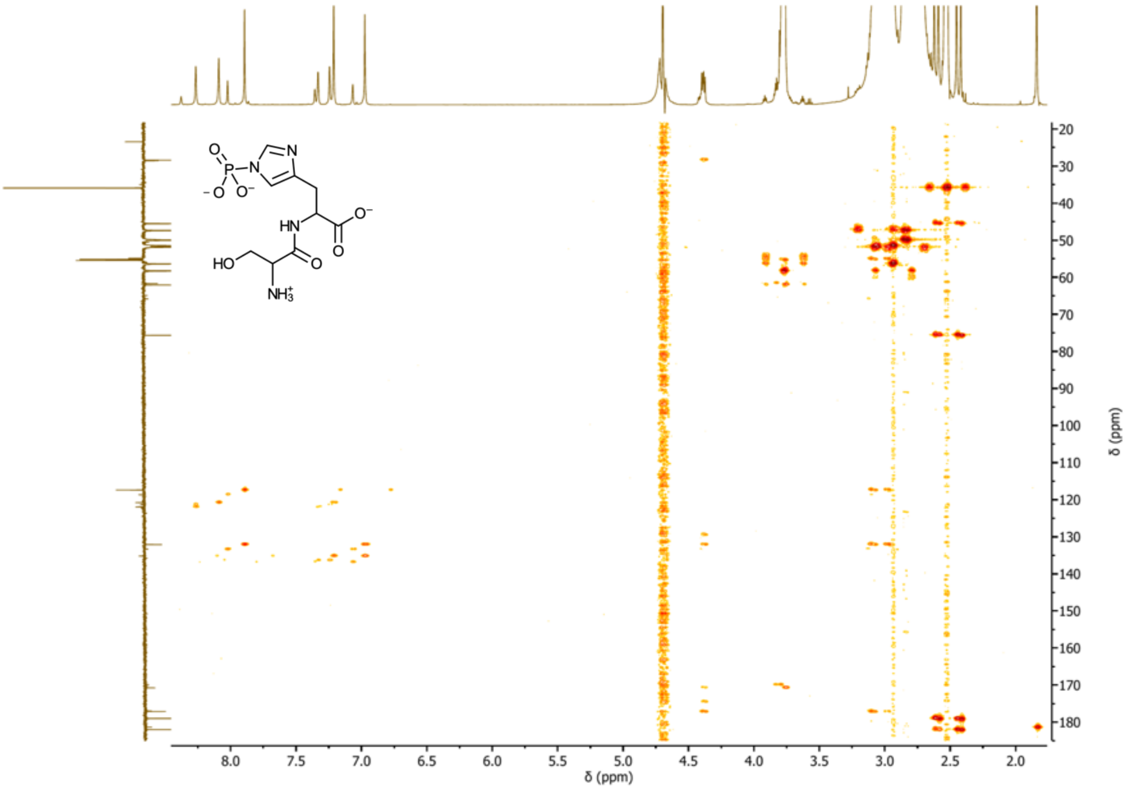

Supporting Figure 324: ^{1}H ^{13}C HMBC spectrum of the phosphorylated Ser-His intermediate. Characterised *in situ* after 48 h starting from a solution of 50 mM Ser-His and 50 mM calcium imidazole phosphate in 0.5 M MOPS buffer at pH 7.5 in 9 : 1 H_2O : D_2O containing 0.1 M citric acid and 50 mM HMPA internal standard.

S5.11 Phosphorylated Arg-His-NH$_2$ Intermediate

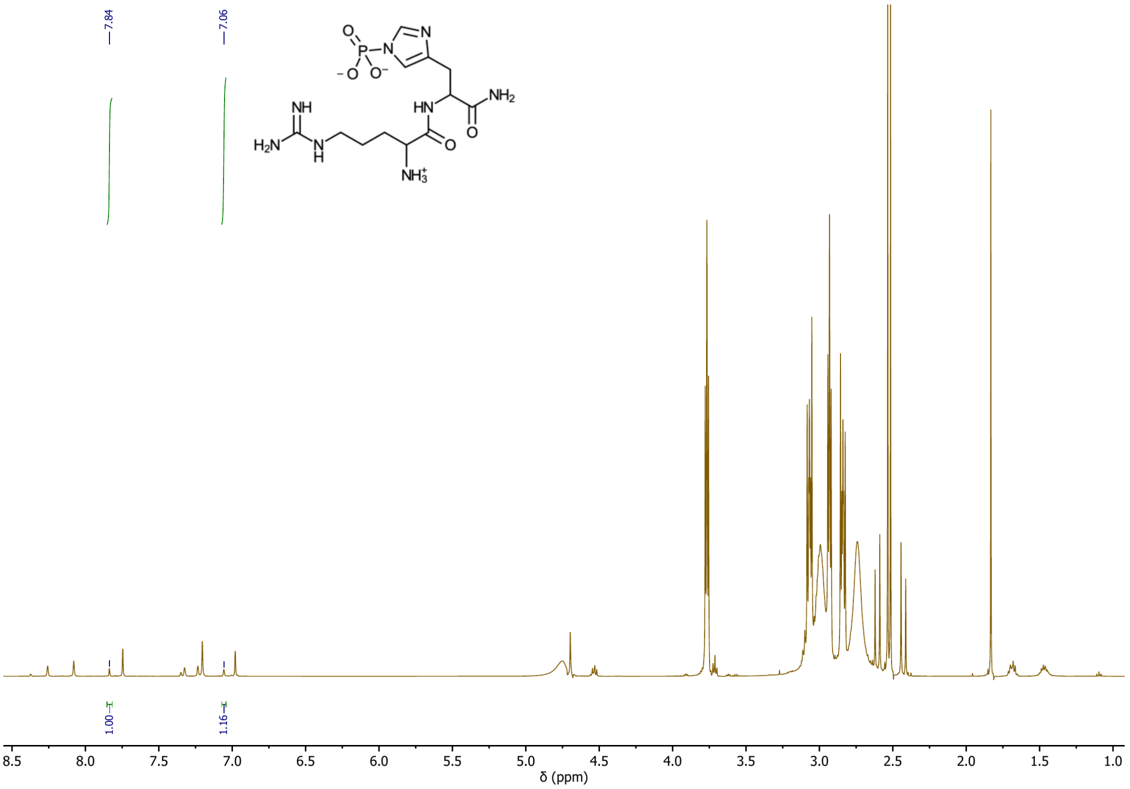

Supporting Figure 325: ^{1}H NMR spectrum of the phosphorylated Arg-His-NH$_2$ intermediate. Characterised *in situ* after 48 h starting from a solution of 50 mM Arg-His-NH$_2$ and 50 mM calcium imidazole phosphate in 0.5 M MOPS buffer at pH 7.5 in 9 : 1 H$_2$O : D$_2$O containing 0.1 M citric acid and 50 mM HMPA internal standard.

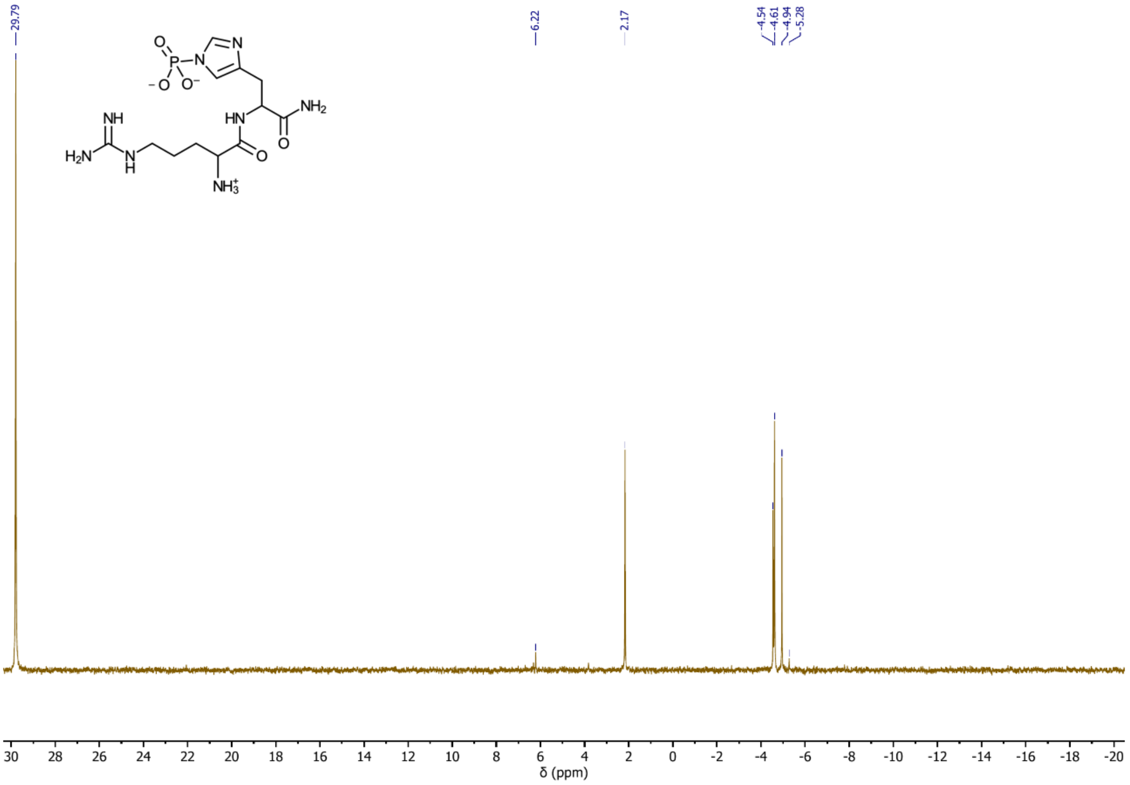

Supporting Figure 326: ^{31}P NMR spectrum of the phosphorylated Arg-His-NH$_2$ intermediate. Characterised *in situ* after 48 h starting from a solution of 50 mM Arg-His-NH$_2$ and 50 mM calcium imidazole phosphate in 0.5 M MOPS buffer at pH 7.5 in 9 : 1 H$_2$O : D$_2$O containing 0.1 M citric acid and 50 mM HMPA internal standard.

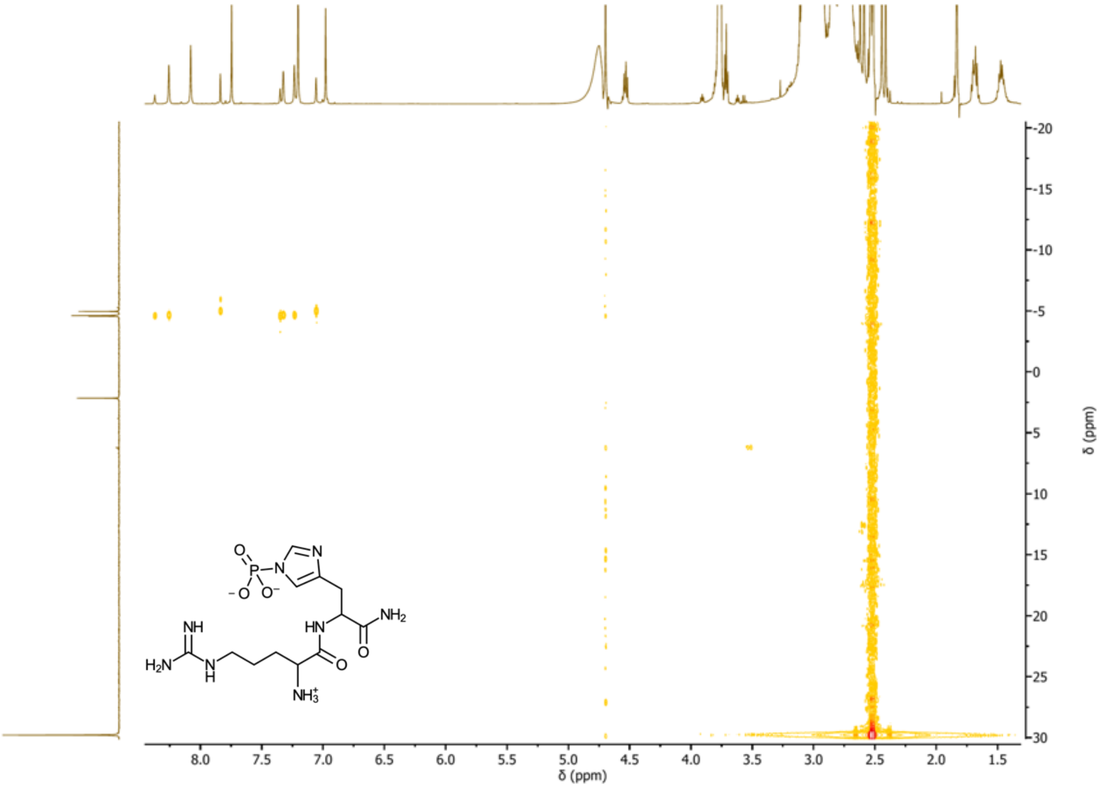

Supporting Figure 327: ^{1}H ^{31}P HMBC spectrum of the phosphorylated Arg-His-NH_2 intermediate. Characterised *in situ* after 48 h starting from a solution of 50 mM Arg-His-NH_2 and 50 mM calcium imidazole phosphate in 0.5 M MOPS buffer at pH 7.5 in 9 : 1 H_2O : D_2O containing 0.1 M citric acid and 50 mM HMPA internal standard.

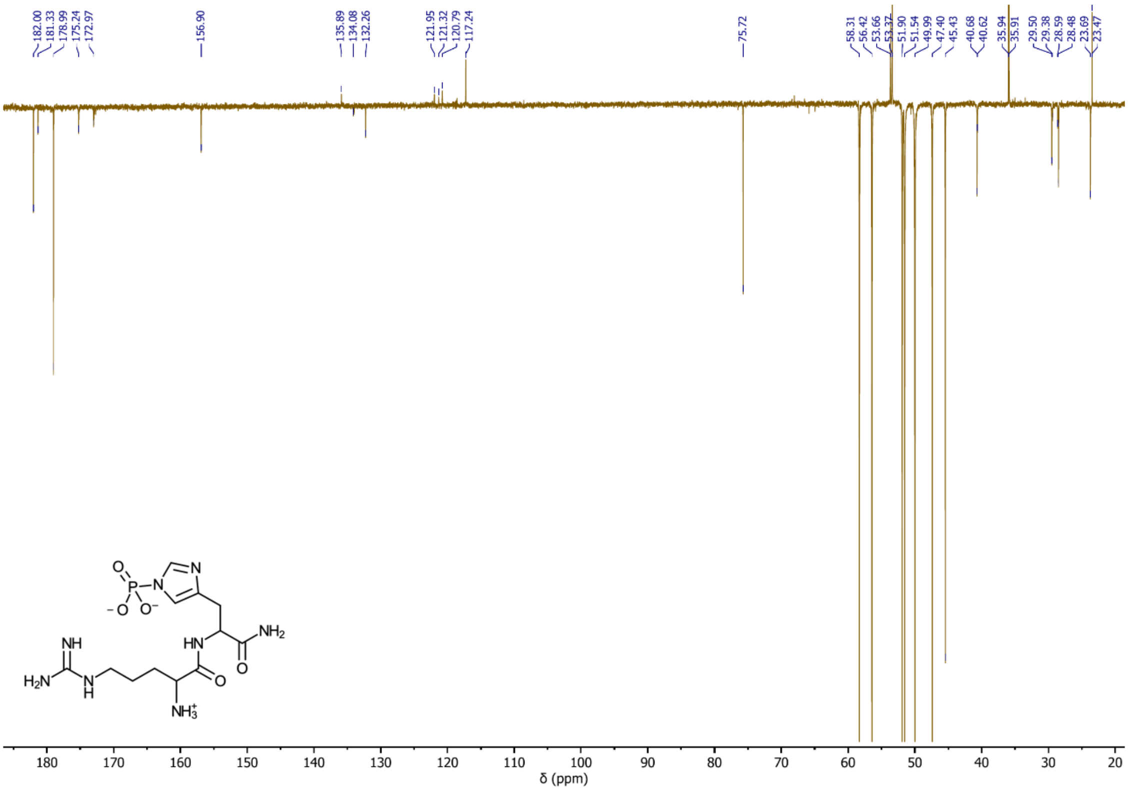

Supporting Figure 328: ^{13}C NMR spectrum of the phosphorylated Arg-His-NH_2 intermediate. Characterised *in situ* after 48 h starting from a solution of 50 mM Arg-His-NH_2 and 50 mM calcium imidazole phosphate in 0.5 M MOPS buffer at pH 7.5 in 9 : 1 H_2O : D_2O containing 0.1 M citric acid and 50 mM HMPA internal standard.

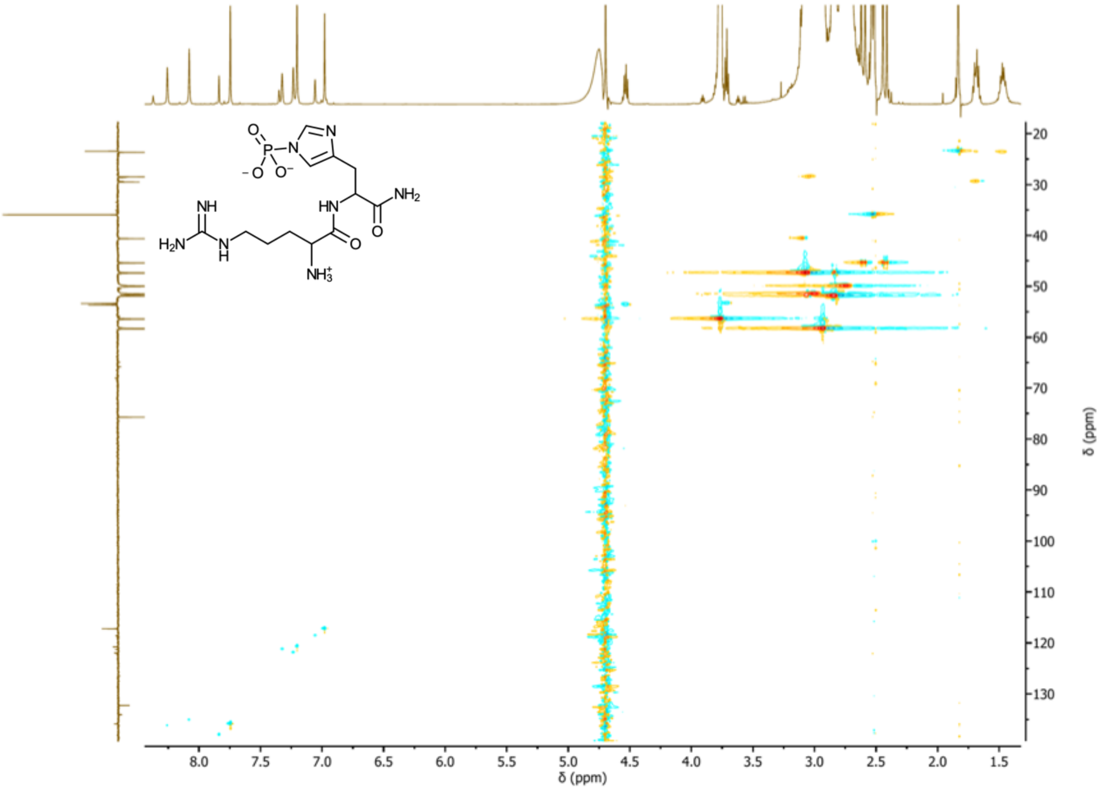

Supporting Figure 329: ^{1}H ^{13}C HSQC spectrum of the phosphorylated Arg-His-NH_2 intermediate. Characterised *in situ* after 48 h starting from a solution of 50 mM Arg-His-NH_2 and 50 mM calcium imidazole phosphate in 0.5 M MOPS buffer at pH 7.5 in 9 : 1 H_2O : D_2O containing 0.1 M citric acid and 50 mM HMPA internal standard.

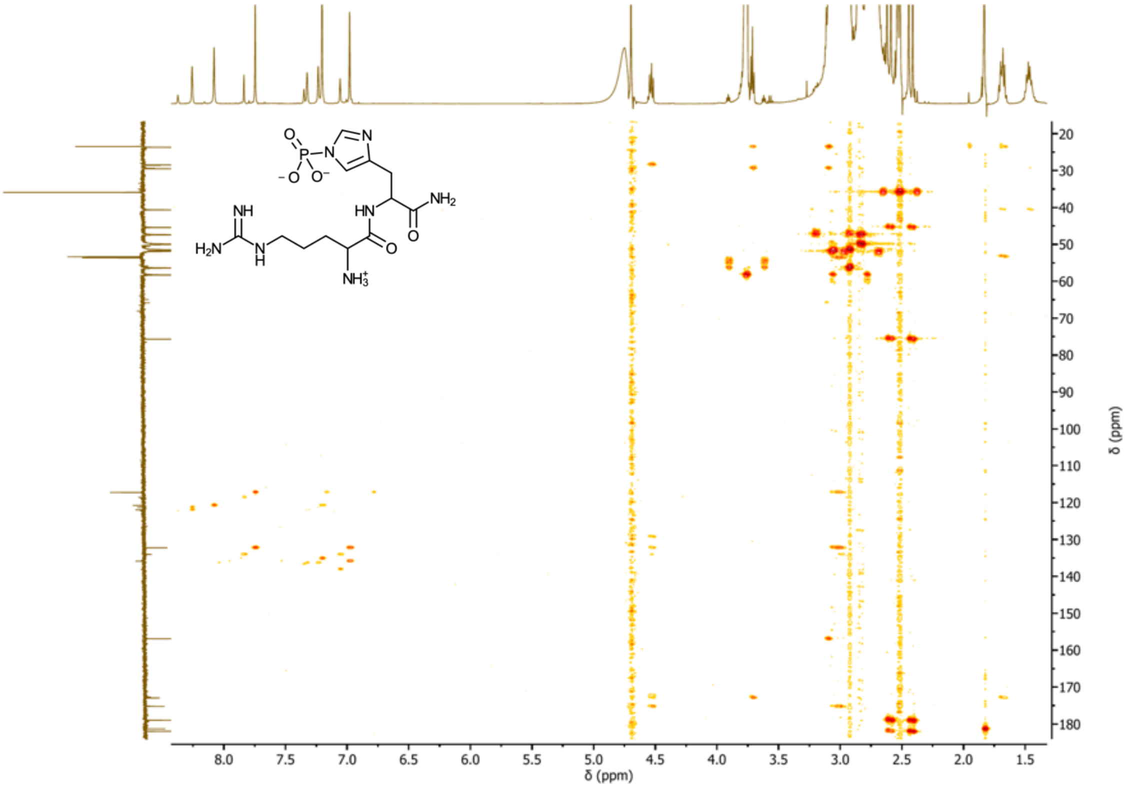

Supporting Figure 330: ^{1}H ^{13}C HMBC spectrum of the phosphorylated Arg-His-NH_2 intermediate. Characterised *in situ* after 48 h starting from a solution of 50 mM Arg-His-NH_2 and 50 mM calcium imidazole phosphate in 0.5 M MOPS buffer at pH 7.5 in 9 : 1 H_2O : D_2O containing 0.1 M citric acid and 50 mM HMPA internal standard.

S5.12 Phosphorylated His-His Intermediate

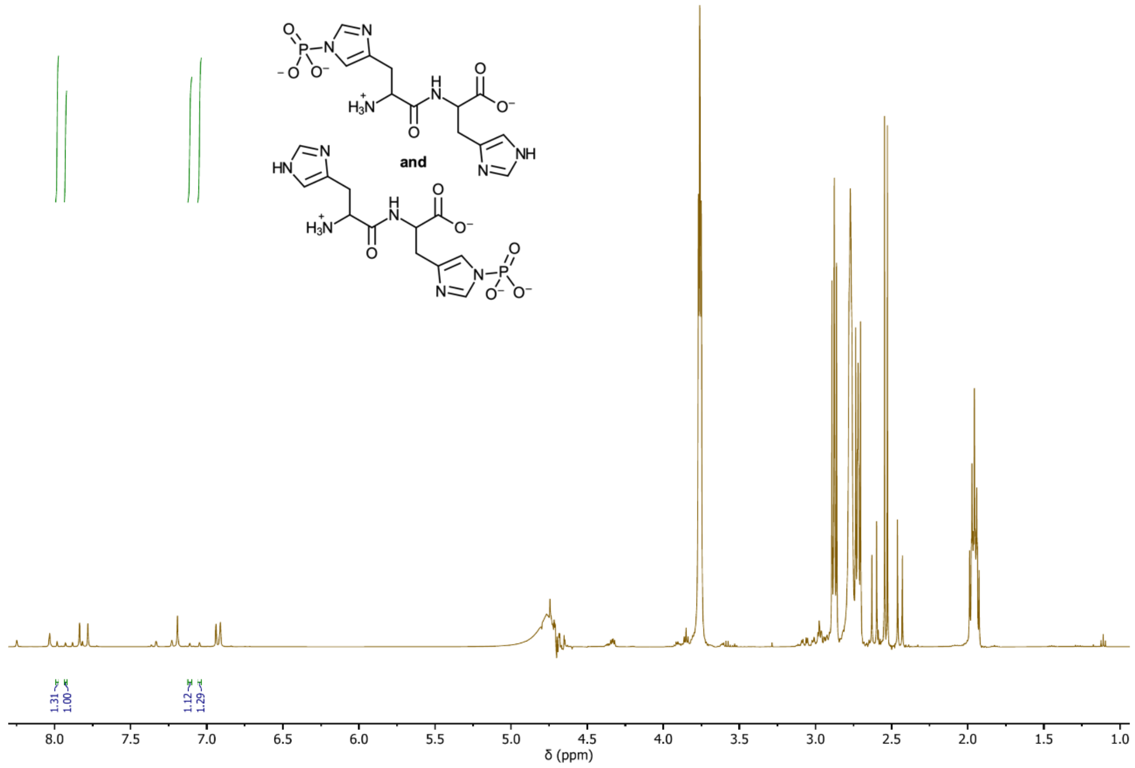

Supporting Figure 331: ^{1}H NMR spectrum of the phosphorylated His-His intermediate. Characterised *in situ* after 48 h starting from a solution of 50 mM His-His and 50 mM calcium imidazole phosphate in 0.5 M MOPS buffer at pH 7.5 in 9 : 1 H_2O : D_2O containing 0.1 M citric acid and 50 mM HMPA internal standard.

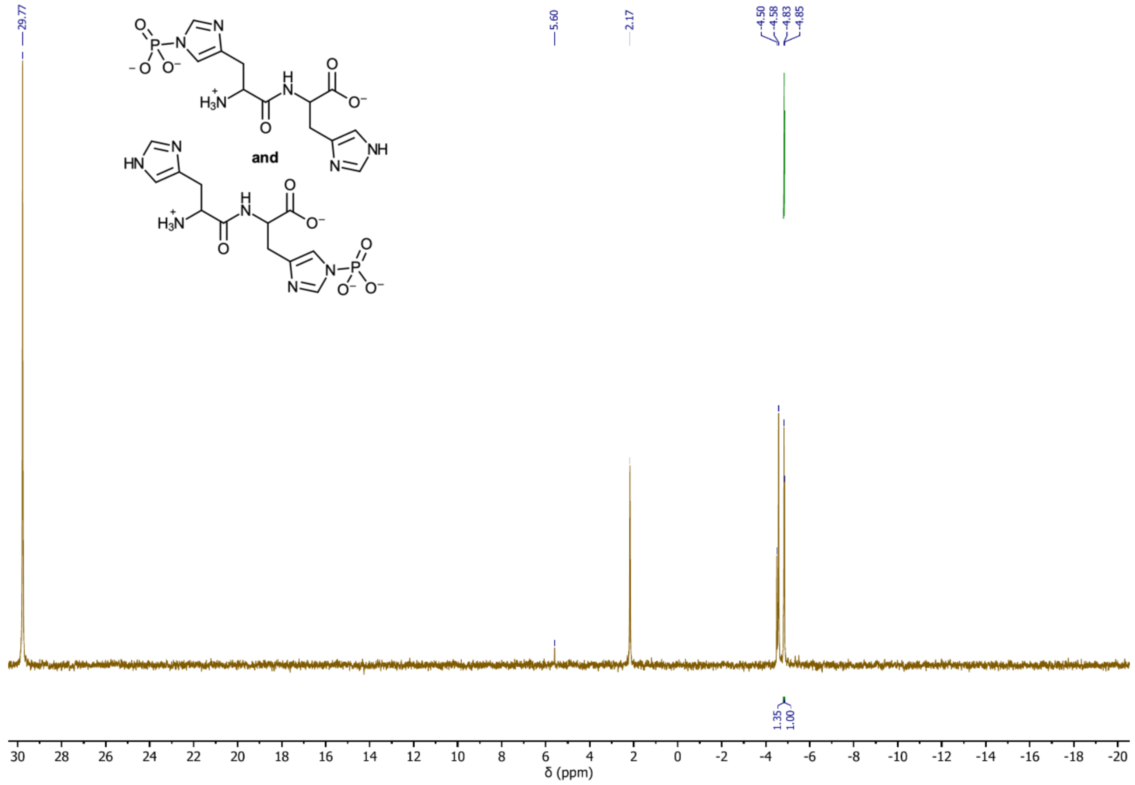

Supporting Figure 332: ^{31}P NMR spectrum of the phosphorylated His-His intermediate. Characterised *in situ* after 48 h starting from a solution of 50 mM His-His and 50 mM calcium imidazole phosphate in 0.5 M MOPS buffer at pH 7.5 in 9 : 1 H_2O : D_2O containing 0.1 M citric acid and 50 mM HMPA internal standard.

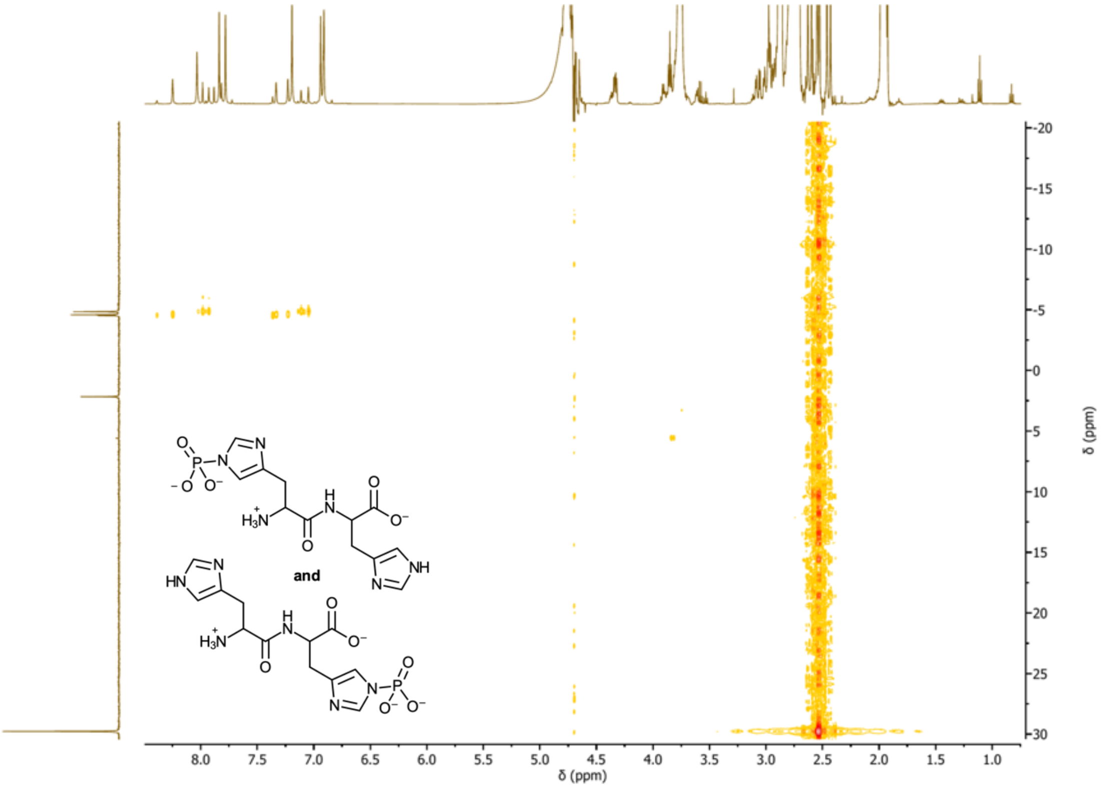

Supporting Figure 333: ^{1}H ^{31}P HMBC spectrum of the phosphorylated His-His intermediate. Characterised *in situ* after 48 h starting from a solution of 50 mM His-His and 50 mM calcium imidazole phosphate in 0.5 M MOPS buffer at pH 7.5 in 9 : 1 H_2O : D_2O containing 0.1 M citric acid and 50 mM HMPA internal standard.

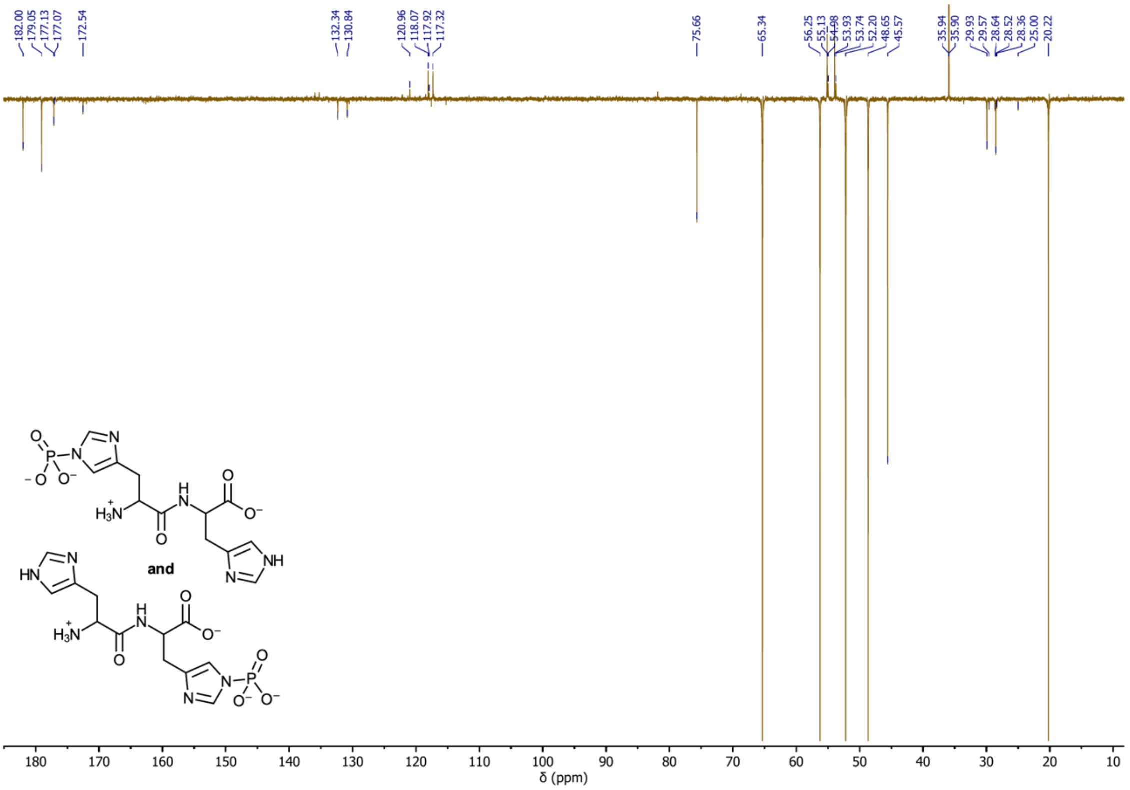

Supporting Figure 334: ^{13}C NMR spectrum of the phosphorylated His-His intermediate. Characterised *in situ* after 48 h starting from a solution of 50 mM His-His and 50 mM calcium imidazole phosphate in 0.5 M MOPS buffer at pH 7.5 in 9 : 1 H_2O : D_2O containing 0.1 M citric acid and 50 mM HMPA internal standard.

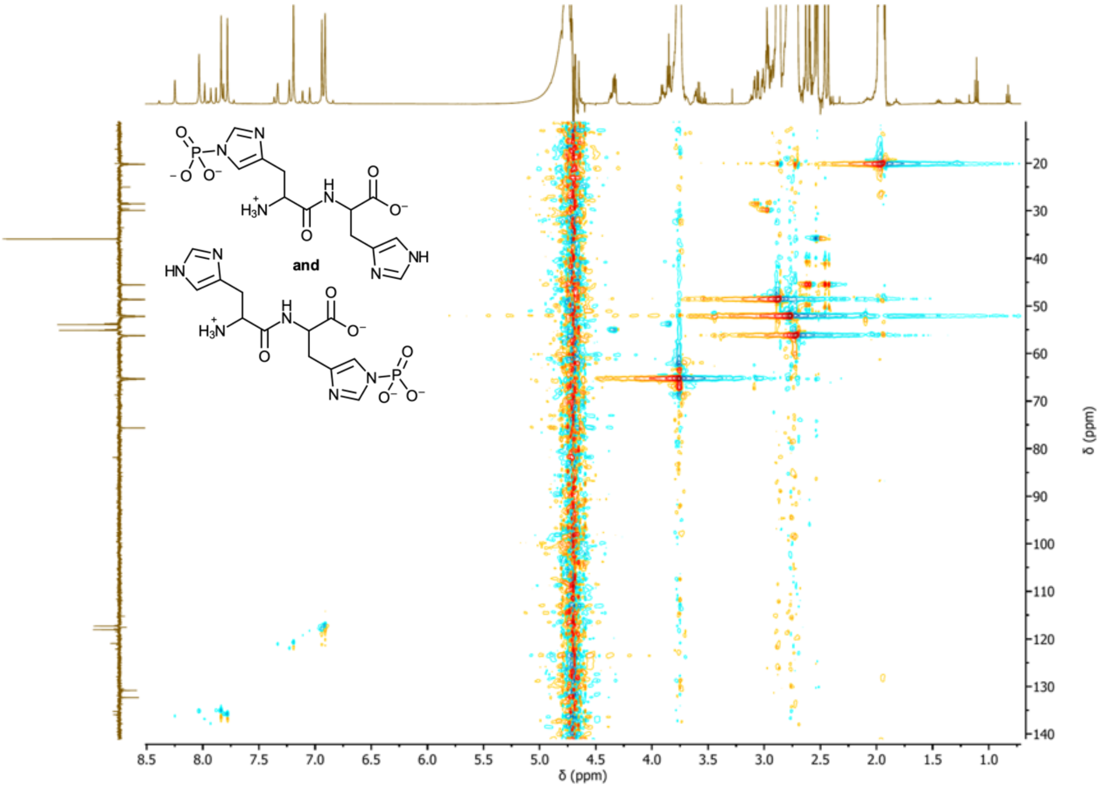

Supporting Figure 335: ^{1}H ^{13}C HSQC spectrum of the phosphorylated His-His intermediate. Characterised *in situ* after 48 h starting from a solution of 50 mM His-His and 50 mM calcium imidazole phosphate in 0.5 M MOPS buffer at pH 7.5 in 9 : 1 H_2O : D_2O containing 0.1 M citric acid and 50 mM HMPA internal standard.

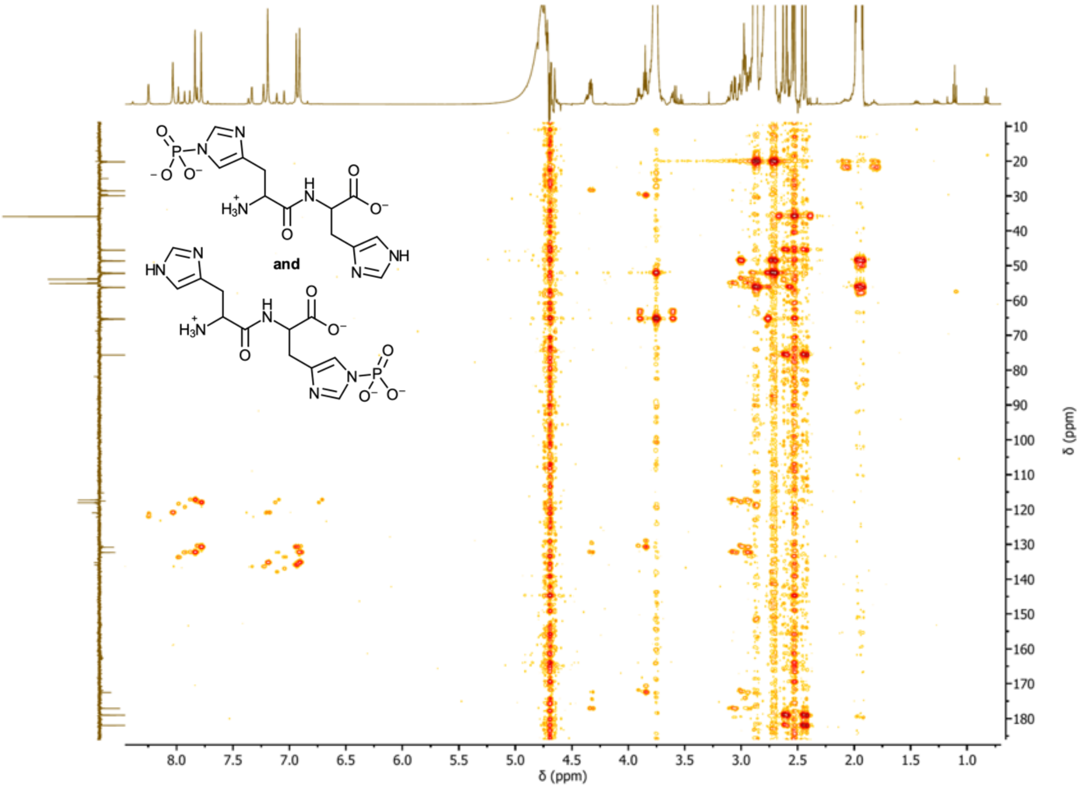

Supporting Figure 336: ^{1}H ^{13}C HMBC spectrum of the phosphorylated His-His intermediate. Characterised *in situ* after 48 h starting from a solution of 50 mM His-His and 50 mM calcium imidazole phosphate in 0.5 M MOPS buffer at pH 7.5 in 9 : 1 H_2O : D_2O containing 0.1 M citric acid and 50 mM HMPA internal standard.

S5.13 Phosphorylated Gly-Gly-His Intermediate

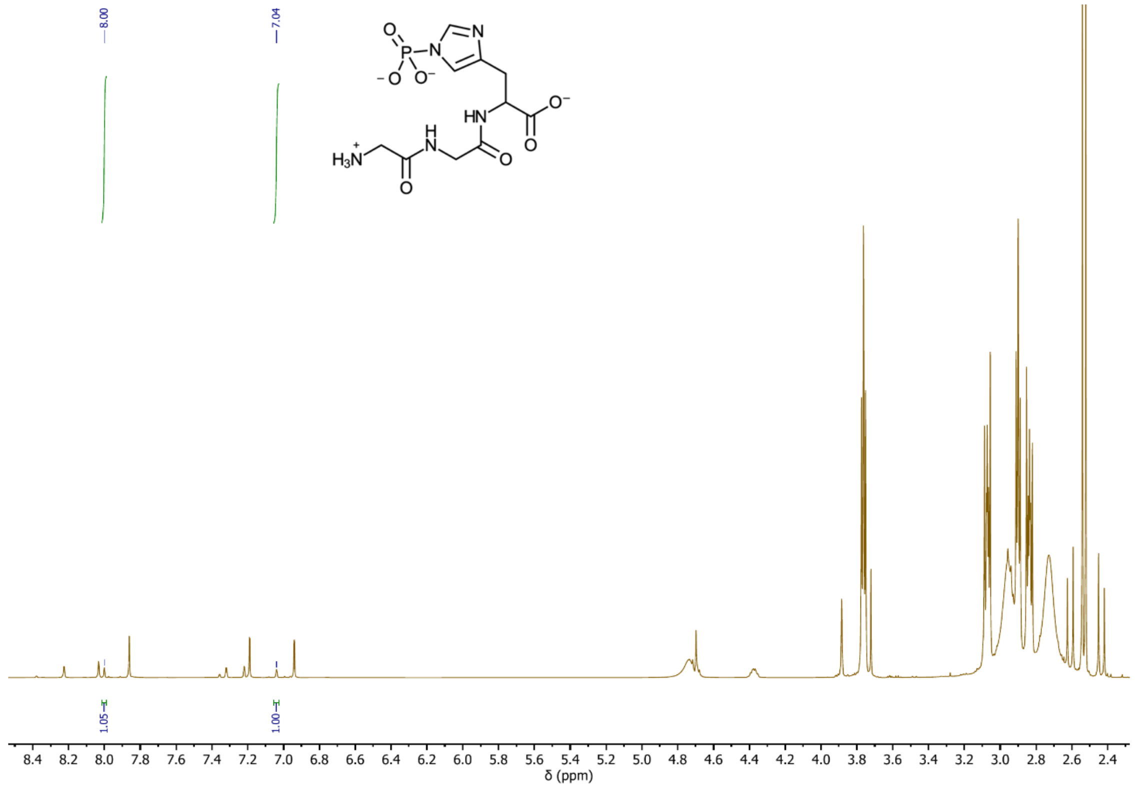

Supporting Figure 337: ^{1}H NMR spectrum of the phosphorylated Gly-Gly-His intermediate. Characterised *in situ* after 48 h starting from a solution of 50 mM Gly-Gly-His and 50 mM calcium imidazole phosphate in 0.5 M MOPS buffer at pH 7.5 in 9 : 1 H_2O : D_2O containing 0.1 M citric acid and 50 mM HMPA internal standard.

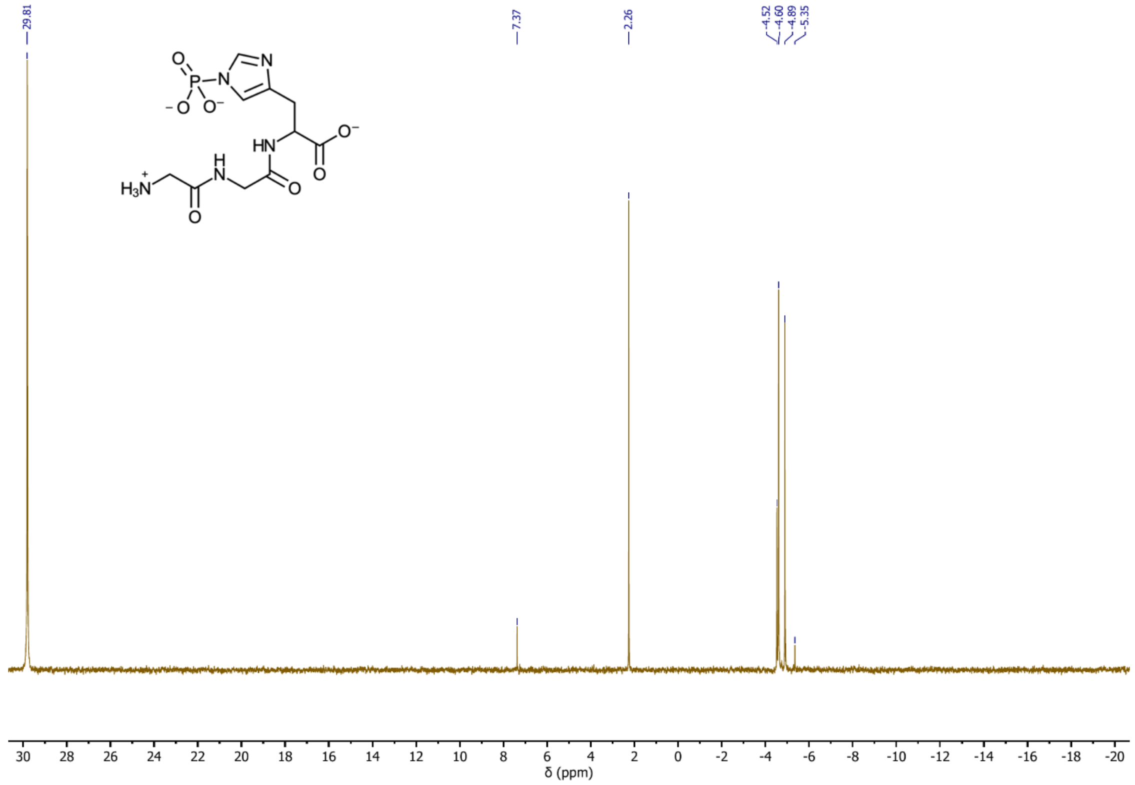

Supporting Figure 338: ^{31}P NMR spectrum of the phosphorylated Gly-Gly-His intermediate. Characterised *in situ* after 48 h starting from a solution of 50 mM Gly-Gly-His and 50 mM calcium imidazole phosphate in 0.5 M MOPS buffer at pH 7.5 in 9 : 1 H_2O : D_2O containing 0.1 M citric acid and 50 mM HMPA internal standard.

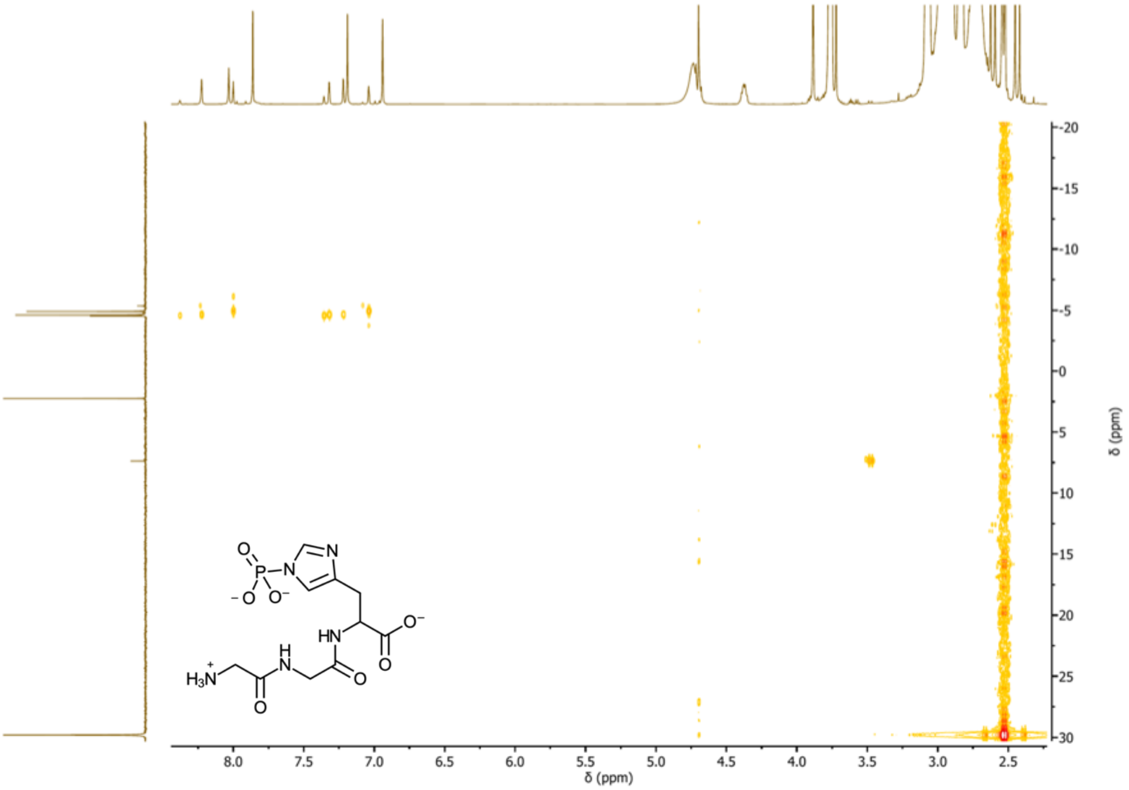

Supporting Figure 339: ^{1}H ^{31}P HMBC spectrum of the phosphorylated Gly-Gly-His intermediate. Characterised *in situ* after 48 h starting from a solution of 50 mM Gly-Gly-His and 50 mM calcium imidazole phosphate in 0.5 M MOPS buffer at pH 7.5 in 9 : 1 H_2O : D_2O containing 0.1 M citric acid and 50 mM HMPA internal standard.

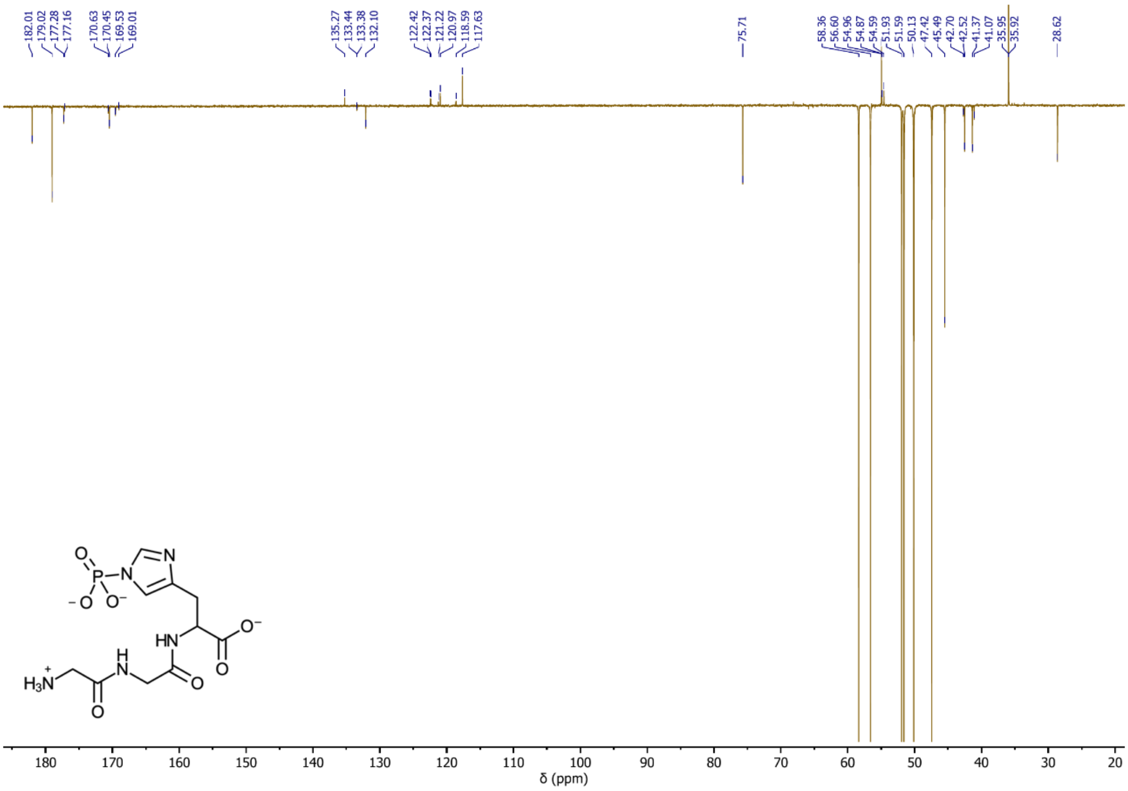

Supporting Figure 340: ^{13}C NMR spectrum of the phosphorylated Gly-Gly-His intermediate. Characterised *in situ* after 48 h starting from a solution of 50 mM Gly-Gly-His and 50 mM calcium imidazole phosphate in 0.5 M MOPS buffer at pH 7.5 in 9 : 1 H_2O : D_2O containing 0.1 M citric acid and 50 mM HMPA internal standard.

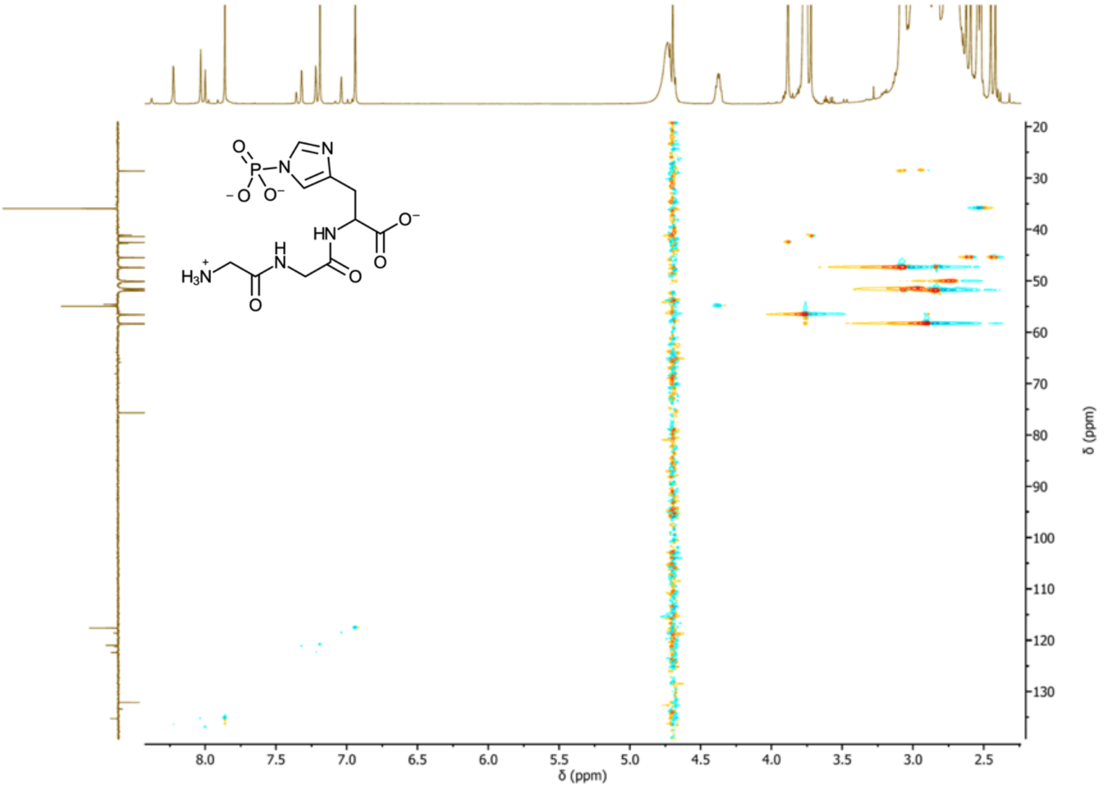

Supporting Figure 341: ^{1}H ^{13}C HSQC spectrum of the phosphorylated Gly-Gly-His intermediate. Characterised *in situ* after 48 h starting from a solution of 50 mM Gly-Gly-His and 50 mM calcium imidazole phosphate in 0.5 M MOPS buffer at pH 7.5 in 9 : 1 H_2O : D_2O containing 0.1 M citric acid and 50 mM HMPA internal standard.

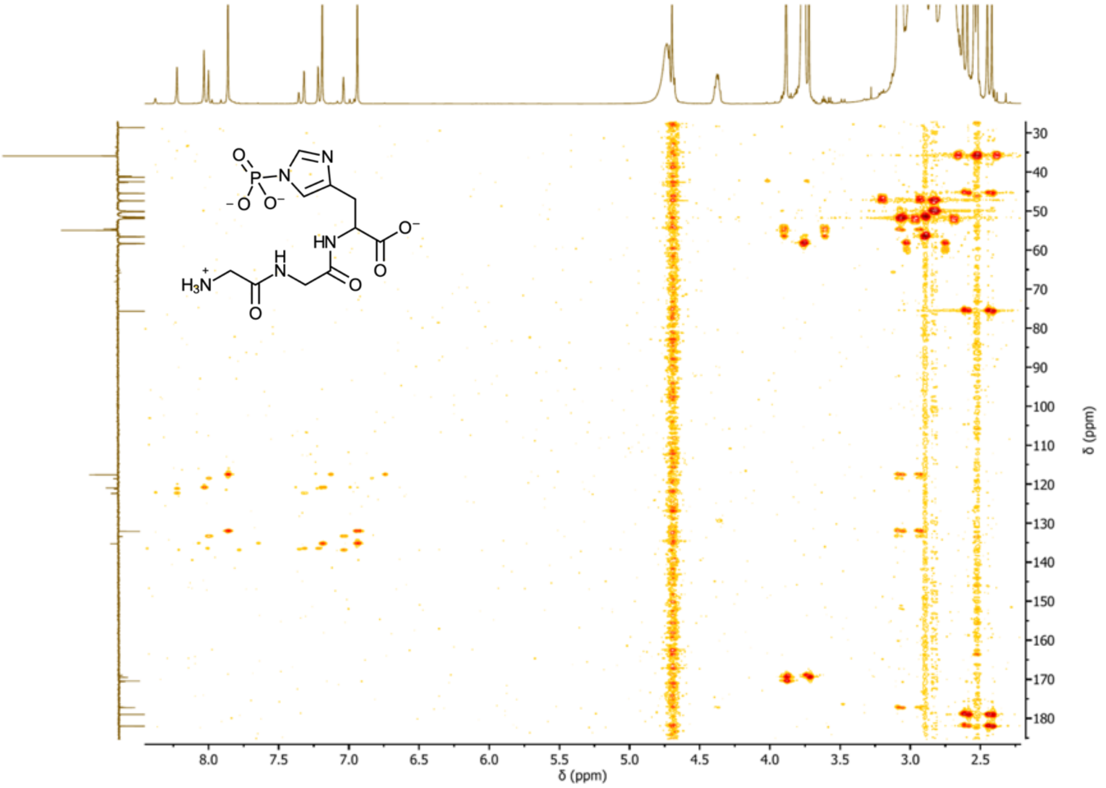

Supporting Figure 342: ^{1}H ^{13}C HMBC spectrum of the phosphorylated Gly-Gly-His intermediate. Characterised *in situ* after 48 h starting from a solution of 50 mM Gly-Gly-His and 50 mM calcium imidazole phosphate in 0.5 M MOPS buffer at pH 7.5 in 9 : 1 H_2O : D_2O containing 0.1 M citric acid and 50 mM HMPA internal standard.

S5.14 Phosphorylated Gly-Lys-His Intermediate

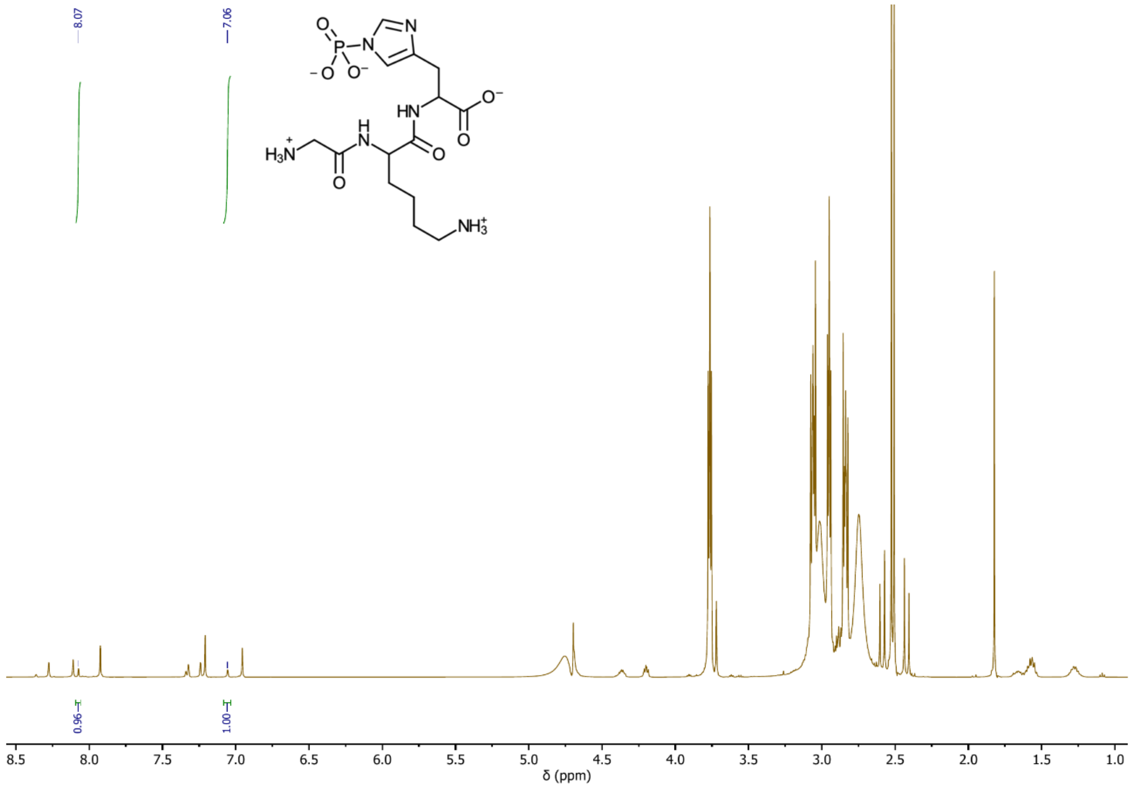

Supporting Figure 343: ^{1}H NMR spectrum of the phosphorylated Gly-Lys-His intermediate. Characterised *in situ* after 48 h starting from a solution of 50 mM Gly-Lys-His and 50 mM calcium imidazole phosphate in 0.5 M MOPS buffer at pH 7.5 in 9 : 1 H_2O : D_2O containing 0.1 M citric acid and 50 mM HMPA internal standard.

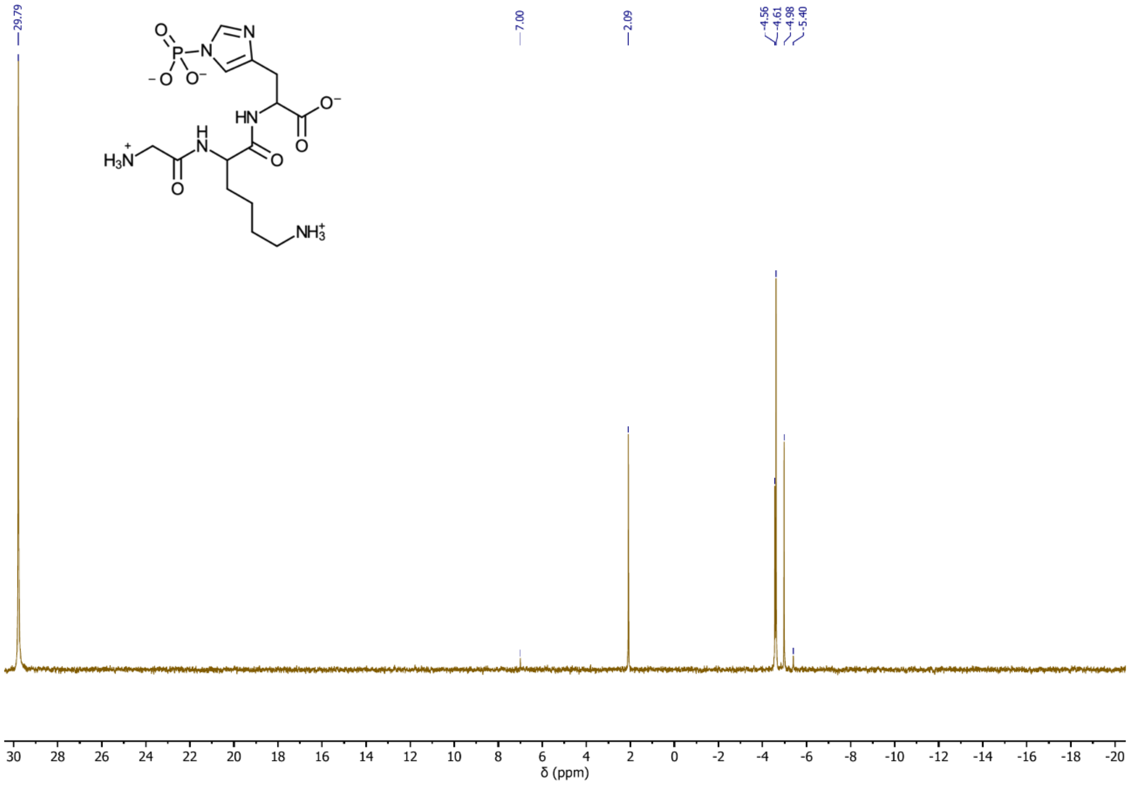

Supporting Figure 344: ^{31}P NMR spectrum of the phosphorylated Gly-Lys-His intermediate. Characterised *in situ* after 48 h starting from a solution of 50 mM Gly-Lys-His and 50 mM calcium imidazole phosphate in 0.5 M MOPS buffer at pH 7.5 in 9 : 1 H_2O : D_2O containing 0.1 M citric acid and 50 mM HMPA internal standard.

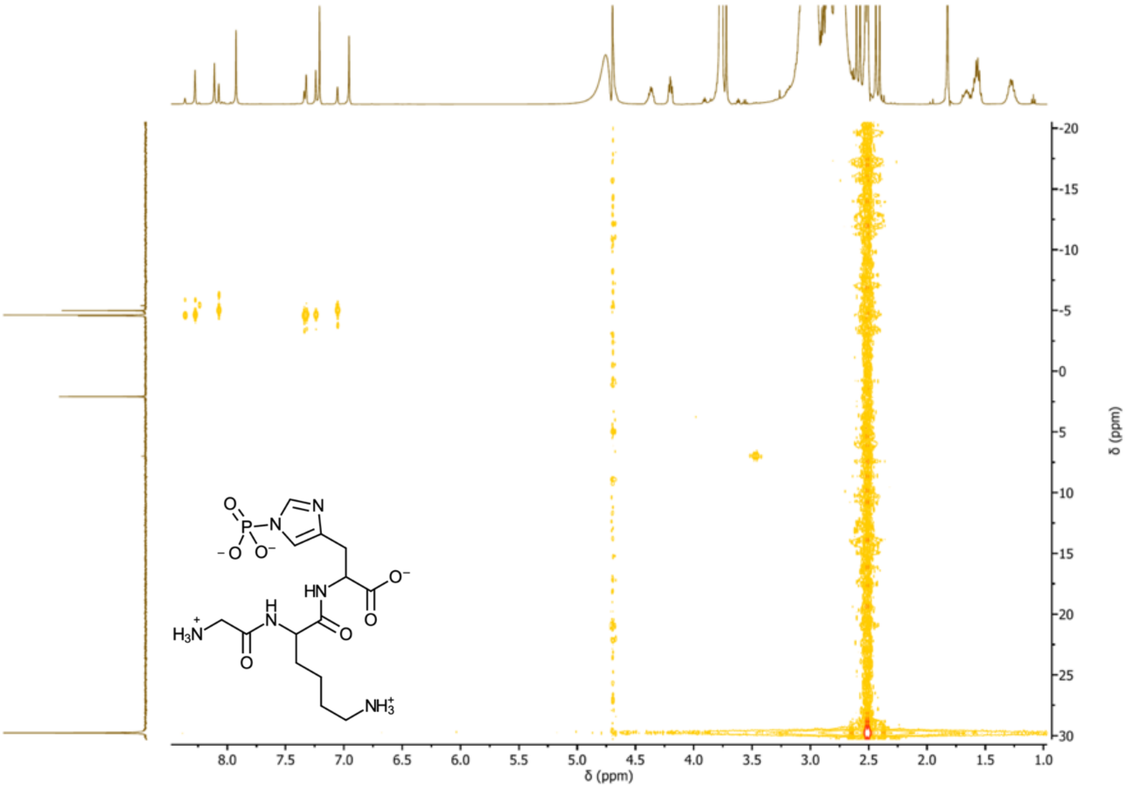

Supporting Figure 345: ^{1}H ^{31}P HMBC spectrum of the phosphorylated Gly-Lys-His intermediate. Characterised *in situ* after 48 h starting from a solution of 50 mM Gly-Lys-His and 50 mM calcium imidazole phosphate in 0.5 M MOPS buffer at pH 7.5 in 9 : 1 H_2O : D_2O containing 0.1 M citric acid and 50 mM HMPA internal standard.

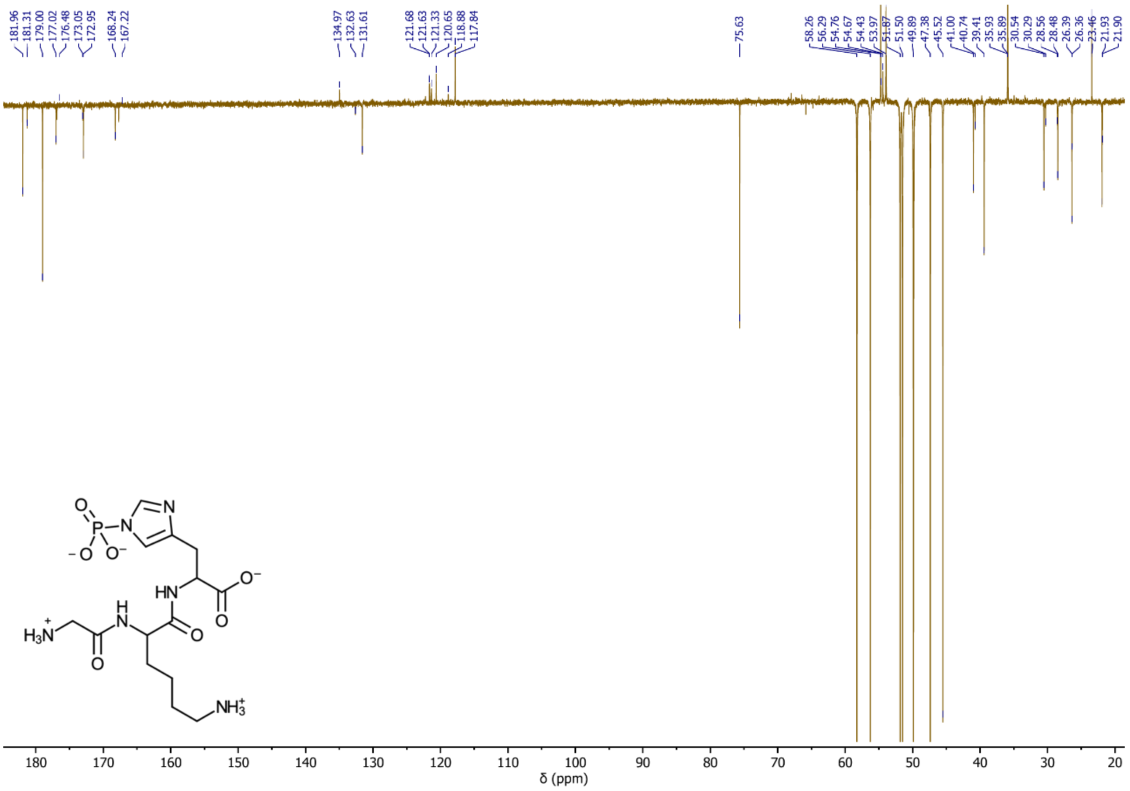

Supporting Figure 346: ^{13}C NMR spectrum of the phosphorylated Gly-Lys-His intermediate. Characterised *in situ* after 48 h starting from a solution of 50 mM Gly-Lys-His and 50 mM calcium imidazole phosphate in 0.5 M MOPS buffer at pH 7.5 in 9 : 1 H_2O : D_2O containing 0.1 M citric acid and 50 mM HMPA internal standard.

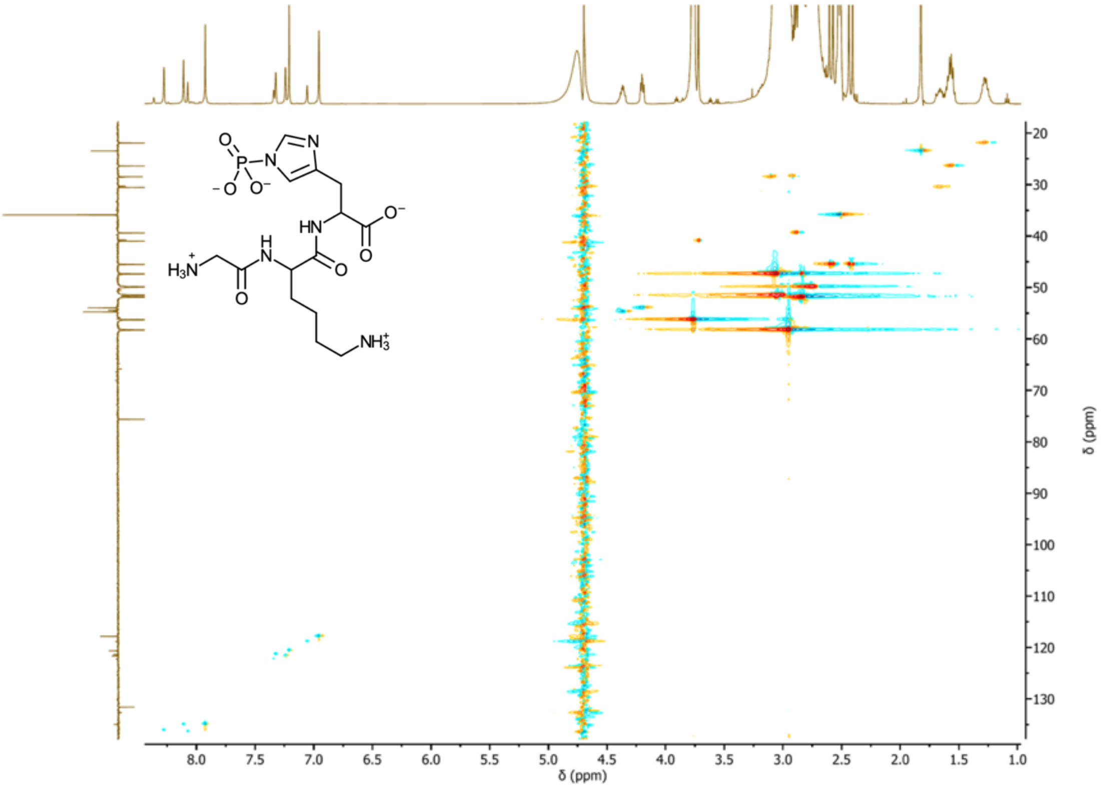

Supporting Figure 347: ^{1}H ^{13}C HSQC spectrum of the phosphorylated Gly-Lys-His intermediate. Characterised *in situ* after 48 h starting from a solution of 50 mM Gly-Lys-His and 50 mM calcium imidazole phosphate in 0.5 M MOPS buffer at pH 7.5 in 9 : 1 H_2O : D_2O containing 0.1 M citric acid and 50 mM HMPA internal standard.

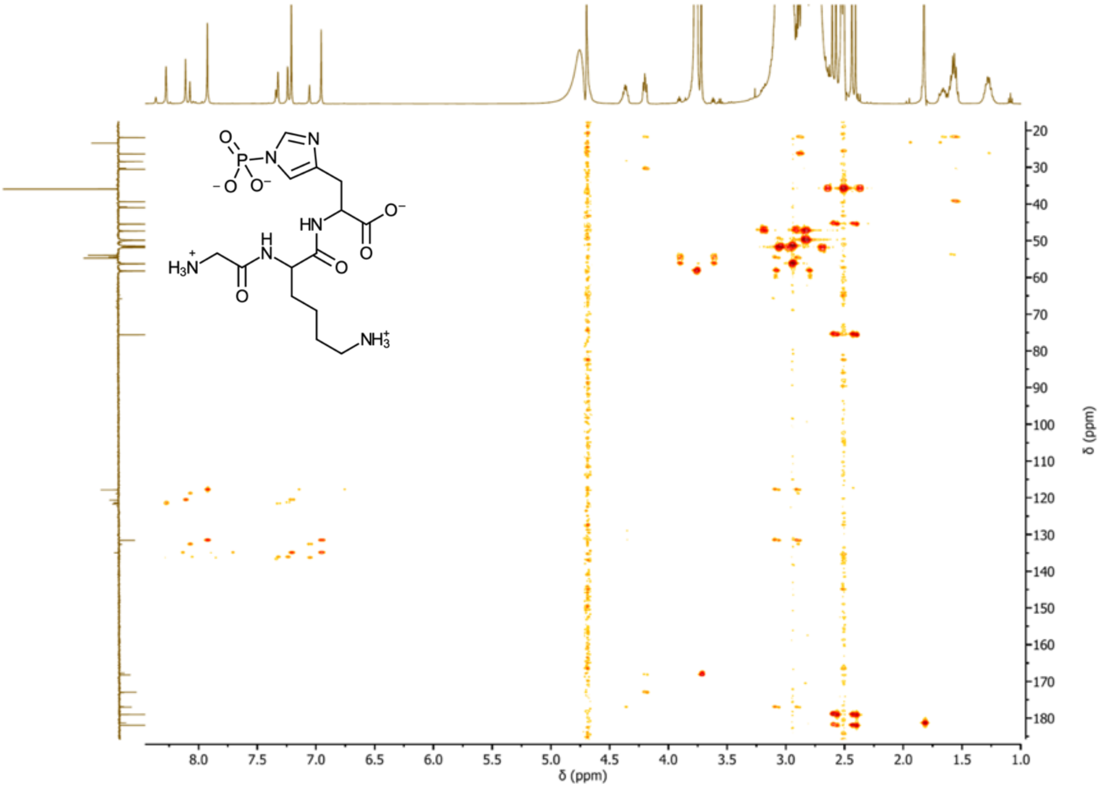

Supporting Figure 348: ^{1}H ^{13}C HMBC spectrum of the phosphorylated Gly-Lys-His intermediate. Characterised *in situ* after 48 h starting from a solution of 50 mM Gly-Lys-His and 50 mM calcium imidazole phosphate in 0.5 M MOPS buffer at pH 7.5 in 9 : 1 H_2O : D_2O containing 0.1 M citric acid and 50 mM HMPA internal standard.

S6 Supporting References

1. Maguire, O. R., Smokers, I. B. A. & Huck, W. T. S. A physicochemical orthophosphate cycle via a kinetically stable thermodynamically activated intermediate enables mild prebiotic phosphorylations. *Nat. Commun.* **12,** 5517 (2021).
2. Roelfs , M. & Kroon, P. C. *SymFit*
